# A quantitative view of the transcriptome of *Schistosoma mansoni *adult-worms using SAGE

**DOI:** 10.1186/1471-2164-8-186

**Published:** 2007-06-21

**Authors:** Elida PB Ojopi, Paulo SL Oliveira, Diana N Nunes, Apuã Paquola, Ricardo DeMarco, Sheila P Gregório, Karina A Aires, Carlos FM Menck, Luciana CC Leite, Sergio Verjovski-Almeida, Emmanuel Dias-Neto

**Affiliations:** 1 Laboratório de Neurociências (LIM27), Instituto de Psiquiatria, Faculdade de Medicina da Universidade de São Paulo; R. Dr. Ovídio Pires de Campos, 785 – 3 rd floor, 05403-010, São Paulo, SP, Brazil; 2 Laboratory of Genetics and Molecular Cardiology, Heart Institute (InCor), Hospital das Clínicas da Faculdade de Medicina da Universidade de São Paulo, São Paulo, SP, Brazil; 3 MD Anderson Cancer Center, University of Texas – 1515 Holcombe Blvd, Unit 1374, 77030 – Houston, TX, USA; 4 Departamento de Bioquímica, Instituto de Química, Universidade de São Paulo, 05508-900, São Paulo, SP, Brazil; 5 Departamento de Microbiologia, Instituto de Ciências Biomédicas, Universidade de São Paulo, Av. Prof. Lineu Prestes, 1374, 05508-900, São Paulo, SP, Brazil; 6 Centro de Biotecnologia, Instituto Butantan, 05503-900, São Paulo, SP, Brazil

## Abstract

**Background:**

Five species of the genus Schistosoma, a parasitic trematode flatworm, are causative agents of Schistosomiasis, a disease that is endemic in a large number of developing countries, affecting millions of patients around the world. By using SAGE (Serial Analysis of Gene Expression) we describe here the first large-scale quantitative analysis of the Schistosoma mansoni transcriptome, one of the most epidemiologically relevant species of this genus.

**Results:**

After extracting mRNA from pooled male and female adult-worms, a SAGE library was constructed and sequenced, generating 68,238 tags that covered more than 6,000 genes expressed in this developmental stage. An analysis of the ordered tag-list shows the genes of F10 eggshell protein, pol-polyprotein, HSP86, 14-3-3 and a transcript yet to be identified to be the five top most abundant genes in pooled adult worms. Whereas only 8% of the 100 most abundant tags found in adult worms of S. mansoni could not be assigned to transcripts of this parasite, 46.9% of the total ditags could not be mapped, demonstrating that the 3 sequence of most of the rarest transcripts are still to be identified. Mapping of our SAGE tags to S. mansoni genes suggested the occurrence of alternative-polyadenylation in at least 13 gene transcripts. Most of these events seem to shorten the 3 UTR of the mRNAs, which may have consequences over their stability and regulation.

**Conclusion:**

SAGE revealed the frequency of expression of the majority of the S. mansoni genes. Transcriptome data suggests that alternative polyadenylation is likely to be used in the control of mRNA stability in this organism. When transcriptome was compared with the proteomic data available, we observed a correlation of about 50%, suggesting that both transcriptional and post-transcriptional regulation are important for determining protein abundance in S. mansoni. The generation of SAGE tags from other life-cycle stages should contribute to reveal the dynamics of gene expression in this important parasite.

## Background

Quantitative and qualitative transcriptome analyses reveal some of the most important biological aspects of an organism. Transcriptome examination is crucial for the understanding of significant biological processes, allowing the study of transcription/translation relationships, the dynamics of gene expression and, an important feature in parasites, a quantitative evaluation of the expression of genes that are potential targets for drugs or vaccines across diverse life-cycle or developmental stages.

Large-scale transcriptome analysis of *S. mansoni *has been mainly performed by the partial sequencing of cDNA clones derived from libraries prepared with RNA derived from diverse life-cycle stages of the parasite [1-4]. The largest collection of ESTs sequenced for this parasite was published by our group [5], where we used cDNA normalization techniques that greatly contributed to gene discovery but are not adequate for quantitative analysis. Large-scale quantitative transcriptome analysis in this parasite has been performed by using cDNA/oligo microarrays for evaluating differences in gene expression among different gender [6-9] or life-cycle stages [10,11]. However, the quantitative analysis obtained by microarrays is not absolute, and the interpretation of the findings is limited by the genes that have been spotted.

Serial Analysis of Gene Expression [12] is one of the most comprehensive approaches to a large-scale transcriptome analysis and, together with cDNA microarray and other techniques, is capable of contributing to a global analysis of gene expression. SAGE permits a quantitative view of a transcriptome, through the generation and sequencing of short nucleotide tags that allow the identification of the corresponding genes, enabling a direct estimation of their frequencies. An important feature of SAGE is its ability to determine the expression of all genes that contain the recognition site of the restriction enzyme used (a four bp cutter), and thus is not limited to the genes that have been used to construct the arrays. As a consequence SAGE simplifies data expression analysis among different experiments, as the data provided reflects a direct measure of gene expression and permits a direct comparison of libraries generated by different groups. SAGE has been used for gene-expression analysis in a series of organisms including *Rattus norvegicus *[13], *Saccharomyces cerevisiae *[14], *Homo sapiens *[15], *Mus musculus *[16], *Caenorhabditis elegans *[17], *Drosophila melanogaster *[18], *Cryptococcus neoformans *[19] and many others. Regarding human parasites, up to now studies have been performed only for *Plasmodium falciparum *[20-22] and more recently for *Giardia lamblia *[23] and *Toxoplasma gondii *[24]. Here we report the results of the first SAGE-library prepared from the adult stage of the parasitic flatworm *Schistosoma mansoni*.

## Methods

### Parasites, mRNA extraction and SAGE

Pooled (male and female) adult worms from BH isolate of *S. mansoni *were maintained in the laboratory by routine passage through mice and snails and recovered from the porto-mesenteric system by perfusion, after 7 to 8 weeks of infection. Worms were washed in saline solution and stored at -20°C in RNAlater (Ambion) prior to mRNA extraction. Poly-A mRNA was isolated with MACS kit (Miltenyi Biotec Auburn, CA, USA), eluted in 200 μL of DEPC-treated water and treated twice with Promega RQ1 RNAse-free DNAse (1 U/10 μL) for 30 min at 37°C. DNAse was inactivated at 65°C for 10 min. mRNA purity and integrity were checked by RT-PCR using appropriate primer pairs of known genes and also negative controls as described in Verjovski-Almeida et al. [5]. Ninety nanograms of poly-A+ mRNA were used for the construction of a SAGE library, according to the standard I-SAGE Kit protocol (Invitrogen, USA). After size-selection, concatamers were cloned into pZERO-1 and sequenced using standard dye terminator techniques.

### Bioinformatic analysis

Sequences from cloning vectors were trimmed and tags were extracted from high-quality segments using Phred [39]. Sequences with Phred-scores bellow 20, as well as identical ditags (which are likely to be the result of amplification or cloning artifacts) were excluded from further analysis. The remaining tags were ordered in a list according to their frequency.

A second list, containing putative SAGE tags of *S. mansoni *genes was generated *in silico *after mapping the *Nla*III restriction sites (CATG) to the complete set of full-length cDNA sequences from *S. mansoni *available from GenBank, from the TIGR tentative consensus and the complete set of clusters and singlets generated by our group as part of the *S. mansoni *transcriptome project [5]. Sequences from the three above-mentioned databases were merged to eliminate the redundancy of transcripts. The downstream 10 nt sequence that was adjacent to each *Nla*III restriction site in the transcripts dataset was extracted, thus generating a list of putative *S. mansoni *tags. These tags were annotated according to the information available for the transcripts from which they were derived. Top priority annotation was given to full-length genes, followed by TIGR consensus and our *S. mansoni *transcriptome project [5]. These tags were then cross-referenced with the tag list derived from our SAGE library, enabling the definition of the most abundant genes in adult worms.

Full length *S. mansoni *transcripts were also screened for putative alternative poly-adenylation sites using SAGE data. For this purpose, the list containing all putative SAGE-tags (adjacent to *NlaIII *sites) from *S. mansoni *full-length genes available in GenBank, was cross-referenced with the tag list and the putative tags and ranked according to their position in relation to the 3' end. The most 3' tags, that are more likely to be *bona fide *tags for the canonical transcripts, were ranked as zero and the remaining tags were organized in ascending order from 3' to 5'. Tags that have rank > 0, a number of counts > 1, and were not followed by a putative site of internal binding of an oligo-dT primer (at least 8 adenines in a window of 10 bases) [28] were considered as indicative of putative poly-adenylation.

Evaluation of positional distribution of SAGE tags and ESTs over S. mansoni full-length cDNAs was carried over a set of 208 genes that were tagged by at least two SAGE tags. Blast analyses showed 26,888 ESTs and 9,589 SAGE tags mapping to these genes, allowing the identification of gene regions covered by these sequences. The mapped coordinates were normalized in terms of relative position of EST over the mRNA and relative coverage over all genes was calculated. This positional distribution was plotted together the distribution of the SAGE tags over the same gene set, where the 0% and 100% are equivalent to 5' and 3' positions of mRNAs, respectively.

Functional classification of S. mansoni transcripts was undertaken using the Gene Ontology database. For this, blast analyses of the genes mapped by our SAGE tags were performed against 2,413,334 protein sequences available from Gene Ontology database (02/2007). All ontologies associated to the first hit matched by the query sequence were recovered and then was assumed that S. mansoni gene would have the same functional annotation. Evaluations of function were performed for 3 different classes of abundance including: abundant (represented by more than 500 tags), intermediate (499 to 100 tags) and less abundant (lower than 100 tags).

## Results

After sequencing and evaluating 5,626 clones of the SAGE library, 4,752 reads (84%) containing 998,200 nucleotides were accepted with the quality criteria adopted. The need for further sequencing was determined by evaluating the frequency of tags that appeared at least twice as a function of total tags sequenced. This curve reached a plateau close to 60,000 tags and suggested coverage of the majority of genes expressed in this developmental stage [5] (Additional File 3). After vector trimming and removal of identical ditags, a total of 68,238 tags (15,655 distinct tags) remained.

The most informative tags are those that appeared at least twice (less likely to contain sequencing artifacts) in the final tag list. These comprised a total of 6,263 distinct tags, which should approximate to the total number of genes expressed in this developmental stage [5]. The list of tags that appeared only once (N = 9,392) may include a number of sequencing artifacts, but also contains the most rare transcripts of *S. mansoni *adult worms. In fact, 2,886 of these tags found matches in the Schistosome gene index or in the list of *S. mansoni *transcripts identified by Verjovski-Almeida et al. [5], which strongly supports a very low expression of those genes in this developmental stage.

Preliminary gene assignments were performed for the tags using parasite full-length genes available in GenBank at the NCBI (nr), followed by TIGR consensus sequences and a clustering of the sequences produced by the *S. mansoni *transcriptome project [5]. By using this approach, 48 out of the top 50 most abundant tags could be assigned to specific transcripts (Table 1). The non gene-assigned tags of this top list appear as the 41^st ^and 46^th ^most abundant transcripts in adult worms. The most frequent transcript encodes the F10 eggshell protein, followed by a pol-polyprotein transcript, heat shock protein 86 and 14-3-3 protein homolog (see Table 1 for a list of the top 50 transcripts). A total of 6,233 tags (39.9%) have identity to *S. mansoni *gene fragments (contigs and singlets). When only tags that appeared at least twice were considered, 3,347 (53.4%) matched *S. mansoni *gene fragments. A complete list of all tags, together with their frequency, tag sequences, and respective gene assignments can be found in the supplementary table that accompanies this paper (see Additional file 1).

**Table 1:** The 50 most abundant transcripts, revealed by SAGE analysis, in *S. mansoni *adult worms.

	**Gene name**	**Tag sequence**	**Number of tags**	**Gene/cluster identification – Accession number**	**Position inside transcript**	**Tag rank**
1	*S.mansoni *eggshell protein (F10) gene	actattcggg	1454	gi| 160993| gb| M14309.1| SCMFSPA	50	0
2	*S. mansoni *pol-polyprotein	cctgtaaact	835	gi| 44829167| tpg| DAA04497.1| C200397.1	99	0
3	*S.mansoni *heat shock protein 86 mRNA	gaagaagtgg	640	gi| 161027| gb| J04017.1| SCMHSP86	96	0
4	*S. mansoni *14-3-3 protein (Sm14-3-3) mRNA	tcatacaaga	588	gi| 790657| gb| U24281.1| SMU24281	95	0
5	Similar to *S. japonicum *L23a ribosomal protein	ccggtgtctc	513	TC13660	97	0
6	No matches	agcgtccaaa	472	TC7406	85	0
7	*S. mansoni *heat shock protein 70 (HSP70) gene	aggcaagtgg	464	gi| 161025| gb| L02415.1| SCMHSP70X	67	0
8	*S. mansoni *(GAPDH)	cataatgaag	438	gi| 160994| gb| M92359.1| SCMGAPDH	89	1
9	*S. mansoni *fructose bisphosphate aldolase mRNA	cggctcagga	398	gi| 2598925| gb| AF026805.1| AF026805	72	0
10	Mitochondrial small subunit ribosomal RNA	gtagtgcttg	364	AF130787_1459_201	94	0
11	No matches	cttttctaaa	349	TC17835	93	0
12	Similar to *S. japonicum *SJCHGC02196 protein	tgttgttcgt	340	TC17184	96	0
13	*S.mansoni *(Liberia) mRNA for tandem repeat	agcaatggaa	319	gi| 454257| emb| Z29960.1| SMTANREP TC7340	20	3
14	*Similar to SJCHGC06305 protein (putative piruvate kinase)*	tgtacgtcat	313	*gi| 56757978| gb| AAW27129.1| TC7454*	94	2
15	*S. mansoni *fatty acid binding protein mRNA	tatcgttcta	302	gi| 160983| gb| M60895.1| SCMFABP14	89	0
16	Similar to unknown protein *S. japonicum *(putative S3a ribosomal protein)	gcgagtcgaa	293	gi| 56758226| gb| AAW27253.1| TC16783	86	0
17	Similar to *S. japonicum *SJCHGC00690 protein. Putative beta thymosin	taatatgcgc	265	gi| 76161984| gb| AAX30141.2| TC11200	71	0
18	Similar to *S. japonicum *Sj-Ts1	gtgctcgaag	255	gi| 14581393| gb| AAF98445.1| TC17397	53	0
19	*S. mansoni *28 kDa glutathione S-transferase (GST) gene	tgactgatct	254	gi| 161010| gb| M98271.1| SCMGSTM	88	1
20	*S. mansoni *tegumental protein Sm 20.8 mRNA	gcacattgtc	252	gi| 2454222| gb| U91941.1| U91941	62	0
21	*S. mansoni *actin mRNA	acatcaacaa	225	gi| 924602| gb| U19945.1| SMU19945	84	0
22	similar *S. japonicum *(putative ribosomal protein L15)	ccttcggtac	225	gi| 29841092| gb| AAP06105.1| TC13745	97	0
23	Similar to SJCHGC09089 *S. japonicum *(putative ribosomal protein S10)	gaggttatgg	215	gi| 56755876| gb| AAW26116.1| TC16990	89	0
24	Similar to *S. japonicum *clone ZZD545 mRNA sequence	ttggaggcaa	211	gi| 28317769| gb| AY223294.1| TC13521	93	0
25	*S.mansoni *SM22.6 antigen (A12) RNA	gagaacacca	198	gi| 161086| gb| M37003.1| SCMSM226	71	0
26	Similar to SJCHGC01209 *S. japonicum *(putative tetraspanin)	gtaaccaatg	193	gi| 56752993| gb| AAW24708.1| TC11060	93	0
27	Similar to *S. japonicum *(putative hnRNP A2)	cagcgtcctt	191	gi| 29841163| gb| AAP06176.1| TC10489	78	3
28	Similar to SJCHGC06078 *S. japonicum *(putative 40S ribosomal protein)	gggattgccg	190	gi| 56758252| gb| AAW27266.1| TC16808	87	0
29	*S. mansoni *receptor for activated PKC mRNA	gtctgctgat	186	gi| 19071248| gb| AF422164.1|	87	0
30	No matches	ccaggttgtg	184	TC9014	0	0
31	Similar to *S. japonicum *HEXBP (putative DNA binding protein)	gttatggcca	174	gi| 29841170| gb| AAP06183.1| TC10354	75	0
32	Similar to S.*japonicum *SJCHGC05715 (putative germinal Histone H4)	tggattcttg	168	gi| 56754980| gb| AY813941.1| TC14578	81	0
33	*S. mansoni *myosin heavy chain (MYH) mRNA	gtacttagtg	167	gi| 161043| gb| L01634.1| SCMMYH	97	0
34	*S. mansoni *lactate dehydrogenase	tatgttctct	165	gi| 4099443| gb| U87629.1| SMU87629	87	0
35	NADH dehydrogenase 4 (ND4) gene	attttgtttg	159	AF130788_1003_2262	87	0
36	*S. mansoni *cathepsin L	gggtatgaat	159	gi| 473158| emb| Z32529.1| SMCATHL	87	2
37	Similar to *S. japonicum *(putative 60S acidic ribosomal protein P0)	ggattcggtt	155	gi| 29841185| gb| AAP06198.1| TC7332	95	0
38	*S. mansoni *cDNA clone (putative 60S acidic ribosomal protein P2)	ccatcagcct	154	gi| 34701209| gb| CD164545.1| CD164545 CD164545	92	0
39	Similar to *S. japonicum *putative L10 ribosomal protein	gccccttgga	154	TC13587	87	0
40	*S. mansoni *enolase trans-spliced mRNA	tcgttctgat	147	gi| 1002615| gb| U30175.1| SMU30175	84	0
41	Non-available	tccccgtaca	145	**no_annot**		
42	*S. mansoni *cysteine protease inhibitor (Cys) mRNA	cccaccactt	144	gi| 33355622| gb| AY334553.1|	84	0
43	Similar to *S. japonicum *cDNA clone (putative ribosomal protein L10)	tacctaggcc	143	gi| 29841379| gb| AAP06411.1| TC7615	79	0
44	Similar to *S. japonicum *SJCHGC01239 protein (putative L8 ribosomal protein)	gtcgcaaagt	139	gi| 56754665| gb| AAW25518.1| TC7475	59	1
45	*S. mansoni *NADH dehydrogenase subunit 5 (NU5M) mRNA	ggtttagtag	138	gi| 3599492| gb| AF085145.1| AF085145	90	0
46	Non-available	tgcgcgcgtg	137	**no_annot**		
47	Similar to *S. japonicum *SJCHGC02603 Protein (putative 40S ribosomal protein S27)	cacagacagc	134	gi| 60687866| gb| AAX30266.1| TC8025	55	0
48	*S. mansoni *clone NLSL20 40S rRNA protein homolog mRNA	tggtgagggc	130	gi| 2623827| gb| AF030961.1| AF030961 TC13698	46	0
49	Similar to *S. japonicum *SJCHGC01998 protein (putative 40S ribosomal protein S25)	ctctcgagga	128	gi| 76162217| gb| ABA40776.1| TC7478	70	0
50	Similar to *S. japonicum *SJCHGC00821 protein (putative myosin regulatory light chain 2A)	ggagaagaaa	123	gi| 56757579| gb| AAW26951.1| TC7368	3	3

In order to evaluate the functional categories most abundantly represented in the transcriptome of *S. mansoni*, blast analyses were performed against 2,413,334 protein sequences available from the Gene Ontology database (Feb/2007). Genes mapped by more than 3 SAGE tags were used as queries. All ontologies associated to the first hit matched by the query sequences were recovered and their functional annotations were given to the respective schistosome gene. In this process, ontologies were assigned to 2,933 genes. Functional classification was then investigated for transcripts distributed in expression classes, according to their tag abundance. We considered that the most abundant functional categories were those containing genes with more than 500 tags; followed by the intermediate (499 to 100 tags) and less abundant classes (lower than 100 tags). This allowed us to describe the most abundant functional classes among the highly expressed, intermediate and lower expressed genes.

**Figure 1:**
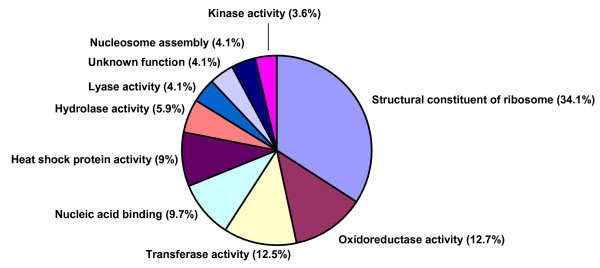
**Gene Ontology analysis of the most abundant proteins classes in adult worms of *Schistosoma mansoni***. Functional classification *S. mansoni *protein groups containing more than 500 tags/functional class.

It can be observed in Figure 1, that the most abundant genes fall into few Gene Ontology functional categories. By far the largest group comprises genes coding for proteins involved in the structural constitution of the ribosome (34.1%), indicating intense protein synthesis activity. These, together with enzymes involved in  DNA or protein modification, nucleic acid binding, nucleosome assembly and kinase activity add more than 2/3 of the highly expressed genes and should be clearly involved in the regular metabolism of the worm. On the other hand, a high expression of genes encoding heat shock proteins and oxidoreductases also comprise a large proportion of the transcriptome indicating the importance of defense mechanisms of the parasite against the potential stress involved in its adaptation to interaction with the host immune system. As expected the proportion of highly expressed genes that have unknown functions is low and, as we move into the intermediate and then less abundant classes (see Additional file 4), the diversity of functional categories increases and so does the proportion of genes involved with functions still to be determined. Within the intermediate class we have several genes coding for proteins that have been investigated as vaccine candidates, such as the membrane proteins Sm20.8 and Sm22.6, or cathepsin and superoxide dismutase.

The list of all putative SAGE-tags that mapped to known full-length mRNA sequences of *S. mansoni *shows that for some transcripts two or more distinct SAGE tags have been sequenced. These distinct tags were used to investigate alternative poly-adenylation events that may occur in these transcripts. After the analysis of these events, using the criteria described in materials and methods at least 16 alternative poly-adenylation events could be identified in 13 full-length *S. mansoni *mRNAs (Table 2).

**Table 2:** Transcripts with putative alternative poly-adenylation events in *S. mansoni*, as suggested by SAGE.

**Gene (Accession GenBank)**	**mRNA size (nt)**	**CDS**	**SAGE tag sequence; tag position (nt); tag frequency (tags/million); tag rank.**	**Use of canonical polyadenylation site? (Sequence & Position – nt)**	**Affecting coding region?#**	AREs possibly removed by the alternative poly-adenylation event*
Rac GTPase (AY158217)	2,383	211..777	tgtgtgtgta; 951; 366; 1.	No	No	3 out of 4
			acaagttatg; 1959; 351; 0.	No	No	
Receptor tyrosine kinase (AF101194)	6,022	120..4799	tcaatcatta; 821; 249; 1.	No	Yes	4 out of 4
			aagaaatgca; 2013; 132; 0.	No	No	
Myosin light chain (AF071011)	965	30..512	aatcctaatc; 651; 29; 1.	No	No	2 out of 2
			aatatataca; 819; 44; 0.	Yes	No	
Calponin homolog (U86674)	1,963	12..1097	tttatcttca; 1467; 44; 1.	Yes (AATAAA – 1,480)	No	2 out of 4
			cccaaccctc; 1677; 513; 0.	No	No	
Dynein light chain (U55992)	469	6..275	gcattgtata; 163; 29; 1.	No	No	0 out of 0
			aaacccataa; 207; 689; 0.	No	No	
Enolase trans-spliced (U30175)	1,461	40..1344	caacgttggt; 650; 29; 1.	Yes (ATTAAA – 1,024)	Yes	2 out of 2
			tcgttctgat; 1235; 2154; 0.	Yes (AATAAA – 1,353)	No	
Cyclophilin (L46884)	571	16..501	tgtcagggtg; 189; 44; 1.	Yes (AATAAA – 250)	Yes	0 out of 0
			ttgttttcgg; 382; 1465; 0.	No	No	
Actin (U19945)	1,538	45..1175	gccgacgagg; 44; 29; 2.	No	Yes	5 out of 5
			aagtgtgatg; 893; 1216; 1.	No	Yes	5 out of 5
			acatcaacaa; 1307; 3297; 0.	Yes (AATAAA – 1,366)	No	
Phosphofructokinase (L31531)	3,087	147..2492	ttcctttcat; 2895; 29; 2.	No	No	1 out of 3
			ttttccgttt; 2984; 59; 1.	Yes (AATAAA – 3,025)	No	0 out of 3
			taaaaaaaaa; 3043; 103; 0.	No	No	
Triose phosphate isomerase (M83294)	1,080	33..794	atgtcgatgg; 714; 29; 1.	Yes (ATTAAA – 749)	No	4 out of 4
			tcagttactt; 844; 938; 0.	Yes (AATAAA – 1,066)	No	
Superoxide dismutase (M27529)	623	7..561	gataccccag; 319; 29; 2.	No	No	0 out of 0
			tgctacaata; 530; 29; 1.	No	No	0 out of 0
			aaatgatttt; 557; 249; 0.	Yes (AATAAA – 588)	No	
Cu/Zn superoxide dismutase (M97298)	605	23..484	cctattctcc; 513; 498; 1.	No	No	2 out of 2
			cacaaataaa; 580; 161; 0.	Yes (AATAAA – 588)	No	
PUR-alpha-like (AF254148)	1,282	77..880	tgcttaatag; 1110; 191; 1.	No	No	1 out of 3
			ttagatttct; 1242; 15; 0.	No	No	

Transcripts with alternative poly-adenylation events were considered only when confirmed by at least 2 SAGE tags, without downstream internal binding sites for oligo-dT (fake polyA). # – alternative-polyadenylation events affecting the coding region were considered when the alternative upstream tags were located at least 256 nt before the original stop-codon. CDS – stands for the cDNA coordinates of the coding region of the gene. * refers to the number of putative Adenylate Rich Elements (AREs) present in the 3'UTR, that are located downstream of the most 5' SAGE tag found in a transcript, out of the total 3' UTR AREs of the transcript.

## Discussion

Our group has generated and deposited in public databases 163,586 ESTs derived from six developmental stages of *S. mansoni *[5]. A total of 33,180 of these sequences were derived from adult worms. However, due to the normalizing approaches employed for preparing the cDNA libraries used for sequencing – ORESTES [25] and traditional normalized cDNA libraries [26], our sequences offered only a qualitative view of the parasite transcriptome. Sequencing of these cDNA clones provided a glimpse of gene expression from different life-cycle stages of the parasite with a dramatic gene-discovery impact. However, while cDNA sequencing from normalized libraries is a powerful tool for gene discovery, it is not adequate for determining quantitative gene expression patterns. As a complement to the qualitative analysis of the transcriptome of *S. mansoni *we have used SAGE to perform a quantitative evaluation of the adult-worms' transcriptome, one of the most complex life-cycle stages of *S. mansoni*, which expresses at least half of the genes transcribed in this organism [5]. In order to quantify the gene expression in adult worms, we produced a SAGE library and generated 68,238 tags that have been clustered and assigned to genes.

The SAGE technique involves generation and sequencing of large numbers of short tags, defined by the occurrence of a recognition site for a type I restriction enzyme in the mRNA [12]. Ideally, these tags are long enough to be unique to the transcript in question, and the number of copies of a given tag is proportional to the expression level of that transcript in the original mRNA pool. Limitations of the technique include the difficulty of tagging very rare transcripts when a reduced number of tags is generated, the possibility of non-specific tags (tags mapping to distinct transcripts) or transcripts that produce no tags, due to the absence of the restriction site or the poly-A tail [27]. Microarray is the most used approach to evaluate gene expression in large-scale. However, this approach relies on the previous knowledge of gene sequences for the design of the array, and thus, the transcriptome coverage depends on how well defined is the gene set of the target organism. Also, gene quantification using microarrays depend on intensity of hybridization signal, which can be affected by many factors such as location of the probe with respect to the 3'-end of the message, length and G+C content of the probe and signal-to-noise ratios. Depending on the probe spotted, the intensity observed in microarray experiments may reflect the expression of either a single or multiple splicing isoforms for a given gene, making the comparisons with SAGE even more complex. Gene expression data produced by arrays are relative, while SAGE provides an absolute measure of expression. Unlike cDNA microarrays, gene expression analysis using SAGE does not depend on previous sequence knowledge and thus it opens up the possibility of discovering and evaluating the expression of new transcripts. However, the process of constructing and sequencing a SAGE library is laborious and expensive, with a final cost that is 5–10 × higher than microarrays. Another limitation of SAGE is that it limits the analysis of genes that contain restriction sites for the enzyme used to construct the library. In an analysis of 364 full-length *S. mansoni *genes available in public databases, we could not identify restriction sites for *NlaIII *(the enzyme used in our library) in 35 (9.6%) of them. An extrapolation of this would suggest that the frequency of expression of 90% of the *S. mansoni *genes expressed in adults could be evaluated by the SAGE approach employed here.

On the other hand, when 8,669 *S. mansoni *Unigene cluster sequences were evaluated, we observed that 2,193 clusters contained ESTs derived from adult worms. Only 169 of these clusters contained full-length sequences. When tags (rank 0 and rank 1) of these 169 clusters were considered, we observed that 132 (78%) were represented in our SAGE tag list. So, this alternative estimate shows that coverage of our SAGE tags was of about 78% of the genes expressed in adult worms. We also noted that 39 UniGene clusters, with no adult-worm derived ESTs in the cluster composition, had their expression confirmed in this stage by our SAGE data.

### Comparing SAGE and EST data

To establish how the transcriptome derived from SAGE and ESTs can be compared to each other, we evaluated the relative distribution of SAGE and EST sequences over a set of 208 worm full-length mRNA sequences available in GenBank. The 208 full-length transcripts are covered by 26,888 ESTs and 9,589 SAGE tags. As expected, 42% of the SAGE tags that map to the set of 208 full-length genes are positioned in the last 20% of the transcripts. On the other hand, only 17% of the ESTs mapped to these genes cover this same 3' portion of the transcripts (see Additional file 3). This clearly results from the biased distribution of the ESTs that were produced using the ORESTES technique (94,308/110,328 ESTs available at the time of preparation of this manuscript) and shows the necessity of generating further *S. mansoni *ESTs from the 3' end of the transcripts, for a more complete knowledge of the schistosome transcriptome. This also points to a reduced overlap of the SAGE and available EST data, which will result in a poor coverage of low expressed genes by non-normalized 3' UTR ESTs and in the failure of SAGE-to-transcript assignment.

Indeed, from the total of 6,263 tags with frequency higher than one, 2,916 (46.6%) found no matches on the transcript databases used. As expected, this failure in finding the correspondent gene for a specific tag was found to be directly related to the low expression of the corresponding transcript, and its reduced coverage by ESTs. In fact this can be used as an indirect measurement of correlation of SAGE and EST coverage. Whereas 96% of the 50 most frequent tags or 92% of top 100 tags could be identified in a transcript, only 53% of all ditags (6,263 top) or only 40% of all 15,655 tags could be assigned to its correspondent gene. As the *S. mansoni *SAGE tags are usually located at 242 nt upstream from the 3' end of the transcripts (average position of the CATG tags in full length transcripts), this data clearly demonstrates that more 3' sequences from normalized cDNA libraries are required for deciphering the transcriptome of this parasite.

### Putative poly-adenylation in *S. mansoni*

While the same tag can be mapped to many transcripts (indicating a conservation of a nucleotide motif), we also see that a single transcript might sometimes generate various different tags. This parallels to what happens in proteomic studies when the same protein sometimes generates different spots in a gel. The occurrence of multiple tags deriving from the same transcript could occur by methodological problems (such as an incomplete digestion by the anchoring enzyme or the presence of false-polyA tails) or due to biological features such as splicing variants in the transcript region containing the most 3' tag or as the result of the use of multiple poly-adenylation sites. Whereas the use of SAGE tags to evaluate alternative-splicing is more difficult, the occurrence of alternative poly-adenylation events could be evaluated with less assumptions. In order to reduce the impact of methodological aspects over the determination of alternative poly-adenylation events, we have not considered tags sequenced only once, ambiguous tags (those that could be mapped to different transcripts) or internal tags that appeared before long stretches of A's in the transcript, which could have been used as false polyA tails during the cDNA synthesis step [28].

After using the above described filters, consistent events of multiple tags in a single transcript were identified in 13 full length genes. Poly-adenylation events cause a reduction in the transcript size, blocking the transcription of portions of its 3' region, together with the most 3' restriction site of the enzyme used for constructing the SAGE library. The reduction of the 3' UTR observed here, caused by the alternative poly-adenylation was usually accompanied by a removal of a significant portion of the putative ARE transcript repertoire (Adenosine and Uridine-Rich Elements) [29]. AREs are elements that can target host mRNAs towards rapid degradation (by a mechanism dependent on deadenylation), can repress their translation or can increase their stability [reviewed in [30]], dependent on the ligation of ARE binding proteins (ARE-BPs). The putative removal of AREs (observed in 11 out of the 16 putative poly-adenylation events), and the identification of ARE-BPs (such as hnRNPs, CUG-BP and nucleolin) in the transcriptome of *S. mansoni*, suggests that this parasite employs this mechanism for regulating mRNA stability. We should note that the occurrence of partial digestion with *NlaIII *seems to be rare here, as in our list of 15,655 distinct tags, not a single CATG (the restriction site for *NlaIII*) could be found.

### Comparing transcriptome and proteome data

Some reports of proteomic analysis of different developmental stages of *S. mansoni *became recently available. Curwen et al. [31], presented an analysis of the four commonly used schistosome-soluble protein preparations (derived from cercariae, lung-stage, adults and eggs), finding 32 distinct proteins among the most expressed. In adult worms, 26 of the 40 most abundant spots were identified, and corresponded to 22 different proteins. According to Curwen et al. [31], the top 40 most abundant soluble proteins in adult worms, accounted for 27.4% of the total protein content of this stage. In our SAGE analysis, we reached a similar value as the 40 top genes were tagged by 12,364 tags or 21% of the total tags. When the top 10 most abundant adult-worm soluble proteins identified by Curwen et al. [31] are compared to our expression rank based on SAGE, we see that 5/10 proteins are ranked among our top 20 most abundant transcripts (14-3-3 homolog, GST28, FABP, fructose 1,6 bisphosphate aldolase and GAPDH). The remaining proteins vary in our ranking from 21^st ^to 253^th ^(see Additional file 2), suggesting higher stability and/or higher translation rates of these less transcribed genes, when pos-transcriptional events are acting as a second mechanism in the regulation of protein abundance.

RNA analysis by SAGE enabled the evaluation of genes coding for proteins whose physical-chemical properties impaired their analysis by 2D gel electrophoresis. An example is the determination of transcript abundance of priority vaccine candidates of the World Health Organization (such as Sm23 the 793^th ^transcript with 13 tags and paramyosin the 1456^th ^with 7 tags) that could not be evaluated by proteomic analysis [31] due to technical limitations, such as protein size or solubility, imposed by 2D gels.

### Functional classification

The analysis of SAGE tags as to their mapping to genes coding for proteins classified into Gene Ontology functional categories, provides a general view of the parasite functions in terms of their relative frequency. From the data generated it is clear that in the adult stage, the parasite still undergoes intense cellular activity, possibly due to its accelerated membrane turnover as well as metabolic activities possibly involved with immune response evasion and the intense egg-laying activity. Furthermore, the large proportion of proteins potentially involved in defense mechanisms, suggests a dynamic interaction with host and its immune defense system.

### The use of SAGE to interrogate the *S. mansoni *transcriptome

The most abundant tag identified here is 'ACTATTCGGG', a sequence tag that matches diverse isoforms of the gene encoding SmP14, or F10 eggshell protein family. The frequency of this tag strongly suggests that this is the most abundant mRNA species found in adult worms. This abundance is highly significant, especially if we consider the larger biomass of male worms as well as the male bias found in the sex ratio of *S. mansoni *infections [32]. Indeed, among the top 5% most abundant transcripts of adult worms, we can find other eggshell-related genes such as P40 (146^th ^most abundant transcript, with 56 tags), P19 (202^nd ^with 42 tags) and P48 (356^th ^with 26 tags), which advocates their importance in the early-stages of eggshell formation. We should observe that no tags could be identified for egg-secreted proteins (such as ESP3-6 and ESP15), suggesting their expression only in later stages of the eggshell development. The high expression of actin and myosin (heavy and light chains) was also observed, with the identification of their respective genes and gene-paralogs among the top 100 transcripts, reflecting the musculature as one of the major worm tissues. Among the 50 top transcripts, as expected, we observe the high abundance of 12 ribosomal-protein genes as well as genes that encode proteins involved in protein and carbohydrate metabolism. It is also interesting to note the high abundance of the gene that codes for a protein similar to thymosin beta (17^th ^most abundant transcript in adult worms), especially due to its involvement with wound healing [33], its anti-inflammatory properties [34-36] and its possible involvement in the escape from the host immune system in malaria [37].

## Conclusion

One of the most notable strengths of the SAGE method is that results from any new experiments are directly comparable to existing databases. SAGE data represent absolute expression levels, based on the digital enumeration of transcript tags in the total transcriptome. This allows the expression level of any gene to be compared with that of any other gene, from among many libraries of different sources and sizes [38]. In this way, this first report of quantitative expression in adult worms may be used for comparing with future profiles investigating differential expression among diverse developmental stages, during drug exposure, single-sex infections and a series of other relevant biological situations. Together with ESTs, one of the most promising applications of SAGE will be to offer a support for gene identification and genome annotation providing accurate methods for the profiling of genes that are not biased by known sequence information.

## Competing interests

The author(s) declare that they have no competing interests.

## Authors' contributions

EPBO constructed the SAGE library presented here; RDM was responsible for RNA extraction and PSLO coordinated the bioinformatics analysis of SAGE data. EPBO, AP, RDM, SPG, KAA, CFMM, LCCL, SVA and EDN participated on the sequencing of the library and on the analysis and interpretation of the data; EPBO, PSLO, AP, RDM, DNN, SVA and EDN performed bioinformatics analysis. EPBO, PSLO, SVA and EDN conceived the study and participated on its design and coordination. All authors contributed to the writing of this manuscript and approved its final form.

## Supplementary Material

Additional file 3 **Analysis of positional distribution of ESTs and SAGE tags for a set of 208 full-length *S. mansoni *genes**. The positional distribution of all ESTs available in GenBank, as well as all SAGE tags from our study was evaluated over a panel of 208 full length *S. mansoni *genes. Only 17% of the ESTs mappedto 208 full-length transcripts cover the final 20% of the transcripts, while 42% of the generated SAGE tags cover this same region. This shows the reduced overlap of SAGE and ESTs suggests the necessity of generating more *S. mansoni *ESTs, especially from the 3' end of the transcripts, for a better knowledge of the schistosome transcriptome. Click here for file

Additional file 1 **Complete list of *Schistosoma mansoni *SAGE tags**. Contains all 15,655 distinct SAGE tags sequenced, together with their frequency (tag count), the accession numbers of the corresponding genes, their relative position on the mRNA, their tag rank and the annotation of the respective gene. Click here for file

Additional file 4 **Functional classification of the intermediate and less abundant schistosome transcripts based in Gene Ontology analysis**. Functional classification of the most abundant *S. mansoni *transcripts based in Gene Ontology analysis. Click here for file

Additional file 2 **Protein and gene rank**. Comparison of the top 10 proteins ranked by proteome analysis with the expression rank obtained by SAGE. Click here for file
