# Supplementary figures and images for: A quantitative view of the transcriptome of Schistosoma mansoni adult-worms using SAGE
Source: BMC Genomics. 2007 Jun 21;8:186. doi: 10.1186/1471-2164-8-186 (PMC1914358; doi:10.1186/1471-2164-8-186)

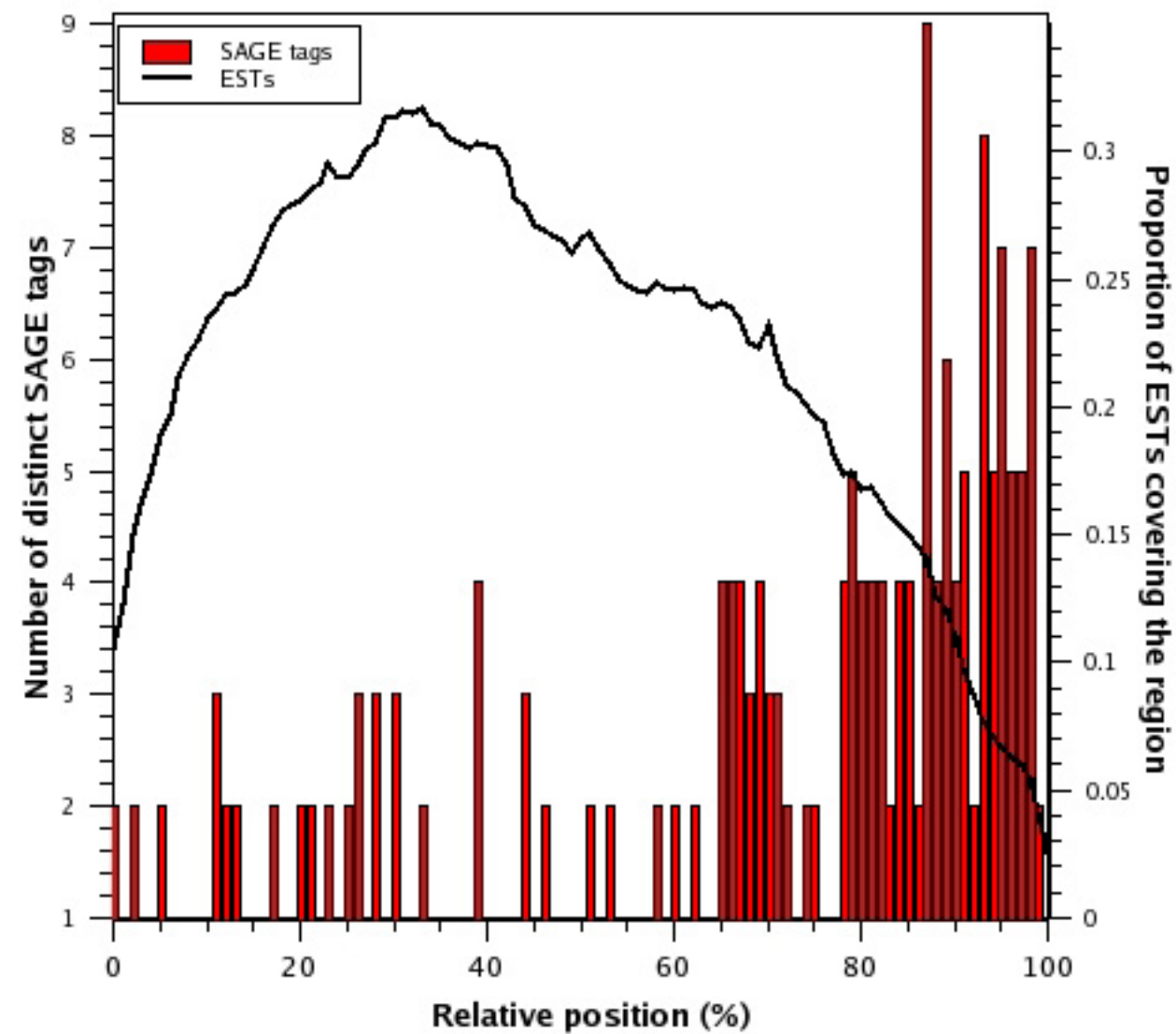

Supplement: Additional file 3 — Analysis of positional distribution of ESTs and SAGE tags for a set of 208 full-length S. mansoni genes. The positional distribution of all ESTs available in GenBank, as well as all SAGE tags from our study was evaluated over a panel of 208 full length S. mansoni genes. Only 17% of the ESTs mappedto 208 full-length transcripts cover the final 20% of the transcripts, while 42% of the generated SAGE tags cover this same region. This shows the reduced overlap of SAGE and ESTs suggests the necessity of generating more S. mansoni ESTs, especially from the 3' end of the transcripts, for a better knowledge of the schistosome transcriptome. [file 1471-2164-8-186-S3.pdf]

## Slide 1
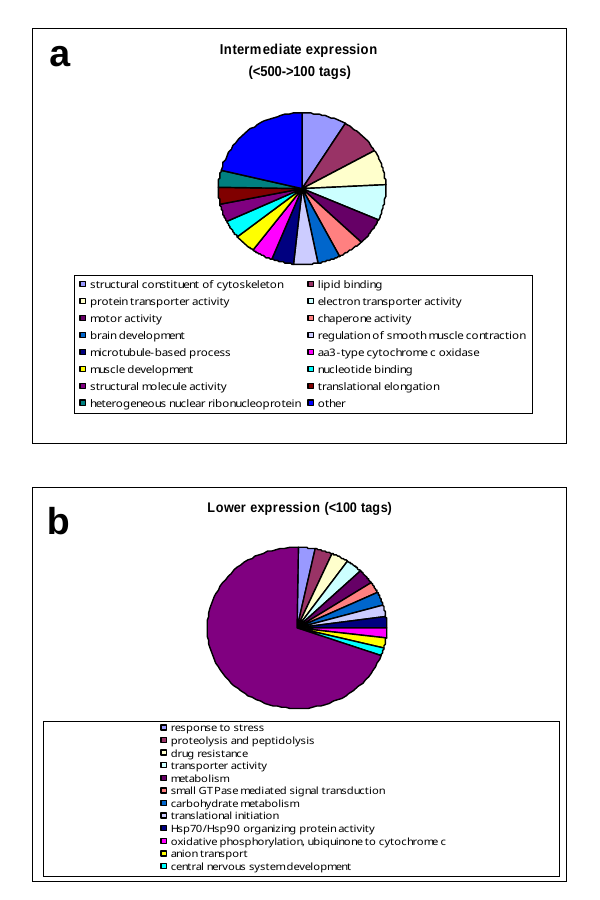

a
b

Supplement: Additional file 4 — Functional classification of the intermediate and less abundant schistosome transcripts based in Gene Ontology analysis. Functional classification of the most abundant S. mansoni transcripts based in Gene Ontology analysis. [file 1471-2164-8-186-S4.ppt]
