# Supplementary material for: A quantitative view of the transcriptome of Schistosoma mansoni adult-worms using SAGE
Source: BMC Genomics. 2007 Jun 21;8:186. doi: 10.1186/1471-2164-8-186 (PMC1914358; doi:10.1186/1471-2164-8-186)
Supplement: Additional file 1 — Complete list of Schistosoma mansoni SAGE tags. Contains all 15,655 distinct SAGE tags sequenced, together with their frequency (tag count), the accession numbers of the corresponding genes, their relative position on the mRNA, their tag rank and the annotation of the respective gene. [file 1471-2164-8-186-S1.pdf]

Tag number	Tag	Tag count	Accession number	Relative position onto mRNA	Tag rank	Annotation
1	actattcggg	1454	M14309lSma.1165	17	0	Female specific polypeptide mRNA
1	actattcggg	1454	gi|160965|gb|J03982.1|SCMEGGA	36	0	S.mansoni eggshell protein (F10) gene, complete cds
1	actattcggg	1454	gi|160942|gb|M21607.1|SCMCHRA	50	0	S.mansoni eggshell (chorion) protein gene, complete cds
2	cctgtaaact	835	C200397.1	99	0	
2	cctgtaaact	835	C211012.1	98	0	
2	cctgtaaact	835	C717678.1	90	0	
3	gaagaagtgg	640	J04017lSma.821	96	0	Heat shock protein 86
4	tcatacaaga	588	U24281lSma.721	95	0	14-3-3 protein (Sm14-3-3)
5	ccggtgtctc	513	TC13660	97	0	weakly similar to GP|24266954|gb|AAN52376.1||AF548321 ribosomal protein L23a {Branchiostoma belcheri}, partial (69%)
6	agcgtccaaa	472	TC7406	85	0	
7	aggcaagtgg	464	AJ311627lSma.863	65	0	Heat shock protein (hsp70 gene)
7	aggcaagtgg	464	gi|161025|gb|L02415.1|SCMHSP70X	67	0	Schistosoma mansoni heat shock protein 70 (HSP70) gene, complete cds
8	cataatgaag	438	TC16678	89	1	SP|P20287|G3P_SCHMA Glyceraldehyde 3-phosphate dehydrogenase(GAPDH) (Major larval surface antigen) (P-37). [Blood fluke] {Schistosoma mansoni}, complete
8	cataatgaag	438	AI976252	67	0	SP|P20287|G3P_SCHMA Glyceraldehyde 3-phosphate dehydrogenase(GAPDH) (Major larval surface antigen) (P-37). [Blood fluke] {Schistosoma mansoni}, partial (39%)
9	cggctcagga	398	AF026805lSma.730	71	0	Fructose bisphosphate aldolase
10	gtagtgcttg	364	AF130787_1459_201	94	0	small subunit ribosomal RNA
11	cttttctaaa	349	TC17835	93	0	similar to GP|8272384|dbj|BAA96444.1||AB021785 metallothionein-like protein {Pyrus pyrifolia}, partial (25%)
12	tgttgttcgt	340	TC17184	96	0	
13	agcaatggaa	319	Z29960lSma.1164	20	2	Tandem repeat
13	agcaatggaa	319	Z29960lSma.1164	20	0	Tandem repeat
14	tgtacgtcat	313	TC7454	94	2	similar to GP|21392234|gb|AAM48471.1||AY118442 SD06874p {Drosophila melanogaster}, partial (78%)
15	tatcgttcta	302	M60895lSma.710	89	0	Sm14 fatty acid-binding protein delta E3 variant mRNA, complete cds; alternatively spliced
16	gcgagtcgaa	293	TC16783	86	0	similar to PIR|JC4662|JC4662 ribosomal protein S3a, cytosolic - human, partial (75%)
17	taatatgcgc	265	TC11200	71	0	similar to GP|10798668|emb|CAC12827.1||AJ250180 beta thymosin {Oncorhynchus mykiss}, partial (51%)
18	gtgctcgaag	255	TC17397	53	0	similar to GP|14581393|gb|AAF98445.1||AY005816 Sj-Ts1 {Schistosoma japonicum}, partial (67%)
19	tgactgatct	254	S71584lSma.780	65	0	Sm28 GST=28 kda glutathione S-transferase [Schistosoma mansoni, Puerto Rican, mRNA, 801 nt]
20	gcacattgtc	252	U91941lSma.154	54	0	Tegumental associated antigen Sm 20.8 (Sm 20.8)
20	gcacattgtc	252	gi|2231616|gb|U87243.1|SMU87243	62	0	Schistosoma mansoni tegumental associated antigen Sm 20.8 (Sm 20.8) mRNA, complete cds
21	acatcaacaa	225	U19945lSma.822	84	0	Actin
22	ccttcggtac	225	TC13745	97	0	similar to GP|15293899|gb|AAK95142.1|AF401570_1|AF401570 ribosomal protein L15 {Ictalurus punctatus}, complete

23	gaggttatgg	215	TC16990	89	0	
24	ttggaggcaa	211	TC13521	93	0	similar to GP|7300349|gb|AAF55508.1||AE003721 CG7187-PA {Drosophila melanogaster}, partial (5%)
24	ttggaggcaa	211	TC13523	82	0	similar to GP|18160467|gb|AAL63818.1||AE009847 phoH like protein {Pyrobaculum aerophilum}, partial (5%)
24	ttggaggcaa	211	CD178269	6	1	
25	gagaacacca	198	M29837|Sma.55	65	0	Tegument antigen (I(H)A)
26	gtaaccaatg	193	TC11060	93	0	similar to GP|2766687|gb|AAC69992.1||AF036955 tetraspanin TE736 {Schistosoma japonicum}, partial (8%)
27	cagcgtcctt	191	TC10489	78	3	weakly similar to SP|P51989|RO21_XENLA Heterogeneous nuclear ribonucleoprotein A2 homolog 1 (hnRNP A2(A)). [African clawed frog] {Xenopus laevis}, partial (60%)
27	cagcgtcctt	191	TC10490	96	0	
28	gggattgccg	190	TC16808	87	0	weakly similar to GP|17298117|dbj|BAB78527.1||AB062685 ribosome-associated protein P40 {Bombyx mori}, partial (60%)
29	gtctgctgat	186	AF422164|Sma.707	87	0	Receptor for activated PKC
30	ccaggttgtg	184	TC9014	0	0	
31	gttatggcca	174	TC10354	75	0	weakly similar to SP|P27484|GRP2_NICSY Glycine-rich protein 2. [Wood tobacco] {Nicotiana sylvestris}, partial (27%)
32	tggattcttg	168	TC14578	81	0	PIR|D40335|HSHU4 histone H4 [validated] - human, complete
33	gtacttagtg	167	L01634|Sma.848	97	0	Myosin heavy chain (MYH)
34	tatgttctct	165	U87629|Sma.614	87	0	Lactate dehydrogenase mRNA, complete sequence
35	attttgtttg	159	AF130788_1003_2262	87	0	[CDS] NADH dehydrogenase 4 (ND4) gene, complete cds
36	gggtatgaat	159	Z32529|Sma.852	87	0	Cathepsin L
37	ggattcggtt	155	TC7332	95	0	weakly similar to SP|P05388|RLA0_HUMAN 60S acidic ribosomal protein P0 (L10E). [Human] {Homo sapiens}, partial (65%)
38	ccatcagcct	154	TC7616	58	0	weakly similar to GP|12963432|gb|AAK11263.1|AF331715_1|AF331715 ribosomal protein P2 {Podospora anserina}, partial (82%)

38	ccatcagcct	154	CD164545	92	0	weakly similar to SP|Q9UUZ6|RLA2_ASPFU 60S acidic ribosomal protein P2 (Allergen Asp f 8). [Sartorya fumigata] {Aspergillus fumigatus}, partial (50%)
39	gccccttgga	154	TC13587	87	0	similar to GP|28200274|gb|AAO31769.1||AY168758 ribosomal protein L10 {Branchiostoma belcheri tsingtaunese}, partial (90%)
40	tcgttctgat	147	U30175|Sma.742	84	0	Enolase trans-spliced
41	tccccgtaca	145				no_annot
42	cccaccactt	144	AY334553|Sma.1043	82	0	Cysteine protease inhibitor (Cys)
43	tacctaggcc	143	TC7615	79	0	similar to GP|28916485|gb|AAO59411.1||AY226981 ATP synthase lipid-binding protein-like protein {Schistosoma japonicum}, complete
44	gtcgcaaagt	139	TC7475	59	1	similar to SP|P25120|RL8_HUMAN 60S ribosomal protein L8. [Rat] {Rattus norvegicus}, partial (98%)
45	ggtttagtag	138	gi|3599492|gb|AF085145.1|AF085145	90	0	Schistosoma mansoni NADH dehydrogenase subunit 5 (NU5M) mRNA, complete cds, mitochondrial gene encoding mitochondrial protein
46	tgcgcgcgtg	137				no_annot
47	cacagacagc	134	TC8025	55	0	similar to GP|6066480|emb|CAB58439.1||AJ250366 40S ribosomal protein S27 {Lumbricus rubellus}, partial (98%)
48	tggtgagggc	130	AF030961|Sma.1154	46	0	Clone NLSL20 40S rRNA protein homolog
49	ctctcgagga	128	TC7478	70	0	similar to GP|24266991|gb|AAN52391.1||AF548336 ribosomal protein S25 {Branchiostoma belcheri}, partial (72%)
50	ggagaagaaa	123	TC7368	3	3	weakly similar to GP|10440990|gb|AAG16892.1|AF173680_1|AF173680 myosin regulatory light chain {Riftia pachyptila}, partial (80%)
51	ccatccgtca	122	U57003|Sma.847	84	0	Breast basic conserved protein (BBC1)
52	gactggccca	120	TC6908	61	3	
53	tctaatgatt	119	TC16865	92	0	
54	ctgtgtctga	118	TC18212	89	0	similar to SP|P02285|H2B_MARGL Histone H2B, sperm. [Spiny starfish] {Marthasterias glacialis}, partial (82%)
55	aagcctgtcg	112	TC17770	74	2	
56	aggaagccca	111	TC17388	72	2	similar to GP|13539680|gb|AAK29203.1|AF225905_1|AF225905 ribosomal protein S15a {Taenia solium}, complete

57	tgttcgtgaa	110	U10431|Sma.680	76	0	Open reading frame
58	gaattaataa	103	TC11030	72	0	similar to PIR|T45729|T45729 dehydrin-like protein - Arabidopsis thaliana, partial (13%)
59	ttgttttcgg	100	L46884|Sma.252	66	0	Cyclophilin
59	ttgttttcgg	100	U30874|Sma.1039	70	0	Cyclophilin B
60	caccctcttt	100	M27512|Sma.782	91	0	Tropomyosin
61	tgatgtggtg	99	TC7130	59	1	homologue to GP|160966|gb|AAA29870.1||J03982 eggshell protein precursor {Schistosoma mansoni}, partial (15%)
61	tgatgtggtg	99	CD128603	53	0	homologue to SP|P13396|EGG3_SCHMA Eggshell protein precursor (Chorion protein). [Blood fluke] {Schistosoma mansoni}, partial (25%)
61	tgatgtggtg	99	CD129000	36	2	homologue to SP|P12796|EGG2_SCHMA Eggshell protein precursor (Chorion protein). [Blood fluke] {Schistosoma mansoni}, partial (47%)
61	tgatgtggtg	99	CD065979	62	0	SP|P13396|EGG3_SCHMA Eggshell protein precursor (Chorion protein). [Blood fluke] {Schistosoma mansoni}, partial (20%)
61	tgatgtggtg	99	CD128615	16	1	GP|10176|emb|CAA29285.1||X05842 eggshell precursor protein {Schistosoma mansoni}, partial (17%)
61	tgatgtggtg	99	CD128642	9	1	homologue to SP|P13396|EGG3_SCHMA Eggshell protein precursor (Chorion protein). [Blood fluke] {Schistosoma mansoni}, partial (18%)
61	tgatgtggtg	99	CD128791	66	0	homologue to SP|P06649|EGG1_SCHMA Eggshell protein precursor (Chorion protein). [Blood fluke] {Schistosoma mansoni}, partial (47%)
61	tgatgtggtg	99	BG932765	82	0	similar to GP|10176|emb|CAA29285.1||X05842 eggshell precursor protein {Schistosoma mansoni}, partial (57%)
61	tgatgtggtg	99	CD063927	69	0	homologue to SP|P06649|EGG1_SCHMA Eggshell protein precursor (Chorion protein). [Blood fluke] {Schistosoma mansoni}, partial (39%)
61	tgatgtggtg	99	CD126385	28	0	SP|P13396|EGG3_SCHMA Eggshell protein precursor (Chorion protein). [Blood fluke] {Schistosoma mansoni}, partial (28%)
61	tgatgtggtg	99	CD126667	67	0	homologue to GP|10176|emb|CAA29285.1||X05842 eggshell precursor protein {Schistosoma mansoni}, partial (40%)

61	tgatgtggtg	99	CD167651	30	0	SP|P13396|EGG3_SCHMA Eggshell protein precursor (Chorion protein). [Blood fluke] {Schistosoma mansoni}, partial (16%)
62	ggcgtactgg	99				no_annot
63	taatgctttg	98	TC13528	89	0	GP|6649234|gb|AAF21436.1|AF195529_1|AF195529 14-3-3 epsilon {Schistosoma mansoni}, complete
64	tgttattgtg	97	TC7358	99	0	similar to SP|O00505|IMA3_HUMAN Importin alpha-3 subunit (Karyopherin alpha-3 subunit) (SRP1-gamma). [Human] {Homo sapiens}, partial (85%)
65	aaccatccag	96	TC16631	71	2	homologue to GP|1619614|emb|CAA69721.1||Y08487 elongation factor 1-alpha {Schistosoma mansoni}, complete
65	aaccatccag	96	TC16638	22	1	weakly similar to PIR|F86214|F86214 protein T6D22.2 [imported] - Arabidopsis thaliana, partial (16%)
65	aaccatccag	96	TC16640	67	0	homologue to GP|1619614|emb|CAA69721.1||Y08487 elongation factor 1-alpha {Schistosoma mansoni}, partial (30%)
65	aaccatccag	96	CD185039	46	0	homologue to GP|1619614|emb|CAA69721.1||Y08487 elongation factor 1-alpha {Schistosoma mansoni}, partial (28%)
66	gtagctcgta	94	TC7643	75	0	weakly similar to GP|15213764|gb|AAK92157.1|AF400185_1|AF400185 ribosomal protein L14 {Spodoptera frugiperda}, partial (36%)
67	tagatgaggg	94	CD080118	53	0	weakly similar to SP|O94111|DYL1_EMENI Dynein light chain, cytoplasmic (8 kDa cytoplasmic dynein light chain). [Aspergillus nidulans] {Emericella nidulans}, partial (56%)
68	cctgagatgt	92	BE431215	55	0	homologue to GP|12248337|gb|AAG13163.2||AF216698 cytochrome c oxidase subunit 3 {Schistosoma mansoni}, partial (21%)
69	agtgttttca	90	AF056330|Sma.773	95	0	AUT1 (AUT1)
70	ttcagctttc	90	TC13565	94	0	GP|5305329|gb|AAD41591.1||AF071011 myosin light chain {Schistosoma mansoni}, complete
71	tttgtttgtt	90	TC14087	90	0	similar to GP|18033709|gb|AAL57218.1|AF361493_1|AF361493 FNP001 {Homo sapiens}, partial (30%)
71	tttgtttgtt	90	TC16880	76	0	similar to GP|24286498|gb|AAN46679.1||AY145443 MAP kinase {Strongylocentrotus purpuratus}, partial (86%)

71	tttgtttgtt	90	TC16992	88	0	weakly similar to GP|15291453|gb|AAK92995.1||AY051571 GH21853p {Drosophila melanogaster}, partial (10%)
71	tttgtttgtt	90	AI977790	46	0	
72	gagcttttgg	90	M86510|Sma.267	71	0	Glutathione peroxidase
73	gttggctgtg	90	TC7532	88	3	similar to GP|23134872|gb|AAG00866.2|AF255664_1|AF255664 major vault protein {Ictalurus punctatus}, partial (54%)
74	gacccaaagt	88	M73624|Sma.61	67	0	Glutathione S-transferase mRNA
75	atcctgttgg	88				no_annot
76	gcgacgacca	87				no_annot
77	gaaggattta	87	TC10437	95	2	PIR|S34275|S34275 protein disulfide-isomerase homolog precursor - fluke (Schistosoma mansoni), complete
77	gaaggattta	87	TC12419	40	1	
78	tgaataagta	87	TC10617	93	0	similar to GP|22004048|dbj|BAC06474.1||AB078979 ubiquitin {Ciona savignyi}, partial (49%)
79	cttatatgcc	86	AF130787_2211-2804	91	0	cytochrome c oxidase subunit 2
79	cttatatgcc	86	AF153976_1_384	86	0	[CDS] Schistosoma mansoni cytochrome c oxidase subunit II (COII) mRNA, partial cds
80	cttattttgt	85	TC10682	94	0	weakly similar to GP|17945700|gb|AAL48899.1||AY071277 RE30690p {Drosophila melanogaster}, partial (83%)
81	gtagattgga	85	TC10803	89	0	weakly similar to GP|7769869|gb|AAF69547.1|AC008007_22|AC008007 F12M16.18 {Arabidopsis thaliana}, partial (9%)
82	acagttttaa	85	TC13692	88	2	weakly similar to GP|15487692|gb|AAL00899.1|AF339837_1|AF339837 retinaldehyde dehydrogenase type 2 {Danio rerio}, partial (71%)
83	gctcggagaa	84				no_annot
84	tcttgcaggt	84	TC17434	35	0	
85	ttaccaccaa	84	TC17090	77	0	weakly similar to GP|15294055|gb|AAK95204.1|AF402830_1|AF402830 40S ribosomal protein S21 {Ictalurus punctatus}, partial (54%)
85	ttaccaccaa	84	TC19195	23	2	weakly similar to PIR|T28793|T28793 diacylglycerol kinase - Caenorhabditis elegans, partial (11%)

86	gacgtgtgat	83	TC7466	91	0	weakly similar to SP|O35777|IAG2_RAT Implantation-associated protein. [Rat] {Rattus norvegicus}, partial (19%)
87	attgtgattt	83	TC13533	97	0	similar to GP|18253047|gb|AAL62468.1||AY072287 ribosomal protein L3 {Spodoptera frugiperda}, partial (91%)
88	aagtgtgatg	83	U19945|Sma.822	58	2	Actin
89	cgcgcacaca	82	TC14031	81	0	
90	cgaagaacat	82	TC10986	83	0	similar to SP|P08708|RS17_HUMAN 40S ribosomal protein S17. [Human] {Homo sapiens}, partial (78%)
91	ctttccctcc	80	TC7368	39	2	weakly similar to GP|10440990|gb|AAG16892.1|AF173680_1|AF173680 myosin regulatory light chain {Riftia pachyptila}, partial (80%)
92	actaggaaca	79	AI976259	54	1	homologue to GP|12248331|gb|AAG13157.2||AF216698 cytochrome c oxidase subunit 1 {Schistosoma mansoni}, partial (19%)
93	tcgcctagtg	79				no_annot
94	ttgccaatga	78				no_annot
95	gttacaaaga	76	AF130788_2465_2827	43	0	[CDS] NADH dehydrogenase 3 (ND3) gene, complete cds
96	ctgcagtaat	76	TC8215	68	0	weakly similar to GP|23172649|gb|AAN14210.1||AE003772 CG7939-PC {Drosophila melanogaster}, partial (92%)
97	aactcggttg	76	TC7442	85	2	similar to GP|13183614|gb|AAK15271.1|AF308144_1|AF308144 Sj-Ts4 protein {Schistosoma japonicum}, complete
97	aactcggttg	76	TC7443	83	0	similar to GP|13183614|gb|AAK15271.1|AF308144_1|AF308144 Sj-Ts4 protein {Schistosoma japonicum}, partial (70%)
97	aactcggttg	76	TC15212	89	0	
98	ctgtaaaatt	75	TC7871	83	2	similar to GP|7295584|gb|AAF50894.1||AE003571 CG15455-PA {Drosophila melanogaster}, partial (5%)
99	cagtgaagcg	75	TC13608	70	2	similar to GP|19571835|emb|CAD27733.1||AJ438987 S6 ribosomal protein {Paracentrotus lividus}, partial (90%)
100	caaacaccaa	74	TC7719	95	0	weakly similar to GP|16769502|gb|AAL28970.1||AY061422 LD35289p {Drosophila melanogaster}, partial (18%)

101	ttgtatcctt	74	TC7464	90	0	similar to GP|15213788|gb|AAK92169.1|AF400197_1|AF400197 ribosomal protein L35A {Spodoptera frugiperda}, partial (65%)
102	tacatcctgt	73	TC6866	85	0	GP|1314714|gb|AAA99796.1||U54584 histone H1 {Schistosoma mansoni}, complete
103	ccagagtcct	73	TC10432	64	0	similar to GP|7294919|gb|AAF50249.1||AE003551 CG3395-PA {Drosophila melanogaster}, partial (93%)
103	ccagagtcct	73	TC10435	92	0	similar to GP|7294919|gb|AAF50249.1||AE003551 CG3395-PA {Drosophila melanogaster}, partial (87%)
104	tcatcgttcc	72	TC14118	60	0	weakly similar to GP|7648816|gb|AAF65682.1|AF216206_1|AF216206 ribosomal protein S19 {Drosophila melanogaster}, partial (54%)
105	gcgaagtttg	71	AF157561|Sma.673	78	0	Thioredoxin peroxidase (TPx-2)
106	ctaaaagggt	71	TC10947	46	0	PIR|A27501|HSHU33 histone H3.3 [validated] - human, complete
107	gttggaggaa	71	TC13604	88	0	similar to SP|P00829|ATPB_BOVIN ATP synthase beta chain, mitochondrial precursor. [Bovine] {Bos taurus}, partial (91%)
108	cacttgcagc	70	TC10534	81	0	weakly similar to SP|P49165|RL4_URECA 60S ribosomal protein L4 (L1). [Innkeeper worm, Spoonworm] {Urechis caupo}, partial (59%)
109	aacatcatct	70	TC13748	92	0	SP|Q95WA2|TCTP_SCHMA Translationally controlled tumor protein homolog (TCTP) (Histamine- releasing factor) (Fragment). [Blood fluke] {Schistosoma mansoni}, complete
109	aacatcatct	70	CD191913	25	1	similar to GP|22759432|gb|AAN06550.1||AE003845 CG1651-PA {Drosophila melanogaster}, partial (3%)
110	gttgcgacaa	69	AF153977|Sma.635	53	0	Eukaryotic translation initiation factor 5 (eIF-5)
111	tccagatcta	68	TC7420	90	0	similar to GP|21309890|gb|AAM46087.1|AF370036_1|AF370036 Cs1 protein {Schistosoma japonicum}, partial (97%)
112	aagagcacgt	68				no_annot
113	gctgtgattc	67	TC8202	91	0	similar to GP|11878097|gb|AAG40801.1||AF286702 Mf2 protein {Schistosoma japonicum}, complete
114	ggtaagatac	67	TC7856	92	0	weakly similar to GP|3413789|emb|CAA09006.1||AJ010116 NADH-cytochrome b5 reductase {Homo sapiens}, partial (34%)

114	ggtaagatac	67	N20779	87	0	weakly similar to GP|10727977|gb|AAG22320.1||AE003543 CG5946-PB {Drosophila melanogaster}, partial (9%)
115	tcaataccaa	67	TC16871	54	2	weakly similar to GP|5531805|gb|AAD44477.1||AF078845 16.7Kd protein {Homo sapiens}, partial (30%)
116	cttcgaacca	66	M80214|Sma.820	61	0	Alpha-tubulin (sat-1)
117	cagctgataa	65	CD080887	80	0	
118	gacgttctgt	65	TC7339	87	1	similar to GP|6942068|gb|AAF32311.1|AF217788_1|AF217788 O-glycosyltransferase {Drosophila melanogaster}, partial (81%)
119	taactggtaa	64	C611930.1	64	3	
120	attgcataaa	64	TC10700	77	0	similar to GP|3876913|emb|CAB07387.1||Z92834 C. elegans RPS-26 protein (corresponding sequence F39B2.6) {Caenorhabditis elegans}, partial (79%)
121	atagcgatgt	64	TC14185	84	2	similar to GP|14646761|dbj|BAB61954.1||AB060654 23-kDa proteolipid {Mus musculus}, partial (63%)
122	tcagttactt	64	M83294|Sma.264	78	0	Triose phosphate isomerase
123	tgagcgcgtt	64	TC7503	88	0	homologue to PIR|H72173|H72173 D5L protein - variola minor virus (strain Garcia-1966), partial (18%)
124	tgaagcccag	63	TC7748	70	0	weakly similar to GP|22758890|gb|AAN05604.1||AF526223 ribosomal protein L21 {Argopecten irradians}, partial (89%)
125	ttttgccgaa	63	TC17186	33	2	SP|O01374|UCR6_SCHMA Ubiquinol-cytochrome C reductase complex 14 kDa protein. [Blood fluke] {Schistosoma mansoni}, complete
126	gggctaattt	63	TC11419	94	0	
127	attaacctaa	62	TC10635	86	0	
128	tcaacatttc	62	TC7046	97	0	weakly similar to SP|O96614|SER1_GALME Sericin-1 (Silk gum protein 1) (Fragment). [Wax moth] {Galleria mellonella}, partial (27%)
129	caagtggtaa	62				no_annot
130	gacatagttc	62	TC13787	88	0	weakly similar to SP|P21796|POR1_HUMAN Voltage-dependent anion-selective channel protein 1 (VDAC-1) (hVDAC1) (Outer mitochondrial membrane protein porin 1) (Plasmalemmal porin) (Porin 31HL) (Porin 31HM). [Human] {Homo sapiens}, partial (10%)

131	gtcgcggcgg	61	TC16556	66	1	weakly similar to PIR|T39903|T39903 serine-rich protein - fission yeast (Schizosaccharomyces pombe), partial (10%)
132	cttatcgacg	61				no_annot
133	atatgatggt	60	M97555|Sma.823	87	0	Tropomyosin
134	aaggaggtcg	60	TC7336	68	1	homologue to SP|P05217|TBB2_HUMAN Tubulin beta-2 chain. [Mouse] {Mus musculus}, complete
135	ttgattattt	59	AF130787_1_229	78	0	cytochrome c oxidase subunit 1
135	ttgattattt	59	gi|4138864|gb|AF101196.1|AF101196	95	0	Schistosoma mansoni cytochrome c oxidase subunit 1 (cox1) mRNA, mitochondrial gene encoding mitochondrial protein, complete cds
136	taatatgcct	59	TC15168	34	1	
136	taatatgcct	59	CD079419	1	1	
137	agttgagtta	59	AF130787_337-1393	88	0	large subunit ribosomal RNA
138	tggttgaata	59	TC16849	94	0	similar to PIR|S34109|S34109 ribosomal protein S13, cytosolic [validated] - human, complete
139	gttacgattc	59				no_annot
140	aattgatcat	58	TC13500	98	0	GP|3892187|gb|AAC78303.1||AF064592 RNA-binding protein {Schistosoma japonicum}, complete
140	aattgatcat	58	TC13509	47	3	GP|3892187|gb|AAC78303.1||AF064592 RNA-binding protein {Schistosoma japonicum}, complete
141	aatatttgtt	58				no_annot
142	atcaatctgc	57	TC7368	58	0	weakly similar to GP|10440990|gb|AAG16892.1|AF173680_1|AF173680 myosin regulatory light chain {Riftia pachyptila}, partial (80%)
143	tatggatgcc	57	TC6902	62	0	weakly similar to GP|23491735|dbj|BAC16802.1||AB082926 ribosomal protein L10a {Homo sapiens}, partial (58%)
143	tatggatgcc	57	TC7157	33	0	similar to SP|P53025|R10A_HUMAN 60S ribosomal protein L10a (CSA-19). [Rat] {Rattus norvegicus}, partial (88%)
144	gtagtgggag	57	TC14194	93	0	
145	taaagtgact	56	TC7042	62	0	similar to SP|P49154|RS2_URECA 40S ribosomal protein S2. [Innkeeper worm, Spoonworm] {Urechis caupo}, partial (79%)

145	taaagtgact	56	TC7096	61	0	similar to SP|P49154|RS2_URECA 40S ribosomal protein S2. [Innkeeper worm, Spoonworm] {Urechis caupo}, partial (79%)
145	taaagtgact	56	CD125483	72	2	
146	cgattactca	56	M15509|Sma.832	90	0	Major egg antigen (p40)
147	acgaaggcga	56	TC16732	50	0	weakly similar to GP|15028450|gb|AAK81721.1|AF395203_1|AF395203 DnaJ-like protein {Cercopithecus aethiops}, partial (77%)
147	acgaaggcga	56	CD112288	27	0	weakly similar to SP|P31689|DJA1_HUMAN DnaJ homolog subfamily A member 1 (Heat shock 40 kDa protein 4) (DnaJ protein homolog 2) (HSJ-2) (HSDJ). [Human] {Homo sapiens}, partial (25%)
148	gaggagaggt	56	TC10840	93	0	similar to SP|P02834|HMUX_DROME Homeotic Ultrabithorax protein. [Fruit fly] {Drosophila simulans}, partial (5%)
149	gatttctcta	55	TC10537	87	0	similar to GP|16566725|gb|AAL26580.1|AF429978_1|AF429978 ribosomal protein S4 {Spodoptera frugiperda}, partial (97%)
150	tggtcctcct	54	CD144909	76	0	similar to SP|Q9BWJ5|S3BA_HUMAN Splicing factor 3B subunit 10 (SF3b10) (Pre-mRNA splicing factor SF3b 10 kDa subunit). [Human] {Homo sapiens}, partial (98%)
151	tcagtactgg	54	L36833|Sma.1189	79	0	(clone C5-1B3) phosphoglycerate kinase (PGK) gene
152	gttatgtaat	54				no_annot
153	atggaggaac	54	TC11203	71	0	weakly similar to GP|10726779|gb|AAF56564.2||AE003754 CG6090-PA {Drosophila melanogaster}, partial (63%)
154	catagcaaaa	53				no_annot
155	cagctgtgaa	53	CD091954	87	0	
156	gctggggaat	53				no_annot
157	gggttctggt	53	TC7675	73	0	similar to PIR|S00081|KHCHL cathepsin L- chicken, partial (88%)
158	cctactatag	52	TC18151	96	0	weakly similar to SP|Q9CQ69|UCRQ_MOUSE Ubiquinol-cytochrome C reductase complex ubiquinone-binding protein QP-C(Ubiquinol-cytochrome C reductase complex 9.5 kDa protein) (Complex III subunit VII). [Mouse] {Mus musculus}, partial (85%)
159	tttgcttgtg	52	TC16561	87	0	

160	tgtcggattt	52	TC7271	66	0	weakly similar to GP|10799003|gb|AAG23159.1||AF257083 polyprotein {Heterodera glycines}, partial (30%)
161	ccgaagcgtg	52	TC10912	4	0	similar to PIR|JC1253|JC1253 ribosomal protein L17A - fruit fly (Drosophila melanogaster), partial (98%)
162	actcagtgtt	52	TC10515	87	0	
162	actcagtgtt	52	TC10516	97	0	
163	gatttgacgt	51	TC10884	85	0	weakly similar to SP|O95182|N4AM_HUMAN NADH-ubiquinone oxidoreductase subunit B14.5a(Complex I-B14.5a) (CI-B14.5a). [Human] {Homo sapiens}, partial (28%)
164	aggacgaagt	51	TC16788	91	0	similar to GP|6517204|dbj|BAA87880.1||AB035354 Drab11 {Drosophila melanogaster}, partial (77%)
164	aggacgaagt	51	TC16789	78	0	
164	aggacgaagt	51	TC16791	88	0	
165	tatcggcgat	51	TC7474	75	3	similar to SP|P10113|RAPA_HUMAN Ras-related protein RAP-1A (C21KG) (KREV-1 protein) (GTP-binding protein SMG-P21A) (G-22K). [Bovine] {Bos taurus}, partial (95%)
166	atggacgacc	51	AY266330|Sma.897	87	0	Tyrosinase 1 precursor (TYR1)
167	gtgttggttt	51				no_annot
168	ttgtgctgtt	50	TC17433	48	0	weakly similar to SP|P23821|RS7_HUMAN 40S ribosomal protein S7 (S8). [Rat] {Rattus norvegicus}, partial (69%)
169	agcggacgag	50	TC17676	11	0	homologue to PIR|S01622|S01622 histone H2A, embryonic (clone L3) - sea urchin (Strongylocentrotus purpuratus), partial (79%)
170	tcggtgaaca	49	TC6865	61	0	
171	aaacaaaaca	49	TC8189	67	0	similar to SP|Q39580|DYL1_CHLRE Dynein 8 kDa light chain, flagellar outer arm. {Chlamydomonas reinhardtii}, partial (97%)
171	aaacaaaaca	49	TC10527	46	0	homologue to GP|15545995|gb|AAK38749.1||AY029255 dynein light chain 2 {Mus musculus}, complete
171	aaacaaaaca	49	TC10529	37	0	similar to SP|O02414|DYL1_ANTCR Dynein light chain LC6, flagellar outer arm. [Sea urchin] {Anthocidaris crassispina}, complete
171	aaacaaaaca	49	TC10531	47	0	similar to GP|15545995|gb|AAK38749.1||AY029255 dynein light chain 2 {Mus musculus}, partial (96%)

172	tggtttggca	49	TC7474	97	0	similar to SP|P10113|RAPA_HUMAN Ras-related protein RAP-1A (C21KG) (KREV-1 protein) (GTP-binding protein SMG-P21A) (G-22K). [Bovine] {Bos taurus}, partial (95%)
173	tgaacgcgtt	48				no_annot
174	attaatttga	48	TC16695	83	2	GP|1314722|gb|AAA99800.1||U54588 fibrillin 2 {Schistosoma mansoni}, partial (90%)
175	ggaggtactg	48	TC10702	55	0	similar to PIR|S49326|S49326 nascent polypeptide-associated complex alpha chain - human, partial (35%)
176	gcagctcaac	48	TC17457	58	0	similar to GP|4883773|gb|AAD31646.1||AF120212 ubiquitin-conjugating enzyme {Gallus gallus}, partial (98%)
177	gggtggacgg	48				no_annot
178	tataagacgc	47	TC18246	88	0	
179	aaatacggac	47	M21309|Sma.712	68	0	Cathepsin B (Sm31)
180	aaacccataa	47	U55992|Sma.893	44	0	Dynein light chain (SmDLC)
181	tccaaaagga	47	TC7701	85	0	similar to GP|1125808|gb|AAA83599.1||U42845 Ribosomal protein, large subunit protein 11.2 {Caenorhabditis elegans}, partial (85%)
181	tccaaaagga	47	TC13216	40	1	
181	tccaaaagga	47	CD087323	19	1	weakly similar to GP|23598392|gb|AAN35167.1||AY149445 60S ribosomal protein L11 {Euprymna scolopes}, partial (63%)
182	gacaaggctg	47	C610224.1	7	0	
183	ctgctggact	47	C200303.1	81	0	
184	tgggtgcgga	46	TC14667	82	0	similar to GP|19528139|gb|AAL90184.1||AY089446 AT26381p {Drosophila melanogaster}, partial (75%)
185	taatgtagac	46				no_annot
186	gggcgaccat	45	TC13968	63	0	weakly similar to GP|22022313|dbj|BAC06513.1||AB079121 transformer-2a {Oryzias latipes}, partial (41%)
187	gatctcttgc	45	TC7537	30	1	similar to GP|7300667|gb|AAF55815.1||AE003732 CG15697-PA {Drosophila melanogaster}, partial (44%)
187	gatctcttgc	45	CD148092	85	0	homologue to GP|7300667|gb|AAF55815.1||AE003732 CG15697-PA {Drosophila melanogaster}, partial (16%)

187	gatctcttgc	45	L47056	77	0	GP|7300667|gb|AAF55815.1||AE003732 CG15697-PA {Drosophila melanogaster}, partial (10%)
188	gcagaccaac	45	TC7816	7	0	similar to SP|P42558|RAN_CHICK GTP-binding nuclear protein RAN (TC4). [Chicken] {Gallus gallus}, partial (94%)
189	ccaagaagtt	45	TC6987	54	1	similar to GP|22655520|gb|AAN04092.1|AF527457_1|AF527457 ribosomal protein S24 {Clonorchis sinensis}, partial (68%)
189	ccaagaagtt	45	TC6987	54	0	similar to GP|22655520|gb|AAN04092.1|AF527457_1|AF527457 ribosomal protein S24 {Clonorchis sinensis}, partial (68%)
189	ccaagaagtt	45	TC7098	38	0	similar to SP|O42387|RS24_FUGRU 40S ribosomal protein S24. [Japanese pufferfish, Takifugu rubripes] {Fugu rubripes}, partial (79%)
190	tcactaacct	44	C607907.1	96	0	
191	tcgactagct	44				no_annot
192	cttgatgtct	44	TC7940	90	0	similar to GP|3641346|gb|AAC36355.1||AF091341 apoferritin-2 {Schistosoma japonicum}, partial (98%)
193	gggtggtgag	44	AW330555	63	0	similar to GP|17945471|gb|AAL48789.1||AY071167 RE21371p {Drosophila melanogaster}, partial (10%)
194	aggaacatta	44				no_annot
195	tattaaccga	43	C210430.1	92	0	
195	tattaaccga	43	C601118.1	92	0	
196	ggttcagatg	43				no_annot
197	tcgtaataaa	43	TC13586	79	0	GP|22531389|emb|CAD44625.1||AJ506158 cathepsin B1 isotype 2 {Schistosoma mansoni}, complete
197	tcgtaataaa	43	TC17646	66	0	homologue to PIR|A26480|A26480 knob protein - malaria parasite (Plasmodium falciparum) (fragments), partial (5%)
198	ctccttggtg	43	TC7364	73	0	similar to GP|3892185|gb|AAC78302.1||AF064591 protein disulfide isomerase {Schistosoma japonicum}, complete
199	tagcttgtcc	43	TC10542	79	0	homologue to GP|21686538|gb|AAM74948.1|AF519808_1|AF519808 MA {Schistosoma japonicum}, partial (20%)

199	tagcttgtcc	43	TC10543	95	0	homologue to GP|21686538|gb|AAM74948.1|AF519808_1|AF519808 MA {Schistosoma japonicum}, partial (83%)
200	ataattcagt	43	gi|160936|gb|M67499.1|SCMCANPA	98	0	S.mansoni calcium-activated neutral proteinase (CANP) mRNA, complete cds
201	gataatatca	42	TC8250	93	0	
202	gtaaaataca	42	Z27402|Sma.237	30	0	Eggshell protein
203	atcccgaatt	42	M93097|Sma.726	69	0	Antigen homolog of human Ro/ss-A autoantigen mRNA, 3 end
204	cataatggtg	42	C202098.1	21	4	
205	tttgtacctc	41	TC8122	80	0	
206	ctgtaaactg	41				no_annot
207	gaagaccgat	41	TC7459	84	0	similar to GP|21068659|emb|CAD21558.1||AJ428513 HEL protein {Chironomus tentans}, partial (96%)
208	cagaatggca	40	TC17015	63	2	
209	gtttacgtta	40				no_annot
210	gtccgtattg	40	TC11350	85	0	weakly similar to GP|21064815|gb|AAM29637.1||AY113632 RH72196p {Drosophila melanogaster}, partial (67%)
211	ctaatccaag	40	TC13727	40	1	
212	tattgtaact	40	TC18160	49	0	
213	gttaccgaat	39	CD084238	91	0	
214	agcactgacc	39				no_annot
215	tgcgtgtgtg	39				no_annot
216	tattgaatac	39	X77211|Sma.27	98	0	IMP25 mRNA
217	ttgtgaaagc	38				no_annot
218	gatgcagaga	38	TC17293	30	0	weakly similar to SP|Q94514|COXA_DROME Cytochrome c oxidase polypeptide Va, mitochondrial precursor. [Fruit fly] {Drosophila melanogaster}, partial (24%)
219	gcaaacaaat	38				no_annot
220	ggctcaggaa	38				no_annot
221	aatctgtagt	38	AF121199|Sma.70	92	0	Thioredoxin peroxidase (TPx-1)
222	ttaacttaac	38				no_annot

223	gacttaattg	37	TC10540	87	0	weakly similar to GP|23093943|gb|AAN12018.1||AE003556 CG7188-PB {Drosophila melanogaster}, partial (18%)
223	gacttaattg	37	AI976823	61	0	
224	agattcgaga	37	TC16722	86	1	similar to PIR|A48133|A48133 pre-mRNA splicing SRp75 - human, partial (33%)
225	cacacaatga	37	TC19306	29	1	
226	atcaagaaat	37	AF473536|Sma.90	75	0	Thioredoxin
227	tgatgttggg	37				no_annot
228	tgattgaaaa	36	TC14054	73	0	
229	caaattgctg	36				no_annot
230	tgttggcttt	36	TC10473	84	1	weakly similar to SP|Q01105|SET_HUMAN SET protein (HLA-DR associated protein II) (PHAPII) (Phosphatase 2A inhibitor I2PP2A). [Human] {Homo sapiens}, partial (68%)
231	gtcaattgtg	36				no_annot
232	attacgatat	36	TC10977	87	0	
233	aataaactac	36	TC11413	93	0	similar to GP|28916483|gb|AAO59410.1||AY226980 nucleoside diphosphate kinase {Schistosoma japonicum}, partial (95%)
234	gcagcgttcc	36	TC14155	84	1	weakly similar to GP|19344076|gb|AAH25628.1||BC025628 expressed sequence AA420417 {Mus musculus}, partial (23%)
235	cagcaattcg	36	TC10506	55	4	weakly similar to PIR|JC1308|JC1308 ribosomal protein L5 - chicken, partial (95%)
236	tgattcggtt	36	TC15634	94	0	
237	ttagggctta	36	TC10568	59	2	similar to GP|17862622|gb|AAL39788.1||AY069643 LD40966p {Drosophila melanogaster}, partial (12%)
238	cttcactgtg	36	TC11008	84	0	similar to PIR|F85176|F85176 isomerase like protein [imported] - Arabidopsis thaliana, partial (22%)
238	cttcactgtg	36	CD164081	31	3	
239	gtaagttgta	36	TC10965	72	0	similar to GP|21392208|gb|AAM48458.1||AY118429 RH26504p {Drosophila melanogaster}, partial (36%)
240	ttaccctttc	36				no_annot
241	tatcaaccca	36	C200369.1	6	2	
242	attgttacaa	35	N21926	58	0	

243	tgtgtgtgtg	35	TC6877	39	1	
243	tgtgtgtgtg	35	TC13323	12	1	
243	tgtgtgtgtg	35	TC14319	2	5	
243	tgtgtgtgtg	35	TC14827	85	0	
243	tgtgtgtgtg	35	TC16575	78	0	GP|161038|gb|AAA29903.1||M15509 major egg antigen {Schistosoma mansoni}, partial (21%)
243	tgtgtgtgtg	35	TC16594	70	4	SP|P12812|P40_SCHMA Major egg antigen (P40). [Blood fluke] {Schistosoma mansoni}, complete
243	tgtgtgtgtg	35	CD077019	46	2	
243	tgtgtgtgtg	35	CD090767	54	2	homologue to GP|14591792|gb| ORF37 ODV-E66 {Cydia pomonella granulovirus}, partial (1%)
243	tgtgtgtgtg	35	CD182269	82	0	
243	tgtgtgtgtg	35	CD079195	16	2	
244	cgcctcttgc	35				no_annot
245	tggagggaca	35	TC7341	52	1	SP|P72983|Y20L_SYNY3 Ycf20-like protein. [strain PCC 6803] {Synechocystis sp.}, partial (10%)
246	gtggttgagg	35				no_annot
247	agattgtaaa	35	TC16831	98	0	weakly similar to GP|7161181|emb|CAB76563.1||AJ276003 GAR1 protein {Homo sapiens}, partial (15%)
247	agattgtaaa	35	TC17287	19	0	weakly similar to GP|14860969|gb|AAK72378.1||AY038364 60S ribosomal protein L7/L12 precursor {Spodoptera frugiperda}, partial (40%)
248	tataactgca	35				no_annot
249	cctcctgctc	35	TC10360	92	0	weakly similar to GP|17158056|gb|AAA17976.2|AAA17976|U05040 FUSE binding protein {Homo sapiens}, partial (20%)
250	ttgtaaagaa	35				no_annot
251	ccccctaagt	35	TC7477	95	0	
252	tgaatctgta	35	TC18597	5	1	
253	gactcacagt	35	M31106|Sma.60	68	0	Clone G2.[1,6] glutathione S-transferase
254	cccaaccctc	35	U86674|Sma.752	85	0	Calponin homolog
255	agcaatgaaa	35	CD116037	36	0	
256	cctattctcc	34	M86867|Sma.743	85	0	Mansoni sytosolic Cu/Zn superoxide dismutase (SOD)

256	cctattctcc	34	gi|161118|gb|M97298.1|SCMSODCT	84	1	Schistosome mansoni sytosolic Cu/Zn superoxide dismutase (SOD) mRNA, complete cds
257	gttacatatg	34	CD083251	36	0	
258	atgcatatcc	34	C207624.1	13	4	
258	atgcatatcc	34	C606657.1	12	5	
259	atcgaagctg	34	AF310263|Sma.695	82	0	Heat shock protein HSP60
260	agactacata	34	M80334|Sma.699	74	0	Actin
261	gaggaatagt	34	TC16819	42	0	
261	gaggaatagt	34	TC16821	27	5	
261	gaggaatagt	34	TC16822	7	6	
262	taaatacctg	34	TC10733	94	2	
263	gcttggagag	34				no_annot
264	acgttattgg	34	TC16813	82	0	GP|4160167|emb|CAA10601.1||AJ132193 calmodulin {Caenorhabditis elegans}, complete
265	cgtgctggaa	34				no_annot
266	tatgatatta	33				no_annot
267	gatttcgaaa	33	TC7449	85	0	weakly similar to GP|2541910|dbj|BAA22850.1||AB008003 troponin T {Mizuhopecten yessoensis}, partial (43%)
268	atcgaaaaag	33	TC10765	77	2	similar to GP|7635738|emb|CAB88388.1||AJ252184 L12 ribosomal protein {Hydra vulgaris}, complete
269	tgaagtatgt	33	CD088945	59	0	
270	ggcagaggta	33				no_annot
271	ttattttagg	33	TC16772	74	0	homologue to GP|12248337|gb|AAG13163.2||AF216698 cytochrome c oxidase subunit 3 {Schistosoma mansoni}, complete
271	ttattttagg	33	H98293	55	0	similar to GP|12248337|gb|AAG13163.2||AF216698 cytochrome c oxidase subunit 3 {Schistosoma mansoni}, partial (42%)
272	gtaattcctt	33	C611310.1	94	0	
273	aataactaag	33	TC17271	92	0	similar to GP|15126600|gb|AAH12236.1||BC012236 Cd151 protein {Mus musculus}, partial (7%)
274	gatactgtat	32	TC8227	27	1	
275	ggatgagttg	32				no_annot

276	gatgagtatg	32	TC9065	92	0	
277	ttgattcgtg	32	TC7786	86	0	
277	ttgattcgtg	32	CD125738	44	0	
278	acattgcgca	32				no_annot
279	tttgtttccg	32	TC7439	68	1	
280	taatcggtgt	32	TC7697	66	0	weakly similar to GP|29344961|gb|AAO82718.1||AE016956 universal stress protein family {Enterococcus faecalis V583}, partial (19%)
281	gccaccccct	32				no_annot
282	attcattgag	31	AF130787_337-1393	5	1	large subunit ribosomal RNA
283	agtggtcgag	31	TC16844	56	0	similar to GP|6746611|gb|AAF27650.1|AF218064_1|AF218064 malate dehydrogenase precursor {Nucella lapillus}, partial (88%)
283	agtggtcgag	31	TC16845	46	1	weakly similar to PIR|S01350|DEMSMM malate dehydrogenaseprecursor, mitochondrial - mouse, partial (20%)
284	ttaatcctcc	31	TC7515	86	0	
285	cctcagaaac	31				no_annot
286	gtcttgatgc	31	U83906|Sma.649	88	0	Putative cytosol aminopeptidase
287	ttgtggtagt	31	TC11891	72	0	weakly similar to GP|2231617|gb|AAB62067.1||U87243 Sm 20.8 {Schistosoma mansoni}, partial (29%)
287	ttgtggtagt	31	TC16628	43	1	
288	cgcttatgta	31	TC14034	96	0	weakly similar to GP|16974785|gb|AAL32469.1|AF441233_1|AF441233 PDZ-domain protein scribble {Mus musculus}, partial (9%)
289	tgcgtaatga	31	AI018926	39	1	homologue to GP|160996|gb|AAA16243.1||L09549 glyceraldehyde-3-phosphate dehydrogenase {Schistosoma japonicum}, partial (9%)
289	tgcgtaatga	31	CD065377	25	2	homologue to SP|P20287|G3P_SCHMA Glyceraldehyde 3-phosphate dehydrogenase(GAPDH) (Major larval surface antigen) (P-37). [Blood fluke] {Schistosoma mansoni}, partial (20%)

289	tgcgtaatga	31	AI977709	31	2	SP|P20287|G3P_SCHMA Glyceraldehyde 3-phosphate dehydrogenase(GAPDH) (Major larval surface antigen) (P-37). [Blood fluke] {Schistosoma mansoni}, partial (37%)
290	gaaaatatat	31	TC10557	98	0	weakly similar to SP|P32067|LA_MOUSE Lupus La protein homolog (La ribonucleoprotein) (La autoantigen homolog). [Mouse] {Mus musculus}, partial (12%)
291	caacacaccg	31	TC10630	79	0	similar to GP|1405323|dbj|BAA10932.1||D64055 LMPX of lamprey {Petromyzon marinus}, partial (74%)
291	caacacaccg	31	TC10631	79	0	similar to GP|1405323|dbj|BAA10932.1||D64055 LMPX of lamprey {Petromyzon marinus}, partial (75%)
292	tccctattaa	31				no_annot
293	tacgtacgga	31				no_annot
294	acgtacaact	30	TC16934	93	0	homologue to SP|P35998|PRS7_HUMAN 26S protease regulatory subunit 7 (MSS1 protein). [Human] {Homo sapiens}, partial (62%)
295	tacattgcaa	30	TC17151	89	0	similar to GP|927736|gb|AAB64908.1||U33050 Ydr492wp; CAI: 0.19 {Saccharomyces cerevisiae}, partial (9%)
296	gcttgccaag	30	TC13777	85	0	
297	cgcggccgtg	30	TC7700	62	0	weakly similar to SP|Q16629|SFR7_HUMAN Splicing factor, arginine/serine-rich 7 (Splicing factor 9G8). [Human] {Homo sapiens}, partial (22%)
298	tgactcataa	30				no_annot
299	tcccgtacat	30				no_annot
300	tgttttgtgt	30				no_annot
301	cattttccag	30	TC7353	70	1	
301	cattttccag	30	AI067442	38	2	
302	gtgaacgtgc	30				no_annot
303	gaaatcgtgg	30	TC12350	49	0	
304	gtgtagctat	30	gi|4138864|gb|AF101196.1|AF101196	25	4	Schistosoma mansoni cytochrome c oxidase subunit 1 (cox1) mRNA, mitochondrial gene encoding mitochondrial protein, complete cds
305	ctgttgcttt	30	TC10668	81	0	weakly similar to GP|15293877|gb|AAK95131.1||AF401559 ribosomal protein L7 {Ictalurus punctatus}, partial (73%)

306	tacacagttt	29	TC8398	92	0	weakly similar to GP|11230766|gb|AAG32076.1||AY008722 GS32 {Mus musculus}, partial (8%)
307	cgcttgtata	29				no_annot
308	taattagttt	29	TC12715	88	0	
309	tatcagcctg	29				no_annot
310	tcgattggtg	29	TC8164	96	0	
311	atatgcatcg	29				no_annot
312	gatagacggt	29				no_annot
313	ttacgccgta	29				no_annot
314	tataccggta	29	TC7890	94	0	weakly similar to SP|O60551|NMT2_HUMAN Glycylpeptide N-tetradecanoyltransferase 2(Peptide N-myristoyltransferase 2) (Myristoyl-CoA:protein N-myristoyltransferase 2) (NMT 2) (Type II N-myristoyltransferase). [Human] {Homo sapiens}, partial (20%)
315	catccgtacc	29	TC6915	77	0	
315	catccgtacc	29	TC7177	54	0	
315	catccgtacc	29	TC19613	55	0	
315	catccgtacc	29	CD116202	7	0	
315	catccgtacc	29	CD087058	36	1	
316	ttgctgattg	29	TC14280	76	0	
317	ataagccaat	29	TC18024	50	0	homologue to SP|P51397|DAP1_HUMAN Death-associated protein 1 (DAP-1). [Human] {Homo sapiens}, partial (13%)
318	ctaaacgggt	29	TC8024	77	0	PIR|A27501|HSHU33 histone H3.3 [validated] - human, complete
319	cggtgtctcg	29				no_annot
320	ggaaccggat	29	TC17077	94	0	weakly similar to GP|11875635|gb|AAG40734.1|AF289539_1|AF289539 PES1 protein {Mus musculus}, partial (24%)
321	tgcattatca	29	TC10753	97	0	weakly similar to GP|161038|gb|AAA29903.1||M15509 major egg antigen {Schistosoma mansoni}, partial (24%)
321	tgcattatca	29	N21748	2	0	
322	gtaggttcca	29				no_annot
323	ccaagtttac	29	TC16898	96	0	

324	tgccgttgat	29	TC10813	96	0	
325	aagttacttc	29	AF395822lSma.95	98	0	Thioredoxin glutathione reductase (TGR)
326	gcggcaatag	28	CD138525	51	0	similar to GPl3540239lgblAAC34362.1llAF013160 NADH dehydrogenase-ubiquinone Fe-S protein 2 precursor {Homo sapiens}, partial (15%)
327	tacacgtaac	28				no_annot
328	gaacctgtgt	28	TC13702	84	0	weakly similar to GPl10178878lemblCAC08449.1llAJ294707 eukaryote initiation factor 2 beta {Gallus gallus}, partial (14%)
328	gaacctgtgt	28	TC13703	62	0	
329	ttatgattgc	28				no_annot
330	gctcactccc	28	TC11594	94	0	homologue to GPl7289279lgblAAF45367.1llAE002611 CG9571-PA {Drosophila melanogaster}, partial (5%)
331	tatacgtgca	28	TC14690	90	0	
332	tcggcgcaat	28	TC14797	48	0	
333	tgttcttgtt	28	TC8185	61	0	
333	tgttcttgtt	28	TC8186	81	0	
333	tgttcttgtt	28	TC10386	52	4	similar to GPl20177041lgblAAM12282.1llAY095189 LD26817p {Drosophila melanogaster}, partial (30%)
334	tccttggcgg	28				no_annot
335	aatgattagg	28	C708031.1	70	0	
336	catccgcacc	28	TC7171	49	0	
337	ttacacggtc	28	TC7540	87	0	similar to SPlQ14498lRNP2_HUMAN RNA-binding region containing protein 2 (Hepatocellular carcinoma protein 1) (Splicing factor HCC1). [Human] {Homo sapiens}, partial (57%)
338	gtcctggatc	28	TC13536	36	3	weakly similar to PIRlA55075lA55075 chaperonin-10 - mouse, partial (97%)
339	taataaatcg	28	TC13731	99	0	SPlQ27778lK6PF_SCHMA 6-phosphofructokinase(Phosphofructokinase) (Phosphohexokinase). [Blood fluke] {Schistosoma mansoni}, complete
340	cactgttccc	27	TC11144	93	0	
341	tagtgtgtcc	27	TC14803	72	1	

342	ttgagttcga	27	TC10783	83	0	similar to GP|5833465|gb|AAD53521.1|AF155581_1|AF155581 proteasome subunit beta 7 {Danio rerio}, partial (75%)
343	cccaagcttt	27				no_annot
344	taaattgtta	27	TC8126	89	0	similar to GP|5052333|gb|AAD38506.1|AF126743_1|AF126743 DNAJ domain-containing protein MCJ {Homo sapiens}, partial (47%)
345	ggaactttgt	27	CD191601	4	0	
346	gcctcgaggt	27	TC14437	88	0	weakly similar to GP|179460|gb|AAA51827.1||M13520 N-acetyl-alpha-glucosaminidase prepro-polypeptide {Homo sapiens}, partial (48%)
347	aaacagtcag	27				no_annot
348	tacatagcgt	27				no_annot
349	ttgttataaa	27	M64538|Sma.63	91	0	Ferritin-1
350	gctctgctaa	27	TC7557	34	0	similar to SP|P10159|IF5A_HUMAN Eukaryotic translation initiation factor 5A (eIF-5A) (eIF-4D) (Rev-binding factor). [Mouse] {Mus musculus}, partial (65%)
351	ttattgtttt	27	TC10433	98	0	weakly similar to GP|12188891|emb|CAC21554.1||AJ298278 poly(A) binding protein {Rattus norvegicus}, partial (47%)
352	tgcatacata	27	CD140403	67	0	
353	aaaaaaaaaa	27	AI447076	81	0	
354	gcagcccaac	27				no_annot
355	tgttgtgtta	27	TC8043	79	2	similar to GP|5305389|gb|AAD41627.1|AF072328_1|AF072328 dynein light chain 2 {Schistosoma japonicum}, complete
355	tgttgtgtta	27	TC9114	3	1	similar to GP|451706|gb|AAA18961.1||U05129 envelope glycoprotein {Simian immunodeficiency virus}, partial (37%)
356	atcacaaaaa	26	M15371|Sma.619	65	0	Eggshell protein mRNA, 3 end
356	atcacaaaaa	26	gi|161049|gb|M74170.1|SCMP48EGG	89	0	Schistosoma mansoni p48 eggshell protein gene, complete cds
357	ggtagatata	26				no_annot
358	taaattcggg	26	TC16982	88	1	similar to GP|7689009|gb|AAF67644.1|AF220051_1|AF220051 uncharacterized hematopoietic stem/progenitor cells protein MDS031 {Homo sapiens}, partial (23%)

359	caacctacgc	26	TC17179	94	0	weakly similar to GP|5679126|gb|AAD46869.1|AF160929_1|AF160929 BcDNA.LD12153 {Drosophila melanogaster}, partial (43%)
360	gacaagtacc	26	TC10736	35	0	similar to GP|22758850|gb|AAN05585.1||AF526199 ribosomal protein L22 {Argopecten irradians}, partial (96%)
361	tgttactcaa	26	AY158214|Sma.118	93	0	Rho2 GTPase (Rho2)
362	aaacaactta	26	TC13813	65	0	homologue to GP|26522784|dbj|BAC44866.1||AB086826 actin {Galaxea fascicularis}, complete
362	aaacaactta	26	TC13814	43	0	homologue to GP|14575741|gb|AAK68711.1|AF329437_1|AF329437 actin {Biomphalaria alexandrina}, partial (52%)
362	aaacaactta	26	R95596	48	0	similar to SP|P35432|ACT1_ECHGR Actin 1. {Echinococcus granulosus}, partial (29%)
363	gctggctctt	26				no_annot
364	tgcattttcg	26	TC13580	98	0	weakly similar to SP|Q00963|SPCB_DROME Spectrin beta chain. [Fruit fly] {Drosophila melanogaster}, partial (15%)
365	ttcaacttcc	26	TC13905	75	0	similar to PIR|S41170|S41170 ribosomal protein S3, cytosolic - mouse, partial (92%)
366	gctacctgat	26	TC13946	96	0	weakly similar to GP|17861540|gb|AAL39247.1||AY069102 GH11824p {Drosophila melanogaster}, partial (11%)
367	tgatttgttt	26	CD096941	5	0	
368	atgatgatga	26	gi|1103828|gb|U31768.1|SMU31768	27	15	Schistosoma mansoni elastase gene, 3045 bp clone, complete cds
369	tgctcgcttt	26	AY722631|Sma.1221	93	0	Clone NS8W-c-10 mRNA sequence
370	atgattcttt	26	TC17453	94	0	similar to SP|Q18421|GRPE_CAEEL GrpE protein homolog, mitochondrial precursor. {Caenorhabditis elegans}, partial (27%)
370	atgattcttt	26	TC17454	87	0	similar to SP|O43047|GRPE_SCHPO GrpE protein homolog, mitochondrial precursor. [Fission yeast] {Schizosaccharomyces pombe}, partial (8%)
371	gttgttcctt	26	TC13607	56	0	similar to SP|P40429|R13A_HUMAN 60S ribosomal protein L13a (23 kDa highly basic protein). [Human] {Homo sapiens}, partial (68%)
372	taaacaaagg	25	TC13465	42	0	

372	taaacaaagg	25	TC13470	80	0	PIR|B54525|B54525 major female-specific polypeptide (frame 2) - fluke (Schistosoma mansoni) (fragment), partial (77%)
372	taaacaaagg	25	TC15674	44	1	
372	taaacaaagg	25	CD200899	61	0	
373	tacaactcgt	25				no_annot
374	tgtgtgtgta	25	AY158217|Sma.772	39	5	Rac GTPase (Rac)
375	agtgctgtca	25				no_annot
376	gtcttcctcg	25	gi|4138864|gb|AF101196.1|AF101196	84	1	Schistosoma mansoni cytochrome c oxidase subunit 1 (cox1) mRNA, mitochondrial gene encoding mitochondrial protein, complete cds
377	tgtacgtgag	25				no_annot
378	aaggacagca	25	C308289.1	73	1	
379	aatacattgg	25	TC7379	74	2	
379	aatacattgg	25	CD124620	51	1	similar to GP|606051|gb|AAA57914.1||U18997 ORF_f425 {Escherichia coli}, partial (6%)
380	gacgtacgtg	25				no_annot
381	tgtgtgggtc	25				no_annot
382	gaaacaacag	25				no_annot
383	cttgcggaga	25				no_annot
384	gcgatcaaag	25	TC17983	30	4	similar to GP|17945451|gb|AAL48779.1||AY071157 RE19416p {Drosophila melanogaster}, partial (17%)
385	tagacaaaga	25	TC7762	77	0	
386	ttgtggcata	25	TC7455	91	0	weakly similar to SP|P13395|SPCA_DROME Spectrin alpha chain. [Fruit fly] {Drosophila melanogaster}, partial (21%)
387	tccaatctgt	25	TC14445	94	0	similar to GP|27820011|gb|AAO25038.1||BT003281 LD01705p {Drosophila melanogaster}, partial (45%)
388	gaaatcctag	25	TC7552	85	0	weakly similar to GP|6759903|gb|AAF28099.1||AF137273 alpha 1 (V) collagen {Gallus gallus}, partial (20%)
389	aaacattcga	25	L25067|Sma.29	95	0	Glucose transporter protein (GTP4)
390	taactgtaga	25	AF030973|Sma.669	81	0	Clone 19 unknown
391	tacattgttt	24	TC13918	87	0	
392	ttggcatctg	24	TC14070	75	0	

393	acaagttatg	24	AY158217|Sma.772	82	0	Rac GTPase (Rac)
394	ttcaatcgat	24				no_annot
395	gtggagatta	24	L01634|Sma.848	36	12	Myosin heavy chain (MYH)
396	gaaactaata	24				no_annot
397	taagtcactt	24	TC17396	96	0	
398	gtgttttgtg	24				no_annot
399	agcaagtgag	24	C602575.1	16	4	
400	gcgccgcttg	24				no_annot
401	tcgggaggta	24	TC11169	16	0	similar to GP|19353381|gb|AAH24355.1||BC024355 Usmg5 protein {Mus musculus}, partial (41%)
401	tcgggaggta	24	TC11170	12	1	similar to GP|19353381|gb|AAH24355.1||BC024355 Usmg5 protein {Mus musculus}, partial (31%)
402	actgtcaata	24				no_annot
403	caatgataac	24	TC10578	66	0	
404	tgcaggtgat	24	TC16843	83	0	similar to GP|25989211|gb|AAL31707.1||AY061936 prophenoloxidase activating factor 3 {Bombyx mori}, partial (10%)
404	tgcaggtgat	24	CD180703	75	0	similar to GP|21645360|gb|AAM70960.1||AE003809 CG30087-PA {Drosophila melanogaster}, partial (8%)
405	gtgattgatt	24	C605265.1	95	0	
406	ttgtccatca	24	TC11765	82	0	weakly similar to GP|12654705|gb|AAH01192.1|AAH01192|BC001192 PTD008 protein {Homo sapiens}, partial (63%)
407	ggatccagtc	24	TC11701	92	0	
408	tatggaatgt	24				no_annot
409	cgtgcaagtg	24	TC7447	6	0	similar to SP|Q02543|RL1X_HUMAN 60S ribosomal protein L18a. [Human] {Homo sapiens}, partial (94%)
410	aaaccaaaca	24				no_annot
411	tatacctgtc	24				no_annot
412	ttttctaaat	24				no_annot
413	catagtttga	24	TC8711	91	0	
414	tgatggcgac	24	L37151|Sma.826	10	0	(clone Smp50) immunophilin

415	cagagctgga	24	TC7888	19	0	similar to SP|Q99462|UBCG_HUMAN Ubiquitin-conjugating enzyme E2 G1(Ubiquitin-protein ligase G1) (Ubiquitin carrier protein G1) (E217K) (UBC7). [Rat] {Rattus norvegicus}, partial (93%)
415	cagagctgga	24	CD081008	31	0	similar to SP|Q99462|UBCG_HUMAN Ubiquitin-conjugating enzyme E2 G1(Ubiquitin-protein ligase G1) (Ubiquitin carrier protein G1) (E217K) (UBC7). [Rat] {Rattus norvegicus}, partial (80%)
416	aaagtgtttc	24	TC10437	88	3	PIR|S34275|S34275 protein disulfide-isomerase homolog precursor - fluke (Schistosoma mansoni), complete
417	gttcgtttct	24	C602647.1	1	0	
418	gcatcagccc	24	TC14803	80	0	
419	aatgtcacta	23	TC17078	96	0	
420	tatgtttata	23	TC16834	94	0	weakly similar to GP|2745838|gb|AAB94760.1||AF039202 Hsp70/Hsp90 organizing protein; hop {Cricetulus griseus}, partial (28%)
421	cttttgatgt	23	TC10552	85	0	
421	cttttgatgt	23	TC10553	82	0	
421	cttttgatgt	23	CD095658	63	1	
421	cttttgatgt	23	CD124143	30	0	weakly similar to GP|1621210|emb|CAA66711.1||X98086 fbiD {Bacillus intermedius}, partial (8%)
422	gatattggta	23	TC11209	25	0	weakly similar to GP|6526355|dbj|BAA88073.1||AB002282 hMBF1alpha {Homo sapiens}, partial (39%)
423	cagtggacaa	23	TC7882	84	0	similar to GP|27354500|dbj|BAC51487.1||AP005957 bll6222 {Bradyrhizobium japonicum USDA 110}, partial (9%)
424	gtttcttcac	23				no_annot
425	atacgtgtgc	23				no_annot
426	tctgtctatc	23	L08641|Sma.630	89	0	SmIrV1 protein
427	ttgtaccgtg	23				no_annot
428	cataatactt	23	TC16746	91	0	similar to GP|20977567|gb|AAM28211.1||AY099523 vacuolar ATP synthase 16 kDa proteolipid subunit {Danio rerio}, partial (89%)
429	tgtttagccc	23	C315093.1	24	1	
429	tgtttagccc	23	C714192.1	24	1	

430	tgatatatgc	23	TC10376	95	0	similar to GP|22832080|gb|AAF48002.2||AE003485 CG2522-PA {Drosophila melanogaster}, partial (40%)
431	acagtctggt	23	CD072947	16	2	similar to GP|10179946|gb|AAG13956.1||AF283991 (N6-adenosine)-methyltransferase {Homo sapiens}, partial (14%)
432	atgatctaac	23	TC7679	96	0	weakly similar to GP|28630303|gb|AAM92833.1||AY130428 protein kinase C {Branchiostoma lanceolatum}, partial (7%)
433	tatccttacc	23	TC7857	62	0	weakly similar to GP|27762280|gb|AAO17575.1||AY188775 dihydrolipoamide S-acetyltransferase {Danio rerio}, partial (44%)
433	tatccttacc	23	TC16780	61	2	similar to SP|P25867|UBC1_DROME Ubiquitin-conjugating enzyme E2-17 kDa(Ubiquitin-protein ligase) (Ubiquitin carrier protein) (Effete protein). [Fruit fly] {Drosophila melanogaster}, partial (95%)
434	gcagcgtaaa	23	TC10403	74	0	weakly similar to GP|25012791|gb|AAN71487.1||BT001732 RE70963p {Drosophila melanogaster}, partial (41%)
434	gcagcgtaaa	23	TC11119	28	2	weakly similar to GP|24980875|gb|AAH39758.1||BC039758 Fms interacting protein {Mus musculus}, partial (10%)
435	agtaactcct	23				no_annot
436	taatgcattt	23				no_annot
437	acacttacta	23				no_annot
438	ggccaggttt	23	TC8825	78	0	weakly similar to GP|7292570|gb|AAF47970.1||AE003484 CG1637-PA {Drosophila melanogaster}, partial (29%)
439	tgcgtgaaca	22				no_annot
440	atcttactgc	22	TC10954	97	0	weakly similar to GP|16768388|gb|AAL28413.1||AY060865 GM03559p {Drosophila melanogaster}, partial (55%)
441	tgttattgaa	22	TC7564	79	0	weakly similar to GP|5902663|gb|AAC13264.2||AF029844 elongation factor 1-beta homolog {Mus musculus}, partial (67%)
442	tattttgggt	22	CD181366	78	0	

443	atgaaaaaaa	22	TC9958	17	1	similar to GP|24986118|gb|AAN69972.1|AE016635_8|AE016790 flagella basal body P-ring formation protein FlgA {Pseudomonas putida KT2440}, partial (34%)
444	tggaagtcag	22	TC10467	35	4	GP|8132322|gb|AAF73250.1|AF153977_1|AF153977 eukaryotic translation initiation factor 5 {Schistosoma mansoni}, complete
444	tggaagtcag	22	TC10470	9	3	GP|8132322|gb|AAF73250.1|AF153977_1|AF153977 eukaryotic translation initiation factor 5 {Schistosoma mansoni}, partial (83%)
445	cactcgattt	22	C604615.1	97	0	
446	cacacacaca	22	TC9491	58	0	
446	cacacacaca	22	TC13956	21	5	
446	cacacacaca	22	TC16613	35	2	
446	cacacacaca	22	CD097719	8	2	
446	cacacacaca	22	CD120338	58	1	
447	ataacttgta	22	TC14542	94	0	weakly similar to PIR|I48080|I48080 coatomer complex epsilon chain - Chinese hamster, partial (33%)
447	ataacttgta	22	TC19449	70	0	
448	tgatcaatct	22	AI740326	31	0	
449	ggtattgctc	22	TC10547	7	0	homologue to GP|28916495|gb|AAO59416.1||AY226986 ribosomal protein S8 {Schistosoma japonicum}, complete
450	tcttcgtctc	22	C202442.1	94	0	
450	tcttcgtctc	22	C605669.1	94	0	
451	acagataaaa	22	TC17025	95	0	similar to GP|21666610|gb|AAM73775.1|AF427342_1|AF427342 glutamate dehydrogenase 1 {Oncorhynchus mykiss}, partial (81%)
452	ttttggcaga	22	TC12029	39	0	
453	ttcatacacc	22				no_annot
454	aaaatcgata	22	TC17035	2	2	
455	tacagaaccg	22				no_annot
456	gcaccagtta	22	C201330.1	79	0	
456	gcaccagtta	22	C609794.1	39	2	

457	gtatctatcc	22	TC10626	89	0	weakly similar to GP|3142634|gb|AAC78612.1||AF063665 small nuclear ribonucleoprotein N {Mus musculus}, partial (36%)
458	gtgttcactt	22	TC7556	79	0	similar to SP|Q24400|MLP2_DROME Muscle LIM protein MLP84B. [Fruit fly] {Drosophila melanogaster}, partial (28%)
459	tgctgtgact	22	CD090739	52	0	
460	agtaatatga	22	TC11303	80	0	
461	taaatgccac	22	TC11759	94	0	similar to GP|7290709|gb|AAF46156.1||AE003437 CG3842-PA {Drosophila melanogaster}, partial (18%)
462	gggagacaga	22	TC16916	40	0	
463	aaagtcaaag	22	TC7713	44	0	similar to PIR|JE0129|JE0129 ribosomal protein S14 - mouse, partial (89%)
464	ctcaatatgc	22	AF484940|Sma.77	80	0	Glutathione S-transferase omega
465	tataatgtcg	22	TC10522	90	0	similar to GP|15042332|gb|AAK82112.1|AF303741_251|AF303741 251L {Chilo iridescent virus}, partial (9%)
466	ttatgtttcc	22	TC17489	81	1	weakly similar to PIR|B38128|B38128 epithelin/granulin precursor - rat, partial (13%)
467	ttgacaccaa	22	TC13801	50	0	
468	gaatatccca	22				no_annot
469	gcgaagagca	22				no_annot
470	taatgtatga	21	TC9458	96	0	
471	aaacaccgca	21	CD131654	75	0	
472	tatttgtgac	21	AF030969|Sma.246	93	0	Clone 10 unknown
473	cagcccctaa	21	TC19181	86	0	
474	gtcaagaaga	21	TC13566	51	2	weakly similar to SP|P40423|MLRN_DROME Myosin regulatory light chain, nonmuscle (MRLC-C) (Spaghetti-squash protein). [Fruit fly] {Drosophila melanogaster}, partial (87%)
475	ttgtagcctc	21	TC14187	78	0	
476	gctgagcagc	21	TC8131	2	0	similar to SP|P04643|RS11_HUMAN 40S ribosomal protein S11. [Rat] {Rattus norvegicus}, partial (75%)
477	gctcagtgtt	21	TC10511	94	0	weakly similar to GP|28564801|dbj|BAC57731.1||AP004401 P0534A03.23 {Oryza sativa (japonica cultivar-group)}, partial (9%)

478	tttaactttc	21	TC10897	90	0	similar to GP|16305123|gb|AAL16984.1||AF371268 19kD alpha zein D2 {Zea mays}, partial (12%)
479	ccggtggcgg	21	TC10781	91	0	similar to SP|Q03577|SYD_CAEEL Aspartyl-tRNA synthetase(Aspartate--tRNA ligase) (AspRS). {Caenorhabditis elegans}, partial (67%)
480	gatggattga	21	TC17432	19	3	
481	ttttcttgaa	21	AF217404|Sma.771	97	0	Endoplasmin
482	agttgccata	21				no_annot
483	gtggagacga	21	TC7784	16	2	similar to GP|21166150|gb|AAM43767.1||AC117081 Mitochondrial genome maintenance protein MGM101 precursor. {Dictyostelium discoideum}, partial (5%)
483	gtggagacga	21	TC15401	5	1	weakly similar to GP|22778426|dbj|BAC14695.1||AP004602 transcriptional regulator {Oceanobacillus iheyensis}, partial (18%)
484	ttgatcacaa	21				no_annot
485	actacttttg	21	TC8157	72	0	similar to PIR|G02666|G02666 cysteine-rich protein 1 - human, partial (84%)
486	gacctgggta	21				no_annot
487	gaaatgcact	21	C604345.1	18	3	
488	cgacgcccca	21	TC16536	79	0	PIR|AF2453|AF2453 oxidoreductase alr5182 [imported] - Nostoc sp. (strain PCC 7120), partial (5%)
489	cagatttcaa	21				no_annot
490	tgcgaacatc	21	TC17285	69	0	homologue to GP|2636680|gb|AAC06263.1||U66331 pol {Schistosoma mansoni}, partial (8%)
491	gaagctaggc	21	TC11530	80	0	similar to GP|10727295|gb|AAF47841.2||AE003480 CG14998-PB {Drosophila melanogaster}, partial (5%)
492	tcattttgct	21				no_annot
493	tatactgaat	21	TC11431	93	0	similar to PIR|T13614|T13614 N-acetyltransferase homolog 8D8.6 - fruit fly (Drosophila melanogaster), partial (28%)
494	ggaaattgaa	21	CD073104	23	2	
495	taatgtgatt	21	TC16889	84	0	similar to SP|P04182|OAT_RAT Ornithine aminotransferase, mitochondrial precursor(Ornithine--oxo-acid aminotransferase). [Rat] {Rattus norvegicus}, partial (86%)
496	aagtgttgat	21				no_annot
497	atgatgatta	21	TC7779	92	0	

498	taatatacgt	21	TC11322	96	0	similar to GP|21428402|gb|AAM49861.1||AY118492 LD05267p {Drosophila melanogaster}, partial (21%)
499	aagacaggga	21				no_annot
500	tgctctagtc	21	TC13765	91	0	weakly similar to GP|16769746|gb|AAL29092.1||AY061544 LP04475p {Drosophila melanogaster}, partial (50%)
501	ggaagttgaa	20	TC17069	39	0	weakly similar to GP|6984224|gb|AAF34800.1|AF227980_1|AF227980 60S ribosomal protein L35 {Euphorbia esula}, partial (48%)
502	tgaggcttcg	20	TC10762	23	3	weakly similar to GP|14025157|dbj|BAB51758.1||AP003006 glucose dehydrogenase {Mesorhizobium loti}, partial (13%)
503	aaatataagt	20	TC13620	98	0	similar to SP|P78371|TCPB_HUMAN T-complex protein 1, beta subunit (TCP-1-beta) (CCT-beta). [Human] {Homo sapiens}, partial (96%)
504	gccggccgat	20	TC13734	28	0	similar to SP|Q25531|VA0D_MANSE Vacuolar ATP synthase subunit d(V-ATPase d subunit) (Vacuolar proton pump d subunit) (V-ATPase 40 KDa subunit) (M40). [Tobacco hawkmoth, Tobacco hornworm] {Manduca sexta}, partial (98%)
505	aacaaatgat	20				no_annot
506	tagaagtcat	20	TC6874	75	0	similar to GP|16768348|gb|AAL28393.1||AY060845 GM02242p {Drosophila melanogaster}, partial (72%)
507	tacacttttg	20	TC10089	95	0	
508	gtatcgtatc	20	TC8149	65	0	
509	gccagtacct	20	TC10916	82	0	similar to GP|25990444|gb|AAN76523.1|AF383945_1|AF383945 MAGO protein {Gallus gallus}, complete
510	gaccacgtgt	20	TC10982	67	0	
510	gaccacgtgt	20	CD080992	93	0	
511	ttcgtgttgc	20	TC10983	50	0	similar to PIR|T49255|T49255 protein kinase-like - Arabidopsis thaliana, partial (7%)
511	ttcgtgttgc	20	TC13384	66	1	
512	gcatagctct	20	TC10178	81	0	weakly similar to GP|7291314|gb|AAF46744.1||AE003454 CG10320-PA {Drosophila melanogaster}, partial (27%)
513	tctacgtttc	20	U39883|Sma.210	62	0	Y-box binding protein

514	gtgtagcgct	20	TC9164	88	0	
515	caaaagaagt	20	M99494|Sma.23	96	0	200 kDa surface protein
516	ggggaggggt	20				no_annot
517	ttaccgctaa	20				no_annot
518	acagctacag	20	TC17269	57	0	weakly similar to GP|23093913|gb|AAF50410.2||AE003555 CG6694-PA {Drosophila melanogaster}, partial (8%)
519	tcggcactga	20				no_annot
520	ttctgaaggc	20	TC13903	63	0	weakly similar to SP|Q9JID6|LCFA_CAVPO Long-chain-fatty-acid--CoA ligase 1(Long-chain acyl-CoA synthetase 1) (LACS 1) (Palmitoyl-CoA ligase). [Guinea pig] {Cavia porcellus}, partial (12%)
521	tatattcagc	20				no_annot
522	taacaaactg	20				no_annot
523	aagtctatgg	20				no_annot
524	ctgaatgttt	19				no_annot
525	ttacgcaaga	19	TC13718	77	2	similar to GP|1262435|emb|CAA61864.1||X89719 put. 26S protease subunit {Sus scrofa}, partial (96%)
526	ttattggtga	19	TC10353	38	2	GP|160925|gb|AAA29853.1||M94346 A.1.12/9 antigen {Schistosoma mansoni}, partial (86%)
526	ttattggtga	19	TC10799	19	5	weakly similar to GP|4138422|emb|CAA04022.1||AJ000347 3'(2'),5'-bisphosphate nucleotidase {Rattus norvegicus}, partial (38%)
526	ttattggtga	19	TC10800	6	2	weakly similar to GP|21428526|gb|AAM49923.1||AY118554 LD34542p {Drosophila melanogaster}, partial (18%)
527	ttacatcctg	19	C606069.1	96	0	
528	ctaaatacaa	19	AF025539|Sma.5	28	0	Ribosomal protein smL37a
529	ttgcaagagt	19	N33643	31	0	
530	tttgttctct	19	C200397.1	97	1	
530	tttgttctct	19	C201473.1	92	0	
530	tttgttctct	19	C608674.1	88	0	
530	tttgttctct	19	C717678.1	83	1	

531	gtgatttata	19	TC8477	93	0	similar to GP|9294023|dbj|BAB01926.1||AP001307 multispanning membrane protein-like {Arabidopsis thaliana}, partial (3%)
532	gataatggtg	19	TC10544	82	0	weakly similar to GP|11527011|gb|AAG36874.1|AF242767_1|AF242767 SF2 {Caenorhabditis elegans}, partial (18%)
532	gataatggtg	19	TC10545	83	0	weakly similar to GP|18034491|gb|AAL57515.1|AF449428_1|AF449428 SRrp35 {Homo sapiens}, partial (23%)
532	gataatggtg	19	TC10546	95	0	
533	taagcccggt	19	TC15032	75	0	
534	gttattgaaa	19	TC11940	80	0	
534	gttattgaaa	19	CD190756	84	0	
535	tattgtttct	19	TC10742	91	0	similar to SP|P53488|ARP2_CHICK Actin-like protein 2 (Actin-like protein ACTL). [Chicken] {Gallus gallus}, partial (92%)
535	tattgtttct	19	CD179365	55	0	
536	gtaaacatta	19	AF065599|Sma.36	11	3	Annexin
537	cgacctgtcg	19	C605478.1	94	0	
538	gccaaagtaa	19				no_annot
539	gcacaacaaa	19				no_annot
540	atgcaccgtc	19	TC7826	85	0	weakly similar to GP|3789911|gb|AAC67541.1||AF081802 developmental protein DG1118 {Dictyostelium discoideum}, partial (34%)
540	atgcaccgtc	19	CD076764	47	0	
541	tacattttga	19	TC7445	91	2	similar to GP|17298103|dbj|BAB78518.1||AB062670 DSec61alpha {Drosophila melanogaster}, partial (76%)
542	tgtttgcgaa	19	TC8086	98	0	similar to GP|16611713|gb|AAL27306.1|AF376481_1|AF376481 BgaC {Carnobacterium piscicola}, partial (8%)
543	ctgattgttg	19				no_annot
544	gttattatta	19	TC10089	16	1	
544	gttattatta	19	TC10413	34	0	
544	gttattatta	19	TC10414	90	0	similar to GP|2541914|dbj|BAA22852.1||AB008005 troponin I {Mizuhopecten yessoensis}, partial (25%)

544	gttattatta	19	TC17855	94	0	similar to GP|23094223|gb|AAF51695.2||AE003594 CG9390-PA {Drosophila melanogaster}, partial (10%)
545	tataccagta	19				no_annot
546	gtaacactat	19	TC17460	94	0	weakly similar to GP|18447650|gb|AAL68385.1||AY075580 SD07362p {Drosophila melanogaster}, partial (11%)
546	gtaacactat	19	CD165745	52	0	
547	taatcagttg	19	C211196.1	9	2	
548	taccagatgc	19				no_annot
549	ggcaactaca	19	TC16936	55	0	weakly similar to PIR|E86466|E86466 protein F23M19.10 [imported] - Arabidopsis thaliana, partial (4%)
550	catagcacat	19				no_annot
551	aggaatatgg	19	N21732	2	0	
552	cttcggtacg	19				no_annot
553	taaacatatg	18				no_annot
554	gttaatatcc	18	TC14109	13	0	similar to GP|22203732|gb|AAM94276.1||AF526255 ribosomal protein L44 {Chlamys farreri}, partial (97%)
555	gcattgtatt	18	TC10527	41	1	homologue to GP|15545995|gb|AAK38749.1||AY029255 dynein light chain 2 {Mus musculus}, complete
555	gcattgtatt	18	TC10529	31	1	similar to SP|O02414|DYL1_ANTCR Dynein light chain LC6, flagellar outer arm. [Sea urchin] {Anthocidaris crassispina}, complete
555	gcattgtatt	18	TC10531	38	1	similar to GP|15545995|gb|AAK38749.1||AY029255 dynein light chain 2 {Mus musculus}, partial (96%)
555	gcattgtatt	18	TC10532	43	0	similar to SP|O96860|DYL2_DROME Dynein light chain 2, cytoplasmic (8 kDa dynein light chain). [Fruit fly] {Drosophila melanogaster}, partial (66%)
555	gcattgtatt	18	TC14891	52	0	homologue to GP|5305391|gb|AAD41628.1|AF072329_1|AF072329 dynein light chain 3 {Schistosoma japonicum}, partial (94%)
555	gcattgtatt	18	CD079649	58	2	
556	gtcaatgcaa	18	TC18105	93	0	
557	ttgtaagcga	18				no_annot

558	agatcggttc	18	TC7633	96	0	homologue to GP|6469318|gb|AAF13300.1|U83399_1|U83399 filamin {Schistosoma japonicum}, partial (95%)
559	gatcgatgag	18	TC10950	85	0	weakly similar to PIR|I50090|I50090 carboxypeptidase gp180 - Anas sp., partial (7%)
560	acgataatga	18	CD074117	7	5	similar to GP|5869817|emb|CAB55574.1||AJ249395 cytochrome oxidase subunit III {Globodera pallida}, partial (6%)
561	tacaagtgcc	18	TC10851	75	0	similar to GP|21430254|gb|AAM50805.1||AY118945 LD31024p {Drosophila melanogaster}, partial (30%)
561	tacaagtgcc	18	TC10852	73	2	weakly similar to GP|21430254|gb|AAM50805.1||AY118945 LD31024p {Drosophila melanogaster}, partial (29%)
562	taatttagtt	18	TC16859	84	2	similar to SP|P27080|ADT_CHLRE ADP,ATP carrier protein (ADP/ATP translocase) (Adenine nucleotide translocator) (ANT). {Chlamydomonas reinhardtii}, partial (52%)
562	taatttagtt	18	TC16860	86	0	similar to PIR|T04608|T04608 ADP,ATP carrier protein F20O9.60 - Arabidopsis thaliana, partial (17%)
563	gaaaaaaaaa	18				no_annot
564	tttgccagtc	18	TC13995	51	0	homologue to GP|29650459|gb|AAO86771.1||AY241391 casein kinase II beta subunit {Schistosoma japonicum}, complete
565	tatctgtcgc	18				no_annot
566	ttgattatcc	18				no_annot
567	atgcatttca	18	TC13655	92	0	similar to GP|1916290|gb|AAC53117.1||U89876 ALY {Mus musculus}, partial (9%)
568	cagtgccata	18	TC7873	52	0	
568	cagtgccata	18	TC7874	76	0	
569	tcagagatgt	18	CD117999	0	1	
570	tgttaattgt	18				no_annot
571	tgttcctgtc	18	TC7855	74	0	
572	gataccattt	18	TC19264	86	0	
573	ggtgggatgt	18	TC8763	40	2	weakly similar to GP|19071882|dbj|BAB85678.1||AB058634 small integral membrane protein of lysosome/late endosome {Gallus gallus}, partial (33%)

574	acgacctggt	18	TC17020	85	0	similar to SP|Q01081|U2AG_HUMAN Splicing factor U2AF 35 kDa subunit (U2 auxiliary factor 35 kDa subunit) (U2 snRNP auxiliary factor small subunit). [Human] {Homo sapiens}, partial (51%)
575	gagttttgta	18	C201389.1	95	0	
575	gagttttgta	18	C608676.1	94	0	
576	gtgaattaga	18				no_annot
577	tataaaagca	18	TC10610	99	0	GP|808821|gb|AAA96714.1||L40328 ATPase {Schistosoma mansoni}, complete
578	gaacaaacga	18	TC14347	80	2	weakly similar to SP|O43181|NUYM_HUMAN NADH-ubiquinone oxidoreductase 18 kDa subunit, mitochondrial precursor(Complex I-18 kDa) (CI-18 kDa) (Complex I- AQDQ) (CI-AQDQ). [Human] {Homo sapiens}, partial (46%)
579	tatggaattc	18	TC13952	87	0	weakly similar to SP|P36543|VATE_HUMAN Vacuolar ATP synthase subunit E(V-ATPase E subunit) (Vacuolar proton pump E subunit) (V-ATPase 31 kDa subunit) (P31). [Human] {Homo sapiens}, partial (75%)
580	gagaattact	18				no_annot
581	cttaataaca	18	TC6886	87	0	weakly similar to GP|3746842|gb|AAC64086.1||AF083385 30kDa splicing factor; SPF 30 {Homo sapiens}, partial (29%)
581	cttaataaca	18	TC6951	92	0	similar to GP|16769358|gb|AAL28898.1||AY061350 LD28068p {Drosophila melanogaster}, partial (28%)
582	ccaagcttac	18				no_annot
583	tctgtaaact	18				no_annot
584	gttgttgttg	18	CD088447	94	0	
585	cctcgacaag	18	AI739696	10	0	weakly similar to GP|24059819|dbj|BAC21648.1||AB093674 ribosomal protein L38 {Macaca fascicularis}, partial (54%)
586	gaacaatatt	18	TC10473	71	3	weakly similar to SP|Q01105|SET_HUMAN SET protein (HLA-DR associated protein II) (PHAPII) (Phosphatase 2A inhibitor I2PP2A). [Human] {Homo sapiens}, partial (68%)
587	ttattcgccc	18	AA525617	29	0	
588	gacttagatt	18	AF030968|Sma.776	93	0	Clone 9 unknown
589	gcgagcagaa	18	TC7340	60	2	This is the longest open reading frame of the sequence that starts with Met

589	gcgagcagaa	18	TC7349	48	5	
589	gcgagcagaa	18	R95579	48	1	
590	ttgacttggt	18				no_annot
591	ttatttgatt	18	TC7645	96	0	
592	tcaatcatta	17	AF101194|Sma.86	13	24	Receptor tyrosine kinase
593	aattggtttc	17	TC10570	88	0	
594	tcgacgactg	17				no_annot
595	acatcaacta	17				no_annot
596	gttaatttgt	17	BE431262	27	0	homologue to GP|3549650|emb| alpha-amylase {Avena fatua}, partial (2%)
597	gcgtggatgt	17	TC7060	69	0	weakly similar to SP|Q9UL36|Z236_HUMAN Zinc finger protein 236. [Human] {Homo sapiens}, partial (3%)
598	ccataacacg	17				no_annot
599	gtgattacgg	17	TC13844	32	3	
600	aacctggtgt	17				no_annot
601	aaatataacg	17	TC10683	98	0	similar to GP|21711773|gb|AAM75077.1||AY128484 RE61939p {Drosophila melanogaster}, partial (91%)
602	gttgaaaaag	17				no_annot
603	acgaaagaac	17				no_annot
604	tgtacgtgat	17				no_annot
605	tgctggttta	17				no_annot
606	aggtccgtta	17				no_annot
607	ggatggaaga	17	TC13162	18	0	
607	ggatggaaga	17	TC16014	21	0	
608	aaatgatttt	17	M27529|Sma.142	89	0	Superoxide dismutase
609	atgcgtcaaa	17	TC7952	86	0	
610	atactcacga	17	R95494	27	0	weakly similar to GP|1519552|gb|AAB07546.1||U67153 13 kDa tegumental antigen Sm13 {Schistosoma mansoni}, partial (31%)
611	tttggctcgg	17	TC7710	86	0	weakly similar to GP|2624407|emb|CAA70732.1||Y09537 beta-1,2-N-acetylglucosaminyltransferase II {Sus scrofa}, partial (18%)

612	tgaatagtaa	17	TC7669	98	0	similar to PIR|T40486|T40486 phosphoinositide-dependent protein kinase 1SPBC4C3.11 [imported] - fission yeast (Schizosaccharomyces pombe), partial (5%)
613	acattgtcag	17	TC7859	97	0	similar to GP|2315354|gb|AAB65902.1||AF016440 Adaptin or adaptin-related protein protein 2 {Caenorhabditis elegans}, partial (95%)
614	tgcagtacat	17				no_annot
615	gtgtaacgaa	17				no_annot
616	gggaaaagat	17				no_annot
617	acgtttatat	17	TC10875	83	0	similar to GP|18480206|gb|AAL61117.1||AY073454 olfactory receptor MOR174-10 {Mus musculus}, partial (6%)
618	cccaatggcc	17	TC17202	75	2	weakly similar to PIR|I48702|S54181 p38-2G4 protein - mouse, partial (40%)
619	tttcacgaat	17	TC8442	97	0	weakly similar to GP|21428486|gb|AAM49903.1||AY118534 LD26412p {Drosophila melanogaster}, partial (27%)
620	tgtacacttt	17	AA218490	83	0	
621	tgcttgttaa	17	C201582.1	68	2	
621	tgcttgttaa	17	C604741.1	58	3	
622	gatgggaaaa	17	AY118110|Sma.87	25	0	Immunophilin FK506 binding protein FKBP12
623	tatgtagaat	17	TC10791	89	0	
624	caggaaaatg	17				no_annot
625	ctttacaggc	17	TC7386	71	0	SP|P13566|CABP_SCHMA Calcium-binding protein (CABP). [Blood fluke] {Schistosoma mansoni}, partial (48%)
626	gttgtaaaac	17				no_annot
627	gaccaggtgg	17	TC17042	41	1	similar to GP|13529545|gb|AAH05489.1||BC005489 Sacm1l protein {Mus musculus}, partial (21%)
627	gaccaggtgg	17	TC17043	69	0	similar to GP|16741488|gb|AAH16559.1|AAH16559|BC016559 suppressor of actin 1 {Homo sapiens}, partial (19%)
628	ttcggaagat	17	TC16803	29	1	weakly similar to GP|15293875|gb|AAK95130.1||AF401558 ribosomal protein L6 {Ictalurus punctatus}, partial (50%)

628	ttcggaagat	17	AI977390	85	0	weakly similar to EGAD|64198|67096 ribosomal protein L6 {Rattus norvegicus}, partial (20%)
629	ctggtggaga	16	TC17080	53	0	weakly similar to GP|28630356|gb|AAN73382.1||AY130455 ribosomal protein L18 {Petromyzon marinus}, complete
630	gatgatggta	16	TC7913	52	1	weakly similar to GP|8918685|dbj|BAA97750.1||AB039932 hutI gene homolog {Rhizobium rhizogenes} [Agrobacterium rhizogenes], partial (7%)
631	actgttgaag	16	TC17064	93	0	weakly similar to GP|12656639|gb|AAK00958.1|AF326987_1|AF326987 equilibrative nucleoside transporter 3 {Homo sapiens}, partial (16%)
632	catataccaa	16	CD081015	64	0	
633	cacaaatggg	16				no_annot
634	aaattgtccc	16				no_annot
635	tttacgatgt	16	TC14162	76	0	weakly similar to PIR|S10501|LUCH2 annexin II - chicken, partial (9%)
636	gcacagtaac	16	C205450.1	36	2	
636	gcacagtaac	16	C601402.1	38	2	
637	agtttattgc	16				no_annot
638	tcatcttgtc	16	TC16813	59	1	GP|4160167|emb|CAA10601.1||AJ132193 calmodulin {Caenorhabditis elegans}, complete
639	actgcattaa	16				no_annot
640	ggcacgtgag	16				no_annot
641	tctttgtgtg	16				no_annot
642	actatactac	16	TC11245	95	0	similar to GP|7297705|gb|AAF52957.1||AE003628 CG5322-PA {Drosophila melanogaster}, partial (5%)
643	ttttgcactg	16				no_annot
644	gcaattgaac	16	TC10585	79	0	similar to SP|P25705|ATPA_HUMAN ATP synthase alpha chain, mitochondrial precursor. [Human] {Homo sapiens}, partial (92%)
645	atggtgttgc	16	TC9868	26	0	
645	atggtgttgc	16	TC11705	58	0	
645	atggtgttgc	16	TC16229	76	0	

645	atggtgttgc	16	TC16875	57	1	similar to GP|5901860|gb|AAD55438.1|AF181653_1|AF181653 BcDNA.LD23634 {Drosophila melanogaster}, partial (3%)
645	atggtgttgc	16	TC16876	95	0	
645	atggtgttgc	16	TC17840	25	3	similar to GP|151463|gb|AAA25945.1||M24281 pilin {Pseudomonas aeruginosa}, partial (17%)
645	atggtgttgc	16	TC19198	77	0	
646	gctattcggg	16				no_annot
647	aaacaggtga	16	AA143873	24	0	
648	cgaagaaggc	16	TC10521	77	0	weakly similar to GP|3790696|gb|AAC68756.1||AF099913 Paralysed arrest at two-fold protein 4 {Caenorhabditis elegans}, partial (10%)
649	tggataagag	16				no_annot
650	tggaaagcaa	16	TC11272	80	0	similar to PIR|JC2378|JC2378 acetyl-CoA C-acetyltransferase, cytosolic - human, partial (32%)
651	tgacacttat	16	R95551	15	0	homologue to GP|3789779|gb|A MHC class I related protein precursor {Callithrix argentata}, partial (4%)
652	tcaactagtt	16	TC14386	96	0	weakly similar to SP|Q9UIJ5|ZDH2_HUMAN Zinc finger DHHC domain containing protein 2 (Zinc finger protein 372) (Reduced expression associated with metastasis protein) (Ream) (Reduced expression in cancer protein) (Rec). [Human] {Homo sapiens}, partial (20%)
652	tcaactagtt	16	TC17856	77	1	
653	tcaaagtgat	16				no_annot
654	ctgattgtat	16	U53177|Sma.9	96	0	Ras protein homolog Psmras1 (Smras1)
655	tttggatcct	16	TC17131	38	0	
656	aaagtgtgtg	16	TC14016	84	2	weakly similar to GP|29897169|gb|AAP10446.1||AE017009 Sulfide-quinone reductase {Bacillus cereus ATCC 14579}, partial (31%)
657	gctgaagtga	16	TC7746	82	0	similar to GP|20152085|gb|AAM11402.1||AY095074 RE21802p {Drosophila melanogaster}, partial (92%)
658	caataaagtg	16				no_annot
659	caactgacag	16	TC15195	93	0	similar to GP|16768368|gb|AAL28403.1||AY060855 GM03174p {Drosophila melanogaster}, partial (56%)

660	tcattctccg	16	TC10833	98	0	
661	ttgttcatcg	16	TC13757	51	0	similar to SP|P25789|PSA4_HUMAN Proteasome subunit alpha type 4(Proteasome component C9) (Macropain subunit C9) (Multicatalytic endopeptidase complex subunit C9) (Proteasome subunit L). [Human] {Homo sapiens}, partial (81%)
662	tgatagcaat	16	TC10921	95	0	similar to PIR|A55262|A55262 protein kinasecdc2-related PITALRE - human, partial (47%)
663	taatttgctg	16	AA999294	87	0	
664	tgcatattcc	16	AA999590	31	0	
665	tgggtttgtg	16	TC8400	76	2	weakly similar to PIR|S65953|S65953 [phosphorylase] phosphatase65K regulatory chain isotype alpha - African clawed frog, partial (25%)
666	aaacgttgat	15	TC8274	97	0	
667	tgcagtgatg	15				no_annot
668	aaccccttat	15	TC7833	91	2	similar to PIR|JX0159|KIHUR1 ribose-phosphate diphosphokinasecatalytic chain I - human, complete
669	ttctgttatt	15	TC16896	93	0	similar to SP|P80317|TCPZ_MOUSE T-complex protein 1, zeta subunit (TCP-1-zeta) (CCT-zeta) (CCT-zeta- 1). [Mouse] {Mus musculus}, partial (92%)
670	gacgaaccaa	15	TC16949	28	0	
671	taccggattt	15	TC16734	90	0	similar to GP|18478500|dbj|BAB84523.1||AB067728 xEB1B {Xenopus laevis}, partial (47%)
672	ggattggaac	15	TC8150	52	0	
673	gcacttatag	15				no_annot
674	tgttcgaaga	15	AY669150|Sma.1218	79	0	Methionine sulfoxide reductase B2b (msrB)
675	ccaatcggct	15	TC17480	58	0	
676	gaaacaatat	15	TC17056	71	2	
676	gaaacaatat	15	CD111345	2	0	
677	acaaacagct	15				no_annot
678	ttcgtgtggg	15				no_annot
679	cgaacctgat	15	TC14846	65	0	similar to GP|2257631|dbj|BAA21484.1||D85135 cdc2-related kinase {Bombyx mori}, partial (36%)
680	tttagtgaat	15				no_annot

681	ccagaatcac	15	TC16972	83	1	similar to PIR|D53737|D53737 phosphate carrier protein precursor, mitochodrial, splice form B - bovine, partial (82%)
682	ttctctgcta	15	TC14248	86	0	weakly similar to GP|5670007|gb|AAD46560.1||AF156102 ELL complex EAP30 subunit {Homo sapiens}, partial (26%)
683	acctacgtga	15				no_annot
684	tatagtgatg	15	TC17940	89	0	similar to SP|Q13216|CSA_HUMAN Cockayne syndrome WD-repeat protein CSA. [Human] {Homo sapiens}, partial (5%)
685	ttgataatca	15	TC17054	97	0	weakly similar to GP|6002571|gb|AAF00041.1|AF091370_1|AF091370 hnRNP I-related RNA transport protein VgRBP60 {Xenopus laevis}, partial (34%)
685	ttgataatca	15	CD128556	76	0	
685	ttgataatca	15	CD163887	38	1	
686	acatacgtaa	15	C204910.1	91	0	
686	acatacgtaa	15	C610024.1	84	0	
687	tcttaatgtt	15	TC19076	75	0	
688	cttgtcatta	15	TC16548	96	0	weakly similar to GP|8515718|gb|AAF76141.1||AF257659 crocalbin-like protein {Homo sapiens}, partial (61%)
689	tacagcgtcc	15	AF301001|Sma.654	11	0	Thioredoxin peroxidase 3 (TPx3)
690	ggtttcactt	15				no_annot
691	ccggttaatg	15	TC10471	87	2	similar to PIR|S05988|S05988 translation elongation factor eEF-2 - fruit fly (Drosophila melanogaster), partial (90%)
692	ttcagtgctt	15	TC10409	19	1	weakly similar to GP|16197913|gb|AAL13720.1||AY058491 GM14452p {Drosophila melanogaster}, partial (45%)
693	cactccgaag	15	TC13667	93	0	weakly similar to SP|Q92900|RNT1_HUMAN Regulator of nonsense transcripts 1 (Nonsense mRNA reducing factor 1) (NORF1) (Up-frameshift suppressor 1 homolog). [Human] {Homo sapiens}, partial (3%)
694	agtacaaaag	15	TC7048	83	0	weakly similar to GP|27497118|gb|AAO17319.1|AF465616_1|AF465616 death inducer with SAP domain DIS {Homo sapiens}, partial (6%)
694	agtacaaaag	15	CD086078	9	3	

694	agtacaaaag	15	CD202760	51	0	homologue to GP|1129142|emb|CAA55482.1||X78884 ORF1 {Hordeum vulgare}, partial (13%)
695	gtattagcag	15	TC13821	60	1	weakly similar to GP|13561418|gb|AAK30301.1|AF289866_1|AF289866 haploid germ cell-specific nuclear protein kinase {Mus musculus}, partial (10%)
696	ttgttacctt	15				no_annot
697	agagatagat	15	TC7036	64	1	similar to SP|Q12224|RLM1_YEAST Transcription factor RLM1. [Baker's yeast] {Saccharomyces cerevisiae}, partial (3%)
697	agagatagat	15	TC7083	60	0	weakly similar to SP|P80316|TCPE_MOUSE T-complex protein 1, epsilon subunit (TCP-1-epsilon) (CCT-epsilon). [Mouse] {Mus musculus}, partial (9%)
697	agagatagat	15	CD155190	18	0	
698	catttagaat	15	TC17989	95	0	similar to GP|14517464|gb|AAK62622.1||AY039567 At1g65820/F1E22_4 {Arabidopsis thaliana}, partial (10%)
699	tatgctaata	15				no_annot
700	taacagtggt	15	CD163887	61	0	
701	tgacaaattt	15	TC7847	88	0	
702	tgataatgtt	15	C209506.1	95	1	
703	gatttcctgc	15				no_annot
704	gaaactcaac	15	TC17063	84	0	weakly similar to GP|22859164|emb|CAD30680.1||AJ459409 RNA-binding protein S1 {Drosophila melanogaster}, partial (8%)
705	tgatattcga	15	TC13717	90	0	weakly similar to SP|Q9UK97|FBX9_HUMAN F-box only protein 9. [Human] {Homo sapiens}, partial (16%)
705	tgatattcga	15	TC18677	38	0	weakly similar to GP|2102722|gb|AAB63357.1||U90750 aspartic protease precursor {Schistosoma japonicum}, partial (28%)
706	acgctaaatt	14	TC16561	60	2	
707	cggataaagg	14	C301044.1	13	0	
707	cggataaagg	14	C709088.1	12	0	
708	acaagtactt	14				no_annot
709	aggggaagcg	14	TC11581	21	0	similar to GP|15128111|gb|AAK84395.1|AF397147_1|AF397147 G10-like protein {Branchiostoma belcheri}, partial (98%)

710	ataataatcg	14	TC10865	96	0	weakly similar to GP|18447221|gb|AAL68201.1||AY075335 GH14278p {Drosophila melanogaster}, partial (23%)
711	tcagtaactg	14	TC17858	83	0	
712	gctaaacgca	14	TC10681	60	0	weakly similar to GP|17945700|gb|AAL48899.1||AY071277 RE30690p {Drosophila melanogaster}, partial (86%)
712	gctaaacgca	14	TC10682	49	1	weakly similar to GP|17945700|gb|AAL48899.1||AY071277 RE30690p {Drosophila melanogaster}, partial (83%)
713	ttgtagctac	14				no_annot
714	acaaaaaaaa	14				no_annot
715	gttaaggaga	14				no_annot
716	gtgaattgat	14	TC17742	93	0	
717	tgtacttcgt	14	TC13771	97	0	similar to SP|P35790|KICH_HUMAN Choline kinase(CK) (CHETK-alpha). [Human] {Homo sapiens}, partial (4%)
718	tactgctact	14	TC17283	4	7	similar to GP|8809745|gb|AAF79928.1|AF191320_1|AF191320 secretory lipase 7 {Candida albicans}, partial (5%)
719	ctatgcaaat	14	TC13862	78	0	weakly similar to SP|P08579|RU2B_HUMAN U2 small nuclear ribonucleoprotein B". [Human] {Homo sapiens}, partial (39%)
720	ttcaatgttt	14	TC16804	86	0	
720	ttcaatgttt	14	AI977390	42	2	weakly similar to EGAD|64198|67096 ribosomal protein L6 {Rattus norvegicus}, partial (20%)
721	tgcataaaga	14				no_annot
722	gaaacaagtg	14	TC14219	41	2	similar to GP|17861498|gb|AAL39226.1||AY069081 GH09638p {Drosophila melanogaster}, partial (34%)
723	gttcttattc	14	TC6881	88	0	similar to GP|12654267|gb|AAH00954.1|AAH00954|BC000954 heterochromatin-like protein 1 {Homo sapiens}, partial (61%)
724	gacaggttcc	14				no_annot
725	tacacagatg	14				no_annot
726	ttcagatgcg	14	TC17167	68	0	homologue to GP|11139544|gb|AAG31750.1|AF275260_1|AF275260 SRPSOX {Homo sapiens}, partial (5%)

727	gcgttcaaat	14	TC10877	89	2	similar to GP|16740615|gb|AAH16192.1||BC016192 Bicd1 protein {Mus musculus}, partial (15%)
728	gattaaaaaa	14				no_annot
729	gctttgacag	14	TC13714	55	0	weakly similar to GP|29897658|gb|AAP10933.1||AE017011 Neopullulanase {Bacillus cereus ATCC 14579}, partial (5%)
730	attccagcca	14	TC7545	54	2	weakly similar to GP|687602|gb|AAA85685.1||U17331 ribosomal protein L9, mutant {Mus musculus}, partial (72%)
731	gcctcgttga	14	TC12185	87	0	
732	atagaagtag	14				no_annot
733	aacgaccatt	14				no_annot
734	gatcgatcaa	14				no_annot
735	gaattatcct	14				no_annot
736	ggttgcggct	14	TC14237	82	0	
737	ctgaagctag	14	AF130788_1003_2262	45	3	[CDS] NADH dehydrogenase 4 (ND4) gene, complete cds
738	tttacgaagc	14	C611925.1	89	0	
739	agatttaggt	14	TC11148	92	0	weakly similar to GP|7415938|dbj|BAA93617.1||AB026688 asc1 {Mus musculus}, partial (10%)
740	taattctcat	14				no_annot
741	catttccatt	14	H49027	45	0	
742	gtgtgtaaaa	14	C210103.1	93	0	
743	gtcataacta	14	TC11040	90	0	
744	atcgtgagtg	14				no_annot
745	gtaacaaaaa	14	TC17489	88	0	weakly similar to PIR|B38128|B38128 epithelin/granulin precursor - rat, partial (13%)
746	aaggttgttg	14				no_annot
747	gttcaacagt	14	TC10533	97	0	weakly similar to GP|1914794|emb|CAA72813.1||Y12106 aminopeptidase {Lumbricus rubellus}, partial (76%)
748	tgaaaaaata	14	TC16680	69	2	similar to GP|23664248|gb|AAN39278.1|AF519182_1|AF519182 eggshell precursor protein {Schistosoma japonicum}, partial (67%)

749	atacttggtt	14	gi|4138864|gb|AF101196.1|AF101196	73	2	Schistosoma mansoni cytochrome c oxidase subunit 1 (cox1) mRNA, mitochondrial gene encoding mitochondrial protein, complete cds
750	cagatgagtg	14	TC8361	75	0	weakly similar to GP|1151093|gb|AAA89164.1||U39045 cytoplasmic dynein intermediate chain 2B {Rattus norvegicus}, partial (27%)
751	cgctgcgatg	14	TC13950	22	0	
752	gaacgaagta	14	AY149571|Sma.1207	69	1	LOK-like protein kinase
753	ggattgtcct	14	TC10730	71	1	weakly similar to GP|7303306|gb|AAF58366.1||AE003818 CG6050-PA {Drosophila melanogaster}, partial (63%)
754	gaaaaagaaa	14	TC7178	44	2	
755	aaagctgttg	14	TC17867	70	0	weakly similar to SP|Q9PW72|RIL_CHICK LIM protein RIL (Reversion-induced LIM protein). [Chicken] {Gallus gallus}, partial (20%)
756	atagaggctg	14	C202428.1	31	0	
756	atagaggctg	14	C204341.1	70	0	
757	gaatgacaaa	14	TC17440	82	3	
758	gtttttaag	14	C601498.1	92	0	
759	atccgagcga	14	C201498.1	22	1	
759	atccgagcga	14	C703360.1	59	0	
760	gagatcgaac	14				no_annot
761	ctcaattgcc	14	TC10904	60	0	weakly similar to GP|2274922|emb|CAA73153.1||Y12573 exon 6' {Drosophila melanogaster}, partial (69%)
762	ggatgacaga	14	TC8784	96	0	
763	attagttgtg	14	TC13937	98	0	weakly similar to GP|16197825|gb|AAL13552.1||AY058323 GH09295p {Drosophila melanogaster}, partial (21%)
764	tacaggacca	14	TC17364	79	0	similar to GP|17946096|gb|AAL49090.1||AY071468 RE54691p {Drosophila melanogaster}, partial (25%)
765	caccattacg	14	TC15256	43	0	
766	gacatcgcgt	14	TC13988	83	0	weakly similar to GP|17945259|gb|AAL48687.1||AY071065 RE14259p {Drosophila melanogaster}, partial (66%)
767	cagtgaagat	14				no_annot
768	gcagattccg	14	C612384.1	77	0	

769	cctattcggg	14				no_annot
770	cttgattgtg	14	TC14338	82	0	similar to SP|Q99471|PFD5_HUMAN Prefoldin subunit 5 (C-myc binding protein Mm-1) (Myc modulator 1). [Human] {Homo sapiens}, partial (21%)
771	tagagctgat	14				no_annot
772	ttcattgttt	14	TC11236	27	1	
773	tacacaaaaa	14	C711805.1	25	1	
774	aatagcttta	14				no_annot
775	tgtatccaat	13				no_annot
776	cgacgtttct	13	BF936070	87	0	
777	aatgtacaaa	13				no_annot
778	gaaggaacta	13				no_annot
779	gtttatcgat	13	TC12212	84	0	similar to PIR|JC7719|JC7719 CPI-17alpha - human, partial (10%)
779	gtttatcgat	13	CD083300	68	1	
780	tgcgacctaa	13	DQ086815|Sma.1057	27	0	Small nuclear ribonucleoprotein SM-D3
781	cggaatcgtc	13	TC8428	33	1	homologue to GP|2688944|gb|AAB88884.1||AF025664 Na-Ca+K exchanger {Bos taurus}, partial (4%)
782	cggctatgtg	13				no_annot
783	gatcgaagac	13	TC10667	39	0	weakly similar to GP|27728696|gb|AAO18670.1||AY175374 dolichyl-diphosphooligosaccharide--protein glycotransferase {Branchiostoma belcheri tsingtaunese}, partial (73%)
784	caagtctgtg	13				no_annot
785	gttttgctcc	13	C611490.1	70	2	
786	gccggaacgc	13	TC7472	58	2	weakly similar to GP|3891849|pdb|1QCR|B Chain B, Crystal Structure Of Bovine Mitochondrial Cytochrome Bc1 Complex, Alpha Carbon Atoms Only, partial (6%)
787	cgattctgtc	13	TC11800	80	0	
788	atgggtgcta	13	TC14899	40	0	
789	ttttacaaac	13				no_annot
790	agattcgagg	13				no_annot

791	aaacaaaaaa	13	TC8763	91	0	weakly similar to GP|19071882|dbj|BAB85678.1||AB058634 small integral membrane protein of lysosome/late endosome {Gallus gallus}, partial (33%)
792	aatattattt	13	TC8846	84	1	
792	aatattattt	13	TC17057	97	0	
793	aatttgattc	13	M34453|Sma.802	39	0	23 kDa integral membrane protein (Sm23)
794	gtgcagtcag	13				no_annot
795	atcattgata	13				no_annot
796	atgctgatga	13	CD179067	26	2	
796	atgctgatga	13	CD090447	76	0	
797	tagatacagt	13				no_annot
798	aattttgtgt	13				no_annot
799	atgcaggagg	13				no_annot
800	caggttgtgg	13				no_annot
801	tccgagacca	13	AI820476	38	2	similar to GP|28894857|gb|AAK26170.1||AY028436 interleukin-4-inducing protein precursor {Schistosoma mansoni}, partial (81%)
802	aatcactatc	13	TC12739	86	0	GP|6573266|gb|AAF17611.1||AF176706 F-box protein FBX11 {Homo sapiens}, partial (6%)
803	tacataccta	13				no_annot
804	atgagtcgaa	13	TC9133	10	1	
805	gctactgctt	13	TC14534	56	2	similar to GP|3293344|gb|AAC25709.1||AF059614 transportin; TRN {Xenopus laevis}, partial (37%)
806	ccttgtgata	13	TC17241	97	0	weakly similar to GP|10048296|gb|AAG12342.1||AF294845 glycerol-3-phosphate dehydrogenase {Mus musculus}, partial (54%)
807	atttcacctt	13	TC12812	61	5	
808	gagaaatgat	13				no_annot
809	tgtgtgtatt	13	TC7315	93	0	
809	tgtgtgtatt	13	TC10910	78	0	
809	tgtgtgtatt	13	TC12841	4	2	
810	tctgctccca	13	AI976884	74	0	

810	tctgctccca	13	CD170905	13	1	
811	tagtgttgtc	13				no_annot
812	agggggaatc	13	TC7489	69	0	similar to SP|Q07955|SFR1_HUMAN Splicing factor, arginine/serine-rich 1 (pre-mRNA splicing factor SF2, P33 subunit) (Alternative splicing factor ASF-1). [Human] {Homo sapiens}, partial (69%)
812	agggggaatc	13	TC7490	24	2	similar to SP|Q13242|SFR9_HUMAN Splicing factor, arginine/serine-rich 9 (Pre-mRNA splicing factor SRp30C). [Human] {Homo sapiens}, partial (36%)
813	taacctgcta	13				no_annot
814	tgcttaatag	13	AF254148|Sma.785	86	1	PUR-alpha-like protein (PUR-alpha)
815	gatactttga	13	TC15632	75	0	weakly similar to GP|5420387|emb|CAB46679.1||AJ243459 proteophosphoglycan {Leishmania major}, partial (12%)
816	attcatctat	13	TC7388	88	2	GP|1778026|gb|AAB63442.1||U60995 aspartic proteinase {Schistosoma mansoni}, complete
817	attctggtgt	13	TC13475	93	0	similar to GP|1469904|gb|AAB49033.1||U13986 JF-2 {Schistosoma japonicum}, partial (68%)
818	ctaggtgact	13				no_annot
819	tatttggtac	13				no_annot
820	gagaataaat	13	TC14779	97	0	similar to GP|20152021|gb|AAM11370.1||AY095042 LD29234p {Drosophila melanogaster}, partial (18%)
821	tcgacgcaaa	13				no_annot
822	gcctacactt	13	TC14190	85	0	similar to GP|17945014|gb|AAL48569.1||AY070947 RE04143p {Drosophila melanogaster}, partial (13%)
823	agatgcctga	13	TC17079	43	0	
824	ttggtggtta	13				no_annot
825	taatcgtcaa	13	TC10650	42	0	weakly similar to GP|19071882|dbj|BAB85678.1||AB058634 small integral membrane protein of lysosome/late endosome {Gallus gallus}, partial (36%)
826	gatagcaagg	13	M29837|Sma.55	21	4	Tegument antigen (I(H)A)
827	ttgttggttg	13	TC8847	59	2	similar to GP|1019439|gb|AAC46908.1||U32449 mucin-like protein {Trypanosoma cruzi}, partial (8%)
828	taaaagaaca	13	TC17087	5	5	similar to GP|15408877|dbj|BAB64268.1||AP004127 P0005H10.5 {Oryza sativa (japonica cultivar-group)}, partial (9%)

829	tacaaagctt	13	AI018948	15	0	
830	gatttattgc	13	TC9126	82	0	weakly similar to GP|8132878|gb|AAF73428.1|AF245664_1|AF245664 histone deacetylase 8; HDAC8 {Homo sapiens}, partial (7%)
830	gatttattgc	13	TC10871	96	0	weakly similar to GP|8132878|gb|AAF73428.1|AF245664_1|AF245664 histone deacetylase 8; HDAC8 {Homo sapiens}, partial (30%)
831	tggaacatct	13				no_annot
832	gtaacgcgta	13				no_annot
833	gtcttggctg	13				no_annot
834	gaaggaagcc	13	TC7370	47	0	weakly similar to GP|24659630|gb|AAH39185.1||BC039185 Tcerg1 protein {Mus musculus}, partial (10%)
835	tttatgtacc	13	TC10549	96	0	
836	tgtaacaata	13				no_annot
837	cggaatttcg	13	CD139204	95	0	weakly similar to GP|16769504|gb|AAL28971.1||AY061423 LD35644p {Drosophila melanogaster}, partial (34%)
838	ctgtagtcat	13	AF030965|Sma.713	87	0	Clone A26 unknown
839	gctatgtatc	13	TC7812	80	1	weakly similar to SP|O12947|ICMT_XENLA Protein-S isoprenylcysteine O-methyltransferase(Isoprenylcysteine carboxylmethyltransferase) (Prenylcysteine carboxyl methyltransferase) (pcCMT) (Prenylated protein carboxyl methyltransferase) (PPMT) (Farnesyl cysteine carboxyl methyltransferase) (FCMT). [African clawed frog] {Xenopus laevis}, partial (39%)
840	attttcttgt	13	CD146478	83	0	
841	ctctccatat	13	TC7333	72	2	similar to GP|3309068|gb|AAC26004.1||AF072935 small GTP-binding protein rab5 {Rattus norvegicus}, partial (73%)
842	ctatatgggg	13	TC9178	86	0	weakly similar to PIR|A46498|A46498 glucocorticoid-sensitive T cell-specific protein 30 - mouse, partial (5%)
843	tgttgaactc	13	TC7725	77	0	
844	cttctccgcc	13	CD161591	42	0	homologue to SP|P08418|HS70_SCHMA Heat shock 70 kDa homolog protein (HSP70) (Major surface antigen). [Blood fluke] {Schistosoma mansoni}, partial (18%)

845	ctcgtcagta	13	TC8181	92	0	weakly similar to EGAD|56711|A-234F9.2 eIF-3 p110 subunit gen {Homo sapiens}, partial (19%)
846	ggcacctgat	13	TC7510	67	1	similar to GP|2245671|gb|AAB62569.1||AF006516 e3B1 {Homo sapiens}, partial (16%)
847	tacctcagta	13				no_annot
848	ttgaacgtga	12	TC7866	73	0	weakly similar to SP|Q94516|ATPF_DROME ATP synthase B chain, mitochondrial precursor(FO-ATP synthase subunit B). [Fruit fly] {Drosophila melanogaster}, partial (25%)
848	ttgaacgtga	12	TC8630	9	9	weakly similar to GP|6980000|gb|AAF34689.1|AF222864_1|AF222864 Dspt5 {Drosophila melanogaster}, partial (4%)
849	gtgttgttcc	12				no_annot
850	caattgggtg	12	TC16825	69	0	similar to SP|P26368|U2AF_HUMAN Splicing factor U2AF 65 kDa subunit (U2 auxiliary factor 65 kDa subunit) (U2 snRNP auxiliary factor large subunit) (hU2AF(65)). [Human] {Homo sapiens}, partial (76%)
851	taccaatacc	12	TC13830	35	0	
851	taccaatacc	12	TC13831	54	0	
852	ttcagagata	12	M21308|Sma.702	70	0	Asparaginyl endopeptidase (c197 Sm32 gene)
853	tgatacgttt	12	TC17326	98	0	
854	gttctcagat	12	C210121.1	91	0	
855	ggtcacgtaa	12	CD073190	31	1	homologue to SP|Q15701|DYL1_HUMAN Dynein light chain 1, cytoplasmic (8 kDa dynein light chain) (DLC8) (Protein inhibitor of neuronal nitric oxide synthase) (PIN). [Rabbit] {Oryctolagus cuniculus}, partial (29%)
856	tctgcaatat	12	TC17357	95	0	weakly similar to SP|P58137|PTE1_MOUSE Peroxisomal acyl-coenzyme A thioester hydrolase 1(Peroxisomal long-chain acyl-coA thioesterase 1). [Mouse] {Mus musculus}, partial (9%)
857	cataatgacg	12				no_annot
858	gtgatctagg	12	TC13277	74	0	
859	ctgttattac	12	C610249.1	91	0	
860	cgggagcttt	12				no_annot
861	tagttttcac	12				no_annot

862	tatatggatg	12	TC11294	75	0	similar to GP|11878276|gb|AAG40877.1||AF320758 programmed cell death gene-5 protein {Drosophila melanogaster}, partial (41%)
863	ttgatttttg	12	TC10294	32	1	
863	ttgatttttg	12	TC17107	83	0	GP|160971|gb|AAA29873.1||L00992 essential myosin light chain {Schistosoma mansoni}, complete
864	gccgcttatt	12				no_annot
865	ccccgtacat	12				no_annot
866	ccaaacattt	12				no_annot
867	ttcgggacca	12	C202031.1	20	0	
867	ttcgggacca	12	C301778.1	79	0	
867	ttcgggacca	12	C305393.1	39	0	
867	ttcgggacca	12	C601188.1	25	0	
867	ttcgggacca	12	C606891.1	22	1	
867	ttcgggacca	12	C612164.1	60	0	
867	ttcgggacca	12	C612193.1	11	1	
867	ttcgggacca	12	C717788.1	39	0	
867	ttcgggacca	12	C701949.1	79	0	
868	gtcatatgaa	12	TC14703	92	0	
869	ctgttcgtat	12				no_annot
870	aaaacgtaac	12	TC11468	78	0	
871	gatgggtggt	12	TC7889	77	0	weakly similar to GP|20072621|gb|AAH27205.1||BC027205 dipeptidyl peptidase 7 {Mus musculus}, partial (33%)
872	ctagctgttc	12	TC6878	91	0	similar to SP|P48643|TCPE_HUMAN T-complex protein 1, epsilon subunit (TCP-1-epsilon) (CCT-epsilon). [Human] {Homo sapiens}, partial (94%)
872	ctagctgttc	12	AW063016	88	0	
873	cctaccacag	12				no_annot
874	ctttccatca	12				no_annot
875	tcgatgggca	12				no_annot
876	aagacaattc	12	U30264|Sma.1084	79	0	Trans-spliced mRNA, clone SL cDNA-6
877	ccgagatggt	12	TC14295	21	2	similar to GP|18447473|gb|AAL68299.1||AY075489 RE41712p {Drosophila melanogaster}, partial (28%)

878	acacgaagtt	12				no_annot
879	ctactgatcc	12	TC13671	80	0	similar to SP|P49368|TCPG_HUMAN T-complex protein 1, gamma subunit (TCP-1-gamma) (CCT-gamma). [Human] {Homo sapiens}, partial (76%)
880	tacatctttg	12	TC8489	87	0	weakly similar to GP|23574715|dbj|BAC20586.1||AB083307 mitochondrial carnitine/acylcarnitine carrier protein {Macaca fascicularis}, partial (52%)
881	aatgtataaa	12	TC13518	33	0	homologue to GP|12248342|gb|AAG13168.2||AF216698 NADH dehydrogenase subunit 1 {Schistosoma mansoni}, partial (96%)
881	aatgtataaa	12	CD080074	74	1	
882	acaaatatga	12	C606449.1	5	3	
883	ggacagagat	12	TC8207	15	0	
883	ggacagagat	12	TC8208	69	0	weakly similar to GP|12641925|gb|AAK00053.1||AF134398 actin-filament fragmenting protein {Echinococcus granulosus}, partial (31%)
884	caaaaaaaaa	12				no_annot
885	aaatcctaaa	12	TC7488	86	0	
886	ccgtcatcaa	12				no_annot
887	aattagtgat	12	TC13488	91	0	PIR|B54525|B54525 major female-specific polypeptide (frame 2) - fluke (Schistosoma mansoni) (fragment), partial (47%)
888	tttccaatcc	12	TC8101	79	0	
889	caatttaata	12	TC13886	97	0	similar to GP|1841843|gb|AAB47536.1||U86674 calponin homolog {Schistosoma mansoni}, partial (20%)
889	caatttaata	12	CD096745	43	1	
889	caatttaata	12	CD201713	16	0	
890	gcactgttgt	12	C301115.1	85	0	
891	attccttcta	12	TC17280	80	1	similar to PIR|A31589|A31589 carboxypeptidase Cprecursor - human, partial (26%)
892	aaatgcgcct	12	TC13907	97	0	
893	ggaacagggt	12	TC10670	64	2	weakly similar to SP|P51989|RO21_XENLA Heterogeneous nuclear ribonucleoprotein A2 homolog 1 (hnRNP A2(A)). [African clawed frog] {Xenopus laevis}, partial (35%)

893	ggaacagggt	12	TC10671	5	3	weakly similar to SP|P09651|ROA1_HUMAN Heterogeneous nuclear ribonucleoprotein A1 (Helix-destabilizing protein) (Single-strand binding protein) (hnRNP core protein A1). [Human] {Homo sapiens}, partial (11%)
894	cctcacgtaa	12				no_annot
895	taggtggttt	12				no_annot
896	ttcattaagt	12	TC8075	68	2	
897	gttacttaag	12				no_annot
898	cccacaagtt	12	TC8428	71	0	homologue to GP|2688944|gb|AAB88884.1||AF025664 Na-Ca+K exchanger {Bos taurus}, partial (4%)
899	ttgaacaatt	12	TC14500	75	0	
900	attaaattcc	12	TC17003	94	0	similar to SP|Q24799|MYPH_ECHGR Myophilin. {Echinococcus granulosus}, complete
901	gagtcatctt	12				no_annot
902	ctcaaacaga	12	TC7554	75	0	similar to GP|21901963|dbj|BAC05520.1||AB078412 casein kinase I {Ciona savignyi}, partial (78%)
903	tttctaataa	12	TC11327	92	0	weakly similar to SP|Q9SMI3|RS12_CYAPA 40S ribosomal protein S12. {Cyanophora paradoxa}, partial (72%)
903	tttctaataa	12	CD152356	14	0	
904	aacgaatgct	12	TC7340	62	1	This is the longest open reading frame of the sequence that starts with Met
904	aacgaatgct	12	TC7349	49	4	
904	aacgaatgct	12	R95579	54	0	
905	atcctgaagc	12	TC8413	79	0	weakly similar to SP|Q9XZ63|ARME_DROME ARMET-like protein precursor. [Fruit fly] {Drosophila melanogaster}, partial (31%)
906	atcatcaatt	12	L27100|Sma.626	72	0	Serpin (SPI) gene, 3 end
907	gaagtcatta	12	TC13961	91	0	homologue to SP|P36417|GBF_DICDI G-box binding factor (GBF). [Slime mold] {Dictyostelium discoideum}, partial (4%)
908	gtggtcgtta	12	TC7770	89	0	weakly similar to SP|Q9VHN6|RM19_DROME 60S ribosomal protein L19, mitochondrial precursor. [Fruit fly] {Drosophila melanogaster}, partial (30%)

909	ccttatgtct	12	TC14196	77	0	weakly similar to SP|O35587|TM21_MESAU Transmembrane protein Tmp21 precursor (21 kDa Transmembrane trafficking protein) (Integral membrane protein p23). [Golden hamster] {Mesocricetus auratus}, partial (39%)
910	tagtgctatc	12	TC9332	92	0	weakly similar to GP|13477139|gb|AAH05026.1|AAH05026|BC005026 sirtuin (silent mating type information regulation 2, S. cerevisiae, homolog) 6 {Homo sapiens}, partial (44%)
911	aatgagaatg	12				no_annot
912	caagcagcct	12	TC17173	59	0	
913	cctataaccc	12	C716918.1	67	0	
914	gaacgcctga	12	TC7677	42	1	similar to GP|7677478|gb|AAF67180.1|AF241222_1|AF241222 mitochondrial import protein {Drosophila melanogaster}, partial (14%)
915	tgagcagctg	12	TC7574	83	0	PIR|D40335|HSHU4 histone H4 [validated] - human, complete
916	attgtaagtt	11	TC11300	88	0	weakly similar to SP|Q94758|DYL1_SCHMA Dynein light chain. [Blood fluke] {Schistosoma mansoni}, complete
917	gtttgagaag	11	AF321922|Sma.48	81	0	Purine-nucleoside phosphorylase
918	aatgcattgg	11				no_annot
919	tactctatga	11	TC8424	95	0	
920	taaacaaaaa	11	AI975349	68	2	
921	acagtatgag	11	TC15810	38	3	
922	gtgactcgga	11	TC7336	14	5	homologue to SP|P05217|TBB2_HUMAN Tubulin beta-2 chain. [Mouse] {Mus musculus}, complete
922	gtgactcgga	11	CD144666	65	0	GP|2827988|gb|AAB99949.1||AF034219 beta tubulin {Trichuris trichiura}, partial (11%)
923	tatgtgtggt	11	AF232691|Sma.69	98	0	Tyrosine kinase 5 (Tk5)
924	ggactgcatc	11	TC10220	35	0	
924	ggactgcatc	11	TC18264	36	0	
924	ggactgcatc	11	CD117113	86	0	
924	ggactgcatc	11	AI964725	68	0	
924	ggactgcatc	11	CD087661	83	0	

924	ggactgcatc	11	CD092044	12	1	
925	tctgagggga	11	TC17164	93	0	weakly similar to SP|Q9NRD5|PIC1_HUMAN PRKCA-binding protein (Protein kinase C-alpha binding protein) (Protein interacting with C kinase 1). [Human] {Homo sapiens}, partial (60%)
926	tgataaacat	11				no_annot
927	gatgcgccta	11	TC17160	56	1	
928	caattagcat	11	TC11474	64	1	weakly similar to SP|P11586|C1TC_HUMAN C-1-tetrahydrofolate synthase, cytoplasmic (C1-THF synthase) [Includes: Methylenetetrahydrofolate dehydrogenase; Methenyltetrahydrofolate cyclohydrolase; Formyltetrahydrofolate synthetase]. [Human] {Homo sapiens}, partial (8%)
929	aactgtacag	11				no_annot
930	ttggttcgtt	11	CD083386	77	0	weakly similar to GP|24485|emb|CAA37078.1||X52897 A1S9 protein (AA 1-803) {Homo sapiens}, partial (6%)
931	taacttatat	11	AY158212|Sma.859	94	0	Rho GTPase (Rho1)
932	cagccatacg	11				no_annot
933	tgtatacacc	11	TC7844	90	0	weakly similar to SP|O60749|SNX2_HUMAN Sorting nexin 2. [Human] {Homo sapiens}, partial (17%)
934	tcgtttagaa	11	TC19140	62	0	
935	ttctttactg	11	TC16261	80	0	
936	ttaagacaaa	11				no_annot
937	tcatccagta	11	TC7728	90	0	weakly similar to GP|12805127|gb|AAH02021.1||BC002021 Scamp3 protein {Mus musculus}, partial (31%)
938	attcattcaa	11	C609535.1	92	0	
939	gaagaacata	11				no_annot
940	aaaatcctgc	11	TC14421	88	0	similar to SP|P46969|RPE_YEAST Ribulose-phosphate 3-epimerase(Pentose-5-phosphate 3- epimerase) (PPE) (RPE). [Baker's yeast] {Saccharomyces cerevisiae}, partial (30%)
941	catcagtgat	11	TC11906	0	2	
942	ggttcacaca	11	TC14195	20	0	similar to GP|24266968|gb|AAN52382.1||AF548327 ribosomal protein L39 {Branchiostoma belcheri}, partial (92%)

943	tgtcaaataa	11	TC17161	93	0	similar to GP|21618189|gb|AAM67239.1||AY088934 sin3 associated polypeptide p18 {Arabidopsis thaliana}, partial (9%)
944	ctaatccaaa	11	C201717.1	97	0	
945	ggccctcatt	11	TC17516	95	0	weakly similar to GP|18478512|gb|AAL73190.1|AF334609_1|AF334609 IMP4 {Mus musculus}, partial (49%)
946	acaattactt	11	CD112040	74	1	similar to PIR|F86214|F86214 protein T6D22.2 [imported] - Arabidopsis thaliana, partial (14%)
947	taaatggggt	11	AA514127	91	0	
948	tttctctgtt	11	CD132203	63	0	
949	caaatccaaa	11				no_annot
950	ttggaaatgg	11				no_annot
951	tactattgca	11				no_annot
952	gaacacaaca	11				no_annot
953	cgtgtacgca	11	TC10100	28	0	
954	tgcggaaaca	11	TC19147	37	0	
955	ttttatctgt	11				no_annot
956	cacaaataaa	11	gi|161118|gb|M97298.1|SCMSODCT	95	0	Schistosome mansoni sytosolic Cu/Zn superoxide dismutase (SOD) mRNA, complete cds
957	gtcaccccca	11	C300826.1	3	1	
957	gtcaccccca	11	C718991.1	3	1	
958	ttttgttcgc	11	TC11086	93	0	
959	tttctcattt	11	TC7704	81	2	similar to PIR|T11115|T11115 NADH2 dehydrogenase (ubiquinone)chain 2 - Florometra serratissima mitochondrion, partial (6%)
959	tttctcattt	11	TC19283	2	3	
959	tttctcattt	11	CD195497	52	1	
960	ttgaactttt	11	C201299.1	75	0	
960	ttgaactttt	11	C300375.1	0	0	
960	ttgaactttt	11	C603032.1	64	0	
960	ttgaactttt	11	C702952.1	81	0	
961	aacacacaca	11				no_annot

962	gtagtagtag	11	TC10905	85	1	weakly similar to GP|28190008|gb|AAO32942.1|AF303586_1|AF303586 NCOR isoform b {Homo sapiens}, partial (5%)
962	gtagtagtag	11	TC11814	63	3	similar to GP|15822892|dbj|BAB68597.1||AB039073 cathelicidin {Mus spicilegus}, partial (14%)
963	gttgtatttg	11	TC11027	96	0	similar to GP|4105476|gb|AAD02424.1||AF047005 asparagine-rich protein {Plasmodium falciparum}, partial (3%)
964	gggttagcga	11	TC7032	52	0	
964	gggttagcga	11	TC7194	56	2	
964	gggttagcga	11	TC7211	31	7	
964	gggttagcga	11	TC7395	48	3	
964	gggttagcga	11	TC13420	15	1	similar to GP|27356310|dbj|BAC53290.1||AP005964 bll8025 {Bradyrhizobium japonicum USDA 110}, partial (9%)
964	gggttagcga	11	CD061619	78	0	
964	gggttagcga	11	CD196542	54	0	
964	gggttagcga	11	CD122228	67	0	
964	gggttagcga	11	CD171401	19	0	similar to GP|27356310|dbj|BAC53290.1||AP005964 bll8025 {Bradyrhizobium japonicum USDA 110}, partial (8%)
965	atgacgatga	11	TC11027	38	3	similar to GP|4105476|gb|AAD02424.1||AF047005 asparagine-rich protein {Plasmodium falciparum}, partial (3%)
965	atgacgatga	11	CD069882	79	0	SP|Q62880|T2D7_RAT Transcription initiation factor TFIID 31 kDa subunit (TAFII-31) (TAFII-32) (TAFII32) (Neuronal cell death related gene in neuron -7) (DN-7). [Rat] {Rattus norvegicus}, partial (5%)
966	tgataacctg	11				no_annot
967	caagttaatt	11	TC13458	91	0	weakly similar to SP|Q24133|DNJ1_DROME DnaJ protein homolog 1 (DROJ1). [Fruit fly] {Drosophila melanogaster}, partial (60%)
968	acgaagatat	11	TC16775	50	0	similar to SP|P25867|UBC1_DROME Ubiquitin-conjugating enzyme E2-17 kDa(Ubiquitin-protein ligase) (Ubiquitin carrier protein) (Effete protein). [Fruit fly] {Drosophila melanogaster}, partial (95%)
968	acgaagatat	11	TC16776	42	0	similar to PIR||I59365|I59365 ubiquitin conjugating enzyme - human, partial (95%)

968	acgaagatat	11	TC16778	28	0	similar to SP|P25867|UBC1_DROME Ubiquitin-conjugating enzyme E2-17 kDa(Ubiquitin-protein ligase) (Ubiquitin carrier protein) (Effete protein). [Fruit fly] {Drosophila melanogaster}, complete
968	acgaagatat	11	TC16779	69	0	similar to SP|P25867|UBC1_DROME Ubiquitin-conjugating enzyme E2-17 kDa(Ubiquitin-protein ligase) (Ubiquitin carrier protein) (Effete protein). [Fruit fly] {Drosophila melanogaster}, partial (73%)
968	acgaagatat	11	TC16780	27	3	similar to SP|P25867|UBC1_DROME Ubiquitin-conjugating enzyme E2-17 kDa(Ubiquitin-protein ligase) (Ubiquitin carrier protein) (Effete protein). [Fruit fly] {Drosophila melanogaster}, partial (95%)
968	acgaagatat	11	CD092723	76	0	similar to SP|P25867|UBC1_DROME Ubiquitin-conjugating enzyme E2-17 kDa(Ubiquitin-protein ligase) (Ubiquitin carrier protein) (Effete protein). [Fruit fly] {Drosophila melanogaster}, partial (59%)
969	cacattttcc	11	TC12150	92	0	
970	ccaaaaatat	11	TC10655	93	2	similar to GP|230468|pdb|2CTS| Citrate Synthase (E.C.4.1.3.7) - (CoA, Citrate) Complex, partial (98%)
971	ctagcagtta	11				no_annot
972	aagaatcaca	11	TC12491	28	0	
972	aagaatcaca	11	TC16507	90	0	
973	cttgtcatcc	11	C200825.1	21	1	
973	cttgtcatcc	11	C612314.1	30	0	
974	agtcaaacgc	11	TC16847	68	0	weakly similar to EGAD|25135|3076 heterogeneous nuclear ribonucleoprotein K {Homo sapiens}, partial (19%)
975	taaatcttgt	11	TC11142	87	2	
976	tatttctgct	11	TC9267	27	3	
976	tatttctgct	11	TC10896	96	0	weakly similar to SP|O08522|GS28_CRIGR 28 kDa Golgi SNARE protein (Golgi SNAP receptor complex member 1) (28 kDa cis-Golgi SNARE p28) (GOS-28). [Chinese hamster] {Cricetulus griseus}, partial (15%)
976	tatttctgct	11	TC16762	55	0	
976	tatttctgct	11	CD066366	44	0	

976	tatttctgct	11	CD085475	33	1	
976	tatttctgct	11	CD087523	24	0	
976	tatttctgct	11	CD177910	42	0	
976	tatttctgct	11	CD124628	67	0	
977	gatatttgtg	11	TC10052	65	0	similar to GP|1905880|gb|AAC82512.1||U91483 cardiac calsequestrin {Mus musculus}, partial (5%)
978	tctccctgaa	11				no_annot
979	tgtacgtacg	11	TC17038	24	4	weakly similar to PIR|T39903|T39903 serine-rich protein - fission yeast (Schizosaccharomyces pombe), partial (5%)
980	ctatacacca	11	TC8360	94	0	weakly similar to PIR|T15494|T15494 aspartate transaminaseC14F11.1 [similarity] - Caenorhabditis elegans, partial (89%)
981	gatttcaaga	11				no_annot
982	tggatgagat	11				no_annot
983	gttgcagttt	11	TC11193	92	0	
984	ggaaatataa	11	TC10669	87	1	
985	atgaacttga	11	TC17305	72	1	weakly similar to GP|4235226|gb|AAD13138.1||AF061025 leucine zipper-EF-hand containing transmembrane protein 1 {Homo sapiens}, partial (11%)
985	atgaacttga	11	TC19167	73	0	
986	aaagtcggtg	11	TC7898	35	0	
987	cttcacaggt	11	TC7727	86	0	similar to GP|21280323|dbj|BAB96814.1||AB081951 AGO1 homologous protein {Oryza sativa (japonica cultivar-group)}, partial (15%)
988	gatttgtttg	11				no_annot
989	ggcagttgtc	11	TC13879	15	7	weakly similar to GP|7295350|gb|AAF50668.1||AE003563 CG10173-PA {Drosophila melanogaster}, partial (9%)
990	gatacgagag	11				no_annot
991	tatggatcag	11				no_annot
992	tgttggattc	11	TC17547	84	0	
992	tgttggattc	11	CD080978	50	0	
993	tgagaaagat	11	C611276.1	94	0	

994	tttgtttcag	11	TC7449	72	1	weakly similar to GP|2541910|dbj|BAA22850.1||AB008003 troponin T {Mizuhopecten yessoensis}, partial (43%)
994	tttgtttcag	11	TC13907	12	6	
994	tttgtttcag	11	AA801608	40	0	
995	ctatgcttag	11	TC10581	83	0	
996	ttatattagt	11	TC7928	88	0	
997	tatacagaat	11	TC17350	94	0	weakly similar to GP|7295114|gb|AAF50440.1||AE003555 CG7083-PA {Drosophila melanogaster}, partial (26%)
998	cctaacactg	11	TC11003	84	0	
999	gtgacctatg	11				no_annot
1000	caaataatga	11	TC13697	90	0	weakly similar to GP|15418966|gb|AAK83461.1||AY039235 annexin 4 {Xenopus laevis}, partial (63%)
1001	ttcaaaagat	11	AF521091|Sma.53	67	0	CD63-like protein Sm-TSP-2
1002	taacaatctg	11				no_annot
1003	atttatcaat	10	U30261|Sma.677	82	0	G protein beta subunit-like protein trans-spliced
1004	aatacaccat	10				no_annot
1005	caaaaggcga	10	TC10679	83	0	similar to SP|Q99832|TCPH_HUMAN T-complex protein 1, eta subunit (TCP-1-eta) (CCT-eta) (HIV-1 Nef interacting protein). [Human] {Homo sapiens}, partial (89%)
1006	gtcgtcaatc	10	C301417.1	56	0	
1006	gtcgtcaatc	10	C709201.1	56	0	
1007	atgattgctt	10	TC17068	48	0	
1008	aagcgtggta	10				no_annot
1009	tggtgctaat	10				no_annot
1010	gtaaatctca	10	TC10556	52	4	RTNL [Schistosoma mansoni]
1011	tcgacaggcc	10	TC7592	73	2	similar to GP|9957034|gb|AAG09182.1|AF175224_1|AF175224 preconditioning-inducible gene 1 protein {Rattus norvegicus}, partial (57%)
1012	atatgtatct	10	TC17017	87	0	similar to GP|17945779|gb|AAL48937.1||AY071315 RE33866p {Drosophila melanogaster}, partial (42%)

1012	atatgtatct	10	TC17018	89	0	weakly similar to GP|17945779|gb|AAL48937.1||AY071315 RE33866p {Drosophila melanogaster}, partial (23%)
1013	tcaacctaga	10				no_annot
1014	acaaaaaaag	10	TC15915	6	3	
1014	acaaaaaaag	10	N21923	77	0	
1015	ttgccaaacc	10	TC11066	69	0	
1016	gtcgctcctg	10	C712397.1	1	1	
1016	gtcgctcctg	10	C716666.1	2	1	
1017	tgcacgcgtt	10	TC7847	25	2	
1017	tgcacgcgtt	10	CD080904	11	0	
1018	accaaacttg	10	TC15276	40	1	similar to GP|15156827|gb|AAK87500.1||AE008094 AGR_C_3176p {Agrobacterium tumefaciens str. C58 (Cereon)}, partial (7%)
1019	tgttgtccat	10				no_annot
1020	ttatatgcct	10				no_annot
1021	ttggtcaacg	10				no_annot
1022	gtagtcggat	10				no_annot
1023	aacaaggaga	10	AI975354	93	0	similar to GP|21702404|gb|AAM75879.1||AF359057 NADH dehydrogenase subunit 1 {Leishmania braziliensis}, partial (8%)
1024	tacgccaatg	10	TC13053	93	0	
1025	cgaattgtga	10	TC14154	77	0	weakly similar to GP|1196428|gb|AAA88032.1||M14123 neutral protease large subunit {Homo sapiens}, partial (36%)
1026	ctttcccact	10	DQ249305|Sma.5246	89	1	CD36-like class B scavenger receptor
1027	ttggtttact	10				no_annot
1028	tttgtaaagc	10	TC13741	96	0	similar to GP|13097204|gb|AAH03368.1||BC003368 dihydrolipoamide dehydrogenase {Mus musculus}, partial (87%)
1029	tgtaagtaag	10				no_annot
1030	aggaatggaa	10				no_annot
1031	ctagggttgt	10	U30263|Sma.249	44	0	Carbonyl reductase-like protein trans-spliced
1032	ccatacaaga	10				no_annot

1033	catattacgt	10	TC14430	94	0	weakly similar to GP|21430736|gb|AAM51046.1||AY119186 SD10846p {Drosophila melanogaster}, partial (15%)
1034	tagctatctt	10				no_annot
1035	atcgcgtgtc	10				no_annot
1036	caagtttttg	10	TC11035	94	0	
1037	catttaaata	10				no_annot
1038	ccatagattg	10	TC10524	44	2	
1039	tgccgaggtc	10				no_annot
1040	ttcgaagatt	10	CD179176	53	0	
1041	taagctgggc	10				no_annot
1042	tcattgaaag	10	TC16693	78	0	
1043	tgggcataaa	10	TC15133	37	0	
1044	ggcaccgttg	10				no_annot
1045	ggtccgtgtt	10	TC14147	80	0	similar to PIR|JH0590|OKGAR1 protein kinase, cAMP-dependent, type I regulatory chain - California sea hare, partial (47%)
1046	gggtttgatt	10				no_annot
1047	tcatttgaat	10				no_annot
1048	ataagtttct	10	TC13648	89	2	homologue to GP|832859|gb|AAA67384.1||L32580 presumptive protein 3 {Sceloporus grammicus}, partial (19%)
1049	caaccatcat	10	TC14755	19	1	weakly similar to SP|P19338|NUCL_HUMAN Nucleolin (Protein C23). [Human] {Homo sapiens}, partial (4%)
1049	caaccatcat	10	TC18263	98	0	weakly similar to GP|15011827|gb|AAA68332.2||U28742 Adaptin or adaptin-related protein protein 4 {Caenorhabditis elegans}, partial (4%)
1050	gtcaatttga	10				no_annot
1051	taatacagtt	10				no_annot
1052	ttcttcgttt	10	TC7702	95	0	weakly similar to SP|P29673|APTE_DROME Apterous protein. [Fruit fly] {Drosophila melanogaster}, partial (6%)
1053	tagataaggt	10	TC14334	55	0	similar to GP|20198901|gb|AAM15614.1|AC025721_6|AC025721 Adaptin or adaptin-related protein protein 8 {Caenorhabditis elegans}, partial (85%)

1054	atgaatgaaa	10				no_annot
1055	acaattaatt	10				no_annot
1056	gtccaaaatc	10	TC11030	31	1	similar to PIR|T45729|T45729 dehydrin-like protein - Arabidopsis thaliana, partial (13%)
1056	gtccaaaatc	10	CD092653	81	0	
1057	tgcaaatatt	10	C200438.1	86	0	
1057	tgcaaatatt	10	C601986.1	86	0	
1058	agctccaaaa	10	TC14946	28	0	
1059	gagtaaaagt	10	TC18479	81	2	
1060	atgtttgtat	10				no_annot
1061	aatttcgcac	10				no_annot
1062	gtacaatcaa	10				no_annot
1063	tcagtttgta	10	C209090.1	88	0	
1063	tcagtttgta	10	C602028.1	80	0	
1064	agcaaatcca	10				no_annot
1065	gcaactacga	10				no_annot
1066	aattttcccg	10				no_annot
1067	attcaatatt	10	TC17148	89	0	
1067	attcaatatt	10	AI447049	86	0	weakly similar to PIR|C83496|C83496 glutathione-regulated potassium-efflux system protein KefB PA1207 [imported] - Pseudomonas aeruginosa (strain PAO1), partial (4%)
1068	ttgttgtaat	10	TC11683	74	0	
1069	attatatcga	10	TC17278	90	0	
1070	attcgtaatg	10	TC15077	5	2	
1071	tgactgtttt	10	M94045|Sma.100	66	0	Calcium binding protein (Sm20)
1072	tggtttcctt	10	TC10640	98	0	
1073	tacactcgga	10	C301983.1	49	0	
1074	caaaatgatt	10				no_annot
1075	tttgcctaca	10				no_annot
1076	agtgtacttg	10				no_annot
1077	tgtgtttgtc	10	TC18513	83	0	

1078	taaatacaac	10	TC17059	39	1	weakly similar to GP|14198122|gb|AAH08121.1||BC008121 AI504353 protein {Mus musculus}, partial (10%)
1079	cgccctcacc	10				no_annot
1080	atgtgtataa	10	TC13928	38	3	weakly similar to SP|Q12874|S3A3_HUMAN Splicing factor 3A subunit 3 (Spliceosome associated protein 61) (SAP 61) (SF3a60). [Human] {Homo sapiens}, partial (45%)
1081	aagtgttgag	10	TC13865	21	1	homologue to GP|23428921|gb|AAM33119.1||AY098931 NADH dehydrogenase subunit 5 {Aplysia punctata}, partial (7%)
1082	taaactgggt	10	C209556.1	86	0	
1082	taaactgggt	10	C607907.1	80	1	
1083	atgaatataa	10	TC14178	92	0	similar to GP|5138993|gb|AAD40384.1||AF100741 vacuolar H-ATPase subunit D {Homo sapiens}, partial (81%)
1084	aaaataccat	10	TC17796	94	0	
1085	gggatgaaca	10				no_annot
1086	gtggtgagta	10	TC10440	74	0	weakly similar to SP|Q99797|PMIP_HUMAN Mitochondrial intermediate peptidase, mitochondrial precursor(MIP). [Human] {Homo sapiens}, partial (14%)
1087	gtgaaaaaaa	10	CD079392	17	0	
1088	ttgtaatagt	10	AI975110	80	0	
1089	tttaatcttt	10	TC7884	94	0	weakly similar to GP|11121262|emb|CAC14790.1||AJ291614 actin related protein 2/3 protein complex subunit p16 {Lumbricus rubellus}, partial (35%)
1090	agtatgttaa	10	TC7811	94	0	similar to SP|P23514|COPB_RAT Coatomer beta subunit (Beta-coat protein) (Beta-COP). [Rat] {Rattus norvegicus}, partial (20%)
1091	tataatcgat	10				no_annot
1092	cgttacgatg	10	CD133474	64	0	
1093	tgagggcttg	10	TC6894	53	0	
1093	tgagggcttg	10	TC6917	90	0	
1093	tgagggcttg	10	TC7150	12	2	
1093	tgagggcttg	10	TC7174	75	0	
1093	tgagggcttg	10	TC7267	58	1	

1093	tgagggcttg	10	TC12329	81	0	
1093	tgagggcttg	10	TC12345	32	1	
1093	tgagggcttg	10	TC14982	43	0	
1093	tgagggcttg	10	AI974978	13	0	
1093	tgagggcttg	10	CD066571	1	0	
1093	tgagggcttg	10	CD166865	29	2	
1094	atgataacgg	10	TC7050	24	4	similar to GP|13171103|gb|AAK13589.1||AF139989 rRNA intron-encoded homing endonuclease {Oryza sativa}, partial (52%)
1094	atgataacgg	10	TC13496	78	0	
1094	atgataacgg	10	CD088145	71	0	
1095	aaacccgact	10				no_annot
1096	atactcgcga	10	TC6888	88	1	similar to GP|16648356|gb|AAL25443.1||AY060404 LD32873p {Drosophila melanogaster}, partial (30%)
1097	ggacctaccc	10				no_annot
1098	aaaaatgatc	10	TC10551	81	2	homologue to GP|1314718|gb|AAA99798.1||U54586 Pro-His-rich protein {Schistosoma mansoni}, complete
1099	tggactgatc	10	TC17097	78	0	weakly similar to PIR|S66288|S66288 nuclear pore-targeting complex protein, 97K - mouse, partial (51%)
1100	tatgtatgtg	10	TC11677	72	1	
1100	tatgtatgtg	10	TC15469	70	0	weakly similar to GP|15292301|gb|AAK93419.1||AY051995 LD46238p {Drosophila melanogaster}, partial (18%)
1100	tatgtatgtg	10	CD167105	30	1	
1101	acgaaactta	10	TC10362	63	0	homologue to GP|2829289|gb|AAC00519.1||AF044412 HSP70 {Schistosoma japonicum}, complete
1101	acgaaactta	10	CD138878	10	1	homologue to GP|2829289|gb|AAC00519.1||AF044412 HSP70 {Schistosoma japonicum}, partial (15%)
1101	acgaaactta	10	CD138878	10	0	homologue to GP|2829289|gb|AAC00519.1||AF044412 HSP70 {Schistosoma japonicum}, partial (15%)
1102	tacagtgatt	10				no_annot
1103	tatagaagcc	10	TC10892	89	0	weakly similar to SP|O94903|POSC_HUMAN Proline synthetase co-transcribed bacterial homolog protein. [Human] {Homo sapiens}, partial (40%)
1104	ttagtgtacc	10				no_annot

1105	tcgaaaccag	10	TC17555	89	0	similar to GP|21103955|gb|AAK39642.1||AY032599 multidrug resistance-associated protein 7 {Homo sapiens}, partial (5%)
1106	tcgctgaact	10				no_annot
1107	agtcacgaat	10	TC11151	96	0	similar to GP|7303935|gb|AAF58979.1||AE003834 CG8057-PA {Drosophila melanogaster}, partial (26%)
1108	tgtacagacg	10	TC17653	68	1	
1109	aactaaatga	9				no_annot
1110	tagagagaag	9	TC10924	79	0	
1111	ctttttggtt	9	AI026208	26	1	
1112	caaaaagaaa	9	C206218.1	72	0	
1112	caaaaagaaa	9	C612159.1	48	3	
1113	ggtacaactc	9	TC8012	35	0	
1114	tacattgtac	9	TC14763	68	0	
1115	tgcgaaacat	9				no_annot
1116	ctttgagtca	9	TC9074	89	0	weakly similar to SP|P34529|DCR1_CAEEL Endoribonuclease dcr-1. {Caenorhabditis elegans}, partial (4%)
1117	tatatctgcc	9	TC13639	79	0	similar to GP|15487549|gb|AAB52440.3||U97193 Tropomodulin protein 1, isoform a {Caenorhabditis elegans}, partial (21%)
1118	aagaaatgca	9	AF101194|Sma.86	33	17	Receptor tyrosine kinase
1119	gccatcctag	9	TC10726	77	0	similar to SP|P51893|SAH1_XENLA Adenosylhomocysteinase 1(S-adenosyl-L-homocysteine hydrolase 1) (ADOHCYASE 1). [African clawed frog] {Xenopus laevis}, partial (98%)
1120	ctttttcgaa	9	TC7706	95	0	weakly similar to SP|P87498|VIT1_CHICK Vitellogenin I precursor (Minor vitellogenin) [Contains: Lipovitellin I (LVI); Phosvitin (PV); Lipovitellin II (LVII); YGP42]. [Chicken] {Gallus gallus}, partial (3%)
1121	gtctcaggtt	9				no_annot
1122	gcatatgtaa	9				no_annot
1123	gtttaatcca	9	TC17578	48	7	weakly similar to GP|11643561|gb|AAG39628.1|AF317058_1|AF317058 porcupine isoform D {Homo sapiens}, partial (10%)
1123	gtttaatcca	9	CD116813	62	0	

1124	acccacaccg	9				no_annot
1125	atgtttttgc	9				no_annot
1126	tgtctgttta	9	TC10907	41	0	weakly similar to SP|Q94743|SORC_SCHJA Sorcin. [Blood fluke] {Schistosoma japonicum}, complete
1127	tgattgcgtg	9	TC17295	81	1	
1128	accgtcgtcg	9	C603285.1	61	0	
1129	tgtgtgtgcg	9				no_annot
1130	ttctggatga	9	TC19172	74	3	
1131	ataatgatgg	9	TC16685	75	2	weakly similar to SP|P56101|CSP_TORCA Cysteine string protein (CCCS1). [Pacific electric ray] {Torpedo californica}, partial (31%)
1132	ataatgatgc	9	TC7916	85	0	weakly similar to GP|1857237|gb|AAB49973.1||U73824 p97 {Homo sapiens}, partial (7%)
1133	gactggaatc	9				no_annot
1134	tgtcgggttt	9				no_annot
1135	gctcgagcag	9	AI977037	83	0	weakly similar to GP|8895101|gb|AAF80763.1|AF164799_1|AF164799 putatative 28 kDa protein {Homo sapiens}, partial (35%)
1136	aaattcgatc	9	TC8500	68	2	
1137	tatacctaaa	9	AI976259	85	0	homologue to GP|12248331|gb|AAG13157.2||AF216698 cytochrome c oxidase subunit 1 {Schistosoma mansoni}, partial (19%)
1138	tgatatctgt	9	CD167413	39	0	
1139	tacatacgag	9				no_annot
1140	aacactgatt	9				no_annot
1141	tttgttgcta	9	TC16694	12	0	similar to SP|P01123|YPT1_YEAST GTP-binding protein YPT1 (Protein YP2). [Baker's yeast] {Saccharomyces cerevisiae}, partial (83%)
1142	aatgcatatg	9	TC14417	97	0	similar to SP|P51966|UBC7_HUMAN Ubiquitin-conjugating enzyme E2-18 kDa UbcH7(Ubiquitin- protein ligase) (Ubiquitin carrier protein) (UbcM4) (E2-F1) (L-UBC). [Mouse] {Mus musculus}, partial (84%)
1143	ctgtggcgtc	9				no_annot
1144	ttaatgaagt	9	TC13578	95	0	weakly similar to GP|7303252|gb|AAF58314.1||AE003816 CG6671-PA {Drosophila melanogaster}, partial (16%)

1145	agcattcttt	9	TC17522	94	0	weakly similar to SP|Q9I047|TAL_PSEAE Transaldolase. {Pseudomonas aeruginosa}, partial (73%)
1146	tatccagcaa	9	AA999471	40	0	
1147	gaattgtgaa	9	C202098.1	36	3	
1147	gaattgtgaa	9	C301867.1	51	0	
1147	gaattgtgaa	9	C605658.1	7	6	
1147	gaattgtgaa	9	C701686.1	51	0	
1148	cgcaatggaa	9				no_annot
1149	taagaagtgg	9				no_annot
1150	tccttactgt	9	AY323529|Sma.76	87	0	ATP-diphosphohydrolase 1 (ATPDase1)
1151	gccaaatcta	9	TC17145	83	0	weakly similar to GP|28628851|gb|AAO49385.1|AF484940_1|AF484940 glutathione S-transferase omega {Schistosoma mansoni}, partial (15%)
1152	tgtgaatgga	9	TC17799	4	0	
1153	ttcaaaaata	9	AF316827|Sma.610	95	0	Zinc finger protein SmZF1
1154	acgtttcagc	9				no_annot
1155	ctttgcttac	9				no_annot
1156	taattcttaa	9	TC14512	83	0	weakly similar to GP|21430068|gb|AAM50712.1||AY118852 GM16138p {Drosophila melanogaster}, partial (43%)
1157	gcccgatagt	9	TC15005	74	0	similar to GP|20373025|dbj|BAB91181.1||AB073746 3-ketoacyl-acyl carrier protein synthase {Arabidopsis thaliana}, partial (8%)
1158	tcgtcaattc	9				no_annot
1159	gactggcttt	9	TC8038	68	0	weakly similar to SP|Q9NZ01|GSN2_HUMAN Synaptic glycoprotein SC2. [Human] {Homo sapiens}, partial (45%)
1160	taaacaccat	9				no_annot
1161	ttaagctctg	9	TC10751	77	3	weakly similar to GP|4490377|emb|CAB38634.1||AJ133649 actin related complex p41 subunit {Drosophila melanogaster}, partial (34%)
1162	tgcatcgaag	9				no_annot
1163	tatcagtttg	9				no_annot
1164	agggacagta	9	TC11109	89	0	

1165	tgttgttgtt	9	TC17146	77	1	similar to GP|16226045|gb|AAL16062.1|AF420278_1|AF420278 carbonyl reductase {Anguilla japonica}, partial (16%)
1166	tgatggattt	9				no_annot
1167	aaacaagtat	9	TC15071	13	5	weakly similar to GP|5305403|gb|AAD41634.1|AF072372_1|AF072372 lysosomal trafficking regulator 2 {Mus musculus}, partial (4%)
1168	cgtactcttg	9	TC14883	89	0	
1169	tatgtgaatg	9				no_annot
1170	taacgaaaac	9	TC8451	93	0	weakly similar to GP|3043760|dbj|BAA25544.1||AB012759 rPOP {Rattus norvegicus}, partial (27%)
1171	agttttgttg	9				no_annot
1172	accatttact	9				no_annot
1173	ttggtgtcga	9	TC11033	56	0	weakly similar to GP|14245742|dbj|BAB56162.1||AB062451 uridine-cytidine kinase 2 {Homo sapiens}, partial (33%)
1173	ttggtgtcga	9	TC14246	75	0	weakly similar to GP|14091952|gb|AAG35185.2|AF239045_1|AF239045 KIDINS220 {Rattus norvegicus}, partial (3%)
1174	ggtggtggac	9	C606463.1	40	0	
1175	ttctatttca	9				no_annot
1176	tatacataca	9	TC13484	43	5	
1176	tatacataca	9	TC13484	43	1	
1176	tatacataca	9	TC16626	76	0	SP|P53471|ACT2_SCHMA Actin 2. [Blood fluke] {Schistosoma mansoni}, partial (27%)
1177	tttaatactt	9	TC11691	60	0	
1178	tgtacaaatc	9				no_annot
1179	acacaaacgc	9				no_annot
1180	ggaagatcaa	9				no_annot
1181	gttcactttg	9	TC13953	83	1	similar to GP|16197981|gb|AAL13761.1||AY058532 LD23816p {Drosophila melanogaster}, partial (44%)
1182	gacatctagg	9				no_annot
1183	acacctatgg	9	C202177.1	14	5	
1183	acacctatgg	9	C606054.1	5	2	

1184	aacagcacca	9				no_annot
1185	taaattagtg	9	TC14415	95	0	similar to SP|P32583|SR40_YEAST Suppressor protein SRP40. [Baker's yeast] {Saccharomyces cerevisiae}, partial (7%)
1186	cataacactg	9				no_annot
1187	ctctaggagc	9				no_annot
1188	ttggatcgaa	9	TC18020	66	0	weakly similar to GP|6648543|gb|AAF21219.1|U62056_1|U62056 TyrA {Vibrio cholerae}, partial (18%)
1189	atagtttccc	9	TC10881	93	0	weakly similar to GP|7300794|gb|AAF55938.1||AE003736 CG6690-PA {Drosophila melanogaster}, partial (12%)
1190	tgtataactt	9	TC16810	93	0	weakly similar to GP|23194245|gb|AAN15068.1||AF533661 ribophorin I {Danio rerio}, partial (36%)
1191	gattcatcta	9	TC14696	80	0	
1192	tcatcctcct	9				no_annot
1193	gaggtcataa	9	TC13742	85	0	similar to GP|1255188|gb|AAC50423.1||U50733 dynamitin {Homo sapiens}, partial (7%)
1194	aatatcggcg	9				no_annot
1195	tactgccact	9				no_annot
1196	aaaccatcct	9	TC7716	86	0	similar to SP|Q9Z2U1|PSA5_MOUSE Proteasome subunit alpha type 5(Proteasome zeta chain) (Macropain zeta chain) (Multicatalytic endopeptidase complex zeta chain). [Mouse] {Mus musculus}, partial (97%)
1197	gggcaagtgg	9				no_annot
1198	tgcgaacaaa	9				no_annot
1199	gaaacaaaaa	9	TC17886	69	0	similar to GP|21303415|gb|AAK67647.1||AY037164 vacuolar proton-ATPase subunit {Homo sapiens}, partial (57%)
1199	gaaacaaaaa	9	CD127265	32	0	similar to SP|Q9UG63|ABF2_HUMAN ATP-binding cassette, sub-family F, member 2 (Iron inhibited ABC transporter 2) (HUSSY-18). [Human] {Homo sapiens}, partial (10%)
1199	gaaacaaaaa	9	CD196878	82	0	
1200	tacatcactc	9	TC11968	95	0	
1201	taacaatgtt	9				no_annot

1202	attactgatt	9	CD194667	70	1	
1203	agttgtatct	9				no_annot
1204	cgtcggtgga	9	TC16098	24	1	
1205	tgtgaaggtg	9				no_annot
1206	tgtaatacaa	9	TC14744	82	0	
1206	tgtaatacaa	9	CD063441	74	0	
1207	cttgtacaac	9				no_annot
1208	actttcgtgt	9	TC13899	91	0	
1209	tagtacgtcg	9				no_annot
1210	tcgacaatgt	9	TC17971	39	0	weakly similar to GP|20151695|gb|AAM11207.1||AY094854 RE13652p {Drosophila melanogaster}, partial (20%)
1211	gcaactaatg	9				no_annot
1212	caacaatgaa	9	TC12410	71	0	similar to SP|P53832|WSC2_YEAST Cell wall integrity and stress response component 2 precursor. [Baker's yeast] {Saccharomyces cerevisiae}, partial (5%)
1213	aagaggtcct	9	TC8109	93	0	weakly similar to GP|5853321|gb|AAD54421.1||AF181685 membrane protein TMS-2 {Mus musculus}, partial (33%)
1214	tcgaggttct	9	TC9287	82	0	
1215	ccagtggata	9	TC10304	67	1	weakly similar to GP|27262947|emb|CAD59550.1||AJ535485 UDP-Glc/Gal endoplasmic reticulum nucleotide sugar transporter {Gallus gallus}, partial (45%)
1216	actgctgata	9				no_annot
1217	ccaaaccttt	9	AI111000	12	0	
1218	tctgatcgtc	9	TC10979	69	0	
1219	tactacaatc	9				no_annot
1220	tgtaaaatgt	9	TC8613	50	2	
1221	tgacagcaga	9	TC11900	85	1	
1222	tgctcactgc	9				no_annot
1223	ataagtcgat	9				no_annot
1224	tagaaggact	9	TC12685	52	0	
1225	gcttcgtgtt	9				no_annot

1226	ggatacctcc	9	TC13738	83	0	weakly similar to SP|P26268|ODPT_ASCSU Pyruvate dehydrogenase E1 component alpha subunit type II, mitochondrial precursor(PDHE1-A) (Fragment). [Pig roundworm, Ascaris lumbricoides] {Ascaris suum}, partial (74%)
1227	tcgtttctgt	9	TC8136	32	5	similar to GP|16769222|gb|AAL28830.1||AY061282 LD20087p {Drosophila melanogaster}, partial (7%)
1227	tcgtttctgt	9	TC8190	81	0	
1228	ccttccatca	9	TC16737	82	0	weakly similar to GP|13529599|gb|AAH05509.1||BC005509 Ldh1 protein {Mus musculus}, partial (34%)
1229	gaaagaccag	9	N20704	68	0	
1230	tgcttgtcaa	9	TC10452	41	5	similar to GP|28630200|gb|AAN77888.1||AY130369 ribosomal protein S5 {Branchiostoma lanceolatum}, partial (97%)
1230	tgcttgtcaa	9	TC10452	41	0	similar to GP|28630200|gb|AAN77888.1||AY130369 ribosomal protein S5 {Branchiostoma lanceolatum}, partial (97%)
1231	gtatttatgt	9	TC17413	13	0	weakly similar to GP|18481626|gb|AAL73488.1|AF464905_1|AF464905 repressor protein {Zea mays}, partial (9%)
1232	cgtgctggtc	9	AA791327	12	1	similar to GP|1842152|dbj|BAA19226.1||AB001052 histone H2A-like protein {Bombyx mori}, partial (41%)
1233	gtatttatgg	9	AF130788_1003_2262	78	1	[CDS] NADH dehydrogenase 4 (ND4) gene, complete cds
1234	aaaccactgt	9				no_annot
1235	ttagtgtatc	9				no_annot
1236	gattactcac	9				no_annot
1237	tgtggtgtac	9	TC16826	2	4	similar to EGAD|31660|4267 splicing factor 9G8, alt. transcript 3 {Homo sapiens}, partial (61%)
1237	tgtggtgtac	9	TC16826	2	3	similar to EGAD|31660|4267 splicing factor 9G8, alt. transcript 3 {Homo sapiens}, partial (61%)
1237	tgtggtgtac	9	TC16827	1	1	weakly similar to GP|6433840|emb|CAB60724.1||AJ249466 DX|6 protein {Drosophila melanogaster}, partial (32%)
1237	tgtggtgtac	9	TC16827	1	0	weakly similar to GP|6433840|emb|CAB60724.1||AJ249466 DX|6 protein {Drosophila melanogaster}, partial (32%)

1237	tgtggtgtac	9	TC16828	35	0	weakly similar to GP|1086592|gb|AAA82270.1||U41007 Sr protein (splicing factor) protein 6, isoform a {Caenorhabditis elegans}, partial (37%)
1238	actaattgaa	9				no_annot
1239	tttttcaatg	9	TC10604	93	0	weakly similar to SP|Q99733|NPL4_HUMAN Nucleosome assembly protein 1-like 4 (Nucleosome assembly protein 2) (NAP2). [Human] {Homo sapiens}, partial (31%)
1239	tttttcaatg	9	TC18821	95	0	similar to GP|4679150|gb|AAD27038.1|AF095316_1|AF095316 cytochrome b {Heterotis niloticus}, partial (10%)
1239	tttttcaatg	9	CD161924	2	0	
1240	tttcggcgtt	9	TC13691	92	0	
1241	gtaaacgtaa	9	TC11005	70	0	
1242	agttcttctt	9				no_annot
1243	aacttaaagc	8	TC17395	93	0	
1244	cttgtttatt	8	TC15240	59	0	
1245	gttatgagcg	8	TC17199	78	1	weakly similar to GP|16768548|gb|AAL28493.1||AY060945 GM08440p {Drosophila melanogaster}, partial (22%)
1246	aagacagtcg	8				no_annot
1247	atcacagggg	8				no_annot
1248	catcagcctg	8				no_annot
1249	cttacttcct	8	C604332.1	96	0	
1250	ttgatccagc	8				no_annot
1251	gaatacgaaa	8				no_annot
1252	cgctagataa	8				no_annot
1253	aactgtattc	8				no_annot
1254	tatccaggat	8	TC10827	94	0	similar to SP|P38062|AMP2_RAT Methionine aminopeptidase 2(MetAP 2) (Peptidase M 2) (Initiation factor 2 associated 67 kDa glycoprotein) (p67) (p67eIF2). [Rat] {Rattus norvegicus}, partial (76%)
1255	agcgtaaact	8				no_annot
1256	catccgtcag	8				no_annot

1257	atgaactagt	8	TC17501	82	1	weakly similar to SP|Q9VCE1|BCN1_DROME Beclin 1-like protein. [Fruit fly] {Drosophila melanogaster}, partial (22%)
1257	atgaactagt	8	CD074889	85	0	
1258	gtgatgcagg	8	C317565.1	92	0	
1259	atcgtggttt	8	TC14674	72	0	weakly similar to GP|4389443|gb|AAD19813.1||AE001572 SPC 21-kDa-like {Drosophila melanogaster}, partial (86%)
1260	tctatccctt	8				no_annot
1261	atgagcttag	8	TC8988	74	0	weakly similar to GP|13195570|gb|AAK15762.1|AF329820_1|AF329820 cornichon {Boltenia villosa}, partial (93%)
1262	gtcccagttt	8	C612462.1	93	0	
1263	gatgagattg	8	TC10310	76	0	similar to GP|2760013|emb|CAA11175.1||AJ223200 actin related protein {Lumbricus rubellus}, partial (98%)
1264	attgattgcg	8				no_annot
1265	tgcattgtca	8				no_annot
1266	cctgaagttt	8				no_annot
1267	gattaccgat	8				no_annot
1268	gaagcgtttg	8	TC17847	82	0	similar to GP|11999092|gb|AAG43048.1|AF077615_1|AF077615 TGF beta inducible nuclear protein TINP1 {Homo sapiens}, partial (68%)
1268	gaagcgtttg	8	TC19495	33	0	similar to GP|11999092|gb|AAG43048.1|AF077615_1|AF077615 TGF beta inducible nuclear protein TINP1 {Homo sapiens}, partial (39%)
1269	aatagctcgt	8				no_annot
1270	ttacttttta	8	TC10735	70	2	similar to SP|Q9VC49|RPBX_DROME DNA-directed RNA polymerase II 7.6 kDa polypeptide(RPB10) (RPB7.6). [Fruit fly] {Drosophila melanogaster}, partial (97%)
1270	ttacttttta	8	CD127132	76	1	
1271	gtacagccca	8				no_annot
1272	ggaaagttca	8	C612146.1	91	0	
1273	acgggagaga	8				no_annot

1274	gtccactcca	8	TC11398	74	0	weakly similar to GP|5738218|gb|AAD50298.1|AF174582_1|AF174582 inorganic pyrophosphatase {Torpedo marmorata}, partial (59%)
1275	aaaatcaaag	8	C312487.1	76	0	
1276	ctgaccacct	8	C608287.1	83	0	
1277	tgtttcggac	8				no_annot
1278	ttgtacattc	8	TC17574	98	0	
1279	gggttatcgt	8	TC8634	58	0	
1279	gggttatcgt	8	CD089245	32	0	
1280	ttgttgtgtt	8				no_annot
1281	ttcacagata	8				no_annot
1282	agtgatagtt	8	TC17154	92	0	weakly similar to SP|O43776|SYN_HUMAN Asparaginyl-tRNA synthetase, cytoplasmic(Asparagine--tRNA ligase) (AsnRS). [Human] {Homo sapiens}, partial (31%)
1283	cgatatctcc	8	TC10935	81	2	
1284	cgcataaaaa	8				no_annot
1285	gtggtgaaat	8				no_annot
1286	tcaatttcct	8	AF128877|Sma.108	90	0	Enterokinase-like protein (EKL)
1287	attcaatggc	8	AF065599|Sma.36	77	0	Annexin
1288	actgggtgat	8	TC14396	79	0	similar to SP|P53702|CCHL_MOUSE Cytochrome c-type heme lyase(CCHL) (Holocytochrome c- type synthase). [Mouse] {Mus musculus}, partial (54%)
1289	ttttgttgcg	8				no_annot
1290	cgtaagtaag	8	TC9579	62	0	
1290	cgtaagtaag	8	TC14339	97	0	
1290	cgtaagtaag	8	CD170951	31	1	
1291	gatcgctacg	8	CD079863	65	0	weakly similar to SP|Q9UHL8|I13A_HUMAN Mitochondrial import inner membrane translocase subunit TIM13 A. [Rat] {Rattus norvegicus}, partial (53%)
1292	actggtggct	8	TC17066	67	0	weakly similar to GP|22946147|gb|AAF52935.2||AE003628 CG5362-PA {Drosophila melanogaster}, partial (85%)
1293	gattgttcag	8				no_annot

1294	gtcaccgtat	8				no_annot
1295	atgcacttct	8	U55769|Sma.241	68	0	T-complex polypeptide 1 alpha subunit (SmTCP-1A)
1296	aattgtgatt	8	TC12820	67	0	
1297	tcaaacatca	8	U47862|Sma.628	88	0	Gynecophoral canal protein
1298	tttagtccac	8	TC7677	94	0	similar to GP|7677478|gb|AAF67180.1|AF241222_1|AF241222 mitochondrial import protein {Drosophila melanogaster}, partial (14%)
1299	aatcaatgct	8				no_annot
1300	tgtatatgta	8	TC8665	96	0	similar to GP|18482486|gb|AAL68961.1||AY072917 RNA-binding protein splice variant a {Homo sapiens}, partial (24%)
1301	tgtaacagtg	8	TC8168	4	2	
1302	tcaaaagaag	8				no_annot
1303	ccattcgatt	8	TC10508	8	3	
1304	tgtgtaaatt	8	TC15036	6	1	
1305	gaaagcgtaa	8	TC14646	89	1	weakly similar to GP|22945397|gb|AAN10419.1||AE003583 CG31686-PA {Drosophila melanogaster}, partial (15%)
1306	gtaaccgtgc	8				no_annot
1307	atcaccatca	8				no_annot
1308	aacaatggga	8				no_annot
1309	cacttgcagt	8	TC13884	35	0	weakly similar to GP|7296057|gb|AAF51353.1||AE003585 CG7361-PA {Drosophila melanogaster}, partial (14%)
1309	cacttgcagt	8	TC13885	19	0	weakly similar to GP|5042402|gb|AAD38242.1|AC007786_1|AC007786 UCRI_HUMAN; RIESKE IRON-SULFUR PROTEIN; RISP {Homo sapiens}, partial (46%)
1310	tgtgtaaaga	8				no_annot
1311	gtttgaacac	8	TC11010	83	0	similar to SP|O16520|ERF1_CAEEL Eukaryotic peptide chain release factor subunit 1 (eRF1) (Eukaryotic release factor 1). {Caenorhabditis elegans}, partial (36%)
1312	gcaaatgttc	8	TC14321	3	4	
1313	aagaaaaaac	8	CD081667	91	0	similar to GP|15488666|gb|AAH13480.1||BC013480 cystathionine beta-synthase {Mus musculus}, partial (23%)

1314	tacctgtgag	8	C611747.1	94	0	
1315	tgctcgtttt	8				no_annot
1316	cattcaccag	8	TC9219	66	0	
1317	taaacacagt	8	TC17412	88	0	weakly similar to GP|1244714|gb|AAB02192.1||U41843 Dr1-associated corepressor {Homo sapiens}, partial (25%)
1318	taccggcgta	8				no_annot
1319	cttcgctata	8				no_annot
1320	cttaaggaac	8	TC17484	67	0	
1321	gaaacccttg	8				no_annot
1322	gaggtctgga	8				no_annot
1323	gtaatattga	8				no_annot
1324	tatattcgac	8	CD081775	79	1	similar to SP|P15927|RFA2_HUMAN Replication protein A 32 kDa subunit (RP-A) (RF-A) (Replication factor-A protein 2). [Human] {Homo sapiens}, partial (9%)
1325	attatctaaa	8	C202393.1	15	5	
1325	attatctaaa	8	C603563.1	15	5	
1326	taatctgtca	8				no_annot
1327	actctgtgag	8	TC10764	55	0	weakly similar to SP|Q924T2|RT02_MOUSE Mitochondrial 28S ribosomal protein S2 (MRP-S2). [Mouse] {Mus musculus}, partial (67%)
1328	acaaaagaag	8	TC10541	31	1	homologue to GP|21686538|gb|AAM74948.1|AF519808_1|AF519808 MA {Schistosoma japonicum}, partial (11%)
1329	attcctttcc	8				no_annot
1330	gcagattgcc	8	CD138753	27	1	
1331	tgcagtaatc	8				no_annot
1332	acgacttgga	8	C302049.1	28	0	
1333	agggatataa	8				no_annot
1334	ggcttacgaa	8	TC14314	70	0	
1335	ggcgctagaa	8				no_annot
1336	ttagtgtttg	8				no_annot
1337	agatttatcg	8	BG931622	75	0	
1338	cactgtgaaa	8				no_annot

1339	atttgaagtc	8	TC8917	89	0	homologue to SP|P06704|CC31_YEAST Cell division control protein 31. [Baker's yeast] {Saccharomyces cerevisiae}, partial (7%)
1340	attgctttga	8	C609110.1	98	0	
1341	tttaaattat	8	X98619|Sma.12	69	0	Sm10 protein
1342	tgacagcccg	8				no_annot
1343	gtcataaatt	8				no_annot
1344	gtgaatgtca	8				no_annot
1345	tttgtcttcg	8				no_annot
1346	ttttgccgct	8	TC7853	71	0	weakly similar to GP|6010208|emb|CAB57276.1||X77725 PIG-A {Homo sapiens}, partial (38%)
1346	ttttgccgct	8	CD191226	62	0	
1347	gttattattg	8				no_annot
1348	tcagcaatgc	8	TC7719	64	4	weakly similar to GP|16769502|gb|AAL28970.1||AY061422 LD35289p {Drosophila melanogaster}, partial (18%)
1349	gcaatagaag	8	TC19169	69	0	weakly similar to PIR|T00741|T00741 NADH2 dehydrogenase (ubiquinone)acyl carrier chain, mitochondrial - human (fragment), partial (39%)
1350	caaacaatga	8				no_annot
1351	tgcttgttga	8	TC10838	91	0	similar to GP|10716080|dbj|BAB16369.1||AB047321 endoglycoceramidase {Cyanea nozakii}, partial (4%)
1352	caccacaacc	8				no_annot
1353	tataaaatct	8	CD169284	48	1	
1354	aagtgtaggt	8				no_annot
1355	gctatcgcaa	8				no_annot
1356	ttttcgtgta	8	TC17316	63	0	similar to SP|P35128|UBC3_DROME Ubiquitin-conjugating enzyme E2-17 kDa(Ubiquitin-protein ligase) (Ubiquitin carrier protein) (Bendless protein). [Fruit fly] {Drosophila melanogaster}, partial (98%)
1357	ttctaattgt	8	TC18499	98	0	weakly similar to GP|868012|dbj|BAA07268.1||D38074 xRlf beta subunit (p100 protein) {Xenopus laevis}, partial (5%)
1358	taacatacac	8	CD081660	82	0	weakly similar to GP|21430284|gb|AAM50820.1||AY118960 LD38689p {Drosophila melanogaster}, partial (12%)

1359	gtaaaatgca	8				no_annot
1360	gaatttcatt	8	TC10535	39	2	weakly similar to PIR|PN0574|PN0574 mucin-like protein - rat (fragment), partial (41%)
1360	gaatttcatt	8	TC16740	86	0	
1361	cttggaacaa	8				no_annot
1362	cccttaagaa	8				no_annot
1363	acatatgcta	8	TC18231	94	0	
1364	catcatcact	8				no_annot
1365	aatctcattc	8				no_annot
1366	tatgcattat	8	TC17485	95	0	weakly similar to GP|5757655|gb|AAD50534.1|AF060089_1|AF060089 proteasome subunit C8 {Mus musculus}, partial (59%)
1367	aaactgatac	8	TC8102	57	0	weakly similar to GP|2707597|gb|AAB92355.1||U89438 nonstructural protein P125-2 {pestivirus type 1} [Pestivirus type 1], partial (15%)
1368	ataaggttgc	8	CD092749	3	2	
1369	atggtccaaa	8	TC8240	90	0	similar to GP|21552719|gb|AAM62284.1|AF399717_1|AF399717 kynurenine formamidase {Mus musculus}, partial (6%)
1370	tttgccgtca	8	C605171.1	25	2	
1371	agctttcgtt	8	TC7365	95	2	PIR|I51382|I51382 achaete-scute homolog - chicken, partial (7%)
1372	acgagtggtt	8				no_annot
1373	atgatgatgg	8	C316930.1	54	0	
1373	atgatgatgg	8	C607976.1	86	0	
1373	atgatgatgg	8	C708577.1	91	0	
1374	atttgtaaag	8				no_annot
1375	tgactgacta	8				no_annot
1376	tggctcagga	8				no_annot
1377	acgttattct	8				no_annot
1378	gatttgatcg	8	TC7721	84	0	similar to GP|7295549|gb|AAF50861.1||AE003569 CG1518-PA {Drosophila melanogaster}, partial (32%)
1379	tttactttaa	8				no_annot
1380	cccttcggtt	8	CD112273	8	1	

1381	taccctttta	8	J05410|Sma.765	94	0	ATP:guanidino kinase (Smc74)
1382	cttatcagta	8	CD127901	17	0	weakly similar to GP|13183088|gb|AAK15047.1|AF237684_1|AF237684 steroid dehydrogenase-like protein {Homo sapiens}, partial (21%)
1383	caataaattg	8	TC11770	96	0	weakly similar to GP|27497737|gb|AAO13168.1||AY171233 protein tyrosine phosphatase {Homo sapiens}, partial (6%)
1384	tatattgaac	8				no_annot
1385	tcggtgtctc	8				no_annot
1386	tgaatcacgg	8				no_annot
1387	cacgtgaaga	8	TC13974	62	0	similar to GP|3851616|gb|AAC72374.1||AF095939 succinate dehydrogenase Fp subunit {Gallus gallus}, partial (53%)
1388	ttggggtttt	8				no_annot
1389	ctgtccaata	8				no_annot
1390	cctcaaaagg	8	TC10561	30	2	similar to SP|P14118|RL19_HUMAN 60S ribosomal protein L19. [Rat] {Rattus norvegicus}, partial (90%)
1390	cctcaaaagg	8	TC10562	33	0	similar to GP|22758856|gb|AAN05588.1||AF526203 ribosomal protein L19 {Argopecten irradians}, partial (50%)
1391	tgttggttct	8	TC11462	89	0	similar to SP|O15491|AN1H_HUMAN WD-repeat protein An11 homolog. [Mouse] {Mus musculus}, partial (62%)
1392	ttgctggagt	8				no_annot
1393	caaaattgga	8				no_annot
1394	tcattaatta	8	TC14098	93	2	weakly similar to GP|7363256|dbj|BAA93011.1||AB032767 CD9 antigen {Gallus gallus}, partial (32%)
1395	ctgtaactta	8	TC11437	58	0	
1395	ctgtaactta	8	TC17251	84	0	similar to PIR|A47740|A47740 glucose-6-phosphate 1-dehydrogenase- fruit fly (Drosophila melanogaster) (strain OK93), partial (42%)
1396	aataactaaa	8	TC16971	93	2	weakly similar to GP|7329074|gb|AAF59902.1|AF177941_1|AF177941 collagen type V alpha 3 chain {Homo sapiens}, partial (25%)
1397	gcgcagtaca	8	C302039.1	19	2	

1397	gcgcagtaca	8	C605085.1	12	3	
1398	tcgtactatg	8				no_annot
1399	atgactcata	8				no_annot
1400	gcaggagaaa	8	C201185.1	14	2	
1400	gcaggagaaa	8	C604220.1	5	2	
1401	gcaaaagatt	7				no_annot
1402	aagaaggaga	7	TC10548	88	2	homologue to GP|2623606|gb|AAC48354.1||AF027877 mucin-like protein {Trypanosoma cruzi}, partial (10%)
1402	aagaaggaga	7	TC10549	0	7	
1402	aagaaggaga	7	TC10549	0	3	
1402	aagaaggaga	7	TC17008	73	0	similar to GP|967251|gb|AAA74951.1||U31460 Arf4 {Xenopus laevis}, complete
1403	tttagattaa	7				no_annot
1404	tggcaagtgg	7				no_annot
1405	ttgtgtgtgt	7				no_annot
1406	tacaacactt	7	TC12394	78	0	
1407	tgataaataa	7	TC10931	96	0	weakly similar to SP|P28687|SP22_CHICK Microsomal signal peptidase 23 kDa subunit(SPase 22 kDa subunit) (SPC22/23) (gp23). [Chicken] {Gallus gallus}, partial (52%)
1407	tgataaataa	7	TC17005	89	2	similar to GP|28916487|gb|AAO59412.1||AY226982 DnaJ-like protein {Schistosoma japonicum}, partial (85%)
1408	gtcaaatatt	7				no_annot
1409	tacagtcacc	7				no_annot
1410	agggctataa	7	C200912.1	0	2	
1410	agggctataa	7	C711228.1	71	0	
1411	atcaaacagc	7	CD061344	89	0	
1412	gtcacctctt	7				no_annot
1413	tgtgcaacgt	7	C200147.1	80	0	
1413	tgtgcaacgt	7	C601338.1	74	0	
1414	aagttgcttg	7				no_annot
1415	ataagtcctg	7				no_annot

1416	taaatccgaa	7				no_annot
1417	tatgtgaggg	7				no_annot
1418	cttcaaaggg	7	TC8896	74	0	similar to GP|21430280|gb|AAM50818.1||AY118958 LD37839p {Drosophila melanogaster}, partial (54%)
1419	ccaccacttg	7				no_annot
1420	gatcaatctt	7	TC17121	82	0	similar to GP|21430900|gb|AAM51128.1||AY119268 SD24339p {Drosophila melanogaster}, partial (21%)
1421	gtgatctcgg	7	TC17183	5	10	similar to SP|P51892|DNL1_XENLA DNA ligase I(Polydeoxyribonucleotide synthase [ATP]). [African clawed frog] {Xenopus laevis}, partial (39%)
1422	ataaacatac	7	TC7412	21	1	
1422	ataaacatac	7	CD197458	74	1	weakly similar to GP|1006605|dbj|BAA10758.1||D64005 slr0712 {Synechocystis sp. PCC 6803}, partial (7%)
1422	ataaacatac	7	BF936883	57	1	
1423	acacggaatg	7	C601690.1	6	2	
1424	ggcttaagtc	7				no_annot
1425	gacccaaagg	7				no_annot
1426	gaagaggtgg	7	C211053.1	95	0	
1426	gaagaggtgg	7	C315171.1	14	3	
1427	tacgaatagg	7				no_annot
1428	taaatgttat	7				no_annot
1429	agaaaagcca	7	TC15851	19	2	
1430	gagctgtcgt	7	TC10593	65	0	weakly similar to SP|Q9JJT0|RCL1_MOUSE RNA 3'-terminal phosphate cyclase-like protein. [Mouse] {Mus musculus}, partial (45%)
1431	caaattgtgt	7	TC11550	89	0	weakly similar to GP|21326755|emb|CAD29128.1||AJ439711 carbonic anhydrase {Riftia pachyptila}, partial (33%)
1432	agttgtgtaa	7				no_annot
1433	tggctaagac	7	C717275.1	59	1	
1434	cgtacttctt	7	N33679	23	0	
1435	tgataccttt	7	TC12288	94	0	
1436	cagctcaata	7				no_annot

1437	gcttaacagt	7	TC17805	79	0	weakly similar to GP|15291727|gb|AAK93132.1||AY051708 LD24671p {Drosophila melanogaster}, partial (21%)
1438	gcctcaagac	7	TC10933	70	0	weakly similar to GP|28971940|dbj|BAC07443.2||AP005194 P0565A07.3 {Oryza sativa (japonica cultivar-group)}, partial (6%)
1439	tacaaaatct	7	TC17286	59	0	weakly similar to SP|Q16550|SP41_HUMAN Transcription initiation protein SPT4 homolog 1. [Mouse] {Mus musculus}, partial (88%)
1439	tacaaaatct	7	BG930190	7	1	
1439	tacaaaatct	7	BG930183	8	0	similar to GP|22121986|gb|AAM89494.1||AY124053 cap binding protein {Verticillium fungicola}, partial (9%)
1440	cttgaatggt	7	AA559539	0	0	
1441	gatttgggac	7				no_annot
1442	tttgaatgca	7				no_annot
1443	gtgccagatt	7				no_annot
1444	tctgtacaca	7	TC7586	92	0	
1445	ttacttttct	7	TC14123	87	0	
1446	tcttggatgg	7				no_annot
1447	gtgatctata	7	TC17038	6	5	weakly similar to PIR|T39903|T39903 serine-rich protein - fission yeast (Schizosaccharomyces pombe), partial (5%)
1448	atcggattta	7	BG932322	78	0	
1449	tacaagagct	7	AI395155	22	0	weakly similar to PIR|JC7823|JC7823 elongation factor 1 gamma-subunit, silk gland - silkworm, partial (9%)
1450	ttttgctgtt	7	C207273.1	40	3	
1450	ttttgctgtt	7	C605499.1	89	0	
1451	caaaataccg	7				no_annot
1452	ttcatcgact	7	TC9994	52	2	weakly similar to GP|16589079|gb|AAL27006.1|AF416510_1|AF416510 WD repeat protein BIG-3 {Mus musculus}, partial (15%)
1453	gacatagatt	7	TC12565	93	0	
1454	ataggaagct	7				no_annot
1455	agctttgatt	7				no_annot
1456	tacaactaaa	7	TC16824	90	2	SP|P06198|MYSP_SCHMA Paramyosin. [Blood fluke] {Schistosoma mansoni}, complete

1457	tttacgatat	7	C202229.1	10	5	
1457	tttacgatat	7	C301167.1	8	4	
1457	tttacgatat	7	C604342.1	26	5	
1457	tttacgatat	7	C716005.1	8	4	
1458	atttggaaga	7	U67153|Sma.244	0	0	13 kDa tegumental antigen Sm13
1459	ttatgtgggg	7				no_annot
1460	tgactaccaa	7	TC15763	72	0	
1461	tggttatgtt	7				no_annot
1462	aatgtattgt	7				no_annot
1463	catagatcac	7				no_annot
1464	gcgaaaaagt	7	TC10704	73	0	weakly similar to GP|19263609|gb|AAH25076.1||BC025076 LOC216829 protein {Mus musculus}, partial (40%)
1465	tatgaaagaa	7	TC10913	54	0	similar to GP|9971613|dbj|BAB12680.1||AB039749 polypeptide release factor 3 {Kluyveromyces lactis}, partial (7%)
1465	tatgaaagaa	7	TC14462	40	0	weakly similar to SP|P35574|GDE_RABIT Glycogen debranching enzyme (Glycogen debrancher) [Includes: 4-alpha-glucanotransferase(Oligo-1,4-1,4-glucantransferase); Amylo-alpha-1,6-glucosidase(Amylo-1,6-glucosidase) (Dextrin 6-alpha-D-glucosidase)]. [Rabbit] {Oryctolagus cuniculus}, partial (7%)
1465	tatgaaagaa	7	CD201813	31	0	weakly similar to GP|23172527|gb|AAF56845.2||AE003768 CG1420-PA {Drosophila melanogaster}, partial (5%)
1466	tacactagac	7	TC8095	73	0	
1467	agattagttt	7	TC13524	64	0	weakly similar to PIR|S59513|S59513 collagen II A1 protein - zebra fish (fragment), partial (16%)
1468	ttagttttca	7	TC13839	78	0	weakly similar to PIR|S05583|XNCHDC aspartate transaminase, cytosolic - chicken, partial (59%)
1469	aacacactat	7				no_annot
1470	taacttttca	7	C612641.1	84	0	
1471	tctgtatgtg	7	TC15743	64	1	
1472	aagaaatctg	7	C205270.1	33	2	
1473	tatggtagtc	7				no_annot

1474	aacttgctgt	7	TC6939	81	0	
1475	gtaaaccatc	7	TC16823	88	0	
1476	tctttctcta	7				no_annot
1477	tagcgtgacg	7				no_annot
1478	ctatttttcc	7	TC16864	91	0	similar to GP|4886558|emb|CAB43352.1||AJ005937 lamin {Tealia sp.}, partial (7%)
1479	agaaatttga	7	TC14685	49	3	
1479	agaaatttga	7	CD134455	42	0	
1480	tacatcgaat	7	C301397.1	36	0	
1480	tacatcgaat	7	C700756.1	37	0	
1481	acagaaagtg	7	TC17326	25	1	
1482	tataggacta	7				no_annot
1483	gctactgttc	7	TC16579	45	0	
1484	tataacaggc	7	TC13531	95	0	
1485	cttataatct	7	gi|161041|gb|L07513.1|SCMMTEGANT	97	0	Schistosoma mansoni major tegumental antigen SM15 (GSM70) gene, complete cds
1486	aaagtagctt	7	TC8400	92	0	weakly similar to PIR|S65953|S65953 [phosphorylase] phosphatase65K regulatory chain isotype alpha - African clawed frog, partial (25%)
1486	aaagtagctt	7	TC17429	18	6	GP|5566124|gb|AAD45325.1|AF158102_1|AF158102 retinoid-x-receptor {Schistosoma mansoni}, complete
1487	agtagattca	7	TC17621	95	0	
1488	tacacagaaa	7	C603782.1	90	1	
1488	tacacagaaa	7	C704873.1	4	1	
1489	tgtataagta	7				no_annot
1490	ttctattgtt	7	TC7957	97	0	weakly similar to SP|Q96GM5|SCD1_HUMAN SWI/SNF related, matrix associated, actin dependent regulator of chromatin subfamily D member 1 (SWI/SNF complex 60 kDa subunit) (BRG1- associated factor 60a). [Human] {Homo sapiens}, partial (57%)
1490	ttctattgtt	7	TC13235	83	0	
1490	ttctattgtt	7	TC15996	32	1	
1491	tttattggtt	7	C602697.1	61	0	

1492	atcagacaac	7	TC11520	80	0	weakly similar to GP|13905162|gb|AAH06873.1||BC006873 LOC214597 protein {Mus musculus}, partial (26%)
1493	ttgtttgtcc	7				no_annot
1494	tataaaccag	7	TC17029	35	0	similar to SP|Q18885|BTF3_CAEEL Transcription factor BTF3 homolog. {Caenorhabditis elegans}, partial (55%)
1494	tataaaccag	7	CD137947	27	1	
1495	acttccaaat	7				no_annot
1496	gcgcacacac	7				no_annot
1497	atgactacga	7				no_annot
1498	aatagatcta	7				no_annot
1499	cctaaccctc	7	TC6906	64	0	weakly similar to GP|16769224|gb|AAL28831.1||AY061283 LD20186p {Drosophila melanogaster}, partial (10%)
1499	cctaaccctc	7	TC18072	27	0	
1499	cctaaccctc	7	CD185276	68	0	
1500	tattatggtg	7	TC11041	48	0	
1500	tattatggtg	7	TC19010	56	0	
1501	aggacacagg	7	C608629.1	20	1	
1502	gaacgctgct	7				no_annot
1503	gttccatcta	7	TC17282	92	0	weakly similar to GP|17862730|gb|AAL39842.1||AY069697 LD46767p {Drosophila melanogaster}, partial (23%)
1504	gtttcgtgat	7				no_annot
1505	tggtcatagg	7				no_annot
1506	caaaccatat	7	TC8643	2	8	
1507	tgtcgtttcc	7	TC16632	44	1	
1507	tgtcgtttcc	7	TC16634	56	0	
1507	tgtcgtttcc	7	TC16635	87	0	
1507	tgtcgtttcc	7	TC16636	77	0	
1507	tgtcgtttcc	7	TC16642	92	0	
1508	agtttgatgt	7				no_annot

1509	gtgtcaagta	7	TC10157	39	0	weakly similar to GP|17945992|gb|AAL49040.1||AY071418 RE49934p {Drosophila melanogaster}, partial (27%)
1510	tgaagtttat	7	TC14900	32	1	
1511	cattttccaa	7	TC7342	65	2	
1511	cattttccaa	7	TC7347	63	0	
1512	tgggtaacta	7				no_annot
1513	acaatcaata	7	TC14694	96	0	
1514	ctaatctggg	7				no_annot
1515	gatctcacac	7				no_annot
1516	gctcctttct	7	TC7612	72	0	
1517	ttaatctgct	7				no_annot
1518	cctattattg	7				no_annot
1519	tgtgcttgtg	7				no_annot
1520	ctttgaagag	7	TC11673	92	0	weakly similar to GP|28381597|gb|AAF46477.2||AE003446 CG15797-PA {Drosophila melanogaster}, partial (11%)
1521	aactctggcc	7	TC7543	28	0	weakly similar to GP|15022507|gb|AAK77961.1|AF315632_1|AF315632 coactivator activator {Homo sapiens}, partial (5%)
1522	cgcagttcaa	7	TC16962	65	1	
1523	tacttgtgtg	7	TC9144	95	0	
1524	ggatttgtaa	7				no_annot
1525	gaaacgttat	7				no_annot
1526	tatatgtttg	7	TC10568	97	0	similar to GP|17862622|gb|AAL39788.1||AY069643 LD40966p {Drosophila melanogaster}, partial (12%)
1527	ctttgttcgc	7				no_annot
1528	acgaataaag	7				no_annot
1529	acttgagggg	7				no_annot
1530	ttagccgcca	7				no_annot
1531	ggatcgtaat	7	TC17426	66	4	
1532	tagactgtat	7				no_annot
1533	cgtttgaaag	7				no_annot

1534	taaaaaaaaa	7	U30259|Sma.804	97	0	Trans-spliced mRNA, clone SL cDNA-14
1534	taaaaaaaaa	7	L31531|Sma.694	98	0	Phosphofructokinase (PFK)
1535	aataacatta	7	TC7791	90	0	weakly similar to GP|5360166|gb|AAD42887.1|AF158186_1|AF158186 pEachy {Rattus norvegicus}, partial (83%)
1536	atcagattgt	7	TC10924	15	3	
1536	atcagattgt	7	AI740432	9	1	
1537	tccacctgat	7	AY277535|Sma.73	99	0	Isolate S47 high voltage-activated calcium channel beta subunit 1
1537	tccacctgat	7	gi|33465408|gb|AY277536.1|	99	0	Schistosoma mansoni isolate S47 high voltage-activated calcium channel beta subunit 1 mRNA, complete cds
1537	tccacctgat	7	gi|33465404|gb|AY277534.1|	99	0	Schistosoma mansoni strain Gene Pool high voltage-activated calcium channel beta subunit 1 mRNA, complete cds
1537	tccacctgat	7	gi|33465402|gb|AY277533.1|	99	0	Schistosoma mansoni strain Puerto Rican high voltage-activated calcium channel beta subunit 1 mRNA, complete cds
1537	tccacctgat	7	gi|15283996|gb|AY033598.1|	99	0	Schistosoma mansoni high voltage-activated calcium channel beta subunit CavB1 mRNA, complete cds
1538	tatcgaatca	7				no_annot
1539	ttcatcacta	7	TC10449	64	4	weakly similar to GP|2636680|gb|AAC06263.1||U66331 pol {Schistosoma mansoni}, partial (3%)
1540	cggcagtggt	7	AY838776|Sma.5388	88	0	Clone C09 Perere 3 retrotransposon mRNA, partial sequence
1541	actttaatcc	7	TC17736	52	0	
1542	catcgtaata	7				no_annot
1543	taatacttta	7				no_annot
1544	tatcgttatc	7				no_annot
1545	cttcccaacc	7				no_annot
1546	aagtttgtac	7	TC13767	6	1	
1547	tagtcgctat	7				no_annot
1548	aatcgcacaa	7				no_annot
1549	tttaaattcc	7	TC8438	95	0	weakly similar to GP|10945625|gb|AAG24620.1|AF299073_1|AF299073 Niemann-Pick type C1 disease protein {Bos taurus}, partial (9%)

1550	tatttagcac	7				no_annot
1551	actgtccgaa	7	TC15696	29	0	
1552	cgtggtacct	7				no_annot
1553	gagaattaaa	7	TC14236	89	0	weakly similar to PIR|T30855|T30855 multidrug resistance protein 2 - fluke (Schistosoma mansoni), partial (15%)
1554	gccgttctta	7	TC7202	23	0	similar to GP|16930529|gb|AAL31950.1||AF421549 CDH1-D {Gallus gallus}, partial (7%)
1555	atatattgaa	7	TC7074	51	0	
1555	atatattgaa	7	TC7285	29	0	
1555	atatattgaa	7	TC14979	41	2	
1555	atatattgaa	7	TC17535	48	1	
1555	atatattgaa	7	TC19350	28	0	
1555	atatattgaa	7	CD063964	32	0	
1556	ccatatccca	7				no_annot
1557	tacttatcgt	7	TC7818	90	0	weakly similar to SP|P29052|TF2B_DROME Transcription initiation factor IIB (General transcription factor TFIIB). [Fruit fly] {Drosophila melanogaster}, partial (70%)
1557	tacttatcgt	7	TC11516	25	6	weakly similar to SP|O43309|Z305_HUMAN Zinc finger protein 305. [Human] {Homo sapiens}, partial (9%)
1557	tacttatcgt	7	CD199970	27	0	
1558	tatgaacgtg	7	TC7954	87	0	weakly similar to GP|4530295|gb|AAD21971.1||AF106679 WD-40 repeat protein {Drosophila melanogaster}, partial (57%)
1558	tatgaacgtg	7	BF936034	8	1	
1559	tttattccca	7				no_annot
1560	atgtttgccg	7	C207064.1	65	0	
1561	gaattcgaaa	7				no_annot
1562	ggtcaggcgg	7	TC7143	94	0	similar to GP|16930529|gb|AAL31950.1||AF421549 CDH1-D {Gallus gallus}, partial (19%)
1562	ggtcaggcgg	7	BG931906	84	0	homologue to EGAD|131264|140075 hemolysin {Acanthamoeba polyphaga}, partial (11%)
1562	ggtcaggcgg	7	BG932855	79	0	homologue to EGAD|131264|140075 hemolysin {Acanthamoeba polyphaga}, partial (11%)

1562	ggtcaggcgg	7	BG932872	85	0	homologue to EGAD|131264|140075 hemolysin {Acanthamoeba polyphaga}, partial (11%)
1562	ggtcaggcgg	7	BG932883	74	0	homologue to EGAD|131264|140075 hemolysin {Acanthamoeba polyphaga}, partial (11%)
1562	ggtcaggcgg	7	BG932892	84	0	homologue to EGAD|131264|140075 hemolysin {Acanthamoeba polyphaga}, partial (11%)
1563	gacaatacgt	7	TC18675	87	0	similar to GP|1143065|gb|AAB17403.1||U29765 alpha-L-fucosidase {Canis familiaris}, partial (8%)
1564	gttgtcgtgt	7	TC13693	65	1	weakly similar to SP|P28650|PUA1_MOUSE Adenylosuccinate synthetase, muscle isozyme(IMP--aspartate ligase) (AdSS) (AMPSase). [Mouse] {Mus musculus}, partial (65%)
1565	tcaatcgatt	7	TC8294	79	0	weakly similar to GP|22758864|gb|AAN05591.1||AF526208 ribosomal protein L7 {Argopecten irradians}, partial (17%)
1566	tacacaactc	7				no_annot
1567	aacgatttcc	7	AF183577|Sma.103	94	0	Alpha 1-3 fucosyltransferase (FucTA)
1568	ttacaattcc	7				no_annot
1569	aaagtcaatt	7	W06726	20	1	
1570	ctttgtgttc	7				no_annot
1571	tggaacttac	7				no_annot
1572	tcgaccaaaa	7	CD096548	37	0	weakly similar to GP|5107178|gb|AAD40001.1|AF150095_1|AF150095 small zinc finger-like protein {Ciona intestinalis}, partial (35%)
1573	atgcacctaa	7	TC8644	84	0	
1574	attctcttgg	7	C202023.1	28	0	
1574	attctcttgg	7	C602915.1	26	0	
1575	tggaatatat	7	TC8115	47	1	
1575	tggaatatat	7	TC9704	83	0	
1575	tggaatatat	7	CD117571	41	2	
1575	tggaatatat	7	CD117575	41	2	
1576	tgaatttttt	7	TC16539	90	2	similar to GP|27526313|emb|CAD45181.1||AJ506761 transketolase {Echinococcus multilocularis}, partial (80%)

1577	cagtgaagtt	7	TC13686	55	3	weakly similar to GP|5052618|gb|AAD38639.1|AF145664_1|AF145664 BcDNA.GH11110 {Drosophila melanogaster}, partial (13%)
1578	tgtggtggta	7	TC6880	76	3	similar to GP|17944974|gb|AAL48550.1||AY070928 RE03018p {Drosophila melanogaster}, partial (3%)
1578	tgtggtggta	7	TC6904	43	1	similar to GP|16040981|dbj|BAB69692.1||AB059656 POEM {Mus musculus}, partial (4%)
1578	tgtggtggta	7	TC7036	10	7	similar to SP|Q12224|RLM1_YEAST Transcription factor RLM1. [Baker's yeast] {Saccharomyces cerevisiae}, partial (3%)
1578	tgtggtggta	7	TC7243	78	0	similar to PIR|T39903|T39903 serine-rich protein - fission yeast (Schizosaccharomyces pombe), partial (4%)
1578	tgtggtggta	7	CD154267	17	4	
1578	tgtggtggta	7	CD097889	31	2	
1579	tacttgtctt	7				no_annot
1580	gaccacgaga	7	AI740290	87	0	
1581	tcagatcaac	7	AI977709	80	0	SP|P20287|G3P_SCHMA Glyceraldehyde 3-phosphate dehydrogenase(GAPDH) (Major larval surface antigen) (P-37). [Blood fluke] {Schistosoma mansoni}, partial (37%)
1582	ttacattaat	7	TC17320	83	4	weakly similar to GP|22324204|emb|CAC82991.1||AJ344146 eIF2B-alpha protein {Drosophila melanogaster}, partial (32%)
1582	ttacattaat	7	TC18480	82	0	
1583	tatgttttga	7	C301456.1	48	0	
1583	tatgttttga	7	C708599.1	47	0	
1584	ataccgcgtt	7				no_annot
1585	caatcgaaac	7				no_annot
1586	atgatgattt	7	TC13468	92	2	similar to GP|17945787|gb|AAL48941.1||AY071319 RE34144p {Drosophila melanogaster}, partial (98%)
1586	atgatgattt	7	TC18787	83	0	
1587	gccttaaacc	7	TC13774	90	0	weakly similar to GP|6467119|dbj|BAA86961.1||AB026125 ART-4 {Homo sapiens}, partial (17%)

1588	caacaaatca	7	TC9523	95	0	similar to GP|4325316|gb|AAD17329.1||AF125042 bisphosphate 3'-nucleotidase {Homo sapiens}, partial (5%)
1588	caacaaatca	7	TC11334	84	0	
1588	caacaaatca	7	TC12202	83	0	
1588	caacaaatca	7	TC12342	92	0	
1588	caacaaatca	7	TC13785	95	0	similar to GP|15291891|gb|AAK93214.1||AY051790 LD30634p {Drosophila melanogaster}, partial (6%)
1588	caacaaatca	7	TC14348	86	0	similar to SP|Q06136|FVT1_HUMAN Follicular variant translocation protein 1 precursor (FVT-1). [Human] {Homo sapiens}, partial (6%)
1588	caacaaatca	7	TC14496	87	0	
1588	caacaaatca	7	TC15022	97	0	
1588	caacaaatca	7	TC15627	96	0	weakly similar to GP|22831969|gb|AAF46404.2||AE003444 CG1795-PA {Drosophila melanogaster}, partial (9%)
1588	caacaaatca	7	TC15791	74	0	
1588	caacaaatca	7	TC18325	76	0	
1588	caacaaatca	7	TC18365	97	0	
1588	caacaaatca	7	CD064575	23	2	
1588	caacaaatca	7	CD148520	87	0	
1588	caacaaatca	7	AW017316	94	0	
1588	caacaaatca	7	CD199841	85	0	
1588	caacaaatca	7	CD199880	86	0	
1588	caacaaatca	7	CD199907	83	0	
1588	caacaaatca	7	CD199923	92	0	weakly similar to GP|23171428|gb CG4261-PA {Drosophila melanogaster}, partial (1%)
1588	caacaaatca	7	AI975513	80	0	
1589	gacaagagga	7	TC8724	60	0	weakly similar to GP|5052566|gb|AAD38613.1|AF145638_1|AF145638 l.2.35Df {Drosophila melanogaster}, partial (19%)
1589	gacaagagga	7	TC14168	71	1	similar to PIR|T39903|T39903 serine-rich protein - fission yeast (Schizosaccharomyces pombe), partial (6%)
1590	tatatgtata	7	TC17373	7	3	
1590	tatatgtata	7	CD075257	18	1	

1591	gtggtctagc	7	TC7313	12	1	
1591	gtggtctagc	7	TC7319	13	3	
1591	gtggtctagc	7	TC7329	93	0	
1591	gtggtctagc	7	TC9841	13	3	
1591	gtggtctagc	7	TC10108	73	1	
1591	gtggtctagc	7	TC14519	37	1	
1591	gtggtctagc	7	TC14958	81	1	
1591	gtggtctagc	7	TC19698	18	0	
1591	gtggtctagc	7	CD084755	26	2	
1591	gtggtctagc	7	CD089078	4	3	
1591	gtggtctagc	7	CD127705	18	3	
1591	gtggtctagc	7	CD060526	61	3	
1591	gtggtctagc	7	CD070143	8	2	
1591	gtggtctagc	7	CD085197	38	2	homologue to GP|29898730|gb|AAP12002.1||AE017014 Murein hydrolase exporter {Bacillus cereus ATCC 14579}, partial (10%)
1591	gtggtctagc	7	CD125767	65	1	
1591	gtggtctagc	7	CD147410	30	1	
1591	gtggtctagc	7	CD202263	89	1	similar to GP|15292589|gb|AAK93563.1||AY052139 SD09738p {Drosophila melanogaster}, partial (4%)
1592	aattgaatta	7	L25065|Sma.629	83	0	Glucose transporter protein (GTP1)
1593	tcaacacacg	7	TC10878	16	0	weakly similar to GP|12005495|gb|AAG44477.1|AF242729_1|AF242729 HT022 {Homo sapiens}, partial (19%)
1594	tattcttata	7				no_annot
1595	ctaatatttc	7	TC11497	10	3	
1596	tactaccgta	7				no_annot
1597	gaagatgctg	7	TC11044	43	0	weakly similar to GP|22268103|gb|AAH27427.1||BC027427 STIP1 homology and U-Box containing protein 1 {Mus musculus}, partial (69%)
1597	gaagatgctg	7	TC11606	49	3	weakly similar to GP|7294667|gb|AAF50005.1||AE003543 CG14130-PA {Drosophila melanogaster}, partial (21%)

1598	gtcctatgat	7	TC14312	93	0	similar to GP|1232077|dbj|BAA12177.1||D83987 huMCM2 {Homo sapiens}, partial (30%)
1598	gtcctatgat	7	AW087127	72	0	
1599	acagtacatc	7	TC17390	65	0	
1600	aaacatactc	7				no_annot
1601	tctattcggg	7				no_annot
1602	tgtatagttt	7	TC14703	23	5	
1602	tgtatagttt	7	TC14834	99	0	weakly similar to PIR|S21977|S21977 Pm5 protein - human, partial (6%)
1603	atcgaaatag	7	TC16981	83	0	GP|4928143|gb|AAD33428.1|AF129816_1|AF129816 retinoid X receptor RXR-2 {Schistosoma mansoni}, complete
1604	aaaatacata	7				no_annot
1605	ttgaagtgct	7				no_annot
1606	taatctcact	7				no_annot
1607	tatgtaagtt	7	TC13467	12	2	PIR|B54525|B54525 major female-specific polypeptide (frame 2) - fluke (Schistosoma mansoni) (fragment), partial (48%)
1608	aaagtaacta	7				no_annot
1609	gttagcgcct	7	TC8269	96	0	
1610	aacatcaccc	7				no_annot
1611	tgtatgtatg	7	CD131675	84	0	
1612	gtgtgtgata	7				no_annot
1613	gatgctgagg	7	TC18966	76	0	similar to PIR|T31655|T31655 DNA excision repair cross-complementing protein - sea squirt (Ciona intestinalis), partial (24%)
1614	gactggatac	7	CD193818	48	0	
1615	ttcaactctg	7				no_annot
1616	attatgaaaa	7	TC10613	55	5	weakly similar to GP|12006785|gb|AAG44917.1|AF290330_1|AF290330 phosphoglucomutase {Drosophila melanogaster}, partial (52%)
1617	acgtgccaga	7	C607187.1	28	1	
1618	ttcaagcatc	7	TC9700	31	1	
1619	gataacagta	7				no_annot

1620	tccattatgt	7	TC11469	31	3	weakly similar to SP|Q03655|GAS3_YEAST GAS3 protein precursor. [Baker's yeast] {Saccharomyces cerevisiae}, partial (5%)
1620	tccattatgt	7	CD201930	3	0	
1621	gaaacagcgc	7	C608973.1	30	2	
1622	tacgtccagc	7	TC16746	73	2	similar to GP|20977567|gb|AAM28211.1||AY099523 vacuolar ATP synthase 16 kDa proteolipid subunit {Danio rerio}, partial (89%)
1622	tacgtccagc	7	CD081462	80	1	similar to GP|10442628|gb|AAG17394.1|AF277150_1|AF277150 V-ATPase 16 kD proteolipid subunit c {Solenopsis invicta}, partial (78%)
1623	ttcgaaaata	7	AA143896	69	0	
1624	acaaccataa	7				no_annot
1625	aacagaaaaa	7	TC14116	93	0	weakly similar to PIR|T41173|T41173 phosphomannomutase homolog - fission yeast (Schizosaccharomyces pombe), partial (22%)
1626	tgaactctta	6				no_annot
1627	gcagtagttg	6	CD163199	73	0	
1628	gttggtttgg	6	TC14406	57	0	weakly similar to SP|O17488|TF2D_ARTSF Transcription initiation factor TFIID (TATA-box factor) (TATA sequence-binding protein) (TBP). [Brine shrimp, Artemia franciscana] {Artemia sanfranciscana}, partial (40%)
1629	tgtgtatgta	6				no_annot
1630	cgattaaaaa	6				no_annot
1631	gtcgattgga	6				no_annot
1632	gtaacagaaa	6	C201230.1	7	9	
1632	gtaacagaaa	6	C604669.1	10	6	
1633	tcattttatt	6	TC7258	61	0	
1633	tcattttatt	6	TC10776	73	2	weakly similar to GP|27374373|gb|AAO01111.1||AY190960 CG4686-PA {Drosophila pseudoobscura}, partial (29%)
1633	tcattttatt	6	TC10777	91	0	weakly similar to GP|27374373|gb|AAO01111.1||AY190960 CG4686-PA {Drosophila pseudoobscura}, partial (23%)
1634	ataagacgta	6	TC8578	83	0	

1635	gaaatcgatc	6	TC10780	76	1	similar to GP|24415014|emb|CAD54736.1||AJ512486 core alpha-6-fucosyltransferase {Caenorhabditis elegans}, partial (13%)
1636	ccactatctt	6	TC7342	83	0	
1636	ccactatctt	6	AI067442	85	0	
1637	gtttgtttca	6	TC11614	48	1	weakly similar to GP|16197488|dbj|BAB69947.1||AB060129 legumain {Bos taurus}, partial (15%)
1637	gtttgtttca	6	TC14076	82	2	weakly similar to PIR|JC7752|JC7752 F-LANa protein - human, partial (79%)
1638	atgtatcctg	6	CD091432	25	2	
1639	tgtttttctt	6	CD080900	82	0	
1640	atgataggat	6	TC12084	80	0	
1641	tgtggaaagg	6				no_annot
1642	gttatttaaa	6	C209652.1	91	0	
1643	tatgtgtgtg	6	TC12619	43	0	
1643	tatgtgtgtg	6	TC15221	50	1	
1644	ctgagatgta	6				no_annot
1645	ttctgcaaca	6	C609365.1	47	0	
1646	tcatatttac	6	CD123102	17	0	
1647	acgggttgtc	6				no_annot
1648	agcgagagga	6	TC7656	44	0	homologue to GP|12005493|gb|AAG44476.1|AF241788_1|AF241788 NPD011 {Homo sapiens}, partial (10%)
1649	acagactgag	6	TC15945	72	0	
1650	gttcgttcgg	6	TC7918	49	1	weakly similar to SP|Q99KK7|DPP3_MOUSE Dipeptidyl-peptidase III(DPP III) (Dipeptidyl aminopeptidase III) (Dipeptidyl arylamidase III). [Mouse] {Mus musculus}, partial (28%)
1651	gaaacggcgt	6	TC15750	11	0	similar to SP|Q99426|TBCB_HUMAN Tubulin-specific chaperone B (Tubulin folding cofactor B) (Cytoskeleton-associated protein CKAPI). [Human] {Homo sapiens}, partial (13%)
1652	gcgtatgaca	6	TC17597	91	0	similar to GP|16768840|gb|AAL28639.1||AY061091 LD07883p {Drosophila melanogaster}, partial (31%)
1653	ttgttctcca	6				no_annot

1654	tcggagttca	6	TC16814	77	0	homologue to GP|14588595|dbj|BAB61794.1||AB063181 calmodulin {Metridium senile}, partial (62%)
1655	aaaatttcac	6				no_annot
1656	catatttgaa	6				no_annot
1657	atcatccttc	6	AI739713	67	0	
1658	taataaacac	6	TC6868	96	0	similar to SP|Q22053|FBRL_CAEEL Fibrillarin. {Caenorhabditis elegans}, partial (78%)
1659	tgaacataga	6				no_annot
1660	gcatatgtac	6	AI977347	94	1	weakly similar to GP|24980965|gb|AAH39799.1||BC039799 Ccs protein {Mus musculus}, partial (8%)
1661	gatgccattt	6	TC13699	84	0	similar to PIR|T44596|T44596 26S proteasome regulatory complex chain p50 [imported] - fruit fly (Drosophila melanogaster), partial (86%)
1662	ttgatctgtg	6	AY747306|Sma.1227	97	0	Polo-like kinase
1663	tttctaaatt	6				no_annot
1664	atggatgagc	6	CD125326	68	1	
1665	taccaagtgg	6	TC18396	64	0	
1666	cgccggagca	6				no_annot
1667	taattggtta	6				no_annot
1668	cattcatagt	6	TC7468	60	2	weakly similar to GP|16974629|gb|AAL31217.1||AY060592 At1g72730/F28P22_8 {Arabidopsis thaliana}, partial (78%)
1669	cggtggttct	6	TC7342	81	1	
1669	cggtggttct	6	AI067442	79	1	
1670	tatagcgttc	6	CD134159	10	1	
1671	atagttgaat	6	AI559056	84	0	
1672	gtcaggtttt	6	TC7417	87	0	weakly similar to GP|5114446|gb|AAD40318.1|AF158370_1|AF158370 DEAD-box RNA helicase {Gallus gallus}, partial (55%)
1673	gcgatggttt	6	TC17463	75	0	similar to GP|16182903|gb|AAL13593.1||AY058364 GH13672p {Drosophila melanogaster}, partial (17%)
1674	aagaagtgga	6	C607597.1	85	1	
1675	ttctagtagt	6				no_annot

1676	gatttgtact	6	TC7688	1	4	similar to PIR|B46619|B46619 Ca2+/calmodulin-dependent protein kinaseII gamma chain, splice form B - human, partial (30%)
1677	ctatggagga	6				no_annot
1678	cggcaacctt	6				no_annot
1679	tatcactcaa	6				no_annot
1680	caaacgtacg	6	TC10554	95	0	similar to GP|8099346|gb|AAF72103.1|AF154845_1|AF154845 MARK {Homo sapiens}, partial (13%)
1681	tatttgttta	6	TC6877	76	0	
1682	ttttttctgg	6	C608957.1	98	0	
1683	gattaaacgt	6				no_annot
1684	gctcaatgga	6				no_annot
1685	acgtattagt	6				no_annot
1686	tcaggttgtg	6				no_annot
1687	gcgcaggcgt	6				no_annot
1688	gtatcgacca	6	TC11287	28	0	weakly similar to GP|4836700|gb|AAD30527.1||AF132794 anaphase promoting complex subunit 10 {Homo sapiens}, partial (79%)
1689	gataacactg	6				no_annot
1690	gtggagattg	6				no_annot
1691	tgtatcttca	6	TC17950	60	0	weakly similar to GP|15636687|gb|AAL02139.1||AY043319 nucleolar protein family A member 2 {Branchiostoma belcheri}, partial (35%)
1692	caataaaaga	6				no_annot
1693	ctgttttatt	6	Z32531|Sma.24	73	0	Cathepsin C
1694	ttaacgctat	6				no_annot
1695	aagttacaga	6	TC13980	89	0	weakly similar to GP|16741131|gb|AAH16418.1||BC016418 expressed sequence AA960436 {Mus musculus}, partial (10%)
1695	aagttacaga	6	TC13981	86	0	
1696	catcaaatta	6				no_annot

1697	taatgaatgc	6	TC15069	63	0	similar to SP|P32321|DCTD_HUMAN Deoxycytidylate deaminase(dCMP deaminase). [Human] {Homo sapiens}, partial (62%)
1698	cttgtaaact	6				no_annot
1699	acttggaggt	6	TC10951	54	0	
1699	acttggaggt	6	TC10953	21	2	
1699	acttggaggt	6	TC10953	21	0	
1699	acttggaggt	6	CD083982	93	0	
1700	acaaaacttt	6	C602137.1	0	6	
1701	gtcgaatgct	6	Z29960|Sma.1164	5	3	Tandem repeat
1701	gtcgaatgct	6	Z29960|Sma.1164	5	1	Tandem repeat
1702	cagtgtgcca	6				no_annot
1703	gttttaccag	6	TC8383	59	0	
1704	atgatggtgg	6				no_annot
1705	ccagttgcca	6	C207043.1	77	1	
1705	ccagttgcca	6	C600958.1	72	1	
1706	gggaattgtt	6				no_annot
1707	attacgtgaa	6	TC11185	33	0	
1708	caagttattc	6	TC9061	95	0	weakly similar to GP|15291809|gb|AAK93173.1||AY051749 LD27988p {Drosophila melanogaster}, partial (4%)
1709	tcaatatttg	6	TC17003	79	1	similar to SP|Q24799|MYPH_ECHGR Myophilin. {Echinococcus granulosus}, complete
1709	tcaatatttg	6	CD184805	45	0	
1710	tgcaatggaa	6	TC12554	7	2	
1711	ttgttgatca	6				no_annot
1712	atagcttctt	6	TC15917	73	0	
1713	tgttatacta	6	TC8285	75	0	
1714	caacgatcct	6	TC11026	86	1	similar to SP|P54578|UBPE_HUMAN Ubiquitin carboxyl-terminal hydrolase 14(Ubiquitin thiolesterase 14) (Ubiquitin-specific processing protease 14) (Deubiquitinating enzyme 14). [Human] {Homo sapiens}, partial (28%)
1715	ttgtcagttc	6				no_annot

1716	cttgattaca	6	TC15971	22	2	
1716	cttgattaca	6	TC16625	84	2	similar to GP|16182307|gb|AAL13472.1||AY058243 GH01077p {Drosophila melanogaster}, partial (62%)
1716	cttgattaca	6	N20714	5	0	
1717	ggattctatc	6	AY371484|Sma.793	91	0	Smad4 (Smad4)
1718	gatttttgtt	6	TC11268	95	0	weakly similar to GP|28628069|gb|AAO38689.1||AY167035 long-chain acyl-CoA synthetase {Mus musculus}, partial (19%)
1719	cagcttgtgt	6	CD192992	65	0	weakly similar to SP|O14735|PIS_HUMAN CDP-diacylglycerol--inositol 3-phosphatidyltransferase(Phosphatidylinositol synthase) (PtdIns synthase) (PI synthase). [Human] {Homo sapiens}, partial (47%)
1720	ttggatacta	6	TC11417	53	0	weakly similar to SP|Q9CXL1|SMP1_MOUSE Small membrane protein 1. [Mouse] {Mus musculus}, partial (29%)
1721	gattgaatat	6	TC12782	29	2	weakly similar to PIR|T42719|T42719 TPR-containing/SH2-binding phosphoprotein - mouse, partial (14%)
1721	gattgaatat	6	TC14238	98	0	
1721	gattgaatat	6	TC17990	6	1	weakly similar to GP|17940120|gb|AAL49497.1|AF408420_1|AF408420 beta-catenin {Chaetopterus variopedatus}, partial (6%)
1722	tttggtttac	6				no_annot
1723	tgttaatgct	6	C602228.1	97	0	
1724	aatattttac	6	TC6907	93	0	weakly similar to GP|28852477|gb|AAO55550.1||AE016863 glutathione S-transferase family protein {Pseudomonas syringae pv. tomato str. DC3000}, partial (20%)
1725	tgtacaacta	6	TC10949	97	0	weakly similar to GP|476274|gb|AAA17871.1||U08191 R kappa B {Homo sapiens}, partial (5%)
1726	tttcgatgta	6	TC19652	20	2	
1727	ccaacgacca	6	AA958304	40	0	
1728	tacttcacag	6	C200970.1	26	2	
1728	tacttcacag	6	C603795.1	84	0	
1729	acctaattaa	6				no_annot
1730	tccacgtata	6				no_annot

1731	ttcaaaggct	6				no_annot
1732	gcaatgtata	6	TC13062	10	0	
1733	agggtatgtg	6	C610619.1	3	5	
1734	aaacgttaaa	6				no_annot
1735	acaggtagac	6	C315456.1	7	2	
1736	catcagatat	6				no_annot
1737	cccaaaagga	6	TC13682	93	0	similar to GP|3851612|gb|AAC72372.1||AF095937 succinate dehydrogenase Ip subunit {Gallus gallus}, partial (84%)
1737	cccaaaagga	6	TC13683	67	0	similar to GP|3851612|gb|AAC72372.1||AF095937 succinate dehydrogenase Ip subunit {Gallus gallus}, partial (64%)
1737	cccaaaagga	6	CD077640	52	0	similar to GP|3851612|gb|AAC72372.1||AF095937 succinate dehydrogenase Ip subunit {Gallus gallus}, partial (18%)
1738	tattgcatag	6	C302022.1	86	0	
1738	tattgcatag	6	C711608.1	86	0	
1739	agggtttcta	6	AY456264|Sma.1225	94	0	Ftz-F1 interacting protein
1740	aacaatggaa	6	TC17725	44	0	
1740	aacaatggaa	6	CD117460	15	0	
1741	tataactttg	6	CD117059	32	1	
1742	tcagatgtga	6	TC11080	61	0	weakly similar to PIR|AI2264|AI2264 aldehyde dehydrogenase [imported] - Nostoc sp. (strain PCC 7120), partial (33%)
1743	agtgtgataa	6				no_annot
1744	aaccgtctcc	6				no_annot
1745	aatgattcta	6				no_annot
1746	tattgtatga	6	TC10376	93	1	similar to GP|22832080|gb|AAF48002.2||AE003485 CG2522-PA {Drosophila melanogaster}, partial (40%)
1747	taaattgact	6	TC11484	84	2	weakly similar to GP|24347445|gb|AAN54706.1|AE015611_4|AE015611 Snf2 family protein {Shewanella oneidensis MR-1}, partial (3%)
1748	tggatagaag	6	TC17421	72	1	weakly similar to GP|13625463|gb|AAK35066.1|AF353992_1|AF353992 BBP-like protein 2 {Homo sapiens}, partial (22%)

1749	ttaccaggta	6	TC10642	94	0	weakly similar to SP|Q9XZL8|SRA_DROME Sarah protein (Nebula protein). [Fruit fly] {Drosophila melanogaster}, partial (23%)
1750	aactttttct	6				no_annot
1751	tttattgttg	6	TC17123	97	0	weakly similar to GP|23172400|gb|AAN14096.1||AE003758 CG6323-PB {Drosophila melanogaster}, partial (39%)
1752	taatgtctga	6	TC9461	67	0	
1753	tgtactctca	6	TC7808	85	0	weakly similar to SP|O95336|6PGL_HUMAN 6-phosphogluconolactonase(6PGL). [Human] {Homo sapiens}, partial (29%)
1754	gttactatca	6	C207959.1	98	0	
1755	tcataatcct	6	TC7712	30	2	
1756	tcaatatctt	6	TC11009	47	2	
1756	tcaatatctt	6	TC11783	81	0	
1756	tcaatatctt	6	CD084208	39	0	
1757	ccaatgttcc	6				no_annot
1758	tatgtacttt	6				no_annot
1759	aaggcatatt	6	C603408.1	22	0	
1760	tacattaggc	6				no_annot
1761	actaatctgg	6	TC13697	78	1	weakly similar to GP|15418966|gb|AAK83461.1||AY039235 annexin 4 {Xenopus laevis}, partial (63%)
1762	ctaaaaaaaa	6				no_annot
1763	aaacaccaag	6				no_annot
1764	tatattcgtt	6				no_annot
1765	tttcgcaagt	6	C611030.1	2	0	
1766	ttcgcacgct	6	TC17368	29	0	
1767	ttacggtaat	6				no_annot
1768	ttgggtattt	6				no_annot
1769	tttccctcct	6				no_annot
1770	tacttgaggt	6				no_annot

1771	gacatcaaat	6	TC9251	54	0	weakly similar to GP|9437347|gb|AAF87318.1|AF168716_1|AF168716 x 007 protein {Homo sapiens}, partial (96%)
1771	gacatcaaat	6	TC16049	36	1	similar to GP|9437347|gb|AAF87318.1|AF168716_1|AF168716 x 007 protein {Homo sapiens}, partial (75%)
1771	gacatcaaat	6	CD084210	60	0	similar to GP|9437347|gb|AAF87318.1|AF168716_1|AF168716 x 007 protein {Homo sapiens}, partial (84%)
1772	tttgaaaatc	6	TC11392	91	0	
1772	tttgaaaatc	6	TC14216	10	3	
1773	tggagatcct	6	TC11707	16	0	
1774	ctgtatggta	6	TC8713	60	0	similar to GP|21113727|gb|AAM41834.1||AE012367 integral membrane protein {Xanthomonas campestris pv. campestris str. ATCC 33913}, partial (5%)
1775	aacactatat	6				no_annot
1776	tattccttgg	6				no_annot
1777	tgcaaaaaaa	6	C200511.1	20	1	
1777	tgcaaaaaaa	6	C609391.1	50	0	
1778	ctagatatgt	6				no_annot
1779	aacgctgttt	6				no_annot
1780	attagagtga	6	C202173.1	12	4	
1780	attagagtga	6	C611269.1	6	7	
1781	gttatctgta	6	TC17831	8	0	
1782	gtaacatata	6	TC9754	52	2	weakly similar to GP|1134884|emb|CAA62607.1||X91200 ligand gated ATP receptor {Rattus norvegicus}, partial (12%)
1783	aaagcttatg	6	TC10915	89	1	similar to GP|1923250|gb|AAC47498.1||U85805 synaptobrevin homolog {Hirudo medicinalis}, partial (25%)
1784	gcgaagctat	6	TC14193	66	0	
1785	actgtaatgt	6	TC6880	49	5	similar to GP|17944974|gb|AAL48550.1||AY070928 RE03018p {Drosophila melanogaster}, partial (3%)
1785	actgtaatgt	6	TC7059	53	0	
1785	actgtaatgt	6	TC7209	77	0	

1785	actgtaatgt	6	TC7243	32	2	similar to PIR|T39903|T39903 serine-rich protein - fission yeast (Schizosaccharomyces pombe), partial (4%)
1785	actgtaatgt	6	CD148646	0	1	
1785	actgtaatgt	6	CD098270	35	0	
1785	actgtaatgt	6	CD115179	55	1	
1786	cccatacggg	6				no_annot
1787	gtggttttgg	6	TC17594	89	0	weakly similar to SP|P59517|SYN_BUCBP Asparaginyl-tRNA synthetase(Asparagine--tRNA ligase) (AsnRS). [subsp. Baizongia pistaciae] {Buchnera aphidicola}, partial (19%)
1788	ttcccggttt	6	C203869.1	80	0	
1788	ttcccggttt	6	C612479.1	80	0	
1789	ataaatctga	6	TC8784	11	9	
1789	ataaatctga	6	CD146742	79	2	
1790	gttaaaaaca	6	TC10504	44	2	
1791	tatcaaggca	6	AA528910	14	1	similar to GP|3641252|gb|A leucine-rich receptor-like protein kinase {Malus x domestica}, partial (1%)
1792	cacaagaaga	6	TC8050	17	1	homologue to GP|6434950|gb|AAF08387.1|AF145306_1|AF145306 26S proteasome regulatory complex subunit p48A {Drosophila melanogaster}, partial (92%)
1792	cacaagaaga	6	TC11034	17	1	
1793	ttggtgaaac	6	C605469.1	21	3	
1794	acgtcgagag	6				no_annot
1795	tttctacatt	6	TC15255	69	0	
1795	tttctacatt	6	TC15369	13	3	weakly similar to GP|12002284|gb|AAG43278.1|AF135156_1|AF135156 D-cadherin precursor {Homo sapiens}, partial (10%)
1795	tttctacatt	6	AA514121	27	0	
1796	aaatgcctgt	6	TC13846	65	0	weakly similar to GP|27466905|gb|AAO12859.1||AY172026 high mobility group protein {Branchiostoma belcheri tsingtaunese}, partial (32%)
1797	aaatagcgag	6				no_annot
1798	ataggaaact	6				no_annot
1799	caagcaaatg	6				no_annot

1800	atattatgag	6				no_annot
1801	ttgttgaacg	6				no_annot
1802	cactcacggt	6				no_annot
1803	caattcaagg	6	TC12687	77	0	
1804	ctgttgaaaa	6	TC8583	71	0	
1804	ctgttgaaaa	6	TC15179	61	2	
1805	aagagatcgc	6	TC8348	72	0	
1806	tgtattgtta	6	TC10131	78	0	weakly similar to SP|P49580|ACHE_XENLA Acetylcholine receptor protein, epsilon chain precursor. [African clawed frog] {Xenopus laevis}, partial (7%)
1806	tgtattgtta	6	TC17731	76	1	
1806	tgtattgtta	6	BF936608	77	0	
1807	gcgtagagaa	6	TC7544	86	0	similar to GP|28806046|dbj|BAC59322.1||AP005076 TolA protein {Vibrio parahaemolyticus}, partial (9%)
1808	acgatgtaaa	6				no_annot
1809	ccctagggag	6				no_annot
1810	atcatctttt	6				no_annot
1811	gaagtgaatc	6				no_annot
1812	gtgcagctgc	6				no_annot
1813	tggtgttggg	6				no_annot
1814	tttcaactca	6	TC15652	56	2	
1815	tatccttcaa	6				no_annot
1816	ttgatgtttt	6				no_annot
1817	attgctttat	6	TC13558	91	0	weakly similar to GP|7291892|gb|AAF47311.1||AE003465 CG15792-PA {Drosophila melanogaster}, partial (29%)
1818	tgaaaaaaac	6	TC9161	68	0	weakly similar to GP|15099955|gb|AAK84177.1|AF384162_1|AF384162 diacylglycerol acyltransferase 2-like protein {Mus musculus}, partial (23%)
1819	tgaaaaaaaa	6	C204803.1	62	2	
1820	ataagcaatg	6				no_annot

1821	gagacttgtt	6	TC7668	54	1	weakly similar to SP|P78697|DIM1_KLULA Dimethyladenosine transferase(S-adenosylmethionine-6-N', N'-adenosyl(rRNA) dimethyltransferase) (18S rRNA dimethylase). [Yeast] {Kluyveromyces lactis}, partial (42%)
1822	ttttcttcag	6				no_annot
1823	ttaaatgaag	6	TC14367	89	0	weakly similar to GP|22946910|gb|AAN11076.1||AE003666 CG31683-PA {Drosophila melanogaster}, partial (13%)
1824	ctaggtcaag	6	TC13642	42	0	weakly similar to GP|18307966|gb|AAL67778.1|AF459094_1|AF459094 splicing factor, arginine/serine-rich 12 {Homo sapiens}, partial (12%)
1825	tttgcaattc	6				no_annot
1826	gagtagtatt	6	TC18090	25	0	similar to GP|18447570|gb|AAL68346.1||AY075539 RH18819p {Drosophila melanogaster}, partial (25%)
1827	tatgtttgta	6	AF130788_1_755	46	1	[CDS] Schistosoma mansoni cytochrome b (Cytb) gene, partial cds
1828	taaagttaca	6	TC17319	91	2	weakly similar to PIR|T11646|T11646 tRNA nucleotidyltransferase homolog - fission yeast (Schizosaccharomyces pombe), partial (17%)
1829	tgaattagag	6	TC7757	87	0	weakly similar to GP|3876769|emb|CAA93469.1||Z69637 C. elegans PHY-2 protein (corresponding sequence F35G2.4) {Caenorhabditis elegans}, partial (29%)
1830	ttatttgtgt	6	C204050.1	90	0	
1830	ttatttgtgt	6	C609632.1	91	0	
1831	tttaataaaa	6	TC13729	97	0	weakly similar to GP|1870696|gb|AAB48831.1||U84114 cleavage stage histone H2A {Psammechinus miliaris}, partial (87%)
1832	aaacaagata	6				no_annot
1833	catatatatg	6				no_annot
1834	aaaatcagtt	6	TC11007	93	0	similar to GP|25989490|gb|AAM18788.1||AY092764 pontin {Danio rerio}, partial (92%)
1835	caaatacatt	6	TC8421	73	0	
1836	ctatttctta	6	TC7523	97	0	weakly similar to GP|20151659|gb|AAM11189.1||AY094836 LD44221p {Drosophila melanogaster}, partial (67%)
1836	ctatttctta	6	TC16650	21	4	

1837	ttgattcttc	6				no_annot
1838	acaaagcaca	6	TC14000	53	0	
1839	gctccgtgtt	6	TC15290	93	0	weakly similar to GP|21627166|gb|AAF58177.2||AE003812 CG10261-PA {Drosophila melanogaster}, partial (33%)
1840	gtctggtgct	6				no_annot
1841	aacattattt	6	TC7850	90	0	similar to GP|12641925|gb|AAK00053.1||AF134398 actin-filament fragmenting protein {Echinococcus granulosus}, partial (36%)
1841	aacattattt	6	TC11056	51	0	
1842	gcccgcatct	6	TC12367	34	0	
1843	ggctctttag	6	TC8347	59	0	similar to GP|19909174|gb|AAM03145.1|AF489958_1|AF489958 reinfection related protein 338 {Schistosoma japonicum}, partial (97%)
1844	taatcagtta	6				no_annot
1845	tagttccagt	6	TC17245	6	8	
1846	gagaaaacag	6	C200147.1	12	3	
1846	gagaaaacag	6	C601338.1	11	3	
1847	ggaacataca	6	TC6944	93	0	weakly similar to GP|20372965|dbj|BAB91223.1||AB066105 aquaporin 10 {Homo sapiens}, partial (15%)
1848	gggtttttgc	6	TC13808	85	0	weakly similar to GP|10727837|gb|AAF49190.2||AE003518 CG11577-PA {Drosophila melanogaster}, partial (26%)
1849	tagattgttt	6				no_annot
1850	ttggcaagtc	6	TC8515	81	0	weakly similar to GP|16182333|gb|AAL13477.1||AY058248 GH01229p {Drosophila melanogaster}, partial (17%)
1851	atataacagc	6				no_annot
1852	cttgctcgtt	6				no_annot
1853	atttccacac	6	TC14948	82	0	weakly similar to GP|10441474|gb|AAG17060.1|AF188891_1|AF188891 zinc finger protein {Drosophila melanogaster}, partial (13%)
1854	gcgtatgtaa	6	TC12170	27	0	weakly similar to PIR|S78727|S78727 protein YLL018c-a - yeast (Saccharomyces cerevisiae), partial (27%)

1855	gcttagaata	6				no_annot
1856	ctcatttata	6				no_annot
1857	ttacgtttgc	6				no_annot
1858	gacaaaatga	6				no_annot
1859	cattcaatgc	6	TC7999	92	0	similar to GP|21430790|gb|AAM51073.1||AY119213 SD15934p {Drosophila melanogaster}, complete
1860	aggcgtatat	6	TC17947	75	0	
1861	ttgtcaaggt	6				no_annot
1862	ttgtttagct	6	TC19144	95	0	
1863	ctcctggttc	6				no_annot
1864	ctctacgttc	6	TC14417	35	1	similar to SP|P51966|UBC7_HUMAN Ubiquitin-conjugating enzyme E2-18 kDa UbcH7(Ubiquitin- protein ligase) (Ubiquitin carrier protein) (UbcM4) (E2-F1) (L-UBC). [Mouse] {Mus musculus}, partial (84%)
1864	ctctacgttc	6	TC18234	9	1	
1865	ttatactata	6	CD079892	96	0	
1866	agaccataga	6				no_annot
1867	tcttgtttac	6	TC16812	63	0	homologue to GP|14588595|dbj|BAB61794.1||AB063181 calmodulin {Metridium senile}, complete
1867	tcttgtttac	6	TC16816	54	0	EGAD|125688|134067 calmodulin-like protein {Branchiostoma lanceolatum}, partial (48%)
1867	tcttgtttac	6	AI478025	24	0	
1868	ttactagtcg	6				no_annot
1869	taggcggatg	6				no_annot
1870	tcactcaatt	6				no_annot
1871	gagttgcact	6	AY675348|Sma.5393	83	0	Tyrosinase 2 precursor (TYR2)
1872	agaaccacgc	6				no_annot
1873	ggtggacttt	6	TC7590	50	0	weakly similar to SP|O35723|DJB3_MOUSE DnaJ homolog subfamily B member 3 (DnaJ protein homolog 3) (Heat shock J3 protein) (HSJ-3) (MSJ-1). [Mouse] {Mus musculus}, partial (50%)
1874	gaacgtaaca	6	AY323529|Sma.76	9	6	ATP-diphosphohydrolase 1 (ATPDase1)
1875	tgaagaacat	6				no_annot

1876	acttgtagtt	6	C200524.1	70	0	
1876	acttgtagtt	6	C602454.1	48	0	
1877	cgcttgactc	6	TC11073	81	0	weakly similar to GP|984509|gb|AAA91778.1||U07802 Tis11d {Homo sapiens}, partial (7%)
1878	taatatacac	6	TC13776	92	0	weakly similar to GP|262250|gb|AAB24621.1||S52010 1st Met is at position 21 {Mus sp.}, partial (22%)
1879	tatgctaaac	6	TC6872	79	2	similar to PIR|A60671|A60671 tubulin alpha chain - sea urchin (Paracentrotus lividus), partial (98%)
1880	tgtttcatca	6	TC16999	75	3	
1880	tgtttcatca	6	TC18970	74	0	
1881	ggtattattt	6	TC9370	76	0	
1882	tactattatg	6				no_annot
1883	cttgaagcca	6	C608739.1	43	0	
1884	gtgctgttgc	6	TC7501	39	0	
1885	gtatgttaca	6				no_annot
1886	ctactactga	6	CD065340	81	0	
1887	taaaacagag	6	TC17646	14	1	homologue to PIR|A26480|A26480 knob protein - malaria parasite (Plasmodium falciparum) (fragments), partial (5%)
1888	gtttgctgaa	6				no_annot
1889	tacattggtt	6	TC18726	96	0	weakly similar to SP|Q8NSJ4|SYW_CORGL Tryptophanyl-tRNA synthetase(Tryptophan--tRNA ligase) (TrpRS). [Brevibacterium flavum] {Corynebacterium glutamicum}, partial (31%)
1890	atgcaccctg	6	TC10828	85	0	weakly similar to GP|3550456|emb|CAA06329.1||AJ005073 Alix {Mus musculus}, partial (4%)
1891	ggaaaaagct	6	CD087440	8	0	
1892	acgcttgtat	6				no_annot
1893	catacagact	6	TC9727	42	0	
1894	caagtgtcga	6	TC14024	77	1	similar to GP|16226747|gb|AAL16250.1|AF428320_1|AF428320 AT5g05080/MUG13_6 {Arabidopsis thaliana}, partial (32%)

1895	gtatagaaga	6	TC8036	76	1	similar to SP|O75251|NUKM_HUMAN NADH-ubiquinone oxidoreductase 20 kDa subunit, mitochondrial precursor(Complex I-20KD) (CI-20KD) (PSST subunit). [Human] {Homo sapiens}, partial (75%)
1896	acatccaagc	6	TC13828	62	0	homologue to GP|15529624|gb|AAL01375.1|AF406557_1|AF406557 PTPRE {Homo sapiens}, partial (8%)
1897	cacattaaaa	6				no_annot
1898	tggaggcttg	6	TC17136	83	0	weakly similar to PIR|S57873|S57873 pendulin - mouse, partial (31%)
1899	tgtgcgtgcg	6				no_annot
1900	catacacata	6	AI395291	10	2	weakly similar to SP|P58249|PHS_DROVI Pterin-4-alpha-carbinolamine dehydratase(PHS) (4-alpha-hydroxy-tetrahydropterin dehydratase) (Pterin carbinolamine dehydratase) (PCD). [Fruit fly] {Drosophila virilis}, partial (49%)
1901	accgtttctt	6	C602341.1	86	0	
1902	agtgaataga	6				no_annot
1903	attggtgtgt	6	C313577.1	97	0	
1904	acgtgactat	6				no_annot
1905	ggattgctga	6	C211130.1	97	0	
1905	ggattgctga	6	C601198.1	87	0	
1906	tatgtctcaa	6	TC7593	4	2	
1907	caaatcaatg	6				no_annot
1908	tagtacccca	6	AA528917	29	2	similar to PIR|S44151|S44151 cathepsin L- fluke (Schistosoma mansoni), partial (16%)
1909	ttattgaacg	6				no_annot
1910	taaatgagtc	6				no_annot
1911	agttctactg	6				no_annot
1912	gggtggtcaa	6	TC8817	68	0	
1913	agttaacgtg	6				no_annot
1914	tgtattgacc	6	TC8229	32	0	similar to SP|Q00380|A2S1_MOUSE Clathrin coat assembly protein AP17 (Clathrin coat associated protein AP17) (Plasma membrane adaptor AP-2 17 kDa protein) (HA2 17 kDa subunit) (Clathrin assembly protein 2 small chain). [Rat] {Rattus norvegicus}, complete

1915	gaaaagtgag	6				no_annot
1916	ttgatgtaga	5	C209733.1	34	2	
1916	ttgatgtaga	5	C604677.1	34	2	
1917	tttaatcctt	5				no_annot
1918	tattagctca	5	TC18133	80	0	
1919	aattcgagtt	5	TC12512	92	0	
1920	cggcaagtgg	5				no_annot
1921	gtttgcttaa	5	TC18383	94	0	
1922	ggattttgga	5	AI977095	60	2	GP|15291599|gb|AAK93068.1||AY051644 GM14238p {Drosophila melanogaster}, partial (7%)
1923	tgtgtatgtg	5				no_annot
1924	taggttatgg	5				no_annot
1925	taaatttatg	5	TC18176	92	0	similar to GP|3876636|emb|CAB04256.1||Z81525 C. elegans LET-858 protein (corresponding sequence F33A8.1) {Caenorhabditis elegans}, partial (3%)
1925	taaatttatg	5	CD127797	15	0	
1926	tttttctaag	5				no_annot
1927	tttttctaaa	5	CD169645	91	0	
1928	cttgttgggc	5	TC7867	86	0	weakly similar to GP|17944258|gb|AAL48023.1||AY070552 LD28127p {Drosophila melanogaster}, partial (38%)
1929	atgtaataat	5	C611351.1	78	0	
1930	tcgtgttttg	5				no_annot
1931	ttttaagact	5				no_annot
1932	taggcaatat	5	C203472.1	89	0	
1933	tggctacagg	5	TC17669	29	0	
1934	catatcagca	5	C607143.1	86	0	
1935	tgcgtactgg	5				no_annot
1936	taaacaaaac	5	TC17427	72	0	
1936	taaacaaaac	5	TC18953	77	0	similar to SP|P19338|NUCL_HUMAN Nucleolin (Protein C23). [Human] {Homo sapiens}, partial (3%)
1937	ataatcaata	5	CD198709	44	1	similar to GP|16267034|dbj NADH dehydrogenase subunit 2 {Crenimugil crenilabis}, partial (3%)

1938	gtggagcagt	5	TC18625	40	2	similar to GP|10039641|gb|AAG12204.1|AF287482_5|AF287482 Orf122 {Chlorobium tepidum}, partial (23%)
1939	gagacaatgt	5				no_annot
1940	gagaccggta	5	CD079711	68	0	
1941	gttgtcgaat	5				no_annot
1942	tatatttgtg	5				no_annot
1943	gacgagctgg	5	AF014465|Sma.6	18	0	Pad1 homolog (SmPOH)
1944	tgcgttcatt	5				no_annot
1945	tggattgccg	5				no_annot
1946	tttcaatgtt	5	TC10857	77	0	
1947	gatatatttt	5	CD138667	95	0	similar to PIR|AC2091|AC2 serine/threonine kinase with two-component sensor domain all2282 [imported] - Nostoc sp., partial (1%)
1948	agtagtatca	5				no_annot
1949	gagcaatgaa	5				no_annot
1950	aaagtgacgc	5				no_annot
1951	ttgagaagct	5	TC12001	2	0	weakly similar to GP|17862438|gb|AAL39696.1||AY069551 LD27581p {Drosophila melanogaster}, partial (21%)
1952	acttagttgt	5				no_annot
1953	ctagtcagtg	5	TC11835	43	0	weakly similar to PIR|JC7861|JC7861 caspase-associated recruitment domain(CARD)-containing pro-apoptotic protein, CARP - Human, partial (18%)
1954	gggtctgctg	5	TC17944	54	1	weakly similar to GP|5689|emb|CAA26480.1||X02633 ribosomal protein eL12' (clone pD3D13) (aa 1-107) {Artemia sp.}, partial (31%)
1955	agcgagagat	5				no_annot
1956	ctacgattat	5	C201137.1	17	2	
1956	ctacgattat	5	C611360.1	22	2	
1957	tatgtttatt	5	TC8591	87	0	
1957	tatgtttatt	5	AA559548	89	0	
1957	tatgtttatt	5	BG932345	76	0	
1958	gcagatgtgt	5				no_annot

1959	tgtgtaattg	5	TC14880	31	0	similar to GP|21627394|gb|AAM68685.1||AE003822 CG30051-PB {Drosophila melanogaster}, partial (14%)
1959	tgtgtaattg	5	CD153242	46	0	
1960	tgtgtaatta	5	C201394.1	22	3	
1960	tgtgtaatta	5	C610914.1	20	5	
1961	tatgaggcaa	5				no_annot
1962	gagtcgaatt	5				no_annot
1963	tttaggatcg	5				no_annot
1964	cttgaggatt	5	TC8146	84	0	weakly similar to GP|11191800|emb|CAC16398.1||AJ276505 Cysteinyl-tRNA-synthetase {Mus musculus domesticus}, partial (6%)
1965	tagatgtaca	5	TC7803	78	2	
1965	tagatgtaca	5	TC16159	93	0	
1966	tattacatca	5	TC10533	92	1	weakly similar to GP|1914794|emb|CAA72813.1||Y12106 aminopeptidase {Lumbricus rubellus}, partial (76%)
1967	gtttaaacgg	5				no_annot
1968	gccaaatgat	5	C202818.1	81	0	
1969	gaggagcata	5	TC9721	46	1	
1969	gaggagcata	5	TC16372	92	0	
1969	gaggagcata	5	CD079688	23	1	similar to SP|P45700|M1A1_MOUSE Mannosyl-oligosaccharide 1,2-alpha-mannosidase IA(Processing alpha-1,2-mannosidase IA) (Alpha-1,2-mannosidase IA) (Mannosidase alpha class 1A member 1) (Man(9)-alpha-mannosidase). [Mouse] {Mus musculus}, partial (3%)
1969	gaggagcata	5	CD131160	80	0	
1969	gaggagcata	5	CD131638	10	1	
1969	gaggagcata	5	CD201406	36	4	similar to GP|13177306|gb|AAK14450.1|AF204951_24|AF204951 EsV-1-24 {Ectocarpus siliculosus virus}, partial (4%)
1969	gaggagcata	5	CD062627	75	0	
1969	gaggagcata	5	CD089301	73	0	
1969	gaggagcata	5	CD167157	50	1	
1970	agtaccattc	5	TC17035	31	0	

1971	ttggactgat	5	CD064558	83	0	similar to GP|5679293|emb|CAB51772.1||Z97187 swiss cheese protein {Drosophila melanogaster}, partial (4%)
1972	ggttacaaga	5	TC17417	86	0	weakly similar to GP|7292846|gb|AAF48239.1||AE003491 CG2453-PA {Drosophila melanogaster}, partial (39%)
1973	tttacttatt	5				no_annot
1974	gagtttgtag	5				no_annot
1975	tattacttat	5				no_annot
1976	aatacacagt	5	TC7658	17	2	
1977	gtgttaggtg	5				no_annot
1978	actcgaatgg	5	C611061.1	89	0	
1979	cacataaatc	5	TC10966	84	0	weakly similar to SP|Q13148|TDBP_HUMAN TAR DNA-binding protein-43 (TDP-43). [Human] {Homo sapiens}, partial (35%)
1980	ttagccacac	5	C610110.1	88	0	
1981	tatagttaag	5	TC7118	97	0	weakly similar to GP|28274854|gb|AAO25692.1||AY195856 ankyrin repeat protein E4_8 {synthetic construct}, partial (28%)
1982	tttctaaatc	5				no_annot
1983	cattcaaaac	5	C301278.1	87	0	
1984	aggttagcat	5				no_annot
1985	agtgtcacta	5	TC12870	63	0	
1986	tgtgacatcg	5	TC8058	93	0	similar to GP|25013101|gb|AAN71648.1||BT001876 SD10626p {Drosophila melanogaster}, partial (6%)
1987	cctcctggca	5	TC13511	0	2	homologue to GP|3063620|gb|AAC14119.1||AF056330 AUT1 {Schistosoma mansoni}, partial (47%)
1988	ttattttgtg	5				no_annot
1989	gatgacgtcc	5	CD136940	86	0	
1990	aaaaaggctg	5				no_annot
1991	agaaggaccc	5	TC13702	19	1	weakly similar to GP|10178878|emb|CAC08449.1||AJ294707 eukaryote initiation factor 2 beta {Gallus gallus}, partial (14%)
1991	agaaggaccc	5	TC13703	21	1	
1992	taatgatggg	5				no_annot
1993	tcatcagtca	5				no_annot

1994	gagatatgtt	5				no_annot
1995	cctttcttcg	5				no_annot
1996	caccagtagg	5	C316774.1	84	0	
1997	tgtatcattc	5	TC19064	75	0	weakly similar to GP|2288903|emb|CAA04368.1||AJ000879 mtAcyl carrier subunit isoform 1 {Drosophila melanogaster}, partial (43%)
1998	ttttctgatt	5	TC15691	38	0	
1999	gccacggtgg	5	C606664.1	40	0	
2000	ttatgattga	5	TC11740	20	4	weakly similar to GP|13661819|gb|AAK38111.1|AF360549_1|AF360549 BRCA1-binding helicase-like protein BACH1 {Homo sapiens}, partial (10%)
2001	cacaccagtc	5				no_annot
2002	agcgttgatt	5	TC14004	86	0	similar to SP|Q9VTF9|UFD1_DROME Ubiquitin fusion degradation protein 1 homolog (UB fusion protein 1). [Fruit fly] {Drosophila melanogaster}, partial (52%)
2002	agcgttgatt	5	TC14005	42	0	similar to GP|11037254|gb|AAG27535.1|AF234601_1|AF234601 UFD1 {Rattus norvegicus}, partial (46%)
2003	gacttctagg	5	CD079214	50	0	weakly similar to GP|7293589|gb|AAF48961.1||AE003512 CG14199-PA {Drosophila melanogaster}, complete
2004	taattgcttt	5	TC11099	89	0	similar to SP|P24155|MEPD_RAT Thimet oligopeptidase(Endo-oligopeptidase A) (Endopeptidase 24.15) (PZ-peptidase) (Soluble metallo-endopeptidase). [Rat] {Rattus norvegicus}, partial (25%)
2005	gatgatgtaa	5	C201468.1	15	3	
2006	ccaatctctt	5	CD130580	87	1	homologue to SP|P23232|GBB_LOLFO Guanine nucleotide-binding protein beta subunit. [Northern European squid] {Loligo forbesi}, partial (22%)
2007	ctttttctgg	5	C209229.1	78	0	
2008	gtggcctgca	5				no_annot
2009	ttaagggctc	5	C603923.1	16	1	
2010	gaaacggata	5	TC7960	62	0	similar to GP|15010514|gb|AAK77305.1||AY047573 GH08474p {Drosophila melanogaster}, partial (69%)
2011	gagaatagta	5				no_annot

2012	gcttgtcatc	5	TC17437	66	0	
2012	gcttgtcatc	5	TC17438	83	0	weakly similar to GP|27820099|gb|AAL28156.2||AY060608 GH02880p {Drosophila melanogaster}, partial (27%)
2013	tatacctacc	5	L01634|Sma.848	90	1	Myosin heavy chain (MYH)
2014	ttagttattt	5				no_annot
2015	ctacaccaga	5	C200617.1	43	1	
2015	ctacaccaga	5	C301239.1	30	0	
2015	ctacaccaga	5	C301778.1	54	1	
2015	ctacaccaga	5	C601188.1	3	1	
2015	ctacaccaga	5	C606891.1	2	2	
2015	ctacaccaga	5	C612164.1	52	1	
2015	ctacaccaga	5	C715432.1	30	0	
2015	ctacaccaga	5	C701949.1	54	1	
2016	tgtacaattg	5	TC16323	84	1	
2017	cattaggatg	5				no_annot
2018	ttgcctccga	5				no_annot
2019	ccacacggat	5	TC10458	72	1	weakly similar to GP|9651711|gb|AAF91234.1|AF224494_1|AF224494 arsenite inducible RNA associated protein {Mus musculus}, partial (23%)
2020	ttcaagttga	5	C202887.1	45	0	
2020	ttcaagttga	5	C303321.1	55	0	
2020	ttcaagttga	5	C609600.1	45	0	
2021	gttgaaaaaa	5				no_annot
2022	ctatcttcac	5	CD079386	68	0	weakly similar to SP|P32583|SR40_YEAST Suppressor protein SRP40. [Baker's yeast] {Saccharomyces cerevisiae}, partial (6%)
2023	agttctgttg	5				no_annot
2024	tgtattatgt	5	CD152794	71	0	similar to GP|28564767|dbj P0683C09.1 {Oryza sativa (japonica cultivar-group)}, partial (4%)
2025	cgactaccaa	5				no_annot

2026	ctacctggct	5	TC14938	50	0	weakly similar to GP|4837604|emb|CAB42989.1||AJ132370 actin {Allogromia sp.}, partial (9%)
2027	ggcacctcag	5	TC8279	33	0	
2028	ttgtggtata	5	TC12447	0	6	GP|4138530|emb|CAA09691.1||AJ011561 serine protease SmSP1 {Schistosoma mansoni}, complete
2029	ggactattgt	5				no_annot
2030	caatgaatgc	5				no_annot
2031	atgggaaatg	5	TC16241	44	0	
2032	ttacatcacc	5	TC6996	29	1	weakly similar to PIR|I50494|I50494 serine proteinase inhibitor CP9 - common carp, partial (5%)
2032	ttacatcacc	5	TC7124	56	1	weakly similar to SP|Q60854|PTI6_MOUSE Placental thrombin inhibitor (Protease inhibitor 6) (PI-6). [Mouse] {Mus musculus}, partial (10%)
2033	aagaagatgg	5				no_annot
2034	gtaaagtatt	5				no_annot
2035	ttagcaataa	5				no_annot
2036	tctattgaca	5	TC9319	90	0	similar to GP|9367031|gb|AAF87089.1|AF155149_1|AF155149 Flt3 ligand {Felis catus}, partial (6%)
2037	gactgactgg	5	TC16654	28	0	similar to GP|16769080|gb|AAL28759.1||AY061211 LD16074p {Drosophila melanogaster}, partial (7%)
2038	tagttcaatg	5	TC14159	88	0	
2039	caacttataa	5				no_annot
2040	cccattaaca	5				no_annot
2041	taatcttctc	5	TC17237	91	0	similar to GP|6273281|gb|AAF06327.1|AF190461_1|AF190461 lifeguard {Homo sapiens}, partial (5%)
2042	tgtgtgttct	5	AI976884	11	2	
2042	tgtgtgttct	5	AW018275	37	3	
2043	aatgacttca	5				no_annot
2044	tgttggcgaa	5	TC9357	19	0	similar to GP|23092876|gb|AAN11538.1||AE003477 CG32281-PA {Drosophila melanogaster}, partial (13%)
2045	ttgaagaact	5	CD202518	25	0	weakly similar to GP|17944266|gb|AAL48027.1||AY070556 LD31670p {Drosophila melanogaster}, partial (38%)

2046	aatgcaaaaa	5	AI975449	47	1	
2047	aatacttcac	5	TC11528	95	0	
2048	acaccccaac	5	CD071904	83	0	homologue to SP|Q27877|ENO_SCHMA Enolase(2-phosphoglycerate dehydratase) (2-phospho-D-glycerate hydro-lyase). [Blood fluke] {Schistosoma mansoni}, partial (20%)
2049	tggttttccg	5	TC16232	12	2	
2050	tttctacttg	5	TC7532	92	2	similar to GP|23134872|gb|AAG00866.2|AF255664_1|AF255664 major vault protein {Ictalurus punctatus}, partial (54%)
2050	tttctacttg	5	BF936458	4	3	
2051	ttgatacaaa	5				no_annot
2052	ctttatggta	5	TC13949	83	0	
2053	tcatcagcaa	5	TC16489	40	2	similar to GP|1698593|gb|AAB37254.1||U61846 3' end, includes homeodomain {Helobdella sp.}, partial (27%)
2054	ggttcacaag	5	C201586.1	60	0	
2054	ggttcacaag	5	C601150.1	88	0	
2055	ggccaaagaa	5	TC18334	64	0	similar to GP|7301095|gb|AAF56229.1||AE003746 CG6000-PA {Drosophila melanogaster}, partial (18%)
2056	gtgctaccta	5	TC17146	67	2	similar to GP|16226045|gb|AAL16062.1|AF420278_1|AF420278 carbonyl reductase {Anguilla japonica}, partial (16%)
2057	tacacttttt	5				no_annot
2058	tctctattag	5				no_annot
2059	ctcccatatg	5				no_annot
2060	atatgtaata	5	TC18196	86	0	
2061	tcatttccgc	5				no_annot
2062	ctggttcttt	5	M86396|Sma.850	98	0	Epidermal growth factor receptor (SER, class 1 product)
2063	tgcttacaac	5	C606404.1	74	1	
2064	ctggctgatt	5	C707965.1	31	0	
2065	aatcaatgcc	5				no_annot
2066	gttatgaatc	5	TC14616	90	0	
2067	tataatagcg	5	W06726	56	0	
2068	gataaaatgg	5				no_annot

2069	caataactta	5	TC10934	90	0	similar to GP|2970625|gb|AAC06013.1||AF051936 ryanodine receptor {Homarus americanus}, partial (7%)
2070	taccattgac	5				no_annot
2071	tgctcgtgaa	5				no_annot
2072	tgatcttcat	5				no_annot
2073	gtgcacccgt	5	CD071904	58	1	homologue to SP|Q27877|ENO_SCHMA Enolase(2-phosphoglycerate dehydratase) (2-phospho-D-glycerate hydro-lyase). [Blood fluke] {Schistosoma mansoni}, partial (20%)
2074	aataccggaa	5	CD186123	76	0	
2075	gcgccgtagc	5				no_annot
2076	catttgtcca	5				no_annot
2077	aacagcttgc	5	TC10746	4	7	similar to GP|13235592|emb|CAC33779.1||AJ301810 SclB protein {Streptococcus pyogenes}, partial (4%)
2078	tgtatgcaat	5				no_annot
2079	ctgtattctg	5	TC14127	35	0	
2080	ttcccccccc	5				no_annot
2081	tgtattacat	5	TC8115	64	0	
2082	ttgtcactga	5	AI764820	57	0	
2083	aatttaacct	5	TC13825	86	0	
2084	cagattttaa	5	AF043418|Sma.2	96	0	Clone WO12 transcription factor (HSF) mRNA, partial cds, alternatively spliced
2085	ccaacacgtg	5				no_annot
2086	taagcccgga	5				no_annot
2087	tctttaatga	5				no_annot
2088	taatgaacaa	5	TC15334	70	0	
2089	tacatcagtc	5	C607642.1	2	1	
2090	ctattctccc	5				no_annot
2091	tattataagc	5	C200285.1	79	0	
2091	tattataagc	5	C201865.1	16	2	
2091	tattataagc	5	C603701.1	91	0	
2091	tattataagc	5	C608340.1	34	2	
2092	aaacagtata	5	AI142130	62	0	

2093	gtttcgtgaa	5	TC10920	89	0	
2093	gtttcgtgaa	5	CD167031	35	0	
2093	gtttcgtgaa	5	BF936920	68	0	
2094	tatatgtgtg	5	TC17384	73	2	similar to GP|11992277|gb|AAG42496.1|AF323918_1|AF323918 NUDE-like protein {Mus musculus}, partial (32%)
2094	tatatgtgtg	5	CD090495	61	1	
2095	gggttagtga	5	TC7124	84	0	weakly similar to SP|Q60854|PTI6_MOUSE Placental thrombin inhibitor (Protease inhibitor 6) (PI-6). [Mouse] {Mus musculus}, partial (10%)
2095	gggttagtga	5	CD085857	80	0	
2096	ataacctgaa	5				no_annot
2097	ttagattgtt	5	TC8493	35	0	weakly similar to GP|499204|gb|AAC14192.1||M74824 D-E-A-D box protein {Drosophila melanogaster}, partial (14%)
2098	acaactgtat	5	C714271.1	10	0	
2099	tactctgtag	5	TC7966	84	0	similar to GP|17861912|gb|AAL39433.1||AY069288 GM14349p {Drosophila melanogaster}, partial (27%)
2100	taaatactct	5				no_annot
2101	tctctaactg	5	TC8298	88	0	
2102	cttatcgtgg	5	C200825.1	4	3	
2102	cttatcgtgg	5	C612314.1	6	2	
2103	taattcactc	5				no_annot
2104	ttactgtgat	5	C310075.1	23	0	
2104	ttactgtgat	5	C713400.1	23	0	
2105	gagggtacaa	5				no_annot
2106	gtgttttgtc	5				no_annot
2107	tacgtattta	5				no_annot
2108	ttacatatcc	5				no_annot
2109	ataatgaagt	5				no_annot
2110	aaataacgaa	5				no_annot
2111	gtgatcgcgt	5				no_annot
2112	gcgtaatgtg	5				no_annot

2113	ataatactta	5	TC12702	16	2	
2114	tcaattgtgc	5	TC9287	24	2	
2114	tcaattgtgc	5	CD166242	28	0	
2115	gctgtacttt	5				no_annot
2116	aaggttaacc	5				no_annot
2117	ctcaccaata	5				no_annot
2118	ttttttcaca	5				no_annot
2119	catcgttcta	5				no_annot
2120	tagcataatt	5	C318412.1	63	0	
2121	acaatctatt	5	TC17369	90	1	weakly similar to GP|11096269|gb|AAG30271.1|AF308470_1|AF308470 sterol C-14 reductase {Dictyostelium discoideum}, partial (8%)
2122	aaaacacctt	5				no_annot
2123	actttttcta	5	C301658.1	42	0	
2123	actttttcta	5	C311646.1	58	0	
2124	ataaagttgc	5	TC7785	69	2	weakly similar to GP|16797927|gb|AAL29216.1||AF411397 NADH dehydrogenase subunit 6 {Numenius madagascariensis}, partial (14%)
2125	atacggtctt	5				no_annot
2126	cttatatcca	5	TC17036	57	2	
2127	cggtcgaatg	5	TC13865	71	0	homologue to GP|23428921|gb|AAM33119.1||AY098931 NADH dehydrogenase subunit 5 {Aplysia punctata}, partial (7%)
2128	catatgatta	5	TC17549	91	0	
2129	aagggggggg	5				no_annot
2130	tacatttgat	5	CD079854	67	1	
2131	aagtaacatt	5	TC17123	64	2	weakly similar to GP|23172400|gb|AAN14096.1||AE003758 CG6323-PB {Drosophila melanogaster}, partial (39%)
2132	tgttgtatcg	5	TC11450	92	1	weakly similar to SP|P30260|CC27_HUMAN Protein CDC27Hs (Cell division cycle protein 27 homolog) (H-NUC). [Human] {Homo sapiens}, partial (6%)
2133	tatttgtaat	5	AI975160	0	2	

2134	tgtatctaca	5	TC15709	72	2	similar to SP|P32391|ARP3_HUMAN Actin-like protein 3 (Actin-related protein 3) (Actin-2). [Bovine] {Bos taurus}, partial (36%)
2135	ttgggggcaa	5				no_annot
2136	atgcgaccgt	5	TC8457	27	1	similar to GP|2832296|gb|AAD09407.1||AF044333 pleiotropic regulator 1 {Homo sapiens}, partial (51%)
2137	aaatagaaga	5	TC14319	88	0	
2138	cgttatcgtc	5	TC14641	55	0	
2139	attatctaat	5	TC15846	55	0	
2140	caaatgctta	5	TC10785	74	0	similar to SP|Q90YP3|RS28_ICTPU 40S ribosomal protein S28. [Channel catfish] {Ictalurus punctatus}, partial (87%)
2141	acaatttatg	5	C300535.1	83	0	
2141	acaatttatg	5	C316844.1	95	0	
2141	acaatttatg	5	C714242.1	92	0	
2141	acaatttatg	5	C703233.1	83	0	
2142	agctccggct	5	TC6931	15	9	similar to GP|19919877|gb|AAM08414.1|AF493056_1|AF493056 MF3 protein {Schistosoma japonicum}, complete
2142	agctccggct	5	TC6931	15	1	similar to GP|19919877|gb|AAM08414.1|AF493056_1|AF493056 MF3 protein {Schistosoma japonicum}, complete
2142	agctccggct	5	TC7183	28	5	similar to GP|19919877|gb|AAM08414.1|AF493056_1|AF493056 MF3 protein {Schistosoma japonicum}, partial (69%)
2142	agctccggct	5	TC7183	28	0	similar to GP|19919877|gb|AAM08414.1|AF493056_1|AF493056 MF3 protein {Schistosoma japonicum}, partial (69%)
2142	agctccggct	5	CD168997	15	1	
2142	agctccggct	5	CD193025	62	0	similar to GP|19919877|gb|AAM08414.1|AF493056_1|AF493056 MF3 protein {Schistosoma japonicum}, partial (34%)
2142	agctccggct	5	CD180317	40	0	
2142	agctccggct	5	CD183106	66	0	homologue to GP|19919877|gb|AAM08414.1|AF493056_1|AF493056 MF3 protein {Schistosoma japonicum}, partial (9%)
2143	gcatcaacaa	5				no_annot

2144	ctaatcattg	5				no_annot
2145	ttctttgttt	5	TC12182	87	0	
2146	caatattgtg	5				no_annot
2147	atgaatcaaa	5	C210213.1	78	1	
2148	tgcgtgtgtt	5				no_annot
2149	aatttgtcat	5	C200024.1	93	0	
2150	ataactttct	5				no_annot
2151	ttaattattg	5				no_annot
2152	aacgaacctt	5				no_annot
2153	tgtttcgaca	5	TC6922	66	0	
2154	gtggtacagg	5	TC11552	7	3	homologue to GP|12248338|gb|AAG13164.2||AF216698 cytochrome b {Schistosoma mansoni}, complete
2155	cgtcggcaac	5	TC10735	81	0	similar to SP|Q9VC49|RPBX_DROME DNA-directed RNA polymerase II 7.6 kDa polypeptide(RPB10) (RPB7.6). [Fruit fly] {Drosophila melanogaster}, partial (97%)
2156	tcaataaatg	5				no_annot
2157	aagtgtcatt	5				no_annot
2158	aattgtattt	5	C611085.1	97	0	
2159	ggtaacgtat	5	TC14202	75	0	
2160	gaattacgta	5	TC10856	46	4	similar to GP|21745342|gb|AAM77350.1|AF520987_1|AF520987 LIMS2 {Homo sapiens}, partial (65%)
2161	cctgcgaaaa	5	TC10767	58	0	
2161	cctgcgaaaa	5	CD059765	34	0	
2162	gaaatgagtt	5				no_annot
2163	cacatacata	5	CD081943	77	0	weakly similar to GP|7582296|gb|AAF64268.1|AF208854_1|AF208854 BM-012 {Homo sapiens}, partial (23%)
2164	caagtgtgtt	5				no_annot
2165	ttgattggac	5	TC15225	31	0	similar to SP|Q12499|NOP5_YEAST Nucleolar protein NOP58 (Nucleolar protein NOP5). [Baker's yeast] {Saccharomyces cerevisiae}, partial (6%)
2166	attactcctc	5				no_annot

2167	tcaatgacga	5	TC8609	90	0	weakly similar to SP|Q9P804|TRM1_SCHPO N(2),N(2)-dimethylguanosine tRNA methyltransferase(tRNA(guanine-26,N(2)-N(2)) methyltransferase) (tRNA 2,2- dimethylguanosine-26 methyltransferase) (tRNA(m(2,2)G26)dimethyltransferase). [Fission yeast] {Schizosaccharomyces pombe}, partial (6%)
2168	cgactattag	5				no_annot
2169	tacaacgaaa	5				no_annot
2170	gttattagag	5				no_annot
2171	tacaagtagc	5	CD147105	8	2	
2172	tgagatagat	5	N21965	82	1	
2173	gtgtggttca	5	TC11511	25	0	weakly similar to GP|23093753|gb|AAF50194.2||AE003550 CG6718-PA {Drosophila melanogaster}, partial (7%)
2173	gtgtggttca	5	CD120299	59	3	
2174	tacatacacc	5	TC15485	26	4	weakly similar to GP|28866788|dbj|BAC65172.1||AB050010 Surf4 {Gallus gallus}, partial (31%)
2175	cagctgggaa	5				no_annot
2176	agaggatctc	5	TC18738	40	0	weakly similar to GP|10954046|gb|AAG25715.1|AF309387_1|AF309387 oxidation protection protein {Homo sapiens}, partial (10%)
2177	catctcattc	5	C211374.1	64	0	
2177	catctcattc	5	C601711.1	64	0	
2178	taggcttgta	5	C609818.1	92	1	
2179	ataccgacct	5				no_annot
2180	tgaccaaaac	5	C201644.1	2	4	
2180	tgaccaaaac	5	C602609.1	1	5	
2181	gtcacactac	5	C718299.1	89	0	
2182	ttgtacgtac	5				no_annot
2183	taagatatat	5	TC16477	78	0	
2184	ctttgtaaat	5				no_annot
2185	tataattcag	5	TC10974	90	1	similar to EGAD|125515|133870 cAMP-regulated phosphoprotein 19k {Sus scrofa}, partial (16%)

2186	aacaaatact	5	TC17683	90	0	similar to GP|17946123|gb|AAL49103.1||AY071481 RE55111p {Drosophila melanogaster}, partial (15%)
2187	agttgggcga	5	TC7740	72	0	weakly similar to GP|15284209|gb|AAF99462.2||AY003872 PV1H14080_P {Plasmodium vivax}, partial (12%)
2188	gaaactgctc	5				no_annot
2189	agatatcata	5				no_annot
2190	tttacgctcc	5	TC17098	34	1	
2191	atcacaatga	5	BF936729	89	0	weakly similar to GP|21717341|gb|AAL35327.2|AF439342_1|AF439342 ERG potassium channel {Oryctolagus cuniculus}, partial (6%)
2192	tttaataaag	5				no_annot
2193	aaactctata	5	TC7988	98	0	
2194	gtgagatgtt	5	C200688.1	27	2	
2194	gtgagatgtt	5	C202175.1	6	3	
2194	gtgagatgtt	5	C603834.1	6	3	
2194	gtgagatgtt	5	C607293.1	49	3	
2195	cgtccgcacg	5	TC7464	70	1	similar to GP|15213788|gb|AAK92169.1|AF400197_1|AF400197 ribosomal protein L35A {Spodoptera frugiperda}, partial (65%)
2196	aatgaaaccg	5				no_annot
2197	ataaatgcct	5	TC14853	93	0	
2197	ataaatgcct	5	BG931612	91	0	weakly similar to GP|20198916|gb histidine kinase DhkG {Dictyostelium discoideum}, partial (0%)
2198	tcagatgatc	5	TC19548	33	0	
2199	gtggctgttc	5	TC12183	93	0	
2200	aggcaaattg	5				no_annot
2201	taaacaatgg	5	CD068220	84	0	
2202	atattattgt	5	CD128521	27	1	
2203	cgacgccaac	5	TC18297	65	2	weakly similar to GP|11494371|gb|AAG35783.1|AF282244_1|AF282244 transformer-2 beta {Gallus gallus}, partial (11%)
2204	catataaagc	5				no_annot

2205	cggatcaagt	5				no_annot
2206	tgaagtaaga	5	TC14351	38	2	similar to PIR|E86368|E86368 F5O8.5 protein - Arabidopsis thaliana, partial (51%)
2207	gcaacaatgt	5	C317805.1	8	1	
2207	gcaacaatgt	5	C603016.1	5	5	
2208	agcaaaaaaa	5				no_annot
2209	gaatagttca	5				no_annot
2210	gatttcaaag	5				no_annot
2211	tgtttagcct	5				no_annot
2212	gtgggaggta	5	C201648.1	1	1	
2212	gtgggaggta	5	C605206.1	5	1	
2213	gatgttaagt	5	TC8025	41	1	similar to GP|6066480|emb|CAB58439.1||AJ250366 40S ribosomal protein S27 {Lumbricus rubellus}, partial (98%)
2213	gatgttaagt	5	TC18524	93	0	
2214	cgctcgaata	5	TC6928	30	5	similar to GP|27948814|gb|AAO25602.1||AY181248 EMG1 {Kluyveromyces delphensis}, partial (41%)
2215	aaatctcttt	5	TC13585	84	0	similar to GP|13277220|emb|CAC34409.1||Y18580 ZF-HD homeobox protein {Flaveria bidentis}, partial (6%)
2216	gctcttttat	5	TC17091	24	1	weakly similar to SP|Q9H3H5|GPT_HUMAN UDP-N-acetylglucosamine--dolichyl-phosphate N-acetylglucosaminephosphotransferase(GPT) (G1PT) (N-acetylglucosamine-1-phosphate transferase) (GlcNAc-1-P transferase). [Human] {Homo sapiens}, partial (12%)
2217	tatggttagc	5				no_annot
2218	tctgatggtc	5				no_annot
2219	tactcccaaa	5	CD079840	15	0	
2220	atttagaccg	5				no_annot
2221	acgttttcac	5				no_annot
2222	tatgtagagt	5				no_annot
2223	tctccagatc	5	CD072641	95	0	similar to GP|21322705|emb|CAD21007.1||AJ428202 glutamate carrier {Homo sapiens}, partial (15%)
2224	gattacttaa	5				no_annot

2225	tgtaaaattg	5				no_annot
2226	tgaatacata	5	CD165999	74	0	
2227	ggcaactcga	5	M60895|Sma.710	28	1	Sm14 fatty acid-binding protein delta E3 variant mRNA, complete cds; alternatively spliced
2227	ggcaactcga	5	gi|20270933|gb|AF492389.1|	19	0	Schistosoma mansoni Sm14 fatty acid-binding protein isoform T20 mRNA, complete cds
2228	cacagatata	5				no_annot
2229	gtaataaata	5				no_annot
2230	gactagtcct	5	TC12138	61	0	
2231	ctctgtatgt	5				no_annot
2232	cagatatctg	5				no_annot
2233	gaacagtcaa	5	TC7254	63	0	similar to PIR|T39903|T39903 serine-rich protein - fission yeast (Schizosaccharomyces pombe), partial (5%)
2234	tttcactgat	5				no_annot
2235	cttgtggagc	5	C200912.1	19	1	
2235	cttgtggagc	5	C713165.1	22	0	
2236	cagaaggagg	5	C610476.1	94	0	
2237	agggtatatc	5				no_annot
2238	ttgtatgctg	5	AA528913	77	0	
2239	tttaacctgg	5	TC13744	80	0	weakly similar to SP|Q9DB05|SNAA_MOUSE Alpha-soluble NSF attachment protein (SNAP-alpha) (N-ethylmaleimide- sensitive factor attachment protein, alpha). [Mouse] {Mus musculus}, partial (71%)
2240	tacttcgcaa	5				no_annot
2241	aacggtcagt	5	C609390.1	50	0	
2242	tggagactaa	5	TC14286	86	0	similar to GP|1199697|dbj|BAA11875.1||D83268 vitellogenin {Athalia rosae}, partial (11%)
2243	tattttttgc	5	TC6996	92	0	weakly similar to PIR|I50494|I50494 serine proteinase inhibitor CP9 - common carp, partial (5%)
2244	tgcatctcaa	5	TC14075	81	0	
2245	ttcagacttc	5	CD093237	35	2	
2246	aaattgtgcg	5	TC8978	49	1	
2247	caatagcact	5	TC10233	76	0	similar to GP|16769390|gb|AAL28914.1||AY061366 LD28985p {Drosophila melanogaster}, partial (96%)

2248	tacccagata	5	TC9832	44	0	homologue to GP|19718173|gb|AAG37698.1||AY009089 CMP190.5bL {Camelpox virus CMS}, partial (13%)
2249	aggttcgcat	5	TC7799	53	0	weakly similar to SP|Q9JLG5|B44L_MOUSE Brain protein 44-like protein (Apoptosis-regulating basic protein). [Rat] {Rattus norvegicus}, partial (83%)
2249	aggttcgcat	5	TC7800	41	0	weakly similar to SP|Q9JLG5|B44L_MOUSE Brain protein 44-like protein (Apoptosis-regulating basic protein). [Rat] {Rattus norvegicus}, partial (70%)
2250	aatctcatcg	5				no_annot
2251	ttgaacaagt	5				no_annot
2252	ataaatcatc	5	TC11284	33	1	
2253	aatcgtcgtg	5	TC15239	57	0	similar to PIR||I38191|I38191 nucleic acid binding protein - human (fragment), partial (14%)
2254	acaacagcat	5	C208512.1	48	1	
2255	ttagagtgaa	5	TC7159	95	2	weakly similar to SP|O88761|PSD1_RAT 26S proteasome non-ATPase regulatory subunit 1 (26S proteasome regulatory subunit S1) (26S proteasome subunit p112). [Rat] {Rattus norvegicus}, partial (65%)
2256	tattatgacg	5				no_annot
2257	tatggtgtaa	5	TC10841	91	0	similar to GP|19879589|gb|AAL71884.1||AY074880 source of immunodominant MHC-associated peptides {Homo sapiens}, partial (27%)
2258	gacaaccgtt	5				no_annot
2259	tttgtatgca	5				no_annot
2260	gactgtggat	5	TC7858	71	0	similar to GP|15291647|gb|AAK93092.1||AY051668 LD21785p {Drosophila melanogaster}, partial (6%)
2261	tggtaatcac	5	TC14434	77	0	
2262	catcgaaatt	5				no_annot
2263	ttgcctggtg	5				no_annot
2264	tgtaattagc	5	TC18741	74	2	
2265	gtttattcac	5	TC8079	12	1	similar to GP|15292401|gb|AAK93469.1||AY052045 LP06017p {Drosophila melanogaster}, partial (15%)
2266	aaacaatgtt	5	U30176|Sma.19	78	0	Trans-spliced
2267	tgattatcat	5	CD181481	7	0	
2268	gttcataaaa	5				no_annot
2269	tttttatgca	5	TC17095	96	0	

2269	tttttatgca	5	CD178109	52	4	similar to GP|3242347|emb|CAA19669.1||AL024484 EG:96G10.1 {Drosophila melanogaster}, partial (10%)
2270	tgaaatcaaa	5	TC8041	81	0	weakly similar to GP|7801298|emb|CAB91167.1||AL355920 NCS1 allantoate transporter {Schizosaccharomyces pombe}, partial (5%)
2271	taaatttcgc	5	TC13989	86	2	weakly similar to GP|1730288|gb|AAC50934.1||U61263 acetolactate synthase homolog {Homo sapiens}, partial (18%)
2272	agaagttatt	5	TC14287	97	0	weakly similar to SP|Q9UIJ5|ZDH2_HUMAN Zinc finger DHHC domain containing protein 2 (Zinc finger protein 372) (Reduced expression associated with metastasis protein) (Ream) (Reduced expression in cancer protein) (Rec). [Human] {Homo sapiens}, partial (24%)
2273	atgatcctgg	5	TC17522	51	4	weakly similar to SP|Q9I047|TAL_PSEAE Transaldolase. {Pseudomonas aeruginosa}, partial (73%)
2273	atgatcctgg	5	CD167049	64	1	similar to SP|P37837|TAL1_HUMAN Transaldolase. [Human] {Homo sapiens}, partial (18%)
2274	gatacccgct	5	TC8694	69	0	weakly similar to GP|1589917|gb|AAC52898.1||U70476 cationic amino acid transporter-1 {Rattus norvegicus}, partial (17%)
2275	ctaaacatat	5				no_annot
2276	gtcaatgtac	5				no_annot
2277	aataaaatgg	5				no_annot
2278	aaatccccac	5	TC14181	65	1	weakly similar to GP|15291409|gb|AAK92973.1||AY051549 GH19706p {Drosophila melanogaster}, partial (20%)
2279	tcatctgtaa	5	TC16890	85	0	
2280	tgatatacga	5				no_annot
2281	tgaacgtttt	5				no_annot
2282	ctcctgctcc	5				no_annot
2283	aaagttgttc	5				no_annot
2284	tcgagatata	5	AI977671	49	3	
2285	tatgtatgta	5	C607765.1	93	0	
2286	gacacttgaa	5				no_annot
2287	aataataatt	5	AJ312106|Sma.810	88	0	Cathepsin B endopeptidase (cb2 gene)

2288	cacacataga	5	TC16965	53	6	weakly similar to GP|2274968|emb|CAA04006.1||AJ000332 Glucosidase II {Homo sapiens}, partial (43%)
2289	aagagtgaat	5	CD076538	19	0	
2290	tttagcaata	5				no_annot
2291	tatattcaga	5	TC14241	85	0	weakly similar to SP|Q92Q54|EFTS_RHIME Elongation factor Ts (EF-Ts). [Sinorhizobium meliloti] {Rhizobium meliloti}, partial (15%)
2292	gtagatgaca	5	TC7051	50	0	weakly similar to GP|20043237|dbj|BAA87877.1||AB009996 Dak2 {Drosophila melanogaster}, partial (65%)
2293	cacacataca	5	TC18168	4	1	
2294	tgtaacgtag	5				no_annot
2295	taacacgtgt	5				no_annot
2296	gctgtttctt	5	C606036.1	87	0	
2297	tgtactattg	5				no_annot
2298	ttattacttt	5	TC10596	98	0	similar to PIR|A26360|VPPG transitional endoplasmic reticulum ATPase - pig, partial (92%)
2299	tgctcttgtt	5	TC12588	40	0	
2300	gtgcatacac	5	TC10554	8	17	similar to GP|8099346|gb|AAF72103.1|AF154845_1|AF154845 MARK {Homo sapiens}, partial (13%)
2300	gtgcatacac	5	TC11097	76	1	weakly similar to GP|15625564|gb|AAL04162.1|AF411978_1|AF411978 WD40- and FYVE-domain containing protein 2 {Homo sapiens}, partial (31%)
2301	gtatcctaat	5	TC17967	96	0	similar to GP|21430724|gb|AAM51040.1||AY119180 SD02215p {Drosophila melanogaster}, partial (17%)
2302	tattggacta	5	CD080057	56	0	weakly similar to SP|P53667|LIK1_HUMAN LIM domain kinase 1(LIMK-1). [Human] {Homo sapiens}, partial (8%)
2303	gataaagcga	5	TC14050	49	0	
2304	cacttgcgtc	5	AY649844|Sma.4532	89	0	Insulin receptor tyrosine kinase 3 (RTK-3)
2305	tacttactga	5	TC13625	94	2	weakly similar to SP|Q63150|DPYS_RAT Dihydropyrimidinase(DHPase) (Hydantoinase) (DHP). [Rat] {Rattus norvegicus}, partial (58%)
2306	taaatgtgtg	5	TC13854	75	0	

2307	cttttctatc	5				no_annot
2308	cactaatctc	5	C202058.1	7	0	
2308	cactaatctc	5	C610132.1	7	0	
2309	tctttggtga	5				no_annot
2310	ttcaaatggt	5				no_annot
2311	taacagttag	5				no_annot
2312	aattccaatt	5				no_annot
2313	gatcgagaag	4	TC11940	53	1	
2314	tcactgatga	4				no_annot
2315	agtggtaaat	4				no_annot
2316	aattcatttt	4	TC10331	57	1	
2316	aattcatttt	4	TC14213	96	0	
2317	tgaccttgga	4				no_annot
2318	agtcttttac	4				no_annot
2319	gagagtggaa	4				no_annot
2320	ttgcttacct	4				no_annot
2321	actggtttag	4	TC14381	81	0	weakly similar to GP|7303053|gb|AAF58121.1||AE003810 CG8200-PB {Drosophila melanogaster}, partial (94%)
2321	actggtttag	4	AA883018	34	0	
2322	tttagttatc	4	TC14284	62	1	weakly similar to GP|23476994|emb|CAD48951.1||AL031744 P. falciparum RESA-like protein with DnaJ domain {Plasmodium falciparum 3D7}, partial (4%)
2323	cagaataaga	4				no_annot
2324	cgcacaaaag	4				no_annot
2325	caaatgagag	4				no_annot
2326	attaatgtag	4				no_annot
2327	ttgctatgat	4				no_annot
2328	gggagaataa	4				no_annot
2329	cttatcattg	4	TC10804	85	0	similar to GP|2245512|gb|AAB62537.1||AF001363 protein phosphatase-1 {Herdmania curvata}, partial (67%)

2330	atcaatatct	4				no_annot
2331	tattcatcgc	4	U53177|Sma.9	60	1	Ras protein homolog Psmras1 (Smras1)
2332	cttgttaaca	4				no_annot
2333	tttgttggga	4				no_annot
2334	aactcgctta	4				no_annot
2335	gatacatcag	4				no_annot
2336	ttacttagtg	4				no_annot
2337	catcatatga	4				no_annot
2338	gctcagcctt	4				no_annot
2339	gcattgaatc	4	TC8595	88	0	similar to GP|17862044|gb|AAL39499.1||AY069354 LD06016p {Drosophila melanogaster}, partial (19%)
2340	taatcttggg	4	TC10458	82	0	weakly similar to GP|9651711|gb|AAF91234.1|AF224494_1|AF224494 arsenite inducible RNA associated protein {Mus musculus}, partial (23%)
2341	gtaactatag	4				no_annot
2342	tgaatatttt	4				no_annot
2343	tgttagctta	4	TC10812	69	0	
2343	tgttagctta	4	TC14587	39	0	
2344	ttgacttttc	4				no_annot
2345	tcaatcatct	4	TC16323	24	3	
2345	tcaatcatct	4	TC18568	79	0	weakly similar to SP|O43815|STRN_HUMAN Striatin. [Human] {Homo sapiens}, partial (13%)
2346	agtgagtata	4	TC7698	85	0	
2347	ttaagtcaaa	4	TC8764	79	0	
2348	ttaattgtgc	4				no_annot
2349	tgatgtattc	4				no_annot
2350	tacacgaaat	4				no_annot
2351	gtcaaaggac	4	TC17541	78	0	weakly similar to GP|29028880|gb|AAO64819.1||BT005884 At3g52390 {Arabidopsis thaliana}, partial (23%)
2352	cccttatcgt	4	CD170219	66	0	similar to GP|12005665|gb|AAG44562.1|AF248964_1|AF248964 CUA001 {Homo sapiens}, partial (15%)

2353	gaagaccaaa	4				no_annot
2354	tgcgtccaaa	4				no_annot
2355	ccttcggaac	4				no_annot
2356	ggagaagtgg	4				no_annot
2357	atatattatg	4				no_annot
2358	cgcgtccaaa	4				no_annot
2359	tagatagtgg	4	Y08487|Sma.698	4	4	Elongation factor 1-alpha
2360	caaacacgtt	4				no_annot
2361	aactatgggc	4	CD197523	5	0	
2362	ccccagagta	4	TC16699	46	0	
2363	gttttacttt	4	X77211|Sma.27	93	1	IMP25 mRNA
2364	taaatgatta	4				no_annot
2365	atgcgtgtgc	4				no_annot
2366	acagcatcga	4	C602012.1	75	0	
2367	gaaaatgttg	4	AY227022|Sma.285	94	0	SINA (SINA)
2368	tatatttggc	4				no_annot
2369	ttatgatgag	4	TC17138	91	0	weakly similar to SP|O08618|KPRB_RAT Phosphoribosyl pyrophosphate synthetase-associated protein 2 (PRPP synthetase-associated protein 2) (41 kDa phosphoribosypyrophosphate synthetase-associated protein) (PAP41). [Rat] {Rattus norvegicus}, partial (77%)
2370	acactctagc	4				no_annot
2371	tagatacttg	4	TC14742	85	0	
2372	ttcgtcttta	4	TC7794	97	0	weakly similar to GP|7291735|gb|AAF47157.1||AE003462 CG3209-PA {Drosophila melanogaster}, partial (21%)
2373	ttcttcattc	4				no_annot
2374	tgaacatatc	4	TC17487	87	0	
2374	tgaacatatc	4	AI975303	62	0	
2375	tgaaaagaag	4	C603031.1	11	4	
2376	acatctgtaa	4				no_annot
2377	aacagagtgt	4				no_annot

2378	tggacctgaa	4	TC11367	69	0	
2379	agttccaaag	4	C207051.1	13	1	
2380	gtcctgttgg	4				no_annot
2381	gtatccctga	4	CD082093	90	0	
2382	tttatacaag	4	CD196372	90	0	similar to GP|7298314|gb|AAF53543.1||AE003651 CG13260-PA {Drosophila melanogaster}, partial (3%)
2383	ctgtaagcac	4				no_annot
2384	tgctcacagc	4	C305167.1	79	0	
2384	tgctcacagc	4	C608104.1	73	0	
2385	tgaccacagt	4				no_annot
2386	ataaaacatc	4				no_annot
2387	tgattgtata	4				no_annot
2388	tttacttatg	4	TC16573	97	0	similar to GP|35830|emb|CAA40296.1||X56976 ubiquitin activating enzyme E1 {Homo sapiens}, partial (51%)
2388	tttacttatg	4	CD197360	74	3	
2389	ctctgttagt	4	TC10664	9	4	similar to GP|16769280|gb|AAL28859.1||AY061311 LD23129p {Drosophila melanogaster}, partial (16%)
2389	ctctgttagt	4	TC19440	25	1	
2390	aacatttaat	4	AY351271|Sma.1202	87	0	Labial-like protein
2391	atcattgtca	4	TC17448	93	0	similar to GP|4959432|gb|AAD34346.1|AF119794_1|AF119794 TNF-receptor-associated factor 1 {Drosophila melanogaster}, partial (4%)
2392	tgctgctcat	4				no_annot
2393	gctctccttt	4	TC10617	35	1	similar to GP|22004048|dbj|BAC06474.1||AB078979 ubiquitin {Ciona savignyi}, partial (49%)
2394	atcattgtaa	4				no_annot
2395	taatctatac	4				no_annot
2396	attaaaaaaa	4	TC9590	2	1	
2396	attaaaaaaa	4	TC13139	48	0	
2396	attaaaaaaa	4	CD200832	67	1	
2397	gatttcgatg	4	C200188.1	62	6	
2397	gatttcgatg	4	C612561.1	76	2	

2398	acagtaatgg	4	TC15644	75	0	
2399	tttgttgtcc	4				no_annot
2400	gttatatctg	4				no_annot
2401	atggcattaa	4	TC10888	54	1	weakly similar to GP|18124147|gb|AAL59846.1|AF272946_1|AF272946 endophilin B1b {Mus musculus}, partial (9%)
2402	tgcggttccg	4	TC14351	65	0	similar to PIR|E86368|E86368 F5O8.5 protein - Arabidopsis thaliana, partial (51%)
2403	caacaaaaca	4				no_annot
2404	gttggaagaa	4	TC13819	75	1	weakly similar to PIR|T24395|T24395 dihydropteridine reductase- Caenorhabditis elegans, partial (53%)
2405	tttctaaatg	4				no_annot
2406	cattcccctt	4				no_annot
2407	gacaagtcat	4	TC9859	47	0	similar to GP|3876209|emb|CAB02667.1||Z81016 C. elegans MRP-4 protein (corresponding sequence F21G4.2) {Caenorhabditis elegans}, partial (5%)
2407	gacaagtcat	4	CD074841	65	1	weakly similar to GP|18478648|gb|AAL73230.1|AF337549_1|AF337549 EKN1 {Homo sapiens}, partial (34%)
2408	gaatcgatgt	4				no_annot
2409	tgctgttcgt	4				no_annot
2410	gtctcataac	4				no_annot
2411	acattattta	4				no_annot
2412	ggtgaccggc	4	CD068271	83	0	
2413	ttgttaattt	4	TC8370	81	0	
2414	taccgtcgaa	4				no_annot
2415	tatattttta	4	TC16973	82	1	similar to GP|18256123|gb|AAH21823.1||BC021823 WW domain binding protein 11 {Mus musculus}, partial (15%)
2415	tatattttta	4	CD086579	42	2	
2415	tatattttta	4	CD119833	53	2	
2416	catctcctac	4	C210886.1	13	0	
2417	ctacttatgc	4	C202265.1	5	10	
2417	ctacttatgc	4	C611330.1	5	10	

2418	cgtttgtgtg	4	C210413.1	81	0	
2419	attacttctt	4	TC13735	90	0	
2420	aggctcttga	4	U30175|Sma.742	11	6	Enolase trans-spliced
2421	ttaatatatg	4	TC19330	55	2	
2422	tccccttgga	4				no_annot
2423	actttagctg	4	TC10758	71	0	similar to GP|403456|gb|AAA35484.1||L02426 26S protease (S4) regulatory subunit {Homo sapiens}, partial (94%)
2424	ttaagttgag	4				no_annot
2425	actgtctgtt	4				no_annot
2426	atatagttag	4				no_annot
2427	atgatccatt	4	AF030964|Sma.97	53	0	Clone CL22 unknown
2428	cgcagtagtg	4				no_annot
2429	cgttgagtta	4				no_annot
2430	ttggactttc	4	TC14031	22	2	
2431	agagacaaag	4	TC19208	53	0	
2431	agagacaaag	4	CD125821	74	0	
2432	gaaccaaata	4				no_annot
2433	taaacgtaaa	4	TC7180	81	0	
2434	aaacgaagtg	4	TC13641	0	6	
2435	taattcagtt	4	TC10028	90	0	
2436	taacgttatg	4	C703336.1	35	0	
2437	catattttgt	4				no_annot
2438	ttgaaacgta	4				no_annot
2439	aagtcaattc	4	TC11884	91	0	weakly similar to SP|P49071|MKK2_DROME MAP kinase-activated protein kinase 2(MAPK-activated protein kinase 2) (MAPKAP kinase 2) (MAPKAPK-2). [Fruit fly] {Drosophila melanogaster}, partial (36%)
2440	caaaactcaa	4				no_annot
2441	tacgaagttt	4				no_annot
2442	acgatgaggg	4	TC18058	43	0	weakly similar to PIR|S26830|S26830 DNA-(apurinic or apyrimidinic site) lyase- bovine, partial (55%)
2443	gtaaacccat	4				no_annot

2444	cgccttattc	4	TC16834	60	1	weakly similar to GP|2745838|gb|AAB94760.1||AF039202 Hsp70/Hsp90 organizing protein; hop {Cricetulus griseus}, partial (28%)
2445	gagcagtgaa	4	CD160375	96	0	
2446	taaaagcaaa	4				no_annot
2447	tctatgtatt	4	TC10799	58	3	weakly similar to GP|4138422|emb|CAA04022.1||AJ000347 3'(2'),5'-bisphosphate nucleotidase {Rattus norvegicus}, partial (38%)
2447	tctatgtatt	4	CD137024	32	2	weakly similar to GP|21428526|gb|AAM49923.1||AY118554 LD34542p {Drosophila melanogaster}, partial (18%)
2448	cggaaaacat	4				no_annot
2449	ccttcattgg	4	TC15074	87	0	weakly similar to GP|5354054|gb|AAD42348.1|AF140360_1|AF140360 histone acetyltransferase {Homo sapiens}, partial (27%)
2450	tatatacaag	4	TC7523	30	4	weakly similar to GP|20151659|gb|AAM11189.1||AY094836 LD44221p {Drosophila melanogaster}, partial (67%)
2451	agagcagcaa	4	TC18811	80	0	weakly similar to GP|23272942|gb|AAH38029.1||BC038029 5'-nucleotidase, cytosolic III {Mus musculus}, partial (16%)
2452	gaacaagact	4	C201091.1	51	1	
2452	gaacaagact	4	C611775.1	9	4	
2453	attggactta	4	CD077845	22	1	
2454	aaaaggaagt	4	TC7740	4	4	weakly similar to GP|15284209|gb|AAF99462.2||AY003872 PV1H14080_P {Plasmodium vivax}, partial (12%)
2455	tacattccta	4				no_annot
2456	taaggattta	4				no_annot
2457	gaaagaattc	4	CD117348	70	0	
2458	caataaggta	4	C609369.1	25	0	
2459	gaatggtcca	4	TC12505	39	0	
2459	gaatggtcca	4	TC14373	78	0	similar to PIR|S27600|S27600 N-acetylglutamate synthase - Pseudomonas aeruginosa, partial (5%)

2460	ttggggtata	4				no_annot
2461	taagaatagt	4				no_annot
2462	tatactaacc	4				no_annot
2463	cataaccaca	4	C202032.1	24	4	
2464	catagaacac	4				no_annot
2465	cagtgaaaat	4	TC10492	38	1	weakly similar to SP|P51991|ROA3_HUMAN Heterogeneous nuclear ribonucleoprotein A3 (hnRNP A3) (D10S102). [Human] {Homo sapiens}, partial (11%)
2465	cagtgaaaat	4	TC16639	14	4	
2466	attcaatgtt	4	CD074845	12	0	
2467	ggactcaaga	4	TC8136	85	0	similar to GP|16769222|gb|AAL28830.1||AY061282 LD20087p {Drosophila melanogaster}, partial (7%)
2468	tcaagaggtt	4	TC18893	58	2	similar to GP|10187175|emb|CAC09061.1||AX025502 cDNA~Mango esterase {Mangifera indica}, partial (23%)
2469	tgtaaacttc	4				no_annot
2470	gatccaaggg	4				no_annot
2471	ttgtcaaaat	4	TC7575	29	4	
2472	atagtgcttg	4				no_annot
2473	tcaagatatg	4	TC14405	83	0	similar to GP|7293077|gb|AAF48462.1||AE003499 CG9203-PA {Drosophila melanogaster}, partial (16%)
2474	cgtggccaat	4				no_annot
2475	tatttcctcc	4				no_annot
2476	gtataacaga	4				no_annot
2477	gaggtcccgg	4	CD154125	24	0	similar to GP|23326083|gb|AAN24672.1|AE014707_11|AE014707 widely conserved GTP-binding protein {Bifidobacterium longum NCC2705}, partial (5%)
2478	cagctttaat	4	C201066.1	24	0	
2479	tgcatacgtg	4	TC17844	75	2	weakly similar to GP|5919219|gb|AAD56248.1|AF186273_1|AF186273 leucine-rich repeats containing F-box protein FBL3 {Homo sapiens}, partial (8%)
2480	gttcagaaag	4	TC7666	72	0	similar to GP|7299019|gb|AAF54221.1||AE003678 CG7483-PA {Drosophila melanogaster}, partial (98%)
2481	caatatgcgc	4				no_annot

2482	atttgtgaaa	4	C207950.1	92	0	
2482	atttgtgaaa	4	C612375.1	91	0	
2483	tttttcgcct	4	TC9941	93	0	similar to SP|P09580|GAL7_KLULA Galactose-1-phosphate uridylyltransferase. [Yeast] {Kluyveromyces lactis}, partial (13%)
2484	gtggagttgc	4	TC11625	75	0	
2485	tgtttgatag	4	TC18317	39	0	
2486	tctgtcacca	4				no_annot
2487	cggttatgca	4	TC13668	19	0	weakly similar to SP|P19338|NUCL_HUMAN Nucleolin (Protein C23). [Human] {Homo sapiens}, partial (7%)
2488	cgtatacaat	4	TC16814	46	1	homologue to GP|14588595|dbj|BAB61794.1||AB063181 calmodulin {Metridium senile}, partial (62%)
2489	tttggctttc	4	AA140575	73	0	
2490	gagtatggtc	4				no_annot
2491	cttggctttt	4	TC7041	69	2	weakly similar to PIR|A46032|S18159 ribosomal protein L7a, cytosolic - chicken, partial (86%)
2492	atctcttcag	4	TC10586	71	0	
2493	aaccaagttg	4				no_annot
2494	cttctagtgt	4	TC16524	94	0	weakly similar to SP|Q06892|POS5_YEAST POS5 protein. [Baker's yeast] {Saccharomyces cerevisiae}, partial (18%)
2495	acaacctagc	4	TC11474	85	0	weakly similar to SP|P11586|C1TC_HUMAN C-1-tetrahydrofolate synthase, cytoplasmic (C1-THF synthase) [Includes: Methylenetetrahydrofolate dehydrogenase; Methenyltetrahydrofolate cyclohydrolase; Formyltetrahydrofolate synthetase]. [Human] {Homo sapiens}, partial (8%)
2496	tcgtggtgtt	4	TC10957	98	0	weakly similar to GP|21428552|gb|AAM49936.1||AY118567 LD40170p {Drosophila melanogaster}, partial (4%)
2497	tggttttcgg	4	CD154578	75	0	similar to GP|2708309|gb|AAC51927.1||AF016371 U-snRNP-associated cyclophilin {Homo sapiens}, partial (86%)
2498	gcacttttaa	4	CD085551	79	0	homologue to GP|14486657|gb|AAK63225.1|AF325156_1|AF325156 merozoite surface protein 8 {Plasmodium falciparum}, partial (3%)
2499	cctgtaagct	4				no_annot

2500	gaaggagtgg	4				no_annot
2501	gcgtaagatc	4	TC17238	77	1	
2502	gatctctttt	4	TC11283	14	8	similar to SP|O31410|DEF2_BACST Peptide deformylase 2(PDF 2) (Polypeptide deformylase 2). {Bacillus stearothermophilus}, partial (8%)
2503	ttgtgtgaac	4				no_annot
2504	gacaatcatt	4	TC10918	51	0	weakly similar to GP|22759582|dbj|BAC10904.1||AB071965 Moco containing protein {Arabidopsis thaliana}, partial (8%)
2505	ctgattttcg	4				no_annot
2506	gacactaaaa	4	TC16987	87	0	weakly similar to SP|Q12904|MCA1_HUMAN Multisynthetase complex auxiliary component p43 [Contains: Endothelial-monocyte activating polypeptide II (EMAP-II) (Small inducible cytokine subfamily E member 1)]. [Human] {Homo sapiens}, partial (40%)
2507	agctgtacaa	4	C604197.1	1	10	
2508	aaaattgtga	4	TC14163	31	2	weakly similar to GP|14530319|emb|CAC42251.1||Z79596 C. elegans DYN-1 protein (corresponding sequence C02C6.1b) {Caenorhabditis elegans}, partial (23%)
2509	gaagggaaaa	4	AI976245	36	1	homologue to PIR|C86483|C86483 protein F5J5.17 [imported] - Arabidopsis thaliana, partial (7%)
2510	ttctcgttct	4	C604741.1	92	0	
2511	tgattttgtt	4	TC10794	84	0	
2512	ttttcagccc	4				no_annot
2513	acaactggga	4				no_annot
2514	cataactgtt	4				no_annot
2515	atatgatttc	4	TC17538	77	0	
2515	atatgatttc	4	CD114913	35	2	
2516	agactggtat	4				no_annot
2517	taccgaaagc	4				no_annot
2518	ttgccccaaa	4				no_annot
2519	gttgagtatt	4	TC11256	9	1	weakly similar to GP|15426576|gb|AAH13409.1|AAH13409|BC013409 transcriptional regulator protein {Homo sapiens}, partial (23%)

2520	gtgttccaaa	4				no_annot
2521	tcctccatac	4	TC16857	86	0	
2522	cgaagttggg	4	TC16819	23	1	
2522	cgaagttggg	4	TC16821	7	6	
2522	cgaagttggg	4	AI395625	73	0	
2523	gcaacacctc	4	C200663.1	23	2	
2524	ggaaggtgtt	4	TC13384	77	0	
2525	caaacgctaa	4				no_annot
2526	tggtattgat	4				no_annot
2527	aatatacata	4	TC13663	83	0	similar to SP|P31153|METK_HUMAN S-adenosylmethionine synthetase gamma form(Methionine adenosyltransferase) (AdoMet synthetase) (MAT-II). [Human] {Homo sapiens}, partial (95%)
2527	aatatacata	4	AI820482	0	1	
2528	caaaatgggc	4	C300028.1	38	0	
2528	caaaatgggc	4	C702601.1	38	0	
2529	tctggaaaac	4				no_annot
2530	ttcgaaccag	4				no_annot
2531	gtaactggat	4	TC9592	32	3	
2531	gtaactggat	4	CD170824	66	0	
2532	gagaggaagt	4	TC18955	54	0	
2533	aatgtgatga	4				no_annot
2534	aagtcaactg	4				no_annot
2535	atatgattga	4	TC19321	65	1	weakly similar to GP|24984038|gb|AAN68087.1|AE016440_7|AE016783 transcriptional regulator, TetR family {Pseudomonas putida KT2440}, partial (13%)
2536	tattctcaat	4				no_annot
2537	gggataatgc	4	AW400400	93	0	
2538	tctctgtggt	4	TC7584	14	0	
2539	gctgtaaact	4				no_annot
2540	tgacagtaat	4	L25067|Sma.29	67	1	Glucose transporter protein (GTP4)

2541	tatttgacga	4	TC17002	72	0	similar to SP|Q9Y2W1|T150_HUMAN Thyroid hormone receptor-associated protein complex 150 kDa component (Trap150). [Human] {Homo sapiens}, partial (3%)
2542	tttgagattg	4	CD092698	66	0	
2543	gttactaggg	4				no_annot
2544	cacaacctct	4	TC10968	90	0	weakly similar to SP|P54619|AAKG_HUMAN 5'-AMP-activated protein kinase, gamma-1 subunit (AMPK gamma-1 chain) (AMPKg). [Human] {Homo sapiens}, partial (14%)
2545	gcgtatatag	4	TC17428	65	1	weakly similar to SP|Q92535|PIGC_HUMAN Phosphatidylinositol-glycan biosynthesis, class C protein (PIG-C). [Human] {Homo sapiens}, partial (8%)
2546	ttgtttgtct	4				no_annot
2547	gatcaacttg	4	TC7539	89	0	weakly similar to GP|16182605|gb|AAL13531.1||AY058302 GH06357p {Drosophila melanogaster}, partial (40%)
2548	tgtactcgat	4	TC17674	81	0	weakly similar to GP|3618343|dbj|BAA33214.1||AB009398 26S proteasome subunit p40.5 {Homo sapiens}, partial (11%)
2549	taaacaccaa	4				no_annot
2550	gcatcgagta	4	CD075570	84	0	
2551	aagttttctc	4				no_annot
2552	tttgtgtatc	4	TC17041	79	0	similar to GP|23170394|gb|AAF51978.2||AE003602 CG1250-PA {Drosophila melanogaster}, partial (9%)
2552	tttgtgtatc	4	CD153609	87	0	
2553	tgtacgatgt	4				no_annot
2554	tagtaacaga	4				no_annot
2555	cctccaaaga	4				no_annot
2556	ggcaatgaag	4				no_annot
2557	actgaactac	4	TC11634	72	0	
2558	cgtataaggt	4				no_annot
2559	ttcaaaaaaa	4				no_annot
2560	tttacaaaat	4	TC17661	90	0	weakly similar to GP|23271482|gb|AAH23905.1||BC023905 SKB1 homolog {Mus musculus}, partial (27%)
2561	cacacacagg	4				no_annot

2562	ttcatttgtt	4	CD097970	72	0	similar to GP|215429|gb|AAA32327.1||M10637 gene E lysis protein {Enterobacteria phage G4}, partial (19%)
2562	ttcatttgtt	4	CD135518	91	0	
2563	aagctgagaa	4	TC14944	67	0	
2564	caaacttgct	4	TC13366	26	0	
2565	gactatacaa	4				no_annot
2566	aagggaggga	4	TC8848	15	0	
2567	gttcaggtac	4	TC10602	72	0	similar to GP|5679074|gb|AAD46846.1|AF160906_1|AF160906 BcDNA.LD02793 {Drosophila melanogaster}, partial (73%)
2568	ttttcttgag	4	TC17731	95	0	
2569	tatctgaacg	4	TC17965	65	0	similar to SP|O75027|ABC7_HUMAN ATP-binding cassette, sub-family B, member 7, mitochondrial precursor (ATP-binding cassette transporter 7) (ABC transporter 7 protein). [Human] {Homo sapiens}, partial (27%)
2570	gtggaaaatc	4	NP191174|AF025681.2|AAD54603.1	79	0	unknown
2571	catcatcgat	4	TC15760	28	2	
2571	catcatcgat	4	AI977016	35	1	
2572	atgttgagaa	4	TC14974	38	2	
2573	cgaatcccca	4				no_annot
2574	caaaccatta	4	AF030963|Sma.98	26	0	Clone CL1 unknown
2575	ttcgttaatc	4				no_annot
2576	ttatcaacta	4				no_annot
2577	catttacgtc	4	AY158214|Sma.118	51	1	Rho2 GTPase (Rho2)
2578	agggtctcga	4				no_annot
2579	gaaacttatg	4				no_annot
2580	gttctctcct	4				no_annot
2581	atggatataa	4				no_annot
2582	acaaaagcag	4	TC16547	13	11	similar to GP|14041148|emb|CAC38753.1||Y17848 heat shock protein 90 {Dendronephthya klunzingeri}, partial (84%)
2582	acaaaagcag	4	CD144666	3	1	GP|2827988|gb|AAB99949.1||AF034219 beta tubulin {Trichuris trichiura}, partial (11%)

2583	ttcttattgt	4	TC17925	43	4	
2584	tactcaacct	4	TC13956	91	0	
2585	aattttgtcg	4	TC7533	78	0	weakly similar to SP|Q9R0B9|PLO2_MOUSE Procollagen-lysine,2-oxoglutarate 5-dioxygenase 2 precursor(Lysyl hydroxylase 2) (LH2). [Mouse] {Mus musculus}, partial (34%)
2586	gattagaata	4	CD194040	2	3	
2587	tgtaattgtt	4	TC12840	55	2	weakly similar to PIR|T39903|T39903 serine-rich protein - fission yeast (Schizosaccharomyces pombe), partial (5%)
2588	ttgcattata	4	TC13827	57	2	similar to GP|4107017|dbj|BAA36294.1||AB001773 PEM-6 {Ciona savignyi}, partial (29%)
2589	aagaaaaaca	4	C306064.1	37	0	
2590	gaagcgaaat	4	U24281|Sma.721	26	1	14-3-3 protein (Sm14-3-3)
2591	ttcgacttag	4	TC7483	66	2	weakly similar to GP|20161358|dbj|BAB90282.1||AP003436 P0470A12.7 {Oryza sativa (japonica cultivar-group)}, partial (6%)
2592	accattaaga	4	TC13684	41	4	weakly similar to SP|Q9H6K4|OPA3_HUMAN Optic atrophy 3 protein. [Human] {Homo sapiens}, partial (12%)
2593	aacctggacc	4				no_annot
2594	tcaattcgat	4				no_annot
2595	gtgcttattg	4				no_annot
2596	taaaccttga	4	CD089191	87	0	
2597	gacagaaaaa	4				no_annot
2598	aaaataaacg	4	CD076881	20	4	similar to PIR|A53118|S39206 proline-rich protein V-beta 1 precursor - rat, partial (15%)
2599	caacattgtt	4	TC12436	82	0	weakly similar to GP|17946430|gb|AAL49248.1||AY071626 RE67480p {Drosophila melanogaster}, partial (10%)
2600	tgttgcattt	4	TC7434	13	2	weakly similar to SP|Q92598|H105_HUMAN Heat-shock protein 105 kDa (Heat shock 110 kDa protein) (Antigen NY-CO-25). [Human] {Homo sapiens}, partial (17%)
2600	tgttgcattt	4	TC7436	5	11	weakly similar to GP|1649012|gb|AAB17669.1||U72874 egg receptor for sperm {Strongylocentrotus purpuratus}, partial (16%)

2600	tgttgcattt	4	CD098226	54	1	weakly similar to SP|P34933|HS73_BOVIN Heat shock 70 kDa protein 3. [Bovine] {Bos taurus}, partial (9%)
2601	caaattgaat	4	CD064691	79	0	
2602	ttcatagatg	4	C205744.1	66	0	
2602	ttcatagatg	4	C605789.1	63	1	
2603	ttcatctttt	4	AY168137|Sma.687	80	0	Leucine-rich protein (SMV0119)
2604	gataattaag	4				no_annot
2605	ttaggataat	4	C300337.1	84	1	
2605	ttaggataat	4	C605658.1	91	1	
2606	gcatcccgat	4	TC8821	26	0	weakly similar to GP|18204708|gb|AAH21373.1||BC021373 synapse associated protein 1 {Mus musculus}, partial (16%)
2607	tgaacttgac	4	C201385.1	25	1	
2607	tgaacttgac	4	C610532.1	17	2	
2608	actctaatta	4	AY886887|Sma.5191	80	0	Sds
2609	atgaacgagt	4				no_annot
2610	catcatcttt	4	TC14756	44	4	similar to EGAD|115689|123873 serine-rich {Caenorhabditis elegans}, partial (12%)
2611	aagttctatg	4				no_annot
2612	agtgaggaga	4	C601718.1	21	0	
2613	cacaagacca	4	C209097.1	9	4	
2613	cacaagacca	4	C603080.1	7	4	
2614	atatttttca	4	C203620.1	17	4	
2614	atatttttca	4	C609245.1	17	4	
2615	acgagttgcc	4	TC17561	10	0	weakly similar to GP|21406625|gb|AAL48452.2||AY070830 AT29831p {Drosophila melanogaster}, partial (31%)
2616	gatatcgatc	4	CD078741	71	0	weakly similar to GP|2636680|gb|AAC06263.1||U66331 pol {Schistosoma mansoni}, partial (4%)
2616	gatatcgatc	4	AI740328	70	0	weakly similar to PIR|AC2453|AC2453 O-methyltransferase [imported] - Nostoc sp. (strain PCC 7120), partial (10%)
2617	atctcaacta	4	TC9818	42	1	
2617	atctcaacta	4	TC14519	41	0	

2617	atctcaacta	4	TC14958	84	0	
2617	atctcaacta	4	CD080881	19	0	
2617	atctcaacta	4	CD084755	31	1	
2617	atctcaacta	4	CD127326	16	2	
2617	atctcaacta	4	CD127998	30	1	
2617	atctcaacta	4	CD157750	2	0	
2617	atctcaacta	4	CD197357	80	0	
2617	atctcaacta	4	AA143826	95	0	
2617	atctcaacta	4	AI014233	77	0	
2617	atctcaacta	4	CD076339	54	1	
2617	atctcaacta	4	CD119656	13	0	
2617	atctcaacta	4	AI976121	7	2	repeat sequences
2618	tcgacggcag	4	TC14217	93	0	
2618	tcgacggcag	4	CD120784	90	0	
2619	gttactatat	4				no_annot
2620	aattatacag	4				no_annot
2621	cacagatttt	4				no_annot
2622	cataatatat	4	TC17726	86	0	
2622	cataatatat	4	CD081100	54	1	
2623	tactgtgcaa	4				no_annot
2624	tgcctcaaat	4				no_annot
2625	cttgcacaac	4				no_annot
2626	ttcaggattg	4	TC17241	95	1	weakly similar to GP|10048296|gb|AAG12342.1||AF294845 glycerol-3-phosphate dehydrogenase {Mus musculus}, partial (54%)
2627	ctcatagact	4				no_annot
2628	gttcattgaa	4	TC8599	90	0	similar to GP|19528489|gb|AAL90359.1||AY089621 RE35395p {Drosophila melanogaster}, partial (50%)
2629	ttgtttgctt	4	L46944	29	0	
2630	gttgccgtat	4				no_annot

2631	tcctgaatcc	4	TC17578	83	0	weakly similar to GP|11643561|gb|AAG39628.1|AF317058_1|AF317058 porcupine isoform D {Homo sapiens}, partial (10%)
2632	caaattcaat	4	C610897.1	33	4	
2633	catacataca	4	TC7937	44	1	similar to GP|1840087|gb|AAB47253.1||U83280 39 kDa antigen {Leishmania donovani}, partial (5%)
2633	catacataca	4	BF936161	65	0	
2634	ttcatacatc	4	TC10734	4	4	
2634	ttcatacatc	4	TC19649	39	0	similar to PIR|T50836|T50836 Yippee protein [imported] - human (fragment), partial (73%)
2635	gcaagaagag	4	TC13342	70	0	weakly similar to GP|2131129|emb|CAA73329.1||Y12802 amidase {Schistosoma mansoni}, partial (4%)
2636	gaaacgatac	4	L25066|Sma.30	91	0	Glucose transporter protein (SGTP2)
2637	tctcatactc	4	TC10769	63	1	similar to GP|7158855|gb|AAF37565.1|AF221506_3|AF221506 scavenger receptor protein CI precursor {Drosophila melanogaster}, partial (4%)
2638	atgtaggatc	4	CD178283	33	3	similar to GP|28972698|dbj|BAC65765.1||AK122483 mKIAA1265 protein {Mus musculus}, partial (6%)
2639	aatattacca	4				no_annot
2640	ggatcagtgt	4				no_annot
2641	tagtaatgat	4	TC15718	25	2	
2642	tgtatattaa	4	TC13959	95	0	
2643	ctgggcatac	4				no_annot
2644	ttttgatgct	4	TC14106	81	0	
2645	atttataaat	4				no_annot
2646	gacattaatt	4	C200428.1	22	2	
2647	tgtctccact	4	TC11669	77	0	
2648	tgaattgaaa	4				no_annot
2649	gagttctgtc	4				no_annot
2650	aaggcataat	4				no_annot
2651	ttgctcgaag	4				no_annot
2652	ctattcaagc	4				no_annot
2653	tttcatcctt	4	TC13848	94	0	

2654	gtatagtgaa	4	C208909.1	9	4	
2654	gtatagtgaa	4	C605486.1	9	4	
2655	attcctatga	4				no_annot
2656	tgtgtgtatg	4	TC13776	83	2	weakly similar to GP|262250|gb|AAB24621.1||S52010 1st Met is at position 21 {Mus sp.}, partial (22%)
2656	tgtgtgtatg	4	TC18106	36	0	
2657	ggcaaaccaa	4	TC8635	95	0	weakly similar to GP|15450581|gb|AAK96562.1||AY052658 AT4g39960/T5J17_130 {Arabidopsis thaliana}, partial (14%)
2658	ttactgatcc	4	TC11312	76	0	
2659	aacaaatcac	4	AW330541	25	1	similar to SP|P82664|RT10_HUMAN Mitochondrial 28S ribosomal protein S10 (MRP-S10) (MSTP040). [Human] {Homo sapiens}, partial (9%)
2660	ctattcgggt	4				no_annot
2661	aatacatcaa	4				no_annot
2662	atttgtttgt	4	TC8040	74	0	
2663	ttgtcgctcc	4	C200019.1	47	1	
2663	ttgtcgctcc	4	C610095.1	47	1	
2664	gcgacatctg	4				no_annot
2665	gatcactgat	4				no_annot
2666	tttgtgctac	4	TC18128	50	1	
2667	ttatcgacgt	4				no_annot
2668	aagttgattc	4	TC14796	78	0	
2668	aagttgattc	4	TC18466	62	1	weakly similar to GP|15010480|gb|AAK77288.1||AY047556 GH06695p {Drosophila melanogaster}, partial (23%)
2668	aagttgattc	4	CD079159	53	0	
2669	ttgcaaacag	4				no_annot
2670	atcaaaggga	4	TC7605	45	0	similar to PIR|A57630|A57630 transcription-associated factor OCP-II - guinea pig, complete
2671	atcttcaatg	4				no_annot
2672	tatacgaatg	4				no_annot
2673	tatatatctc	4	TC15904	95	0	

2674	gtcggttctt	4	C604398.1	90	0	
2675	gaagtataaa	4	AI976245	65	0	homologue to PIR|C86483|C86483 protein F5J5.17 [imported] - Arabidopsis thaliana, partial (7%)
2676	tttggaatgc	4				no_annot
2677	gtaacaaaca	4				no_annot
2678	tcacagaatg	4	TC16725	71	0	similar to PIR|T13159|T13159 E1B-55kDa-associated protein - human, partial (18%)
2678	tcacagaatg	4	CD198449	14	0	
2678	tcacagaatg	4	CD200688	80	0	
2679	acgtaactta	4	TC11239	78	0	weakly similar to GP|10716803|dbj|BAB16409.1||AB049623 HSCO protein {Mus musculus}, partial (78%)
2680	tatgatctct	4				no_annot
2681	tctaaccaac	4				no_annot
2682	gagatgacaa	4				no_annot
2683	tgttcggcta	4	CD062077	62	0	
2683	tgttcggcta	4	CD193006	86	0	
2684	gtctatctgt	4	TC17356	63	0	weakly similar to SP|O00303|IF35_HUMAN Eukaryotic translation initiation factor 3 subunit 5 (eIF-3 epsilon) (eIF3 p47 subunit) (eIF3f). [Human] {Homo sapiens}, partial (28%)
2685	atgcaattgt	4	TC8309	79	0	similar to SP|P41236|IPP2_HUMAN Protein phosphatase inhibitor 2 (IPP-2). [Human] {Homo sapiens}, partial (16%)
2686	taaatttgtt	4	TC8144	98	0	
2686	taaatttgtt	4	CD077818	87	0	
2687	taattcaata	4	TC11077	81	0	weakly similar to GP|18447293|gb|AAL68223.1||AY075385 LD23669p {Drosophila melanogaster}, partial (9%)
2687	taattcaata	4	CD067222	7	2	similar to PIR|A39314|A39314 gastricsinprecursor - bullfrog, partial (5%)
2687	taattcaata	4	CD123220	54	0	
2688	gcaaaaaaaa	4				no_annot
2689	gaaaattgat	4				no_annot
2690	tcactttttt	4	TC10443	96	0	similar to GP|29165352|gb|AAO65270.1||AY221261 selenoprotein W2a {Danio rerio}, partial (20%)

2691	gagaaaatac	4				no_annot
2692	cggcccagga	4				no_annot
2693	ctgaatatcc	4	AF130788_1_755	84	0	[CDS] Schistosoma mansoni cytochrome b (Cytb) gene, partial cds
2694	tacagtcgct	4	TC17659	47	0	homologue to GP|13751639|emb|CAC37285.1||Z97634 C367G8.1 (melanoma antigen P15) {Homo sapiens}, partial (5%)
2694	tacagtcgct	4	CD080930	42	1	similar to GP|13751639|emb|CAC37285.1||Z97634 C367G8.1 (melanoma antigen P15) {Homo sapiens}, partial (9%)
2695	tccattccag	4	TC17019	93	0	similar to GP|4204470|gb|AAD13394.1||AF090120 splicing factor U2AF35 {Takifugu rubripes}, partial (90%)
2696	gttgtgtgtg	4				no_annot
2697	gggttagcaa	4	CD079688	43	0	similar to SP|P45700|M1A1_MOUSE Mannosyl-oligosaccharide 1,2-alpha-mannosidase IA(Processing alpha-1,2-mannosidase IA) (Alpha-1,2-mannosidase IA) (Mannosidase alpha class 1A member 1) (Man(9)-alpha-mannosidase). [Mouse] {Mus musculus}, partial (3%)
2698	ttttaataca	4				no_annot
2699	ttttgaataa	4	TC7546	85	0	homologue to GP|16904828|gb|AAL30898.1||AF438343 phosphoglycerate mutase {Schistosoma japonicum}, complete
2700	tcctttatca	4	TC10874	94	0	weakly similar to GP|2792496|gb|AAB97076.1||AF041107 tulip 2 {Rattus norvegicus}, partial (17%)
2700	tcctttatca	4	TC15514	3	0	
2700	tcctttatca	4	CD115343	76	0	similar to PIR|I56530|I56530 gene VGF protein - rat, partial (4%)
2701	aaatgtgtac	4	TC17337	5	1	
2702	ggaaggacgt	4	TC15803	52	0	weakly similar to PIR|JN0500|JN0500 dihydroorotate oxidase, mitochondrial - fruit fly (Drosophila melanogaster), partial (32%)
2703	cgtgccttac	4	TC10471	67	6	similar to PIR|S05988|S05988 translation elongation factor eEF-2 - fruit fly (Drosophila melanogaster), partial (90%)
2704	tgtacacgag	4	CD137510	27	1	

2705	taccagcaca	4	TC9413	60	0	weakly similar to SP|P40124|CAP1_MOUSE Adenylyl cyclase-associated protein 1 (CAP 1). [Mouse] {Mus musculus}, partial (5%)
2706	ctttgcaaca	4				no_annot
2707	tacattttct	4	TC8679	48	0	
2708	aaaattgaat	4	TC17493	54	0	similar to GP|10121677|gb|AAG13318.1|AF266198_1|AF266198 GABA(A) receptor associated protein {Gillichthys mirabilis}, partial (95%)
2709	tatttggtat	4				no_annot
2710	tcacctaatg	4				no_annot
2711	atggacaata	4				no_annot
2712	aacaagacga	4	C607139.1	86	1	
2713	aaccatatcg	4				no_annot
2714	tcaggtaaaa	4				no_annot
2715	gaaaacatat	4	C207977.1	86	0	
2715	gaaaacatat	4	C611195.1	87	0	
2716	gtggatcact	4	gi|161025|gb|L02415.1|SCMHSP70X	35	3	Schistosoma mansoni heat shock protein 70 (HSP70) gene, complete cds
2717	gcgcaaacgt	4				no_annot
2718	actgtttact	4				no_annot
2719	aataaacatc	4				no_annot
2720	ttttgtaacc	4	TC10674	91	0	
2721	aacaaaaaaa	4				no_annot
2722	gtggatcaat	4				no_annot
2723	acaatgttca	4				no_annot
2724	gaaacatata	4				no_annot
2725	tgcatttttt	4	TC10879	86	0	similar to GP|29373133|gb|AAO72715.1||AY242061 thioredoxin 2 {Melopsittacus undulatus}, partial (33%)
2726	ttttcttcaa	4	TC14249	94	0	
2727	gaaaacttag	4	CD154428	69	0	
2728	tccatccatc	4				no_annot
2729	tatgtttgtg	4	CD064801	62	2	similar to GP|24983263|gb| DNA gyrase subunit A {Pseudomonas putida KT2440}, partial (1%)

2730	gttttgtgag	4				no_annot
2731	cgacaacagg	4	TC10433	71	2	weakly similar to GP|12188891|emb|CAC21554.1||AJ298278 poly(A) binding protein {Rattus norvegicus}, partial (47%)
2732	ataaaaaaaa	4	C606907.1	26	4	
2733	atgtgttgac	4	TC17833	57	1	weakly similar to GP|21430834|gb|AAM51095.1||AY119235 SD18780p {Drosophila melanogaster}, partial (12%)
2734	tatttcagac	4	CD086854	8	3	
2735	ttgatgataa	4	TC13951	82	0	similar to GP|3874218|emb|CAA90435.1||Z50109 C. elegans NUO-1 protein (corresponding sequence C09H10.3) {Caenorhabditis elegans}, partial (51%)
2735	ttgatgataa	4	TC16259	30	0	weakly similar to SP|Q12215|WSC3_YEAST Cell wall integrity and stress response component 3 precursor. [Baker's yeast] {Saccharomyces cerevisiae}, partial (6%)
2735	ttgatgataa	4	TC16556	4	4	weakly similar to PIR|T39903|T39903 serine-rich protein - fission yeast (Schizosaccharomyces pombe), partial (10%)
2736	aaggaatgaa	4	N20715	42	1	
2737	attacagtgt	4	TC17450	78	2	weakly similar to PIR|C86453|C86453 CDS protein F9L11.5 [imported] - Arabidopsis thaliana, partial (13%)
2738	aatcatctca	4	C602520.1	89	0	
2739	ttgcgaattt	4	TC17777	61	3	similar to GP|12239336|gb|AAG49437.1|AF136976_1|AF136976 sec13-like protein {Homo sapiens}, partial (39%)
2740	atgtcggtct	4	TC14251	74	0	
2741	cccaaccttc	4	TC6937	28	0	
2742	ttgcaaaaga	4	TC18612	4	1	weakly similar to GP|16769504|gb|AAL28971.1||AY061423 LD35644p {Drosophila melanogaster}, partial (33%)
2743	accataaaga	4				no_annot
2744	ggatgcatat	4	TC8869	59	0	
2745	ttccttcccg	4	TC12337	66	0	
2746	tactttcttg	4	TC14426	62	1	weakly similar to GP|1280392|gb|AAB41818.1||L49055 autoantigen {Rhipicephalus appendiculatus}, partial (30%)
2746	tactttcttg	4	CD091045	40	1	

2747	ttatcgtata	4	TC13935	91	0	weakly similar to GP|21392222|gb|AAM48465.1||AY118436 RH49423p {Drosophila melanogaster}, partial (28%)
2747	ttatcgtata	4	AI976798	73	0	
2748	gaatccattt	4	L27100|Sma.626	12	2	Serpin (SPI) gene, 3 end
2749	tctaacagaa	4				no_annot
2750	aaaagatgcc	4				no_annot
2751	gttgctgcaa	4				no_annot
2752	atgtataatg	4				no_annot
2753	ggcgtccaaa	4				no_annot
2754	ccccccctcg	4				no_annot
2755	cagaccgcaa	4	C318338.1	46	0	
2755	cagaccgcaa	4	C601209.1	50	0	
2756	gataaataca	4	TC10574	96	0	weakly similar to SP|P12695|ODP2_YEAST Dihydrolipoamide acetyltransferase component of pyruvate dehydrogenase complex, mitochondrial precursor(E2) (PDC-E2). [Baker's yeast] {Saccharomyces cerevisiae}, partial (18%)
2757	ggtttatgat	4	TC8367	89	0	weakly similar to GP|21464464|gb|AAM52035.1||AY121708 RH59310p {Drosophila melanogaster}, partial (22%)
2757	ggtttatgat	4	N21712	46	0	
2758	taaattatct	4				no_annot
2759	cagttgaagt	4				no_annot
2760	aagctcccag	4				no_annot
2761	ctctgttgat	4	TC8133	87	0	similar to GP|11094141|dbj|BAB17612.1||AB044661 XPA binding protein 1 {Homo sapiens}, partial (49%)
2762	cagacatacc	4				no_annot
2763	tcatacgaga	4				no_annot
2764	ctttcaatgg	4	TC10059	24	0	similar to GP|21430246|gb|AAM50801.1||AY118941 LD27255p {Drosophila melanogaster}, partial (7%)
2764	ctttcaatgg	4	CD163848	72	0	
2765	cacctaggcc	4				no_annot
2766	aatcaaaata	4	TC16804	32	1	

2766	aatcaaaata	4	TC17923	81	1	
2767	taaacaatat	4				no_annot
2768	atccattcgg	4	TC15639	74	0	
2769	gtaaaaagca	4	C305771.1	55	1	
2770	gcttgtaata	4	TC10722	36	0	
2771	gtcgatccga	4	J04017|Sma.821	50	6	Heat shock protein 86
2772	ttgaacacac	4				no_annot
2773	ggtttttcat	4	TC7416	87	0	GP|2246652|gb|AAC62254.1||AF006678 lysophospholipase homolog {Schistosoma mansoni}, complete
2774	tttaggccat	4	AY267032|Sma.1210	81	0	Clone 72D1 arginase
2775	ggctgggaac	4	TC15979	65	0	similar to SP|Q13437|DSS1_HUMAN Deleted in split hand/split foot protein 1 (Split hand/foot deleted protein 1). [Mouse] {Mus musculus}, partial (63%)
2776	gagaagatat	4	TC11029	83	0	similar to GP|10177292|dbj|BAB10553.1||AB008265 histone deacetylase {Arabidopsis thaliana}, partial (17%)
2777	taaactaatt	4				no_annot
2778	cgagcaggga	4				no_annot
2779	aatattgttg	4	TC9944	49	0	
2779	aatattgttg	4	TC12898	79	0	
2780	tggtatggca	4				no_annot
2781	gatatcattt	4	TC7535	92	0	similar to GP|3986285|dbj|BAA34993.1||AB017002 DjVLGA {Dugesia japonica}, partial (56%)
2782	taaacattca	4				no_annot
2783	gatagctaaa	4	TC7939	70	0	
2784	gcgtcctttt	4				no_annot
2785	gttcatacaa	4				no_annot
2786	gcgtcctttc	4				no_annot
2787	gttattttag	4				no_annot
2788	atgattggtc	4				no_annot
2789	taaactggtg	4				no_annot
2790	tattaacgag	4				no_annot
2791	tcgtgactca	4				no_annot

2792	tcgatgccga	4	CD183762	17	2	similar to PIR|A48133|A48133 pre-mRNA splicing SRp75 - human, partial (11%)
2793	tacagaaata	4	TC7511	61	4	
2793	tacagaaata	4	TC11364	7	1	similar to GP|22022313|dbj|BAC06513.1||AB079121 transformer-2a {Oryzias latipes}, partial (7%)
2793	tacagaaata	4	CD089949	53	0	
2793	tacagaaata	4	CD114436	37	0	
2794	taccagtgat	4	AW186610	13	0	
2795	aaaagcaatg	4	TC7692	3	2	weakly similar to GP|7573635|dbj|BAA94544.1||AB039933 polyposis locus protein 1-like 1 (TB2 protein-like 1) {Mus musculus}, partial (24%)
2796	caatttgttg	4				no_annot
2797	tcaatgaaac	4	TC6885	81	0	weakly similar to GP|15291535|gb|AAK93036.1||AY051612 GH25653p {Drosophila melanogaster}, partial (6%)
2798	gattttaaag	4	TC7481	82	0	weakly similar to SP|Q9QUM9|PSA6_MOUSE Proteasome subunit alpha type 6(Proteasome iota chain) (Macropain iota chain) (Multicatalytic endopeptidase complex iota chain). [Mouse] {Mus musculus}, partial (58%)
2799	atagtgattg	4				no_annot
2800	ttctgggcaa	4				no_annot
2801	atgtgtttct	4				no_annot
2802	aacgttcgat	4	C209916.1	5	6	
2802	aacgttcgat	4	C602538.1	5	6	
2803	tagacagtta	4				no_annot
2804	tcttcacctc	4	TC14866	90	0	
2805	ttaattacaa	4				no_annot
2806	aataaatgta	4				no_annot
2807	tgaaaacttt	4	C610174.1	7	4	
2808	atgttgaagg	4	C605636.1	63	0	
2809	aagttataag	4	C201482.1	10	0	
2809	aagttataag	4	C608323.1	9	0	
2810	ttttgatcag	4				no_annot

2811	acaggcggag	4				no_annot
2812	tgaattttga	4				no_annot
2813	taatttctta	4	TC10612	94	0	similar to SP|Q93009|UBP7_HUMAN Ubiquitin carboxyl-terminal hydrolase 7(Ubiquitin thiolesterase 7) (Ubiquitin-specific processing protease 7) (Deubiquitinating enzyme 7) (Herpesvirus associated ubiquitin-specific protease). [Human] {Homo sapiens}, partial (27%)
2814	agttatgtgc	4	TC10384	79	0	similar to GP|6563200|gb|AAF17194.1|AF112206_1|AF112206 ras-related protein rab-14 {Homo sapiens}, partial (60%)
2815	tccaccactt	4				no_annot
2816	cttcattgtg	4				no_annot
2817	tatgcattgg	4	TC12292	45	0	weakly similar to SP|P32529|RPA9_YEAST DNA-directed RNA polymerase I 13.7 kDa polypeptide(A12.2). [Baker's yeast] {Saccharomyces cerevisiae}, partial (32%)
2818	caattatcag	4				no_annot
2819	tcattcactg	4				no_annot
2820	atgaaacaag	4	CD074117	18	4	similar to GP|5869817|emb|CAB55574.1||AJ249395 cytochrome oxidase subunit III {Globodera pallida}, partial (6%)
2821	tcagtggaaa	4	TC10942	58	0	similar to GP|17027227|gb|AAK72580.1||AY038184 stromal cell-derived growth factor SF20/IL25 {Mus musculus}, partial (11%)
2821	tcagtggaaa	4	TC10943	62	0	similar to GP|17027227|gb|AAK72580.1||AY038184 stromal cell-derived growth factor SF20/IL25 {Mus musculus}, partial (11%)
2821	tcagtggaaa	4	TC10945	80	0	
2821	tcagtggaaa	4	CD082107	63	0	
2822	actactcggg	4				no_annot
2823	gtaataaacg	4				no_annot
2824	cgtggttagt	4	TC8169	79	0	homologue to SP|P52798|EFA4_HUMAN Ephrin-A4 precursor (EPH-related receptor tyrosine kinase ligand 4) (LERK-4). [Human] {Homo sapiens}, partial (6%)
2825	attaccagta	4	TC10998	60	0	weakly similar to SP|O15372|IF33_HUMAN Eukaryotic translation initiation factor 3 subunit 3 (eIF-3 gamma) (eIF3 p40 subunit) (eIF3h). [Human] {Homo sapiens}, partial (15%)

2826	tcagcgttta	4	TC7099	10	3	
2827	caagaatgcc	4				no_annot
2828	tgccactaat	4				no_annot
2829	tgattgatat	4	CD146179	47	1	
2830	taggtcagtt	4				no_annot
2831	tgttggtgac	4				no_annot
2832	ttttccgttt	4	L31531|Sma.694	96	1	Phosphofructokinase (PFK)
2833	gtgtacatta	4	TC10692	24	1	similar to GP|14714696|gb|AAH10489.1||BC010489 septin 6 {Mus musculus}, partial (37%)
2833	gtgtacatta	4	TC10693	73	0	similar to GP|14714696|gb|AAH10489.1||BC010489 septin 6 {Mus musculus}, partial (79%)
2834	gtaacagctg	4	C300792.1	63	0	
2834	gtaacagctg	4	C708173.1	63	0	
2835	aaaaaacgac	4				no_annot
2836	gatgggcgaa	4				no_annot
2837	acccggactc	4	TC8417	87	0	similar to SP|Q9Y4I5|MTL5_HUMAN Tesmin (Metallothionein-like 5, testis-specific) (Testis-specific metallothionein-like protein). [Human] {Homo sapiens}, partial (19%)
2838	aaattttatt	4	CD064012	52	2	
2839	gatgtcgtac	4				no_annot
2840	ttattgtcca	4	TC14144	73	0	weakly similar to GP|16588408|gb|AAL26792.1|AF310723_1|AF310723 NEDD8-conjugating enzyme NCE2 {Homo sapiens}, partial (46%)
2841	aatcgttagg	4				no_annot
2842	ttgttttggg	4	AF130788_1_755	22	2	[CDS] Schistosoma mansoni cytochrome b (Cytb) gene, partial cds
2843	tctcctttct	4	TC11938	65	1	
2844	agtacgggtg	4				no_annot
2845	caccacgtgt	4	AF358445|Sma.1137	58	0	Glutaminyl-tRNA synthetase
2846	taagattcat	4	TC17281	50	3	
2847	cggctcaggg	4				no_annot
2848	acaagtattt	4				no_annot

2849	aaaggcgaat	4	TC18747	75	0	
2850	tatatgaaaa	4	CD118831	14	4	
2851	tttgcgttcg	4	TC14285	73	0	
2852	ttgaacaagg	4	TC7796	63	3	
2852	ttgaacaagg	4	TC18983	18	2	
2853	cttattcaaa	4	TC7797	55	2	homologue to GP|26105953|gb|AAN78331.1||AC137988 TcC31.4 {Trypanosoma cruzi}, partial (5%)
2853	cttattcaaa	4	TC7798	69	0	
2853	cttattcaaa	4	TC7948	96	0	
2853	cttattcaaa	4	CD087039	90	0	
2854	gttcactaaa	4				no_annot
2855	aaggtgaagc	4	CD085455	57	1	
2856	tccttgtgta	4				no_annot
2857	aatcagtatg	4	M85304|Sma.283	0	8	Homeodomain protein (smox-5)
2858	tggctttgta	4				no_annot
2859	tcatacagga	4				no_annot
2860	cactgcttat	4				no_annot
2861	tgacaattta	4				no_annot
2862	aattgctatg	4	TC7609	97	0	
2863	tttgccctct	4				no_annot
2864	cagcaagttc	4	C202178.1	84	0	
2864	cagcaagttc	4	C609804.1	85	0	
2865	agcgtccttt	4				no_annot
2866	atacagagtc	4	TC16682	72	2	similar to SP|P38646|GR75_HUMAN Stress-70 protein, mitochondrial precursor (75 kDa glucose regulated protein) (GRP 75) (Peptide-binding protein 74) (PBP74) (Mortalin) (MOT). [Human] {Homo sapiens}, partial (89%)
2867	aataaatttt	4	TC7067	95	0	similar to GP|20067631|emb|CAC83780.1||AJ306394 phosphoglucose isomerase {Boiga kraepelini}, partial (51%)
2867	aataaatttt	4	TC15718	7	5	
2867	aataaatttt	4	TC17767	80	0	similar to GP|19528535|gb|AAL90382.1||AY089644 RE71924p {Drosophila melanogaster}, partial (77%)

2868	tatgtaagtg	4				no_annot
2869	gtgattatta	4				no_annot
2870	tgcgttgcag	4				no_annot
2871	caacattaat	4	TC7388	11	6	GP|1778026|gb|AAB63442.1||U60995 aspartic proteinase {Schistosoma mansoni}, complete
2871	caacattaat	4	TC7390	32	2	
2871	caacattaat	4	TC13944	72	1	similar to GP|1943772|gb|AAB52431.1||U97191 Rab family protein 2 {Caenorhabditis elegans}, partial (89%)
2871	caacattaat	4	CD166606	80	0	
2872	gaaggattat	4	TC11280	47	0	similar to SP|Q29466|VPP1_BOVIN Vacuolar proton translocating ATPase 116 kDa subunit A isoform 1 (Clathrin-coated vesicle/synaptic vesicle proton pump 116 kDa subunit) (Vacuolar proton pump subunit 1) (Vacuolar adenosine triphosphatase subunit Ac116). [Bovine] {Bos taurus}, partial (14%)
2873	gaaatatata	4	TC13398	6	1	
2873	gaaatatata	4	TC19660	87	0	
2874	cttctctaaa	4				no_annot
2875	aattgattca	4				no_annot
2876	atataaagag	4	TC13947	97	0	similar to GP|17945957|gb|AAL49023.1||AY071401 RE48767p {Drosophila melanogaster}, partial (26%)
2877	gaaggtggtc	4	TC17292	8	0	
2878	ctcacagttc	4				no_annot
2879	tacaaacatt	4	C611269.1	29	4	
2880	atgacgaaaa	4				no_annot
2881	aataaaatca	4	AF030966|Sma.731	95	0	Clone 5 unknown
2881	aataaaatca	4	gi|2623839|gb|AF030967.1|AF030967	97	0	Schistosoma mansoni clone 5 unknown mRNA, complete cds
2882	tgaactcgag	4				no_annot
2883	ttggagaaaa	4	TC10782	4	7	
2883	ttggagaaaa	4	TC14129	89	0	weakly similar to GP|3237304|gb|AAC23707.1||U91561 pyridoxine 5'-phosphate oxidase {Rattus norvegicus}, partial (70%)
2883	ttggagaaaa	4	CD080156	53	0	
2884	gctattcatt	4	TC13869	80	0	

2885	tgtgaccaca	4	TC12228	20	0	weakly similar to GP|29468131|gb|AAO85407.1|AF400670_1|AF400670 TRP26 {Mus musculus}, partial (37%)
2886	gaatcctcac	4	TC10961	59	4	similar to GP|7293941|gb|AAF49303.1||AE003522 CG5589-PA {Drosophila melanogaster}, partial (10%)
2887	gtagaactga	4	TC7430	39	0	
2887	gtagaactga	4	TC7431	87	0	
2887	gtagaactga	4	TC7432	30	1	
2888	atcaacattt	4	TC11037	67	0	homologue to GP|7293029|gb|AAF48416.1||AE003498 CG5548-PA {Drosophila melanogaster}, partial (10%)
2889	taaataattc	4	TC17629	47	4	similar to GP|11065995|gb|AAG28413.1|AF193757_1|AF193757 neuronal calcium binding protein NECAB2 {Rattus norvegicus}, partial (5%)
2890	aatagctttt	4				no_annot
2891	tctggaagaa	4	U54588|Sma.716	75	0	Fibrillin 2
2891	tctggaagaa	4	M86396|Sma.850	13	29	Epidermal growth factor receptor (SER, class 1 product)
2891	tctggaagaa	4	U54590|Sma.861	39	1	Fibrillin 2
2892	gtcttgcatc	4				no_annot
2893	tagaagagtg	4	TC8614	93	0	similar to GP|17945072|gb|AAL48597.1||AY070975 RE07451p {Drosophila melanogaster}, partial (82%)
2894	caaaattggt	4				no_annot
2895	taagagtggt	4	TC16869	77	0	similar to SP|P07199|CENB_HUMAN Major centromere autoantigen B (Centromere protein B) (CENP-B). [Human] {Homo sapiens}, partial (4%)
2896	aatgacgatg	4	TC10866	97	0	GP|24415108|gb|AAN59790.1||AF540394 trimeric G-protein alpha o subunit {Schistosoma mansoni}, complete
2897	tcaaaaaaaa	4	L81252	78	0	weakly similar to SP|Q9VMV6|SELT_DROME SelT-like protein precursor. [Fruit fly] {Drosophila melanogaster}, partial (13%)
2898	gaaggtggaa	4				no_annot
2899	gttaattgat	4	TC14168	67	2	similar to PIR|T39903|T39903 serine-rich protein - fission yeast (Schizosaccharomyces pombe), partial (6%)
2900	gtactctcgt	4				no_annot
2901	acctaagcaa	4				no_annot

2902	ctttgtgcac	4				no_annot
2903	aagctgctga	4	C210278.1	25	5	
2903	aagctgctga	4	C210278.1	25	0	
2904	tacatcgtta	4	TC8836	80	0	weakly similar to GP|3789917|gb|AAC67543.1||AF084928 erythroblast macrophage protein EMP {Homo sapiens}, partial (9%)
2905	tagaacaaac	4	gi|161041|gb|L07513.1|SCMMTEGANT	46	19	Schistosoma mansoni major tegumental antigen SM15 (GSM70) gene, complete cds
2905	tagaacaaac	4	gi|161041|gb|L07513.1|SCMMTEGANT	46	15	Schistosoma mansoni major tegumental antigen SM15 (GSM70) gene, complete cds
2905	tagaacaaac	4	gi|161041|gb|L07513.1|SCMMTEGANT	46	11	Schistosoma mansoni major tegumental antigen SM15 (GSM70) gene, complete cds
2905	tagaacaaac	4	gi|161041|gb|L07513.1|SCMMTEGANT	46	7	Schistosoma mansoni major tegumental antigen SM15 (GSM70) gene, complete cds
2906	gtgcctgaat	4				no_annot
2907	atagtatata	4				no_annot
2908	ctgatgaagc	4				no_annot
2909	tccccgtaat	4				no_annot
2910	tttgtgagcc	4				no_annot
2911	tcgtaattgt	4				no_annot
2912	tattacaggc	4				no_annot
2913	aggttgaacg	4	TC8029	84	0	
2914	tcgtgctttt	4	TC15157	2	2	
2914	tcgtgctttt	4	TC16313	92	0	
2915	gcgccttata	4				no_annot
2916	ccacctcctc	4				no_annot
2917	ggacaagtaa	4				no_annot
2918	tgtacagaag	3				no_annot
2919	ataaccctgt	3	CD159665	78	0	similar to GP|29714090|gb|AAO99855.1| Sequence 18 from patent US 6514697, partial (19%)
2920	ggcgagcgta	3				no_annot
2921	attattgaaa	3	C602505.1	89	0	
2922	tattcaacct	3				no_annot

2923	gtaactatta	3				no_annot
2924	aagtatctgt	3				no_annot
2925	tatctgtggt	3				no_annot
2926	ctgtgtgtgt	3	C300872.1	31	2	
2926	ctgtgtgtgt	3	C715734.1	31	2	
2927	gtggttgtaa	3				no_annot
2928	ttttgtcccc	3				no_annot
2929	taagtcgatt	3	TC14747	80	0	
2930	atctgtttca	3	TC15528	95	0	
2930	atctgtttca	3	TC18130	98	0	
2930	atctgtttca	3	TC18890	55	0	
2931	aaacacggta	3				no_annot
2932	ctgaacgtag	3	TC10291	57	0	
2933	agtaacaaca	3	C201478.1	41	0	
2934	gtgaagcgcc	3				no_annot
2935	cttttattag	3	gi|3599492|gb|AF085145.1|AF085145	61	1	Schistosoma mansoni NADH dehydrogenase subunit 5 (NU5M) mRNA, complete cds, mitochondrial gene encoding mitochondrial protein
2936	ggctgcgtca	3	C610988.1	60	0	
2937	tttttctact	3	C311983.1	72	0	
2938	tcgatattgt	3				no_annot
2939	cccagatcta	3				no_annot
2940	tacatatctg	3				no_annot
2941	tggccaatat	3	TC10255	49	1	
2942	tcattgtgga	3				no_annot
2943	acattaagtg	3				no_annot
2944	atattgtcgg	3				no_annot
2945	gcgtacgatt	3	C201971.1	22	6	
2945	gcgtacgatt	3	C608846.1	17	8	
2946	ttacagacgt	3				no_annot
2947	tacgcgccaa	3				no_annot

2948	caagtgtatc	3	C211012.1	76	1	
2949	tgtactttca	3				no_annot
2950	tggcaccatt	3				no_annot
2951	taggatgata	3				no_annot
2952	attactttgt	3				no_annot
2953	gcaacatata	3	CD195915	62	0	
2954	atgatgacat	3	TC9774	78	0	
2955	ctttcatata	3	TC17249	87	0	
2956	gtttgtttga	3	TC12293	66	0	
2957	gttaattttg	3	TC17296	78	0	
2958	tatcatatcg	3				no_annot
2959	catcaacata	3				no_annot
2960	gaaagacagg	3	TC13888	49	2	
2961	tctgaaaaac	3				no_annot
2962	gaagggttgt	3	TC13823	63	0	similar to GP|16565980|gb|AAL26325.1|AF399909_1|AF399909 alcohol dehydrogenase {Danio rerio}, partial (58%)
2963	tacataacat	3				no_annot
2964	aattgtgttt	3	TC7514	75	0	
2964	aattgtgttt	3	TC7965	80	3	weakly similar to PIR|AG1889|AG1889 WD-40 repeat protein [imported] - Nostoc sp. (strain PCC 7120), partial (3%)
2964	aattgtgttt	3	TC9641	11	1	
2965	cgagcgctga	3				no_annot
2966	gcgggtcgaa	3				no_annot
2967	attgggacta	3	TC15746	89	0	similar to GP|13242031|gb|AAK16516.1|AF331156_1|AF331156 serine protease precursor {Trichinella spiralis}, partial (4%)
2968	actatccggg	3				no_annot
2969	ttctatgctt	3				no_annot
2970	caggttttgc	3	TC15566	62	0	similar to GP|20151259|gb|AAM10989.1||AY094636 AT05708p {Drosophila melanogaster}, partial (6%)
2971	tatgtgagtg	3	AY299474|Sma.1185	87	0	Neuropeptide F precursor (NPF)

2972	aggattgttg	3				no_annot
2973	agcaagctcg	3				no_annot
2974	atgcagtaaa	3				no_annot
2975	aatatgaaag	3	TC10154	72	0	
2976	ttgtgtctga	3				no_annot
2977	tacatcctct	3				no_annot
2978	tacaccgaga	3	TC15527	42	2	weakly similar to GP|21645452|gb|AAM71014.1||AE003455 CG30398-PA {Drosophila melanogaster}, partial (9%)
2979	gatcatcgaa	3				no_annot
2980	tctgaataat	3				no_annot
2981	atgttcatta	3				no_annot
2982	cgaataatca	3				no_annot
2983	ctctccacag	3	TC18764	9	0	weakly similar to PIR|D96606|D96606 20S proteasome beta subunit (PBG1) [imported] - Arabidopsis thaliana, partial (11%)
2984	cacattcttt	3	TC16945	89	0	weakly similar to SP|Q99595|IM7A_HUMAN Mitochondrial import inner membrane translocase subunit TIM17 A (Inner membrane preprotein translocase Tim17a). [Human] {Homo sapiens}, partial (63%)
2985	tacaccgact	3				no_annot
2986	acattaagag	3				no_annot
2987	tacactgatc	3				no_annot
2988	tagatagtgt	3	CD146579	74	0	
2989	ttgactttgt	3	TC9108	57	2	weakly similar to GP|18447170|gb|AAL68176.1||AY075309 AT31826p {Drosophila melanogaster}, partial (12%)
2989	ttgactttgt	3	TC11070	88	0	weakly similar to SP|O60704|TPS2_HUMAN Protein-tyrosine sulfotransferase 2(Tyrosylprotein sulfotransferase-2) (TPST-2). [Human] {Homo sapiens}, partial (15%)
2990	tttatcttca	3	U86674|Sma.752	74	1	Calponin homolog
2991	tagttacaga	3	TC14194	35	3	
2992	cagtgcgtgc	3	TC16731	11	1	weakly similar to SP|Q9JMC3|DJA4_MOUSE DnaJ homolog subfamily A member 4 (MmDjA4). [Mouse] {Mus musculus}, partial (33%)

2992	cagtgcgtgc	3	TC16732	40	1	weakly similar to GP|15028450|gb|AAK81721.1|AF395203_1|AF395203 DnaJ-like protein {Cercopithecus aethiops}, partial (77%)
2993	cgccaaaatg	3	TC13984	72	0	weakly similar to SP|Q02645|HTS_DROME Hu-li tai shao protein. [Fruit fly] {Drosophila melanogaster}, partial (7%)
2994	gcacatctat	3	TC17376	0	4	weakly similar to GP|18089162|gb|AAH20819.1|AAH20819|BC020819 cholinephosphotransferase 1 {Homo sapiens}, partial (13%)
2995	tgagtagtta	3				no_annot
2996	tggaattata	3	TC18641	71	0	
2997	ttgtaggtca	3	C605209.1	77	0	
2998	tgatgttttg	3				no_annot
2999	aaggaaaaat	3	TC14363	75	0	weakly similar to GP|9963791|gb|AAG09695.1|AF183426_1|AF183426 HT004 protein {Homo sapiens}, partial (54%)
3000	atccaaaatg	3				no_annot
3001	tacaaaagat	3				no_annot
3002	cgctccttgg	3	TC16812	17	2	homologue to GP|14588595|dbj|BAB61794.1||AB063181 calmodulin {Metridium senile}, complete
3002	cgctccttgg	3	TC16813	15	3	GP|4160167|emb|CAA10601.1||AJ132193 calmodulin {Caenorhabditis elegans}, complete
3003	aattgtgtat	3	TC19634	88	0	similar to GP|1736829|dbj|BAA15973.1||D90848 Histidine-rich glycoprotein precursor. {Escherichia coli}, partial (5%)
3004	ataatattgt	3	TC7806	69	0	
3005	atgaatggtt	3				no_annot
3006	cttattatga	3	TC18789	79	0	
3007	atgaatggtc	3				no_annot
3008	aggggtacca	3				no_annot
3009	acagtttcct	3				no_annot
3010	ttccaccatt	3	TC11382	69	0	
3011	ttagtgcttg	3	C604858.1	5	3	
3012	gaaacctttc	3	TC14277	35	0	similar to PIR|D87498|D87498 HesB/YadR/YfhF family protein [imported] - Caulobacter crescentus, partial (24%)

3013	aaaactgtgc	3	CD083680	50	0	weakly similar to GP|21741703|emb|CAD41326.1||AL662946 oj991113_30.8 {Oryza sativa (japonica cultivar-group)}, partial (30%)
3014	tatagcaaaa	3				no_annot
3015	cttggtgaag	3	TC14122	66	0	weakly similar to SP|Q9VVG6|COQ4_DROME Ubiquinone biosynthesis protein COQ4 homolog. [Fruit fly] {Drosophila melanogaster}, partial (41%)
3016	gcaaaacaaa	3				no_annot
3017	gtatgaaatg	3	TC17217	64	2	similar to SP|Q9GP36|ERH_ECHMU Enhancer of rudimentary homolog. [Pork tapeworm] {Taenia solium}, complete
3018	catagcaaag	3	C702089.1	8	2	
3019	ctgctaatgt	3	TC13715	63	0	weakly similar to SP|O95865|DDH2_HUMAN NG,NG-dimethylarginine dimethylaminohydrolase 2(Dimethylargininase 2) (Dimethylarginine dimethylaminohydrolase 2) (DDAHII) (G6a). [Human] {Homo sapiens}, partial (19%)
3020	cagatgaaat	3	TC12769	84	0	weakly similar to GP|27574029|pdb|1N11|A Chain A, D34 Region Of Human Ankyrin-R And Linker, partial (7%)
3020	cagatgaaat	3	TC15814	20	3	weakly similar to GP|20306404|gb|AAH28501.1||BC028501 melanocyte prolifeating gene 1 {Mus musculus}, partial (23%)
3021	tatttgtgtt	3				no_annot
3022	atttggacga	3	TC17425	42	0	similar to PIR|T45963|T45963 cell division-like protein - Arabidopsis thaliana, partial (59%)
3023	atttgatatg	3				no_annot
3024	gactcaacat	3				no_annot
3025	ctggagctgc	3	C607478.1	16	0	
3026	taacagactg	3	TC8041	68	2	weakly similar to GP|7801298|emb|CAB91167.1||AL355920 NCS1 allantoate transporter {Schizosaccharomyces pombe}, partial (5%)
3027	actttactca	3	TC10634	41	0	
3027	actttactca	3	TC10635	3	1	
3028	ccagtaactt	3				no_annot
3029	aagagaccgt	3	Y12802|Sma.950	81	0	Amidase

3030	gctcgtgaat	3	TC19213	71	0	
3031	agatttctgt	3	TC19046	90	0	
3032	agatttctgg	3	C208909.1	4	5	
3032	agatttctgg	3	C605486.1	4	5	
3033	ttgatggtgt	3	TC8665	71	2	similar to GP|18482486|gb|AAL68961.1||AY072917 RNA-binding protein splice variant a {Homo sapiens}, partial (24%)
3033	ttgatggtgt	3	CD068576	70	0	
3034	caaggaagta	3				no_annot
3035	tcatttcgct	3	TC12426	60	0	
3036	ttggcaacaa	3				no_annot
3037	tttacattgt	3				no_annot
3038	aaaactcttg	3				no_annot
3039	ttgctcaacc	3				no_annot
3040	cttttgtttg	3				no_annot
3041	taataaacca	3	TC11191	15	4	
3042	gatagtccat	3				no_annot
3043	caattactca	3				no_annot
3044	gaccagcctt	3				no_annot
3045	tcgctggtac	3				no_annot
3046	attcttaaaa	3				no_annot
3047	tttgtgttct	3				no_annot
3048	ggtaggtcat	3				no_annot
3049	gctgattttt	3				no_annot
3050	ttacgtaaac	3	TC10883	83	0	similar to SP|Q13435|S3B2_HUMAN Splicing factor 3B subunit 2 (Spliceosome associated protein 145) (SAP 145) (SF3b150) (Pre-mRNA splicing factor SF3b 145 kDa subunit). [Human] {Homo sapiens}, partial (31%)
3050	ttacgtaaac	3	TC12557	34	0	
3051	ttgatggtaa	3	TC8205	27	0	similar to PIR|JE0307|JE0307 membrane protein - rat, partial (11%)
3051	ttgatggtaa	3	TC9930	67	1	similar to GP|15042371|gb|AAK82151.1|AF303741_290|AF303741 290R {Chilo iridescent virus}, partial (40%)

3052	cgcgatctac	3				no_annot
3053	tatgcaaaac	3	TC11155	68	0	weakly similar to GP|15291461|gb|AAK92999.1||AY051575 GH22314p {Drosophila melanogaster}, partial (7%)
3054	catttcggaa	3				no_annot
3055	tgtcttggaa	3	TC12718	7	4	
3055	tgtcttggaa	3	AA999449	16	1	weakly similar to GP|14334082|gb|AAK60524.1||AF367970_1|AF367970 thymus LIM protein TLP-A {Mus musculus}, partial (21%)
3055	tgtcttggaa	3	CD075781	14	1	
3056	gaaaacctgc	3				no_annot
3057	tttggtcact	3				no_annot
3058	atgtttatgt	3	CD126295	60	2	
3059	tgagaaactt	3	C202255.1	34	0	
3059	tgagaaactt	3	C606840.1	35	0	
3060	gtattttcac	3	TC13437	89	0	
3061	gagactggag	3				no_annot
3062	ttcattccta	3	TC17181	85	0	weakly similar to GP|21961626|gb|AAH34567.1||BC034567 Osbp2 protein {Mus musculus}, partial (40%)
3063	cgctttcaac	3				no_annot
3064	aaccatctct	3	TC18229	81	0	
3065	tccatctcag	3				no_annot
3066	cagacagaga	3				no_annot
3067	ttccgtgtat	3				no_annot
3068	ttttcagttg	3				no_annot
3069	cctacagcga	3				no_annot
3070	tatgatttga	3	TC12882	0	0	
3070	tatgatttga	3	CD200188	85	0	
3071	tggtttttgt	3	C719351.1	88	0	

3072	agacccagaa	3	TC8066	15	0	homologue to GP|22293702|dbj|BAC10046.1||AP003756 Speckle-type POZ protein-like~contains ESTs AU030338(E50924),AU030339(E50924) {Oryza sativa (japonica cultivar-group)}, partial (5%)
3073	gtcagtcgtg	3				no_annot
3074	gttatgttca	3	TC13528	78	3	GP|6649234|gb|AAF21436.1|AF195529_1|AF195529 14-3-3 epsilon {Schistosoma mansoni}, complete
3074	gttatgttca	3	AI068308	48	0	
3075	cacataaatg	3	C604698.1	3	2	
3076	ttgttttcat	3				no_annot
3077	tgtaaatact	3				no_annot
3078	gggttgatgg	3	TC14048	24	3	
3079	gacagcggtg	3				no_annot
3080	atcacactta	3				no_annot
3081	gtaacctctt	3				no_annot
3082	cctgtagact	3				no_annot
3083	gatcttcaca	3				no_annot
3084	gatgaaactc	3				no_annot
3085	gtgtagagtt	3	TC14767	16	3	
3085	gtgtagagtt	3	CD080975	84	0	
3086	tactccacaa	3	CD084154	77	0	
3087	tactgctatt	3	TC17501	97	0	weakly similar to SP|Q9VCE1|BCN1_DROME Beclin 1-like protein. [Fruit fly] {Drosophila melanogaster}, partial (22%)
3087	tactgctatt	3	CD195042	56	1	
3088	taactaccgg	3	TC10669	96	0	
3089	tgtagtaatt	3				no_annot
3090	tattatttgt	3	TC15601	10	0	
3091	acaagaatat	3	TC8322	21	1	similar to GP|20161512|dbj|BAB90435.1||AP003709 OSJNBb0006H05.12 {Oryza sativa (japonica cultivar-group)}, partial (17%)
3091	acaagaatat	3	CD114438	38	0	homologue to SP|P53471|ACT2_SCHMA Actin 2. [Blood fluke] {Schistosoma mansoni}, partial (19%)
3092	gaattatgtg	3				no_annot

3093	atatacgtga	3	CD089815	62	0	
3093	atatacgtga	3	CD164339	85	0	
3094	gtggacaaag	3				no_annot
3095	gaaggcgctg	3				no_annot
3096	tatatagacg	3				no_annot
3097	tcctctcaaa	3				no_annot
3098	cattttcgta	3	CD071621	94	0	
3099	catctatcca	3				no_annot
3100	aacgcttttc	3				no_annot
3101	cgtctgttta	3	TC10929	52	0	weakly similar to SP|O75340|PCD6_HUMAN Programmed cell death protein 6 (Probable calcium-binding protein ALG- 2). [Human] {Homo sapiens}, partial (74%)
3102	tatacatttt	3				no_annot
3103	ttgaaaaaaa	3	TC12931	36	1	
3103	ttgaaaaaaa	3	CD153720	79	1	similar to GP|14318657|gb|AAH09128.1||BC009128 DNA polymerase delta 1, catalytic domain {Mus musculus}, partial (15%)
3104	ttaatatata	3				no_annot
3105	taaacacttt	3				no_annot
3106	cttttatcct	3	TC17426	94	0	
3107	cacttttggt	3				no_annot
3108	gaactcaaga	3				no_annot
3109	aatgattgtt	3	C200810.1	88	0	
3110	tgtttattcg	3				no_annot
3111	ttttgggtct	3	TC16323	91	0	
3111	ttttgggtct	3	CD179019	19	5	
3112	agtagaagtg	3	TC17163	18	4	homologue to GP|1736824|dbj|BAA15968.1||D90848 Ribokinase. {Escherichia coli}, partial (6%)
3113	tagcgtcctt	3				no_annot
3114	atctgttcac	3	C202161.1	91	0	
3114	atctgttcac	3	C608299.1	89	0	
3115	gggttaacga	3	CD178519	12	1	

3116	gtggcgctga	3	TC12354	28	1	
3117	aatagttcct	3	TC10940	91	0	
3118	attactacca	3	TC10769	94	0	similar to GP|7158855|gb|AAF37565.1|AF221506_3|AF221506 scavenger receptor protein CI precursor {Drosophila melanogaster}, partial (4%)
3119	tgttacataa	3	C607143.1	56	1	
3120	tgattactag	3	TC8320	75	0	
3121	gaattatggt	3				no_annot
3122	caattggtgg	3				no_annot
3123	cgcctagatt	3	C605570.1	28	1	
3124	aaatgtatgg	3	C200453.1	2	1	
3124	aaatgtatgg	3	C604755.1	1	5	
3125	agtactatct	3	TC11016	78	1	weakly similar to SP|P47758|SRPB_MOUSE Signal recognition particle receptor beta subunit (SR-beta). [Mouse] {Mus musculus}, partial (28%)
3126	gtcaccaact	3	C206140.1	46	0	
3126	gtcaccaact	3	C601920.1	57	0	
3127	gtgcagatcc	3				no_annot
3128	ttgaaatact	3	TC13371	4	2	
3128	ttgaaatact	3	CD147645	48	0	
3129	ttgtgtcctg	3				no_annot
3130	ctgctgattt	3	gi|161056|gb|M35499.1|SCMPMYA1	6	5	Schistosoma mansoni paramyosin mRNA, complete cds
3131	tcagaatgat	3				no_annot
3132	atttaggcgt	3				no_annot
3133	ctggtcaacc	3				no_annot
3134	tgctttagtg	3				no_annot
3135	ctttaaagag	3				no_annot
3136	atttggaatg	3				no_annot
3137	ttacgtgttt	3	TC7950	8	5	weakly similar to GP|28272155|emb|CAD65059.1||AL935260 extracellular protein {Lactobacillus plantarum WCFS1}, partial (7%)
3138	tgtttgtgca	3				no_annot

3139	gtaggcgatg	3				no_annot
3140	tgttacttct	3				no_annot
3141	tatcttctac	3				no_annot
3142	aattattgta	3	TC18063	80	0	weakly similar to GP|3806019|gb|AAC69179.1||AF053770 ubiquinone biosynthesis protein coq7 {Mus musculus}, partial (59%)
3143	gatgcacata	3				no_annot
3144	atattccaat	3	TC7786	14	4	
3144	atattccaat	3	TC17239	6	1	weakly similar to GP|19879862|gb|AAM00189.1|AF248955_1|AF248955 arsenite-resistant protein ASR2 {Homo sapiens}, partial (5%)
3144	atattccaat	3	CD156035	72	1	
3145	actcatccgg	3				no_annot
3146	gctttgtttg	3	TC8078	80	0	weakly similar to SP|Q99614|TTC1_HUMAN Tetratricopeptide repeat protein 1 (TPR repeat protein 1). [Human] {Homo sapiens}, partial (29%)
3147	gagttgactt	3				no_annot
3148	attgttggct	3				no_annot
3149	ctcaatcgat	3	C204840.1	34	1	
3150	tacgtcataa	3				no_annot
3151	gtatatatta	3	TC7699	70	1	weakly similar to GP|19571635|emb|CAA87774.2||Z47808 C. elegans RAB-39 protein (corresponding sequence D2013.1) {Caenorhabditis elegans}, partial (41%)
3152	attacttcag	3	TC14709	94	0	
3153	tatacattac	3	TC18042	65	0	
3154	ttttatacat	3	TC19377	52	0	
3155	gtgatcaaca	3	TC10433	94	1	weakly similar to GP|12188891|emb|CAC21554.1||AJ298278 poly(A) binding protein {Rattus norvegicus}, partial (47%)
3156	tagcgtcccc	3	C300718.1	93	0	
3156	tagcgtcccc	3	C701001.1	91	0	
3157	gagattacac	3	TC9351	52	0	weakly similar to SP|Q9BRJ2|RM45_HUMAN 39S ribosomal protein L45, mitochondrial precursor (L45mt) (MRP-L45). [Human] {Homo sapiens}, partial (23%)

3158	ctcctctcct	3	TC6921	78	1	similar to GP|13508744|emb|CAC35115.1||AJ299689 NADH dehydrogenase subunit 6 {Ficedula parva}, partial (10%)
3159	gttaaatgag	3	C707909.1	8	0	
3160	gtgattaatt	3				no_annot
3161	ctcggatcgt	3	TC16348	47	0	weakly similar to GP|17648144|gb|AAC39568.2||AF016833 maltase-glucoamylase {Homo sapiens}, partial (5%)
3162	tgtgcacgtg	3	C312615.1	47	1	
3163	gcaaatacat	3	TC17236	84	0	weakly similar to GP|16648689|gb|AAL25537.1||AY058120 AT3g15460/MJK13_12 {Arabidopsis thaliana}, partial (33%)
3164	tgcgcaagat	3				no_annot
3165	cataatgagg	3				no_annot
3166	ctaatacgta	3				no_annot
3167	tatggtcaaa	3				no_annot
3168	ccacactagt	3	TC11501	62	0	weakly similar to PIR|T06379|T06379 SAR DNA-binding protein 2 - garden pea, partial (33%)
3169	acgaaagaga	3				no_annot
3170	gaacttaaaa	3				no_annot
3171	atgcgataga	3	TC17536	75	0	weakly similar to GP|22946167|gb|AAF52959.2||AE003628 CG31719-PA {Drosophila melanogaster}, partial (7%)
3172	tgggtatgta	3				no_annot
3173	gtgatattat	3	TC19078	2	5	weakly similar to PIR|A30350|A30350 dorsal protein - fruit fly (Drosophila melanogaster), partial (4%)
3174	agaagacggg	3				no_annot
3175	attatcatac	3	TC13591	86	1	similar to GP|3153910|gb|AAC17451.1||AF066859 muscle glycogen phosphorylase {Homo sapiens}, partial (91%)
3176	aaattgtatg	3				no_annot
3177	atgatgcgca	3	TC8999	25	0	
3177	atgatgcgca	3	CD081502	29	0	
3178	agcgtccaga	3				no_annot
3179	cacgaagtgg	3				no_annot

3180	ctgtacatat	3				no_annot
3181	tatagtgtgt	3	TC15487	44	0	
3182	tatcgttgtg	3	TC18741	89	0	
3183	tatgacacgt	3				no_annot
3184	ttaaaccgtg	3	TC16622	71	0	weakly similar to PIR|T42719|T42719 TPR-containing/SH2-binding phosphoprotein - mouse, partial (12%)
3185	atacagtccg	3	TC10760	94	0	similar to PIR|S65491|S65491 26S proteasome regulatory chain 12 - human, partial (60%)
3186	gtataacatt	3				no_annot
3187	tgtatttttt	3	TC10859	88	0	weakly similar to SP|P52907|CAZ1_HUMAN F-actin capping protein alpha-1 subunit (CapZ alpha-1). [Human] {Homo sapiens}, partial (22%)
3188	agcgtccaca	3				no_annot
3189	taagcaaggt	3	TC14694	15	3	
3190	gcacgccgct	3				no_annot
3191	acgattaaaa	3				no_annot
3192	aaatagttta	3	TC14750	97	0	
3193	cacagcttat	3	AI977486	68	0	weakly similar to SP|Q13616|CUL1_HUMAN Cullin homolog 1 (CUL-1). [Human] {Homo sapiens}, partial (9%)
3194	gtaattattg	3				no_annot
3195	actttttgtc	3	TC8948	96	0	similar to GP|12002020|gb|AAG43153.1|AF063594_1|AF063594 brain my036 protein {Homo sapiens}, partial (24%)
3196	tatgtatcat	3				no_annot
3197	aaaaatttag	3				no_annot
3198	cgaaaatgtt	3	C300826.1	55	0	
3198	cgaaaatgtt	3	C718991.1	55	0	
3199	taatgatttt	3	C200156.1	16	9	
3199	taatgatttt	3	C606630.1	44	4	
3199	taatgatttt	3	C717354.1	31	4	
3200	ctctgatcag	3	TC7765	79	0	
3201	cagaaatatc	3	CD161216	80	0	

3202	gaattcaata	3				no_annot
3203	tcgaattcat	3				no_annot
3204	atcaaaatga	3				no_annot
3205	ccaaggtctg	3				no_annot
3206	cagtgataca	3	TC11135	33	0	
3207	gccaattacg	3				no_annot
3208	agaagtccag	3	AY729668|Sma.1222	17	0	Glutathione peroxidase-2 (GPx2)
3209	gggataagca	3				no_annot
3210	tatgaaagat	3				no_annot
3211	aatagcaaaa	3				no_annot
3212	ggcagctaca	3	TC6890	72	0	weakly similar to GP|15010474|gb|AAK77285.1||AY047553 GH06247p {Drosophila melanogaster}, partial (18%)
3213	gttactcaca	3	TC14064	94	0	similar to GP|25009985|gb|AAN71158.1||BT001403 GH07620p {Drosophila melanogaster}, partial (49%)
3214	aaattgtaca	3	TC17215	96	0	weakly similar to GP|20380650|gb|AAH27575.1||BC027575 tRNA splicing 2' phosphotransferase 1 {Mus musculus}, partial (20%)
3214	aaattgtaca	3	CD185548	17	4	similar to GP|13161188|gb|AAK13499.1|AF338242_1|AF338242 nuclear protein 5qNCA {Homo sapiens}, partial (3%)
3215	taattaccca	3				no_annot
3216	gcaactcaat	3				no_annot
3217	ccaactgtcg	3	C714342.1	77	0	
3218	tactaacttc	3	TC7651	90	0	
3219	tgtttgttta	3	TC11488	74	0	similar to GP|15291313|gb|AAK92925.1||AY051501 GH15453p {Drosophila melanogaster}, partial (5%)
3220	cattttctta	3	U30260|Sma.18	85	0	Trans-spliced mRNA, clone SL cDNA-22
3221	gcgcgacttt	3				no_annot
3222	tacagacagc	3				no_annot
3223	aatgtgtggg	3				no_annot
3224	caatttagaa	3	TC17557	80	0	
3225	tccaagcctc	3				no_annot
3226	gctcgttgcc	3				no_annot

3227	atcaacaacg	3	TC19542	10	3	similar to SP|P59285|ALLA_PSEPK Ureidoglycolate hydrolase. [strain KT2440] {Pseudomonas putida}, partial (95%)
3228	tacttttgt	3				no_annot
3229	gtaattatct	3				no_annot
3230	ttatttatca	3				no_annot
3231	atgaggcaag	3				no_annot
3232	ttgtcataaa	3	Z34087|Sma.735	78	0	Fimbrin (FM)
3233	ttctgtctac	3	TC16193	85	0	
3234	tttcgtttag	3				no_annot
3235	acctttctaa	3	TC13591	92	0	similar to GP|3153910|gb|AAC17451.1||AF066859 muscle glycogen phosphorylase {Homo sapiens}, partial (91%)
3236	tataccgttt	3				no_annot
3237	ggttatgtgg	3				no_annot
3238	caaataaaat	3				no_annot
3239	gcgataccga	3	L40328|Sma.796	97	0	Ca2+ transport ATPase (SMA1)
3240	gacgtgttgc	3	TC17012	63	1	similar to GP|401789|gb|AAA21425.1||L24206 ipiB1 {Phytophthora infestans}, partial (8%)
3241	tcgactcaaa	3				no_annot
3242	cataccgttg	3				no_annot
3243	taaaaaatcc	3	TC8403	48	0	weakly similar to GP|7297164|gb|AAF52431.1||AE003614 CG11201-PA {Drosophila melanogaster}, partial (3%)
3244	attacattca	3	CD169932	67	0	
3245	atcaagagag	3				no_annot
3246	taattttgat	3				no_annot
3247	actggttagg	3				no_annot
3248	cagaaacgta	3	C601722.1	17	0	
3249	tactcattag	3				no_annot
3250	agaaatataa	3	TC13844	41	2	
3251	gtaactaatt	3				no_annot
3252	tggaaaaata	3	C209911.1	21	2	

3253	cgtaatgaag	3				no_annot
3254	gtttaacggt	3				no_annot
3255	tgtttatctg	3	TC8036	93	0	similar to SP|O75251|NUKM_HUMAN NADH-ubiquinone oxidoreductase 20 kDa subunit, mitochondrial precursor(Complex I-20KD) (CI-20KD) (PSST subunit). [Human] {Homo sapiens}, partial (75%)
3256	caattcattg	3				no_annot
3257	aaatgggaat	3				no_annot
3258	tacattgatt	3	C600091.1	57	1	
3259	tcgattgctg	3				no_annot
3260	gacaaactag	3				no_annot
3261	ttttgagatt	3	C311983.1	36	1	
3262	cagttcaact	3				no_annot
3263	cagttattgt	3	C201159.1	52	4	
3263	cagttattgt	3	C610150.1	49	5	
3264	tatcacggca	3				no_annot
3265	agagatgtta	3				no_annot
3266	cagtttaatc	3				no_annot
3267	tactcgtgca	3	C607470.1	80	0	
3268	agtggacgag	3	C202222.1	60	1	
3269	aaaagggttc	3				no_annot
3270	aaaagggtta	3				no_annot
3271	acacgttgta	3				no_annot
3272	accgtatatt	3				no_annot
3273	tcgaaaatac	3				no_annot
3274	aacctcttct	3	TC16406	3	0	
3275	tttgataaca	3				no_annot
3276	tgagaacgta	3	AI977156	79	0	
3277	tttataatga	3				no_annot
3278	tataccgtat	3	TC16962	92	0	
3279	ggcaaagtgg	3				no_annot
3280	gggtaaatgt	3				no_annot

3281	gagaaggcgg	3	TC13026	65	0	
3282	cctagttctt	3				no_annot
3283	cattcccaat	3				no_annot
3284	gttcatcata	3				no_annot
3285	caccttacac	3	TC16978	46	0	
3286	tcatcagcct	3				no_annot
3287	ctctcgttca	3				no_annot
3288	caaattcctc	3				no_annot
3289	gtaactaaca	3				no_annot
3290	ataggattgt	3				no_annot
3291	aaaattgtag	3	C604796.1	22	2	
3292	caacagcgag	3	TC16740	51	2	
3293	atttttgtca	3				no_annot
3294	cggaaaaacg	3	TC17707	9	1	
3295	gatagctgat	3	CD098410	65	0	weakly similar to GP|27762276|gb|AAO20276.1||AY178802 annexin 11b {Danio rerio}, partial (27%)
3296	tatcggcctg	3				no_annot
3297	aattcacgtt	3				no_annot
3298	ttgactaagc	3				no_annot
3299	aatatgcctc	3	C711220.1	23	1	
3300	attttatccg	3				no_annot
3301	tatgaatttt	3				no_annot
3302	aagttatgtt	3	C609928.1	86	1	
3303	aatgtgttta	3	TC10618	91	0	weakly similar to SP|O14638|NPP3_HUMAN Ectonucleotide pyrophosphatase/phosphodiesterase 3 (E-NPP 3) (Phosphodiesterase I/nucleotide pyrophosphatase 3) (Phosphodiesterase I beta) (PD-Ibeta) (CD203c antigen) [Includes: Alkaline phosphodiesterase I; Nucleotide pyrophosphatase(NPPase)]. [Human] {Homo sapiens}, partial (6%)
3304	tatgtgacaa	3	TC7060	41	4	weakly similar to SP|Q9UL36|Z236_HUMAN Zinc finger protein 236. [Human] {Homo sapiens}, partial (3%)

3305	tccctctgtc	3	AI740263	74	0	
3306	tggacaagga	3	TC8249	55	0	similar to GP|21703188|gb|AAM76091.1|AF483011_1|AF483011 thrombospondin-like protein {Boltenia villosa}, partial (9%)
3307	gcgtttaaat	3	TC8843	95	0	
3308	ttgcgaccgt	3				no_annot
3309	aagttaaggt	3				no_annot
3310	tgacccaact	3	C201119.1	17	5	
3310	tgacccaact	3	C604217.1	17	5	
3311	cataactggt	3	C201028.1	64	1	
3311	cataactggt	3	C611856.1	60	1	
3312	gtgcaaaaaa	3				no_annot
3313	gttctcacta	3	TC17690	51	0	
3314	aacaaaatag	3				no_annot
3315	tcactgcggt	3				no_annot
3316	aaattagctg	3	TC17741	46	0	
3317	tacgtgcata	3	TC14591	18	5	
3318	atctgaacta	3	TC7311	56	0	
3319	gtatcagtac	3	C211197.1	73	0	
3319	gtatcagtac	3	C604753.1	63	0	
3320	tgatgtggta	3	TC16653	22	2	
3320	tgatgtggta	3	TC16653	22	1	
3320	tgatgtggta	3	TC16653	22	0	
3321	cgaaactatg	3	CD116774	64	0	
3322	tggacaagaa	3	C602116.1	21	1	
3323	tgcaccattg	3	CD062333	49	2	
3323	tgcaccattg	3	CD081571	13	1	similar to GP|19528453|gb|AAL90341.1||AY089603 RE19804p {Drosophila melanogaster}, partial (72%)
3324	gcgagccgaa	3				no_annot
3325	ctcccaaacg	3				no_annot
3326	agtttctaaa	3	TC11219	95	0	

3327	tatacatcac	3	C300705.1	27	0	
3327	tatacatcac	3	C713851.1	27	0	
3328	agtgatatat	3				no_annot
3329	ctttttccca	3	TC11870	83	0	weakly similar to GP|28852226|gb|AAO55300.1||AE016862 PilB-related protein {Pseudomonas syringae pv. tomato str. DC3000}, partial (72%)
3330	gatggttata	3				no_annot
3331	ataaacaaga	3				no_annot
3332	gctttaaaga	3				no_annot
3333	tgaaacaaaa	3	TC17082	83	0	similar to GP|1943772|gb|AAB52431.1||U97191 Rab family protein 2 {Caenorhabditis elegans}, partial (37%)
3334	aatcaatgat	3	TC7412	8	2	
3335	aagctcaaag	3				no_annot
3336	ttgtccgagg	3				no_annot
3337	atggtcggcc	3				no_annot
3338	caacgtctac	3	TC7670	77	0	weakly similar to GP|20976838|gb|AAM27494.1||AY102665 GH22474p {Drosophila melanogaster}, partial (71%)
3339	tgaaaattca	3	C300229.1	14	1	
3339	tgaaaattca	3	C711404.1	14	1	
3340	atggttggtc	3	TC14270	20	0	
3341	tacttgcata	3				no_annot
3342	aaagaaaagc	3	C316127.1	74	0	
3342	aaagaaaagc	3	C706607.1	74	0	
3343	gtagctgaac	3				no_annot
3344	gaagttgttg	3	TC15486	67	0	
3345	ggcacgtgaa	3	S71584|Sma.780	25	2	Sm28 GST=28 kda glutathione S-transferase [Schistosoma mansoni, Puerto Rican, mRNA, 801 nt]
3346	attcactagt	3				no_annot
3347	ttcatttgta	3	CD073583	65	0	
3348	gaaactaatc	3	AI975270	32	1	
3348	gaaactaatc	3	AA999493	11	2	

3349	catataattg	3	TC17750	65	0	
3350	caaaacgttc	3				no_annot
3351	aacaattata	3	TC17700	6	0	
3351	aacaattata	3	CD112386	15	0	
3351	aacaattata	3	CD139340	17	0	
3351	aacaattata	3	AI394818	88	0	
3352	cttgtgaaaa	3	CD077341	31	1	
3353	caactatgta	3	TC17273	73	2	weakly similar to GP|7109287|gb|AAF36718.1|AF227508_1|AF227508 intestinal alkaline phosphatase-II {Rattus norvegicus}, partial (13%)
3354	ttacattgac	3	CD184291	49	2	
3355	cacctaatgg	3	C608771.1	34	1	
3356	tcattgtatt	3	TC17632	83	0	similar to SP|Q9Z2I9|SCB1_MOUSE Succinyl-CoA ligase [ADP-forming] beta-chain, mitochondrial precursor(Succinyl-CoA synthetase, betaA chain) (SCS-betaA) (ATP- specific succinyl-CoA synthetase beta subunit) (Fragment). [Mouse] {Mus musculus}, partial (51%)
3357	cccaactctc	3				no_annot
3358	tcacctttag	3				no_annot
3359	aaattgctga	3				no_annot
3360	gccgacaggt	3				no_annot
3361	gatatataag	3	AI740223	56	0	similar to GP|18496636|gb| NADH-ubiquinone oxidoreductase chain 1 {Hypocrea jecorina}, partial (3%)
3362	gactattcgc	3				no_annot
3363	gtccagataa	3				no_annot
3364	gatgaagttg	3	TC13718	52	5	similar to GP|1262435|emb|CAA61864.1||X89719 put. 26S protease subunit {Sus scrofa}, partial (96%)
3364	gatgaagttg	3	TC18818	72	1	weakly similar to GP|3873414|gb|AAC77512.1||U00043 Set (trithorax/polycomb) domain containing protein 1 {Caenorhabditis elegans}, partial (35%)
3365	ctccaacaat	3	TC17410	55	0	weakly similar to GP|18491239|gb|AAL69444.1||AY074628 At2g44070/F6E13.20 {Arabidopsis thaliana}, partial (13%)

3366	tccatatccg	3	TC18443	82	0	similar to GP|22001123|gb|AAM88323.1|AF512426_1|AF512426 presenilin enhancer {Drosophila melanogaster}, partial (24%)
3367	gaggctttta	3	C313986.1	74	0	
3367	gaggctttta	3	C609999.1	72	0	
3367	gaggctttta	3	C705216.1	69	0	
3368	tctttcaatt	3				no_annot
3369	tcagacgtca	3	C201239.1	43	0	
3369	tcagacgtca	3	C600580.1	43	0	
3370	gaaactggta	3				no_annot
3371	cccccctcgc	3				no_annot
3372	tctactacta	3				no_annot
3373	aagtactgaa	3				no_annot
3374	ttccctccta	3				no_annot
3375	tgtttcgctt	3				no_annot
3376	agaaagcata	3				no_annot
3377	tgaagagaaa	3				no_annot
3378	ttgatgcatt	3	C604362.1	90	1	
3379	atatgttaac	3	CD131908	42	1	
3380	caataactag	3	TC11143	64	0	
3381	gaaacgtgcc	3	TC9290	34	0	
3382	tagacgagaa	3	TC10467	24	7	GP|8132322|gb|AAF73250.1|AF153977_1|AF153977 eukaryotic translation initiation factor 5 {Schistosoma mansoni}, complete
3382	tagacgagaa	3	CD182257	75	0	similar to SP|Q22918|IF5_CAEEL Eukaryotic translation initiation factor 5 (eIF-5). {Caenorhabditis elegans}, partial (22%)
3383	aaccaggttt	3	TC17235	41	2	homologue to GP|1813565|gb|AAB41699.1||U72709 7B2 {Lymnaea stagnalis}, partial (6%)
3383	aaccaggttt	3	TC17662	63	0	weakly similar to PIR|T40916|T40916 ngg1-interacting factor 3 homolog - fission yeast (Schizosaccharomyces pombe), partial (13%)
3384	tgtacaacag	3				no_annot

3385	taactatgct	3				no_annot
3386	ccatcggctt	3	TC11439	90	0	weakly similar to SP|P39952|OXA1_YEAST Cytochrome oxidase biogenesis protein OXA1, mitochondrial precursor. [Baker's yeast] {Saccharomyces cerevisiae}, partial (13%)
3387	tactttacat	3	TC10728	87	0	similar to SP|O76767|ERD2_DROME ER lumen protein retaining receptor. [Fruit fly] {Drosophila melanogaster}, complete
3388	ccatttgata	3	C202177.1	42	3	
3388	ccatttgata	3	C606054.1	91	0	
3389	tacttatgat	3				no_annot
3390	agtgtgatga	3				no_annot
3391	aaacaagtgc	3				no_annot
3392	tttagactgt	3	TC7036	50	3	similar to SP|Q12224|RLM1_YEAST Transcription factor RLM1. [Baker's yeast] {Saccharomyces cerevisiae}, partial (3%)
3392	tttagactgt	3	CD154267	97	0	
3392	tttagactgt	3	CD078088	18	1	
3392	tttagactgt	3	CD097889	92	0	
3392	tttagactgt	3	CD199560	77	0	homologue to GP|4521264|dbj guanylate cyclase OlGC-R2 {Oryzias latipes}, partial (1%)
3393	cgaagacata	3	C711220.1	59	0	
3394	caagcttaca	3				no_annot
3395	cctaaacgtg	3				no_annot
3396	tatatgtgtt	3				no_annot
3397	cctaaacgta	3	TC16826	90	0	similar to EGAD|31660|4267 splicing factor 9G8, alt. transcript 3 {Homo sapiens}, partial (61%)
3398	ggtggcgggc	3	TC13521	62	3	similar to GP|7300349|gb|AAF55508.1||AE003721 CG7187-PA {Drosophila melanogaster}, partial (5%)
3398	ggtggcgggc	3	CD134178	46	0	weakly similar to GP|7300349|gb|AAF55508.1||AE003721 CG7187-PA {Drosophila melanogaster}, partial (7%)
3399	tcccctacat	3				no_annot
3400	acaccttgtg	3	TC13331	90	0	
3401	aacactagca	3	TC17956	97	0	

3402	atttcggtta	3	TC6940	4	3	
3402	atttcggtta	3	AI395144	11	0	
3403	tatatattgg	3				no_annot
3404	tccaaatggt	3	TC8548	73	0	similar to GP|22654872|gb|AAM98737.1||AY129231 protein related to Narf {Homo sapiens}, partial (10%)
3405	atcattcata	3	AA528947	73	0	
3406	ggttttagat	3				no_annot
3407	tggtgggggc	3				no_annot
3408	acttatcagg	3	TC8394	76	0	weakly similar to SP|Q14254|FLT2_HUMAN Flotillin-2 (Epidermal surface antigen) (ESA). [Human] {Homo sapiens}, partial (53%)
3409	tttagtgaaa	3	TC16126	91	0	
3410	tactggacaa	3				no_annot
3411	cttgctttta	3	C612151.1	83	0	
3412	tatcaaacac	3				no_annot
3413	atatcatttc	3				no_annot
3414	gtaacaacac	3	CD138090	43	2	
3415	gggaagccca	3				no_annot
3416	aaatgattgt	3				no_annot
3417	agcccctaat	3				no_annot
3418	tattcttgtt	3	TC10996	96	0	similar to GP|11762142|gb|AAG40349.1|AF324997_1|AF324997 AT4g17486 {Arabidopsis thaliana}, partial (19%)
3419	tatccttttg	3	TC14690	17	5	
3420	ttgtgttgtg	3	TC15265	60	0	
3421	gcaacagaga	3				no_annot
3422	atagttttaa	3	C610759.1	97	0	
3423	aagagaatat	3				no_annot
3424	tgctaggtat	3				no_annot
3425	gtaaactgct	3	CD146429	48	0	
3426	ctttttgaaa	3				no_annot

3427	aacgatgaac	3	CD081111	71	1	similar to GP|14325605|dbj|BAB60508.1||AP000996 metabolite transporter {Thermoplasma volcanium}, partial (4%)
3428	gttgtctctt	3	TC16824	72	5	SP|P06198|MYSP_SCHMA Paramyosin. [Blood fluke] {Schistosoma mansoni}, complete
3429	aaaacagctc	3	TC14516	93	0	weakly similar to SP|P36047|SD22_YEAST Protein phosphatases PP1 regulatory subunit SDS22. [Baker's yeast] {Saccharomyces cerevisiae}, partial (8%)
3430	ttgactgtga	3				no_annot
3431	aaattttgat	3	CD150210	11	0	
3432	ttacacttac	3	TC13820	80	0	similar to GP|5419655|emb|CAB46442.1||AJ012737 filamin, muscle isoform {Homo sapiens}, partial (8%)
3433	gtgcaagtgg	3				no_annot
3434	agagaaaaca	3	TC15587	2	3	
3435	aaaacggtag	3	CD073581	19	2	
3436	gtgggattgc	3	TC17967	77	1	similar to GP|21430724|gb|AAM51040.1||AY119180 SD02215p {Drosophila melanogaster}, partial (17%)
3437	tcaattgtta	3				no_annot
3438	gtgttttgta	3				no_annot
3439	acaataattc	3				no_annot
3440	accattcgtt	3				no_annot
3441	ttgttatatg	3	TC10678	59	0	weakly similar to GP|21711667|gb|AAM75024.1||AY128431 GM10569p {Drosophila melanogaster}, partial (5%)
3442	tgtcttgata	3				no_annot
3443	tcgacagaca	3				no_annot
3444	tgaattgatt	3				no_annot
3445	agcaatggga	3				no_annot
3446	tctgatttct	3	TC10677	95	0	weakly similar to PIR|I51681|I51681 poly(A) polymerase - African clawed frog, partial (37%)
3447	acctttgatt	3				no_annot
3448	ttacgactta	3	AI740215	14	0	
3448	ttacgactta	3	CD085454	33	4	

3449	gatacttgaa	3	AI977226	31	0	weakly similar to GP|28274800|gb|AAO34710.1||AY190280 neuronal calcium sensor 1 {Danio rerio}, partial (22%)
3450	aggcgagtgg	3				no_annot
3451	tgtatctatc	3	C208696.1	81	0	
3451	tgtatctatc	3	C600131.1	65	0	
3452	atgttgatgc	3				no_annot
3453	taattatgtt	3	AF510339|Sma.805	93	0	Elastase 2a (CE-2a)
3454	ctgtgttgat	3	TC11701	75	1	
3455	gacaacattt	3				no_annot
3456	taccaacaac	3				no_annot
3457	tacaaatacc	3	C201119.1	33	4	
3457	tacaaatacc	3	C604217.1	32	4	
3458	aaaaagtgga	3	TC9004	30	0	similar to GP|15292197|gb|AAK93367.1||AY051943 LD41978p {Drosophila melanogaster}, partial (6%)
3459	gattcattat	3	TC17785	9	2	weakly similar to GP|927736|gb|AAB64908.1||U33050 Ydr492wp; CAI: 0.19 {Saccharomyces cerevisiae}, partial (15%)
3459	gattcattat	3	TC18036	44	2	
3460	ggcgacaatc	3				no_annot
3461	gtttcgatga	3	AW017274	42	2	
3462	gccgtgtgta	3				no_annot
3463	cgttaccgta	3	AF232691|Sma.69	72	2	Tyrosine kinase 5 (Tk5)
3464	ataaacgtcc	3				no_annot
3465	caaacacaca	3				no_annot
3466	agcatagttt	3				no_annot
3467	tgctcaacaa	3				no_annot
3468	aatcataaca	3				no_annot
3469	gaaggctgat	3				no_annot
3470	agcattccaa	3				no_annot
3471	tacatttggc	3	Z27402|Sma.237	12	2	Eggshell protein
3472	ctcgttcgag	3				no_annot

3473	catcggcacc	3				no_annot
3474	ttaataccct	3	TC7396	94	0	similar to SP|P50538|MAD_MOUSE MAD protein (MAX dimerizer). [Mouse] {Mus musculus}, partial (11%)
3475	ataatgtaat	3				no_annot
3476	acgtgtaacg	3				no_annot
3477	ctttgttctt	3				no_annot
3478	ttcccgtaca	3				no_annot
3479	attgaccggc	3				no_annot
3480	ttagtcaaag	3				no_annot
3481	cttaacacca	3	TC11478	12	6	
3481	cttaacacca	3	CD073110	18	3	
3482	tgtatattca	3	TC8146	80	1	weakly similar to GP|11191800|emb|CAC16398.1||AJ276505 Cysteinyl-tRNA-synthetase {Mus musculus domesticus}, partial (6%)
3482	tgtatattca	3	TC17267	76	0	similar to GP|20377682|gb|AAM20821.1|AF368921_1|AF368921 P26 protein {Mus musculus}, partial (44%)
3483	atgaatgaat	3	TC9181	14	1	similar to SP|Q9H6R6|ZDH6_HUMAN Zinc finger DHHC domain containing protein 6 (Zinc finger protein 376) (Transmembrane protein H4). [Human] {Homo sapiens}, partial (6%)
3483	atgaatgaat	3	CD091992	78	1	
3484	gcatccgtca	3				no_annot
3485	atccaagagc	3	TC10902	34	0	weakly similar to SP|P51970|NUPM_HUMAN NADH-ubiquinone oxidoreductase 19 kDa subunit(Complex I-19KD) (CI-19KD) (Complex I-PGIV) (CI-PGIV). [Human] {Homo sapiens}, partial (49%)
3486	aggcctgcgt	3				no_annot
3487	taatttacgt	3				no_annot
3488	tccataaaaa	3				no_annot
3489	ccacgtaata	3				no_annot
3490	gtatcagcac	3				no_annot
3491	caaagatgaa	3				no_annot
3492	ggttggattc	3				no_annot

3493	gtatccagct	3	TC15814	80	0	weakly similar to GP|20306404|gb|AAH28501.1||BC028501 melanocyte prolifeating gene 1 {Mus musculus}, partial (23%)
3494	actggtgctg	3				no_annot
3495	tgtatgtggt	3	TC13460	28	1	
3495	tgtatgtggt	3	TC13491	94	0	
3495	tgtatgtggt	3	CD088917	39	2	
3495	tgtatgtggt	3	CD088917	39	0	
3495	tgtatgtggt	3	CD085302	0	1	
3495	tgtatgtggt	3	CD129000	96	0	homologue to SP|P12796|EGG2_SCHMA Eggshell protein precursor (Chorion protein). [Blood fluke] {Schistosoma mansoni}, partial (47%)
3496	tacagttatt	3	TC10027	55	0	
3497	tacgtgtgat	3				no_annot
3498	gggaaaagta	3	TC10925	92	1	weakly similar to GP|467528|dbj|BAA01185.1||D10354 alanine aminotransferase {Rattus norvegicus}, partial (64%)
3499	agtaccgttt	3				no_annot
3500	tacagctaag	3	C610986.1	96	0	
3501	tttacgtacc	3	TC16735	87	1	weakly similar to GP|6048361|gb|AAF02213.1|AF067202_1|AF067202 lactate dehydrogenase B4 {Danio rerio}, partial (69%)
3502	aaatagtatc	3	CD083349	82	0	
3503	atggtatcct	3	TC8297	83	0	weakly similar to SP|O76924|ARI2_DROME Ariadne-2 protein (Ari-2). [Fruit fly] {Drosophila melanogaster}, partial (46%)
3504	ggtgagggca	3				no_annot
3505	tttcaacggt	3	TC15795	92	0	weakly similar to PIR|T14302|T14302 glycine-rich cell wall protein - carrot (fragment), partial (28%)
3506	tattccatac	3				no_annot
3507	gttgtttcaa	3				no_annot
3508	aacaaatcat	3				no_annot
3509	ttcatacaaa	3				no_annot

3510	ataacgccta	3	TC13463	87	0	weakly similar to SP|Q24740|CC37_DROVI Hsp90 co-chaperone Cdc37 (Hsp90 chaperone protein kinase-targeting subunit). [Fruit fly] {Drosophila virilis}, partial (46%)
3510	ataacgccta	3	TC13467	76	0	PIR|B54525|B54525 major female-specific polypeptide (frame 2) - fluke (Schistosoma mansoni) (fragment), partial (48%)
3511	cattaaacga	3				no_annot
3512	aagtcaggat	3	C200944.1	38	0	
3512	aagtcaggat	3	C603345.1	37	0	
3513	tctcatacca	3				no_annot
3514	taaacggatt	3				no_annot
3515	gaatctcaca	3	TC7555	7	2	similar to SP|P47825|T2D3_DROME Transcription initiation factor TFIID 110 kDa subunit (P110) (TAFII-110) (110 kDa TBP-associated factor). [Fruit fly] {Drosophila melanogaster}, partial (4%)
3516	ctgagacgat	3				no_annot
3517	tgcaatgctt	3	TC15814	22	2	weakly similar to GP|20306404|gb|AAH28501.1||BC028501 melanocyte prolifeating gene 1 {Mus musculus}, partial (23%)
3518	gggaacacca	3				no_annot
3519	atcaagaccc	3	TC10451	65	2	weakly similar to GP|14165239|gb|AAK55444.1|AF380350_1|AF380350 interferon regulatory factor 10 {Gallus gallus}, partial (19%)
3520	ggctccagta	3	TC10220	24	1	
3520	ggctccagta	3	TC18264	17	1	
3520	ggctccagta	3	BG930994	38	1	
3520	ggctccagta	3	CD181435	82	0	GP|8101501|gb| cytochrome P450 2K5 {Oncorhynchus mykiss}, partial (2%)
3521	aaatacgacg	3				no_annot
3522	cttattctat	3	C601498.1	73	1	
3523	ttttacgctc	3	TC8137	97	0	
3524	taccgggttt	3	TC16523	29	0	weakly similar to SP|P50408|VATF_RAT Vacuolar ATP synthase subunit F(V-ATPase F subunit) (Vacuolar proton pump F subunit) (V-ATPase 14 kDa subunit). [Rat] {Rattus norvegicus}, partial (76%)

3525	tgtacgtcgt	3				no_annot
3526	acaacgacca	3				no_annot
3527	gtgtacgatt	3				no_annot
3528	ggatttctga	3				no_annot
3529	caaatctgtt	3				no_annot
3530	gtaaacttgg	3	TC18052	67	5	
3530	gtaaacttgg	3	CD066600	2	0	
3531	atacattggt	3	TC11032	89	0	
3532	tgggctgagt	3	TC8642	91	0	
3533	aactcgtttg	3	TC13606	29	2	homologue to GP|27530990|dbj|BAC54557.1||AB073635 histone 2B {Drosophila yakuba}, partial (91%)
3534	caatattgtt	3	TC11590	6	0	GP|3892189|gb|AAC78304.1||AF064593 ubiquitin/ribosomal fusion protein {Schistosoma japonicum}, complete
3535	tccttcttta	3				no_annot
3536	gtttattgtc	3				no_annot
3537	cattttcata	3	CD201104	77	1	
3537	cattttcata	3	CD114226	21	2	
3538	ttactatctg	3	TC12392	31	3	
3539	tgattgcata	3				no_annot
3540	tgaacacagc	3	TC13998	68	0	
3541	tcttttgtta	3				no_annot
3542	aagaagaaac	3	CD060710	27	0	
3543	ttatacttac	3	TC18826	91	0	
3544	cctgtaacct	3				no_annot
3545	tcgccgtcta	3				no_annot
3546	tccaccagaa	3	TC17333	77	0	similar to GP|28410503|emb|CAD66889.1||BX251410 50s ribosomal protein l22 {Tropheryma whipplei TW08/27}, partial (15%)
3547	taaacaagtc	3				no_annot
3548	tatcaaaaag	3				no_annot
3549	aactctgttg	3	TC8607	71	0	

3549	aactctgttg	3	CD081983	73	0	
3550	ctatcaaaaa	3	CD131086	69	0	
3551	ttcttcctgg	3	TC8856	54	0	similar to SP|Q13630|FCL_HUMAN GDP-L-fucose synthetase(FX protein) (Red cell NADP(H)- binding protein) (GDP-4-keto-6-deoxy-D-mannose-3,5-epimerase-4-reductase). [Human] {Homo sapiens}, partial (85%)
3552	ataaggcagc	3				no_annot
3553	atgtaatggt	3				no_annot
3554	tggcaattca	3				no_annot
3555	taaaagtcgt	3	AY698060|Sma.5386	46	1	TLL (TLL)
3556	ttccgttggt	3				no_annot
3557	tgcctgtttt	3				no_annot
3558	atgcaattga	3	AA559829	85	0	
3559	atcgacatcg	3	TC8169	20	2	homologue to SP|P52798|EFA4_HUMAN Ephrin-A4 precursor (EPH-related receptor tyrosine kinase ligand 4) (LERK-4). [Human] {Homo sapiens}, partial (6%)
3559	atcgacatcg	3	TC9506	70	0	
3559	atcgacatcg	3	CD076480	37	1	
3560	tacattttta	3	TC16698	67	0	GP|160960|gb|AAA29867.1||M86397 epidermal growth factor receptor {Schistosoma mansoni}, complete
3560	tacattttta	3	AI976700	55	1	epidermal growth factor receptor
3561	ttagggtaac	3				no_annot
3562	aattttgcat	3				no_annot
3563	gttctccgat	3				no_annot
3564	ttatatttgc	3				no_annot
3565	atggattcca	3	TC15432	19	0	similar to GP|17945742|gb|AAL48919.1||AY071297 RE32705p {Drosophila melanogaster}, partial (15%)
3566	gtgctggaag	3				no_annot
3567	tgcacgcaat	3	AI976881	18	0	
3568	ttgattggga	3				no_annot

3569	ttctttcttg	3	TC10574	75	1	weakly similar to SP|P12695|ODP2_YEAST Dihydrolipoamide acetyltransferase component of pyruvate dehydrogenase complex, mitochondrial precursor(E2) (PDC-E2). [Baker's yeast] {Saccharomyces cerevisiae}, partial (18%)
3570	gtatccattt	3				no_annot
3571	tatcgaatat	3				no_annot
3572	aatatctata	3	TC14298	92	0	weakly similar to GP|16648450|gb|AAL25490.1||AY060451 LP09416p {Drosophila melanogaster}, partial (30%)
3573	aggtatatct	3	TC7681	47	0	
3574	cataacacgt	3				no_annot
3575	tcttttgtca	3				no_annot
3576	ttactatcag	3				no_annot
3577	tacaagaaaa	3	TC8152	80	0	weakly similar to GP|27817927|dbj|BAC55691.1||AP004275 P0453E05.1 {Oryza sativa (japonica cultivar-group)}, partial (30%)
3578	attttgggag	3	TC17875	68	0	
3579	tcaggcgggg	3	TC7110	59	2	similar to GP|6715146|gb|AAF26302.1|AF184617_1|AF184617 proprotein convertase aPC6C isoform {Branchiostoma californiense}, partial (3%)
3579	tcaggcgggg	3	TC7242	30	2	similar to GP|6715146|gb|AAF26302.1|AF184617_1|AF184617 proprotein convertase aPC6C isoform {Branchiostoma californiense}, partial (3%)
3579	tcaggcgggg	3	CD060844	34	0	similar to GP|6715146|gb|AAF26302.1|AF184617_1|AF184617 proprotein convertase aPC6C isoform {Branchiostoma californiense}, partial (3%)
3580	cacgcacgca	3				no_annot
3581	actatttggc	3				no_annot
3582	ctttgctata	3				no_annot
3583	ctttaaggat	3				no_annot
3584	aaggaaagtc	3	TC7625	78	0	weakly similar to SP|Q9QXK3|CPG2_MOUSE Coatomer gamma-2 subunit (Gamma-2 coat protein) (Gamma-2 COP). [Mouse] {Mus musculus}, partial (13%)
3585	tttgtttgat	3	TC17698	33	3	

3586	agccaaccaa	3	C607425.1	33	0	
3587	aatattaata	3				no_annot
3588	cccctaagtt	3				no_annot
3589	tcaaacaatc	3	CD084087	23	0	
3590	aacctttatt	3	TC11958	92	0	weakly similar to SP|Q07994|NCPR_MUSDO NADPH-cytochrome P450 reductase(CPR) (P450R). [House fly] {Musca domestica}, partial (16%)
3591	cacaatgatc	3	AY392155|Sma.1212	43	8	Nicotinic acetylcholine receptor non-alpha subunit precursor
3592	tcctggatgc	3				no_annot
3593	gtttgacgcc	3				no_annot
3594	tcttccgaaa	3				no_annot
3595	gaatagtgat	3	AJ312106|Sma.810	70	2	Cathepsin B endopeptidase (cb2 gene)
3596	gacgacctct	3	TC17453	77	1	similar to SP|Q18421|GRPE_CAEEL GrpE protein homolog, mitochondrial precursor. {Caenorhabditis elegans}, partial (27%)
3597	cgttcagacg	3	TC14729	70	2	similar to GP|15291531|gb|AAK93034.1||AY051610 GH25564p {Drosophila melanogaster}, partial (16%)
3598	gtcttgctta	3				no_annot
3599	tgacatttta	3	C208405.1	44	5	
3599	tgacatttta	3	C600526.1	42	5	
3600	tacagtgttc	3	TC14527	62	1	similar to GP|28564890|gb|AAO32529.1||AY144966 YPL105C {Saccharomyces castellii}, partial (4%)
3600	tacagtgttc	3	CD147085	52	2	
3601	caattcaacc	3	TC9198	15	0	similar to EGAD|113431|121573 guanylate kinase {Caenorhabditis elegans}, partial (27%)
3602	tttacagcta	3	TC8042	75	2	
3603	tttcttttcc	3	TC10734	24	2	
3604	gtgtatgaat	3				no_annot
3605	aataaagaga	3	TC19370	54	1	
3606	gattacagat	3	TC18182	78	0	
3607	acaatgatcg	3	CD110831	10	1	
3608	tacacattac	3	TC9158	83	0	
3609	tgcaatccaa	3				no_annot

3610	tacaatgagc	3	TC11715	65	1	
3611	ttattgatgt	3	TC17178	33	0	similar to SP|P47756|CAPB_HUMAN F-actin capping protein beta subunit (CapZ beta). [Human] {Homo sapiens}, partial (78%)
3611	ttattgatgt	3	CD145193	38	2	
3612	atgccttgtg	3	C610823.1	94	0	
3613	ttgtatgtta	3				no_annot
3614	tcacgcgtat	3	TC17285	53	1	homologue to GP|2636680|gb|AAC06263.1||U66331 pol {Schistosoma mansoni}, partial (8%)
3614	tcacgcgtat	3	AW061426	74	0	
3615	tataatacca	3				no_annot
3616	tcaacgcttt	3	TC17649	17	0	similar to GP|15010742|gb|AAK74030.1||AY045672 At1g54320/F20D21_50 {Arabidopsis thaliana}, partial (6%)
3617	tatacactac	3	TC15521	56	0	similar to GP|27263154|emb|CAD59446.1||AJ535316 structural maintenance of chromosomes protein 3 {Xenopus laevis}, partial (14%)
3618	aaactaattt	3	AI018926	68	0	homologue to GP|160996|gb|AAA16243.1||L09549 glyceraldehyde-3-phosphate dehydrogenase {Schistosoma japonicum}, partial (9%)
3618	aaactaattt	3	CD065377	56	0	homologue to SP|P20287|G3P_SCHMA Glyceraldehyde 3-phosphate dehydrogenase(GAPDH) (Major larval surface antigen) (P-37). [Blood fluke] {Schistosoma mansoni}, partial (20%)
3618	aaactaattt	3	AI977709	41	1	SP|P20287|G3P_SCHMA Glyceraldehyde 3-phosphate dehydrogenase(GAPDH) (Major larval surface antigen) (P-37). [Blood fluke] {Schistosoma mansoni}, partial (37%)
3619	tatccttcac	3				no_annot
3620	gggttaccat	3	TC17964	66	0	
3621	taaacttcca	3	TC11013	32	0	
3622	ctcataagac	3	TC13835	57	0	weakly similar to SP|P28072|PSB6_HUMAN Proteasome subunit beta type 6 precursor(Proteasome delta chain) (Macropain delta chain) (Multicatalytic endopeptidase complex delta chain) (Proteasome subunit Y). [Human] {Homo sapiens}, partial (79%)
3623	gatcattttg	3				no_annot
3624	atgatttcat	3				no_annot

3625	tttggttaat	3	C200529.1	58	1	
3625	tttggttaat	3	C604622.1	56	1	
3626	gtatcttttg	3	TC10499	94	0	SP|P42638|TPM2_SCHMA Tropomyosin 2 (TMII). [Blood fluke] {Schistosoma mansoni}, partial (47%)
3627	atgaattgtc	3	TC6922	38	2	
3628	tattccactt	3				no_annot
3629	aacccataat	3	CD123473	6	2	similar to GP|2636680|gb|AAC06263.1||U66331 pol {Schistosoma mansoni}, partial (15%)
3630	tcggaggcaa	3				no_annot
3631	tcagttattg	3				no_annot
3632	gctcgtgggc	3	TC10534	41	1	weakly similar to SP|P49165|RL4_URECA 60S ribosomal protein L4 (L1). [Innkeeper worm, Spoonworm] {Urechis caupo}, partial (59%)
3632	gctcgtgggc	3	TC10536	59	0	similar to PIR|JC4277|JC4277 ribosomal protein L4, cytosolic [validated] - rat, partial (51%)
3633	gtgttggatt	3	CD178442	4	0	
3634	tgaaagtcga	3				no_annot
3635	gtggcgatta	3				no_annot
3636	caactgttac	3				no_annot
3637	tatgtttgtc	3	TC19220	83	0	
3638	tgttctttac	3	TC7535	25	5	similar to GP|3986285|dbj|BAA34993.1||AB017002 DjVLGA {Dugesia japonica}, partial (56%)
3638	tgttctttac	3	TC7536	88	0	weakly similar to GP|3986285|dbj|BAA34993.1||AB017002 DjVLGA {Dugesia japonica}, partial (22%)
3639	ctgatgatct	3				no_annot
3640	gaaacaaaca	3				no_annot
3641	gcatattttg	3				no_annot
3642	gtaaacactc	3				no_annot
3643	ttagcgaagt	3	TC17591	65	0	weakly similar to GP|16769542|gb|AAL28990.1||AY061442 LD38070p {Drosophila melanogaster}, partial (29%)
3644	gtcgttagca	3				no_annot
3645	gtacagacag	3	TC17517	73	0	

3646	agcatcagtt	3	C302138.1	4	0	
3646	agcatcagtt	3	C611965.1	4	0	
3647	ggagccagaa	3	TC6881	13	2	similar to GP|12654267|gb|AAH00954.1|AAH00954|BC000954 heterochromatin-like protein 1 {Homo sapiens}, partial (61%)
3647	ggagccagaa	3	TC6881	13	1	similar to GP|12654267|gb|AAH00954.1|AAH00954|BC000954 heterochromatin-like protein 1 {Homo sapiens}, partial (61%)
3648	taaaagaata	3	TC18742	93	0	weakly similar to SP|P43090|HEM0_OPSTA 5-aminolevulinic acid synthase, erythroid-specific, mitochondrial precursor(Delta-aminolevulinate synthase) (Delta-ALA synthetase) (ALAS-E). [Oyster toadfish] {Opsanus tau}, partial (30%)
3649	cagacaggta	3	CD113500	56	0	
3649	cagacaggta	3	CD178873	64	0	
3650	acaacagtga	3				no_annot
3651	ttcactgtgt	3	CD087993	13	1	
3652	atgttgtcga	3				no_annot
3653	tagacaaagt	3				no_annot
3654	aataatttcc	3				no_annot
3655	taatgagtag	3	TC12348	65	0	similar to GP|20270909|gb|AAM18471.1|AF483532_1|AF483532 VHSV-induced protein-6 {Oncorhynchus mykiss}, partial (15%)
3656	acaacgaagt	3	CD164915	25	1	similar to GP|28950825|gb|AAO63336.1||BT005272 At1g74430 {Arabidopsis thaliana}, partial (6%)
3656	acaacgaagt	3	CD076960	23	0	similar to GP|11230872|dbj|BAB18102.1||AB034969 cyclomaltodextrin binding protein {Thermococcus sp. B1001}, partial (5%)
3657	taaacaatta	3				no_annot
3658	gaaaggaacc	3	TC9143	84	0	similar to GP|20905053|gb|AAM30355.1||AE013290 GDP-mannose 4,6 dehydratase {Methanosarcina mazei Goe1}, partial (59%)
3659	tagaagcctc	3				no_annot
3660	ataagaatca	3	CD075035	8	1	

3661	atactgattt	3				no_annot
3662	ctgatgttga	3	TC10750	39	1	similar to GP|17862736|gb|AAL39845.1||AY069700 LD46935p {Drosophila melanogaster}, partial (27%)
3663	caatggaaca	3				no_annot
3664	gaaaggaaaa	3	TC11672	14	0	weakly similar to GP|7303100|gb|AAF58167.1||AE003811 CG8090-PA {Drosophila melanogaster}, partial (44%)
3664	gaaaggaaaa	3	TC13700	81	0	similar to SP|O60832|DKC1_HUMAN Dyskerin (Nucleolar protein NAP57) (CBF5 homolog). [Human] {Homo sapiens}, partial (60%)
3665	gttacaattg	3				no_annot
3666	actgttcggg	3				no_annot
3667	agggcagtgt	3	AI394925	10	2	
3668	tttgtacata	3	TC7529	96	0	
3668	tttgtacata	3	TC17126	37	2	similar to SP|P38344|YB9M_YEAST 45.8 kDa protein in SHM1-MRPL37 intergenic region. [Baker's yeast] {Saccharomyces cerevisiae}, partial (5%)
3669	attattctta	3				no_annot
3670	acgattcagt	3	C201383.1	23	3	
3670	acgattcagt	3	C610185.1	23	3	
3671	caatttggag	3				no_annot
3672	cacaatcaga	3	TC11285	70	0	weakly similar to GP|7299193|gb|AAF54390.1||AE003683 CG9378-PA {Drosophila melanogaster}, partial (49%)
3673	ttgagtcagc	3				no_annot
3674	ttgaacactc	3	C603312.1	26	1	
3675	taaaagggtt	3				no_annot
3676	tgttgtacat	3	TC17214	91	0	similar to SP|P09789|GRP1_PETHY Glycine-rich cell wall structural protein 1 precursor. [Petunia] {Petunia hybrida}, partial (7%)
3677	catttctgct	3				no_annot
3678	agtatattcg	3	TC11247	92	0	
3679	tttgagaaag	3	TC19223	95	0	
3680	tgtacgtaca	3				no_annot

3681	ccgaaagttt	3	TC10719	67	0	similar to SP|Q39644|LEA5_CITSI Late embryogenesis abundant protein Lea5. [Sweet orange] {Citrus sinensis}, partial (23%)
3682	ataagcaaaa	3				no_annot
3683	ttcacatcat	3				no_annot
3684	ttgtgaaggt	3				no_annot
3685	cgttgttcgt	3				no_annot
3686	aaaatcagtg	3				no_annot
3687	ccccaaccct	3				no_annot
3688	tttttacata	3				no_annot
3689	ttataaaggt	3				no_annot
3690	catcagaaac	3	C611003.1	33	4	
3691	ggggtagact	3	TC14591	29	4	
3692	gtaactgatt	3				no_annot
3693	aagatttctc	3				no_annot
3694	tattcttctc	3	AW017279	67	2	tetraspanin
3695	ccagctaatc	3	C208236.1	36	2	
3695	ccagctaatc	3	C603935.1	29	3	
3696	atggattagc	3	TC15184	64	2	
3697	actttgtttc	3	TC9203	66	0	
3698	tattatgcat	3				no_annot
3699	tattgattat	3	TC13637	83	0	weakly similar to GP|20151533|gb|AAM11126.1||AY094773 GM14501p {Drosophila melanogaster}, partial (73%)
3700	acggctgtca	3				no_annot
3701	taatgactaa	3	TC16812	38	1	homologue to GP|14588595|dbj|BAB61794.1||AB063181 calmodulin {Metridium senile}, complete
3701	taatgactaa	3	TC16813	34	2	GP|4160167|emb|CAA10601.1||AJ132193 calmodulin {Caenorhabditis elegans}, complete
3701	taatgactaa	3	CD179197	9	0	similar to GP|1883001|emb|CAA62809.1||X91509 histone H4 {Apis mellifera}, partial (17%)
3702	gtctgttatt	3	TC10971	78	0	weakly similar to GP|3063649|gb|AAC97476.1||AF057286 intersectin-EH binding protein Ibp2 {Mus musculus}, partial (7%)

3702	gtctgttatt	3	CD167858	57	1	
3703	agtcctttgg	3	C200387.1	78	0	
3703	agtcctttgg	3	C603989.1	80	0	
3704	ctactataga	3				no_annot
3705	atcaatgaca	3	CD077879	68	0	GP|490332|emb|CAA00285.1||A03654 fusion protein c11/p28 {synthetic construct}, partial (30%)
3706	tgaacttcag	3				no_annot
3707	cctttaaaat	3	TC7962	76	0	
3707	cctttaaaat	3	TC10386	69	2	similar to GP|20177041|gb|AAM12282.1||AY095189 LD26817p {Drosophila melanogaster}, partial (30%)
3708	atagcaaaat	3				no_annot
3709	catatatagt	3				no_annot
3710	gtaaaatgga	3	TC14055	67	0	weakly similar to GP|7296323|gb|AAF51613.1||AE003591 CG6020-PA {Drosophila melanogaster}, partial (25%)
3711	attccttcaa	3				no_annot
3712	gttttgttta	3	TC12469	90	0	weakly similar to GP|10726700|gb|AAF56017.2||AE003739 CG5315-PB {Drosophila melanogaster}, partial (14%)
3713	atcttccttg	3				no_annot
3714	ttgcggagaa	3				no_annot
3715	tatatataca	3	TC17325	92	0	similar to GP|20073320|gb|AAH27061.1||BC027061 Recql protein {Mus musculus}, partial (17%)
3715	tatatataca	3	AA999403	10	2	
3716	taaagcgtat	3				no_annot
3717	tcttcggtac	3				no_annot
3718	tataataatc	3	TC8436	85	0	
3719	atcactaaat	3	TC7958	97	0	similar to GP|17945868|gb|AAL48980.1||AY071358 RE39465p {Drosophila melanogaster}, partial (9%)
3720	tttggtgtgt	3				no_annot
3721	aatatatctg	3				no_annot
3722	gaaaggcgaa	3				no_annot

3723	gagctttcgg	3	TC17883	8	3	similar to SP|Q9P0J6|RM36_HUMAN 60S ribosomal protein L36, mitochondrial precursor (L36mt) (BRCA1-interacting protein 1). [Human] {Homo sapiens}, partial (27%)
3724	gatgcaaaaa	3	TC10448	88	0	
3724	gatgcaaaaa	3	TC17300	4	0	
3725	ggaatttagg	3	TC7361	73	0	similar to PIR|T39903|T39903 serine-rich protein - fission yeast (Schizosaccharomyces pombe), partial (7%)
3726	taaaaactat	3	TC16455	20	2	
3726	taaaaactat	3	TC17483	19	0	
3726	taaaaactat	3	TC19553	69	0	
3726	taaaaactat	3	CD128141	89	0	
3726	taaaaactat	3	CD170943	73	1	
3727	aaccttgtac	3	C200067.1	17	1	
3727	aaccttgtac	3	C602098.1	16	1	
3728	ggaattggag	3	TC13733	61	1	
3729	tgtcagggtg	3	L46884|Sma.252	33	1	Cyclophilin
3730	tatattgcac	3	CD134268	20	3	
3731	tgttgatttg	3	TC7793	77	2	unknown
3731	tgttgatttg	3	TC12823	73	0	
3732	cacaacaggt	3				no_annot
3733	cacacatctt	3				no_annot
3734	ttcgcttgtc	3	C201887.1	43	0	
3735	cttcaagcga	3	TC8562	39	0	
3736	aaaaaaaaac	3	TC18969	35	2	
3737	gaaatatctc	3				no_annot
3738	attagtaggc	3	CD166946	70	0	
3739	tatacagcag	3				no_annot
3740	tacgtatatg	3				no_annot
3741	taattgttag	3				no_annot
3742	gtacagaata	3	TC17773	58	1	weakly similar to GP|1907386|gb|AAC53130.1||U91538 vesicle trafficking protein sec22b {Mus musculus}, partial (58%)

3743	caaagaacaa	3	TC17299	52	0	similar to GP|20151605|gb|AAM11162.1||AY094809 LD29185p {Drosophila melanogaster}, partial (98%)
3744	aacaagaaga	3	M64539|Sma.624	66	1	Ferritin-2
3745	ctggaaaagt	3	TC17570	68	0	similar to GP|21064785|gb|AAM29622.1||AY113617 RH65663p {Drosophila melanogaster}, partial (51%)
3746	gaggaggtgc	3				no_annot
3747	tgaattttta	3				no_annot
3748	gaatgatgtt	3	TC8214	64	0	
3749	aaaactacta	3				no_annot
3750	tataagtgac	3				no_annot
3751	ttttctaaaa	3				no_annot
3752	ttatgttgtt	3				no_annot
3753	ccatcgtgat	3	C201387.1	22	3	
3753	ccatcgtgat	3	C609801.1	22	3	
3754	atcatcacta	3	TC7493	63	0	
3754	atcatcacta	3	CD127227	1	2	
3755	actctaggtg	3	TC17720	13	0	similar to SP|P55769|NHPX_HUMAN NHP2-like protein 1 (High mobility group-like nuclear protein 2 homolog 1) ([U4/U6.U5] tri-snRNP 15.5 kDa protein) (OTK27). [Human] {Homo sapiens}, partial (84%)
3756	tacatttcgc	3	TC11867	93	0	
3757	atacaatgtt	3	C301497.1	42	0	
3757	atacaatgtt	3	C606057.1	42	0	
3758	ctggaaaaca	3	CD120410	29	1	SP|P08418|HS70_SCHMA Heat shock 70 kDa homolog protein (HSP70) (Major surface antigen). [Blood fluke] {Schistosoma mansoni}, partial (18%)
3758	ctggaaaaca	3	CD111891	37	0	SP|P08418|HS70_SCHMA Heat shock 70 kDa homolog protein (HSP70) (Major surface antigen). [Blood fluke] {Schistosoma mansoni}, partial (8%)
3759	aattcacaaa	3	CD063970	77	0	
3760	gtttgtcaag	3				no_annot
3761	ctccaaacat	3				no_annot
3762	ttctcctgat	3				no_annot
3763	aatatatcag	3	TC14082	97	0	

3764	tagatgttat	3	TC9749	34	1	
3765	cagtatcttc	3	TC11400	62	0	weakly similar to GP|19572248|emb|CAD27772.1||AL596140 AK001373_like {Tetraodon nigroviridis}, partial (8%)
3766	ctattcgctc	3				no_annot
3767	ttgttcagat	3				no_annot
3768	cagtaatcag	3				no_annot
3769	gaacagtcag	3				no_annot
3770	ctggtttgta	3	TC9769	84	0	similar to GP|2734866|gb|AAB96360.1||U72484 RNA helicase {Takifugu rubripes}, partial (26%)
3770	ctggtttgta	3	TC16902	92	0	similar to GP|22946759|gb|AAF53680.2||AE003659 CG10333-PA {Drosophila melanogaster}, partial (59%)
3771	gatggactgt	3	TC15488	68	0	
3771	gatggactgt	3	TC19654	67	2	weakly similar to GP|18044822|gb|AAH20002.1||BC020002 cDNA sequence BC020002 {Mus musculus}, partial (3%)
3772	acctttataa	3	TC13479	92	1	homologue to PIR|B54525|B54525 major female-specific polypeptide (frame 2) - fluke (Schistosoma mansoni) (fragment), partial (95%)
3773	tgacttctgc	3				no_annot
3774	caattatcgt	3				no_annot
3775	gtttaggaaa	3	TC11048	85	0	weakly similar to GP|26449182|dbj|BAC41721.1||AB086192 Translocating chain-associated membrane protein {Gallus gallus}, partial (8%)
3776	tgctcactgt	3				no_annot
3777	gattaacaga	3	TC18022	59	0	
3778	agctcgacga	3	TC13331	23	2	
3779	aacacattgt	3				no_annot
3780	ttgataaata	3	TC19137	29	4	homologue to SP|P35813|P2CA_HUMAN Protein phosphatase 2C alpha isoform(PP2C-alpha) (IA) (Protein phosphatase 1A). [Human] {Homo sapiens}, partial (25%)
3781	ggaagccagt	3	CD059681	39	1	
3781	ggaagccagt	3	CD059681	39	0	
3782	taataatgtg	3				no_annot

3783	tggaggcgtg	3				no_annot
3784	aaagtgtggc	3	C210912.1	9	3	
3784	aaagtgtggc	3	C318643.1	90	0	
3784	aaagtgtggc	3	C607169.1	8	3	
3785	gatatacatc	3				no_annot
3786	aacgctccac	3				no_annot
3787	atccataatc	3				no_annot
3788	aaatttaatc	3	TC16752	94	0	
3789	aggactgtga	3				no_annot
3790	gcaatcactt	3				no_annot
3791	gagttatgta	3	TC13661	87	0	similar to GP|13436359|gb|AAH04964.1|AAH04964|BC004964 oxoglutarate dehydrogenase (lipoamide) {Homo sapiens}, partial (70%)
3792	tagcaatact	3				no_annot
3793	tacagataca	3				no_annot
3794	ctcttcgatt	3				no_annot
3795	tgctgtagtt	3	TC12708	40	0	weakly similar to SP|P16026|SODE_SCHMA Extracellular superoxide dismutase [Cu-Zn] precursor(EC-SOD). [Blood fluke] {Schistosoma mansoni}, partial (38%)
3796	agcagtggaa	3				no_annot
3797	atcaacagtt	3	AI977779	70	0	
3798	aacatacaaa	3	TC13972	89	0	weakly similar to GP|16769424|gb|AAL28931.1||AY061383 LD30731p {Drosophila melanogaster}, partial (77%)
3798	aacatacaaa	3	TC14847	44	1	
3799	ttgatgtctt	3				no_annot
3800	ggttgagtta	3				no_annot
3801	tgaatacaat	3				no_annot
3802	aagttcccaa	3				no_annot
3803	gaaaggcttt	3	TC14737	81	0	similar to GP|12017959|gb|AAG45339.1|AF312865_1|AF312865 C1orf28 {Homo sapiens}, partial (4%)

3804	acaaaaatag	3				no_annot
3805	cctacatatg	3	C604748.1	32	0	
3806	taataaagag	3	TC18652	96	0	
3807	ttataaatgc	3	TC12515	91	0	similar to GP|17946329|gb|AAL49204.1||AY071582 RE63764p {Drosophila melanogaster}, partial (15%)
3808	agttatcttt	3	CD135028	35	0	
3809	ggtcttgttg	3	C200693.1	69	0	
3809	ggtcttgttg	3	C200782.1	40	1	
3809	ggtcttgttg	3	C201929.1	48	2	
3809	ggtcttgttg	3	C201929.1	48	0	
3809	ggtcttgttg	3	C300093.1	33	0	
3809	ggtcttgttg	3	C301822.1	71	0	
3809	ggtcttgttg	3	C610715.1	11	7	
3809	ggtcttgttg	3	C610715.1	11	3	
3809	ggtcttgttg	3	C610715.1	11	0	
3809	ggtcttgttg	3	C707556.1	71	0	
3809	ggtcttgttg	3	C717014.1	33	0	
3810	ttattaaaaa	3				no_annot
3811	aacaaactcg	3				no_annot
3812	ttggtttgcc	3	CD149221	48	0	
3813	ttgtgaataa	3				no_annot
3814	ggtgggaagg	3				no_annot
3815	gtaatagggt	3	C317193.1	34	1	
3815	gtaatagggt	3	C609828.1	34	1	
3816	gtgaaataaa	3				no_annot
3817	gtattttgca	3				no_annot
3818	tccccgtcat	3				no_annot
3819	tacagtgcca	3	C601595.1	5	4	
3820	tattggatct	3				no_annot
3821	atttggtgca	3	TC17366	80	0	

3822	tactataatt	3	TC8008	98	0	weakly similar to PIR|A45601|A45601 22K antigen - fluke (Schistosoma mansoni), partial (27%)
3823	aacaatgaaa	3	CD069635	4	3	
3823	aacaatgaaa	3	CD184852	39	0	
3824	ggtgatatat	3	TC8321	98	0	
3825	tccccataca	3				no_annot
3826	tatatgtatt	3	TC11693	91	0	
3826	tatatgtatt	3	AW330554	3	1	
3827	tatatgtatg	3				no_annot
3828	aacgcgtatg	3				no_annot
3829	atattcgtct	3	CD191752	75	0	
3830	acaattgcga	3				no_annot
3831	aagtgtatgt	3				no_annot
3832	ttattgtcat	3				no_annot
3833	tttgctgttt	3	TC14470	62	0	
3834	tattgtgttt	3	C200926.1	90	0	
3834	tattgtgttt	3	C606259.1	90	0	
3835	aggagataga	3				no_annot
3836	gaaaaatgca	3				no_annot
3837	aaatacttca	3	AF521090|Sma.82	2	0	Clone p33F5 unknown mRNA
3838	ctgtgcaaac	3				no_annot
3839	tttactttcg	3				no_annot
3840	gctttacaga	3	TC17587	55	0	
3841	tgtaataaga	3				no_annot
3842	atgataactg	3				no_annot
3843	atgataacta	3				no_annot
3844	ctgtgattct	3				no_annot
3845	gtaaacaaaa	3	CD093359	57	3	
3846	atgatgatca	3	TC10792	45	1	weakly similar to PIR|T39903|T39903 serine-rich protein - fission yeast (Schizosaccharomyces pombe), partial (7%)
3847	gtaggtggat	3	C610974.1	4	2	

3848	acccttacac	3				no_annot
3849	agcgtgtttt	3	TC8846	96	0	
3850	aattaaaagc	3				no_annot
3851	acttattccc	3	R95585	88	0	similar to GP|7298505|gb|AAF53724.1||AE003661 CG10679-PA {Drosophila melanogaster}, partial (62%)
3852	gcccccttt	3				no_annot
3853	gtaacagcga	3				no_annot
3854	ctgtatcctt	3				no_annot
3855	caatgtagtc	3	TC8510	67	0	weakly similar to PIR|JC7117|JC7117 ubiquitin carboxy-terminal hydrolase-6- chicken, partial (37%)
3856	cattgatctc	3	TC13107	60	1	
3856	cattgatctc	3	TC15112	79	0	PIR|A49957|A49957 CD45-associated 30k phosphoprotein - mouse, partial (6%)
3856	cattgatctc	3	CD198709	67	0	similar to GP|16267034|dbj NADH dehydrogenase subunit 2 {Crenimugil crenilabis}, partial (3%)
3857	tttgtatgcg	3	CD140495	47	0	
3858	atgtttcata	3	TC8070	67	0	weakly similar to GP|19354431|gb|AAH24401.1||BC024401 LOC232210 protein {Mus musculus}, partial (9%)
3859	atgctcagca	3				no_annot
3860	cagactgaga	3				no_annot
3861	aagttgaatc	3	L37100|Sma.21	91	0	NF-YA subunit
3862	acagtagaag	3				no_annot
3863	atatttgtgt	3				no_annot
3864	tagaggctgt	3				no_annot
3865	gatttatctc	3				no_annot
3866	actatgcaac	3	TC7108	66	7	
3866	actatgcaac	3	TC7108	66	3	
3866	actatgcaac	3	CD153646	15	4	
3866	actatgcaac	3	CD060168	35	1	GP|10176|emb|CAA29285.1||X05842 eggshell precursor protein {Schistosoma mansoni}, partial (24%)
3867	tgtttggcac	3				no_annot
3868	aagtgtttct	3				no_annot

3869	cattgaaccc	3	CD081824	64	3	
3870	atagaaaggg	3				no_annot
3871	aaatgacttg	3				no_annot
3872	acatcaatta	3	TC11062	96	0	
3872	acatcaatta	3	CD084264	84	0	
3873	cgaacttaat	3				no_annot
3874	tgctaatttc	3	C210172.1	75	0	
3875	ttccaaaatg	3	C601686.1	88	0	
3876	aaaatttttt	3	CD148458	9	1	similar to GP|28317134|gb|AAO39584.1||BT003580 LD24589p {Drosophila melanogaster}, partial (40%)
3877	cgtttttttg	3	C201117.1	12	1	
3877	cgtttttttg	3	C610867.1	11	1	
3878	ataaatcagc	3	TC16382	44	2	
3879	attgaggggt	3	CD162534	89	0	weakly similar to SP|O67613|YH18_AQUAE Maf-like protein AQ_1718. {Aquifex aeolicus}, partial (15%)
3880	agtcatcctc	3	C301509.1	47	0	
3880	agtcatcctc	3	C601563.1	50	0	
3881	atttccaatc	3				no_annot
3882	aatctacata	3	TC17217	89	0	similar to SP|Q9GP36|ERH_ECHMU Enhancer of rudimentary homolog. [Pork tapeworm] {Taenia solium}, complete
3883	tctttgtaat	3				no_annot
3884	ccatcgttcc	3				no_annot
3885	ataacccgaa	3				no_annot
3886	tgcgacaaac	3	C200465.1	40	6	
3887	tcactattct	3				no_annot
3888	tatcgaaagt	3				no_annot
3889	aagcttcaaa	3	TC13768	36	0	similar to GP|16648004|gb|AAL25267.1||AY060228 GH01635p {Drosophila melanogaster}, partial (79%)
3890	atttagaaga	3	TC12131	80	0	similar to GP|7296948|gb|AAF52221.1||AE003608 CG8680-PA {Drosophila melanogaster}, partial (39%)
3891	taggttaggg	3	TC16678	67	4	SP|P20287|G3P_SCHMA Glyceraldehyde 3-phosphate dehydrogenase(GAPDH) (Major larval surface antigen) (P-37). [Blood fluke] {Schistosoma mansoni}, complete

3891	taggttaggg	3	AI976252	21	3	SP|P20287|G3P_SCHMA Glyceraldehyde 3-phosphate dehydrogenase(GAPDH) (Major larval surface antigen) (P-37). [Blood fluke] {Schistosoma mansoni}, partial (39%)
3892	atagttctgt	3	C200019.1	80	0	
3892	atagttctgt	3	C610095.1	80	0	
3893	aaaaaagtat	3	CD144077	30	4	
3894	aagcggcagt	3				no_annot
3895	ttgtgttact	3				no_annot
3896	atggtggtgg	3				no_annot
3897	ccgcctagtg	3				no_annot
3898	atgatatcgt	3				no_annot
3899	acaaatcgtc	3	TC7772	55	0	weakly similar to GP|6007612|gb|AAF00976.1|AF116341_1|AF116341 poly(A)-binding protein II {Drosophila melanogaster}, partial (44%)
3900	gaaataggtg	3	C202265.1	15	9	
3900	gaaataggtg	3	C611330.1	13	9	
3901	aaattcgttg	3				no_annot
3902	gtgattattt	3	TC17498	32	2	
3903	tttaaaaata	3	TC13934	97	0	similar to PIR|C96954|C96954 permease, probable chloride channel [imported] - Clostridium acetobutylicum, partial (4%)
3904	taaaggacaa	3	TC19751	60	0	
3905	aaaacaatat	3	C309093.1	0	0	
3905	aaaacaatat	3	C700114.1	0	0	
3906	ctagtcgatc	3	TC12496	38	0	homologue to GP|21928439|dbj|BAC05810.1||AB065580 seven transmembrane helix receptor {Homo sapiens}, partial (7%)
3907	ttgaataaaa	3				no_annot
3908	ttatttccct	3				no_annot
3909	taagaaataa	3	TC7032	12	3	
3910	acaaagattc	3	AI394892	73	1	
3911	ccctatttta	3	AA999350	17	0	

3912	tcacatatgt	3	AI374468	14	1	
3913	actccttgat	3	TC14234	10	0	weakly similar to GP|11177032|dbj|BAB17854.1||AB042616 cytochrome b5 {Ciona savignyi}, partial (57%)
3914	aacataaggt	3	TC11100	79	0	
3915	ttgctcactt	3				no_annot
3916	tccaaagttg	3				no_annot
3917	ttgctcactg	3				no_annot
3918	tcacttaaaa	3				no_annot
3919	tgataatgaa	3	TC17235	69	0	homologue to GP|1813565|gb|AAB41699.1||U72709 7B2 {Lymnaea stagnalis}, partial (6%)
3920	taccctttcc	3	TC8966	82	0	
3921	cagccatttg	3	C206035.1	49	0	
3922	ttccgatttt	3	TC7975	19	0	
3923	cgcaacattc	3				no_annot
3924	gtatggtttc	3	AW497747	20	1	
3925	aatatataca	3	AF071011|Sma.779	84	0	Myosin light chain
3926	gcgcttatgg	3	TC14177	54	0	weakly similar to GP|29290598|emb|CAD83056.1||AL732594 bN189G18.1.1 (otholog of human PRP4 pre-mRNA processing factor 4 homolog (yeast) PRPF4) {Mus musculus}, partial (46%)
3927	actttaggct	3				no_annot
3928	tcgacatcgt	3				no_annot
3929	ttatagattt	3				no_annot
3930	aaattgatca	3	CD072031	5	2	
3930	aaattgatca	3	CD076896	48	0	similar to SP|Q02169|MAF_BACSU Septum formation protein Maf. {Bacillus subtilis}, partial (23%)
3931	gttgacaaaa	3				no_annot
3932	tgtatgaaac	3				no_annot
3933	gatggcagct	3				no_annot
3934	ttttgataca	3				no_annot
3935	aatccgtgga	3	TC11173	63	0	
3936	ttcacagtgg	3	M86396|Sma.850	89	3	Epidermal growth factor receptor (SER, class 1 product)

3937	taataaatac	3				no_annot
3938	taccgttcta	3				no_annot
3939	tagaaatttg	3				no_annot
3940	caattatagt	3				no_annot
3941	aaataggatt	3				no_annot
3942	agtgaaggac	3				no_annot
3943	catattcagt	3				no_annot
3944	gtatggatat	3				no_annot
3945	tcatattggt	3				no_annot
3946	tgtaagataa	3	CD166119	38	0	
3947	aaaacctaaa	3				no_annot
3948	atgaagcact	3				no_annot
3949	tgatattcat	3				no_annot
3950	ttccaataag	3				no_annot
3951	atgattctca	3	TC12304	82	0	
3952	catacacagt	3	CD185362	59	2	
3953	ttgtcaatat	3				no_annot
3954	ccatctctga	3				no_annot
3955	gtatcattat	3	TC16965	64	5	weakly similar to GP|2274968|emb|CAA04006.1||AJ000332 Glucosidase II {Homo sapiens}, partial (43%)
3956	ttgtttttgg	3	U30265|Sma.7	80	0	Cyclophylin-like protein trans-spliced
3956	ttgtttttgg	3	gi|1245401|gb|U50388.1|SMU50388	72	0	Schistosoma mansoni cyclophilin A mRNA, complete cds
3957	cgttatcatc	3	TC14025	59	1	similar to GP|3800793|gb|AAC68871.1||AF072242 methyl-CpG binding protein MBD2 {Homo sapiens}, partial (10%)
3958	ccgccttgcc	3	TC11947	69	0	
3959	aaagcagtat	3				no_annot
3960	gatggtaggt	3	CD137062	93	0	homologue to GP|11994449|dbj cytochrome P450 {Arabidopsis thaliana}, partial (2%)
3961	gactatcatt	3	TC10509	57	0	
3961	gactatcatt	3	TC10512	31	1	

3962	aattttattg	3	TC11288	70	0	weakly similar to GP|3875796|emb|CAA97788.1||Z73425 C. elegans SEC-24.1 protein (corresponding sequence F12F6.6) {Caenorhabditis elegans}, partial (9%)
3962	aattttattg	3	TC14218	71	0	homologue to PIR|S03282|HSCH2F histone H2A.F, embryonic - chicken, partial (95%)
3963	gtactttctg	3				no_annot
3964	ctgtagtaca	3	TC16913	53	2	GP|1016750|gb|AAA79138.1||U35432 rab-related GTP-binding protein {Schistosoma mansoni}, complete
3965	tcaaaataca	3	TC11527	2	2	
3965	tcaaaataca	3	TC16374	22	0	
3966	aatcgcctca	3	TC17467	51	0	weakly similar to GP|17065286|gb|AAL32797.1||AY062719 SUDD-like protein {Arabidopsis thaliana}, partial (31%)
3967	taatacggaa	3	CD152318	30	0	
3968	tgaccttgta	3				no_annot
3969	aattgtcgtg	3	TC11187	94	0	similar to GP|12001996|gb|AAG43141.1|AF061730_1|AF061730 My027 protein {Homo sapiens}, partial (57%)
3970	ggcagtatcc	3				no_annot
3971	atggatgctt	3				no_annot
3972	tccccgtacc	3				no_annot
3973	aatgcatttg	3				no_annot
3974	ccacaacaac	3				no_annot
3975	aataataaaa	3	TC17567	14	0	
3975	aataataaaa	3	CD132812	17	0	
3976	atctcctgct	3				no_annot
3977	gttggttttg	3				no_annot
3978	tttgtattct	3				no_annot
3979	ggctagaagg	3				no_annot
3980	ataacaacct	3				no_annot
3981	ccagttaatc	3	TC11548	35	1	
3982	gtctagctga	3	C210171.1	25	1	
3982	gtctagctga	3	C600260.1	39	0	
3983	tgtattgaat	3				no_annot

3984	taaatgtgtt	3				no_annot
3985	cattgaaata	3				no_annot
3986	tgtaaaacag	3				no_annot
3987	cttacaagaa	3	TC17925	76	0	
3987	cttacaagaa	3	CD146881	78	0	
3988	agcgctgaaa	3	TC16777	8	8	homologue to SP|P16026|SODE_SCHMA Extracellular superoxide dismutase [Cu-Zn] precursor(EC-SOD). [Blood fluke] {Schistosoma mansoni}, partial (96%)
3989	tcattcaagg	3	TC7561	79	0	similar to GP|22164073|gb|AAM93547.1|AF412216_1|AF412216 polyprotein {Schistosoma japonicum}, partial (39%)
3989	tcattcaagg	3	CD202468	4	4	
3989	tcattcaagg	3	CD085776	21	0	similar to GP|22164073|gb|AAM93547.1|AF412216_1|AF412216 polyprotein {Schistosoma japonicum}, partial (35%)
3989	tcattcaagg	3	CD152996	2	2	similar to GP|19067879|gb|AAK14815.1||AY027869 polyprotein {Schistosoma japonicum}, partial (12%)
3989	tcattcaagg	3	CD178280	22	1	similar to GP|22164073|gb|AAM93547.1|AF412216_1|AF412216 polyprotein {Schistosoma japonicum}, partial (75%)
3990	cacattcgaa	3	C202063.1	21	3	
3991	taatataacc	2				no_annot
3992	cgaaggacat	2				no_annot
3993	ggaagttgga	2	TC6877	8	2	
3994	tatctattct	2	BF440117	49	1	
3995	caacccataa	2				no_annot
3996	gacagcactg	2				no_annot
3997	cttagattct	2	C200320.1	93	1	
3998	tatgtttcat	2				no_annot
3999	cagcaccacc	2	C200942.1	5	3	
4000	caatataacg	2				no_annot
4001	agcgcccaaa	2				no_annot
4002	gaatcaatta	2				no_annot
4003	ataatttcct	2				no_annot

4004	tgcaaacatt	2	C612498.1	87	0	
4005	cggctagacg	2				no_annot
4006	gtacgatatg	2				no_annot
4007	aattagaagc	2	C317275.1	16	0	
4008	cactccttgt	2				no_annot
4009	ttataaacgt	2	C605978.1	14	3	
4010	agccaattct	2	AI974942	33	2	
4011	aggtagatag	2				no_annot
4012	gttgctagca	2				no_annot
4013	atgcccaatt	2				no_annot
4014	cattagctca	2	TC13880	73	2	weakly similar to SP|Q13614|MTR2_HUMAN Myotubularin-related protein 2. [Human] {Homo sapiens}, partial (38%)
4015	gctggaaaat	2	TC17525	69	0	similar to GP|2253101|gb|AAB62870.1||AF006658 beta-glucosidase {Bacteroides fragilis}, partial (4%)
4016	ggatcctccc	2				no_annot
4017	ttcactgaag	2	TC14333	66	0	
4018	tacattgttg	2	TC14758	83	0	
4019	aactgcattt	2	TC13555	70	3	
4020	gtttgtatgg	2				no_annot
4021	tgcccacagc	2				no_annot
4022	tttgagcctg	2				no_annot
4023	aacagataca	2				no_annot
4024	taatcctgag	2	C302117.1	63	0	
4024	taatcctgag	2	C705338.1	64	0	
4025	tcaatcattg	2	TC15536	36	2	
4026	ggcatagtgt	2				no_annot
4027	ctattactcg	2	TC9347	81	0	weakly similar to GP|12958620|gb|AAK09377.1|AF321816_1|AF321816 hepatic glucose transporter GLUT2 {Oncorhynchus mykiss}, partial (10%)
4028	gtgaagcgcg	2				no_annot
4029	gtgaagcgca	2				no_annot

4030	gatacatcgt	2				no_annot
4031	tattctgtat	2				no_annot
4032	cgtgtatgta	2	CD188360	27	0	
4033	gtggcagtaa	2	TC14412	5	4	
4034	tacacagtta	2	CD096977	33	2	
4035	tttgcaaact	2	TC13812	86	0	similar to GP|4966317|gb|AAA88778.2||U46856 vitellogenin {Anolis pulchellus}, partial (3%)
4036	gaaagtatag	2				no_annot
4037	tggcttataa	2				no_annot
4038	agacattcga	2				no_annot
4039	gcacgaagtt	2	C209095.1	59	0	
4039	gcacgaagtt	2	C603372.1	58	0	
4040	tatgtaattt	2				no_annot
4041	ggtcagtgtc	2	C210791.1	65	0	
4042	accagcaatg	2	C209723.1	60	0	
4042	accagcaatg	2	C600584.1	60	0	
4043	tccgttgtga	2	TC10489	74	4	weakly similar to SP|P51989|RO21_XENLA Heterogeneous nuclear ribonucleoprotein A2 homolog 1 (hnRNP A2(A)). [African clawed frog] {Xenopus laevis}, partial (60%)
4043	tccgttgtga	2	TC10490	82	1	
4044	atgagttggg	2				no_annot
4045	tgatacaaga	2				no_annot
4046	ctcgaaaaag	2				no_annot
4047	cgataaatat	2	TC13591	81	2	similar to GP|3153910|gb|AAC17451.1||AF066859 muscle glycogen phosphorylase {Homo sapiens}, partial (91%)
4048	ttcaattcag	2				no_annot
4049	gagcagcatt	2				no_annot
4050	tttggagtat	2				no_annot
4051	ttgtcttact	2				no_annot
4052	ggacaactga	2	TC14229	50	0	
4053	aaatatacgt	2				no_annot

4054	tggccgtgta	2	TC11034	69	0	
4055	tgatacaact	2	C604765.1	28	2	
4056	tggataaaaa	2				no_annot
4057	taaaacttaa	2	TC13536	82	2	weakly similar to PIR|A55075|A55075 chaperonin-10 - mouse, partial (97%)
4058	ctccttattt	2				no_annot
4059	ttggtggaga	2	TC8198	69	4	similar to GP|26000552|gb|AAN75454.1||AY151155 Na-dependent Cl/HCO3 exchanger {Loligo pealei}, partial (24%)
4060	atcatttaaa	2	TC14643	70	0	
4060	atcatttaaa	2	CD189286	70	0	
4061	ctatctatgc	2	CD076960	6	1	similar to GP|11230872|dbj|BAB18102.1||AB034969 cyclomaltodextrin binding protein {Thermococcus sp. B1001}, partial (5%)
4062	tcatcatata	2	TC13345	50	1	
4063	caaacaaagg	2				no_annot
4064	tacgcacaac	2				no_annot
4065	atatacaagg	2				no_annot
4066	gagtaacagc	2	TC7959	83	0	similar to GP|21623729|dbj|BAC00945.1||AB072486 HT-A protein {Lycopersicon hirsutum}, partial (18%)
4066	gagtaacagc	2	CD088106	58	0	
4067	tgtgtttcta	2	C600143.1	30	0	
4068	ttgctttcat	2	TC10751	97	0	weakly similar to GP|4490377|emb|CAB38634.1||AJ133649 actin related complex p41 subunit {Drosophila melanogaster}, partial (34%)
4069	tttgggaaag	2				no_annot
4070	ccgtgcttaa	2				no_annot
4071	tggtggagaa	2				no_annot
4072	agtaactacg	2				no_annot
4073	gataaaccca	2				no_annot
4074	cataagacgc	2				no_annot
4075	gtagtatgtt	2	TC11816	37	2	
4076	atactagtat	2				no_annot

4077	taacctttgg	2				no_annot
4078	aaacacggga	2				no_annot
4079	cataaatatc	2	CD080201	71	0	
4079	cataaatatc	2	AW146508	48	1	
4080	taaaggaagt	2				no_annot
4081	ttgtaggtta	2	C205729.1	86	0	
4082	accactacta	2				no_annot
4083	aaataatgat	2	CD157937	16	0	
4084	atataattgt	2				no_annot
4085	atagattatc	2				no_annot
4086	atagattata	2	TC14527	85	0	similar to GP|28564890|gb|AAO32529.1||AY144966 YPL105C {Saccharomyces castellii}, partial (4%)
4087	gttcgcacac	2	C201431.1	87	0	
4088	ttctatgcta	2				no_annot
4089	tacttggttg	2				no_annot
4090	aagaattctg	2	TC18339	68	0	
4091	ttttcaaaat	2	TC13802	95	0	weakly similar to SP|Q9DCD0|6PGD_MOUSE 6-phosphogluconate dehydrogenase, decarboxylating. [Mouse] {Mus musculus}, partial (91%)
4092	acataggtaa	2				no_annot
4093	gacgtttcta	2				no_annot
4094	tgtctaatct	2				no_annot
4095	tttattattg	2	AF074400|Sma.114	99	0	Calcium ATPase 2 (SMA2)
4096	taaatccggt	2				no_annot
4097	gggtacatca	2				no_annot
4098	aaatgcagca	2				no_annot
4099	gctacaagtt	2	TC11245	82	1	similar to GP|7297705|gb|AAF52957.1||AE003628 CG5322-PA {Drosophila melanogaster}, partial (5%)
4100	ggaaatggtt	2	CD133358	62	0	weakly similar to GP|28381101|gb|AAF52110.2||AE003605 CG18271-PA {Drosophila melanogaster}, partial (24%)
4101	gaatctaata	2	CD069579	84	0	
4102	atacttgata	2	C203119.1	81	0	

4102	atacttgata	2	C602222.1	83	1	
4103	gagaagaaaa	2	TC7796	16	6	
4104	gtaattcagt	2				no_annot
4105	ttcaaaattg	2	C209556.1	68	1	
4105	ttcaaaattg	2	C306434.1	72	1	
4105	ttcaaaattg	2	C607907.1	61	2	
4106	cttctaacaa	2				no_annot
4107	aaaatctcga	2	TC12470	78	1	
4108	ttcgtttggt	2				no_annot
4109	gacacacaca	2	TC17510	16	5	
4110	tttacaattt	2				no_annot
4111	tttgaacaca	2				no_annot
4112	tagcgtctag	2	TC12300	29	0	
4113	ccaattattg	2				no_annot
4114	gatgccagga	2	TC18233	3	3	similar to PIR|T03949|T03949 serine-tRNA ligaseserS - maize (fragment), partial (8%)
4115	tattattgct	2				no_annot
4116	aggacgaatt	2	C200042.1	23	1	
4116	aggacgaatt	2	C605453.1	22	1	
4117	cactcacacg	2	C611884.1	13	2	
4118	ctcactcact	2	TC10981	82	0	weakly similar to GP|22324206|emb|CAC82992.1||AJ344147 eIF2B-beta protein {Drosophila melanogaster}, partial (7%)
4119	tacaacaccc	2				no_annot
4120	cagcaggtgt	2	TC10773	92	0	
4120	cagcaggtgt	2	CD144187	43	0	
4121	aaacataagg	2				no_annot
4122	gattaattgt	2				no_annot
4123	gcattgtata	2	U55992|Sma.893	34	1	Dynein light chain (SmDLC)
4124	tttatggccc	2				no_annot
4125	tttatggcca	2				no_annot

4126	ggagagggtg	2	TC16858	13	0	similar to GP|3220183|gb|AAC23561.1||AF049130 ADP/ATP carrier {Trypanosoma brucei brucei}, partial (57%)
4127	acagacagct	2				no_annot
4128	aatatgtaga	2	TC12314	25	0	homologue to GP|15290619|gb|AAK94914.1||AF400155 maturase K {Arenaria tetraquetra}, partial (9%)
4129	acggggctaa	2				no_annot
4130	tatactactt	2	TC19381	28	3	
4131	gaaatagtaa	2	CD086358	56	2	
4131	gaaatagtaa	2	CD162304	83	0	
4132	cgatttccta	2				no_annot
4133	gttgcctttg	2				no_annot
4134	taaaatttcc	2				no_annot
4135	gagtttcgca	2				no_annot
4136	tcatcgtcac	2				no_annot
4137	tcgtgtttat	2				no_annot
4138	ggatagaata	2	TC9340	89	0	weakly similar to GP|10177013|dbj|BAB10201.1||AB023032 contains similarity to CHP-rich zinc finger protein~gene_id:K5J14.8 {Arabidopsis thaliana}, partial (3%)
4139	cttttttaaa	2				no_annot
4140	ccaatggccc	2				no_annot
4141	gaaatgaaaa	2	AY653161|Sma.263	52	0	Glycerol 3-phosphate dehydrogenase
4142	ataataataa	2	TC9762	5	1	
4142	ataataataa	2	TC18014	86	0	homologue to GP|2289237|gb|AAB64410.1||U95301 calcium-dependent group X phospholipase A2 {Homo sapiens}, partial (8%)
4142	ataataataa	2	CD122413	39	0	
4143	atgtgtaacg	2	TC17418	35	1	similar to GP|22759353|gb|AAF59336.2||AE003843 CG1970-PA {Drosophila melanogaster}, partial (62%)
4144	tataaatacg	2				no_annot
4145	agtagtatct	2	TC14735	23	2	

4146	tcactatcat	2	CD129601	12	0	homologue to PIR|T18403|T18 asparagine/aspartate rich protein - malaria parasite (Plasmodium falciparum), partial (0%)
4147	gctactactg	2				no_annot
4148	gattaattac	2				no_annot
4149	ctgactttgt	2				no_annot
4150	aaagctcaac	2				no_annot
4151	tactgatgag	2	TC11485	73	0	weakly similar to GP|16648426|gb|AAL25478.1||AY060439 LD46868p {Drosophila melanogaster}, partial (16%)
4152	tatgttaata	2				no_annot
4153	acctttaatt	2	TC8096	97	0	similar to SP|P45985|MPK4_HUMAN Dual specificity mitogen-activated protein kinase kinase 4(MAP kinase kinase 4) (JNK activating kinase 1) (c-Jun N- terminal kinase kinase 1) (JNKK) (SAPK/ERK kinase 1) (SEK1). [Human] {Homo sapiens}, partial (57%)
4154	atttcatcta	2	TC8891	33	2	
4154	atttcatcta	2	TC11180	98	0	
4154	atttcatcta	2	CD090913	0	2	similar to GP|11034538|dbj|BAB17062.1||AP002523 P0013F10.8 {Oryza sativa (japonica cultivar-group)}, partial (4%)
4155	tttattatgt	2	TC18786	1	5	
4156	ggaagcttca	2				no_annot
4157	tgtggaaaac	2	TC11759	85	1	similar to GP|7290709|gb|AAF46156.1||AE003437 CG3842-PA {Drosophila melanogaster}, partial (18%)
4157	tgtggaaaac	2	CD150119	5	0	
4158	tgatgagaaa	2				no_annot
4159	ttagtcagtg	2	TC14826	88	0	
4160	atctagttac	2				no_annot
4161	atagctagga	2				no_annot
4162	caatcggctt	2				no_annot
4163	tggattcctg	2				no_annot
4164	ggagaagtaa	2				no_annot

4165	cgtttatgat	2	TC17114	88	0	weakly similar to SP|P49915|GUAA_HUMAN GMP synthase [glutamine-hydrolyzing](Glutamine amidotransferase) (GMP synthetase). [Human] {Homo sapiens}, partial (36%)
4166	ctatgatggt	2	CD128209	37	4	
4167	gtcggagtta	2	CD073028	57	0	weakly similar to GP|21618729|gb|AAH31544.1||BC031544 Facl5 protein {Mus musculus}, partial (9%)
4168	ctacgattgg	2				no_annot
4169	tagggaacac	2				no_annot
4170	cgactactat	2				no_annot
4171	ttgttatgtg	2	TC8749	7	2	
4172	aactatggct	2				no_annot
4173	gtgatatgta	2				no_annot
4174	attctattca	2				no_annot
4175	atatataaat	2	TC8616	77	0	
4176	ttctatgcag	2				no_annot
4177	atgagtattc	2	TC8165	78	0	weakly similar to GP|21645201|gb|AAF46707.2||AE003453 CG30390-PA {Drosophila melanogaster}, partial (34%)
4178	ggaagacatt	2				no_annot
4179	acatttaccc	2	TC9074	41	4	weakly similar to SP|P34529|DCR1_CAEEL Endoribonuclease dcr-1. {Caenorhabditis elegans}, partial (4%)
4180	attttatgat	2				no_annot
4181	tggttcatcg	2	C206222.1	11	1	
4181	tggttcatcg	2	C600420.1	11	1	
4182	tacaagcaga	2				no_annot
4183	atggtcttgc	2				no_annot
4184	tgtctttatg	2	X77211|Sma.27	79	2	IMP25 mRNA
4185	gcattgtaga	2	TC14887	56	0	
4185	gcattgtaga	2	CD113507	74	0	
4186	cgtttttcgt	2				no_annot
4187	ccaacaatgt	2				no_annot

4188	tccctaggcc	2				no_annot
4189	gactagctca	2				no_annot
4190	aattgatcaa	2	CD064057	49	0	homologue to GP|7291633|gb|AAF47056.1||AE003461 CG12491-PA {Drosophila melanogaster}, partial (8%)
4191	gagaaactaa	2				no_annot
4192	tacagctgta	2				no_annot
4193	atatcttgtt	2				no_annot
4194	gttttgtaac	2				no_annot
4195	tgaagcgata	2	CD187144	5	1	
4196	aaactagttg	2				no_annot
4197	gtatagctgg	2	TC8373	46	1	
4198	tttaagtagc	2				no_annot
4199	ctcaccagtt	2				no_annot
4200	tgtcattttg	2				no_annot
4201	gaaactattc	2				no_annot
4202	taaatgtttg	2				no_annot
4203	atacccttag	2				no_annot
4204	taaagtgaaa	2				no_annot
4205	tcacatacgc	2	TC12617	60	0	
4206	gaactttcga	2	AA517945	40	0	
4206	gaactttcga	2	CD083164	76	1	
4207	tatgcatatt	2	TC19634	46	1	similar to GP|1736829|dbj|BAA15973.1||D90848 Histidine-rich glycoprotein precursor. {Escherichia coli}, partial (5%)
4208	cgttattgtg	2				no_annot
4209	tacctggcta	2	TC7591	97	0	SP|P30669|GBAS_SCHMA Guanine nucleotide-binding protein G(S), alpha subunit (Adenylate cyclase-stimulating G alpha protein). [Blood fluke] {Schistosoma mansoni}, complete
4210	ttggctgtga	2				no_annot
4211	ctcagggctg	2				no_annot
4212	ttaaaatgga	2				no_annot
4213	gttatgtttt	2	TC14618	36	0	

4213	gttatgtttt	2	TC14664	68	0	
4214	tttttgtttg	2	TC10842	60	0	similar to GP|10047435|gb|AAG12241.1||AF183938 GDI {Giardia intestinalis}, partial (6%)
4215	tgcgcacaca	2				no_annot
4216	tcacgctgtt	2	CD082475	43	0	
4217	ccaatgccgt	2				no_annot
4218	ataagcgata	2	CD073564	49	0	similar to GP|21064395|gb|AAM29427.1||AY113422 RE19904p {Drosophila melanogaster}, partial (24%)
4219	tcaactaatg	2				no_annot
4220	aacgagtttt	2	TC13197	58	0	weakly similar to SP|O95777|LSM8_HUMAN U6 snRNA-associated Sm-like protein LSm8. [Human] {Homo sapiens}, partial (65%)
4221	atacatcttc	2	TC14205	43	0	homologue to GP|1377882|gb|AAB02564.1||U58359 orf205 {Chlorobium vibrioforme}, partial (6%)
4221	atacatcttc	2	TC18530	80	0	weakly similar to SP|P19338|NUCL_HUMAN Nucleolin (Protein C23). [Human] {Homo sapiens}, partial (4%)
4222	gtggctcaca	2	AF056330|Sma.773	62	1	AUT1 (AUT1)
4223	accgatttgt	2	TC13234	95	0	
4223	accgatttgt	2	CD074376	67	2	
4224	aatatgcgcg	2	TC18257	37	3	
4225	tgacgatgta	2	TC17476	52	0	
4226	tccaagcttt	2				no_annot
4227	gaaaatgtga	2	TC7159	92	3	weakly similar to SP|O88761|PSD1_RAT 26S proteasome non-ATPase regulatory subunit 1 (26S proteasome regulatory subunit S1) (26S proteasome subunit p112). [Rat] {Rattus norvegicus}, partial (65%)
4227	gaaaatgtga	2	TC14483	49	1	
4228	atttgagggt	2				no_annot
4229	ataaattttt	2				no_annot
4230	ttcaaattag	2				no_annot
4231	gtaacggatt	2				no_annot
4232	gaaaaaacac	2				no_annot
4233	agcaacattt	2				no_annot
4234	ttttgccaac	2	CD078403	83	0	

4235	ataaccgcgt	2	TC10858	54	1	similar to SP|P23232|GBB_LOLFO Guanine nucleotide-binding protein beta subunit. [Northern European squid] {Loligo forbesi}, complete
4236	ctaaagcaat	2				no_annot
4237	tagtgatcga	2	C209280.1	83	1	
4238	atgacttgta	2				no_annot
4239	gtgtgttgtg	2	TC7373	87	0	similar to GP|24659630|gb|AAH39185.1||BC039185 Tcerg1 protein {Mus musculus}, partial (3%)
4239	gtgtgttgtg	2	TC7376	51	1	weakly similar to GP|24659630|gb|AAH39185.1||BC039185 Tcerg1 protein {Mus musculus}, partial (22%)
4240	aacgtgaaaa	2	TC17017	63	1	similar to GP|17945779|gb|AAL48937.1||AY071315 RE33866p {Drosophila melanogaster}, partial (42%)
4240	aacgtgaaaa	2	TC17018	62	1	weakly similar to GP|17945779|gb|AAL48937.1||AY071315 RE33866p {Drosophila melanogaster}, partial (23%)
4241	tgatgtttac	2	C700860.1	37	0	
4242	tagatactgt	2				no_annot
4243	tacttagcat	2				no_annot
4244	gctttattat	2				no_annot
4245	tttacattaa	2	TC14782	28	1	
4246	ggatttttgt	2				no_annot
4247	aaagtataag	2	TC11208	81	0	
4248	ggctaggtct	2				no_annot
4249	atgttcttcc	2	TC11912	81	0	
4250	aacaatataa	2	AA999380	17	0	
4251	gtttatgtga	2	TC17336	37	0	
4251	gtttatgtga	2	CD124434	39	2	
4252	cggaattaat	2				no_annot
4253	gcttgtccat	2				no_annot
4254	taccatccag	2				no_annot
4255	atcataacct	2	TC7989	80	0	weakly similar to GP|16768252|gb|AAL28345.1||AY060797 GH26345p {Drosophila melanogaster}, partial (23%)

4256	tcccactacc	2				no_annot
4257	taccgttact	2				no_annot
4258	ggattgccgc	2				no_annot
4259	ataattaaac	2	CD061713	33	1	
4260	ttgttatgag	2				no_annot
4261	acaagaatta	2	TC7979	16	1	similar to GP|5921645|gb|AAD56281.1|AF137264_1|AF137264 glycine decarboxylase p protein {Anas platyrhynchos}, partial (5%)
4262	gtggacaata	2				no_annot
4263	ataagagtga	2				no_annot
4264	catagtaact	2				no_annot
4265	tagcctgtcg	2				no_annot
4266	tctccctacc	2				no_annot
4267	ctgctattca	2	C209179.1	86	0	
4268	ttaagtagtt	2	TC18542	40	1	
4269	gaaaacctgg	2	TC7314	3	5	homologue to GP|12248331|gb|AAG13157.2||AF216698 cytochrome c oxidase subunit 1 {Schistosoma mansoni}, partial (28%)
4269	gaaaacctgg	2	TC7325	54	0	
4269	gaaaacctgg	2	TC7327	69	0	
4269	gaaaacctgg	2	TC13386	71	0	
4269	gaaaacctgg	2	TC15792	89	0	
4269	gaaaacctgg	2	BE431303	63	0	
4269	gaaaacctgg	2	CD071790	8	1	
4269	gaaaacctgg	2	CD083581	58	0	
4269	gaaaacctgg	2	CD084422	67	0	
4269	gaaaacctgg	2	CD178738	81	0	
4269	gaaaacctgg	2	CD183222	78	0	
4269	gaaaacctgg	2	CD189458	82	0	
4269	gaaaacctgg	2	CD190175	75	0	
4269	gaaaacctgg	2	CD071742	84	0	

4269	gaaaacctgg	2	CD079198	73	1	
4269	gaaaacctgg	2	CD090074	78	0	
4269	gaaaacctgg	2	CD092870	62	1	
4269	gaaaacctgg	2	CD096454	68	1	
4269	gaaaacctgg	2	CD114357	76	0	
4269	gaaaacctgg	2	CD137193	46	0	
4269	gaaaacctgg	2	CD160715	72	1	
4269	gaaaacctgg	2	CD184963	20	0	
4269	gaaaacctgg	2	CD186244	48	0	
4269	gaaaacctgg	2	AI976121	55	0	repeat sequences
4270	tcattgatat	2	CD073082	1	0	
4271	acgctagtta	2	C210467.1	77	0	
4272	taacaatatt	2	TC8369	31	0	
4273	ctgggaaaat	2	TC17047	61	0	
4274	tgtgtaatga	2	C300772.1	70	0	
4274	tgtgtaatga	2	C602264.1	32	3	
4275	aaggcgcaaa	2	C210594.1	60	0	
4275	aaggcgcaaa	2	C603443.1	51	0	
4276	atgaattaac	2				no_annot
4277	aaagcagcgt	2				no_annot
4278	tgtaaaaaaa	2	C201016.1	13	3	
4278	tgtaaaaaaa	2	C609394.1	13	3	
4279	tcatttgttt	2				no_annot
4280	aaagactagg	2				no_annot
4281	agttatgact	2				no_annot
4282	tgattgaaga	2	TC10029	43	0	
4283	catccgaatg	2	TC10468	77	0	
4284	ataaatatac	2	N21941	13	0	
4284	ataaatatac	2	CD071544	6	1	
4285	tagaaaacaa	2	CD111706	7	2	

4286	tcccagtgaa	2				no_annot
4287	tgtatttgtg	2				no_annot
4288	tgtagaatgc	2				no_annot
4289	gccgaatagt	2	TC7621	74	1	similar to GP|16741554|gb|AAH16585.1||BC016585 UDP-N-acetyl-alpha-D-galactosamine:polypeptide N-acetylgalactosaminyltransferase 9 {Mus musculus}, partial (40%)
4290	ctgttttcgg	2				no_annot
4291	aagcttttgg	2				no_annot
4292	gaagacagtt	2	AI975650	20	0	
4293	ataattggat	2				no_annot
4294	tccccgctaa	2				no_annot
4295	aattcgtttg	2	CD133178	81	0	
4296	tgtgtaatat	2	TC17170	97	0	similar to EGAD|66062|69017 ras-related protein RAL-A {Drosophila melanogaster}, partial (30%)
4297	tctttctttt	2	TC17242	91	0	weakly similar to GP|20151713|gb|AAM11216.1||AY094863 RE24638p {Drosophila melanogaster}, partial (42%)
4298	tgcaaatgta	2				no_annot
4299	agcgatggaa	2	TC14579	70	2	
4300	tggccgtttg	2				no_annot
4301	aacaatttaa	2	C201355.1	15	3	
4302	gcgcattgtc	2				no_annot
4303	aagagcattt	2	TC8108	77	1	
4304	agacagtgac	2				no_annot
4305	atcacagtag	2	TC13034	41	2	
4306	gtaccgtcga	2				no_annot
4307	attaaagggc	2				no_annot
4308	gggaaccata	2				no_annot
4309	attcaacagt	2	TC14045	28	4	weakly similar to SP|P08621|RU17_HUMAN U1 small nuclear ribonucleoprotein 70 kDa (U1 snRNP 70 kDa) (snRNP70) (U1-70K). [Human] {Homo sapiens}, partial (17%)
4310	acgaatagcc	2				no_annot

4311	ttgtattgtt	2				no_annot
4312	aagggtaact	2				no_annot
4313	ggtcttgatg	2	TC18595	51	0	
4314	tgtttatttt	2	TC9111	74	2	
4315	gtaacaagtt	2	TC10750	22	2	similar to GP|17862736|gb|AAL39845.1||AY069700 LD46935p {Drosophila melanogaster}, partial (27%)
4316	ttgggataaa	2	TC14323	94	0	weakly similar to SP|P27393|CA24_ASCSU Collagen alpha 2(IV) chain precursor. [Pig roundworm, Ascaris lumbricoides] {Ascaris suum}, partial (4%)
4317	ataacatacg	2				no_annot
4318	gaaagatgta	2				no_annot
4319	agtgcttaat	2				no_annot
4320	ctgtgaaacg	2				no_annot
4321	tacttttgtt	2	TC17033	74	0	weakly similar to GP|5002178|gb|AAD37345.1|AF141864_1|AF141864 AtaAp {Emericella nidulans}, partial (23%)
4322	gcagttcaaa	2				no_annot
4323	ttcaatgggc	2				no_annot
4324	acagtctata	2				no_annot
4325	atgcaggtct	2				no_annot
4326	gttcaaaaaa	2				no_annot
4327	tcccgacatc	2				no_annot
4328	attataccgc	2				no_annot
4329	ggatatgaat	2				no_annot
4330	caaagtcaac	2	TC7463	60	0	similar to GP|19773876|gb|AAL98920.1||AF387339 Bat1 {Rattus norvegicus}, partial (17%)
4331	atcccttctt	2				no_annot
4332	ccgaaggata	2	TC13602	81	1	homologue to GP|27372732|gb|AAO06264.1||AY158954 histone protein Hist2h3c1 {Mus musculus}, partial (80%)
4333	aatgtatgct	2				no_annot
4334	cctgggatta	2	TC10602	43	3	similar to GP|5679074|gb|AAD46846.1|AF160906_1|AF160906 BcDNA.LD02793 {Drosophila melanogaster}, partial (73%)

4335	tgcctttgaa	2	TC13654	88	0	similar to PIR|I38968|I38968 100 kDa coactivator - human, partial (24%)
4336	atcaccgaat	2				no_annot
4337	gttggatgga	2	TC7125	83	2	weakly similar to GP|18413527|emb|CAD22045.1||AJ428992 glutamine synthetase {Amanita muscaria}, partial (12%)
4338	tactccacag	2				no_annot
4339	ccacgaccgc	2				no_annot
4340	gtaaaaaaaa	2				no_annot
4341	gaagaagcgg	2				no_annot
4342	tttcaaattg	2	TC16573	89	1	similar to GP|35830|emb|CAA40296.1||X56976 ubiquitin activating enzyme E1 {Homo sapiens}, partial (51%)
4343	ttagtaccaa	2				no_annot
4344	ttatgatttt	2	C605211.1	2	1	
4345	tgtagtaata	2				no_annot
4346	ctagaataac	2	C317087.1	21	0	
4347	aatgaacctt	2				no_annot
4348	tacatatatt	2	C209280.1	29	3	
4349	tgaagactga	2				no_annot
4350	attaaataat	2	TC15913	4	3	
4350	attaaataat	2	TC17266	69	0	similar to SP|P09234|RU1C_HUMAN U1 small nuclear ribonucleoprotein C (U1-C). [Human] {Homo sapiens}, partial (43%)
4351	tacatatata	2	TC7734	97	0	weakly similar to GP|10801574|dbj|BAB16700.1||AB041639 TIA-1 like protein {Bombyx mori}, partial (10%)
4351	tacatatata	2	CD155266	32	1	
4352	atgtgtgttt	2				no_annot
4353	gttttggtta	2	TC9535	38	0	weakly similar to GP|25988816|gb|AAN76273.1|AF453745_1|AF453745 proton/amino acid transporter 3 {Mus musculus}, partial (7%)
4354	tgactggtaa	2				no_annot
4355	ctgattagtg	2				no_annot
4356	ctgcatatcc	2				no_annot

4357	tgattgtaca	2	TC11284	76	0	
4357	tgattgtaca	2	TC16063	70	1	similar to GP|18652299|gb|AAL77055.1|AF467986_1|AF467986 ADP-ribosylation factor-like protein 3 {Xenopus laevis}, partial (98%)
4358	aagagagatg	2	TC15681	81	0	similar to GP|5869817|emb|CAB55574.1||AJ249395 cytochrome oxidase subunit III {Globodera pallida}, partial (9%)
4359	gaaattcaaa	2	AA528904	4	0	
4360	tgagatgtgt	2				no_annot
4361	taatcacact	2	TC8435	66	2	similar to SP|Q9QZB7|ARPB_MOUSE Actin-related protein 11. [Mouse] {Mus musculus}, partial (5%)
4362	cacatatatt	2				no_annot
4363	gttatcctgg	2	C200188.1	22	9	
4363	gttatcctgg	2	C200361.1	39	2	
4363	gttatcctgg	2	C300502.1	6	0	
4363	gttatcctgg	2	C300503.1	6	1	
4363	gttatcctgg	2	C301777.1	9	0	
4363	gttatcctgg	2	C302051.1	47	0	
4363	gttatcctgg	2	C601980.1	39	1	
4363	gttatcctgg	2	C602671.1	36	1	
4363	gttatcctgg	2	C704495.1	6	1	
4363	gttatcctgg	2	C707143.1	6	0	
4363	gttatcctgg	2	C714195.1	47	0	
4363	gttatcctgg	2	C702571.1	9	0	
4364	cgtgtattaa	2				no_annot
4365	tatatagaat	2				no_annot
4366	ttacttatgc	2				no_annot
4367	atgatgaact	2				no_annot
4368	ttgtgataac	2	C209951.1	64	0	
4368	ttgtgataac	2	C607924.1	63	0	
4369	tgttactttg	2	CD136988	93	0	
4370	acaagcaacg	2	TC8828	17	0	

4370	acaagcaacg	2	CD089264	51	1	
4371	tgttacttta	2	C604369.1	14	1	
4372	tggtatgaat	2				no_annot
4373	actactgata	2				no_annot
4374	gatttgaaaa	2				no_annot
4375	agattgtctc	2	TC6931	30	8	similar to GP|19919877|gb|AAM08414.1|AF493056_1|AF493056 MF3 protein {Schistosoma japonicum}, complete
4375	agattgtctc	2	TC6931	30	0	similar to GP|19919877|gb|AAM08414.1|AF493056_1|AF493056 MF3 protein {Schistosoma japonicum}, complete
4375	agattgtctc	2	CD168997	76	0	
4375	agattgtctc	2	CD168563	13	4	similar to GP|19919877|gb|AAM08414.1|AF493056_1|AF493056 MF3 protein {Schistosoma japonicum}, partial (37%)
4376	aatgctgtcc	2	TC16861	91	0	
4377	ttggtaatga	2	TC11157	81	0	similar to GP|6980078|gb|AAF34715.1|AF225902_1|AF225902 separation anxiety protein {Drosophila melanogaster}, partial (40%)
4378	tgatagttct	2	TC9439	15	2	
4379	tgacagacta	2	C609050.1	16	0	
4380	taagtcagtg	2				no_annot
4381	atgcggactg	2				no_annot
4382	gtgactctgg	2				no_annot
4383	cgataccttt	2				no_annot
4384	cgaagccagt	2	TC11129	79	0	weakly similar to GP|21428348|gb|AAM49834.1||AY118465 GM02257p {Drosophila melanogaster}, partial (31%)
4385	cataagaaga	2				no_annot
4386	ggtctactcg	2				no_annot
4387	ttacagaaat	2				no_annot
4388	tatttgattt	2	TC17244	86	0	
4389	tttgactcag	2				no_annot
4390	ctgaaaaaaa	2				no_annot

4391	tgcactgacc	2				no_annot
4392	gtgctcgagg	2	TC16748	35	1	similar to SP|O01666|ATPG_DROME ATP synthase gamma chain, mitochondrial precursor. [Fruit fly] {Drosophila melanogaster}, partial (23%)
4393	aacagtcatc	2				no_annot
4394	ttggtaatcg	2				no_annot
4395	cctgggatgc	2				no_annot
4396	tagattcaaa	2	TC7212	57	5	
4397	aattttacaa	2				no_annot
4398	cattctgcct	2				no_annot
4399	gtcaggatgt	2				no_annot
4400	gccggtaagt	2	TC18099	22	1	similar to GP|13542967|gb|AAH05671.1||BC005671 Dctn5 protein {Mus musculus}, partial (63%)
4401	taacctacgc	2				no_annot
4402	attctgatgc	2				no_annot
4403	tgtagctaac	2				no_annot
4404	cagctatttg	2	CD082435	14	1	
4405	atgccatctg	2	TC11093	51	0	similar to GP|2809420|gb|AAB97740.1||AF041254 translocase of inner mitochondrial membrane Tim44 precursor {Homo sapiens}, partial (24%)
4406	gttccttcta	2				no_annot
4407	tacctgccct	2				no_annot
4408	tacaaaatca	2	C201160.1	2	2	
4408	tacaaaatca	2	C604658.1	2	2	
4409	tatattatag	2				no_annot
4410	tctgcattgt	2				no_annot
4411	gatggcactg	2				no_annot
4412	ttaagttgat	2				no_annot
4413	tcgccaaggc	2				no_annot
4414	aacctcagga	2				no_annot
4415	gcgcacattt	2				no_annot
4416	cgttacatat	2				no_annot

4417	tatgtaacta	2	TC11052	82	0	similar to GP|5231255|gb|AAD41149.1|U87145_18|U87145 clp {Toxoplasma gondii}, partial (3%)
4417	tatgtaacta	2	TC15681	4	4	similar to GP|5869817|emb|CAB55574.1||AJ249395 cytochrome oxidase subunit III {Globodera pallida}, partial (9%)
4418	ggacccctcc	2				no_annot
4419	agaaatagca	2				no_annot
4420	aaatattagg	2				no_annot
4421	gcccctaatc	2				no_annot
4422	gacagctact	2				no_annot
4423	cataaggggt	2				no_annot
4424	aacattgttt	2	TC14804	74	0	
4425	ttatggagcc	2	CD084314	38	0	
4426	ttctcgagga	2				no_annot
4427	gataccccag	2	M27529|Sma.142	51	3	Superoxide dismutase
4428	gatttgtaaa	2	C314855.1	36	1	
4428	gatttgtaaa	2	C606836.1	21	3	
4429	ccaggctgtg	2				no_annot
4430	atgatgtaac	2				no_annot
4431	taaacgtaac	2	TC12967	76	0	
4432	cataacgaag	2				no_annot
4433	tgaggtactg	2				no_annot
4434	acagattttg	2	C200246.1	76	0	
4434	acagattttg	2	C600074.1	36	1	
4435	tgatttcatt	2				no_annot
4436	agcgaaactc	2				no_annot
4437	ggaagaagtg	2				no_annot
4438	gcctacccat	2	TC18176	5	3	similar to GP|3876636|emb|CAB04256.1||Z81525 C. elegans LET-858 protein (corresponding sequence F33A8.1) {Caenorhabditis elegans}, partial (3%)
4438	gcctacccat	2	CD131996	22	0	
4439	gaatatagtt	2				no_annot

4440	tgatatgtat	2	TC8885	84	1	
4441	ggtttagttt	2				no_annot
4442	tatacattgt	2	CD197087	3	1	
4442	tatacattgt	2	CD178302	19	1	
4443	tacaacaaac	2	TC13598	19	1	
4444	ttttactcat	2				no_annot
4445	ctaaactatg	2				no_annot
4446	atgatactga	2	TC13984	58	1	weakly similar to SP|Q02645|HTS_DROME Hu-li tai shao protein. [Fruit fly] {Drosophila melanogaster}, partial (7%)
4447	gtcatacaca	2	TC13379	4	0	weakly similar to SP|O76041|NEBL_HUMAN Nebulette (Actin-binding Z-disk protein). [Human] {Homo sapiens}, partial (5%)
4448	cgctttagtg	2	C611276.1	81	1	
4449	attagtaacc	2				no_annot
4450	tggatactat	2				no_annot
4451	ttaataggca	2	TC18903	6	2	homologue to GP|15529624|gb|AAL01375.1|AF406557_1|AF406557 PTPRE {Homo sapiens}, partial (9%)
4452	atcttgctgg	2				no_annot
4453	attgatatct	2				no_annot
4454	gaggcggtta	2				no_annot
4455	attctgttga	2				no_annot
4456	tcaatcacgt	2				no_annot
4457	ttataacggt	2				no_annot
4458	aattctcttt	2	C301434.1	64	0	
4459	tcatcgcgtc	2				no_annot
4460	acacctcccc	2	TC17169	46	0	weakly similar to GP|7297347|gb|AAF52607.1||AE003620 CG8552-PA {Drosophila melanogaster}, partial (3%)
4461	agcgtccata	2				no_annot
4462	tcgtggcaaa	2				no_annot
4463	cacgatccat	2				no_annot

4464	tttcacaaat	2	TC17108	51	3	weakly similar to PIR|T50839|T50839 U4/U6 small nuclear ribonucleoprotein hPrp3 [imported] - human, partial (27%)
4465	ttatgttatc	2				no_annot
4466	tccaattgac	2	TC17583	38	0	homologue to GP|10998535|gb|AAG25965.1|AF300968_7|AF300968 FlhA {Sinorhizobium meliloti}, partial (8%)
4466	tccaattgac	2	CD202621	34	0	
4467	gacttttatg	2				no_annot
4468	tgtgcgtcat	2				no_annot
4469	aacctctgga	2	TC11916	11	0	weakly similar to GP|15076873|gb|AAK82973.1|AF279307_1|AF279307 anti-silencing function 1B {Homo sapiens}, partial (72%)
4470	tcacataata	2	TC9641	93	0	
4471	gcctaacgca	2				no_annot
4472	tacaactagg	2				no_annot
4473	tatttaactt	2				no_annot
4474	gtcagatctt	2				no_annot
4475	caaaatacca	2	C306029.1	93	0	
4475	caaaatacca	2	C608190.1	82	1	
4475	caaaatacca	2	C715765.1	93	0	
4476	actgagacaa	2	TC17036	94	0	
4477	gtgaaagatc	2	TC8543	97	0	
4478	attctgttcg	2	AI395787	8	4	
4479	acgcttgaat	2				no_annot
4480	tgaatgttaa	2				no_annot
4481	agtgaagcgc	2				no_annot
4482	cgttatatgc	2				no_annot
4483	atttattttt	2	TC7049	50	4	
4484	aatccaaaat	2	TC9378	49	5	
4484	aatccaaaat	2	TC17624	65	0	
4485	ggatgtctcc	2				no_annot
4486	tataatgaga	2				no_annot

4487	ctatgtaagc	2				no_annot
4488	accaaccaaa	2				no_annot
4489	aatccaaaaa	2				no_annot
4490	ataacgaatt	2	CD081816	85	0	similar to GP|2425133|gb| DG2033 gene product {Dictyostelium discoideum}, partial (1%)
4491	atagttcact	2	TC7976	37	3	
4492	tcaaacagtg	2				no_annot
4493	tgtaagaatc	2	TC16374	6	1	
4494	cttaacagtt	2				no_annot
4495	atcagctgag	2	C602648.1	29	2	
4496	aaatagattt	2	TC7384	81	0	weakly similar to GP|14334082|gb|AAK60524.1|AF367970_1|AF367970 thymus LIM protein TLP-A {Mus musculus}, partial (30%)
4497	ttacaccaga	2				no_annot
4498	cggatccaat	2				no_annot
4499	tctttacaac	2	AF502282|Sma.856	57	0	Multiubiquitin binding protein S5a (S5a)
4500	gacgcgtcat	2	TC10886	54	0	
4500	gacgcgtcat	2	AW146529	46	1	
4501	cacaactacg	2	TC13851	62	1	
4502	ttaaaattat	2	TC17036	80	1	
4503	cgacggagtg	2				no_annot
4504	ggttacgtta	2				no_annot
4505	tactaagttt	2	CD195657	2	1	
4506	tgcactcaca	2				no_annot
4507	cggagggaca	2				no_annot
4508	tatgttgtga	2				no_annot
4509	tcgcgccgtc	2	C703490.1	48	0	
4510	aactttcacc	2				no_annot
4511	ggcggacgag	2				no_annot
4512	ttctatcata	2				no_annot
4513	taatgaattt	2	C607544.1	86	0	

4514	gacatcatct	2	C202635.1	79	0	
4514	gacatcatct	2	C601268.1	78	0	
4515	tagaaaaata	2				no_annot
4516	cgctctactg	2	TC10728	36	1	similar to SP|O76767|ERD2_DROME ER lumen protein retaining receptor. [Fruit fly] {Drosophila melanogaster}, complete
4516	cgctctactg	2	TC10729	8	1	similar to SP|O76767|ERD2_DROME ER lumen protein retaining receptor. [Fruit fly] {Drosophila melanogaster}, partial (51%)
4517	aagtagaaag	2	AA999379	46	0	
4518	atttatatat	2	TC11035	67	1	
4519	tactttattg	2				no_annot
4520	gattaatcct	2				no_annot
4521	gcatccactg	2	C201534.1	66	0	
4521	gcatccactg	2	C612343.1	65	0	
4522	taacttattt	2	C200881.1	53	5	
4523	aagtaacttt	2				no_annot
4524	tgaaaggaag	2				no_annot
4525	ctaattgcaa	2				no_annot
4526	caaaattcca	2				no_annot
4527	cacttcatcc	2	TC17429	16	7	GP|5566124|gb|AAD45325.1|AF158102_1|AF158102 retinoid-x-receptor {Schistosoma mansoni}, complete
4528	acggtagtgc	2				no_annot
4529	gtggtcgatc	2	TC7700	20	1	weakly similar to SP|Q16629|SFR7_HUMAN Splicing factor, arginine/serine-rich 7 (Splicing factor 9G8). [Human] {Homo sapiens}, partial (22%)
4530	attacaatta	2				no_annot
4531	attaacgata	2				no_annot
4532	ttcaccatca	2	C604328.1	78	1	
4533	accgaaagcc	2	TC6995	77	1	
4533	accgaaagcc	2	TC9448	74	0	
4533	accgaaagcc	2	TC13409	52	2	
4534	tggattcagt	2	C201002.1	61	1	

4535	tacgcaatgt	2	C605195.1	84	0	
4536	gagagaaaaa	2				no_annot
4537	ttcctttcgt	2				no_annot
4538	agacaccttt	2				no_annot
4539	ttaaccagat	2				no_annot
4540	atcccaatga	2	TC18748	68	1	
4540	atcccaatga	2	CD149172	75	0	
4541	acatcaggca	2				no_annot
4542	taagaatacg	2				no_annot
4543	ttgtttaaga	2				no_annot
4544	aacacacttt	2	TC11443	78	0	weakly similar to GP|505150|dbj|BAA06693.1||D31895 xpacxe2 protein {Xenopus laevis}, partial (31%)
4545	ctacgtgtat	2				no_annot
4546	tagatagcat	2				no_annot
4547	tttcgttttt	2				no_annot
4548	atacgcaaga	2	C603057.1	74	0	
4549	ggaaatctaa	2				no_annot
4550	tacagacata	2				no_annot
4551	tattttgtgt	2				no_annot
4552	tatgaatgtt	2				no_annot
4553	gtatatatat	2	TC12205	24	1	similar to PIR|S59634|S59634 endo-1,4-beta-xylanaseF precursor - Pseudomonas fluorescens, partial (4%)
4553	gtatatatat	2	CD061067	30	1	
4554	tttatatgcc	2				no_annot
4555	atgagcacaa	2				no_annot
4556	atcatcgcgc	2				no_annot
4557	gacaggattt	2	TC13827	35	3	similar to GP|4107017|dbj|BAA36294.1||AB001773 PEM-6 {Ciona savignyi}, partial (29%)
4558	ctatttattg	2				no_annot
4559	gccgacgagg	2	U19945|Sma.822	2	8	Actin
4560	tcaatatgca	2				no_annot
4561	ccattgactt	2				no_annot

4562	cattttgtgt	2	TC11716	35	2	
4563	aaatttgatt	2	BE519995	45	0	
4564	ttcctttcat	2	L31531|Sma.694	93	2	Phosphofructokinase (PFK)
4565	aagatgaagt	2				no_annot
4566	cccatattcg	2				no_annot
4567	ttcattgaat	2				no_annot
4568	taccatcaat	2				no_annot
4569	taattttgtt	2	TC18071	69	0	
4570	gtggtagtct	2	C209456.1	31	4	
4570	gtggtagtct	2	C612231.1	31	4	
4571	ttccactgac	2	TC11715	69	0	
4572	gctgatactt	2	TC15477	88	0	
4573	tgccttttca	2	TC11548	58	0	
4574	ttacatcaga	2				no_annot
4575	caatagtcgt	2				no_annot
4576	cagtgaggcg	2				no_annot
4577	gagcgacaat	2				no_annot
4578	gctttaacat	2	CD117801	52	0	
4579	atggtttctt	2				no_annot
4580	gaacatttat	2	TC15232	53	0	
4581	tctgatcaaa	2	TC16851	68	1	SP|P52798|EFA4_HUMAN Ephrin-A4 precursor (EPH-related receptor tyrosine kinase ligand 4) (LERK-4). [Human] {Homo sapiens}, partial (5%)
4582	aagtcattat	2				no_annot
4583	atgtcgatgg	2	M83294|Sma.264	66	1	Triose phosphate isomerase
4584	tatttatcgc	2				no_annot
4585	ggctgtccgg	2	TC13839	37	7	weakly similar to PIR|S05583|XNCHDC aspartate transaminase, cytosolic - chicken, partial (59%)
4586	tttgaatttc	2	TC18693	57	1	
4587	ggacttaatc	2				no_annot
4588	tttggttgag	2				no_annot
4589	tgtaatgata	2	TC17576	58	0	

4590	atgtacagtg	2	C201230.1	65	3	
4590	atgtacagtg	2	C602856.1	7	3	
4590	atgtacagtg	2	C604669.1	97	0	
4591	gtgtgtttac	2				no_annot
4592	atcagcattt	2				no_annot
4593	gttgtttggt	2	TC7358	74	3	similar to SP|O00505|IMA3_HUMAN Importin alpha-3 subunit (Karyopherin alpha-3 subunit) (SRP1-gamma). [Human] {Homo sapiens}, partial (85%)
4594	aaatatgttt	2				no_annot
4595	gaagtgtgtg	2	TC10148	0	3	
4596	acgatttaaa	2	C202088.1	22	2	
4596	acgatttaaa	2	C605086.1	22	2	
4597	ttgttttagt	2	CD190090	22	0	
4598	aatttcaagt	2	TC16991	5	0	weakly similar to SP|O08765|GEF2_HUMAN Ganglioside expression factor 2 (GEF-2) (General protein transport factor p16) (GATE-16) (GABA(A) receptor-associated protein-like 2) (MAP1 light chain 3 related protein). [Bovine] {Bos taurus}, partial (96%)
4599	tgcataggat	2				no_annot
4600	acgttagtca	2				no_annot
4601	cacttacatc	2				no_annot
4602	aaaagtgaga	2	CD202859	94	0	similar to GP|19067879|gb|AAK14815.1||AY027869 polyprotein {Schistosoma japonicum}, partial (10%)
4603	tggtctgttt	2	CD156091	75	0	
4604	gtgtcttgct	2	TC15956	88	0	
4605	ccccctatgg	2				no_annot
4606	ctcaacctgt	2				no_annot
4607	acccacaagt	2				no_annot
4608	gaacaacaac	2	TC16784	5	10	
4608	gaacaacaac	2	CD136633	36	1	
4609	tagaaaaaaa	2	L78441|Sma.14	95	0	14-3-3-2 mRNA, 3 end of cds
4610	ccagtaaact	2				no_annot
4611	ctactcacta	2	CD135771	65	0	

4612	actggttatg	2				no_annot
4613	ttgcttgtcc	2				no_annot
4614	taaaacaata	2	L26968|Sma.22	98	0	Potassium channel protein (SKv1.1)
4615	actatgacac	2	BF936577	32	0	weakly similar to GP|28854921|gb|AAO57983.1||AE016872 peptide ABC transporter, ATP-binding protein {Pseudomonas syringae pv. tomato str. DC3000}, partial (10%)
4616	tgaacgcgtc	2				no_annot
4617	gacattatac	2	TC16936	20	3	weakly similar to PIR|E86466|E86466 protein F23M19.10 [imported] - Arabidopsis thaliana, partial (4%)
4617	gacattatac	2	TC17014	1	5	weakly similar to GP|5825503|gb|AAD53274.1||AF169146 fragile X-related protein {Danio rerio}, partial (9%)
4617	gacattatac	2	TC17014	1	2	weakly similar to GP|5825503|gb|AAD53274.1||AF169146 fragile X-related protein {Danio rerio}, partial (9%)
4617	gacattatac	2	CD140601	4	0	
4618	caaatgacgc	2				no_annot
4619	ccctattaag	2				no_annot
4620	cagcagccgc	2				no_annot
4621	gtggtcgaat	2	TC8014	71	0	
4621	gtggtcgaat	2	TC10937	77	0	similar to SP|O76484|KC2A_SPOFR Casein kinase II, alpha chain (CK II). [Fall armyworm] {Spodoptera frugiperda}, partial (91%)
4622	gaaaaataaa	2				no_annot
4623	cattcgaaca	2	C203332.1	34	0	
4624	aagtaactaa	2				no_annot
4625	taagacccat	2				no_annot
4626	gtgatttacg	2	TC8145	76	0	similar to SP|P35249|AC12_HUMAN Activator 1 37 kDa subunit (Replication factor C 37 kDa subunit) (A1 37 kDa subunit) (RF-C 37 kDa subunit) (RFC37). [Human] {Homo sapiens}, partial (42%)
4627	ttttacaata	2	CD189639	21	0	
4628	cttgtagggg	2				no_annot
4629	ggttcggcga	2				no_annot

4630	cattcgaaaa	2				no_annot
4631	agtttttcaa	2	BG932580	25	3	
4632	tcatacaata	2				no_annot
4633	taatggagga	2	TC8683	52	0	
4634	cagttcaatt	2				no_annot
4635	cttgaaatca	2	CD073583	7	3	
4636	atcttcattc	2				no_annot
4637	tccaatatgt	2	C701484.1	93	0	
4638	gatgtgctta	2				no_annot
4639	gtgatttaaa	2	TC19231	4	4	similar to GP|458945|gb|AAB68370.1||U00030 Yhr204wp {Saccharomyces cerevisiae}, partial (8%)
4640	gcataataat	2				no_annot
4641	taatgtaata	2	TC8113	7	2	
4642	gtaaagaacc	2				no_annot
4643	gttccaatct	2	TC11366	87	0	weakly similar to PIR|F86260|F86260 protein T12C24.26 [imported] - Arabidopsis thaliana, partial (5%)
4644	tagactacta	2				no_annot
4645	agtgattggc	2	TC8140	42	0	similar to GP|7008402|gb|AAF34998.1||AF229839 kappa B-ras 1 {Homo sapiens}, partial (9%)
4646	ttgaggattt	2	CD183431	44	1	
4647	tgaattctac	2	AI975047	89	2	
4648	ccaaccctcc	2				no_annot
4649	ttgaggattc	2				no_annot
4650	taaacggtgt	2	C200949.1	72	0	
4651	acttgctaga	2	TC15032	65	1	
4652	tatctgtcga	2				no_annot
4653	ttttaatttt	2	C210056.1	77	1	
4654	cgttgccacc	2				no_annot
4655	aagatgtacg	2	TC8132	83	0	weakly similar to GP|7294263|gb|AAF49614.1||AE003530 CG7764-PA {Drosophila melanogaster}, partial (32%)
4656	aatgaagaag	2				no_annot

4657	cttgtgtcat	2				no_annot
4658	tacatagtgt	2	CD072170	32	0	
4658	tacatagtgt	2	CD075112	13	4	
4659	ccgttctgat	2				no_annot
4660	acacaatgca	2				no_annot
4661	ataagctgta	2				no_annot
4662	ggttaaacag	2				no_annot
4663	actcagcgtc	2	TC8889	42	0	homologue to GP|15042221|gb|AAK82001.1|AF303741_79|AF303741 079L {Chilo iridescent virus}, partial (18%)
4664	cagtgtcctt	2				no_annot
4665	ccattgacaa	2				no_annot
4666	gtgaaacaca	2	L01634|Sma.848	79	6	Myosin heavy chain (MYH)
4667	aagtgatcgg	2				no_annot
4668	taacagtaaa	2				no_annot
4669	tggcttggtt	2	TC13833	26	1	
4670	tgagaaaacc	2				no_annot
4671	tacatagtcg	2				no_annot
4672	gttctacagg	2	C317950.1	12	2	
4673	gatattctgc	2				no_annot
4674	aaacccactg	2				no_annot
4675	gttaactatt	2				no_annot
4676	aaaaggcgat	2				no_annot
4677	ttttactatc	2				no_annot
4678	agcggtacca	2	TC8108	93	0	
4679	gcacccaaag	2	C605473.1	58	4	
4680	ttttacaagt	2	TC13648	84	3	homologue to GP|832859|gb|AAA67384.1||L32580 presumptive protein 3 {Sceloporus grammicus}, partial (19%)
4681	ttatctaatg	2	TC13191	12	2	
4682	agtttaatta	2				no_annot
4683	gtgctggtct	2				no_annot

4684	ctaaacgttt	2	C211246.1	96	0	
4685	tatcacaaaa	2				no_annot
4686	cttgcagcgt	2	TC7889	30	2	weakly similar to GP|20072621|gb|AAH27205.1||BC027205 dipeptidyl peptidase 7 {Mus musculus}, partial (33%)
4687	tcttaggtga	2	CD179460	35	0	weakly similar to GP|19918226|gb|AAM07471.1||AE011124 proteasome-activating nucleotidase {Methanosarcina acetivorans str. C2A} [Methanosarcina acetivorans C2A], partial (17%)
4688	ccgtaataaa	2				no_annot
4689	tgatgaattg	2	C209233.1	76	0	
4689	tgatgaattg	2	C602506.1	70	0	
4690	ctgattatcc	2				no_annot
4691	gcaaaaatat	2	AW061428	52	0	
4692	ttgggtcata	2	TC13919	74	0	
4693	gaagcgaccc	2	TC11918	82	0	similar to GP|7296845|gb|AAF52120.1||AE003606 CG1078-PA {Drosophila melanogaster}, partial (5%)
4694	ctacttacta	2				no_annot
4695	cttttaggcg	2				no_annot
4696	tgattcagat	2	TC12034	46	0	weakly similar to SP|Q9Y5T5|UBPG_HUMAN Ubiquitin carboxyl-terminal hydrolase 16(Ubiquitin thiolesterase 16) (Ubiquitin-specific processing protease 16) (Deubiquitinating enzyme 16) (Ubiquitin processing protease UBP-M). [Human] {Homo sapiens}, partial (5%)
4697	ttgaatgata	2				no_annot
4698	ttacagcttt	2				no_annot
4699	tgtagtgtga	2				no_annot
4700	aagaatgtgt	2				no_annot
4701	tcattgtcaa	2				no_annot
4702	aggcaggtgg	2				no_annot
4703	agtctccgaa	2	TC17490	35	1	
4704	actaactagc	2				no_annot
4705	gagtagtgaa	2	CD081916	70	0	

4706	tcacagtttc	2	AI394782	64	0	
4706	tcacagtttc	2	CD117970	88	0	
4707	ccactagtcc	2				no_annot
4708	ccatccagta	2				no_annot
4709	aaaacgaata	2				no_annot
4710	tctgtctcca	2	TC6894	42	1	
4710	tctgtctcca	2	TC7017	96	0	
4710	tctgtctcca	2	TC7267	49	2	
4710	tctgtctcca	2	TC13155	84	0	
4710	tctgtctcca	2	CD067002	81	1	
4710	tctgtctcca	2	CD161137	51	1	
4710	tctgtctcca	2	CD166865	4	3	
4710	tctgtctcca	2	CD184819	76	0	
4710	tctgtctcca	2	CD139674	21	0	
4711	aacgcaagga	2	C201331.1	9	4	
4711	aacgcaagga	2	C611827.1	10	5	
4712	aagcgaaaac	2	C315385.1	14	0	
4712	aagcgaaaac	2	C718283.1	14	0	
4713	ttctccgtct	2	C210587.1	22	1	
4714	gggaactgag	2				no_annot
4715	cgactcataa	2				no_annot
4716	acttcttcaa	2	C200978.1	72	0	
4716	acttcttcaa	2	C608620.1	71	0	
4717	actgacgatc	2				no_annot
4718	agatagtttt	2	C208868.1	86	0	
4718	agatagtttt	2	C603784.1	84	0	
4719	gttcaactcc	2	TC19228	46	0	
4720	gctatatgtt	2				no_annot
4721	gatactgaat	2				no_annot
4722	ttgaaccatt	2	C300418.1	9	2	

4722	ttgaaccatt	2	C715656.1	9	2	
4723	aacgaataat	2				no_annot
4724	accttgactt	2				no_annot
4725	ttactaagat	2	C201391.1	40	1	
4725	ttactaagat	2	C601096.1	89	0	
4726	gggttcgcga	2				no_annot
4727	attcgttgtt	2				no_annot
4728	gctatcacac	2	CD132796	49	1	
4729	ataggcggtt	2				no_annot
4730	cacccccttt	2				no_annot
4731	gatttctgtg	2	TC14764	60	0	weakly similar to GP|19744157|dbj|BAB86840.1||AB056444 Toll-like receptor 4 {Bos taurus}, partial (3%)
4731	gatttctgtg	2	TC19661	39	0	
4732	tatcacggag	2				no_annot
4733	gcctcctaat	2				no_annot
4734	taaacctgtt	2				no_annot
4735	gtggtccaac	2	TC7873	25	1	
4735	gtggtccaac	2	TC7874	48	1	
4736	tccaggtaag	2				no_annot
4737	gaattgtgct	2				no_annot
4738	acagaggaat	2	TC17156	68	0	weakly similar to GP|10314023|gb|AAG15309.2|AF151697_1|AF151697 sentrin-specific protease {Homo sapiens}, partial (10%)
4739	ctgttgtttt	2				no_annot
4740	tcacagatca	2				no_annot
4741	ttttaattag	2				no_annot
4742	ggggtataca	2	TC16992	86	1	weakly similar to GP|15291453|gb|AAK92995.1||AY051571 GH21853p {Drosophila melanogaster}, partial (10%)
4743	gtgatgatga	2	TC9327	61	1	
4744	ttgaagtaag	2				no_annot
4745	gtagaggagt	2				no_annot

4746	tagctgataa	2				no_annot
4747	aagacacatt	2				no_annot
4748	gagaacatac	2	CD084252	76	0	
4749	tgtgcacttg	2				no_annot
4750	gctgtgactc	2				no_annot
4751	ttgagtgttt	2				no_annot
4752	attagtgtca	2				no_annot
4753	aacagtagat	2				no_annot
4754	atgcttaaat	2	TC9980	12	3	
4754	atgcttaaat	2	TC15071	96	0	weakly similar to GP|5305403|gb|AAD41634.1|AF072372_1|AF072372 lysosomal trafficking regulator 2 {Mus musculus}, partial (4%)
4755	tagaaacgcc	2				no_annot
4756	ccaatattta	2	C201061.1	1	0	
4757	ctaaacgtgt	2	TC9366	57	1	similar to GP|21429162|gb|AAM50300.1||AY119646 RE44923p {Drosophila melanogaster}, partial (9%)
4757	ctaaacgtgt	2	TC13602	77	2	homologue to GP|27372732|gb|AAO06264.1||AY158954 histone protein Hist2h3c1 {Mus musculus}, partial (80%)
4758	aacagaccat	2				no_annot
4759	agaatgatta	2				no_annot
4760	actgtttttt	2				no_annot
4761	cagtaggcgt	2				no_annot
4762	gttttctttt	2	C211248.1	96	0	
4762	gttttctttt	2	C610242.1	92	0	
4763	ttttccagga	2				no_annot
4764	tccaccccct	2	AW061393	40	1	
4765	catcaccgtt	2				no_annot
4766	ctaatcgaaa	2				no_annot
4767	atcgttatac	2				no_annot
4768	aagagcacgc	2				no_annot
4769	gtgatgatca	2	TC19441	41	1	

4770	taaattccta	2				no_annot
4771	tatagaaatc	2	TC19218	53	0	
4772	caaaagtgga	2				no_annot
4773	aatgtgatta	2				no_annot
4774	tttatctata	2	CD126199	30	1	
4775	tatattacgt	2				no_annot
4776	tagttttatt	2	TC10032	84	0	
4777	taatatccat	2				no_annot
4778	cactctgggg	2	CD080712	82	0	
4779	gaaggcgaac	2				no_annot
4780	cattgctggc	2	Y08487|Sma.698	28	3	Elongation factor 1-alpha
4781	cgaaaaattc	2				no_annot
4782	acttgccgat	2	TC17439	88	0	
4783	aatccagtca	2	TC10782	92	0	
4784	tgaactcaat	2	C200015.1	26	1	
4784	tgaactcaat	2	C201390.1	34	4	
4785	ttttccagct	2				no_annot
4786	ctaatccatt	2				no_annot
4787	taatttattt	2	TC13817	86	0	similar to GP|25012819|gb|AAN71500.1||BT001745 RE74312p {Drosophila melanogaster}, partial (91%)
4787	taatttattt	2	CD144143	85	0	similar to GP|11527140|gb|AAG36902.1|AF257236_1|AF257236 poly(A) binding protein II {Xenopus laevis}, partial (20%)
4788	acaagatcct	2				no_annot
4789	aaacaataca	2	TC13459	89	2	weakly similar to GP|5410296|gb|AAD43019.1||AF100755 homeobox prox 1 {Homo sapiens}, partial (32%)
4790	tcaatcaaga	2				no_annot
4791	atgtggtcac	2	TC13163	60	1	
4792	tcgaagagca	2				no_annot
4793	gaaatcatat	2				no_annot
4794	tgttttcttt	2	CD076353	4	2	

4795	ttagctcgta	2				no_annot
4796	agagatgtga	2				no_annot
4797	cgactacata	2	CD067232	70	0	
4798	caagtttttt	2	TC7032	0	5	
4799	tatatgcaaa	2	TC8706	52	1	
4800	aataaagtgt	2				no_annot
4801	catatgcaca	2	TC14161	54	0	
4802	tacatactca	2	C202089.1	3	0	
4802	tacatactca	2	C302209.1	58	0	
4802	tacatactca	2	C600718.1	89	0	
4802	tacatactca	2	C602031.1	6	0	
4803	atagcttctg	2				no_annot
4804	gactttgtcc	2				no_annot
4805	aaacaataaa	2	TC12528	1	4	
4806	ccaataataa	2				no_annot
4807	tgttcgaaat	2	TC19727	31	1	similar to PIR|A35477|URHUAP peptidylglycine monooxygenase/ peptidylamidoglycolate lyaseprecursor - human, partial (4%)
4808	gagtgtttga	2	L06180|Sma.282	86	0	MAK16-like protein
4809	ttggtaacaa	2	TC17832	22	3	weakly similar to SP|P29720|TMPB_TREPH Treponemal membrane protein B precursor (Antigen tmpB). {Treponema phagedenis}, partial (6%)
4809	ttggtaacaa	2	CD074665	30	0	
4810	tgctaacttg	2	C601182.1	12	0	
4811	atgaaaaact	2	CD139368	20	1	homologue to PIR|A48433|A48433 tubulin alpha chain - fluke (Schistosoma mansoni), partial (22%)
4812	cagcacactg	2				no_annot
4813	cttatgtgta	2	CD201253	1	6	
4813	cttatgtgta	2	CD201253	1	2	
4813	cttatgtgta	2	N21839	70	0	
4814	atctaagtat	2	TC13518	14	1	homologue to GP|12248342|gb|AAG13168.2||AF216698 NADH dehydrogenase subunit 1 {Schistosoma mansoni}, partial (96%)

4815	ctttcaccta	2	TC13889	15	1	similar to SP|P38607|VAA2_HUMAN Vacuolar ATP synthase catalytic subunit A, osteoclast isoform(V-ATPase A subunit 2) (Vacuolar proton pump alpha subunit 2) (V-ATPase 69 kDa subunit 2) (Isoform HO68). [Human] {Homo sapiens}, partial (61%)
4816	caattggcga	2	CD076793	15	0	
4817	tcacaaacgc	2				no_annot
4818	acaacttaag	2	TC12951	64	0	
4819	ctacctgtgt	2				no_annot
4820	tacaaaacat	2	TC16668	24	1	
4820	tacaaaacat	2	TC16671	32	2	
4821	aagtaagctg	2				no_annot
4822	tataagctca	2				no_annot
4823	ttttataaat	2	N21877	42	0	
4824	caaaataaac	2				no_annot
4825	ttagatcaat	2	AY323529|Sma.76	60	1	ATP-diphosphohydrolase 1 (ATPDase1)
4826	ttccaggcca	2	C200237.1	92	0	
4826	ttccaggcca	2	C603239.1	94	0	
4827	ttaattgagg	2	C601720.1	12	3	
4828	caaagttttc	2				no_annot
4829	taacctcgtg	2				no_annot
4830	cacaaaacac	2				no_annot
4831	aatggtgaag	2	CD118994	22	1	
4832	atacagaaag	2	TC13483	68	1	
4832	atacagaaag	2	TC15882	31	2	
4832	atacagaaag	2	CD077268	89	0	
4832	atacagaaag	2	BG932580	61	2	
4833	tacaatgtct	2	TC14533	75	0	similar to GP|17945143|gb|AAL48631.1||AY071009 RE09163p {Drosophila melanogaster}, partial (5%)
4833	tacaatgtct	2	CD186818	5	3	
4833	tacaatgtct	2	CD186818	5	1	
4834	taaagacatc	2	TC18587	72	2	similar to PIR|F88924|F88924 protein R02C2.2 [imported] - Caenorhabditis elegans, partial (4%)

4835	agcactgaca	2				no_annot
4836	tttgtgaata	2				no_annot
4837	gctcattagt	2				no_annot
4838	tattaaatta	2	TC14115	1	2	similar to GP|18677192|gb|AAL78228.1|AF345800_1|AF345800 SCN esophageal secretory protein {Heterodera glycines}, partial (9%)
4838	tattaaatta	2	TC14908	43	0	
4838	tattaaatta	2	CD132796	86	0	
4839	tcgcgtgtct	2				no_annot
4840	gcggtttaag	2				no_annot
4841	aagagaagaa	2				no_annot
4842	atcacttggt	2				no_annot
4843	ttcttttcat	2				no_annot
4844	ctggaggaac	2				no_annot
4845	cacccatcaa	2	C203423.1	94	0	
4845	cacccatcaa	2	C609616.1	94	0	
4845	cacccatcaa	2	C609826.1	25	2	
4846	actatagttt	2				no_annot
4847	taatagtatt	2	TC12026	37	0	
4848	tgggtattgt	2	CD061201	83	1	
4848	tgggtattgt	2	CD073412	12	4	
4849	tttaacatac	2				no_annot
4850	ttaaatgtgt	2				no_annot
4851	aaatgtacaa	2	C609414.1	20	1	
4852	tatactgtat	2				no_annot
4853	ggtcgtggta	2	TC10776	16	6	weakly similar to GP|27374373|gb|AAO01111.1||AY190960 CG4686-PA {Drosophila pseudoobscura}, partial (29%)
4853	ggtcgtggta	2	TC10777	5	4	weakly similar to GP|27374373|gb|AAO01111.1||AY190960 CG4686-PA {Drosophila pseudoobscura}, partial (23%)

4853	ggtcgtggta	2	CD084181	28	3	weakly similar to GP|27374373|gb|AAO01111.1||AY190960 CG4686-PA {Drosophila pseudoobscura}, partial (34%)
4854	gcaaattagg	2				no_annot
4855	tgacttttca	2	TC16269	89	0	
4855	tgacttttca	2	TC18643	22	0	
4855	tgacttttca	2	CD127936	19	0	
4855	tgacttttca	2	CD069266	67	0	
4856	gctatttcgg	2	TC8081	14	0	weakly similar to GP|25058652|gb|AAH39272.1||BC039272 WD repeat domain 4 {Mus musculus}, partial (6%)
4857	tacaacctct	2				no_annot
4858	gattcaagga	2	CD082742	64	0	similar to GP|3170241|gb|AAC18057.1||AF042732 TU37B2 {Anopheles gambiae}, partial (80%)
4859	gtatcagtat	2				no_annot
4860	attatggaga	2	TC15694	72	0	
4861	gaacctgata	2				no_annot
4862	ctcaccactt	2				no_annot
4863	gtgataacat	2				no_annot
4864	atggactttg	2				no_annot
4865	tggttctatg	2				no_annot
4866	ccgtttattg	2				no_annot
4867	cacagggaat	2	L01634|Sma.848	29	13	Myosin heavy chain (MYH)
4868	tcaatgtgga	2				no_annot
4869	aacgtctttg	2				no_annot
4870	ccggtgcctc	2				no_annot
4871	acacactata	2				no_annot
4872	ttaatccaag	2				no_annot
4873	gtttcatttt	2				no_annot
4874	ctcagaattt	2				no_annot
4875	tcatttccca	2				no_annot
4876	ggtgccactc	2	C605547.1	80	1	

4877	cttctgcgct	2				no_annot
4878	tttatgtgca	2	TC10714	74	0	weakly similar to GP|11878247|gb|AAG40865.1|AF311225_1|AF311225 geranylgeranyltransferase beta subunit {Arabidopsis thaliana}, partial (8%)
4879	aggtctggag	2				no_annot
4880	aacaacaaag	2				no_annot
4881	ttgtttgtat	2				no_annot
4882	taatttatac	2				no_annot
4883	aaccaactca	2	C206603.1	88	0	
4883	aaccaactca	2	C600102.1	87	0	
4884	aaaacactgc	2				no_annot
4885	tacagatgta	2				no_annot
4886	gactattctg	2				no_annot
4887	atgcccccat	2				no_annot
4888	tacactttgt	2	CD201406	68	2	similar to GP|13177306|gb|AAK14450.1|AF204951_24|AF204951 EsV-1-24 {Ectocarpus siliculosus virus}, partial (4%)
4889	cttgtgtatt	2				no_annot
4890	gtcgaattcg	2	TC7409	98	0	similar to SP|Q00610|CLH1_HUMAN Clathrin heavy chain 1 (CLH-17). [Human] {Homo sapiens}, partial (95%)
4891	catttgacag	2				no_annot
4892	tggttgaggt	2				no_annot
4893	ttcgtatttg	2				no_annot
4894	gattatgccg	2	TC18311	45	0	
4895	gaaaatgatg	2	TC9298	80	0	
4896	ttcaagacaa	2				no_annot
4897	cctaccactt	2				no_annot
4898	atgttaatag	2	C201135.1	18	0	
4898	atgttaatag	2	C607873.1	12	1	

4899	tcaattaacg	2	TC16681	63	0	weakly similar to GP|20977567|gb|AAM28211.1||AY099523 vacuolar ATP synthase 16 kDa proteolipid subunit {Danio rerio}, partial (84%)
4900	gattcctcac	2	TC7695	94	0	
4901	cgaagagatt	2				no_annot
4902	gaaaccaaga	2	TC8055	39	0	similar to SP|Q15050|RRS1_HUMAN Ribosome biogenesis regulatory protein homolog. [Human] {Homo sapiens}, partial (25%)
4903	ttaaaatcaa	2				no_annot
4904	tcgcatcaca	2	TC14413	78	0	
4905	gcagccgggt	2				no_annot
4906	gcagttttaa	2				no_annot
4907	tagcgggtaa	2				no_annot
4908	gagttacttc	2				no_annot
4909	ggaaaacgaa	2				no_annot
4910	atatgctaaa	2	TC18195	77	0	similar to GP|17861418|gb|AAL39186.1||AY069041 GH03649p {Drosophila melanogaster}, partial (7%)
4911	ttatccgttg	2				no_annot
4912	ttgtttcttg	2	C611310.1	81	1	
4913	gaggctattg	2	TC7349	59	1	
4914	ggattttatc	2				no_annot
4915	tctaaatata	2	TC15507	1	1	
4916	caccctctct	2	C608351.1	99	0	
4917	gagaggtaat	2				no_annot
4918	tgaaagagag	2				no_annot
4919	ctttgacagc	2				no_annot
4920	ggtttctgta	2	TC16846	86	1	GP|6980092|gb|AAF34722.1|AF232025_1|AF232025 TGF-beta signal transducer Smad2 {Schistosoma mansoni}, complete
4921	aattggcgct	2				no_annot
4922	caccattgaa	2				no_annot
4923	gaacctgagt	2	TC11658	31	0	

4924	cactctcgat	2	TC10641	80	1	homologue to GP|2687973|gb|AAC66478.1||AE001121 B. burgdorferi predicted coding region BB0079 {Borrelia burgdorferi B31}, partial (5%)
4925	ttaagcacat	2	AA999404	13	0	
4926	agcgaacgta	2	C201017.1	21	1	
4926	agcgaacgta	2	C600144.1	20	1	
4927	ttcccataac	2	BE473328	19	2	
4928	tcaacaaaaa	2	TC8200	50	0	
4929	tacctattta	2	TC17323	87	0	
4930	taagcctaaa	2				no_annot
4931	ctaagtactg	2	CD087095	59	1	
4932	gtcgactaac	2				no_annot
4933	gtgttggtga	2	C208281.1	60	0	
4933	gtgttggtga	2	C312346.1	91	0	
4933	gtgttggtga	2	C707782.1	91	0	
4934	tctttaatta	2	TC16751	62	2	weakly similar to GP|4972623|gb|AAD34720.1|AF118103_1|AF118103 glutamine synthetase {Opsanus beta}, partial (83%)
4934	tctttaatta	2	TC17037	77	0	similar to GP|28856144|gb|AAH48081.1||BC048081 casein kinase 1, alpha 1 {Mus musculus}, partial (73%)
4935	gtttctaatg	2				no_annot
4936	gtaacgtttg	2	C202323.1	92	0	
4937	aacaccagca	2	C606311.1	12	0	
4938	cgaacagccc	2				no_annot
4939	tagaggatga	2	CD137389	38	1	similar to GP|19421994|gb|AAL87849.1||AF414456 calcium-binding protein {Echinococcus granulosus}, partial (16%)
4940	aatgttgtgc	2				no_annot
4941	ccctctttca	2				no_annot
4942	tttcttcaaa	2	TC17054	38	3	weakly similar to GP|6002571|gb|AAF00041.1|AF091370_1|AF091370 hnRNP I-related RNA transport protein VgRBP60 {Xenopus laevis}, partial (34%)
4943	tttaggaaaa	2	CD186636	16	1	

4944	aaccgatcga	2	C202270.1	11	1	
4945	atgatcattg	2	TC12365	87	0	similar to GP|22651742|gb|AAM26940.1||AY096120 maturase K {Aldrovanda vesiculosa}, partial (5%)
4945	atgatcattg	2	BG932334	4	0	SP|P34072|MKS1 Negative regulator of RAS-cAMP pathway. [Baker's yeast] {Saccharomyces cerevisiae}, partial (2%)
4946	attgtaaatg	2				no_annot
4947	tgcaaatcta	2	C608019.1	9	6	
4948	agacctactc	2				no_annot
4949	acaataagca	2				no_annot
4950	gtcatatttc	2	TC12322	75	0	similar to GP|29898827|gb|AAP12098.1||AE017015 N-acetylmuramoyl-L-alanine amidase {Bacillus cereus ATCC 14579}, partial (3%)
4951	gcgagtcgag	2				no_annot
4952	atttggctat	2	TC7488	77	1	
4953	acaataagaa	2				no_annot
4954	cacctctatt	2				no_annot
4955	ttcattagat	2				no_annot
4956	catttataaa	2	C301198.1	18	0	
4956	catttataaa	2	C700213.1	18	0	
4957	aaccgtcttt	2	TC14732	72	0	
4958	acttctaagt	2				no_annot
4959	gattgtaact	2				no_annot
4960	taaatgaaag	2				no_annot
4961	cacgatagtg	2				no_annot
4962	gataaattca	2				no_annot
4963	tataacattt	2				no_annot
4964	tatgagtgac	2	TC17241	71	2	weakly similar to GP|10048296|gb|AAG12342.1||AF294845 glycerol-3-phosphate dehydrogenase {Mus musculus}, partial (54%)
4965	atctgtgtac	2	CD145448	40	0	
4966	catcaaacta	2	TC18524	37	1	

4967	gtgaaatgga	2	CD066322	63	0	weakly similar to SP|P47943|IF4A_SCHPO Eukaryotic initiation factor 4A (eIF-4A) (eIF4A). [Fission yeast] {Schizosaccharomyces pombe}, partial (16%)
4968	ttccctcctt	2				no_annot
4969	tttttgaaaa	2	TC10570	72	1	
4969	tttttgaaaa	2	TC13764	47	6	
4970	catatattta	2				no_annot
4971	tgatacgatc	2				no_annot
4972	gatacctgta	2				no_annot
4973	gttatcgacg	2				no_annot
4974	ttgtttctcc	2				no_annot
4975	gctatatttt	2	TC19418	68	0	weakly similar to PIR|AC0184|AC0184 malate dehydrogenase (oxaloacetate-decarboxylating)[imported] - Yersinia pestis (strain CO92), partial (4%)
4976	gagagccaat	2				no_annot
4977	tccttggtgt	2				no_annot
4978	tagaaactgt	2				no_annot
4979	cactcccttc	2				no_annot
4980	agcttttgga	2				no_annot
4981	acggtctggt	2	CD185748	80	0	weakly similar to GP|13488609|gb|AAK26168.1||AY028916 GAJ {Homo sapiens}, partial (36%)
4982	tctataccct	2	TC10613	87	1	weakly similar to GP|12006785|gb|AAG44917.1|AF290330_1|AF290330 phosphoglucomutase {Drosophila melanogaster}, partial (52%)
4983	tgttgtagct	2	TC12002	45	0	homologue to GP|4160443|gb|AAD05243.1||AF098297 CBF1 interacting corepressor CIR {Homo sapiens}, partial (4%)
4984	acaacgttat	2	TC14282	8	3	
4985	ttgcaattca	2	TC17747	72	0	weakly similar to PIR|B88483|B88483 protein mel-32 [imported] - Caenorhabditis elegans, partial (28%)
4986	aatgcgaaaa	2	TC11382	55	1	
4987	agcataaaaa	2				no_annot
4988	cgccctcttt	2				no_annot

4989	gaatgtcagt	2	C209400.1	53	1	
4990	ttattttcta	2	TC12484	88	0	
4991	ttatactgtg	2	TC11289	33	0	
4992	ggcgttctgt	2				no_annot
4993	ctcttgtgtt	2				no_annot
4994	atgtatatgt	2				no_annot
4995	cactagctga	2				no_annot
4996	tagaggttct	2	TC9501	77	0	
4997	aaagcaagag	2	CD098365	78	0	
4998	ttttcctttc	2				no_annot
4999	aacacaccgc	2				no_annot
5000	ggaaagtatg	2	C307791.1	63	0	
5000	ggaaagtatg	2	C606675.1	52	0	
5001	atcacagacg	2	C207228.1	48	1	
5002	aaaaaaatta	2	CD083448	60	1	
5003	tgattttttg	2				no_annot
5004	aagtgatact	2				no_annot
5005	taaatgtaaa	2	TC10751	89	1	weakly similar to GP|4490377|emb|CAB38634.1||AJ133649 actin related complex p41 subunit {Drosophila melanogaster}, partial (34%)
5005	taaatgtaaa	2	TC15006	10	1	
5006	ctttacgtcg	2	TC10393	42	0	similar to GP|9971154|dbj|BAB12429.1||AB018543 mkpA {Dictyostelium discoideum}, partial (3%)
5007	ccatagataa	2				no_annot
5008	cattataaga	2	TC10948	76	1	weakly similar to GP|7295709|gb|AAF51014.1||AE003576 CG15432-PA {Drosophila melanogaster}, partial (28%)
5009	ttgtgcagat	2				no_annot
5010	atcacagaag	2	C200976.1	20	0	
5011	cgattagaaa	2				no_annot

5012	ctactattga	2	TC13144	45	0	similar to SP|Q16531|DDB1_HUMAN DNA damage binding protein 1 (Damage-specific DNA binding protein 1) (DDB p127 subunit) (DDBa) (UV-damaged DNA-binding protein 1) (UV-DDB 1) (Xeroderma pigmentosum group E complementing protein) (XPCe) (X- associated protein 1) (XAP-1). [Human] {Homo sapiens}, partial (7%)
5013	atgtatatct	2	TC13582	85	0	similar to SP|Q8PS69|HCP_METMA Hydroxylamine reductase(Hybrid-cluster protein) (HCP). [Methanosarcina frisia] {Methanosarcina mazei}, partial (4%)
5014	tgttgttgga	2				no_annot
5015	tgcaaatcca	2				no_annot
5016	tatgttccct	2				no_annot
5017	tgaatagttt	2	TC14449	87	0	similar to GP|7299916|gb|AAF55090.1||AE003706 CG7552-PA {Drosophila melanogaster}, partial (16%)
5018	atgatcatat	2				no_annot
5019	gaacgtagca	2				no_annot
5020	gactcggttg	2				no_annot
5021	gtgactcata	2				no_annot
5022	atgatcataa	2	CD084124	19	0	
5023	acaaactgga	2				no_annot
5024	aagaaaaaag	2	CD162609	10	4	weakly similar to PIR|E87691|E87691 fumarylacetoacetate hydrolase family protein [imported] - Caulobacter crescentus, partial (32%)
5025	aagaaaaaaa	2	TC10062	17	1	
5026	atcgctcgca	2				no_annot
5027	tggtcgtcag	2				no_annot
5028	tgataagtaa	2	TC12597	25	0	
5029	gtttgatcga	2	TC11090	69	1	similar to SP|Q9CQC1|CRN1_MOUSE Crooked neck-like protein 1 (Crooked neck homolog). [Rat] {Rattus norvegicus}, partial (24%)
5030	ttttctattt	2				no_annot
5031	ggcgaatgaa	2				no_annot
5032	gtaaacagag	2				no_annot
5033	tggccgaaca	2	TC18141	57	0	

5034	gtcccttttt	2				no_annot
5035	taggtatcga	2				no_annot
5036	ttctttggta	2				no_annot
5037	cgaagtttgt	2				no_annot
5038	tcatccatag	2				no_annot
5039	atattacatt	2	TC11665	47	0	
5040	gggttgaacg	2	TC11256	27	0	weakly similar to GP|15426576|gb|AAH13409.1|AAH13409|BC013409 transcriptional regulator protein {Homo sapiens}, partial (23%)
5041	tcaattcgac	2	C208657.1	65	0	
5042	acggaataaa	2				no_annot
5043	ggatgactgg	2				no_annot
5044	taaattgaca	2	TC8400	71	3	weakly similar to PIR|S65953|S65953 [phosphorylase] phosphatase65K regulatory chain isotype alpha - African clawed frog, partial (25%)
5045	agacaacgca	2	C201396.1	29	1	
5046	gtctattact	2				no_annot
5047	gaatttgtag	2	C304964.1	89	0	
5048	taaaatgtga	2				no_annot
5049	aaaccaataa	2				no_annot
5050	ctcttctacg	2				no_annot
5051	gtcatctaaa	2	TC9646	31	2	similar to GP|3320019|emb|CAA04068.1||AJ000420 cytochrome b {Sorex coronatus}, partial (5%)
5052	cgtgcgctta	2				no_annot
5053	ttatctgtcg	2				no_annot
5054	atccattgga	2	TC18163	50	0	weakly similar to GP|7766908|pdb|1EH5|A Chain A, Crystal Structure Of Palmitoyl Protein Thioesterase 1 Complexed With Palmitate, partial (40%)
5055	cgtgaattgt	2				no_annot
5056	ttcgtttaga	2				no_annot
5057	atgctcctat	2	TC13780	98	0	similar to SP|O08810|U5S1_MOUSE 116 kDa U5 small nuclear ribonucleoprotein component (U5 snRNP-specific protein, 116 kDa) (U5-116 kDa). [Mouse] {Mus musculus}, partial (78%)

5058	gctaatattt	2	CD167178	68	1	weakly similar to PIR|T30810|T30810 chromatin structure regulator Supt6h - mouse, partial (4%)
5059	agataaaatg	2	TC15362	97	0	weakly similar to GP|21430882|gb|AAM51119.1||AY119259 SD22572p {Drosophila melanogaster}, partial (32%)
5060	gaattcctca	2	TC18081	86	0	weakly similar to GP|23093177|gb|AAF49213.2||AE003519 CG6843-PA {Drosophila melanogaster}, partial (20%)
5061	tcattactat	2				no_annot
5062	atgatagaat	2				no_annot
5063	ttacaggcac	2	C311814.1	48	2	
5064	gagtgaagga	2				no_annot
5065	cagcgtagct	2				no_annot
5066	agtcgtagtg	2	gi|3599492|gb|AF085145.1|AF085145	47	3	Schistosoma mansoni NADH dehydrogenase subunit 5 (NU5M) mRNA, complete cds, mitochondrial gene encoding mitochondrial protein
5067	tggtaacaaa	2	CD154434	80	0	
5068	atttaaatgt	2	TC10664	97	0	similar to GP|16769280|gb|AAL28859.1||AY061311 LD23129p {Drosophila melanogaster}, partial (16%)
5068	atttaaatgt	2	TC11001	97	0	weakly similar to GP|15292337|gb|AAK93437.1||AY052013 LD47309p {Drosophila melanogaster}, partial (5%)
5069	taaatccaat	2	C315885.1	78	0	
5069	taaatccaat	2	C704620.1	78	0	
5070	taagtggaaa	2	TC14661	90	0	
5070	taagtggaaa	2	CD095762	81	0	
5071	tatcgctcta	2				no_annot
5072	tgcttatgta	2				no_annot
5073	aggctattat	2	TC7914	84	0	
5074	ttattttcaa	2	TC8272	97	0	weakly similar to GP|21428902|gb|AAM50170.1||AY119516 GH14470p {Drosophila melanogaster}, partial (31%)
5074	ttattttcaa	2	TC8841	24	1	
5075	tactggtccc	2				no_annot
5076	tttggatgta	2				no_annot

5077	tttattgtta	2	TC8179	96	0	
5077	tttattgtta	2	TC8180	93	0	
5078	tcatatgtct	2				no_annot
5079	tatgtgtata	2	TC16334	45	2	
5080	ttttatgtaa	2	AF314754|Sma.85	98	0	Insulin receptor protein kinase RTK-2 (RTK-2)
5081	tttatgttgg	2				no_annot
5082	catacatata	2				no_annot
5083	ataaacgttt	2				no_annot
5084	gcaaatgtat	2				no_annot
5085	tcacagacga	2				no_annot
5086	gcctgaggct	2	C301780.1	12	0	
5086	gcctgaggct	2	C310829.1	31	0	
5086	gcctgaggct	2	C705761.1	31	0	
5086	gcctgaggct	2	C705834.1	12	0	
5087	cttttccaaa	2				no_annot
5088	gcaaatgtaa	2				no_annot
5089	taaaacctat	2				no_annot
5090	acgcttataa	2				no_annot
5091	attgaattaa	2	TC8774	88	0	
5092	cctttctaaa	2				no_annot
5093	tactgataaa	2	TC14019	47	0	
5094	tttatccttt	2				no_annot
5095	cgcacacaaa	2	C301581.1	14	1	
5095	cgcacacaaa	2	C603469.1	7	2	
5096	tctgcatagt	2	C202157.1	95	0	
5096	tctgcatagt	2	C601498.1	38	2	
5096	tctgcatagt	2	C608298.1	94	0	
5097	gtagcgcttg	2				no_annot
5098	tttgttcact	2				no_annot

5099	taatccgtta	2	TC13900	89	0	weakly similar to SP|Q26619|KAPR_STRPU cAMP-dependent protein kinase type II regulatory chain. [Purple sea urchin] {Strongylocentrotus purpuratus}, partial (67%)
5100	acatacaaga	2				no_annot
5101	tacccaggat	2				no_annot
5102	actattcagg	2				no_annot
5103	ttgggcattc	2	Z32529|Sma.852	39	4	Cathepsin L
5104	aggctccgtc	2				no_annot
5105	cacccaggcc	2	C308058.1	37	0	
5105	cacccaggcc	2	C607252.1	3	5	
5105	cacccaggcc	2	C716981.1	37	0	
5106	tttgctttaa	2				no_annot
5107	tttgatctgc	2				no_annot
5108	tgctggacta	2				no_annot
5109	gtggtttgca	2				no_annot
5110	aagagattat	2				no_annot
5111	aaaaacaaaa	2	TC9105	86	0	
5111	aaaaacaaaa	2	TC17581	84	2	weakly similar to GP|12862302|dbj|BAB32409.1||AB047003 caspase-3 {Danio rerio}, partial (21%)
5111	aaaaacaaaa	2	CD072808	53	0	
5111	aaaaacaaaa	2	CD179176	44	1	
5112	tataatatca	2	TC9135	18	2	
5113	atttataatt	2	TC7862	74	0	similar to SP|O15258|RER1_HUMAN RER1 protein. [Human] {Homo sapiens}, partial (52%)
5114	ttgccaccaa	2				no_annot
5115	aaggaggccg	2				no_annot
5116	ttgagaaaac	2				no_annot
5117	gaacagtgtg	2	C200146.1	28	1	
5118	atttataatc	2	TC11706	50	0	homologue to GP|7290473|gb|AAF45927.1||AE003430 CG15570-PA {Drosophila melanogaster}, partial (3%)

5118	atttataatc	2	CD075154	44	0	homologue to PIR|A82704|A82704 1,4-beta-cellobiosidase XF1267 [imported] - Xylella fastidiosa (strain 9a5c), partial (5%)
5119	cagttatcaa	2	TC7761	34	9	
5120	gtgtaataac	2				no_annot
5121	tagtctgata	2	CD086935	41	0	
5122	ttgttgacca	2	TC15485	50	3	weakly similar to GP|28866788|dbj|BAC65172.1||AB050010 Surf4 {Gallus gallus}, partial (31%)
5123	aacaagataa	2				no_annot
5124	actttcacat	2				no_annot
5125	tgatctaagt	2	TC11771	61	0	
5126	aaaaagagat	2	TC10235	80	0	weakly similar to SP|P53602|ER19_HUMAN Diphosphomevalonate decarboxylase(Mevalonate pyrophosphate decarboxylase) (Mevalonate (diphospho)decarboxylase). [Human] {Homo sapiens}, partial (22%)
5127	cacatttgta	2				no_annot
5128	gatggaacgc	2	TC10734	15	3	
5129	tgaatgtagt	2	TC14865	75	2	weakly similar to GP|34226|emb|CAA41418.1||X58531 laminin A chain {Homo sapiens}, partial (3%)
5130	gtcagtgatt	2	TC7679	66	6	weakly similar to GP|28630303|gb|AAM92833.1||AY130428 protein kinase C {Branchiostoma lanceolatum}, partial (7%)
5131	gcattctctc	2				no_annot
5132	taacattgaa	2				no_annot
5133	cgataactat	2	TC13179	16	0	
5134	gatctatgtg	2				no_annot
5135	tctattattc	2				no_annot
5136	ttcctatccg	2	TC11677	83	0	
5137	ttaaaaaaaa	2				no_annot
5138	actgacagga	2				no_annot
5139	agatatgctg	2	TC14383	57	2	weakly similar to GP|15128553|dbj|BAB62747.1||AB055493 UDP-galactose transporter {Drosophila melanogaster}, partial (38%)

5139	agatatgctg	2	CD068270	11	1	weakly similar to SP|Q9Y2D2|UGNT_HUMAN UDP N-acetylglucosamine transporter (Golgi UDP-GlcNAc transporter). [Human] {Homo sapiens}, partial (16%)
5140	tgatagcggt	2				no_annot
5141	gaatatacca	2	TC14091	25	0	
5142	tttttggttt	2	TC19271	91	0	
5143	gcccctgcgc	2	TC14586	22	0	
5144	taatttacta	2	CD166460	70	1	
5144	taatttacta	2	CD096187	7	1	weakly similar to SP|O34338|MNTB_BACSU Manganese transport system ATP-binding protein mntB. {Bacillus subtilis}, partial (10%)
5145	tgaaatgttt	2	CD201489	73	0	
5146	taattatgta	2	C301983.1	0	1	
5147	gacgataact	2				no_annot
5148	tatcgttctg	2				no_annot
5149	taatacagta	2				no_annot
5150	ccatttcagt	2				no_annot
5151	ccagatctat	2	TC9236	54	2	similar to GP|1684843|gb|AAB48302.1||U77717 pinin {Bos taurus}, partial (4%)
5152	ttttctttcg	2				no_annot
5153	atgagtgttg	2				no_annot
5154	tttattgtcc	2				no_annot
5155	gcaatggttt	2	TC11210	77	0	weakly similar to SP|O08950|T2AG_RAT Transcription initiation factor IIA gamma chain (TFIIA P12 subunit) (TFIIA-12) (TFIIAS) (TFIIA-gamma). [Rat] {Rattus norvegicus}, partial (38%)
5155	gcaatggttt	2	CD186084	70	0	
5156	tccctacatc	2				no_annot
5157	aaaacagcaa	2	CD157225	44	1	
5158	cgcctattcc	2				no_annot
5159	tacaaacgtt	2				no_annot
5160	ggtcgatcgg	2	C208313.1	9	5	

5161	atttcagcca	2	CD133853	13	2	weakly similar to GP|17945700|gb|AAL48899.1||AY071277 RE30690p {Drosophila melanogaster}, partial (32%)
5161	atttcagcca	2	CD133853	13	1	weakly similar to GP|17945700|gb|AAL48899.1||AY071277 RE30690p {Drosophila melanogaster}, partial (32%)
5162	attatctatt	2				no_annot
5163	aagttctacc	2				no_annot
5164	accattcggg	2				no_annot
5165	atctaagcaa	2				no_annot
5166	ttcggtgaat	2	TC17168	97	0	similar to GP|14919411|gb|AAH05728.1||BC005728 microfibrillar-associated protein 1 {Mus musculus}, partial (47%)
5166	ttcggtgaat	2	TC19448	78	1	
5167	atatgtgtgg	2				no_annot
5168	cttcagtcac	2	C603157.1	96	0	
5169	tacagattgt	2	CD076100	93	0	weakly similar to GP|6066445|emb|CAB58387.1||AL121861 possible proline synthetase associated protein {Leishmania major}, partial (15%)
5170	gatcctgttg	2	TC11490	75	1	similar to GP|7158855|gb|AAF37565.1|AF221506_3|AF221506 scavenger receptor protein CI precursor {Drosophila melanogaster}, partial (4%)
5171	cacataccaa	2				no_annot
5172	aggctttacc	2				no_annot
5173	cgacagctct	2				no_annot
5174	aaagaagtgg	2				no_annot
5175	tagcacaaac	2	TC16931	58	0	
5176	acatcagcaa	2				no_annot
5177	ctcaagaaca	2				no_annot
5178	cataatttaa	2	TC18866	96	0	
5179	cagacgtttt	2	C202256.1	70	0	
5179	cagacgtttt	2	C716105.1	73	0	
5180	atgacttatt	2	C207562.1	78	0	

5181	tcccgtcgaa	2	TC10890	72	0	weakly similar to GP|20151369|gb|AAM11044.1||AY094691 GH08974p {Drosophila melanogaster}, partial (35%)
5182	aagcgtcagt	2	CD133809	62	0	
5183	ccaacagtca	2				no_annot
5184	gataacgtga	2	CD120419	20	0	
5185	ctaagaataa	2	TC19086	10	0	
5186	atccttggtc	2				no_annot
5187	tcagtcagtg	2	C200841.1	76	0	
5187	tcagtcagtg	2	C202109.1	9	5	
5187	tcagtcagtg	2	C602518.1	9	5	
5187	tcagtcagtg	2	C607553.1	52	0	
5188	caaggaggta	2	C201078.1	69	0	
5188	caaggaggta	2	C606050.1	65	1	
5189	tctacgagtc	2				no_annot
5190	tattcagata	2	TC16446	26	1	
5190	tattcagata	2	TC17197	93	0	weakly similar to SP|Q94497|SELD_DICDI Selenide,water dikinase(Selenophosphate synthetase) (Selenium donor protein) (Fragment). [Slime mold] {Dictyostelium discoideum}, partial (58%)
5191	aaaagagaaa	2				no_annot
5192	ttggactaaa	2				no_annot
5193	ttgtacaaac	2				no_annot
5194	tcaacacttg	2	TC8099	50	0	
5195	atctgaaagt	2	TC10148	14	2	
5196	tttaccgaat	2				no_annot
5197	cagtatacat	2	C608973.1	44	1	
5198	taactcaact	2				no_annot
5199	aaggcatact	2	C313957.1	18	0	
5200	tgaaatgtgt	2				no_annot
5201	aggaaatagg	2				no_annot

5202	tacatttgaa	2	TC17844	94	0	weakly similar to GP|5919219|gb|AAD56248.1|AF186273_1|AF186273 leucine-rich repeats containing F-box protein FBL3 {Homo sapiens}, partial (8%)
5203	agatgattga	2				no_annot
5204	gtgtggtgag	2				no_annot
5205	tcccagtaca	2				no_annot
5206	gaaatgcaca	2	TC13211	88	0	similar to GP|18000909|gb|AAL55210.1||AF280305 cytochrome b {Mabuya fogoensis nicolauensis}, partial (6%)
5207	tgggtcctaa	2	TC10661	90	0	similar to GP|632500|gb|AAB50269.1||U17394 polyadenylation factor 64 kDa subunit {Xenopus laevis}, partial (32%)
5207	tgggtcctaa	2	TC10662	38	0	similar to GP|632500|gb|AAB50269.1||U17394 polyadenylation factor 64 kDa subunit {Xenopus laevis}, partial (18%)
5208	ttgtcagcgt	2				no_annot
5209	agctgaactg	2				no_annot
5210	tcgtcgatca	2				no_annot
5211	tcgacacaat	2				no_annot
5212	ctgatatttt	2	TC14740	42	0	
5213	acacttgaat	2				no_annot
5214	aaggatttat	2	CD064236	35	0	
5215	aacaatgtac	2				no_annot
5216	atttgacata	2				no_annot
5217	taatatagtt	2				no_annot
5218	tttgtagcga	2				no_annot
5219	ttattattgt	2	AF029222|Sma.889	90	0	Sm29 (Sm29)
5220	ttgtttgcat	2				no_annot
5221	tgtgtgggtg	2	CD132819	17	2	
5222	tattctattc	2				no_annot
5223	tagatgcagt	2	TC17843	61	0	weakly similar to SP|Q60936|CABC_MOUSE Chaperone-activity of bc1 complex-like, mitochondrial precursor (Chaperone-ABC1-like). [Mouse] {Mus musculus}, partial (46%)

5223	tagatgcagt	2	AI740337	22	0	
5224	tggactggaa	2	TC11132	50	0	weakly similar to GP|21464322|gb|AAM51964.1||AY121637 HL04344p {Drosophila melanogaster}, partial (52%)
5225	tgtctgcgat	2				no_annot
5226	tatatgtttc	2	TC8252	71	3	similar to GP|5138930|gb|AAD40382.1||AF093680 transcription factor IIB {Homo sapiens}, partial (97%)
5227	gagtgaatta	2	TC17882	66	0	similar to GP|5817310|gb|AAD52698.1|AF091537_1|AF091537 very low density lipoprotein binding protein precursor {Schistosoma japonicum}, partial (96%)
5228	ggataaaggc	2				no_annot
5229	tggtaggaat	2				no_annot
5230	agtcagtgtt	2				no_annot
5231	gtatataaac	2				no_annot
5232	aacttggagt	2				no_annot
5233	gctgccttcc	2				no_annot
5234	ggtgttttca	2				no_annot
5235	tgcacaagtg	2				no_annot
5236	ttactgatcg	2				no_annot
5237	ttcgtaacga	2				no_annot
5238	ccgggaggta	2				no_annot
5239	tgaaacctat	2				no_annot
5240	tcacgccgta	2				no_annot
5241	ccttgtgacg	2	C202087.1	8	10	
5241	ccttgtgacg	2	C602027.1	8	10	
5242	tgcttaattg	2	AI395626	9	2	
5243	ttttgttcat	2				no_annot
5244	gtctcagaag	2	TC7872	68	1	weakly similar to SP|O77264|RTC1_DROME RNA 3'-terminal phosphate cyclase(RNA-3'-phosphate cyclase) (RNA cyclase). [Fruit fly] {Drosophila melanogaster}, partial (14%)
5245	tagatatgcc	2				no_annot
5246	gactatcgtt	2	C209062.1	61	0	

5247	attcaatcat	2				no_annot
5248	gaacattaaa	2				no_annot
5249	ctgagacgac	2	C609087.1	26	1	
5250	ttgtagtgat	2				no_annot
5251	ggttcaatat	2				no_annot
5252	tgcaaaaaac	2				no_annot
5253	atgtttgtaa	2	TC12791	74	0	
5254	ttttaaaatg	2	TC13708	80	0	
5254	ttttaaaatg	2	TC17666	84	0	similar to SP|Q9VAF0|Y816_DROME Hypothetial protein CG7816. [Fruit fly] {Drosophila melanogaster}, partial (29%)
5254	ttttaaaatg	2	TC19329	37	0	
5255	cagtgttgtc	2				no_annot
5256	gtaggtttct	2	TC10613	91	0	weakly similar to GP|12006785|gb|AAG44917.1|AF290330_1|AF290330 phosphoglucomutase {Drosophila melanogaster}, partial (52%)
5257	tgaagcaccc	2				no_annot
5258	aatgaattgt	2				no_annot
5259	agtaacagtt	2	TC13191	76	0	
5260	gcggtgtctc	2				no_annot
5261	tctcctgaat	2				no_annot
5262	gcaaaaaatt	2	CD073303	43	0	
5262	gcaaaaaatt	2	CD076339	86	0	
5263	ttattgtggt	2				no_annot
5264	tcaaatacag	2	CD145499	66	0	
5265	aagaagaaga	2	TC10786	35	10	weakly similar to GP|17940124|gb|AAL49499.1|AF408422_1|AF408422 beta-catenin {Platynereis dumerilii}, partial (54%)
5265	aagaagaaga	2	TC17529	6	4	weakly similar to GP|17862592|gb|AAL39773.1||AY069628 LD39967p {Drosophila melanogaster}, partial (17%)
5265	aagaagaaga	2	CD077202	24	0	
5266	tattcttttt	2	CD162471	20	0	

5267	tttactgaat	2				no_annot
5268	actagaattg	2	C208753.1	16	1	
5268	actagaattg	2	C606058.1	30	1	
5269	gacgtgaaga	2				no_annot
5270	atttgtttct	2	AY158216|Sma.689	93	0	Cdc42 (Cdc42)
5271	ggcgactaaa	2				no_annot
5272	tttagtagta	2				no_annot
5273	atcaaaaaaa	2				no_annot
5274	tttagcagaa	2				no_annot
5275	gattatcaaa	2	TC17416	97	0	
5276	gcctatttta	2				no_annot
5277	ttaccgaatg	2	C300853.1	93	0	
5277	ttaccgaatg	2	C706924.1	93	0	
5278	caccgtcaca	2				no_annot
5279	tacatcatct	2				no_annot
5280	tagcttccct	2	TC13051	91	1	
5280	tagcttccct	2	TC19551	38	0	
5281	gtccagacaa	2				no_annot
5282	gtggttatat	2				no_annot
5283	caacttaaaa	2	TC14315	1	1	similar to GP|26801231|emb|CAD37993.1||AJ493124 NADH dehydrogenase subunit 2 {Varroa destructor}, partial (6%)
5283	caacttaaaa	2	CD197071	1	2	
5284	aaactatgta	2	TC11228	10	4	
5285	ggaattactt	2				no_annot
5286	tatgcacaga	2				no_annot
5287	gcatataggc	2				no_annot
5288	gagactcaat	2				no_annot
5289	tgcgtattgt	2				no_annot
5290	gtgcatcact	2				no_annot
5291	tatatatcta	2	C209911.1	30	1	

5292	gcttatgtac	2				no_annot
5293	gccaaagttc	2				no_annot
5294	tgtattcgtt	2	C612021.1	0	2	
5295	caggttgctc	2	TC10955	62	0	similar to GP|23273556|gb|AAH35974.1||BC035974 DEAD/H (Asp-Glu-Ala-Asp/His) box polypeptide 15 {Homo sapiens}, partial (34%)
5296	tatgatctgt	2				no_annot
5297	ttatatatgg	2	CD130230	5	0	
5297	ttatatatgg	2	CD116781	51	0	similar to GP|1617040|emb| Evx-2 {Danio rerio}, partial (3%)
5298	acgatgaata	2				no_annot
5299	catatccttg	2				no_annot
5300	ggataatgaa	2				no_annot
5301	tattctataa	2				no_annot
5302	gtaattaaag	2				no_annot
5303	tacacaatta	2	CD119925	53	0	similar to PIR|H69369|H69369 branched-chain amino acid ABC transporter, permease protein (braE-3) homolog - Archaeoglobus fulgidus, partial (5%)
5304	ttatgagtga	2				no_annot
5305	aatatcaatg	2	TC11847	92	0	weakly similar to GP|21703244|gb|AAM76119.1|AF483039_1|AF483039 Y38C1AA-like protein {Boltenia villosa}, partial (30%)
5306	tgtaatttca	2	TC14336	98	0	weakly similar to GP|19343763|gb|AAH25586.1||BC025586 cDNA sequence BC025586 {Mus musculus}, partial (30%)
5307	tctttatctt	2	TC7558	74	0	similar to GP|19067879|gb|AAK14815.1||AY027869 polyprotein {Schistosoma japonicum}, partial (12%)
5307	tctttatctt	2	TC7618	33	0	weakly similar to GP|10120972|pdb|1C9Q|A Chain A, Average Nmr Solution Structure Of The Bir-2 Domain Of Xiap, partial (21%)
5308	cttgtttgtt	2				no_annot
5309	tgacgacgga	2				no_annot
5310	aaatcctagt	2				no_annot
5311	atggaatgaa	2				no_annot
5312	atgtaaagag	2				no_annot

5313	aaaatcgagt	2				no_annot
5314	ataactttat	2				no_annot
5315	atattatgtc	2	TC11179	33	0	similar to GP|3721565|dbj|BAA33547.1||AB012611 Hrs binding Protein {Mus musculus}, partial (9%)
5316	tcattacctg	2				no_annot
5317	aaattacaac	2	TC7692	19	1	weakly similar to GP|7573635|dbj|BAA94544.1||AB039933 polyposis locus protein 1-like 1 (TB2 protein-like 1) {Mus musculus}, partial (24%)
5318	ttatcaaaag	2				no_annot
5319	aagtgtgacg	2				no_annot
5320	tgtaatttaa	2				no_annot
5321	ggttccggaa	2	TC19323	28	0	
5322	agaatacggc	2	TC7464	34	2	similar to GP|15213788|gb|AAK92169.1|AF400197_1|AF400197 ribosomal protein L35A {Spodoptera frugiperda}, partial (65%)
5323	ccctttatta	2				no_annot
5324	ttcagcctca	2	TC9360	69	0	
5325	gagcaacttc	2	AF521094|Sma.5608	23	2	Clone p83H3 unknown mRNA
5325	gagcaacttc	2	AF521094|Sma.5608	23	1	Clone p83H3 unknown mRNA
5326	gtgttacatc	2				no_annot
5327	gaaaattggt	2	TC7774	40	0	similar to SP|P77225|YDHR_ECOLI Protein ydhR precursor. {Shigella flexneri}, partial (16%)
5328	gtcacagtgc	2				no_annot
5329	catagtcaca	2				no_annot
5330	actagaatgc	2				no_annot
5331	tctactaaaa	2				no_annot
5332	gtattgggtg	2				no_annot
5333	gcaacaagtc	2				no_annot
5334	aatagcccct	2				no_annot
5335	ttcacttact	2				no_annot
5336	aatctcgaag	2				no_annot
5337	cgtttacttt	2				no_annot

5338	aaaatcgaca	2	CD084097	45	0	similar to GP|16648426|gb|AAL25478.1||AY060439 LD46868p {Drosophila melanogaster}, partial (11%)
5339	ctatatatat	2				no_annot
5340	atgtaatgga	2				no_annot
5341	gacacataaa	2	TC15881	63	0	
5342	gttcatttga	2				no_annot
5343	ttaatagagt	2				no_annot
5344	accaccgaca	2	TC16925	53	1	weakly similar to GP|3005587|gb|AAC09321.1||AF048977 Ser/Arg-related nuclear matrix protein {Homo sapiens}, partial (20%)
5345	tgattttctc	2				no_annot
5346	atgaaagcta	2				no_annot
5347	catccgcatc	2				no_annot
5348	tattctttca	2	TC11788	6	2	similar to GP|21647772|gb|AAM72982.1||AE012929 iron(III) ABC transporter, permease protein {Chlorobium tepidum TLS}, partial (6%)
5349	ttatatttgt	2	TC8019	96	0	
5350	ggaagcgctg	2	TC16761	93	0	
5351	atcttcaaga	2				no_annot
5352	ttaccagtac	2				no_annot
5353	caacagccca	2				no_annot
5354	cactatttac	2	CD145538	61	0	weakly similar to GP|29896645|gb|AAP09924.1||AE017007 Pyrroline-5-carboxylate reductase {Bacillus cereus ATCC 14579}, partial (26%)
5355	acagctattg	2				no_annot
5356	atcttgtgtt	2				no_annot
5357	aacagttcgt	2	TC17071	35	2	
5357	aacagttcgt	2	TC17073	44	2	
5358	tctgctgatg	2				no_annot
5359	acagtactgg	2				no_annot
5360	tcattttgtt	2	TC7065	45	4	
5361	gtctgtacga	2	C207958.1	16	2	
5361	gtctgtacga	2	C606669.1	20	1	

5362	ggtaagttgt	2				no_annot
5363	aagggggttt	2				no_annot
5364	tctcctcaag	2	TC7973	85	0	
5365	atgcaattaa	2	TC15417	96	0	
5366	tgtttgcgtt	2				no_annot
5367	tgaagttcga	2				no_annot
5368	tatccaagcc	2	TC11742	92	0	
5369	gtgctgcata	2				no_annot
5370	tatgtggtat	2				no_annot
5371	tacaagaaag	2				no_annot
5372	gttgaagatg	2				no_annot
5373	gcttttcaaa	2				no_annot
5374	cagtaaatac	2				no_annot
5375	actttctata	2	TC8733	51	1	
5375	actttctata	2	TC9488	81	0	
5376	ctacatacta	2				no_annot
5377	caattattta	2	TC14163	88	0	weakly similar to GP|14530319|emb|CAC42251.1||Z79596 C. elegans DYN-1 protein (corresponding sequence C02C6.1b) {Caenorhabditis elegans}, partial (23%)
5378	tacccgtaca	2				no_annot
5379	ttaaagaagc	2	TC10543	55	3	homologue to GP|21686538|gb|AAM74948.1|AF519808_1|AF519808 MA {Schistosoma japonicum}, partial (83%)
5379	ttaaagaagc	2	TC17146	35	4	similar to GP|16226045|gb|AAL16062.1|AF420278_1|AF420278 carbonyl reductase {Anguilla japonica}, partial (16%)
5380	attattcggg	2				no_annot
5381	gtgtttttcg	2	C611452.1	40	0	
5382	tcctcaatgc	2	TC13649	76	0	
5382	tcctcaatgc	2	TC13650	63	1	
5383	ctaatcttat	2				no_annot
5384	aagaaactaa	2	TC18609	66	0	

5385	actgtattac	2				no_annot
5386	tttatatatc	2				no_annot
5387	tggagaaatt	2	gi|28261408|tpg|BK000685.1|	94	0	TPA: Schistosoma mansoni penelope-like transposable element Cercyon reverse transcriptase gene, complete cds
5388	taaataccaa	2				no_annot
5389	ttcgaatgct	2				no_annot
5390	agatttatgc	2				no_annot
5391	acatacgtac	2				no_annot
5392	gatcgacgcc	2				no_annot
5393	aattgggcac	2				no_annot
5394	cacaaacttt	2	CD155697	67	0	
5395	gcagtgaggt	2				no_annot
5396	agtatttcag	2				no_annot
5397	ctgcatctgg	2				no_annot
5398	ctaggtgacc	2				no_annot
5399	aaacgatatg	2				no_annot
5400	acattgtaat	2				no_annot
5401	ttctggcttt	2	TC18320	11	0	
5402	acgatactta	2				no_annot
5403	tgtagccact	2	TC15535	63	0	
5404	ttttaacgtc	2				no_annot
5405	tacattttgt	2				no_annot
5406	acgctttcta	2				no_annot
5407	agcaaatcaa	2				no_annot
5408	attcagactt	2				no_annot
5409	ctagtgttta	2				no_annot
5410	taattcaagt	2	TC15993	10	1	
5410	taattcaagt	2	TC17443	72	4	
5411	aagacgtgaa	2	C208658.1	14	3	
5412	atacacatat	2				no_annot
5413	ttacaaagag	2				no_annot

5414	tgttttgacc	2	C200584.1	46	2	
5414	tgttttgacc	2	C600911.1	52	2	
5414	tgttttgacc	2	C602112.1	90	0	
5415	aataccgctg	2	TC17185	7	0	weakly similar to SP|P18747|ZO28_XENLA Oocyte zinc finger protein XLCOF28 (Fragment). [African clawed frog] {Xenopus laevis}, partial (5%)
5416	tagatgagtc	2	C611311.1	32	0	
5417	caaagcaaat	2	TC11651	57	1	
5418	ttatgactgt	2				no_annot
5419	caagtattac	2	TC14045	44	3	weakly similar to SP|P08621|RU17_HUMAN U1 small nuclear ribonucleoprotein 70 kDa (U1 snRNP 70 kDa) (snRNP70) (U1-70K). [Human] {Homo sapiens}, partial (17%)
5419	caagtattac	2	TC17099	70	2	
5419	caagtattac	2	CD078403	21	2	
5420	aattgtatgc	2				no_annot
5421	tctgttctct	2				no_annot
5422	ctaaagaaaa	2	C201079.1	3	2	
5423	tgccacactt	2	TC10524	98	0	
5424	ttgttgaaaa	2	TC17015	11	6	
5425	cagctcgaaa	2				no_annot
5426	tatcatcctg	2	CD145118	68	0	
5427	ttaacaataa	2	CD075795	62	0	GP|27374368|gb|AAO01107.1||AY190959 CG30421-PA {Drosophila willistoni}, partial (5%)
5428	ccaccaatag	2	C202229.1	28	4	
5428	ccaccaatag	2	C301167.1	27	3	
5428	ccaccaatag	2	C604342.1	41	4	
5428	ccaccaatag	2	C716005.1	27	3	
5429	atcgccgcag	2	TC10724	94	0	weakly similar to PIR|S57447|S57447 HPBRII-7 protein - human, partial (32%)
5430	cagagtcctc	2				no_annot
5431	gttgttggtg	2				no_annot
5432	cttaaaatgt	2	TC8252	97	0	similar to GP|5138930|gb|AAD40382.1||AF093680 transcription factor IIB {Homo sapiens}, partial (97%)

5433	aatattggtt	2				no_annot
5434	cctacgattg	2	TC17862	53	0	weakly similar to GP|21627004|gb|AAF57625.2||AE003798 CG15100-PA {Drosophila melanogaster}, partial (5%)
5435	gtaaccccaa	2	AI394873	44	0	
5436	ttgtaaactg	2				no_annot
5437	attataacta	2	gi|425475|gb|L26287.1|SCMSMDR2AA	94	0	Schistosoma mansoni SMDR2 gene, complete cds
5438	ttgatgattt	2	TC11326	67	0	similar to GP|19343890|gb|AAH25585.1||BC025585 Ugp2 protein {Mus musculus}, partial (53%)
5439	atgatgcaaa	2				no_annot
5440	ctcctataaa	2	TC11555	91	0	weakly similar to GP|13874419|dbj|BAB46916.1||D87060 cerebral protein-1 {Homo sapiens}, partial (14%)
5441	gctttggtcc	2	C717275.1	84	0	
5442	atttaaacac	2				no_annot
5443	ttgaggcgtt	2				no_annot
5444	acaatcgtga	2				no_annot
5445	ccctcgagga	2				no_annot
5446	ctgtaaacta	2	TC14461	24	1	similar to GP|11464653|gb|AAG35265.1|AF215933_1|AF215933 Smad1 {Schistosoma mansoni}, partial (59%)
5446	ctgtaaacta	2	CD195273	23	0	weakly similar to GP|11464653|gb|AAG35265.1|AF215933_1|AF215933 Smad1 {Schistosoma mansoni}, partial (37%)
5447	caattcaaaa	2				no_annot
5448	gattggtcta	2				no_annot
5449	aaggcacttg	2	TC10502	40	6	similar to GP|2661460|emb|CAA05921.1||AJ003165 ABI3 {Populus balsamifera subsp. trichocarpa}, partial (3%)
5450	ttttgtggtt	2	TC13689	69	0	
5451	gcagtttatt	2				no_annot
5452	tagtttcctt	2				no_annot
5453	tggagaaagt	2				no_annot
5454	gttcgccaca	2	TC9037	64	0	
5455	tggtgtagag	2				no_annot

5456	tcattttgat	2	TC8035	19	4	
5456	tcattttgat	2	TC10007	33	0	
5457	acgaccatca	2	M15371|Sma.619	55	4	Eggshell protein mRNA, 3 end
5457	acgaccatca	2	gi|161049|gb|M74170.1|SCMP48EGG	86	4	Schistosoma mansoni p48 eggshell protein gene, complete cds
5458	taagcaaagg	2	C201056.1	42	5	
5458	taagcaaagg	2	C612592.1	42	5	
5459	taagcaaaga	2	CD186601	67	0	
5460	cctgctgaat	2	C209921.1	43	0	
5460	cctgctgaat	2	C309700.1	90	0	
5460	cctgctgaat	2	C608670.1	43	0	
5461	ctaacattgc	2				no_annot
5462	taactgatac	2	CD197027	41	1	
5463	ttagcagtta	2				no_annot
5464	atcggcgacg	2	TC11399	83	0	
5465	ttctggctga	2				no_annot
5466	cgaccaaaca	2				no_annot
5467	cacaatgaga	2				no_annot
5468	tatcgtaact	2				no_annot
5469	actctgttgc	2	TC10519	83	0	
5470	aagttatcga	2	C717625.1	56	1	
5471	aggaagggga	2				no_annot
5472	aattgtttgg	2				no_annot
5473	tacagaacgc	2				no_annot
5474	tagatattgt	2	TC11677	2	7	
5475	cagctactgt	2				no_annot
5476	gaatcctgaa	2	AY838777|Sma.5389	21	2	Clone F04 Perere 3 retrotransposon mRNA, partial sequence
5476	gaatcctgaa	2	AY838779|Sma.5390	26	0	Clone H10 Perere 3 retrotransposon mRNA, partial sequence
5476	gaatcctgaa	2	AY838780|Sma.5391	30	0	Clone H09 Perere 3 retrotransposon mRNA, partial sequence

5477	tgtatgttag	2				no_annot
5478	aaccttggag	2				no_annot
5479	aaattggaga	2				no_annot
5480	caagcgacac	2				no_annot
5481	tacactaagg	2	TC9718	49	0	
5481	tacactaagg	2	TC18048	35	1	
5482	atagattgct	2				no_annot
5483	ttaatacaaa	2				no_annot
5484	agcagtacgg	2	C207051.1	3	2	
5485	gaaaaagaga	2				no_annot
5486	cataatacac	2				no_annot
5487	gaaaacttgg	2				no_annot
5488	ttggctttta	2				no_annot
5489	ttattgataa	2				no_annot
5490	ttggttcata	2				no_annot
5491	cacaatgaaa	2				no_annot
5492	gattattgtt	2	CD193978	66	0	
5493	caagcaaaac	2				no_annot
5494	ctccactaat	2	AY158215|Sma.78	92	0	Rho3 GTPase (Rho3)
5495	aagtaatgtg	2				no_annot
5496	tacagaacaa	2	TC10129	33	0	weakly similar to SP|P40112|PSB3_RAT Proteasome subunit beta type 3(Proteasome theta chain) (Proteasome chain 13) (Proteasome component C10-II). [Rat] {Rattus norvegicus}, partial (40%)
5496	tacagaacaa	2	TC14949	82	0	
5496	tacagaacaa	2	TC18243	0	2	weakly similar to SP|Q23237|PSB3_CAEEL Proteasome subunit beta type 3(Proteasome subunit beta 3). {Caenorhabditis elegans}, partial (19%)
5497	cacagaaccg	2				no_annot
5498	tatttagccc	2				no_annot
5499	ttgatgatga	2				no_annot
5500	cggcgtttct	2	C604187.1	68	0	

5501	gcgtgcttca	2	TC10791	8	4	
5502	aacgtaatca	2	TC11142	64	3	
5503	ttattgttgt	2	TC15738	67	0	weakly similar to GP|3288147|emb|CAA06415.1||AJ005174 GAGA factor class A-isoform {Drosophila virilis}, partial (4%)
5504	tgctcataac	2				no_annot
5505	gaaaaagaag	2	TC14367	37	4	weakly similar to GP|22946910|gb|AAN11076.1||AE003666 CG31683-PA {Drosophila melanogaster}, partial (13%)
5506	tggtttgctt	2				no_annot
5507	caaacttcaa	2				no_annot
5508	ttaaatgaaa	2	TC13874	97	0	weakly similar to GP|9931608|gb|AAG02229.1|AF293979_1|AF293979 transcriptional coactivator MED7 {Drosophila melanogaster}, partial (30%)
5508	ttaaatgaaa	2	AA269312	16	1	
5509	catactcatt	2				no_annot
5510	tatcgtggag	2	C202179.1	6	2	
5511	taatacttaa	2	TC14290	83	0	weakly similar to GP|3360516|gb|AAC69756.1||AF071773 LIM-domain protein {Branchiostoma floridae}, partial (22%)
5512	tgttgctcgt	2				no_annot
5513	actgcataaa	2				no_annot
5514	acgactatgt	2	TC14755	29	0	weakly similar to SP|P19338|NUCL_HUMAN Nucleolin (Protein C23). [Human] {Homo sapiens}, partial (4%)
5515	aatacatagt	2	CD111728	32	1	
5516	atcccagtag	2				no_annot
5517	gttatcttta	2				no_annot
5518	cagctgcggt	2				no_annot
5519	ctttcttcaa	2	AY323529|Sma.76	40	5	ATP-diphosphohydrolase 1 (ATPDase1)
5520	ttttgttagt	2	TC14135	36	0	
5521	ataccaggag	2				no_annot
5522	atagtcggta	2				no_annot
5523	ggtcacattt	2	TC9094	6	4	

5524	aacgtgtgtg	2	TC11651	85	0	
5525	ttccgcttat	2				no_annot
5526	tgttttcaac	2	C201133.1	65	0	
5526	tgttttcaac	2	C606705.1	63	0	
5527	caggcttggc	2				no_annot
5528	accctcttat	2				no_annot
5529	gacattgtgc	2				no_annot
5530	caagtccgcc	2	C203177.1	57	3	
5531	agaaacgtca	2				no_annot
5532	tttcagcgca	2				no_annot
5533	aaaggaaatt	2	TC14009	99	0	similar to PIR|AG0096|AG0096 phosphoenolpyruvate-protein phosphotransferase[imported] - Yersinia pestis (strain CO92), partial (3%)
5534	gggtacccca	2				no_annot
5535	aacaaataca	2	TC11319	41	0	
5535	aacaaataca	2	TC17792	18	2	
5536	gtttttaaac	2	TC10869	81	0	weakly similar to PIR|I68523|I68523 protein tyrosine phosphatase - human (fragment), partial (15%)
5536	gtttttaaac	2	CD075590	13	0	
5537	tggaattggg	2				no_annot
5538	taatcaaaaa	2	TC10672	62	2	
5539	atagtaatcg	2				no_annot
5540	tgagactttt	2				no_annot
5541	ataatttgtg	2	TC18125	24	0	
5542	aaattcacaa	2	TC17687	77	0	weakly similar to SP|P18420|PSA1_RAT Proteasome subunit alpha type 1(Proteasome component C2) (Macropain subunit C2) (Multicatalytic endopeptidase complex subunit C2) (Proteasome nu chain). [Rat] {Rattus norvegicus}, partial (89%)
5543	gatcttggca	2				no_annot
5544	tatgccacac	2				no_annot
5545	ctaagaacat	2				no_annot
5546	tttgtagaca	2				no_annot

5547	acggttactg	2				no_annot
5548	caaagttaca	2				no_annot
5549	tttcgtgtac	2				no_annot
5550	actatagaga	2	C302862.1	13	1	
5551	tgtaagcata	2				no_annot
5552	gaagtgaaaa	2				no_annot
5553	atatattgag	2	CD135112	31	0	
5554	gggtggtggt	2				no_annot
5555	cgattatttt	2				no_annot
5556	gcaaatccaa	2				no_annot
5557	gggaagtgtt	2	TC14354	20	0	weakly similar to GP|12805177|gb|AAH02047.1||BC002047 protoporphyrinogen oxidase {Mus musculus}, partial (9%)
5558	caatccgcac	2				no_annot
5559	aataaacaga	2	TC19128	91	0	
5559	aataaacaga	2	CD202612	30	1	
5560	attattgtaa	2	CD149672	21	2	
5560	attattgtaa	2	CD095109	70	0	
5561	tatctcttgc	2				no_annot
5562	tttaataacg	2	TC10275	92	0	
5563	atagtgtgtg	2				no_annot
5564	gggaacaaca	2	TC8392	36	1	similar to GP|7295594|gb|AAF50904.1||AE003571 CG1676-PA {Drosophila melanogaster}, partial (11%)
5565	ttggtggttt	2	TC10644	44	4	
5566	tattaaaaaa	2				no_annot
5567	gagctggact	2				no_annot
5568	aagtcactgc	2				no_annot
5569	gttcaggaca	2				no_annot
5570	gtgagggaat	2				no_annot
5571	ggatgtatta	2				no_annot
5572	ccgttccgtc	2	TC11382	27	4	

5573	taaccagtca	2				no_annot
5574	taaatcttga	2				no_annot
5575	ggaatctata	2	TC19529	48	0	
5575	ggaatctata	2	CD199467	33	0	similar to SP|Q07422|DRTS_TOXGO Bifunctional dihydrofolate reductase-thymidylate synthase (DHFR-TS) [Includes: Dihydrofolate reductase; Thymidylate synthase]. {Toxoplasma gondii}, partial (5%)
5576	attattcttc	2	TC10888	89	0	weakly similar to GP|18124147|gb|AAL59846.1|AF272946_1|AF272946 endophilin B1b {Mus musculus}, partial (9%)
5577	tgaagtaatc	2	TC17818	82	0	similar to PIR|T49152|T49152 serine/threonine-specific protein kinase-like protein - Arabidopsis thaliana, partial (7%)
5578	taatatttga	2				no_annot
5579	aattttgatc	2	TC17841	86	0	
5579	aattttgatc	2	CD064495	92	0	
5580	agagatcaag	2				no_annot
5581	taattttaaa	2	C201385.1	66	0	
5581	taattttaaa	2	C610532.1	43	1	
5582	tatcagtatc	2				no_annot
5583	tactttcttc	2	TC7944	79	0	
5584	ctacaaattc	2				no_annot
5585	aagatatgtg	2	TC15593	89	0	
5586	tgattaatag	2				no_annot
5587	atttatctaa	2				no_annot
5588	tggcttgatc	2	C603986.1	10	3	
5589	tcgggacttg	2				no_annot
5590	cttatacgat	2	TC7604	38	3	
5591	tgcagcgtga	2				no_annot
5592	tgccttctcc	2	TC7190	64	0	
5593	ccggtttaca	2	TC16819	3	3	
5593	ccggtttaca	2	BG931428	64	1	
5594	gaaagcgaaa	2				no_annot

5595	acaacgaaac	2				no_annot
5596	gattattgac	2				no_annot
5597	atcgtgatcg	2				no_annot
5598	aaaatggtgg	2				no_annot
5599	ttgagtcact	2				no_annot
5600	ttatttgtac	2	TC16908	85	0	SP|P49965|SR72_SCHMA Signal recognition particle 72 kDa protein (SRP72). [Blood fluke] {Schistosoma mansoni}, complete
5601	ggaaaagatc	2				no_annot
5602	gtaattgtag	2	AF129816|Sma.747	95	0	Retinoid-x-receptor (RXR)
5603	aatctggtca	2	TC7484	80	0	weakly similar to SP|P50990|TCPQ_HUMAN T-complex protein 1, theta subunit (TCP-1-theta) (CCT-theta). [Human] {Homo sapiens}, partial (55%)
5604	gtacttagcg	2				no_annot
5605	tatccatttg	2				no_annot
5606	ataagtaata	2				no_annot
5607	tttacttgta	2				no_annot
5608	atctgactcc	2				no_annot
5609	taaaagaagt	2	TC14770	35	2	
5610	tgccaatgaa	2				no_annot
5611	tctgtaaaca	2				no_annot
5612	taatggggat	2	TC10386	60	3	similar to GP|20177041|gb|AAM12282.1||AY095189 LD26817p {Drosophila melanogaster}, partial (30%)
5613	caaatagaaa	2				no_annot
5614	gtatgttctt	2				no_annot
5615	aggagatcgt	2				no_annot
5616	tttgcaataa	2				no_annot
5617	gcaaccaatg	2				no_annot
5618	atgctcgaag	2				no_annot
5619	ccgatcatcg	2	AA999403	19	1	
5620	ggcagtcata	2				no_annot
5621	cctgtaaacc	2				no_annot

5622	attgaaaatg	2				no_annot
5623	cttgccaagg	2				no_annot
5624	gctcaagtca	2	TC16922	35	0	weakly similar to PIR|JC7601|JC7601 vesicular membrane-associated Dsr-1B protein - rat, partial (42%)
5625	taagtgtttc	2				no_annot
5626	tatgcgcaat	2	TC16038	81	0	
5627	gataaataaa	2	TC8605	91	0	
5627	gataaataaa	2	CD185889	21	1	weakly similar to SP|Q16974|KPC1_APLCA Calcium-dependent protein kinase C(APL I). [California sea hare] {Aplysia californica}, partial (12%)
5628	tacataagtc	2				no_annot
5629	catatatata	2	TC13898	99	0	similar to SP|Q9Y297|FW1A_HUMAN F-box/WD-repeat protein 1A (F-box and WD-repeats protein beta-TrCP) (E3RSIkappaB) (pIkappaBalpha-E3 receptor subunit). [Human] {Homo sapiens}, partial (50%)
5629	catatatata	2	TC18018	57	0	
5629	catatatata	2	TC18221	7	3	
5629	catatatata	2	CD129703	42	0	
5630	aattaccttt	2	TC15675	74	0	
5631	attgtagcgt	2				no_annot
5632	ttcgaattgg	2	CD082171	64	0	
5633	cctgtaaaaa	2	TC11919	86	1	
5633	cctgtaaaaa	2	TC19025	35	1	
5634	tatcacgaca	2				no_annot
5635	gctcagaacc	2				no_annot
5636	tgagcgacct	2				no_annot
5637	cgactgatct	2				no_annot
5638	aagggtagca	2	TC14481	87	0	
5639	agtggacaaa	2				no_annot
5640	actgtgatac	2				no_annot
5641	atgataatct	2				no_annot
5642	tgtattctat	2				no_annot

5643	catagttgca	2				no_annot
5644	caacaaagat	2				no_annot
5645	cctaattgta	2				no_annot
5646	atacgccaat	2	CD145737	91	0	
5646	atacgccaat	2	CD081872	61	0	homologue to PIR|A86527|A865 neutral amino acid (glutamate) transporter [imported] - Chlamydophila pneumoniae (strain J138), partial (3%)
5647	aagattacga	2	CD157721	31	2	
5648	tttgttttag	2	CD111359	46	1	
5649	tgacttgttg	2				no_annot
5650	ttggtggtcc	2	TC9760	24	0	
5651	attttgcttg	2	TC7725	44	3	
5652	taaacaataa	2	TC19760	37	1	
5653	attttgctta	2	TC12949	7	1	
5653	attttgctta	2	TC13468	76	3	similar to GP|17945787|gb|AAL48941.1||AY071319 RE34144p {Drosophila melanogaster}, partial (98%)
5654	tgattcggac	2	TC10492	93	0	weakly similar to SP|P51991|ROA3_HUMAN Heterogeneous nuclear ribonucleoprotein A3 (hnRNP A3) (D10S102). [Human] {Homo sapiens}, partial (11%)
5655	actgttgtta	2				no_annot
5656	tgcgttgtga	2	TC17296	69	1	
5657	gttacaatct	2				no_annot
5658	ggaaggaatc	2	C207779.1	81	0	
5658	ggaaggaatc	2	C608588.1	80	0	
5659	ataatcattt	2	CD067067	55	0	
5660	ttcaattggt	2				no_annot
5661	ttacaaatcc	2	TC15918	73	0	weakly similar to GP|15808053|gb|AAB57817.2||U96705 R86 {Mus musculus}, partial (6%)
5661	ttacaaatcc	2	CD187939	61	2	
5662	atcttgatac	2	TC16958	80	1	
5663	tgattctacc	2				no_annot
5664	gatgttggtg	2				no_annot
5665	tttatcgatg	2				no_annot

5666	gatgttggta	2				no_annot
5667	gctcccaagg	2	TC13660	5	1	weakly similar to GP|24266954|gb|AAN52376.1||AF548321 ribosomal protein L23a {Branchiostoma belcheri}, partial (69%)
5668	cagttgaaaa	2				no_annot
5669	gttcagcagt	2				no_annot
5670	tacaaggtcc	2	C202161.1	29	5	
5670	tacaaggtcc	2	C608299.1	22	5	
5671	catattgcta	2				no_annot
5672	tggtgcttca	2				no_annot
5673	tttctggaag	2	TC11367	29	1	
5674	tgtgcaagta	2	TC10957	65	2	weakly similar to GP|21428552|gb|AAM49936.1||AY118567 LD40170p {Drosophila melanogaster}, partial (4%)
5675	tgagatatca	2				no_annot
5676	tatcagtacc	2	C200532.1	46	0	
5676	tatcagtacc	2	C300058.1	55	0	
5676	tatcagtacc	2	C608016.1	52	0	
5676	tatcagtacc	2	C610098.1	13	7	
5677	gagtcctaaa	2	TC11148	40	1	weakly similar to GP|7415938|dbj|BAA93617.1||AB026688 asc1 {Mus musculus}, partial (10%)
5677	gagtcctaaa	2	TC18441	17	1	
5678	ttgaaatgtt	2				no_annot
5679	ccagttttaa	2	C315977.1	76	0	
5680	attgtcagcg	2				no_annot
5681	taaccactga	2	TC7118	47	6	weakly similar to GP|28274854|gb|AAO25692.1||AY195856 ankyrin repeat protein E4_8 {synthetic construct}, partial (28%)
5682	gaagtccact	2				no_annot
5683	cttctctctg	2				no_annot
5684	taaagcgtga	2				no_annot
5685	gatacgcaca	2				no_annot
5686	gatttattga	2				no_annot

5687	taagttgttc	2	C715566.1	82	0	
5688	ggaatccgtc	2				no_annot
5689	gaatctattc	2	CD060423	93	0	
5690	tgtaattcga	2				no_annot
5691	aagattacac	2	CD131268	96	0	
5692	taattgtttg	2	CD183226	56	1	weakly similar to GP|7292043|gb|AAF47456.1||AE003471 CG9194-PA {Drosophila melanogaster}, partial (4%)
5693	tgatcatata	2				no_annot
5694	atgacgacta	2	TC13460	72	0	
5694	atgacgacta	2	TC13489	12	0	
5694	atgacgacta	2	CD089019	6	1	SP|P54674|P3K Phosphatidylinositol 3-kinase 2 (EC 2.7.1.137) (PI3-kinase) (PtdIns-3-kinase) (PI3K). [Slime mold], partial (0%)
5695	taatttcgaa	2				no_annot
5696	ccttcggtgc	2				no_annot
5697	gtcaacaacc	2	TC17168	81	1	similar to GP|14919411|gb|AAH05728.1||BC005728 microfibrillar-associated protein 1 {Mus musculus}, partial (47%)
5698	gcggaatctg	2				no_annot
5699	acgtagctgt	2				no_annot
5700	gacaaatgat	2				no_annot
5701	atagttggga	2	TC9158	75	1	
5702	aactacttat	2				no_annot
5703	tgtcgtggca	2				no_annot
5704	agcatttgta	2				no_annot
5705	cttagacact	2	C200184.1	53	0	
5706	gttttgtttg	2				no_annot
5707	ttaaaagctt	2	TC11120	98	0	weakly similar to SP|O15173|PGC2_HUMAN Membrane associated progesterone receptor component 2 (Progesterone membrane binding protein) (Steroid receptor protein DG6). [Human] {Homo sapiens}, partial (30%)
5708	gacacactac	2	TC10911	90	0	similar to GP|17946020|gb|AAL49053.1||AY071431 RE52028p {Drosophila melanogaster}, partial (92%)

5709	ttttagtacc	2				no_annot
5710	ctcatcaatc	2				no_annot
5711	atagtttagt	2				no_annot
5712	tcgattatcc	2				no_annot
5713	ttatttctct	2				no_annot
5714	atatttaagc	2	C209911.1	58	0	
5715	aaatcatcaa	2	TC13887	16	5	similar to GP|1794137|dbj|BAA19098.1||AB000402 DMO25 {Drosophila melanogaster}, partial (45%)
5716	gttttgatgg	2				no_annot
5717	tcgattataa	2	CD076180	30	2	
5718	ggatgacagg	2				no_annot
5719	tcctcaactt	2				no_annot
5720	ttgaaaagac	2				no_annot
5721	attgatgctg	2	C208072.1	58	0	
5721	attgatgctg	2	C607063.1	58	0	
5722	cgatatatgc	2				no_annot
5723	ctgttagctg	2	C201140.1	43	0	
5723	ctgttagctg	2	C609770.1	42	0	
5724	aagctagttg	2				no_annot
5725	gtaaaatgac	2	TC16680	56	3	similar to GP|23664248|gb|AAN39278.1|AF519182_1|AF519182 eggshell precursor protein {Schistosoma japonicum}, partial (67%)
5726	gtaactgact	2				no_annot
5727	aatatgttca	2	CD091205	88	0	
5727	aatatgttca	2	CD160101	26	3	
5728	gtagtgctcg	2				no_annot
5729	cagcgttcca	2	TC11486	61	0	weakly similar to GP|17946032|gb|AAL49059.1||AY071437 RE52151p {Drosophila melanogaster}, partial (7%)
5730	atatatatgt	2	TC10843	69	2	similar to GP|2217962|emb|CAA64439.1||X94983 GDP-dissociation inhibitor {Geodia cydonium}, partial (67%)
5730	atatatatgt	2	TC15462	27	0	

5731	tgccgaccaa	2				no_annot
5732	aaaacggagt	2	C209221.1	6	2	
5732	aaaacggagt	2	C605498.1	6	2	
5733	caacattcga	2				no_annot
5734	aagttatatt	2	TC13864	80	1	similar to PIR|D88678|D88678 protein H06H21.3 [imported] - Caenorhabditis elegans, partial (59%)
5735	gaccatccag	2				no_annot
5736	gttcggctcc	2				no_annot
5737	ctttaactgg	2				no_annot
5738	attgtctgaa	2				no_annot
5739	cgtcacgatg	2	TC7045	65	0	GP|160925|gb|AAA29853.1||M94346 A.1.12/9 antigen {Schistosoma mansoni}, partial (5%)
5739	cgtcacgatg	2	TC7147	71	2	weakly similar to GP|21553851|gb|AAM62944.1||AY085726 40S ribosomal protein S2 {Arabidopsis thaliana}, partial (7%)
5739	cgtcacgatg	2	CD134136	63	0	
5739	cgtcacgatg	2	CD134151	43	0	
5739	cgtcacgatg	2	CD144227	62	0	
5739	cgtcacgatg	2	CD133474	12	1	
5739	cgtcacgatg	2	CD146471	55	0	
5740	aagaactgag	2	TC12681	92	0	similar to GP|26105953|gb|AAN78331.1||AC137988 TcC31.4 {Trypanosoma cruzi}, partial (8%)
5741	gcagtaacga	2				no_annot
5742	tgctcgaaga	2				no_annot
5743	tacattacga	2				no_annot
5744	aatggttctg	2				no_annot
5745	cggttgaata	2				no_annot
5746	agcagtaatg	2	CD081410	46	0	
5747	tatattgcaa	2				no_annot
5748	atgacatatc	2	CD140886	39	0	weakly similar to GP|20977577|gb|AAM28216.1||AY099528 lysosome membrane protein II {Danio rerio}, partial (13%)
5749	gctgagaatc	2	CD089726	42	0	

5749	gctgagaatc	2	CD092684	27	2	similar to GP|19067879|gb|AAK14815.1||AY027869 polyprotein {Schistosoma japonicum}, partial (6%)
5750	tttcactgtg	2				no_annot
5751	ttatcccaat	2				no_annot
5752	tggaaacatc	2	TC10471	52	9	similar to PIR|S05988|S05988 translation elongation factor eEF-2 - fruit fly (Drosophila melanogaster), partial (90%)
5753	acgtctcttt	2				no_annot
5754	gattacttat	2				no_annot
5755	tgtttttgtt	2	TC13916	9	0	
5756	ttgtaaaatt	2				no_annot
5757	aataaaagtg	2	C205920.1	89	0	
5757	aataaaagtg	2	C605798.1	89	0	
5758	ggtatttgtt	2				no_annot
5759	catcatcatc	2				no_annot
5760	cgtcggattt	2				no_annot
5761	tctttcccga	2				no_annot
5762	gaaagtcaga	2				no_annot
5763	tggacaaaaa	2	TC7937	46	0	similar to GP|1840087|gb|AAB47253.1||U83280 39 kDa antigen {Leishmania donovani}, partial (5%)
5764	tgtgtttggt	2	TC14166	14	1	
5765	aacaagtatg	2	TC11197	69	0	
5766	aacaagtatc	2	CD076203	41	0	
5767	ccaattgatc	2				no_annot
5768	gaataatcaa	2	C300792.1	43	1	
5768	gaataatcaa	2	C708173.1	43	1	
5769	ggcacgaaag	2	TC7643	62	1	weakly similar to GP|15213764|gb|AAK92157.1|AF400185_1|AF400185 ribosomal protein L14 {Spodoptera frugiperda}, partial (36%)
5770	ctctccagat	2	TC13525	23	1	
5771	aattcacagc	2	C603031.1	74	0	
5772	tacaaacctt	2				no_annot

5773	atatggaaac	2	TC12990	36	0	
5773	atatggaaac	2	TC17516	64	2	weakly similar to GP|18478512|gb|AAL73190.1|AF334609_1|AF334609 IMP4 {Mus musculus}, partial (49%)
5774	tagcaaggtc	2	TC10491	58	0	
5775	cagctcagga	2				no_annot
5776	cataactaag	2				no_annot
5777	tgtccatttt	2	C306966.1	6	0	
5778	atcgaaagag	2	C208623.1	15	7	
5778	atcgaaagag	2	C605473.1	11	8	
5779	ttgactgaaa	2				no_annot
5780	agcctgatgg	2	U54586|Sma.317	42	0	Pro-His-rich protein
5781	gtcatacgca	2	TC14388	83	0	weakly similar to PIR|A72230|A72230 endopeptidase La- Thermotoga maritima (strain MSB8), partial (3%)
5782	gcctgctgat	2				no_annot
5783	tgaatcttac	2				no_annot
5784	acaactcaga	2				no_annot
5785	gattctgctg	2				no_annot
5786	gcacgttgtc	2	TC13219	26	0	similar to EGAD|93314|101313 thrombospondin 2 precursor {Bos taurus}, partial (4%)
5786	gcacgttgtc	2	BG931373	83	0	similar to GP|11320818|dbj VSGP/F-spondin {Bos taurus}, partial (1%)
5787	acggattgat	2				no_annot
5788	tatgagaagt	2	TC11694	67	0	weakly similar to SP|Q9H9B4|SFX1_HUMAN Sideroflexin 1. [Human] {Homo sapiens}, partial (68%)
5789	aactaacaat	2				no_annot
5790	acattttgtc	2				no_annot
5791	tccctattag	2				no_annot
5792	tagactccaa	2	C209090.1	58	1	
5792	tagactccaa	2	C602028.1	53	1	
5793	ttcaacatca	2	TC10782	13	5	
5794	cgaattatgt	2	C603196.1	39	0	
5794	cgaattatgt	2	C610058.1	43	4	

5795	gaggaggtcg	2				no_annot
5796	acataatagt	2				no_annot
5797	gttagatcgt	2				no_annot
5798	tcgaaactat	2	TC7894	80	1	weakly similar to GP|1196428|gb|AAA88032.1||M14123 neutral protease large subunit {Homo sapiens}, partial (27%)
5799	gaaaaaagta	2				no_annot
5800	taggctttca	2				no_annot
5801	tactctcacc	2	C317805.1	92	0	
5801	tactctcacc	2	C603016.1	77	4	
5802	aatccaagca	2				no_annot
5803	tcctcaacaa	2				no_annot
5804	tatagagaaa	2	TC7794	72	4	weakly similar to GP|7291735|gb|AAF47157.1||AE003462 CG3209-PA {Drosophila melanogaster}, partial (21%)
5805	caagaatgta	2				no_annot
5806	ttctcttgtc	2				no_annot
5807	tgcagtctca	2				no_annot
5808	gttacttaga	2	C206140.1	14	1	
5808	gttacttaga	2	C601920.1	17	1	
5809	gggtcgagaa	2	TC14236	72	1	weakly similar to PIR|T30855|T30855 multidrug resistance protein 2 - fluke (Schistosoma mansoni), partial (15%)
5810	ttcattggtg	2				no_annot
5811	ctaattacaa	2				no_annot
5812	tttaaaacga	2				no_annot
5813	ataacattta	2	C200002.1	58	0	
5813	ataacattta	2	C208116.1	26	1	
5813	ataacattta	2	C612096.1	26	1	
5814	tatagtatga	2	AY663841|Sma.5380	69	0	HR96-a (HR96-a)
5815	ctaaacagac	2				no_annot
5816	agtgtccaaa	2				no_annot
5817	acgaaaaaaa	2				no_annot

5818	gtgctttagg	2				no_annot
5819	aaaagcaacc	2				no_annot
5820	ataaaagtat	2	TC14653	76	0	similar to GP|13660779|gb|AAK33013.1||AY029526 brain-enriched WD-repeat protein {Rattus norvegicus}, partial (21%)
5821	gattgtgatt	2	C209641.1	36	0	
5821	gattgtgatt	2	C610520.1	38	0	
5822	taatttgtac	2				no_annot
5823	gagagcacca	2				no_annot
5824	gctagagaaa	2	TC15343	37	0	similar to GP|16768172|gb|AAL28305.1||AY060757 GH20858p {Drosophila melanogaster}, partial (46%)
5825	cagcaaaacg	2				no_annot
5826	atgatttaaa	2	CD078855	42	4	similar to GP|16769418|gb|AAL28928.1||AY061380 LD30319p {Drosophila melanogaster}, partial (3%)
5826	atgatttaaa	2	CD198005	12	0	weakly similar to PIR|D96538|D96538 cytosolic tRNA-Ala synthetase [imported] - Arabidopsis thaliana, partial (9%)
5827	tgtattgctt	2				no_annot
5828	ttaccaccgt	2				no_annot
5829	ataattatcg	2				no_annot
5830	tgtgaacgcc	2				no_annot
5831	cacaccctct	2				no_annot
5832	caacgttggt	2	U30175|Sma.742	44	2	Enolase trans-spliced
5833	ttggcaaggt	2				no_annot
5834	cgagtaactg	2				no_annot
5835	taatgggtgt	2	TC9086	60	1	
5836	tatttttttt	2	TC17529	90	0	weakly similar to GP|17862592|gb|AAL39773.1||AY069628 LD39967p {Drosophila melanogaster}, partial (17%)
5836	tatttttttt	2	AW017417	8	1	
5837	atttcaagag	2	TC15262	21	2	
5838	cgacagcaga	2				no_annot
5839	tttcattgtt	2				no_annot
5840	tattttttg	2				no_annot

5841	tatcatcaat	2				no_annot
5842	cgtataaaac	2				no_annot
5843	ggaacacgga	2				no_annot
5844	catagtatct	2				no_annot
5845	atgtagatgt	2				no_annot
5846	ataacaatca	2	TC8882	25	4	similar to GP|14193302|gb|AAK55887.1|AF267210_2|AF267210 ATP synthase gamma subunit {Candidatus Carsonella ruddii}, partial (8%)
5847	acagtcattt	2	TC17384	99	0	similar to GP|11992277|gb|AAG42496.1|AF323918_1|AF323918 NUDE-like protein {Mus musculus}, partial (32%)
5848	tatgttttgg	2				no_annot
5849	tcacattgtc	2				no_annot
5850	aaaaatgcag	2				no_annot
5851	tgttgcggct	2				no_annot
5852	cataatggag	2				no_annot
5853	aaattgtgta	2				no_annot
5854	agttaaacaa	2	C609744.1	97	0	
5855	tatgcgtgtg	2	TC11403	41	0	
5855	tatgcgtgtg	2	CD124669	61	0	
5856	tgctttctat	2				no_annot
5857	ttaagcgcct	2	TC7753	21	1	weakly similar to GP|19570497|dbj|BAB86334.1||AB057596 VANGL1 {Homo sapiens}, partial (9%)
5858	tccccgacat	2				no_annot
5859	taatccgact	2				no_annot
5860	ctcaacttcc	2				no_annot
5861	ttgtggagtg	2				no_annot
5862	cgaagcgtgg	2				no_annot
5863	tagtttcacc	2	CD178846	17	1	
5864	ttgttctgct	2				no_annot
5865	ctgtaaaaat	2				no_annot

5866	attggtaact	2	TC14168	90	0	similar to PIR|T39903|T39903 serine-rich protein - fission yeast (Schizosaccharomyces pombe), partial (6%)
5867	tgtaagtgtt	2				no_annot
5868	agcgatttgc	2	TC11219	51	1	
5868	agcgatttgc	2	CD193276	69	0	
5869	acggttaatt	2	TC17656	89	0	weakly similar to GP|17946324|gb|AAL49202.1||AY071580 RE63629p {Drosophila melanogaster}, partial (7%)
5870	aaaggcgata	2				no_annot
5871	atttgattaa	2	TC14680	7	5	similar to SP|Q9UBS0|K6B2_HUMAN Ribosomal protein S6 kinase beta 2(S6K-beta 2) (70 kDa ribosomal protein S6 kinase 2) (p70-S6KB) (p70 ribosomal S6 kinase beta) (p70 S6Kbeta) (S6K2) (S6 kinase-related kinase) (SRK) (Serine/threonine-protein kinase 14 beta). [Human] {Homo sapiens}, partial (5%)
5872	ttctgtttcg	2				no_annot
5873	ttgttctgat	2				no_annot
5874	tcaagaagta	2				no_annot
5875	ctaccaccaa	2				no_annot
5876	tgaccactac	2				no_annot
5877	tatacaccaa	2				no_annot
5878	atgttgtaga	2	CD196758	79	0	
5879	taatgcagta	2				no_annot
5880	accgatgaaa	2	C301359.1	95	0	
5880	accgatgaaa	2	C718285.1	49	0	
5881	gtttctgatt	2				no_annot
5882	ttggtttggc	2				no_annot
5883	tacataattt	2	C208109.1	94	0	
5883	tacataattt	2	C210021.1	90	1	
5884	agcagttact	2				no_annot
5885	aaactaacta	2				no_annot
5886	tgacctgaaa	2	CD076827	8	0	homologue to PIR|C86287|C862 F9L1.24 protein - Arabidopsis thaliana, partial (1%)
5887	acttattctt	2	TC11191	37	2	

5887	acttattctt	2	TC17330	74	0	weakly similar to GP|7248365|dbj|BAA92700.1||AB039962 proliferating cell nuclear antigen subtype1 {Xenopus laevis}, partial (98%)
5888	ctgacggttg	2				no_annot
5889	tatctctctt	2	CD155004	35	0	
5890	atccagagaa	2	C606775.1	31	2	
5890	atccagagaa	2	C606775.1	31	1	
5891	tcttgtttgt	2				no_annot
5892	gcactaaaaa	2				no_annot
5893	gcgtgaagat	2	TC13468	45	5	similar to GP|17945787|gb|AAL48941.1||AY071319 RE34144p {Drosophila melanogaster}, partial (98%)
5894	agctccgcac	2	TC17371	81	0	similar to GP|7296251|gb|AAF51542.1||AE003590 CG11454-PA {Drosophila melanogaster}, partial (6%)
5895	caacactatt	2				no_annot
5896	tattacgaat	2				no_annot
5897	tattggatga	2	TC10965	41	2	similar to GP|21392208|gb|AAM48458.1||AY118429 RH26504p {Drosophila melanogaster}, partial (36%)
5898	gtgttatttg	2				no_annot
5899	gctacaccaa	2	TC8630	63	3	weakly similar to GP|6980000|gb|AAF34689.1|AF222864_1|AF222864 Dspt5 {Drosophila melanogaster}, partial (4%)
5900	acagcttcac	2				no_annot
5901	taaatggaca	2				no_annot
5902	gaaaactccg	2	CD168328	59	0	homologue to GP|15458837|gb| ABC transporter ATP-binding/membrane spanning protein - multidrug resistance, partial (2%)
5903	gtatgattca	2				no_annot
5904	acaatgtcaa	2				no_annot
5905	aaccattcca	2	U30663|Sma.20	71	1	Sm24
5906	ttatgcattt	2	TC11382	45	3	
5907	aaacaggatg	2				no_annot
5908	ggaatcgtcg	2				no_annot
5909	aagatattgt	2	TC17974	85	0	similar to GP|22946910|gb|AAN11076.1||AE003666 CG31683-PA {Drosophila melanogaster}, partial (7%)

5910	ttgtaataat	2	TC9751	86	0	
5910	ttgtaataat	2	TC11926	88	0	weakly similar to GP|18043915|gb|AAH19374.1||BC019374 glutamate-cysteine ligase, catalytic subunit {Mus musculus}, partial (12%)
5911	gagctaatat	2				no_annot
5912	gtgttaatgc	2	C201598.1	16	3	
5913	aaacttgtta	2				no_annot
5914	ctgccaatga	2				no_annot
5915	ttggcgcaat	2	TC14772	35	5	
5916	ctcagcgagg	2				no_annot
5917	tatccgttta	2				no_annot
5918	ccagttacta	2				no_annot
5919	tgcgaaattt	2	C609435.1	27	6	
5920	ataaatgagt	2	CD183868	30	3	
5921	gataaccata	2	C607402.1	17	6	
5922	ttactgaagt	2				no_annot
5923	gaaaaatgga	2				no_annot
5924	ttagaattag	2				no_annot
5925	gatacattgg	2				no_annot
5926	aaaagtaaat	2				no_annot
5927	aggaggtcga	2				no_annot
5928	gtatgtggta	2				no_annot
5929	tatctcacat	2	TC17097	75	1	weakly similar to PIR|S66288|S66288 nuclear pore-targeting complex protein, 97K - mouse, partial (51%)
5929	tatctcacat	2	TC19392	18	3	
5930	ttgaagagtc	2	TC7218	39	0	weakly similar to GP|7293971|gb|AAF49330.1||AE003523 CG7564-PA {Drosophila melanogaster}, partial (43%)
5931	gttctataca	2				no_annot
5932	cctgtgaact	2				no_annot
5933	gcaatggaat	2				no_annot

5934	accaacgtga	2				no_annot
5935	tgcttgactt	2	C608661.1	43	0	
5936	tacccggctc	2				no_annot
5937	tacatatttt	2				no_annot
5938	tatatgaagg	2				no_annot
5939	tacatatttg	2	TC17762	95	0	
5940	gccaattgat	2	TC10249	93	0	
5941	cctgagatgc	2				no_annot
5942	tccaaccctc	2				no_annot
5943	cttattcaga	2				no_annot
5944	tgaacgtgaa	2	TC18483	75	1	
5945	acttgcagcg	2				no_annot
5946	gaaattctag	2				no_annot
5947	taatcactct	2				no_annot
5948	tctattggac	2	TC12685	28	1	
5949	tcccggtaca	2				no_annot
5950	gcttccaaaa	2	AI394831	72	0	
5951	ttgccgaggc	2				no_annot
5952	tatgatcatc	2				no_annot
5953	tatggaacag	2	TC8382	54	0	
5954	tatatagtat	2				no_annot
5955	gataaactgc	2				no_annot
5956	tacggcttct	2				no_annot
5957	tgcaacagac	2	C210732.1	24	1	
5958	cttactttct	2				no_annot
5959	taaacgatca	2	AI559078	9	1	
5960	aatgtttgtc	2	TC10556	38	5	RTNL [Schistosoma mansoni]
5961	atatcgaatt	2				no_annot
5962	ggttcaaaag	2	TC10689	23	0	similar to GP|29409366|gb|AAM29179.1||AY093954 prohibitin protein Wph {Triticum aestivum}, partial (90%)

5963	catttacaat	2	TC7679	45	8	weakly similar to GP|28630303|gb|AAM92833.1||AY130428 protein kinase C {Branchiostoma lanceolatum}, partial (7%)
5963	catttacaat	2	CD064334	9	0	
5964	ttggcttcct	2	CD121906	94	0	
5965	atgatgatat	2	TC17301	81	0	weakly similar to GP|21645541|gb|AAM71069.1||AE003833 CG1884-PA {Drosophila melanogaster}, partial (6%)
5965	atgatgatat	2	CD116769	4	2	
5966	tgttttattt	2	TC7621	88	0	similar to GP|16741554|gb|AAH16585.1||BC016585 UDP-N-acetyl-alpha-D-galactosamine:polypeptide N-acetylgalactosaminyltransferase 9 {Mus musculus}, partial (40%)
5966	tgttttattt	2	TC11388	98	0	weakly similar to PIR|T39903|T39903 serine-rich protein - fission yeast (Schizosaccharomyces pombe), partial (9%)
5966	tgttttattt	2	CD145567	7	0	
5966	tgttttattt	2	CD129445	84	0	GP|161061|gb|AAA29916.1||M17423 protease {Schistosoma mansoni}, partial (8%)
5967	aaagtccgat	2				no_annot
5968	tactagtggc	2				no_annot
5969	ttgaacaagc	2				no_annot
5970	cacgtacgat	2				no_annot
5971	ttctcagatg	2	TC14538	92	0	
5972	tacataatat	2	CD154748	65	3	
5973	tcatagcttg	2	CD080587	45	0	similar to SP|P15711|104K_ 104 kDa microneme-rhoptry antigen. {Theileria parva}, partial (1%)
5974	tatctttctg	2	TC12326	21	2	GP|8698893|gb|AAF78512.1|AF195210_1|AF195210 photolyase {Pyrus pyrifolia}, partial (14%)
5975	caaccagcca	2	TC9359	16	0	
5976	ttagcgctag	2				no_annot
5977	acttattcaa	2				no_annot
5978	actattacgg	2				no_annot
5979	tattatgaat	2	TC7608	91	0	
5980	cacataatac	2	AW146473	34	0	

5981	aatttctggg	2				no_annot
5982	cgacaaattt	2				no_annot
5983	aaaagccgac	2				no_annot
5984	attaagtgag	2	C600634.1	51	0	
5985	tggataacgt	2	TC16705	64	3	
5986	atcagatatg	2				no_annot
5987	ttgcctggtt	2	CD180276	47	0	
5988	ttttctgttc	2	C210916.1	54	2	
5988	ttttctgttc	2	C602591.1	54	2	
5989	tgtgttatcg	2	C209456.1	86	0	
5989	tgtgttatcg	2	C612231.1	85	0	
5990	cagcaacgaa	2	TC7692	38	0	weakly similar to GP|7573635|dbj|BAA94544.1||AB039933 polyposis locus protein 1-like 1 (TB2 protein-like 1) {Mus musculus}, partial (24%)
5991	gcgattctct	2				no_annot
5992	atcatttcgt	2	TC13469	96	0	
5993	agtaacacaa	2	TC17620	34	2	
5994	tacatattga	2				no_annot
5995	attctggatc	2				no_annot
5996	accttggatg	2				no_annot
5997	tgctacaata	2	M27529|Sma.142	85	1	Superoxide dismutase
5998	gaagaatagt	2				no_annot
5999	ctatgtattg	2				no_annot
6000	atgcgcaatt	2	TC8088	77	1	weakly similar to GP|23171634|gb|AAF55546.2||AE003721 CG7675-PB {Drosophila melanogaster}, partial (8%)
6001	taaatgcact	2				no_annot
6002	tacgatcgtt	2	C201206.1	13	3	
6002	tacgatcgtt	2	C601217.1	28	3	
6003	atagctgatg	2				no_annot
6004	atatcgtatt	2				no_annot

6005	acgagttagc	2				no_annot
6006	tatgatcagg	2				no_annot
6007	tcactgtgtg	2				no_annot
6008	cacatcaaat	2				no_annot
6009	gaggtaattg	2	TC19469	46	2	GP|9843775|emb|CAC03718.1||AJ272345 PS1D protein {Mus musculus}, partial (6%)
6010	aatggtaata	2	C608362.1	75	2	
6011	catctatgct	2	TC12248	40	0	weakly similar to SP|P40692|MLH1_HUMAN DNA mismatch repair protein Mlh1 (MutL protein homolog 1). [Human] {Homo sapiens}, partial (6%)
6012	acaaatgggt	2				no_annot
6013	tgtactaggc	2				no_annot
6014	tttctattgc	2	TC14039	17	2	weakly similar to GP|21483368|gb|AAM52659.1||AY122147 LD02379p {Drosophila melanogaster}, partial (10%)
6015	gattgggttc	2	CD063619	73	0	weakly similar to GP|21617886|gb|AAM66936.1||AY088613 40S ribosomal protein S5 {Arabidopsis thaliana}, partial (13%)
6016	ttattacgga	2				no_annot
6017	cccctgtttg	2				no_annot
6018	ttgtgtaaac	2				no_annot
6019	aattccacca	2	TC7488	28	2	
6020	agaagattct	2	TC16873	7	7	
6020	agaagattct	2	BF936149	42	2	weakly similar to GP|19067879|gb|AAK14815.1||AY027869 polyprotein {Schistosoma japonicum}, partial (3%)
6021	taagtatatt	2	TC17273	89	0	weakly similar to GP|7109287|gb|AAF36718.1|AF227508_1|AF227508 intestinal alkaline phosphatase-II {Rattus norvegicus}, partial (13%)
6022	ctcttactgc	2				no_annot
6023	gaaggaatat	2				no_annot
6024	cacacaaata	2	CD144168	73	1	
6025	cctggcactg	2	TC8521	48	0	

6026	aatgacccga	2	TC11150	48	0	weakly similar to SP|O70593|SGT_RAT Small glutamine-rich tetratricopeptide repeat-containing protein. [Rat] {Rattus norvegicus}, partial (37%)
6027	taaatatgat	2				no_annot
6028	attgcaatca	2	TC13967	76	0	
6029	tacaacatag	2				no_annot
6030	taaattacaa	2				no_annot
6031	cgacgttgct	2				no_annot
6032	tacagagttg	2				no_annot
6033	cctttgggaa	2				no_annot
6034	aagaggaacc	2				no_annot
6035	gttatatgcc	2				no_annot
6036	gcttctaaaa	2				no_annot
6037	tagagctgga	2				no_annot
6038	aaacctgatg	2				no_annot
6039	tatgatcaat	2	TC18304	66	2	
6039	tatgatcaat	2	CD131623	92	0	
6040	ttactactcc	2	TC16948	58	8	similar to PIR|PC4365|PC4365 calcium-binding protein - tapeworm (Echinococcus granulosus) (fragment), partial (43%)
6041	tggacgaagt	2				no_annot
6042	aatatatata	2	TC7980	95	0	homologue to GP|3372810|gb|AAC28342.1||AF064492 LIM homeobox protein cofactor {Homo sapiens}, partial (9%)
6042	aatatatata	2	TC9236	16	3	similar to GP|1684843|gb|AAB48302.1||U77717 pinin {Bos taurus}, partial (4%)
6042	aatatatata	2	TC10142	81	0	similar to GP|1785638|emb|CAA71235.1||Y10151 CRF2 receptor, beta isoform {Homo sapiens}, partial (20%)
6042	aatatatata	2	TC11563	60	1	similar to GP|21430868|gb|AAM51112.1||AY119252 SD21818p {Drosophila melanogaster}, partial (11%)
6042	aatatatata	2	TC19038	48	4	
6042	aatatatata	2	CD196655	32	2	
6043	taaccttgta	2	TC12205	10	2	similar to PIR|S59634|S59634 endo-1,4-beta-xylanaseF precursor - Pseudomonas fluorescens, partial (4%)

6044	aattgaatac	2	TC10352	12	2	weakly similar to SP|P53832|WSC2_YEAST Cell wall integrity and stress response component 2 precursor. [Baker's yeast] {Saccharomyces cerevisiae}, partial (6%)
6044	aattgaatac	2	TC13867	77	0	weakly similar to GP|23093631|gb|AAN11878.1||AE003544 CG6084-PB {Drosophila melanogaster}, partial (37%)
6045	attagtatat	2	TC7368	44	1	weakly similar to GP|10440990|gb|AAG16892.1|AF173680_1|AF173680 myosin regulatory light chain {Riftia pachyptila}, partial (80%)
6046	cgtttacata	2	TC12120	14	0	
6047	ttgaactaat	2	TC15996	24	2	
6048	gacgggatga	2	TC11339	79	0	
6049	ttaatattct	2	TC16316	82	0	
6049	ttaatattct	2	CD069199	31	2	
6050	ttgattcctt	2				no_annot
6051	ttcgctatac	2				no_annot
6052	tacatttatt	2	TC10774	2	7	
6052	tacatttatt	2	TC10774	2	2	
6053	gaacagtatc	2				no_annot
6054	aaattgattg	2				no_annot
6055	tacatttatg	2				no_annot
6056	ggatgatgat	2				no_annot
6057	tagtcaagtt	2	CD059843	0	0	
6058	gtattggccg	2				no_annot
6059	tcaagttcct	2				no_annot
6060	actcaccgaa	2				no_annot
6061	gatcctcaaa	2				no_annot
6062	attatagttg	2				no_annot
6063	atcaaaccca	2				no_annot
6064	aaccaaatat	2	TC12203	37	2	
6065	tgcttatagc	2				no_annot
6066	ggcaaaatat	2	TC17539	81	0	

6067	ttggaagtga	2				no_annot
6068	cttcttggtt	2	TC11281	11	2	
6069	cattgatcat	2	TC17839	82	1	
6069	cattgatcat	2	CD139164	67	0	
6070	attgttttcc	2				no_annot
6071	ttggtttttc	2	TC10741	57	0	weakly similar to GP|2358262|gb|AAB69115.1||U89514 calpain large subunit {Rattus norvegicus}, partial (23%)
6072	cgcttccctc	2				no_annot
6073	ttcttccaag	2	C606688.1	56	0	
6074	cacttagttt	2				no_annot
6075	agagatatgc	2	C201150.1	2	20	
6075	agagatatgc	2	C608679.1	2	20	
6076	acgtcgtcaa	2	C717352.1	37	1	
6077	aagtagatga	2				no_annot
6078	aatcttttgc	2				no_annot
6079	caattgtctg	2	C611011.1	5	0	
6080	ttgcgatccc	2				no_annot
6081	aaaaggatgt	2				no_annot
6082	tcgcgttttt	2				no_annot
6083	ttgaataaat	2	TC12777	90	0	
6084	aaaacaataa	2	C610688.1	84	0	
6085	taaaattgta	2				no_annot
6086	gctatactac	2				no_annot
6087	tatgtctctc	2				no_annot
6088	agcaatgaac	2				no_annot
6089	ttgatacgat	2	C600899.1	61	1	
6090	gggcgacgac	2	TC7564	62	1	weakly similar to GP|5902663|gb|AAC13264.2||AF029844 elongation factor 1-beta homolog {Mus musculus}, partial (67%)
6091	tataacctga	2				no_annot
6092	gttccaccca	2				no_annot
6093	tgcttataac	2	TC11908	28	1	

6093	tgcttataac	2	CD189762	11	2	
6094	cgctcgcttt	2				no_annot
6095	tgttcattgt	2				no_annot
6096	aatcctaatc	2	AF071011|Sma.779	67	1	Myosin light chain
6097	tgaaaagctt	2	TC7644	64	0	similar to SP|Q16254|E2F4_HUMAN Transcription factor E2F4 (E2F-4). [Human] {Homo sapiens}, partial (30%)
6098	atcgttgcat	2				no_annot
6099	gattgagctg	2				no_annot
6100	ttaattaaga	2	TC8233	23	2	
6100	ttaattaaga	2	TC14931	88	0	similar to GP|22335385|dbj|BAC10416.1||AB079385 Apg7p {Mus musculus}, partial (17%)
6101	ttatatcgtt	2	CD068279	9	2	
6102	tttgtaatta	2	CD084069	75	1	GP|12054832|em PstA protein {Streptomyces griseus subsp. griseus}, partial (2%)
6103	gataatcaaa	2				no_annot
6104	ccttcggcac	2				no_annot
6105	tactggccca	2				no_annot
6106	agagcgtcga	2	C202138.1	22	1	
6107	gtgccagtca	2				no_annot
6108	ctaattaagc	2	C209289.1	29	4	
6108	ctaattaagc	2	C609001.1	27	5	
6109	cttccaaaga	2				no_annot
6110	cacaggtata	2				no_annot
6111	aacatcacac	2	C201692.1	32	0	
6111	aacatcacac	2	C608050.1	62	0	
6112	tagatgtctt	2				no_annot
6113	aatctgccag	2	BF936813	14	3	
6114	atccagatgt	2				no_annot
6115	catacacatc	2				no_annot

6116	tttccaaacc	2	TC14009	76	1	similar to PIR|AG0096|AG0096 phosphoenolpyruvate-protein phosphotransferase[imported] - Yersinia pestis (strain CO92), partial (3%)
6117	aattggtggt	2				no_annot
6118	tttcaacata	2				no_annot
6119	ctggagaaac	2				no_annot
6120	ccttcgcctt	2				no_annot
6121	atgttggtgc	2	TC7662	73	1	
6122	cttcttaaat	2				no_annot
6123	acaaacgttt	2	TC18594	72	0	
6124	tatgtatgtc	2				no_annot
6125	taattaaaaa	2				no_annot
6126	agaagttagc	2				no_annot
6127	gatgttctaa	2	C201602.1	10	4	
6128	actctgaagt	2	TC18856	45	0	
6129	gccgatcgag	2				no_annot
6130	ttacgtactt	2				no_annot
6131	tttgtcaata	2				no_annot
6132	acttacaaga	2				no_annot
6133	ttggagtaga	2				no_annot
6134	gctcaaccat	2				no_annot
6135	ctcgatctct	2				no_annot
6136	gtagtacaat	2	TC16520	22	0	
6137	gaatgattat	2	C608397.1	41	4	
6138	gagtgacggc	2	TC7143	90	2	similar to GP|16930529|gb|AAL31950.1||AF421549 CDH1-D {Gallus gallus}, partial (19%)
6138	gagtgacggc	2	BG931906	29	2	homologue to EGAD|131264|140075 hemolysin {Acanthamoeba polyphaga}, partial (11%)
6138	gagtgacggc	2	CD061927	42	0	
6138	gagtgacggc	2	BG932832	84	0	
6138	gagtgacggc	2	BG932902	54	0	

6138	gagtgacggc	2	BG931581	44	0	homologue to EGAD|131264|140075 hemolysin {Acanthamoeba polyphaga}, partial (11%)
6138	gagtgacggc	2	BG931709	47	0	homologue to EGAD|131264|140075 hemolysin {Acanthamoeba polyphaga}, partial (11%)
6138	gagtgacggc	2	BG931760	40	1	similar to EGAD|131264|140075 hemolysin {Acanthamoeba polyphaga}, partial (22%)
6138	gagtgacggc	2	BG932855	10	2	homologue to EGAD|131264|140075 hemolysin {Acanthamoeba polyphaga}, partial (11%)
6138	gagtgacggc	2	BG932872	34	2	homologue to EGAD|131264|140075 hemolysin {Acanthamoeba polyphaga}, partial (11%)
6138	gagtgacggc	2	BG932892	34	2	homologue to EGAD|131264|140075 hemolysin {Acanthamoeba polyphaga}, partial (11%)
6139	ggattctgta	2				no_annot
6140	aagctgctta	2	BG932332	71	0	GP|1401252|gb|AAB03395.1||U59509 mlrq-like protein {Mus musculus}, partial (80%)
6141	ttgactagct	2	N20792	70	0	
6142	gcagacaaaa	2				no_annot
6143	aatgaagttg	2				no_annot
6144	cataggcata	2				no_annot
6145	ttggaccata	2				no_annot
6146	gcatacggtg	2				no_annot
6147	gtgggtgaat	2				no_annot
6148	ctggtgccgg	2	TC14567	88	0	
6149	taatacaagg	2				no_annot
6150	attgatcata	2				no_annot
6151	ttcacgtata	2				no_annot
6152	ccggtgtcgc	2				no_annot
6153	tttgtatttg	2				no_annot
6154	ttgtcaatga	2				no_annot
6155	gatgattcgt	2	TC8806	92	0	weakly similar to GP|19699033|gb|AAL91140.1||AY081909 Smad nuclear-interacting protein 1 {Homo sapiens}, partial (33%)
6156	aggcatcttt	2	C612459.1	91	0	

6157	tacacataca	2	TC9599	24	2	similar to SP|P35659|DEK_HUMAN DEK protein. [Human] {Homo sapiens}, partial (5%)
6158	tttccgtcct	2	C300810.1	37	1	
6158	tttccgtcct	2	C606855.1	35	1	
6159	tgtattgata	2	CD160736	80	0	
6160	tattctcgct	2				no_annot
6161	taatccagtg	2				no_annot
6162	tcaccatagg	2	CD073219	94	0	similar to GP|8132322|gb|AAF73250.1|AF153977_1|AF153977 eukaryotic translation initiation factor 5 {Schistosoma mansoni}, partial (37%)
6162	tcaccatagg	2	CD184803	31	0	GP|8132322|gb|AAF73250.1|AF153977_1|AF153977 eukaryotic translation initiation factor 5 {Schistosoma mansoni}, partial (22%)
6163	aggaacttca	2	C209730.1	12	3	
6163	aggaacttca	2	C607916.1	16	3	
6164	acattacaag	2				no_annot
6165	tgctctagtg	2	TC9268	4	3	
6165	tgctctagtg	2	TC15693	49	0	
6165	tgctctagtg	2	TC16708	47	2	
6165	tgctctagtg	2	CD158289	41	0	
6166	gtaagacaaa	2				no_annot
6167	gtcaatctgc	2				no_annot
6168	tagcttttgg	2				no_annot
6169	gatacgtttt	2	TC12435	56	2	
6170	tttaaaaaat	2	CD080561	13	1	
6170	tttaaaaaat	2	CD192366	7	1	
6171	ggcgagtatt	2				no_annot
6172	cgttaactac	2	C603542.1	68	0	
6173	atcatctata	2	TC13816	76	0	similar to GP|25990819|gb|AAN76709.1|AF443827_1|AF443827 acheron {Manduca sexta}, partial (5%)
6174	actgagtgct	2	AF418550|Sma.67	30	7	Albumin precursor
6175	tcgcgacagc	2	C303525.1	25	0	

6176	taatacaact	2	AF030969|Sma.246	49	3	Clone 10 unknown
6177	gaaatatacc	2	C200837.1	40	8	
6177	gaaatatacc	2	C605319.1	30	9	
6178	cagggccgtt	2	TC7774	3	2	similar to SP|P77225|YDHR_ECOLI Protein ydhR precursor. {Shigella flexneri}, partial (16%)
6178	cagggccgtt	2	TC7775	9	1	
6179	ccggtgtccc	2				no_annot
6180	tgaaactgta	2	CD145459	55	0	
6181	ataaccgtcg	2				no_annot
6182	aagagccaat	2	TC14221	59	0	
6183	gtgagtcgaa	2	C318073.1	46	0	
6184	gatacgatgg	2	TC12443	65	0	weakly similar to GP|15099957|gb|AAK84178.1|AF384163_1|AF384163 diacylglycerol acyltransferase 2-like protein {Homo sapiens}, partial (17%)
6185	gaaaataata	2	Y12802|Sma.950	6	6	Amidase
6186	tttcaacagt	2				no_annot
6187	tccatcttcg	2				no_annot
6188	ttcaagcata	2	TC13251	37	0	similar to GP|12054502|emb|CAC20564.1||AJ401156 PD2 protein {Homo sapiens}, partial (8%)
6189	cagcccaact	2				no_annot
6190	tactttgatc	2				no_annot
6191	tatacacaca	2				no_annot
6192	gagaacccca	2				no_annot
6193	ttttattgaa	2				no_annot
6194	aaaaagcgga	2	TC15234	8	0	similar to GP|18447214|gb|AAL68198.1||AY075331 GH11935p {Drosophila melanogaster}, partial (5%)
6195	gcatacaaca	2				no_annot
6196	gtgattttga	2				no_annot
6197	tcaccacgta	2				no_annot
6198	caatgctttg	2				no_annot
6199	gcagactaag	2				no_annot
6200	agtagaccat	2				no_annot

6201	tatagctcat	2	TC11253	73	3	similar to GP|18479498|gb|AAL60763.1||AY073100 olfactory receptor MOR261-1 {Mus musculus}, partial (5%)
6202	catacacacg	2	CD096981	0	4	similar to GP|21428434|gb|AAM49877.1||AY118508 LD13350p {Drosophila melanogaster}, partial (3%)
6203	atggagacct	2				no_annot
6204	tgcgcaccat	2	C711050.1	96	0	
6205	cagaaaatat	2	TC13074	49	1	similar to SP|P43249|GRK5_BOVIN G protein-coupled receptor kinase GRK5. [Bovine] {Bos taurus}, partial (25%)
6206	gttgagtgca	2				no_annot
6207	ataaacaggt	2	TC18823	94	0	
6207	ataaacaggt	2	CD156115	44	0	
6208	gggtgagggc	2				no_annot
6209	gaatcttcca	2				no_annot
6210	tattcgatac	2	TC17291	99	0	weakly similar to GP|22324559|gb|AAM95612.1||AF520952 PHD protein Jade-1 {Homo sapiens}, partial (21%)
6211	ttcggttccc	2	TC14941	24	0	
6211	ttcggttccc	2	TC19283	20	2	
6212	tgaattacac	2	TC17482	68	0	
6213	tcgctgaagt	2	C208687.1	67	0	
6214	tcgtacaaga	2				no_annot
6215	taatttgcag	2				no_annot
6216	gactaccaat	2				no_annot
6217	gacaacgtaa	2	TC18934	24	1	weakly similar to SP|P58165|ATB2_OREMO Plasma membrane calcium-transporting ATPase 2(PMCA2) (Plasma membrane calcium pump isoform 2) (Plasma membrane calcium ATPase isoform 2) (Fragment). [)] {mossambica}, partial (8%)
6218	cggcgttact	2				no_annot
6219	aggatttaca	2	TC11335	54	2	
6220	ctttacatta	2				no_annot
6221	aatgttatgg	2				no_annot
6222	tgcctccaag	2				no_annot

6223	gtggtccttt	2	TC12466	47	0	
6224	cctccaagct	2				no_annot
6225	cagaataata	2				no_annot
6226	tgaatttcga	2	C201865.1	42	1	
6227	ggaaaaattt	2				no_annot
6228	gagaggtgat	2	TC10768	70	0	similar to SP|Q13573|SKIP_HUMAN Nuclear protein SkiP (Ski-interacting protein) (SNW1 protein) (Nuclear receptor coactivator NCoA-62). [Human] {Homo sapiens}, partial (68%)
6229	tacattcgct	2				no_annot
6230	ttgtcattaa	2				no_annot
6231	aacgtaaaac	2	TC9029	6	2	
6232	tttttaatct	2	TC8319	63	2	
6232	tttttaatct	2	TC10948	83	0	weakly similar to GP|7295709|gb|AAF51014.1||AE003576 CG15432-PA {Drosophila melanogaster}, partial (28%)
6233	tgaaactgga	2	C210912.1	78	0	
6233	tgaaactgga	2	C607169.1	72	0	
6234	actatattgt	2	C611643.1	66	0	
6235	aagtaatcat	2	TC11031	84	2	
6236	aatgaagtag	2				no_annot
6237	aatgaagtac	2	CD178657	56	0	
6238	atgctcatcc	2	TC8387	51	0	weakly similar to GP|1667611|gb|AAB18823.1||U76992 Tat-SF1 {Homo sapiens}, partial (9%)
6239	taatctagtt	2				no_annot
6240	aagccggact	2				no_annot
6241	gatggtagaa	2				no_annot
6242	taggttattc	2				no_annot
6243	ccctcccccc	2				no_annot
6244	tgtctttcac	2				no_annot
6245	tacattcgaa	2	TC14159	73	1	
6246	caatataagt	2				no_annot

6247	cgtattgacg	2	TC18568	60	1	weakly similar to SP|O43815|STRN_HUMAN Striatin. [Human] {Homo sapiens}, partial (13%)
6248	agaaaacttt	2				no_annot
6249	ataaagctaa	2	TC7646	47	0	similar to PIR|T03140|T03140 uracil-DNA glucosidase- alcelaphine herpesvirus 1, partial (7%)
6249	ataaagctaa	2	TC7647	57	0	
6250	gtaatgtaca	2	TC9897	80	0	
6251	acagatcaac	2	TC11083	87	0	weakly similar to SP|P41726|BVCP_GVCL DNA-binding protein (Arginine rich protein 7.3 kDa) (Basic viral core protein) (Nucleocapsid protein). [)] {leucotreta granulovirus}, partial (72%)
6252	cgagaaatga	2	TC14153	80	0	similar to GP|2098575|gb|AAB57629.1||AC002115 F25451_2 {Homo sapiens}, partial (34%)
6253	aaagaaagct	2				no_annot
6254	taacaaacgt	2	C201644.1	54	0	
6254	taacaaacgt	2	C602609.1	44	1	
6255	tgcaaagaat	2				no_annot
6256	caacaatctt	2				no_annot
6257	tagacctgtc	2	C208249.1	81	0	
6258	ctcacgtaac	2				no_annot
6259	atcatttatc	2	TC10832	91	0	
6260	gataaagcag	2				no_annot
6261	caaaaagatg	2				no_annot
6262	agtaaaatgc	2	CD145384	40	1	weakly similar to GP|28380248|gb|AAF50974.2||AE003575 CG12194-PA {Drosophila melanogaster}, partial (18%)
6263	gaattcatat	2	CD120409	40	1	
6264	aatggtcgag	1				no_annot
6265	aacgacaagt	1				no_annot
6266	gagatagatg	1				no_annot
6267	ttagcagacc	1				no_annot
6268	tttgggaatg	1				no_annot
6269	atacgtatac	1				no_annot

6270	ctgtgatctg	1	TC6880	73	4	similar to GP|17944974|gb|AAL48550.1||AY070928 RE03018p {Drosophila melanogaster}, partial (3%)
6270	ctgtgatctg	1	TC7036	7	8	similar to SP|Q12224|RLM1_YEAST Transcription factor RLM1. [Baker's yeast] {Saccharomyces cerevisiae}, partial (3%)
6270	ctgtgatctg	1	TC7243	69	1	similar to PIR|T39903|T39903 serine-rich protein - fission yeast (Schizosaccharomyces pombe), partial (4%)
6270	ctgtgatctg	1	CD138090	88	0	
6270	ctgtgatctg	1	CD153966	15	3	
6270	ctgtgatctg	1	CD154267	6	5	
6270	ctgtgatctg	1	CD097889	15	3	
6271	gttatgtgtc	1	TC11623	55	0	
6272	gtgaatgaca	1	TC8351	90	0	homologue to GP|18028649|gb|AAL56085.1|AF334030_10|AF334030 ORF79 {Helicoverpa zea single nucleocapsid nucleopolyhedrovirus}, partial (24%)
6273	atgcacgata	1				no_annot
6274	atagcggtgt	1				no_annot
6275	tttgtctact	1	TC18107	80	0	
6276	cacacacgca	1				no_annot
6277	gcggagaata	1				no_annot
6278	caaatcaact	1	AI740412	11	1	
6279	gaagttatgg	1				no_annot
6280	atagaaatgg	1				no_annot
6281	ctcaaatggg	1				no_annot
6282	aatgatgata	1	C709289.1	29	1	
6283	tatgattgtt	1	TC7547	64	3	similar to SP|P52565|GDIR_HUMAN Rho GDP-dissociation inhibitor 1 (Rho GDI 1) (Rho-GDI alpha). [Human] {Homo sapiens}, partial (15%)
6284	agttatgcaa	1	TC16178	66	0	
6285	aacagagggg	1				no_annot
6286	acaaccttgt	1				no_annot
6287	tatgattgtg	1				no_annot
6288	ttgtctaagt	1	C611222.1	80	4	

6289	ggtgttctgt	1	AF130788_1003_2262	68	2	[CDS] NADH dehydrogenase 4 (ND4) gene, complete cds
6290	gcgtgtaaaa	1	C609726.1	32	0	
6291	gtgaatgaat	1				no_annot
6292	tatttatgat	1	CD077589	23	1	
6293	gttgattacg	1				no_annot
6294	tactaaacca	1				no_annot
6295	gctttgcact	1				no_annot
6296	tcactgttta	1	C601718.1	4	1	
6297	ttataatctt	1				no_annot
6298	gaagtacatt	1				no_annot
6299	caggcaatta	1	CD140123	49	0	
6300	tttctgatgt	1	TC6868	50	3	similar to SP|Q22053|FBRL_CAEEL Fibrillarin. {Caenorhabditis elegans}, partial (78%)
6300	tttctgatgt	1	TC17956	25	2	
6301	gcatcacctg	1				no_annot
6302	cgtacagaat	1				no_annot
6303	ttacagactt	1	TC14325	47	0	weakly similar to GP|4514554|dbj|BAA75466.1||AB023967 Rod1 {Homo sapiens}, partial (25%)
6304	tggttttgtt	1	TC16740	68	1	
6305	tttgtttatc	1				no_annot
6306	ggaggtggtc	1				no_annot
6307	tagtgttagc	1				no_annot
6308	tttgtttata	1				no_annot
6309	tgtatccaaa	1				no_annot
6310	ggagatccta	1				no_annot
6311	taacttgact	1				no_annot
6312	gtcagttcaa	1	TC9695	84	0	
6313	cgtacagaag	1	C607082.1	10	5	
6314	tttgtctaat	1	TC17902	81	0	
6315	cctccgcacc	1				no_annot

6316	caaatcaaat	1	AW017392	71	1	
6317	gaaacctggg	1				no_annot
6318	ttacgttcat	1				no_annot
6319	agaccactgc	1	CD132012	16	0	
6320	acatatcagt	1				no_annot
6321	ttcaaatgca	1				no_annot
6322	ctgcgaaatt	1	TC16907	27	2	similar to SP|Q12873|CHD3_HUMAN Chromodomain helicase-DNA-binding protein 3 (CHD-3) (Mi-2 autoantigen 240 kDa protein) (Mi2-alpha). [Human] {Homo sapiens}, partial (3%)
6322	ctgcgaaatt	1	CD194985	83	0	weakly similar to SP|O43918|AIRE_HUMAN Autoimmune regulator (Autoimmune polyendocrinopathy candidiasis ectodermal dystrophy protein) (APECED protein). [Human] {Homo sapiens}, partial (7%)
6323	gggatttgta	1				no_annot
6324	actatcggca	1	TC19621	66	1	
6324	actatcggca	1	AI395375	76	0	
6325	tgtgctcgct	1				no_annot
6326	agtcccaaaa	1				no_annot
6327	gttgttccca	1				no_annot
6328	agcatcaccc	1				no_annot
6329	cttatttgca	1				no_annot
6330	attacagaca	1	TC11028	55	0	weakly similar to GP|2224741|dbj|BAA57829.1||D50088 G-3-P dehydrogenase {Drosophila novamexicana}, partial (21%)
6331	acacattgtc	1				no_annot
6332	atcaaagaat	1				no_annot
6333	tggagtagag	1	TC16987	69	1	weakly similar to SP|Q12904|MCA1_HUMAN Multisynthetase complex auxiliary component p43 [Contains: Endothelial-monocyte activating polypeptide II (EMAP-II) (Small inducible cytokine subfamily E member 1)]. [Human] {Homo sapiens}, partial (40%)
6334	cgcattaaat	1				no_annot
6335	gaagaagtag	1				no_annot
6336	gctatggtat	1	TC8213	78	1	

6337	aatattccca	1				no_annot
6338	gctttgcaat	1				no_annot
6339	tagcagcgca	1				no_annot
6340	accaccactt	1	TC18655	60	2	
6341	ttaattcgaa	1				no_annot
6342	atattgacga	1				no_annot
6343	gtcaaaaatt	1				no_annot
6344	agcatatgtt	1				no_annot
6345	gacaaaacaa	1				no_annot
6346	cattttacag	1	C608282.1	59	0	
6347	ccgataatga	1	TC7430	36	1	
6347	ccgataatga	1	TC7432	28	2	
6348	accaccactg	1	TC7458	67	0	weakly similar to GP|2104431|emb|CAB08743.1||Z95395 vacuolar sorting protein {Schizosaccharomyces pombe}, partial (5%)
6349	gttgtagttt	1				no_annot
6350	taaaacatcc	1				no_annot
6351	aagttggttg	1				no_annot
6352	tcgctgtact	1				no_annot
6353	cttttattga	1	TC15761	85	0	weakly similar to SP|P01122|RHO_APLCA RAS-like GTP-binding protein RHO. [California sea hare] {Aplysia californica}, partial (70%)
6354	agtggtaaag	1				no_annot
6355	tagaataagg	1				no_annot
6356	cacttactac	1				no_annot
6357	cttgcaggta	1				no_annot
6358	cgcacaaact	1				no_annot
6359	gatgtgaccc	1				no_annot
6360	tgtatgcgca	1	TC14336	65	1	weakly similar to GP|19343763|gb|AAH25586.1||BC025586 cDNA sequence BC025586 {Mus musculus}, partial (30%)
6361	cacctctgtg	1	C606301.1	11	1	
6362	cacttccaga	1	C610542.1	70	0	

6363	tgcacaaaag	1				no_annot
6364	ttctacgctg	1	TC18830	69	2	
6365	taatgtatgg	1				no_annot
6366	aatttacatt	1	TC12446	10	2	
6367	ataaattgtc	1				no_annot
6368	ttcccttccg	1				no_annot
6369	ctcgtcagca	1	C610444.1	91	0	
6370	ggtctaaaaa	1				no_annot
6371	taaatattat	1	TC11566	67	1	
6372	tctctcattt	1				no_annot
6373	taaatgagag	1				no_annot
6374	aaatgggtcg	1	CD146525	60	0	similar to GP|28381025|gb|AAO41479.1||BT003796 AT25873p {Drosophila melanogaster}, partial (7%)
6375	taaaatattg	1				no_annot
6376	ttgatgggag	1				no_annot
6377	taaatattac	1				no_annot
6378	gaacctcgta	1				no_annot
6379	agtccattca	1	TC10786	71	4	weakly similar to GP|17940124|gb|AAL49499.1|AF408422_1|AF408422 beta-catenin {Platynereis dumerilii}, partial (54%)
6380	gttacaaata	1	CD131102	7	2	
6381	ttgctatgct	1	C202227.1	10	0	
6382	gtttgcttat	1	TC11697	95	0	
6383	ttgaacgtat	1				no_annot
6384	tcaaacccaa	1				no_annot
6385	aaacaaatgt	1				no_annot
6386	tccttaactc	1				no_annot
6387	gcttttacaa	1	C211406.1	64	0	
6388	taaaacatac	1				no_annot
6389	atcaatatgc	1	TC7793	19	12	unknown
6390	atattaaagt	1				no_annot
6391	aacgtacgtt	1				no_annot

6392	gtttgcttag	1				no_annot
6393	ttatgcactt	1	TC14054	67	1	
6394	gtacccctga	1				no_annot
6395	gtggaggaac	1				no_annot
6396	cgcgtgaaca	1				no_annot
6397	actgggccat	1				no_annot
6398	gtggaggaaa	1				no_annot
6399	tccaggttga	1				no_annot
6400	gaagttataa	1				no_annot
6401	aacaaaccat	1				no_annot
6402	ttatgcactg	1	AI395602	37	0	
6403	ttttacatca	1	TC14370	78	1	
6404	gattcggttt	1				no_annot
6405	tccatacaat	1				no_annot
6406	atgcaagtgg	1				no_annot
6407	agctgagcaa	1	TC8443	37	1	similar to GP|3702848|gb|AAC62955.1||AF073333 reverse transcriptase SjR1 {Schistosoma japonicum}, partial (6%)
6408	aacgtacgta	1				no_annot
6409	tgctcttgct	1				no_annot
6410	agatgccact	1				no_annot
6411	tactttcatt	1	C209656.1	77	0	
6412	taaaaggcga	1				no_annot
6413	caaaatattt	1				no_annot
6414	caatgtatga	1				no_annot
6415	acaagaagtt	1				no_annot
6416	ccaagtggtg	1				no_annot
6417	aattttatct	1				no_annot
6418	gcagaccgac	1				no_annot
6419	gcaatattct	1	TC13567	13	2	
6419	gcaatattct	1	TC13569	43	2	

6420	aacaaaccaa	1				no_annot
6421	ctgtgtgtga	1				no_annot
6422	atgggtaatg	1				no_annot
6423	aaatccgaaa	1				no_annot
6424	acaattcata	1	CD074743	3	5	
6425	tagaagtgag	1				no_annot
6426	ctgcagctgg	1	TC15874	45	0	
6427	tagtttattg	1				no_annot
6428	aaaacgattc	1	TC12739	21	2	GP|6573266|gb|AAF17611.1||AF176706 F-box protein FBX11 {Homo sapiens}, partial (6%)
6429	taaccttttg	1	U10431|Sma.680	12	4	Open reading frame
6430	ttcagtagtt	1	AI975641	66	1	similar to SP|P32926|DSG3_ Desmoglein 3 precursor (130 kDa pemphigus vulgaris antigen) (PVA). [Human] {Homo sapiens}, partial (1%)
6431	atgcttttta	1				no_annot
6432	gggtacattt	1				no_annot
6433	caaagagttt	1	AI395007	28	1	
6434	tgggtttgca	1				no_annot
6435	ctgaacgtat	1				no_annot
6436	ttagctatca	1	C200943.1	51	6	
6436	ttagctatca	1	C608614.1	48	6	
6437	atgaaaattc	1	AY090636|Sma.96	96	0	Na+/Cl- dependent neurotransmitter transporter-like protein (NAT)
6438	acgagatcca	1	C603383.1	7	5	
6439	ttgctatgac	1	C201691.1	67	0	
6440	taatcctgaa	1	C204352.1	32	0	
6441	ttgatgtaaa	1	CD090263	81	0	
6441	ttgatgtaaa	1	AI976162	38	1	
6442	tagaataacg	1				no_annot
6443	gacatccaag	1				no_annot
6444	gtccaagaat	1				no_annot
6445	tggatatatg	1	TC19371	3	0	

6446	cgtatgcgaa	1				no_annot
6447	attcggtcca	1				no_annot
6448	gaagatgatg	1	TC16948	78	3	similar to PIR|PC4365|PC4365 calcium-binding protein - tapeworm (Echinococcus granulosus) (fragment), partial (43%)
6449	ttaataacta	1				no_annot
6450	taacgaagta	1				no_annot
6451	tattccctgg	1				no_annot
6452	tggcttatcc	1				no_annot
6453	acaaatgtaa	1				no_annot
6454	tctattctcc	1				no_annot
6455	tcactgatat	1				no_annot
6456	ggtgaatgag	1				no_annot
6457	gtctccctca	1	TC13584	48	4	homologue to GP|18250327|gb|AAL60588.1||AY071854 MHC class I antigen {Oncorhynchus mykiss}, partial (6%)
6458	gtaaatgtgt	1				no_annot
6459	attgaagctg	1	CD119263	42	1	
6460	gcgtatccag	1				no_annot
6461	atggcgcagt	1				no_annot
6462	cacattgttg	1				no_annot
6463	aactactata	1				no_annot
6464	taccaatata	1	TC13204	96	0	
6464	taccaatata	1	CD133573	93	0	
6465	tcattctagc	1				no_annot
6466	gtactttcat	1				no_annot
6467	ggcaatcggg	1	TC16735	65	5	weakly similar to GP|6048361|gb|AAF02213.1|AF067202_1|AF067202 lactate dehydrogenase B4 {Danio rerio}, partial (69%)
6467	ggcaatcggg	1	TC16737	33	2	weakly similar to GP|13529599|gb|AAH05509.1||BC005509 Ldh1 protein {Mus musculus}, partial (34%)
6468	tcgatgtgtt	1				no_annot
6469	tgcacataga	1				no_annot

6470	aggttgtaaa	1	CD073101	37	0	
6471	aacataattg	1	TC10455	80	0	weakly similar to GP|7294767|gb|AAF50102.1||AE003546 CG7828-PA {Drosophila melanogaster}, partial (10%)
6472	tgtgtaagga	1				no_annot
6473	caatatggca	1	C206768.1	95	0	
6473	caatatggca	1	C605002.1	95	0	
6474	aacagataag	1				no_annot
6475	atataatttg	1				no_annot
6476	tccagatctg	1				no_annot
6477	cgaaatagac	1				no_annot
6478	atataattta	1	TC16534	43	0	
6478	atataattta	1	CD153124	35	2	similar to GP|2145046|gb|AAB58406.1||AF000409 merozoite surface protein-1 {Plasmodium berghei}, partial (13%)
6479	gtacattgtc	1				no_annot
6480	atatgttgag	1				no_annot
6481	ctatttgacg	1				no_annot
6482	tcatacataa	1				no_annot
6483	tacacagttg	1				no_annot
6484	tcagatatgt	1	TC8575	32	0	weakly similar to GP|15291141|gb|AAK92839.1||AY051415 GH09360p {Drosophila melanogaster}, partial (19%)
6485	cttttctaga	1				no_annot
6486	gtaacggcaa	1				no_annot
6487	ctgatgtaac	1				no_annot
6488	cggagttggg	1				no_annot
6489	gcaatcggct	1				no_annot
6490	ctcagtagta	1				no_annot
6491	gggttcggtt	1				no_annot
6492	cagaataaca	1				no_annot
6493	agaatacaat	1				no_annot
6494	tggataaaga	1				no_annot

6495	ctaatgatct	1				no_annot
6496	aatctctctc	1				no_annot
6497	tattactgct	1				no_annot
6498	tttgcaaaca	1	TC13507	3	4	homologue to GP|3063620|gb|AAC14119.1||AF056330 AUT1 {Schistosoma mansoni}, partial (16%)
6498	tttgcaaaca	1	TC13512	83	0	homologue to GP|3063620|gb|AAC14119.1||AF056330 AUT1 {Schistosoma mansoni}, partial (11%)
6499	ctaatcgtgc	1	L01634|Sma.848	84	3	Myosin heavy chain (MYH)
6500	ccattattat	1	C206085.1	83	0	
6501	tctgttagtt	1	TC14237	62	1	
6502	acagtgcatt	1	C606865.1	42	2	
6503	aactacaaga	1	TC13272	28	4	
6504	tattgaaaca	1	TC8658	34	0	
6504	tattgaaaca	1	CD202456	38	2	
6505	gatttgacgg	1				no_annot
6506	tcatttggtg	1	TC8059	70	1	weakly similar to GP|4104873|gb|AAD11976.1||AF040386 calponin homolog {Schistosoma japonicum}, partial (56%)
6507	tcacttgttt	1				no_annot
6508	cacacagttt	1				no_annot
6509	tacgcacagc	1				no_annot
6510	aagtgatggt	1				no_annot
6511	aaataaagaa	1				no_annot
6512	cactcacatt	1				no_annot
6513	atattaaaat	1	TC10090	41	0	
6513	atattaaaat	1	H98384	11	0	
6514	aattcaatat	1	TC8979	6	5	weakly similar to GP|6561829|gb|AAF17084.1|AF202893_1|AF202893 Kif21b {Mus musculus}, partial (3%)
6514	aattcaatat	1	TC16996	90	1	
6515	aatctggaag	1				no_annot
6516	tgcacgtcat	1				no_annot
6517	tattattgtt	1	TC18496	48	0	

6517	tattattgtt	1	CD113082	70	0	
6517	tattattgtt	1	CD160666	36	0	
6518	ctagctatac	1	AY158217|Sma.772	59	1	Rac GTPase (Rac)
6519	tttgggaagt	1				no_annot
6520	catttttcct	1	TC10391	92	0	weakly similar to GP|1575609|gb|AAC50893.1||U69127 FUSE binding protein 3 {Homo sapiens}, partial (6%)
6521	aaaggagcac	1				no_annot
6522	attgttacta	1				no_annot
6523	ggacttttat	1				no_annot
6524	agtagcttta	1				no_annot
6525	acgtcagtca	1				no_annot
6526	tattattgtg	1				no_annot
6527	tgattatagc	1				no_annot
6528	atattaaaaa	1	TC19612	16	0	
6529	aatgtacaag	1				no_annot
6530	atccctttcc	1				no_annot
6531	cgataaatct	1				no_annot
6532	tgactgaaga	1	TC17392	92	0	homologue to GP|24496255|gb|AAN59958.1||AY131972 histone H2A {Homo sapiens}, partial (95%)
6533	cacactccac	1				no_annot
6534	tgtactttgt	1				no_annot
6535	tacacgaata	1				no_annot
6536	tctgtacctc	1				no_annot
6537	ctgaaatctt	1				no_annot
6538	gtgacttaga	1				no_annot
6539	gataattgtc	1				no_annot
6540	gaaggaactc	1				no_annot
6541	ttccatccaa	1	TC19707	74	0	
6542	tattcatcct	1				no_annot
6543	attgacagtc	1				no_annot
6544	ttgaaaacgc	1				no_annot

6545	acagcgatgc	1	TC6953	53	0	
6545	acagcgatgc	1	TC7007	56	0	similar to SP|P35661|GT27_SCHMA Glutathione S-transferase 26 kDa(GST 26) (SM26/2 antigen) (GST class-alpha). [Blood fluke] {Schistosoma mansoni}, partial (12%)
6545	acagcgatgc	1	TC7246	63	0	
6545	acagcgatgc	1	CD183658	30	0	
6545	acagcgatgc	1	CD114231	43	0	
6546	tccactaagt	1				no_annot
6547	ttttacttgg	1	TC7391	84	0	
6548	aatagtttag	1	C603504.1	96	0	
6549	aatgatgaga	1	BF937048	44	1	
6550	ttatatgtca	1	TC16156	62	0	
6550	ttatatgtca	1	CD132937	33	1	
6551	tcagttgggc	1				no_annot
6552	tgactagtct	1				no_annot
6553	tgtcattgca	1	TC16174	31	3	
6554	gatagaaaaa	1	TC7518	98	0	similar to GP|15824396|gb|AAL09322.1|AF303222_1|AF303222 SNaK1 {Schistosoma mansoni}, partial (41%)
6554	gatagaaaaa	1	CD118659	65	0	
6555	tggaaatttg	1				no_annot
6556	gcgatacgcg	1				no_annot
6557	tcctctccat	1				no_annot
6558	gcgaataaat	1				no_annot
6559	gaggctgcag	1				no_annot
6560	tttacttcca	1	CD149869	54	0	
6561	gagtatttga	1				no_annot
6562	cttagtaacc	1				no_annot
6563	ctgtcttaga	1	C201585.1	84	0	
6563	ctgtcttaga	1	C606780.1	82	0	
6564	ggaggtgggc	1				no_annot

6565	caattagttt	1	CD081740	81	1	weakly similar to GP|27352995|dbj|BAC49996.1||AP005952 bll4731 {Bradyrhizobium japonicum USDA 110}, partial (10%)
6566	aaacagtgtg	1				no_annot
6567	tacataacgc	1				no_annot
6568	cttgatatct	1				no_annot
6569	gtaatccaag	1				no_annot
6570	acgttatcca	1				no_annot
6571	tcggatcgtg	1				no_annot
6572	atcaggaaat	1				no_annot
6573	tgtcctctca	1				no_annot
6574	cagcatcagg	1	TC11823	26	1	similar to SP|Q15427|S3B4_HUMAN Splicing factor 3B subunit 4 (Spliceosome associated protein 49) (SAP 49) (SF3b50) (Pre-mRNA splicing factor SF3b 49 kDa subunit). [Human] {Homo sapiens}, partial (41%)
6575	gcaatctaat	1				no_annot
6576	cacggaagca	1				no_annot
6577	atgcttatat	1				no_annot
6578	ggaatgtcat	1				no_annot
6579	cgataaataa	1				no_annot
6580	caattagttc	1				no_annot
6581	agaaatgact	1				no_annot
6582	ctacaaggtg	1				no_annot
6583	gtcattagta	1				no_annot
6584	atatactatt	1				no_annot
6585	aaatactcaa	1				no_annot
6586	atgataaagg	1				no_annot
6587	ggctccggct	1				no_annot
6588	gtaactatgg	1				no_annot
6589	taaaaagaac	1	CD096883	57	1	
6590	gccatccttt	1				no_annot
6591	cctgcggaga	1				no_annot

6592	tcaacttcca	1				no_annot
6593	attttctctt	1				no_annot
6594	tcgatattct	1	CD154178	80	0	
6595	cgcgatgttt	1	TC13521	19	8	similar to GP|7300349|gb|AAF55508.1||AE003721 CG7187-PA {Drosophila melanogaster}, partial (5%)
6595	cgcgatgttt	1	TC13523	23	2	similar to GP|18160467|gb|AAL63818.1||AE009847 phoH like protein {Pyrobaculum aerophilum}, partial (5%)
6595	cgcgatgttt	1	CD095083	4	1	
6595	cgcgatgttt	1	CD095083	4	0	
6596	cttagtaaat	1				no_annot
6597	ttttcatatg	1	CD124458	22	1	
6598	taaatgtgaa	1				no_annot
6599	caaatgtgcg	1				no_annot
6600	cttttctaag	1				no_annot
6601	gggaatgccg	1				no_annot
6602	tccaaccact	1				no_annot
6603	tgcaggggct	1	TC13587	48	1	similar to GP|28200274|gb|AAO31769.1||AY168758 ribosomal protein L10 {Branchiostoma belcheri tsingtaunese}, partial (90%)
6604	cttttctaac	1				no_annot
6605	gttacatatc	1				no_annot
6606	tttctatctc	1				no_annot
6607	tggctgccga	1	TC17241	60	3	weakly similar to GP|10048296|gb|AAG12342.1||AF294845 glycerol-3-phosphate dehydrogenase {Mus musculus}, partial (54%)
6608	ttgattgact	1	TC7324	20	1	
6609	tgagactaca	1				no_annot
6610	aaatatacgc	1				no_annot
6611	ggcgctaaaa	1				no_annot
6612	ccctctccac	1				no_annot
6613	aacttataac	1				no_annot
6614	attagatgtg	1				no_annot

6615	gcgcatcagt	1				no_annot
6616	tccttatcta	1				no_annot
6617	ccattttata	1				no_annot
6618	gagcacacca	1				no_annot
6619	caggagaaaa	1				no_annot
6620	cgagatggtg	1				no_annot
6621	acaagcacca	1				no_annot
6622	atattccctg	1				no_annot
6623	gctgtaattc	1				no_annot
6624	attttggaat	1				no_annot
6625	tgtactttcc	1	C202678.1	50	1	
6626	ggttgctgta	1				no_annot
6627	aagttggtga	1	CD197577	41	2	
6628	tgtgaagcgc	1				no_annot
6629	caatgtttgt	1				no_annot
6630	caagtgtata	1				no_annot
6631	tacgcacaat	1	C202178.1	42	5	
6631	tacgcacaat	1	C609804.1	46	5	
6632	gccctgctaa	1				no_annot
6633	ggtgattcct	1	C201330.1	51	1	
6633	ggtgattcct	1	C609794.1	25	3	
6634	atataaatat	1	CD059695	73	2	
6635	tgtagaagct	1				no_annot
6636	tataagacgt	1				no_annot
6637	tagtaagtgg	1	C207580.1	16	5	
6637	tagtaagtgg	1	C605859.1	15	7	
6638	caacaatcac	1				no_annot
6639	ttgatactag	1				no_annot
6640	caattgaatc	1				no_annot
6641	tgtgtgcagt	1				no_annot

6642	ccacctccac	1	TC10626	70	1	weakly similar to GP|3142634|gb|AAC78612.1||AF063665 small nuclear ribonucleoprotein N {Mus musculus}, partial (36%)
6643	tggtctacgg	1				no_annot
6644	cacgcacacg	1				no_annot
6645	gcaaaagacc	1				no_annot
6646	cttggagtat	1				no_annot
6647	gatagccctt	1				no_annot
6648	cacgcacaca	1	TC7881	59	2	
6648	cacgcacaca	1	CD069923	55	0	weakly similar to PIR|I46880|I46880 T-cell receptor beta chain - rabbit (fragment), partial (18%)
6649	aaaaaagcta	1	CD085895	2	0	
6650	atcagtgatg	1				no_annot
6651	taaattaaat	1				no_annot
6652	taaaatatga	1				no_annot
6653	aaagctcata	1				no_annot
6654	ggaggtggct	1				no_annot
6655	agtagcatcg	1				no_annot
6656	cataaaggtc	1	TC12528	90	0	
6657	gagctatcaa	1				no_annot
6658	cacaccgatg	1				no_annot
6659	gttacaaagg	1				no_annot
6660	acaagatgta	1	TC10931	4	1	weakly similar to SP|P28687|SP22_CHICK Microsomal signal peptidase 23 kDa subunit(SPase 22 kDa subunit) (SPC22/23) (gp23). [Chicken] {Gallus gallus}, partial (52%)
6661	ccagtttaga	1				no_annot
6662	actgtgaaat	1				no_annot
6663	gtagctcgaa	1				no_annot
6664	tatgtcaatt	1				no_annot
6665	tgagcgctga	1				no_annot
6666	ccgtgcttag	1				no_annot
6667	tggagcattc	1	TC14499	62	0	

6668	aaacgagatc	1	TC8307	35	0	
6669	tagaattact	1				no_annot
6670	tatccaaaaa	1				no_annot
6671	aacgagtgct	1				no_annot
6672	tacagtcaat	1				no_annot
6673	tataaatata	1	C312949.1	67	0	
6673	tataaatata	1	C602227.1	44	2	
6673	tataaatata	1	C716514.1	42	2	
6674	ataactctgc	1	TC13881	68	0	weakly similar to GP|16768574|gb|AAL28506.1||AY060958 GM08907p {Drosophila melanogaster}, partial (16%)
6675	actacgtcgt	1	U24281|Sma.721	0	3	14-3-3 protein (Sm14-3-3)
6676	gatgaattgt	1				no_annot
6677	tagaattacg	1	TC8243	12	1	
6678	ttgattgaaa	1				no_annot
6679	ctggtggagt	1	TC12836	67	0	weakly similar to GP|13623199|gb|AAH06195.1|AAH06195|BC006195 ATP citrate lyase {Homo sapiens}, partial (7%)
6680	ggtatatgta	1				no_annot
6681	ccgcttgaat	1				no_annot
6682	cagcatcacc	1				no_annot
6683	aggtatgtgt	1				no_annot
6684	tgtattcaat	1	CD154104	41	0	
6685	tgtacgccat	1				no_annot
6686	gcgaattaga	1				no_annot
6687	ctcagctgct	1	C200032.1	22	3	
6687	ctcagctgct	1	C611578.1	32	3	
6688	tctgaaaaaa	1				no_annot
6689	cgtgcgttgg	1	TC12061	40	1	similar to GP|27316434|gb|AAO05609.1|AE016750_214|AE016750 nitrite extrusion protein {Staphylococcus epidermidis ATCC 12228}, partial (5%)
6690	tgtattcaag	1				no_annot

6691	tcgaaggcga	1				no_annot
6692	acaagaaggt	1				no_annot
6693	tcgccagaat	1	TC18020	47	1	weakly similar to GP|6648543|gb|AAF21219.1|U62056_1|U62056 TyrA {Vibrio cholerae}, partial (18%)
6694	gacattcagt	1				no_annot
6695	cggcccaacc	1				no_annot
6696	aaaaaggtat	1	TC11755	57	0	
6696	aaaaaggtat	1	CD183044	21	0	
6697	gatgtataaa	1				no_annot
6698	aagcgcggcc	1				no_annot
6699	tcatagcctt	1				no_annot
6700	tgctttgata	1	TC9552	70	0	
6701	tgatgctttg	1				no_annot
6702	atcttgaacc	1				no_annot
6703	acaattcaga	1				no_annot
6704	cgcagccata	1				no_annot
6705	aatatgaaga	1				no_annot
6706	ctgaatgtgg	1				no_annot
6707	taagtagtag	1				no_annot
6708	cataaatatt	1	C210228.1	91	0	
6709	atcaaacaga	1				no_annot
6710	taagtagtaa	1				no_annot
6711	tttgtttaaa	1	CD148165	87	0	
6712	tcatttaaca	1	C606688.1	6	3	
6713	cttctaacga	1				no_annot
6714	ctgctttcag	1				no_annot
6715	atgaaaatgg	1	TC12222	72	1	
6715	atgaaaatgg	1	TC14215	70	0	similar to SP|Q9U1H8|FAC2_DROME CAAX prenyl protease 2(Prenyl protein-specific endoprotease 2) (Farnesylated-proteins converting enzyme 2) (FACE-2) (Severas protein). [Fruit fly] {Drosophila melanogaster}, partial (8%)

6716	gcgacctgat	1	TC9115	30	3	
6717	atttcgtttg	1				no_annot
6718	ctggtaagtc	1				no_annot
6719	ggataacgat	1				no_annot
6720	ctaacgaatt	1	gi|160942|gb|M21607.1|SCMCHRA	22	1	S.mansoni eggshell (chorion) protein gene, complete cds
6721	accaaccctc	1				no_annot
6722	taaactgctt	1				no_annot
6723	tgtgcatctg	1	CD137428	64	0	
6724	gcaaagccta	1				no_annot
6725	tgttcaactt	1				no_annot
6726	aaacaattcg	1				no_annot
6727	catattagtg	1				no_annot
6728	tatcatataa	1	TC17767	53	4	similar to GP|19528535|gb|AAL90382.1||AY089644 RE71924p {Drosophila melanogaster}, partial (77%)
6729	ttggctcggg	1				no_annot
6730	tagaattaac	1				no_annot
6731	aacaacatta	1	TC13879	35	5	weakly similar to GP|7295350|gb|AAF50668.1||AE003563 CG10173-PA {Drosophila melanogaster}, partial (9%)
6732	agattgttcc	1				no_annot
6733	actaggaacg	1				no_annot
6734	ggtacaactt	1				no_annot
6735	actcttcaca	1				no_annot
6736	tgacaaactg	1				no_annot
6737	ataggcatat	1				no_annot
6738	tggatataga	1	TC17856	88	0	
6739	aggctcagga	1				no_annot
6740	gtgtcgaacc	1				no_annot
6741	ctatctatca	1				no_annot
6742	ccatcaaagc	1				no_annot
6743	caggttttgt	1				no_annot
6744	tggtgtggtg	1				no_annot

6745	caaaaacata	1	TC13213	70	0	
6746	gcggcaatat	1				no_annot
6747	aacagcccat	1				no_annot
6748	aaatatacat	1	TC18924	64	0	
6749	tgtagttctt	1				no_annot
6750	tgaatgagta	1	C307249.1	0	1	
6751	caagagtttt	1				no_annot
6752	ttaccctctt	1	TC13776	45	5	weakly similar to GP|262250|gb|AAB24621.1||S52010 1st Met is at position 21 {Mus sp.}, partial (22%)
6753	tcatatatga	1				no_annot
6754	cctttagcac	1	TC8343	40	1	
6755	tgcttgtagc	1				no_annot
6756	ccagtttaca	1				no_annot
6757	ttagcacaca	1	TC18854	14	1	
6758	tatatgctaa	1				no_annot
6759	tcgggtttat	1				no_annot
6760	cagtttatgc	1	TC11391	68	0	
6761	taaagagtcg	1	C204890.1	87	0	
6761	taaagagtcg	1	C609652.1	81	0	
6762	cataaaaaga	1	CD063670	93	0	
6763	caatagatta	1				no_annot
6764	agcatatgaa	1				no_annot
6765	acaacacagc	1				no_annot
6766	aaagcgcgaa	1	C201199.1	2	10	
6766	aaagcgcgaa	1	C610885.1	4	4	
6767	atcaagccag	1	TC11151	64	3	similar to GP|7303935|gb|AAF58979.1||AE003834 CG8057-PA {Drosophila melanogaster}, partial (26%)
6767	atcaagccag	1	CD134016	8	2	similar to SP|O43741|AAKC_HUMAN 5'-AMP-activated protein kinase, beta-2 subunit (AMPK beta-2 chain). [Human] {Homo sapiens}, partial (17%)
6768	tcagttcgta	1				no_annot
6769	ctgtaggtta	1				no_annot

6770	aagacgtaac	1				no_annot
6771	gggactacat	1	C717040.1	75	0	
6772	caaccgccga	1				no_annot
6773	tatgtatttg	1	AA559753	48	0	
6774	catacaagtt	1	TC12514	9	0	
6775	gaaggatcta	1				no_annot
6776	gctgctctcc	1	CD183585	46	0	
6777	gggctaactt	1				no_annot
6778	caaacaaaac	1				no_annot
6779	tggtctacag	1				no_annot
6780	tgaataatgt	1				no_annot
6781	acggaatgat	1				no_annot
6782	tccacttagt	1				no_annot
6783	atccgtacca	1				no_annot
6784	tattgataag	1				no_annot
6785	ctcagaccgg	1	C607353.1	89	0	
6786	gaatccaact	1				no_annot
6787	gatataattg	1	TC15780	74	1	similar to GP|15149021|gb|AAK84971.1||AF325528 LSDV010 LAP/PHD-finger protein {lumpy skin disease virus} [Lumpy skin disease virus], partial (9%)
6788	accaaaggaa	1	CD184766	26	0	
6789	aacctccata	1				no_annot
6790	tagctaagta	1				no_annot
6791	atattacgtc	1				no_annot
6792	ttttatatca	1	CD184344	31	2	
6793	taacctttca	1	TC10599	74	0	
6794	agtttcatct	1	C609521.1	88	0	
6795	ctcctggcat	1				no_annot
6796	tatatctgga	1				no_annot
6797	actaatttga	1				no_annot
6798	tagacaccat	1				no_annot

6799	taacgaagct	1				no_annot
6800	tgcatcgtta	1				no_annot
6801	agtgtagaat	1	CD196688	35	2	
6802	tcaatcatcg	1				no_annot
6803	taatgacaaa	1				no_annot
6804	caatgacacg	1	C208540.1	47	1	
6804	caatgacacg	1	C602466.1	45	1	
6805	acagactgtc	1				no_annot
6806	aacgtacgac	1				no_annot
6807	tttttttagt	1				no_annot
6808	tcatatatct	1				no_annot
6809	catagcaata	1				no_annot
6810	taccaatact	1	TC18738	6	2	weakly similar to GP|10954046|gb|AAG25715.1|AF309387_1|AF309387 oxidation protection protein {Homo sapiens}, partial (10%)
6811	gcttggtcaa	1	TC19581	93	0	
6812	cttgcataaa	1				no_annot
6813	agctgtagct	1				no_annot
6814	catttaattt	1				no_annot
6815	taattagtga	1	TC13661	55	4	similar to GP|13436359|gb|AAH04964.1|AAH04964|BC004964 oxoglutarate dehydrogenase (lipoamide) {Homo sapiens}, partial (70%)
6816	cttatcatcc	1				no_annot
6817	gaaatgtatt	1	TC10779	87	1	similar to GP|9800238|gb|AAF99112.1|AF232689_3|AF232689 pr2.1 {rat cytomegalovirus Maastricht} [Rat cytomegalovirus Maastricht], partial (4%)
6818	aagagagcgt	1				no_annot
6819	ttcacggttt	1				no_annot
6820	aacgatggga	1				no_annot
6821	cagatgaatt	1	TC7409	62	7	similar to SP|Q00610|CLH1_HUMAN Clathrin heavy chain 1 (CLH-17). [Human] {Homo sapiens}, partial (95%)

6822	ctgaaatcga	1				no_annot
6823	atggcttccg	1	CD126185	30	2	
6824	cccacttagt	1				no_annot
6825	cacattgtca	1				no_annot
6826	catttaattg	1				no_annot
6827	tgtgtatgac	1	C300610.1	49	0	
6827	tgtgtatgac	1	C719303.1	49	0	
6828	caactggtta	1	C202798.1	39	0	
6829	tcaattattg	1	TC12490	83	0	weakly similar to GP|8037915|gb|AAF71530.1|AF252293_1|AF252293 partitioning-defective 3 splice variant c {Homo sapiens}, partial (3%)
6830	atttcaactg	1	CD178619	8	2	
6831	cacaactctc	1				no_annot
6832	tcgatgtgct	1				no_annot
6833	catacggact	1				no_annot
6834	gacattcaaa	1				no_annot
6835	atacaaacta	1	CD189832	72	1	
6836	cctgaatagc	1				no_annot
6837	tgaggttttt	1				no_annot
6838	atttcaacta	1	TC7313	18	0	
6838	atttcaacta	1	TC7328	12	1	
6838	atttcaacta	1	TC8290	22	3	
6838	atttcaacta	1	TC9338	32	2	
6838	atttcaacta	1	TC17895	80	0	similar to GP|2327067|gb|AAC53319.1||AF008439 natural resistance-associated macrophage protein 2 {Rattus norvegicus}, partial (21%)
6838	atttcaacta	1	CD097726	14	2	
6838	atttcaacta	1	CD124339	69	0	
6838	atttcaacta	1	CD127705	23	2	
6838	atttcaacta	1	CD133685	93	0	GP|19067879|g polyprotein {Schistosoma japonicum}, partial (1%)

6838	atttcaacta	1	CD085197	44	1	homologue to GP|29898730|gb|AAP12002.1||AE017014 Murein hydrolase exporter {Bacillus cereus ATCC 14579}, partial (10%)
6838	atttcaacta	1	CD090477	33	1	
6838	atttcaacta	1	CD167183	19	1	
6839	cagatgaatg	1	TC13998	12	3	
6840	taaccttac	1				no_annot
6841	cagtttatcg	1	TC8161	79	0	
6842	atgcagcgtt	1				no_annot
6843	tccagatcca	1				no_annot
6844	atgcgtgttt	1				no_annot
6845	ttggtgcatt	1				no_annot
6846	gtgacgattt	1	CD196642	68	0	
6847	atgaacaagc	1				no_annot
6848	ttactttgtc	1				no_annot
6849	atactcgaaa	1				no_annot
6850	agatgtcaac	1				no_annot
6851	accgtcaaaa	1				no_annot
6852	aaacactaca	1				no_annot
6853	tcagatttag	1				no_annot
6854	gttcctttcc	1				no_annot
6855	caatgacaaa	1				no_annot
6856	ccagaacaga	1				no_annot
6857	ggattcttta	1				no_annot
6858	ccacaaagtt	1				no_annot
6859	cgaatcggtg	1				no_annot
6860	attaatctct	1				no_annot
6861	acactaatgc	1				no_annot
6862	ctttgagtcc	1	C201169.1	17	2	
6863	gcctctttca	1	TC7123	3	3	
6863	gcctctttca	1	TC7123	3	1	

6863	gcctctttca	1	TC8846	60	3	
6863	gcctctttca	1	CD096709	12	0	
6863	gcctctttca	1	CD144245	35	0	
6864	cttgctgcga	1	TC16847	53	2	weakly similar to EGAD|25135|3076 heterogeneous nuclear ribonucleoprotein K {Homo sapiens}, partial (19%)
6865	ccagagccct	1				no_annot
6866	gtgtcgtagt	1				no_annot
6867	aattcgtgga	1				no_annot
6868	tcggtgtatc	1				no_annot
6869	ccagcacgtt	1				no_annot
6870	cttttttagg	1				no_annot
6871	tgcttgtaac	1	TC12342	8	1	
6872	accacaagcg	1				no_annot
6873	ctatctttgg	1				no_annot
6874	acgtcgcgtt	1	TC17179	30	1	weakly similar to GP|5679126|gb|AAD46869.1|AF160929_1|AF160929 BcDNA.LD12153 {Drosophila melanogaster}, partial (43%)
6875	actacataaa	1				no_annot
6876	tgttcgttgt	1				no_annot
6877	tatctccgag	1				no_annot
6878	gctgacattg	1				no_annot
6879	aatctgcagt	1				no_annot
6880	gctgacattc	1				no_annot
6881	ccaattatta	1				no_annot
6882	attgtatgct	1				no_annot
6883	ttggtatgtt	1				no_annot
6884	cataaaaaaa	1				no_annot
6885	tcgattaact	1	AY698060|Sma.5386	85	0	TLL (TLL)
6886	cgggatccct	1	TC7111	61	0	similar to GP|14799394|emb|CAC44272.1||AJ311840 XNop56 protein {Xenopus laevis}, partial (36%)
6887	tttgggtaag	1				no_annot

6888	aagggaatta	1				no_annot
6889	gggaggtgat	1				no_annot
6890	ttaacagtgc	1	C610909.1	44	0	
6891	tcgacgacca	1				no_annot
6892	ttatcacagc	1				no_annot
6893	tggtggtgag	1				no_annot
6894	tatcatttcc	1	TC16844	46	1	similar to GP|6746611|gb|AAF27650.1|AF218064_1|AF218064 malate dehydrogenase precursor {Nucella lapillus}, partial (88%)
6895	ggtatgcaga	1				no_annot
6896	tatcatttca	1				no_annot
6897	gatgagtggg	1				no_annot
6898	aaacataagt	1				no_annot
6899	ttgaaatcaa	1				no_annot
6900	atcttccata	1				no_annot
6901	ggatatccca	1				no_annot
6902	gatgagtgga	1				no_annot
6903	gattacaaca	1	TC10995	70	0	weakly similar to SP|P24547|IMD2_MOUSE Inosine-5'-monophosphate dehydrogenase 2(IMP dehydrogenase 2) (IMPDH-II) (IMPD 2). [Mouse] {Mus musculus}, partial (83%)
6903	gattacaaca	1	TC11775	39	1	weakly similar to GP|12061185|gb|AAG45474.1||AY013288 ASC-1 complex subunit P200 {Homo sapiens}, partial (5%)
6904	taaagtgatg	1				no_annot
6905	ccggtgtatc	1				no_annot
6906	cttacaatta	1				no_annot
6907	tgaatcaagg	1	CD073625	18	1	
6908	tatatctgaa	1	TC16519	75	0	
6909	ttgtccgtta	1				no_annot
6910	gattaattga	1				no_annot
6911	cctttctgga	1				no_annot
6912	tcatcgtcct	1				no_annot

6913	actaagccat	1	C609637.1	24	2	
6914	gatgactaat	1	C202178.1	72	2	
6914	gatgactaat	1	C607224.1	28	2	
6914	gatgactaat	1	C609804.1	74	2	
6915	aggcgcaata	1	C204475.1	4	0	
6916	acattaagcg	1				no_annot
6917	cactggcagg	1				no_annot
6918	accacctcct	1				no_annot
6919	cgatattata	1	TC18730	62	0	
6920	attgtatgaa	1				no_annot
6921	ctggttactg	1				no_annot
6922	aaatattcca	1	TC13782	34	0	
6922	aaatattcca	1	AA566154	18	1	
6922	aaatattcca	1	CD191761	78	0	weakly similar to GP|28626247|gb|AAO49153.1||AF536748 calcium-permeable store-operated channel TRPM3a {Homo sapiens}, partial (3%)
6923	tttttttaat	1	TC8351	27	5	homologue to GP|18028649|gb|AAL56085.1|AF334030_10|AF334030 ORF79 {Helicoverpa zea single nucleocapsid nucleopolyhedrovirus}, partial (24%)
6923	tttttttaat	1	AI975736	31	0	
6924	tgggagaatg	1	C612420.1	81	0	
6925	ctttttggaa	1				no_annot
6926	gtcgacttta	1	CD065377	20	3	homologue to SP|P20287|G3P_SCHMA Glyceraldehyde 3-phosphate dehydrogenase(GAPDH) (Major larval surface antigen) (P-37). [Blood fluke] {Schistosoma mansoni}, partial (20%)
6926	gtcgacttta	1	AI977709	28	3	SP|P20287|G3P_SCHMA Glyceraldehyde 3-phosphate dehydrogenase(GAPDH) (Major larval surface antigen) (P-37). [Blood fluke] {Schistosoma mansoni}, partial (37%)
6927	catatttgtt	1				no_annot
6928	agagccgaag	1				no_annot
6929	ttttgactta	1				no_annot

6930	tgatctagtt	1				no_annot
6931	aataagatac	1				no_annot
6932	acactctatc	1	CD091240	92	0	
6933	gtgtaaagaa	1				no_annot
6934	cgaataatat	1				no_annot
6935	aagagagcat	1				no_annot
6936	cagaagattc	1				no_annot
6937	gattacaaaa	1				no_annot
6938	gcgtgtcgaa	1				no_annot
6939	ttcatatata	1	TC7424	95	0	similar to GP|7294506|gb|AAF49848.1||AE003539 CG11274-PA {Drosophila melanogaster}, partial (6%)
6940	tgacagttct	1				no_annot
6941	ctggtcacac	1				no_annot
6942	ttttgtgatt	1				no_annot
6943	atactgtacc	1	TC14868	48	0	
6944	tgttcgttct	1				no_annot
6945	tgtgctgtca	1				no_annot
6946	caaccacaaa	1	TC14672	60	3	
6947	tttccagtag	1				no_annot
6948	cgttgaaatc	1				no_annot
6949	gaaatgaacc	1				no_annot
6950	aaaccgctct	1				no_annot
6951	ttcaacaatg	1				no_annot
6952	aattgaaccc	1				no_annot
6953	tttcccgaga	1				no_annot
6954	cttgggcgtc	1				no_annot
6955	tcattttaat	1				no_annot
6956	tccgcgtaca	1				no_annot
6957	tagaacgttt	1				no_annot
6958	atcttgtaag	1	C300754.1	45	0	
6959	ccattttact	1				no_annot

6960	cacacgaaat	1				no_annot
6961	cagtttttgt	1	TC12025	3	3	
6962	ccagctgcca	1				no_annot
6963	gctcaacagt	1				no_annot
6964	ccgattaaat	1				no_annot
6965	acattaagaa	1				no_annot
6966	ataataatca	1	TC11861	7	2	weakly similar to GP|2246667|gb|AAB62703.1||AF006990 alpha-adaptin C {Mus musculus}, partial (5%)
6966	ataataatca	1	CD187111	84	0	
6967	ggaaccagaa	1				no_annot
6968	atttcgttgt	1				no_annot
6969	tatcggtacc	1				no_annot
6970	cattattgac	1	TC15047	15	2	
6971	cacatatcag	1	TC14779	66	1	similar to GP|20152021|gb|AAM11370.1||AY095042 LD29234p {Drosophila melanogaster}, partial (18%)
6972	tgagctaggc	1				no_annot
6973	ccgattaaaa	1				no_annot
6974	gtagaagcct	1				no_annot
6975	tttcacaccc	1				no_annot
6976	atcagactcc	1	BG931922	21	0	homologue to GP|5597017|gb|AAC24982.2||AF025672 reverse transcriptase {synthetic construct}, partial (9%)
6977	tggacctgtc	1				no_annot
6978	tatagctatg	1	TC19611	10	1	
6979	atatcgctgt	1				no_annot
6980	gagttgtttg	1				no_annot
6981	tttcatactt	1	TC16548	89	1	weakly similar to GP|8515718|gb|AAF76141.1||AF257659 crocalbin-like protein {Homo sapiens}, partial (61%)
6982	caattgaacc	1				no_annot
6983	aattatcata	1				no_annot
6984	tataagtcaa	1	CD066957	45	1	
6985	ataatcaagt	1				no_annot

6986	ctcaacaatg	1				no_annot
6987	gatcagatcg	1				no_annot
6988	tagatagtgc	1				no_annot
6989	acaagtacca	1				no_annot
6990	tcaatagaca	1				no_annot
6991	ccattttaat	1				no_annot
6992	atgtaacgaa	1				no_annot
6993	aatcacagct	1				no_annot
6994	agttggattc	1	CD160384	84	0	
6995	cttgtcgtta	1				no_annot
6996	tacgagactt	1				no_annot
6997	gggatagata	1				no_annot
6998	taaaggggag	1	CD090988	53	0	
6999	aaaatgcact	1	TC17291	30	3	weakly similar to GP|22324559|gb|AAM95612.1||AF520952 PHD protein Jade-1 {Homo sapiens}, partial (21%)
7000	tttatagaaa	1	TC17757	17	1	
7001	acactgtgtt	1				no_annot
7002	ccctttcttt	1	TC15333	67	1	similar to PIR|A40438|A40438 GTP-binding protein arl (arf-like) - fruit fly (Drosophila melanogaster), partial (98%)
7003	gagaggataa	1				no_annot
7004	tgaatcaaat	1	TC7878	54	0	
7005	taggatgaat	1				no_annot
7006	tcacatactt	1				no_annot
7007	ccaccagtag	1	C201468.1	51	1	
7007	ccaccagtag	1	C208823.1	85	0	
7007	ccaccagtag	1	C603782.1	65	2	
7008	gtgcagaggt	1				no_annot
7009	gaacaagcga	1				no_annot
7010	ggaagagaca	1				no_annot
7011	tgaatcaaaa	1				no_annot

7012	ttgctcaatc	1				no_annot
7013	aatctatgat	1				no_annot
7014	attctattgt	1	TC13717	60	3	weakly similar to SP|Q9UK97|FBX9_HUMAN F-box only protein 9. [Human] {Homo sapiens}, partial (16%)
7015	atgttggctt	1	AF051138|Sma.1083	37	0	Trispanning orphan receptor
7016	aggattgtgt	1	TC9879	53	0	similar to GP|7303136|gb|AAF58201.1||AE003813 CG10202-PA {Drosophila melanogaster}, partial (3%)
7017	tcactatcac	1				no_annot
7018	tagtagtaac	1	TC18807	63	0	
7019	gctcggggaa	1				no_annot
7020	cagaatcggt	1				no_annot
7021	accgacttgt	1				no_annot
7022	agtaatggaa	1				no_annot
7023	gttactgcgc	1				no_annot
7024	atgttggcta	1	CD202043	46	1	weakly similar to GP|21411199|gb|AAH32923.1||BC032923 Abcf3 protein {Mus musculus}, partial (17%)
7025	tcgtcttgca	1	AF195529|Sma.951	76	0	14-3-3 epsilon
7026	ctctatgcgt	1				no_annot
7027	ttccccaagg	1				no_annot
7028	tagctaagga	1				no_annot
7029	cagtggatag	1				no_annot
7030	atgtgtaaag	1				no_annot
7031	atcgtccaaa	1				no_annot
7032	acgaagctta	1				no_annot
7033	ataatgtgtg	1				no_annot
7034	tctggattcg	1	TC15304	84	0	weakly similar to PIR|S39295|S39295 beta-adaptin 1 - fruit fly (Drosophila melanogaster), partial (7%)
7035	tcctaaagga	1				no_annot
7036	gaatcctaaa	1	TC6959	72	1	
7036	gaatcctaaa	1	CD067244	15	2	
7036	gaatcctaaa	1	CD153144	25	2	

7037	ttaaaatgtt	1	AI561387	32	1	
7038	ttcgtcatta	1				no_annot
7039	acatttacgc	1				no_annot
7040	aaacgtttta	1				no_annot
7041	aatagggatc	1				no_annot
7042	gatccctttt	1				no_annot
7043	aaaatgcaaa	1				no_annot
7044	gagtgtcatt	1				no_annot
7045	tagtgtgttg	1				no_annot
7046	tcaatgccta	1	CD061081	70	0	
7047	taagttgagt	1				no_annot
7048	tacgaaaatt	1				no_annot
7049	ccaatcttcg	1				no_annot
7050	gaaatcgaac	1				no_annot
7051	ccgtgacacg	1	C209409.1	16	1	
7051	ccgtgacacg	1	C601061.1	14	1	
7052	tagtgtgtta	1	TC13802	56	6	weakly similar to SP|Q9DCD0|6PGD_MOUSE 6-phosphogluconate dehydrogenase, decarboxylating. [Mouse] {Mus musculus}, partial (91%)
7052	tagtgtgtta	1	CD167034	8	1	
7053	ttcttttggt	1	TC18092	30	0	weakly similar to GP|29469993|gb|AAO74601.1||AY233002 choline transporter-like protein 2 {Cavia porcellus}, partial (21%)
7054	gccactgatc	1				no_annot
7055	tgatgtttta	1	TC14678	43	0	
7055	tgatgtttta	1	AI395626	1	3	
7056	gatagacggg	1				no_annot
7057	gtgtctgagt	1				no_annot
7058	gaggtccttg	1				no_annot
7059	cacaactcgt	1				no_annot
7060	tttaaagtcg	1				no_annot
7061	caatttgatt	1				no_annot

7062	acaatagctt	1				no_annot
7063	tttgttcgca	1				no_annot
7064	ttggtctccc	1				no_annot
7065	gaatatgaaa	1				no_annot
7066	tatataccaa	1	CD145718	42	3	similar to GP|1553007|gb|AAB08699.1||U67836 TipA {Dictyostelium discoideum}, partial (3%)
7067	ttctatgcct	1				no_annot
7068	cagatgaagt	1				no_annot
7069	gtgtctgagg	1				no_annot
7070	atccaaaatc	1	TC9675	35	3	
7071	cctttaccga	1				no_annot
7072	aaagtatata	1				no_annot
7073	agtgcgtgtg	1				no_annot
7074	aaagacaatt	1				no_annot
7075	gtgtctgaga	1				no_annot
7076	ttctcttctg	1				no_annot
7077	gaactatgga	1				no_annot
7078	ggggggggga	1				no_annot
7079	actattcgga	1				no_annot
7080	ggcgccgttg	1				no_annot
7081	atgattgcat	1	C304550.1	28	0	
7082	aaacgtatgg	1				no_annot
7083	tgagctagaa	1	TC14116	10	2	weakly similar to PIR|T41173|T41173 phosphomannomutase homolog - fission yeast (Schizosaccharomyces pombe), partial (22%)
7083	tgagctagaa	1	TC14117	67	0	weakly similar to GP|7303944|gb|AAF58988.1||AE003834 CG8073-PA {Drosophila melanogaster}, partial (22%)
7084	ccctaaagga	1				no_annot
7085	cataaataaa	1	TC13012	66	2	
7085	cataaataaa	1	CD112129	52	1	
7086	tagtcgagag	1	CD062893	33	0	

7087	aattgtgtac	1				no_annot
7088	ataacgactt	1				no_annot
7089	attttatgct	1				no_annot
7090	aagcatccag	1				no_annot
7091	ccgaggaact	1				no_annot
7092	cgcgacctaa	1				no_annot
7093	ttcataaaaa	1				no_annot
7094	ggattcttgg	1				no_annot
7095	atgattgcaa	1				no_annot
7096	aatactattc	1				no_annot
7097	tgatgtatga	1	C200887.1	69	4	
7097	tgatgtatga	1	C610802.1	60	4	
7098	atctgcactg	1				no_annot
7099	cataaacgtt	1				no_annot
7100	tttttgattg	1	TC11195	97	0	PIR|H72173|H72173 D5L protein - variola minor virus (strain Garcia-1966), partial (16%)
7101	cgaacaaata	1				no_annot
7102	caaaaacaaa	1	TC8977	81	0	
7103	acatcaacga	1				no_annot
7104	tttacaatga	1	TC19741	24	2	
7105	tcaatacata	1	TC11752	64	0	
7106	gttaggtaac	1				no_annot
7107	acaagttcgt	1				no_annot
7108	tcggcagatg	1				no_annot
7109	attatcagtc	1	TC17106	65	0	
7110	tactgttcga	1				no_annot
7111	tcttcctcgt	1				no_annot
7112	tctcagaaac	1				no_annot
7113	ggtcctaggt	1	CD181147	47	0	
7114	aaccctttta	1				no_annot
7115	attgattgtc	1				no_annot

7116	agttaagtag	1	C202220.1	79	0	
7116	agttaagtag	1	C604744.1	88	0	
7117	caatcagatg	1				no_annot
7118	tatgtgagag	1				no_annot
7119	atgagcatag	1				no_annot
7120	tcattgatta	1				no_annot
7121	ttagagactg	1				no_annot
7122	tggttcatca	1	CD158164	26	1	
7123	tcataacaag	1				no_annot
7124	tgcattgttt	1	AI723340	64	0	
7125	cttgttcgat	1				no_annot
7126	catacaagat	1				no_annot
7127	gtaatggtgg	1				no_annot
7128	atcatcgtgg	1				no_annot
7129	aacaggaatc	1	C604281.1	98	0	
7130	tatgtattaa	1				no_annot
7131	gggtatatct	1				no_annot
7132	tatttcaagt	1	C200529.1	68	0	
7132	tatttcaagt	1	C604622.1	66	0	
7133	aacgcttggg	1				no_annot
7134	catagattgt	1				no_annot
7135	ttccagtaca	1				no_annot
7136	ctgctatttt	1				no_annot
7137	gatatcaagt	1				no_annot
7138	tggtttattt	1	TC17684	79	1	
7138	tggtttattt	1	CD088384	27	1	weakly similar to GP|28272281|emb|CAD65194.1||AL935260 extracellular protein {Lactobacillus plantarum WCFS1}, partial (15%)
7139	tatacttctt	1	TC8354	65	0	weakly similar to GP|22946874|gb|AAG22443.2||AE003664 CG10628-PA {Drosophila melanogaster}, partial (18%)
7140	actattcgct	1				no_annot

7141	atacttgacg	1				no_annot
7142	gaatgaacaa	1	C608024.1	7	8	
7143	ctgggaaatg	1				no_annot
7144	tatttaataa	1	CD195805	66	0	
7145	catttctatg	1				no_annot
7146	aaggaatagt	1				no_annot
7147	agtatgaata	1	CD183491	52	1	
7148	taattgtaca	1				no_annot
7149	ggaattccgg	1	TC13664	71	0	weakly similar to SP|P10659|METK_YEAST S-adenosylmethionine synthetase 1(Methionine adenosyltransferase 1) (AdoMet synthetase 1). [Baker's yeast] {Saccharomyces cerevisiae}, partial (24%)
7150	catttctata	1				no_annot
7151	tttattatat	1	TC14795	72	0	similar to GP|16226907|gb|AAL16295.1|AF428365_1|AF428365 AT5g64470/T12B11_6 {Arabidopsis thaliana}, partial (5%)
7152	gatgattagg	1	TC7729	40	7	similar to GP|19527995|gb|AAL90112.1||AY089374 AT19436p {Drosophila melanogaster}, partial (7%)
7152	gatgattagg	1	TC7730	41	3	weakly similar to SP|O60930|RNH1_HUMAN Ribonuclease H1(RNase H1) (Ribonuclease H type II). [Human] {Homo sapiens}, partial (14%)
7153	actgtccaca	1				no_annot
7154	acgcataaga	1				no_annot
7155	cttattatct	1	TC17322	91	0	similar to GP|4322936|gb|AAD16137.1||AF096300 HPK/GCK-like kinase HGK {Homo sapiens}, partial (3%)
7156	ggatccaatg	1				no_annot
7157	ttcaactata	1				no_annot
7158	atgattcctt	1				no_annot
7159	aagtaaaagt	1	C207822.1	48	3	
7159	aagtaaaagt	1	C600502.1	44	3	
7160	ttaaaaagct	1				no_annot
7161	tttggtcatt	1				no_annot
7162	atactactac	1				no_annot

7163	tgttgcgcga	1	TC8488	13	1	
7164	gatcccatct	1				no_annot
7165	gtttccttta	1				no_annot
7166	aacatttatt	1				no_annot
7167	gatgcttgtt	1	TC10786	45	6	weakly similar to GP|17940124|gb|AAL49499.1|AF408422_1|AF408422 beta-catenin {Platynereis dumerilii}, partial (54%)
7168	cggttcatcg	1				no_annot
7169	aaaggttgat	1	C602575.1	46	3	
7170	tacaaatggg	1				no_annot
7171	gacgactgaa	1				no_annot
7172	ggtgcggtgc	1				no_annot
7173	tgagcttggg	1	C309631.1	70	0	
7174	gtcatttgat	1				no_annot
7175	gtcctgatga	1				no_annot
7176	aatatactcg	1				no_annot
7177	actggaagtc	1	CD162683	21	0	
7178	tatgttggtg	1	TC7127	12	3	homologue to GP|6478621|gb|AAF13926.1|AF195870_1|AF195870 brain tumor {Drosophila melanogaster}, partial (3%)
7178	tatgttggtg	1	TC7945	4	3	weakly similar to SP|P52735|VAV2_HUMAN Vav-2 protein. [Human] {Homo sapiens}, partial (3%)
7179	gatttacggt	1				no_annot
7180	ttcgcttcca	1				no_annot
7181	atacttgaaa	1				no_annot
7182	tttgtaactt	1				no_annot
7183	tgaggtttat	1				no_annot
7184	tagatggggg	1				no_annot
7185	agtggtgttg	1				no_annot
7186	aattgatcag	1				no_annot
7187	gctgacatcc	1				no_annot
7188	actattcgaa	1	C611003.1	4	6	

7189	aataaaacct	1	AI975262	8	2	
7190	tatttgtgtc	1				no_annot
7191	tctgtcattc	1	CD098057	12	1	similar to SP|P59032|RR8_CHAGL Chloroplast 30S ribosomal protein S8. {Chaetosphaeridium globosum}, partial (11%)
7192	cagtccggag	1	TC16831	47	1	weakly similar to GP|7161181|emb|CAB76563.1||AJ276003 GAR1 protein {Homo sapiens}, partial (15%)
7193	tactgttcca	1				no_annot
7194	ataaatattc	1				no_annot
7195	tttgtaacta	1				no_annot
7196	tacaagcact	1				no_annot
7197	atgtgttact	1	TC8303	31	1	
7198	actgatgtac	1				no_annot
7199	ggacatcaaa	1				no_annot
7200	atggccaatc	1				no_annot
7201	aacattaagt	1	CD072733	3	1	
7202	gttattcggg	1				no_annot
7203	gaatgtgtca	1				no_annot
7204	gagaggttag	1	CD097562	40	1	weakly similar to GP|10039641|gb|AAG12204.1|AF287482_5|AF287482 Orf122 {Chlorobium tepidum}, partial (22%)
7205	cagcccgtaa	1				no_annot
7206	tagtaactaa	1				no_annot
7207	aaacttccca	1	TC8651	62	0	
7208	aatcgaaaca	1				no_annot
7209	gaggttgtgg	1				no_annot
7210	ccacaaagag	1	TC19235	73	0	
7211	tagcgaaatc	1				no_annot
7212	gagttgttgt	1				no_annot
7213	gcattgtact	1				no_annot
7214	cctggttatg	1				no_annot
7215	taaagtgaca	1				no_annot

7216	gtttagttgg	1				no_annot
7217	ccggacgtaa	1				no_annot
7218	agtgaactgt	1				no_annot
7219	ggtgcagctt	1				no_annot
7220	gattagtgaa	1				no_annot
7221	aatattgaat	1	TC11507	91	0	
7221	aatattgaat	1	CD169561	32	1	
7222	aattatcagc	1				no_annot
7223	catactacgg	1	TC8435	77	1	similar to SP|Q9QZB7|ARPB_MOUSE Actin-related protein 11. [Mouse] {Mus musculus}, partial (5%)
7224	gatgattaca	1	C202177.1	12	6	
7225	tttgtgatat	1				no_annot
7226	agagctgacc	1				no_annot
7227	tcaacaatat	1	TC12308	2	2	
7227	tcaacaatat	1	TC14718	68	0	weakly similar to PIR|S35491|S35491 rap30 protein - rat, partial (51%)
7228	tcttgcgggt	1	CD115520	60	0	
7229	agacaaataa	1	TC7990	43	0	
7230	gattataaca	1				no_annot
7231	agttggatga	1				no_annot
7232	tatatttgat	1				no_annot
7233	tttattttgt	1				no_annot
7234	gttgcagacg	1				no_annot
7235	gcagatgtgc	1	TC8070	41	1	weakly similar to GP|19354431|gb|AAH24401.1||BC024401 LOC232210 protein {Mus musculus}, partial (9%)
7236	acgacgttca	1				no_annot
7237	ttgtctgttt	1	TC16109	61	1	weakly similar to SP|O84439|GLYA_CHLTR Serine hydroxymethyltransferase(Serine methylase) (SHMT). {Chlamydia trachomatis}, partial (4%)
7238	ggcagcacgt	1	CD190167	62	1	weakly similar to GP|2745838|gb|AAB94760.1||AF039202 Hsp70/Hsp90 organizing protein; hop {Cricetulus griseus}, partial (21%)

7239	tcaacaataa	1				no_annot
7240	tattttaata	1	TC10994	37	0	
7240	tattttaata	1	TC11589	84	0	
7241	ttaacgtaat	1				no_annot
7242	taatctattc	1				no_annot
7243	ttatgttcga	1				no_annot
7244	aatcactgac	1				no_annot
7245	gaaatactgt	1				no_annot
7246	tgcaacactt	1				no_annot
7247	attacggatc	1				no_annot
7248	gatattaatc	1				no_annot
7249	gtaggaaact	1				no_annot
7250	cagtcctaaa	1				no_annot
7251	cattactttc	1				no_annot
7252	caaagtgact	1				no_annot
7253	taagcgccta	1				no_annot
7254	ggtagtgctt	1	TC8283	29	0	
7255	atctatgtat	1				no_annot
7256	aattcccgat	1	C706685.1	31	0	
7257	aacttgaatt	1				no_annot
7258	ggaatagttc	1				no_annot
7259	tgacgggaac	1	TC7263	18	4	similar to PIR|T02995|T02995 unspecific monooxygenase- common tobacco, partial (18%)
7260	actagtcata	1				no_annot
7261	gttcgttcct	1				no_annot
7262	tacagagccg	1				no_annot
7263	ccttatgcct	1				no_annot
7264	aggagagctg	1	TC7609	60	2	
7265	aggagggtct	1				no_annot
7266	aacacaaagg	1	C209920.1	55	4	
7266	aacacaaagg	1	C606720.1	55	4	

7267	gaatcttagc	1				no_annot
7268	aaatgttggt	1	CD072906	29	0	
7269	tacctggctt	1				no_annot
7270	tcgtacgtgt	1				no_annot
7271	gagcgctgct	1				no_annot
7272	cattttaatt	1				no_annot
7273	tttcttctct	1				no_annot
7274	aaagaaatct	1				no_annot
7275	aaaaattgtt	1	CD136156	67	0	
7276	tcaattttga	1				no_annot
7277	aagctggcag	1				no_annot
7278	tttgaggatg	1				no_annot
7279	gaaaaaacga	1	CD095269	94	0	weakly similar to SP|O95847|UCP4_HUMAN Mitochondrial uncoupling protein 4 (UCP 4). [Human] {Homo sapiens}, partial (9%)
7280	cacaaatgcc	1				no_annot
7281	gtacatattt	1	TC17215	44	1	weakly similar to GP|20380650|gb|AAH27575.1||BC027575 tRNA splicing 2' phosphotransferase 1 {Mus musculus}, partial (20%)
7281	gtacatattt	1	CD066385	86	0	
7282	ataatgtgga	1	TC10435	35	6	similar to GP|7294919|gb|AAF50249.1||AE003551 CG3395-PA {Drosophila melanogaster}, partial (87%)
7283	ccttgattct	1	TC16876	83	5	
7284	gttgcttttc	1				no_annot
7285	ctcgtcttta	1				no_annot
7286	gtttagttct	1	C210955.1	12	1	
7286	gtttagttct	1	C600230.1	12	1	
7286	gtttagttct	1	C706721.1	50	0	
7287	caatagttca	1				no_annot
7288	cgtgtaattc	1				no_annot
7289	ggtaggtcgt	1				no_annot
7290	gttgttcatt	1	TC9759	87	0	

7291	aatgtctctg	1	TC10602	1	6	similar to GP|5679074|gb|AAD46846.1|AF160906_1|AF160906 BcDNA.LD02793 {Drosophila melanogaster}, partial (73%)
7292	tttgttgttg	1				no_annot
7293	ataacaggtt	1				no_annot
7294	gtatggtctt	1	TC18284	78	0	weakly similar to GP|4519431|dbj|BAA75628.1||AB011472 CDC23 {Homo sapiens}, partial (31%)
7295	atggaagtca	1				no_annot
7296	attttttccg	1				no_annot
7297	atgctggata	1				no_annot
7298	ttccgatctt	1				no_annot
7299	taacaaaatg	1				no_annot
7300	cgcagtcagg	1				no_annot
7301	gctgaagagc	1	TC15335	75	0	similar to GP|13358802|dbj|BAB33147.1||AB047823 O-sialoglycoprotein endopeptidase {Homo sapiens}, partial (78%)
7302	aatacttttg	1				no_annot
7303	tatagctaca	1	TC11244	72	0	
7304	tgaaattttt	1				no_annot
7305	ctcatatacc	1	TC16477	64	1	
7305	ctcatatacc	1	N21939	71	1	
7306	atgagcttca	1	TC15390	75	0	
7307	tgttgagggc	1				no_annot
7308	aaaagtgctg	1	TC17577	82	0	similar to GP|29028770|gb|AAO64764.1||BT005829 At2g35930 {Arabidopsis thaliana}, partial (6%)
7309	tcgaaacaag	1				no_annot
7310	tgttaaccga	1				no_annot
7311	tctaaaatcg	1				no_annot
7312	atgtcaatta	1	C308862.1	51	0	
7313	aattatcacc	1	TC11646	66	2	similar to GP|22831636|gb|AAF45888.2||AE003428 CG13316-PB {Drosophila melanogaster}, partial (3%)
7314	ctcggttagt	1				no_annot

7315	tttttgtttc	1				no_annot
7316	gttcgttcaa	1				no_annot
7317	ttcaacaaaa	1				no_annot
7318	cataatcctc	1				no_annot
7319	gagactaacg	1				no_annot
7320	tggtggattt	1	C204524.1	83	0	
7321	ttctcttcgt	1				no_annot
7322	gagactaaca	1				no_annot
7323	ttattgcttt	1	TC11868	17	4	
7324	tacacactaa	1				no_annot
7325	cacaaatgat	1				no_annot
7326	aaagacaagt	1	TC13879	5	9	weakly similar to GP|7295350|gb|AAF50668.1||AE003563 CG10173-PA {Drosophila melanogaster}, partial (9%)
7327	tttattttct	1	TC8964	30	3	
7327	tttattttct	1	TC11251	43	3	
7327	tttattttct	1	TC14295	90	0	similar to GP|18447473|gb|AAL68299.1||AY075489 RE41712p {Drosophila melanogaster}, partial (28%)
7327	tttattttct	1	TC19692	83	1	
7328	aagtagtcga	1	TC7468	6	6	weakly similar to GP|16974629|gb|AAL31217.1||AY060592 At1g72730/F28P22_8 {Arabidopsis thaliana}, partial (78%)
7329	tagctatgca	1				no_annot
7330	ttgagatgat	1	TC12461	95	0	similar to GP|19715609|gb|AAL91627.1||AY075613 AT3g59630/T16L24_180 {Arabidopsis thaliana}, partial (5%)
7331	gattatggga	1				no_annot
7332	tacccttcat	1				no_annot
7333	ccaattttgc	1	C610174.1	37	3	
7334	tttaccaaat	1	BF936035	7	1	
7335	acagaaccac	1				no_annot

7336	accctgttca	1	TC14760	54	1	weakly similar to GP|500677|gb|AAB68410.1|AAB68410|U10398 Yhr122wp {Saccharomyces cerevisiae}, partial (28%)
7337	gtgaactgtc	1				no_annot
7338	taaacacgac	1				no_annot
7339	aagcaagtgg	1				no_annot
7340	tatgattttt	1				no_annot
7341	ttgcacttta	1				no_annot
7342	gggaccaata	1				no_annot
7343	aggacgtagt	1				no_annot
7344	aatatccaaa	1				no_annot
7345	cttcgctgtc	1	CD159614	62	0	weakly similar to SP|O57478|HEMZ_XENLA Ferrochelatase, mitochondrial precursor(Protoheme ferro-lyase) (Heme synthetase). [African clawed frog] {Xenopus laevis}, partial (17%)
7346	tggacctgac	1				no_annot
7347	ttggtttcgg	1				no_annot
7348	atggacgagg	1				no_annot
7349	aaggcaagaa	1				no_annot
7350	atggcaatga	1				no_annot
7351	gtggaaattt	1				no_annot
7352	ttcagcttta	1	CD096879	15	2	similar to SP|Q14562|DDX8_HUMAN ATP-dependent helicase DDX8 (RNA helicase HRH1) (DEAH-box protein 8). [Human] {Homo sapiens}, partial (6%)
7353	tatatcatta	1	TC14155	95	0	weakly similar to GP|19344076|gb|AAH25628.1||BC025628 expressed sequence AA420417 {Mus musculus}, partial (23%)
7353	tatatcatta	1	CD075716	1	0	
7354	tatagctaaa	1				no_annot
7355	tatgtgtgaa	1				no_annot
7356	aagttttgta	1				no_annot
7357	agacataatc	1	CD116229	8	0	
7358	accatcttta	1				no_annot
7359	acatactgcg	1				no_annot

7360	ataatccgta	1	C200945.1	6	6	
7360	ataatccgta	1	C607639.1	4	11	
7361	aattatcaaa	1	C602648.1	7	3	
7362	cacacactat	1				no_annot
7363	taaggggaaa	1	TC7847	66	1	
7363	taaggggaaa	1	CD119787	23	1	
7364	cgaaaagaaa	1				no_annot
7365	taatccttgt	1				no_annot
7366	tttttgatga	1	TC16747	43	2	similar to SP|O01666|ATPG_DROME ATP synthase gamma chain, mitochondrial precursor. [Fruit fly] {Drosophila melanogaster}, partial (50%)
7366	tttttgatga	1	TC16748	88	0	similar to SP|O01666|ATPG_DROME ATP synthase gamma chain, mitochondrial precursor. [Fruit fly] {Drosophila melanogaster}, partial (23%)
7366	tttttgatga	1	TC16750	87	0	similar to SP|O01666|ATPG_DROME ATP synthase gamma chain, mitochondrial precursor. [Fruit fly] {Drosophila melanogaster}, partial (24%)
7367	gcaattcgac	1				no_annot
7368	cttttgtttc	1				no_annot
7369	gtcaaacgag	1	TC7495	43	1	weakly similar to GP|2696611|dbj|BAA23885.1||D78303 RNA splicing-related protein {Rattus norvegicus}, partial (16%)
7370	tatgttaaca	1				no_annot
7371	atttgaaaag	1	C315433.1	38	1	
7372	acacacttca	1				no_annot
7373	gcgtgaacca	1				no_annot
7374	aaccgcacaa	1	TC11855	2	6	weakly similar to SP|P23798|ME18_MOUSE DNA-binding protein Mel-18. [Mouse] {Mus musculus}, partial (17%)
7375	taataaaccc	1				no_annot
7376	ctttttcgac	1	TC7586	35	1	
7377	ttgtacaggc	1				no_annot
7378	tgtccaaaaa	1	gi|11387388|gb|L04480.2|SCMHEXK	17	4	Schistosoma mansoni hexokinase gene, complete cds
7379	taaataactg	1	CD074743	6	4	

7380	cctatccctt	1				no_annot
7381	ggatcctatc	1				no_annot
7382	gcgtcgtgat	1	AI975874	73	1	homologue to GP|13676650|gb|AAK38216.1|AF319782_10|AF319782 C2 {callitrichine herpesvirus 3} [Callitrichine herpesvirus 3], partial (5%)
7383	ctgtgaaata	1				no_annot
7384	taaatcgtgg	1				no_annot
7385	taattactag	1	TC10371	13	1	weakly similar to GP|12803373|gb|AAH02507.1|AAH02507|BC002507 WD repeat domain 13 {Homo sapiens}, partial (17%)
7386	ccagttgttc	1				no_annot
7387	attgattgga	1				no_annot
7388	tgaacaaact	1				no_annot
7389	agtgatgaag	1				no_annot
7390	ttaaataccc	1	CD141106	1	0	GP|14039811|gb|AAK53394.1||AF367371 Y-box binding protein {Schistosoma japonicum}, partial (35%)
7391	gacccaaagc	1				no_annot
7392	agacgctttt	1	TC17581	95	1	weakly similar to GP|12862302|dbj|BAB32409.1||AB047003 caspase-3 {Danio rerio}, partial (21%)
7393	aatacacata	1				no_annot
7394	tgatgacaga	1				no_annot
7395	gattcctgca	1				no_annot
7396	ttttggcgga	1				no_annot
7397	gctcgccaag	1				no_annot
7398	ccctttctaa	1				no_annot
7399	caagacctca	1				no_annot
7400	gtacaagagt	1	TC13304	79	0	
7401	tttacattct	1	C316935.1	96	0	
7402	gaaaatgtgt	1	AW017440	90	1	
7403	taaatgatca	1	C312524.1	6	1	
7403	taaatgatca	1	C604700.1	33	1	

7404	acttcttttc	1	TC7519	96	0	similar to SP|O08755|HNF6_MOUSE Hepatocyte nuclear factor 6 (HNF-6) (One cut domain family member 1). [Mouse] {Mus musculus}, partial (31%)
7405	cgcagtcaca	1				no_annot
7406	aatatgcgcc	1				no_annot
7407	tggagtatag	1				no_annot
7408	atggagaaac	1	Z34087|Sma.735	36	3	Fimbrin (FM)
7409	atagtagtga	1				no_annot
7410	gagagtgtca	1				no_annot
7411	acttccttat	1				no_annot
7412	gaaaatgtgg	1				no_annot
7413	tttagtgtta	1	C200684.1	75	1	
7413	tttagtgtta	1	C606913.1	69	1	
7414	atagtgtata	1	TC10987	16	3	weakly similar to SP|Q9DGN0|SA2_XENLA Cohesin subunit SA-2 (XSA-2) (Stromal antigen 2 homolog) (SCC3 homolog 2). [African clawed frog] {Xenopus laevis}, partial (3%)
7415	atgaattagg	1				no_annot
7416	gttggggaat	1				no_annot
7417	gtttcagaga	1	TC10776	32	5	weakly similar to GP|27374373|gb|AAO01111.1||AY190960 CG4686-PA {Drosophila pseudoobscura}, partial (29%)
7417	gtttcagaga	1	TC10777	27	3	weakly similar to GP|27374373|gb|AAO01111.1||AY190960 CG4686-PA {Drosophila pseudoobscura}, partial (23%)
7418	catttctagg	1	TC11215	96	0	weakly similar to GP|1079683|gb|AAB68306.1||U39205 Lpe13p {Saccharomyces cerevisiae}, partial (41%)
7419	gaaattgaaa	1	TC13716	57	0	similar to GP|19572637|emb|CAA49535.2||X69910 P63 protein {Homo sapiens}, partial (3%)
7420	atcaccgatt	1	C202301.1	34	0	
7420	atcaccgatt	1	C606508.1	34	0	
7421	ggatcaatag	1	TC10981	73	2	weakly similar to GP|22324206|emb|CAC82992.1||AJ344147 eIF2B-beta protein {Drosophila melanogaster}, partial (7%)
7422	aataaatcct	1				no_annot
7423	aagatgtctg	1	C605638.1	45	1	

7424	atgacttgtt	1				no_annot
7425	atggacgact	1				no_annot
7426	ccaaccaaaa	1				no_annot
7427	agttagcgtg	1				no_annot
7428	ttataaaatc	1	C209289.1	87	0	
7428	ttataaaatc	1	C609001.1	80	1	
7429	tttttcgtgt	1				no_annot
7430	taggtcactt	1				no_annot
7431	agatttctag	1	TC11885	87	0	similar to GP|14029263|gb|AAK52607.1|AF296656_1|AF296656 ubiquitin conjugating enzyme 6 {Mus musculus}, partial (30%)
7432	tgaacaaaat	1	C200223.1	74	0	
7432	tgaacaaaat	1	C314899.1	65	0	
7432	tgaacaaaat	1	C604098.1	87	0	
7432	tgaacaaaat	1	C713539.1	65	0	
7433	ttggtttccg	1				no_annot
7434	gtatctgtcc	1				no_annot
7435	tatgaacata	1	TC7672	85	0	
7436	tatcttgtta	1	TC13934	90	1	similar to PIR|C96954|C96954 permease, probable chloride channel [imported] - Clostridium acetobutylicum, partial (4%)
7437	aaggatgcat	1				no_annot
7438	gaggacacca	1				no_annot
7439	taaatgataa	1				no_annot
7440	tcccttgcac	1	TC8280	32	0	weakly similar to GP|23170838|gb|AAF54431.2||AE003684 CG9484-PA {Drosophila melanogaster}, partial (6%)
7441	aaagaattga	1	CD147478	68	1	
7442	atgctcactg	1				no_annot
7443	gcaagccaat	1				no_annot
7444	gaggcgaaat	1	TC12746	34	0	
7445	atgacctgac	1	C206893.1	81	0	

7446	tccatctcgt	1				no_annot
7447	gtagatagag	1				no_annot
7448	gtcccgaatt	1				no_annot
7449	tacgacgcag	1				no_annot
7450	ttagaaaagg	1				no_annot
7451	tgtatgctag	1				no_annot
7452	aataacctaa	1				no_annot
7453	gctttctaga	1				no_annot
7454	atctgtactg	1	TC14123	4	2	
7455	gcaataactg	1				no_annot
7456	gcactctgtt	1	TC10881	81	2	weakly similar to GP|7300794|gb|AAF55938.1||AE003736 CG6690-PA {Drosophila melanogaster}, partial (12%)
7457	tgctcacagg	1	CD191973	61	0	weakly similar to SP|O00764|PDXK_HUMAN Pyridoxal kinase(Pyridoxine kinase). [Human] {Homo sapiens}, partial (31%)
7458	taacaaggtt	1	CD075781	37	0	
7459	tgatgacacc	1	CD168010	64	2	homologue to GP|17066106|emb|CAD12457.1||AJ277892 Novex-3 Titin Isoform {Homo sapiens}, partial (4%)
7460	aacaggaact	1	TC17038	77	0	weakly similar to PIR|T39903|T39903 serine-rich protein - fission yeast (Schizosaccharomyces pombe), partial (5%)
7460	aacaggaact	1	TC17039	28	2	
7461	ttggtttcaa	1	Z29959|Sma.25	48	1	Zinc finger protein (putative)
7462	atgaatggaa	1	TC8334	42	2	
7463	tttgtgttac	1	TC8095	56	1	
7463	tttgtgttac	1	TC9146	13	3	similar to PIR|JC5308|JC5308 testis-specific, vespid, and pathogenesis-related protein 1 precursor - human, partial (10%)
7464	tacaattcca	1				no_annot
7465	tagattgaga	1				no_annot
7466	gataacttgt	1				no_annot
7467	ccaccactaa	1				no_annot
7468	tcctgaccta	1	CD158757	5	0	

7469	aatactataa	1				no_annot
7470	attccaaaaa	1				no_annot
7471	tcgcgttcct	1				no_annot
7472	taaattgttt	1	C200625.1	90	1	
7472	taaattgttt	1	C602715.1	91	1	
7473	ttcttctttt	1				no_annot
7474	aagatatatg	1				no_annot
7475	atccagtcgt	1				no_annot
7476	tctaacaaag	1				no_annot
7477	tgttgataat	1	TC9016	37	2	weakly similar to PIR|T29144|T29144 partial CDS - Caenorhabditis elegans, partial (4%)
7477	tgttgataat	1	TC17958	31	0	weakly similar to GP|16767916|gb|AAL28176.1||AY060628 GH04826p {Drosophila melanogaster}, partial (11%)
7478	aatcgataaa	1	TC18190	54	0	
7479	aggttcagtt	1				no_annot
7480	gaaaatgtcc	1				no_annot
7481	atgagtatat	1	CD111760	1	1	similar to GP|148017|gb|AAA24682.1||M37393 transposition protein D {Escherichia coli}, partial (54%)
7482	atgaattaca	1	CD184175	31	0	similar to PIR|T39903|T39903 serine-rich protein - fission yeast (Schizosaccharomyces pombe), partial (4%)
7483	atacgtacta	1				no_annot
7484	taatgaagtg	1				no_annot
7485	atccagtcga	1	C201417.1	31	3	
7485	atccagtcga	1	C202886.1	13	3	
7485	atccagtcga	1	C309293.1	60	0	
7485	atccagtcga	1	C314729.1	11	1	
7485	atccagtcga	1	C318055.1	55	0	
7486	ccttgccgtt	1				no_annot
7487	atctctttgc	1	TC8729	84	0	
7488	gtatactgcg	1				no_annot
7489	tagcctgtcc	1				no_annot

7490	gacaagacga	1				no_annot
7491	tggatgaata	1				no_annot
7492	cttatacaag	1				no_annot
7493	tgacctttca	1				no_annot
7494	aaatgcattc	1				no_annot
7495	aaagagagct	1				no_annot
7496	aaagacgggg	1				no_annot
7497	tacgtttgta	1				no_annot
7498	aacattggaa	1				no_annot
7499	gtaatctgtc	1				no_annot
7500	gacattagtt	1				no_annot
7501	ctgatcagtc	1	TC8521	11	2	
7502	actgttcact	1				no_annot
7503	cgcggtacct	1				no_annot
7504	tcattgatac	1				no_annot
7505	atagtagtag	1				no_annot
7506	tcctctcata	1				no_annot
7507	gcaatgatac	1	CD117258	57	1	
7508	actacgaata	1				no_annot
7509	tcgttactgt	1				no_annot
7510	catttttata	1	C610987.1	40	3	
7511	cggtacgagg	1				no_annot
7512	atccgaatga	1				no_annot
7513	gttatcgtca	1				no_annot
7514	atagatcgac	1	C201026.1	4	4	
7514	atagatcgac	1	C608652.1	4	4	
7515	tcgagtgaat	1	TC13947	22	3	similar to GP|17945957|gb|AAL49023.1||AY071401 RE48767p {Drosophila melanogaster}, partial (26%)
7516	tttacctacg	1	TC14252	19	0	weakly similar to GP|7290790|gb|AAF46235.1||AE003439 CG4593-PA {Drosophila melanogaster}, partial (73%)
7517	tgcattgtag	1	AA559395	62	0	

7518	tattcatatt	1	TC17191	62	0	weakly similar to GP|17945621|gb|AAL48861.1||AY071239 RE27904p {Drosophila melanogaster}, partial (75%)
7518	tattcatatt	1	CD179175	79	0	
7519	caccgttacg	1				no_annot
7520	gaaactatga	1				no_annot
7521	tcggttcata	1				no_annot
7522	tcgttactgc	1				no_annot
7523	atttggtcag	1				no_annot
7524	gtatcatcct	1				no_annot
7525	gggcaaagaa	1				no_annot
7526	caaattgttc	1	TC17084	41	0	
7527	caaattgtta	1				no_annot
7528	gattctagcc	1				no_annot
7529	ctcaactact	1				no_annot
7530	gtttctttta	1	TC11470	95	0	weakly similar to GP|14275916|dbj|BAB58904.1||AB042241 MRG15 {Mus musculus}, partial (41%)
7531	ctcacagcca	1				no_annot
7532	tgtctttaac	1				no_annot
7533	cagcctgtcg	1				no_annot
7534	ttcattcctt	1	C610499.1	11	2	
7535	tactaaaagt	1	C302118.1	15	2	
7535	tactaaaagt	1	C709194.1	13	2	
7536	aatttctcta	1				no_annot
7537	gatacatata	1				no_annot
7538	tagattgact	1	CD085227	82	0	
7539	tttggtcaat	1	CD080577	90	0	
7540	gtttgaagtc	1				no_annot
7541	aatagatgta	1				no_annot
7542	aagtatgcgc	1				no_annot
7543	gagggcagtc	1				no_annot

7544	tttggtcaag	1	CD076728	90	0	
7545	tttttcgtac	1	C607036.1	36	0	
7546	agctcagcaa	1	M86867|Sma.743	70	1	Mansoni sytosolic Cu/Zn superoxide dismutase (SOD)
7546	agctcagcaa	1	gi|161118|gb|M97298.1|SCMSODCT	67	2	Schistosome mansoni sytosolic Cu/Zn superoxide dismutase (SOD) mRNA, complete cds
7547	ctcaattatt	1				no_annot
7548	actgttcaag	1				no_annot
7549	taatgctctg	1				no_annot
7550	gttcagacgt	1				no_annot
7551	tttgttgtgt	1	TC13237	0	1	
7552	catttccgta	1				no_annot
7553	gattctagat	1				no_annot
7554	gtttatgtat	1	TC10629	70	0	similar to SP|Q9W799|CPSB_XENLA Cleavage and polyadenylation specificity factor, 100 kDa subunit (CPSF 100 kDa subunit). [African clawed frog] {Xenopus laevis}, partial (21%)
7555	acaacaagtt	1				no_annot
7556	aaattgtcct	1	TC18594	13	1	
7556	aaattgtcct	1	L76110	45	0	
7557	gcaattgttc	1				no_annot
7558	taacaaaaga	1	CD139256	17	0	
7559	tcgatgatga	1	TC14127	3	4	
7560	aatactttga	1				no_annot
7561	gagtttgtaa	1	TC15811	73	0	similar to GP|11612596|gb|AAD42950.2|AF114378_1|AF114378 PDZ-LIM protein cypher1c {Mus musculus}, partial (4%)
7562	ttagaaaaaa	1	CD125666	21	1	
7563	tggagtttca	1	C209524.1	53	1	
7563	tggagtttca	1	C601070.1	56	1	
7564	aagaaatttt	1				no_annot
7565	attttaattt	1	TC13735	83	1	
7566	cctcaactac	1				no_annot

7567	attgtattta	1	TC12536	93	0	
7568	gaccgcgaga	1				no_annot
7569	tttacccgtt	1				no_annot
7570	gatacaaaga	1				no_annot
7571	caaacagttt	1				no_annot
7572	ctttttgttc	1				no_annot
7573	tacttgctac	1				no_annot
7574	gttcaatatg	1	TC10056	82	0	
7575	ttccagcgag	1				no_annot
7576	agtattgatg	1				no_annot
7577	actagggtta	1				no_annot
7578	cggtggtttt	1				no_annot
7579	aaccatctca	1				no_annot
7580	atcaactcta	1	TC10386	42	6	similar to GP|20177041|gb|AAM12282.1||AY095189 LD26817p {Drosophila melanogaster}, partial (30%)
7581	tctattgcca	1				no_annot
7582	cttacttatg	1				no_annot
7583	agaataagac	1				no_annot
7584	tagattgaaa	1	CD133547	35	2	
7585	acggaaatat	1				no_annot
7586	gttacagttt	1				no_annot
7587	tgaacgtcag	1	TC12136	65	0	
7588	gtacaacatt	1	TC11650	25	2	
7589	gaaagggaaa	1				no_annot
7590	tctatcccgt	1				no_annot
7591	ccaccccaac	1				no_annot
7592	gagcacaatg	1				no_annot
7593	tgcatatccc	1				no_annot
7594	actagtcact	1				no_annot
7595	acagtgagtg	1				no_annot
7596	gcatatgtag	1				no_annot

7597	gaagaggtat	1				no_annot
7598	aagcagtagt	1				no_annot
7599	atttattggt	1				no_annot
7600	gaaaatcttc	1	TC14328	45	1	weakly similar to GP|16767914|gb|AAL28175.1||AY060627 GH04687p {Drosophila melanogaster}, partial (14%)
7601	gcccttattg	1	TC14779	45	2	similar to GP|20152021|gb|AAM11370.1||AY095042 LD29234p {Drosophila melanogaster}, partial (18%)
7601	gcccttattg	1	CD136921	95	0	similar to GP|20152021|gb|AAM11370.1||AY095042 LD29234p {Drosophila melanogaster}, partial (22%)
7602	tgtatttgta	1				no_annot
7603	gttctcagag	1				no_annot
7604	ttttctccaa	1				no_annot
7605	ttaaattccc	1				no_annot
7606	ggcgagctgg	1				no_annot
7607	tacagctgaa	1				no_annot
7608	ggcgagctgc	1				no_annot
7609	agctgagagg	1	CD164815	29	4	weakly similar to GP|15293871|gb|AAK95128.1|AF401556_1|AF401556 ribosomal protein L5a {Ictalurus punctatus}, partial (42%)
7610	tgaagtgata	1				no_annot
7611	gtcaagaatt	1				no_annot
7612	acttgatgtt	1				no_annot
7613	tctaatggtt	1				no_annot
7614	cgggcattat	1				no_annot
7615	tcaaatgaaa	1				no_annot
7616	tgttattgat	1				no_annot
7617	agctgcttgt	1	C207961.1	40	0	
7618	cagaaaacat	1				no_annot
7619	ttggatagag	1				no_annot
7620	cgaaatttga	1				no_annot
7621	atattgtatg	1	AY045762|Sma.744	90	0	Ferredoxin NADP+ reductase mRNA, complete cds; nuclear gene for mitochondrial product

7622	tgatagtttt	1				no_annot
7623	acaaaaaacc	1				no_annot
7624	cggctggaaa	1				no_annot
7625	gagttatcct	1				no_annot
7626	cgtatttgtt	1				no_annot
7627	ttatttagtt	1				no_annot
7628	ttagtttgta	1				no_annot
7629	tgatagtttg	1	TC12522	36	0	
7629	tgatagtttg	1	CD080806	47	1	
7630	gcgacggcca	1				no_annot
7631	ctctgttaca	1				no_annot
7632	ctgtgaaagc	1				no_annot
7633	gtgttgtcta	1	C203463.1	95	0	
7634	aaatacaact	1	TC13789	74	0	
7634	aaatacaact	1	CD116339	66	0	
7635	ttttcgaatg	1				no_annot
7636	ctgaaaaatg	1	TC11955	15	2	
7636	ctgaaaaatg	1	TC13872	30	1	
7637	aggccttccc	1	C300775.1	94	0	
7637	aggccttccc	1	C715107.1	94	0	
7638	tttagtgtgt	1	TC8135	0	6	
7639	tgttgtgcaa	1				no_annot
7640	tgattgaacg	1				no_annot
7641	ctagaagggt	1				no_annot
7642	taacaaaaca	1	TC9860	18	1	
7643	cacagctgaa	1				no_annot
7644	actattgtgt	1	CD084404	6	1	
7645	aacaatttat	1				no_annot
7646	gcacaaaaga	1	CD125437	90	0	

7647	gcaatccttt	1	TC14760	74	0	weakly similar to GP|500677|gb|AAB68410.1|AAB68410|U10398 Yhr122wp {Saccharomyces cerevisiae}, partial (28%)
7648	aaaatcaatc	1				no_annot
7649	tttttgttcc	1				no_annot
7650	caataatcat	1				no_annot
7651	ctggatagat	1				no_annot
7652	tactatgcta	1				no_annot
7653	gcaatccttc	1				no_annot
7654	acgagatagt	1				no_annot
7655	agatattcac	1				no_annot
7656	tccaagctgc	1	TC15254	12	0	weakly similar to SP|P51951|MAT1_XENLA CDK-activating kinase assembly factor MAT1 (RING finger protein MAT1) (Menage a trois) (CDK7/cyclin H assembly factor). [African clawed frog] {Xenopus laevis}, partial (35%)
7657	gtaataccta	1				no_annot
7658	cgatagtttt	1	TC10709	11	9	similar to GP|21392552|gb|AAM51498.1|AF039717_4|AF039717 Abnormal dauer formation protein 16, isoform d {Caenorhabditis elegans}, partial (8%)
7659	ccatctccag	1				no_annot
7660	tctatcccct	1				no_annot
7661	gccagttgta	1				no_annot
7662	tcatcgaata	1				no_annot
7663	ctctgttaaa	1				no_annot
7664	cttagaacca	1				no_annot
7665	taaagtcact	1	TC9046	13	5	weakly similar to SP|P29540|T2EB_XENLA Transcription initiation factor IIE, beta subunit (TFIIE-beta). [African clawed frog] {Xenopus laevis}, partial (19%)
7665	taaagtcact	1	TC15770	84	0	similar to GP|14209854|gb|AAK56875.1|AF314019_1|AF314019 NADH dehydrogenase subunit I {Rana boylii}, partial (21%)
7666	tgtgtgtgtt	1	TC7629	2	8	similar to GP|27371005|gb|AAH40802.1||BC040802 Eprs protein {Mus musculus}, partial (33%)

7666	tgtgtgtgtt	1	TC7630	7	0	
7667	tacgaacgtg	1				no_annot
7668	tcgatgatat	1	TC19066	53	0	
7669	atcacagtaa	1				no_annot
7670	gaggttctgc	1				no_annot
7671	gatacaaaat	1	TC16413	14	3	similar to GP|18181863|emb|CAC85211.2||AJ312106 cathepsin B endopeptidase {Schistosoma mansoni}, partial (20%)
7671	gatacaaaat	1	CD188353	47	0	
7672	tcatacactg	1	AI395588	87	0	homologue to GP|5597017|gb|AAC24982.2||AF025672 reverse transcriptase {synthetic construct}, partial (6%)
7673	gtgaattgtt	1	TC18801	55	0	
7674	ataatttaac	1	TC15129	78	0	
7675	aaattacgtt	1				no_annot
7676	gtttctataa	1				no_annot
7677	tatgatttca	1				no_annot
7678	ttgttttcag	1				no_annot
7679	atgtggatga	1	TC7940	11	3	similar to GP|3641346|gb|AAC36355.1||AF091341 apoferritin-2 {Schistosoma japonicum}, partial (98%)
7680	taacacgcat	1	TC10444	69	4	
7680	taacacgcat	1	TC10449	84	3	weakly similar to GP|2636680|gb|AAC06263.1||U66331 pol {Schistosoma mansoni}, partial (3%)
7680	taacacgcat	1	CD063619	42	2	weakly similar to GP|21617886|gb|AAM66936.1||AY088613 40S ribosomal protein S5 {Arabidopsis thaliana}, partial (13%)
7681	ttgttttcaa	1	TC19240	58	0	weakly similar to GP|17945986|gb|AAL49037.1||AY071415 RE49852p {Drosophila melanogaster}, partial (33%)
7682	cattcaaacc	1				no_annot
7683	tttttgttac	1	TC7412	42	0	
7683	tttttgttac	1	TC9634	36	0	
7683	tttttgttac	1	TC17742	88	1	
7684	tagtatcaaa	1				no_annot
7685	tgaaatttaa	1				no_annot

7686	aagacggtcg	1				no_annot
7687	cagagaagtt	1	M64539lSma.624	36	3	Ferritin-2
7688	tatgcacgtg	1				no_annot
7689	agaggaccaa	1				no_annot
7690	cattttgggt	1				no_annot
7691	ttaccaattg	1	TC17218	94	0	
7692	gtttggcatt	1	C210924.1	83	0	
7693	tttctaaata	1				no_annot
7694	gcacagacag	1				no_annot
7695	aaatgctttg	1				no_annot
7696	ttgcttaacg	1				no_annot
7697	attatctgac	1	TC10621	25	2	
7698	gtgagaaaaa	1				no_annot
7699	gtcctatcgc	1				no_annot
7700	cgaacacgta	1				no_annot
7701	ttcgaagtaa	1				no_annot
7702	acacaacagc	1				no_annot
7703	gcaggaagag	1				no_annot
7704	ttcgacgaga	1	C202097.1	27	6	
7704	ttcgacgaga	1	C604216.1	27	6	
7705	ctgatctgta	1				no_annot
7706	aaagagtgcg	1				no_annot
7707	tagcaacccc	1				no_annot
7708	gatgtgaaaa	1				no_annot
7709	acttcacaga	1				no_annot
7710	ctcaataaaa	1				no_annot
7711	aagcttttat	1	TC11281	72	0	
7712	tgacgatgca	1				no_annot
7713	cgtgtgtgtg	1				no_annot
7714	tacacggttt	1				no_annot

7715	gaattcacgt	1	TC8075	44	3	
7716	caacaaaaaa	1				no_annot
7717	aaacaaactg	1	TC8818	78	0	
7718	acattcaatg	1				no_annot
7719	cgattgaaaa	1				no_annot
7720	aatctaatga	1				no_annot
7721	tactgaatga	1				no_annot
7722	aagggtaaaa	1	C200984.1	9	2	
7722	aagggtaaaa	1	C609906.1	7	5	
7723	atagacgtag	1				no_annot
7724	gtggacaaga	1				no_annot
7725	acgaaaccag	1				no_annot
7726	gctaatccaa	1	TC11343	15	1	weakly similar to SP|O43837|IDHB_HUMAN Isocitrate dehydrogenase [NAD] subunit beta, mitochondrial precursor(Isocitric dehydrogenase) (NAD+-specific ICDH). [Human] {Homo sapiens}, partial (16%)
7727	gcgaatgtca	1				no_annot
7728	taaattgtga	1				no_annot
7729	tgtaaataat	1	TC8598	96	0	
7730	aagaacaagg	1				no_annot
7731	taaataacat	1				no_annot
7732	tgtttaatga	1				no_annot
7733	taatttcaga	1				no_annot
7734	ataacataag	1	CD154752	26	0	
7735	cttactaaaa	1				no_annot
7736	tggctaagga	1				no_annot
7737	catagttaag	1				no_annot
7738	ttgtgagggc	1				no_annot
7739	actcgaatcc	1	TC14498	84	0	
7740	ctgtgaaaac	1	TC13944	75	0	similar to GP|1943772|gb|AAB52431.1||U97191 Rab family protein 2 {Caenorhabditis elegans}, partial (89%)
7741	tttgttcttt	1				no_annot

7742	taaacgggta	1				no_annot
7743	atccgcaaaa	1	TC8627	12	5	weakly similar to GP|15010482|gb|AAK77289.1||AY047557 GH07062p {Drosophila melanogaster}, partial (10%)
7744	cgattacttg	1	C201162.1	60	0	
7744	cgattacttg	1	C610878.1	58	0	
7745	gcttctgctt	1				no_annot
7746	tctacacctc	1				no_annot
7747	aaatagagat	1	CD086850	55	1	
7748	ggaatcgaaa	1				no_annot
7749	tgaatgatta	1				no_annot
7750	ctgaccactt	1	C302012.1	80	0	
7750	ctgaccactt	1	C714778.1	80	0	
7751	gcgaaatcgg	1				no_annot
7752	gcttctgctg	1				no_annot
7753	tagagcacgt	1				no_annot
7754	gaacattgat	1	CD069208	27	2	
7755	gataggaaca	1	TC10843	9	4	similar to GP|2217962|emb|CAA64439.1||X94983 GDP-dissociation inhibitor {Geodia cydonium}, partial (67%)
7756	aaactgtagc	1				no_annot
7757	cttagtgtca	1				no_annot
7758	gaagctgttg	1				no_annot
7759	cccaagctct	1				no_annot
7760	tattcccctg	1				no_annot
7761	taacacccta	1	C206861.1	49	1	
7761	taacacccta	1	C608195.1	61	1	
7762	actcgaatat	1				no_annot
7763	gaataagctc	1				no_annot
7764	caggaggtcg	1				no_annot
7765	atattgaaag	1	TC18096	43	0	
7766	tacataggtt	1				no_annot

7767	attgtgtgta	1				no_annot
7768	caaattgtga	1				no_annot
7769	gtacgactta	1	M74233lSma.65	88	1	Calcium-activated neutral proteinase (CANP)
7769	gtacgactta	1	gil160936lgblM67499.1lSCMCANPA	87	1	S.mansoni calcium-activated neutral proteinase (CANP) mRNA, complete cds
7770	ggttatggta	1				no_annot
7771	aggcaagttt	1				no_annot
7772	gacataccgg	1				no_annot
7773	tgtgcaaatt	1				no_annot
7774	cgataaactc	1	C307352.1	96	0	
7774	cgataaactc	1	C604928.1	61	3	
7775	ttcaatggct	1	C208875.1	84	0	
7776	cggctaagga	1				no_annot
7777	tcgtgggact	1				no_annot
7778	tatgtgatta	1	TC14744	67	1	
7779	ttataaaaaa	1	CD162544	23	0	similar to GPl29894057lgblAAP07349.1llAE016998 Guanine-hypoxanthine permease {Bacillus cereus ATCC 14579}, partial (10%)
7780	acaaccacaa	1				no_annot
7781	ttcacttgta	1				no_annot
7782	atgaatcggg	1				no_annot
7783	cttcctcaga	1				no_annot
7784	ggaatgggct	1				no_annot
7785	cgacgtacaa	1				no_annot
7786	gacgtgtgtt	1				no_annot
7787	catcactctc	1	C211378.1	10	0	
7787	catcactctc	1	C609526.1	15	0	
7788	gaaactttcg	1				no_annot
7789	cataacgata	1				no_annot
7790	tttactggct	1				no_annot
7791	tatccagtca	1				no_annot
7792	ttatgattta	1				no_annot

7793	taatgaagca	1	AI394900	15	0	
7794	agacattaga	1	TC8349	32	1	similar to GP|11321424|gb|AAG34162.1|AF312924_1|AF312924 Ral-A exchange factor RalGPS2 {Mus musculus}, partial (6%)
7794	agacattaga	1	TC16890	38	1	
7795	aacaccccccc	1				no_annot
7796	acattatttc	1				no_annot
7797	caacttaggg	1	TC15006	11	0	
7798	gtaaaagggt	1				no_annot
7799	catcttgtat	1				no_annot
7800	gacttattta	1				no_annot
7801	tttagatcct	1				no_annot
7802	caggtatgtg	1				no_annot
7803	ccccgtatcg	1				no_annot
7804	taccacgaga	1	TC11936	61	0	similar to PIR|T24629|T24629 glutamate synthase (NADH2)precursor [similarity] - Caenorhabditis elegans, partial (3%)
7805	taaacgaagt	1				no_annot
7806	cgatagataa	1				no_annot
7807	tccactgtcc	1				no_annot
7808	attttaatgc	1				no_annot
7809	aaactgtact	1				no_annot
7810	aagaaattga	1	CD084178	64	0	similar to PIR|T39340|T39340 deoxyhypusine synthaseSPBC1271.04c [similarity] - fission yeast (Schizosaccharomyces pombe), partial (9%)
7811	tgtgtgagag	1	CD080159	33	0	
7812	tgtatttggt	1				no_annot
7813	ggttgggcga	1				no_annot
7814	ctttaaagtg	1				no_annot
7815	aaatgtattt	1	C308078.1	84	1	
7816	tgtgatatta	1				no_annot
7817	cattggaaaa	1				no_annot

7818	attgtcaaag	1				no_annot
7819	gtgttaaaaa	1				no_annot
7820	aaactgtaca	1				no_annot
7821	cggatcgagt	1				no_annot
7822	aagatataaa	1	TC11118	65	0	weakly similar to GP|24980875|gb|AAH39758.1||BC039758 Fms interacting protein {Mus musculus}, partial (8%)
7822	aagatataaa	1	TC11119	57	0	weakly similar to GP|24980875|gb|AAH39758.1||BC039758 Fms interacting protein {Mus musculus}, partial (10%)
7823	agctgtatcc	1				no_annot
7824	gtggacaaac	1				no_annot
7825	cccgtaaact	1				no_annot
7826	tgtggagttt	1				no_annot
7827	actattcttc	1				no_annot
7828	ttcttgccta	1				no_annot
7829	aagcccggag	1				no_annot
7830	tcgggaagca	1	C600227.1	35	0	
7831	ttttaggaca	1	TC7628	70	0	similar to SP|P41091|IF2G_HUMAN Eukaryotic translation initiation factor 2 subunit 3 (Eukaryotic translation initiation factor 2 gamma subunit) (eIF-2-gamma). [Human] {Homo sapiens}, partial (93%)
7832	taaattgtaa	1	CD075942	93	0	
7833	tacaacaatc	1				no_annot
7834	caagtggtgt	1				no_annot
7835	taatcgccaa	1				no_annot
7836	tgcacgcgtg	1				no_annot
7837	gaacacattc	1				no_annot
7838	ataaaacaac	1				no_annot
7839	ataaaacaaa	1				no_annot
7840	ttgacaagtt	1				no_annot
7841	tatcagcata	1	CD097148	53	1	GP|3892187|gb|AAC78303.1||AF064592 RNA-binding protein {Schistosoma japonicum}, partial (23%)
7842	acgttgtcag	1				no_annot

7843	gctcaggaag	1				no_annot
7844	ttttttgtca	1				no_annot
7845	gcttttggag	1				no_annot
7846	caagtggtga	1				no_annot
7847	aaatattatt	1	TC8709	84	0	
7847	aaatattatt	1	CD121584	10	0	similar to GP|27692297|gb|AAH05580.2||BC005580 polymerase (RNA) II (DNA directed) polypeptide G {Mus musculus}, partial (53%)
7848	ttgacaagtg	1				no_annot
7849	tctgcaatga	1				no_annot
7850	gtaacgcata	1				no_annot
7851	atgttacaca	1				no_annot
7852	caaacgaagt	1	AI067604	80	0	
7853	acaaaataaa	1	CD191326	65	1	
7854	ggcttatgaa	1	TC10492	21	3	weakly similar to SP|P51991|ROA3_HUMAN Heterogeneous nuclear ribonucleoprotein A3 (hnRNP A3) (D10S102). [Human] {Homo sapiens}, partial (11%)
7855	gacaaataaa	1	C209729.1	46	1	
7856	gatgatgtta	1				no_annot
7857	cgtatttggt	1				no_annot
7858	gttcgaatca	1				no_annot
7859	cacatatata	1	CD197233	83	0	
7860	gagtagcagc	1				no_annot
7861	cagtctcggg	1	C201077.1	14	1	
7861	cagtctcggg	1	C201104.1	50	0	
7861	cagtctcggg	1	C202127.1	53	2	
7861	cagtctcggg	1	C601564.1	45	0	
7861	cagtctcggg	1	C605519.1	39	4	
7861	cagtctcggg	1	C708347.1	4	2	
7862	ctattttgtc	1				no_annot
7863	tatctatcat	1	TC8245	78	0	homologue to GP|2289237|gb|AAB64410.1||U95301 calcium-dependent group X phospholipase A2 {Homo sapiens}, partial (7%)

7864	tgtgttggta	1				no_annot
7865	caatttcaag	1				no_annot
7866	gagatggaac	1	CD111464	72	0	
7867	accaatgatt	1				no_annot
7868	atgatagtat	1	TC8990	90	0	similar to GP|17981443|gb|AAL51007.1|AF454432_1|AF454432 NYD-SP28 protein {Mus musculus}, partial (18%)
7869	acaataccaa	1				no_annot
7870	agagtcgcct	1				no_annot
7871	gataggtaga	1				no_annot
7872	gctgctgcat	1				no_annot
7873	aattgaaata	1	AY826981|Sma.4207	91	0	SARC
7874	ctgacaagtt	1				no_annot
7875	tcctgttggt	1				no_annot
7876	gttattgtac	1				no_annot
7877	atgatagtag	1				no_annot
7878	ccaagtcttt	1				no_annot
7879	ttgtgataat	1	TC13387	89	0	
7880	tcgggctcct	1				no_annot
7881	aagaaattcg	1				no_annot
7882	agctattaag	1	AI395121	71	0	
7883	acaaatgcgc	1				no_annot
7884	ctcgttttga	1	TC8054	87	0	similar to GP|18150102|dbj|BAB83666.1||AB065095 transmembrane protein FEND {Drosophila melanogaster}, partial (6%)
7884	ctcgttttga	1	CD078904	95	0	
7885	ccagctgatc	1				no_annot
7886	tatatttttg	1	CD080880	76	0	
7887	aatagatgaa	1				no_annot
7888	tactgcaact	1				no_annot
7889	atatcctttg	1	C201333.1	67	1	
7889	atatcctttg	1	C601605.1	26	4	

7890	accaccaagt	1				no_annot
7891	aattgcgctg	1				no_annot
7892	aaatataaga	1	TC10924	21	2	
7893	tatatgcctt	1				no_annot
7894	cagagatgta	1	TC14798	68	2	
7895	taaattcttt	1				no_annot
7896	gcttcaggag	1	TC8019	16	1	
7897	ctttaggaat	1				no_annot
7898	catatcttct	1				no_annot
7899	tggcgaaatc	1				no_annot
7900	gattagatgc	1				no_annot
7901	tatatgcctc	1				no_annot
7902	tatatgccta	1				no_annot
7903	tatatcttac	1	C307242.1	70	0	
7903	tatatcttac	1	C701108.1	70	0	
7904	ctttaggaag	1	TC11536	83	0	weakly similar to GP|12642962|gb|AAG37303.1||AY009320 tumorous imaginal discs protein Tid56-like protein long form; TID1L; mTid-1L {Mus musculus}, partial (41%)
7905	aatggaaagg	1	M74233|Sma.65	45	7	Calcium-activated neutral proteinase (CANP)
7905	aatggaaagg	1	gi|160936|gb|M67499.1|SCMCANPA	44	7	S.mansoni calcium-activated neutral proteinase (CANP) mRNA, complete cds
7906	ctacttatgt	1	CD120478	30	1	
7906	ctacttatgt	1	CD092320	30	0	
7907	tttgggctta	1				no_annot
7908	aattcatctt	1	CD188622	16	2	
7909	tcggggcagt	1				no_annot
7910	ttatgaatca	1				no_annot
7911	ggttagtgac	1				no_annot
7912	ggcaagacta	1				no_annot
7913	aggttctggt	1				no_annot
7914	tgcggaattt	1				no_annot

7915	acaagtaatg	1	TC8576	12	1	
7916	tataagaaga	1				no_annot
7917	tgaagccagg	1				no_annot
7918	agtgccgttt	1				no_annot
7919	ttcactagac	1				no_annot
7920	aagaaattac	1	CD154214	82	0	
7921	agaactgtac	1				no_annot
7922	tgcaataccg	1	CD097111	85	0	similar to GP|21105547|gb|AAM34697.1|AF508870_1|AF508870 elongation factor 1-alpha {Xyleborus annexus}, partial (12%)
7923	ttgaaaaaat	1	C211016.1	4	7	
7923	ttgaaaaaat	1	C610229.1	4	7	
7924	ttttcagtag	1				no_annot
7925	atgatgaaaa	1				no_annot
7926	ataattcggg	1				no_annot
7927	ttgcagccgg	1	TC17332	42	1	
7928	cgattgcgtg	1				no_annot
7929	tttttatcct	1				no_annot
7930	ttaatatatt	1				no_annot
7931	tccttcgcgt	1				no_annot
7932	acagtgagac	1	C210542.1	17	3	
7932	acagtgagac	1	C600194.1	16	3	
7933	aactagttag	1	TC14134	6	2	
7934	ggcctgtggt	1				no_annot
7935	gtcaccggta	1	C310793.1	91	0	
7935	gtcaccggta	1	C716468.1	91	0	
7936	atgggtgtca	1				no_annot
7937	ctgaaaaacg	1	AF217404|Sma.771	8	3	Endoplasmin
7938	tgaatgtttg	1				no_annot
7939	gatttacttc	1				no_annot
7940	ttttcgaaca	1				no_annot

7941	gccggcggat	1	TC17989	68	3	similar to GP|14517464|gb|AAK62622.1||AY039567 At1g65820/F1E22_4 {Arabidopsis thaliana}, partial (10%)
7942	aaaatcaact	1				no_annot
7943	caagtggtat	1				no_annot
7944	gatgccatca	1	TC10752	50	1	
7944	gatgccatca	1	CD141105	32	1	weakly similar to SP|Q9WU56|TRUA_MOUSE tRNA pseudouridine synthase A(Pseudouridylate synthase I) (Pseudouridine synthase I) (Uracil hydrolyase). [Mouse] {Mus musculus}, partial (17%)
7945	gatgaccttc	1				no_annot
7946	atactcagtc	1				no_annot
7947	atgtcgtggt	1				no_annot
7948	acaacttctt	1				no_annot
7949	gacatttgga	1	TC7118	36	9	weakly similar to GP|28274854|gb|AAO25692.1||AY195856 ankyrin repeat protein E4_8 {synthetic construct}, partial (28%)
7950	aaaatcaacg	1				no_annot
7951	caagtggtag	1				no_annot
7952	accagaactt	1				no_annot
7953	tagactttca	1				no_annot
7954	gcggtctcga	1	TC13641	91	0	
7955	gcaatcctct	1	TC18693	39	2	
7956	tcgattgtgt	1				no_annot
7957	cgagcagaag	1				no_annot
7958	agggaataaa	1				no_annot
7959	tacaaaatgg	1				no_annot
7960	tacataaaat	1				no_annot
7961	tacaaaatga	1				no_annot
7962	tttgagcagt	1				no_annot
7963	agatgttggg	1				no_annot
7964	aattagctgg	1				no_annot
7965	tctaattact	1				no_annot

7966	gatgagttcc	1	TC17086	80	0	
7967	gcaaccgaga	1				no_annot
7968	catattatga	1				no_annot
7969	aatccaaatg	1				no_annot
7970	tattctgcct	1				no_annot
7971	ctgcagccgg	1				no_annot
7972	tatacaatgt	1				no_annot
7973	caacgattgc	1	TC15759	96	0	weakly similar to GP|14626709|gb|AAK71636.1|AF387900_1|AF387900 protein disulfide isomerase-related protein P5 precursor {Danio rerio}, partial (17%)
7974	ttacgtcgca	1				no_annot
7975	caagtcagtg	1				no_annot
7976	ttacttatat	1	CD139927	69	0	
7977	agggtaccag	1				no_annot
7978	attctgtttg	1				no_annot
7979	gatttcgaag	1	TC12390	5	4	
7980	tctttcttac	1	C606886.1	66	2	
7981	tttttatcag	1	TC14885	70	2	
7982	ctatgcaagt	1				no_annot
7983	aaaatcaaat	1				no_annot
7984	tggtgtgtgc	1	TC10506	75	2	weakly similar to PIR|JC1308|JC1308 ribosomal protein L5 - chicken, partial (95%)
7985	tttgttctga	1	TC18802	80	0	
7986	tggggatcag	1				no_annot
7987	catacatttg	1				no_annot
7988	ttttacacat	1				no_annot
7989	cataaagtcg	1	TC16678	88	2	SP|P20287|G3P_SCHMA Glyceraldehyde 3-phosphate dehydrogenase(GAPDH) (Major larval surface antigen) (P-37). [Blood fluke] {Schistosoma mansoni}, complete
7989	cataaagtcg	1	AA917145	2	1	similar to GP|25045186|emb|CAC95192.1||AJ416904 NADH dehydrogenase subunit 6 {Schistosoma mansoni}, partial (15%)

7989	cataaagtcg	1	AI976252	64	1	SP|P20287|G3P_SCHMA Glyceraldehyde 3-phosphate dehydrogenase(GAPDH) (Major larval surface antigen) (P-37). [Blood fluke] {Schistosoma mansoni}, partial (39%)
7990	taagtgcttg	1				no_annot
7991	atgaacgtgc	1	AF521094|Sma.5608	79	0	Clone p83H3 unknown mRNA
7992	tactgattcc	1				no_annot
7993	aggtgatttg	1	TC17557	76	1	
7994	gagaacagtt	1				no_annot
7995	gtcaaacttc	1	H49031	69	0	
7996	atttacttta	1				no_annot
7997	gataaacaac	1				no_annot
7998	agcaatatgt	1				no_annot
7999	gatgaaacgg	1				no_annot
8000	accaggatat	1				no_annot
8001	tttgatacgt	1				no_annot
8002	taacgattct	1	TC12900	64	0	
8003	gtgggcatac	1				no_annot
8004	gaacgacgac	1	CD065531	25	2	
8005	cgtgaggcaa	1				no_annot
8006	ttcagtttca	1				no_annot
8007	ctaggcggcc	1				no_annot
8008	tgattactct	1				no_annot
8009	ataagccagt	1				no_annot
8010	tacccattgg	1	C606580.1	59	1	
8011	gcagtcagtt	1	CD202633	70	0	homologue to GP|1072041|gb|AAA90952.1||U29875 Flt3 ligand alternatively spliced isoform {Mus musculus}, partial (7%)
8012	tgccatccgt	1				no_annot
8013	ggccaagact	1				no_annot
8014	aagaagtggg	1				no_annot
8015	cacataaaac	1				no_annot

8016	cacataaaaa	1				no_annot
8017	aagaattatt	1	gi|11387388|gb|L04480.2|SCMHEXK	75	0	Schistosoma mansoni hexokinase gene, complete cds
8018	atttttaatt	1	C608275.1	89	0	
8019	ccattatcca	1	TC8502	65	1	
8020	aggcaagtgc	1				no_annot
8021	gaattttctg	1				no_annot
8022	gcaaaatgta	1				no_annot
8023	accttgatgt	1	C210956.1	35	3	
8023	accttgatgt	1	C612600.1	35	3	
8024	acacttcgac	1				no_annot
8025	gtccttctgg	1	C606396.1	41	1	
8026	tatccccata	1	TC11250	71	1	
8027	aatggaaaaa	1				no_annot
8028	atttttaata	1	TC15222	96	0	
8029	tacacagctc	1				no_annot
8030	gcaaacacaa	1	TC8135	19	2	
8031	atgaagatag	1				no_annot
8032	gctggactaa	1				no_annot
8033	attgtctaaa	1	TC7454	85	3	similar to GP|21392234|gb|AAM48471.1||AY118442 SD06874p {Drosophila melanogaster}, partial (78%)
8034	gaattctcag	1				no_annot
8035	gtgctgaaaa	1				no_annot
8036	atcattctac	1				no_annot
8037	catattatct	1	TC14056	69	0	similar to GP|29373127|gb|AAO72712.1||AY241394 Mn superoxide dismutase {Melopsittacus undulatus}, partial (84%)
8038	ttatgtaatt	1				no_annot
8039	cagagaagat	1				no_annot
8040	atagacctga	1				no_annot
8041	gtaacaagag	1				no_annot
8042	gttggttcca	1	TC11867	88	1	
8043	tttctaaaaa	1	TC18687	97	0	

8044	acggcctggt	1				no_annot
8045	tttctcgcat	1	CD073130	41	0	
8046	tgttacatag	1	TC10530	62	1	similar to SP|O96860|DYL2_DROME Dynein light chain 2, cytoplasmic (8 kDa dynein light chain). [Fruit fly] {Drosophila melanogaster}, partial (74%)
8047	ggcccaaagt	1				no_annot
8048	ctgtctctct	1				no_annot
8049	ctcgcgagaa	1	TC13713	74	2	similar to GP|22832584|gb|AAN09504.1||AE003512 CG14217-PD {Drosophila melanogaster}, partial (3%)
8050	tatacaatct	1				no_annot
8051	ccacaatttt	1				no_annot
8052	aaaggtttgt	1	TC13809	60	4	
8053	tataaacttt	1	TC11304	89	0	weakly similar to PIR|JC4237|JC4237 leukotriene-A4 hydrolase- guinea pig, partial (23%)
8054	acgtacggat	1				no_annot
8055	tgtgatatgt	1				no_annot
8056	tttacgattc	1				no_annot
8057	cgattactcg	1				no_annot
8058	cagcgtcctc	1				no_annot
8059	ttctttttgg	1	TC15839	26	1	
8060	tttggctgtg	1	CD144919	45	0	
8061	aagaataagg	1	TC18503	47	0	
8062	cacttttgca	1				no_annot
8063	gtaagcaaag	1	CD189721	28	1	
8063	gtaagcaaag	1	CD069145	54	1	
8064	gtagtgagac	1				no_annot
8065	atatagttac	1	C311888.1	85	0	
8066	gtccgagcga	1				no_annot
8067	tttctgtcgt	1	TC17108	74	1	weakly similar to PIR|T50839|T50839 U4/U6 small nuclear ribonucleoprotein hPrp3 [imported] - human, partial (27%)
8068	gtagtattag	1				no_annot
8069	tgatttgaag	1				no_annot

8070	ctgcatttag	1				no_annot
8071	gtagtctagc	1	CD090477	25	2	
8072	tgcacccaca	1				no_annot
8073	tgataaacag	1	C707428.1	34	1	
8074	aaaaatattg	1	TC18666	84	0	weakly similar to GP|28950130|emb|CAD70988.1||BX294024 related to SCJ1 protein {Neurospora crassa}, partial (7%)
8075	atcactgacc	1				no_annot
8076	aagtatccac	1	TC6904	12	3	similar to GP|16040981|dbj|BAB69692.1||AB059656 POEM {Mus musculus}, partial (4%)
8076	aagtatccac	1	TC7108	21	12	
8076	aagtatccac	1	TC7108	21	11	
8076	aagtatccac	1	TC7108	21	10	
8077	taaacccata	1				no_annot
8078	cgtttattac	1	C209967.1	48	0	
8079	atacaaaatc	1				no_annot
8080	ggttatggcc	1				no_annot
8081	ctctagtagt	1				no_annot
8082	gttaactcgt	1	TC10742	60	3	similar to SP|P53488|ARP2_CHICK Actin-like protein 2 (Actin-like protein ACTL). [Chicken] {Gallus gallus}, partial (92%)
8083	gatttgtaca	1	CD166959	41	1	
8084	tataatgatg	1	CD116659	33	3	GP|4322668|gb|AAD16119.1||AF094759 retinoic acid receptor RXR {Schistosoma mansoni}, partial (13%)
8085	ctagtcattt	1				no_annot
8086	atcgaaaagg	1				no_annot
8087	taaaaaagac	1				no_annot
8088	tgctccactg	1				no_annot
8089	gtccagcacc	1				no_annot
8090	tatacaatat	1	TC17605	42	0	similar to SP|Q08220|GSHB_YEAST Glutathione synthetase(Glutathione synthase) (GSH synthetase) (GSH-S). [Baker's yeast] {Saccharomyces cerevisiae}, partial (9%)
8091	cgttgtccat	1				no_annot

8092	cgttacataa	1				no_annot
8093	ttcgcctaca	1				no_annot
8094	accaggttgt	1	TC10617	15	2	similar to GP|22004048|dbj|BAC06474.1||AB078979 ubiquitin {Ciona savignyi}, partial (49%)
8095	aggcaagtca	1				no_annot
8096	aaaaacatag	1	TC9644	46	0	
8097	cataacgact	1	C211158.1	26	1	
8097	cataacgact	1	C611788.1	26	1	
8098	tgtcttgtta	1				no_annot
8099	gaattgcacg	1				no_annot
8100	gttcggagaa	1				no_annot
8101	tgcgaaaaat	1				no_annot
8102	taaacgtacg	1				no_annot
8103	ttatcctcag	1	AI395852	47	2	
8104	gcccctaata	1				no_annot
8105	cctgctaata	1				no_annot
8106	aatctctacg	1				no_annot
8107	gatgaaacat	1				no_annot
8108	ttctaccata	1				no_annot
8109	cataaactta	1				no_annot
8110	gcggcatctg	1				no_annot
8111	acactaactt	1				no_annot
8112	cacatatagc	1				no_annot
8113	gtcaaccata	1				no_annot
8114	tttataagta	1	C609556.1	21	4	
8115	actgtagcca	1				no_annot
8116	agatcggtgc	1				no_annot
8117	ctccaagcta	1				no_annot
8118	gtccagcaat	1	C311588.1	78	0	
8118	gtccagcaat	1	C707109.1	78	0	
8119	aatgatagcg	1	AW017390	16	0	

8120	gtcaagtact	1	AI395768	1	0	
8121	aaatcttgtc	1				no_annot
8122	tgaggcaccc	1	TC17269	20	1	weakly similar to GP|23093913|gb|AAF50410.2||AE003555 CG6694-PA {Drosophila melanogaster}, partial (8%)
8123	gcaaaaagct	1				no_annot
8124	attgttaact	1	C200996.1	5	2	
8124	attgttaact	1	C608279.1	5	2	
8125	aatcacttgt	1				no_annot
8126	gtcacattga	1	TC17472	34	2	
8127	gtgtcagcgc	1	AA931037	23	1	
8128	tatattttgt	1	C607588.1	43	2	
8128	tatattttgt	1	C607588.1	43	0	
8129	ctgaaaggat	1	C605905.1	83	0	
8130	tagtctgttt	1				no_annot
8131	cataatgata	1	TC12817	79	1	
8131	cataatgata	1	TC14201	96	0	weakly similar to GP|11640592|gb|AAG39293.1|AF113222_1|AF113222 MSTP042 {Homo sapiens}, partial (60%)
8132	cccagataca	1				no_annot
8133	aagtttattc	1	CD081677	77	0	
8134	tacgtctttg	1				no_annot
8135	ataagccaac	1				no_annot
8136	ggtatttatt	1				no_annot
8137	agtaaatcac	1				no_annot
8138	tgctgaattt	1				no_annot
8139	caggacagct	1				no_annot
8140	agtaaatcaa	1				no_annot
8141	gagtctcatt	1				no_annot
8142	ttcctcacat	1				no_annot
8143	ttgtgcccaa	1				no_annot
8144	atataaacca	1	TC16515	25	1	

8145	taaaattcta	1	C300393.1	78	0	
8146	taggcaaccg	1	CD187142	55	1	
8147	ccctcagctg	1				no_annot
8148	tacatataca	1	TC7879	59	2	similar to SP|P51610|HFC1_HUMAN Host cell factor C1 (HCF) (VP16 accessory protein) (HFC1) (VCAF) (CFF). [Human] {Homo sapiens}, partial (3%)
8149	atttgtccat	1				no_annot
8150	caggacagca	1				no_annot
8151	tactgctaaa	1				no_annot
8152	aggatatatc	1				no_annot
8153	atgcggtctg	1	C200947.1	40	2	
8153	atgcggtctg	1	C602463.1	39	2	
8154	ttccacagta	1				no_annot
8155	ttggtcaaag	1				no_annot
8156	aaacacctga	1	TC9872	13	2	
8157	ttttcgtacc	1				no_annot
8158	ctctcgaggg	1				no_annot
8159	ccttagaaac	1				no_annot
8160	tgcagctgga	1				no_annot
8161	gcggcgttac	1				no_annot
8162	gtgtcctgtc	1	AI975673	83	0	
8163	aaggttatgg	1				no_annot
8164	gtagaagaga	1	CD169300	39	0	
8165	aaatatggac	1	C208070.1	21	0	
8165	aaatatggac	1	C603961.1	25	0	
8166	gtattcaacc	1	M80214|Sma.820	5	3	Alpha-tubulin (sat-1)
8167	tgtacttcca	1				no_annot
8168	atagctatat	1				no_annot
8169	acattctaga	1	TC11539	44	0	
8169	acattctaga	1	CD090120	61	0	
8170	ttaaactatg	1	TC18725	65	1	

8171	ttttactcca	1				no_annot
8172	tggtgatcca	1				no_annot
8173	actaagcaat	1	TC16997	98	0	similar to PIR|S34995|S34995 surface lipoprotein p27 - Lyme disease spirochete (strain B29), partial (8%)
8174	aactcacatt	1				no_annot
8175	ttgaaacgtg	1				no_annot
8176	acgaaagata	1	AF030969|Sma.246	71	2	Clone 10 unknown
8177	acgcagttcg	1	TC14835	83	0	similar to GP|19909515|dbj|BAB87800.1||AB013910 dermal papilla derived protein 6 (DERP6) {Homo sapiens}, partial (6%)
8178	atatttgaac	1	C204305.1	31	0	
8179	ggcccaaaaa	1	C610133.1	37	1	
8180	accacctact	1				no_annot
8181	tagagatgcc	1				no_annot
8182	gtagtttata	1	CD075661	15	0	
8183	gactacatac	1				no_annot
8184	cgatcagaac	1	C200556.1	57	5	
8185	ctccacagtt	1	CD118747	75	1	
8186	cgatcagaaa	1				no_annot
8187	aagcctggaa	1				no_annot
8188	tataccaacc	1				no_annot
8189	ctaccattgg	1				no_annot
8190	tttggcagca	1				no_annot
8191	aagaataaaa	1				no_annot
8192	aaggaagtca	1	TC18097	53	1	PIR|H72173|H72173 D5L protein - variola minor virus (strain Garcia-1966), partial (18%)
8193	ttctgtgttt	1				no_annot
8194	tcgccaagac	1				no_annot
8195	ccttaacttc	1				no_annot
8196	gatttactgt	1				no_annot
8197	agcaggaaac	1				no_annot
8198	gaaggaaaaa	1				no_annot

8199	gattctattc	1				no_annot
8200	gtgggttttt	1				no_annot
8201	ttgcccccct	1				no_annot
8202	gacttcggcg	1	TC11159	34	1	similar to GP|16198271|gb|AAL13964.1||AY058735 LP02521p {Drosophila melanogaster}, partial (33%)
8203	ttattttgac	1	TC15732	62	0	
8204	atactagcct	1	TC15379	61	0	
8205	taagccactt	1				no_annot
8206	ttttttctgt	1				no_annot
8207	atgactattt	1				no_annot
8208	cggtgcctcg	1				no_annot
8209	tacaacaaaa	1				no_annot
8210	ataatgataa	1	TC8338	46	0	similar to SP|Q8YRR1|MOAC_ANASP Molybdenum cofactor biosynthesis protein C. [strain PCC 7120] {Anabaena sp.}, partial (36%)
8211	atactcagga	1	TC14299	57	0	weakly similar to SP|Q9U1X4|MSS4_CAEEL Guanine nucleotide exchange factor MSS4 homolog. {Caenorhabditis elegans}, partial (37%)
8212	gatatgtttc	1				no_annot
8213	attaaacgat	1				no_annot
8214	cattgtgaca	1	C205781.1	83	0	
8214	cattgtgaca	1	C604964.1	86	0	
8215	cgtttctaaa	1				no_annot
8216	ccctacaaga	1				no_annot
8217	aagacctgga	1				no_annot
8218	ttgagcaaca	1				no_annot
8219	tatactaatc	1	AA931039	53	1	
8220	gaccaggatt	1				no_annot
8221	aaccaaaaga	1	C602993.1	39	0	
8222	caactcattg	1				no_annot
8223	cgtacttcca	1	TC14532	34	0	
8224	tacatacgtc	1				no_annot

8225	ttatatccta	1				no_annot
8226	gttattctga	1				no_annot
8227	tcagctgaaa	1				no_annot
8228	tagcactttg	1				no_annot
8229	gaaatgcttc	1	TC11050	61	0	
8230	agagagagag	1	C204022.1	90	1	
8231	ccttggatgg	1				no_annot
8232	attgcaaaat	1				no_annot
8233	tacagctttc	1				no_annot
8234	cagttttctt	1	C205375.1	81	0	
8235	gtacgtcata	1				no_annot
8236	cacatataac	1				no_annot
8237	cctgtacaac	1				no_annot
8238	tgatgctctc	1				no_annot
8239	gcacaacgga	1				no_annot
8240	cacaacaaat	1				no_annot
8241	atcttgctga	1				no_annot
8242	gatcaaaatt	1				no_annot
8243	agtgcgataa	1				no_annot
8244	aaaacgacga	1	TC11254	14	5	
8245	agcagtgatt	1				no_annot
8246	catacattgg	1				no_annot
8247	tactgtaact	1	CD079861	62	0	
8248	atcgaagggt	1				no_annot
8249	ttcatcgagt	1	TC17435	50	0	
8250	ctagcttcca	1	C203028.1	52	1	
8250	ctagcttcca	1	C607715.1	23	5	
8251	tagctgagga	1	CD202333	4	0	

8252	ttcaagtttg	1	TC13787	86	1	weakly similar to SP|P21796|POR1_HUMAN Voltage-dependent anion-selective channel protein 1 (VDAC-1) (hVDAC1) (Outer mitochondrial membrane protein porin 1) (Plasmalemmal porin) (Porin 31HL) (Porin 31HM). [Human] {Homo sapiens}, partial (10%)
8253	ttggatattg	1	TC7690	73	0	similar to SP|Q9DE27|RUV2_XENLA RuvB-like 2(Reptin). [African clawed frog] {Xenopus laevis}, partial (86%)
8254	ctccagcttg	1	C601645.1	33	0	
8255	ttgacctcca	1	TC14750	88	1	
8256	tcgaccgaat	1	AI977508	88	0	
8257	agcacataca	1				no_annot
8258	catactaata	1	N25373	50	0	
8259	ttggtattaa	1	CD187574	93	0	
8259	ttggtattaa	1	CD095670	25	0	similar to GP|4206713|gb|AAD11782.1||AF091239 c-13 antigen {Plasmodium falciparum}, partial (4%)
8260	taaaatacct	1	Z29959|Sma.25	7	3	Zinc finger protein (putative)
8261	atcgaaggga	1				no_annot
8262	gaatgatatc	1				no_annot
8263	cacgaagttt	1				no_annot
8264	attttctaag	1				no_annot
8265	tagcttaatc	1	TC9094	58	3	
8266	ctatcgcaaa	1				no_annot
8267	acactgttgt	1				no_annot
8268	aaaggacaaa	1				no_annot
8269	attttctaac	1	TC18348	77	2	weakly similar to GP|15799259|dbj|BAB20592.2||AB043587 golgi resident protein GCP60 {Homo sapiens}, partial (10%)
8270	gcttgactac	1				no_annot
8271	catcttctac	1				no_annot
8272	acatcgtctt	1				no_annot
8273	ttatgttatg	1				no_annot
8274	acgtataaag	1	CD082653	26	1	PIR|C96619|C96619 protein T30E16.7 [imported] - Arabidopsis thaliana, partial (31%)

8275	aacgctttaa	1				no_annot
8276	actccttcat	1				no_annot
8277	aaaaacttct	1				no_annot
8278	tcatatacga	1	TC10731	16	1	weakly similar to GP|13435488|gb|AAH04614.1||BC004614 mitochondrial ribosomal protein L30 {Mus musculus}, partial (22%)
8279	gacgatatct	1	CD135651	71	0	GP|860916|emb|CAA34726.1||X16826 60C beta tubulin {Drosophila melanogaster}, partial (9%)
8279	gacgatatct	1	CD135493	46	0	homologue to SP|P11833|TBB_PARLI Tubulin beta chain. [Common sea urchin] {Paracentrotus lividus}, partial (24%)
8280	tgttagccta	1				no_annot
8281	gtctggcgcg	1	TC7110	96	0	similar to GP|6715146|gb|AAF26302.1|AF184617_1|AF184617 proprotein convertase aPC6C isoform {Branchiostoma californiense}, partial (3%)
8281	gtctggcgcg	1	TC7188	46	2	similar to PIR|T02995|T02995 unspecific monooxygenase- common tobacco, partial (5%)
8281	gtctggcgcg	1	TC7242	74	0	similar to GP|6715146|gb|AAF26302.1|AF184617_1|AF184617 proprotein convertase aPC6C isoform {Branchiostoma californiense}, partial (3%)
8281	gtctggcgcg	1	TC7268	38	1	similar to PIR|T02995|T02995 unspecific monooxygenase- common tobacco, partial (5%)
8281	gtctggcgcg	1	TC7309	68	0	similar to PIR|T02995|T02995 unspecific monooxygenase- common tobacco, partial (7%)
8281	gtctggcgcg	1	CD088192	41	1	homologue to PIR|T02995|T02995 unspecific monooxygenase- common tobacco, partial (13%)
8281	gtctggcgcg	1	CD145873	88	0	similar to GP|22758896|gb|AAN05607.1||AF526226 ribosomal protein L7a {Argopecten irradians}, partial (26%)
8281	gtctggcgcg	1	CD201546	25	2	similar to PIR|T02995|T02995 unspecific monooxygenase- common tobacco, partial (20%)
8282	taaaaatgcc	1	C201490.1	74	1	
8282	taaaaatgcc	1	C607564.1	78	1	
8283	ggagtcacca	1	CD077324	32	0	
8284	gacgcaaaaa	1	TC18167	48	0	

8285	tcataatgga	1				no_annot
8286	tgaaacactt	1	TC14977	43	0	
8286	tgaaacactt	1	CD195288	25	1	
8287	tacttggctt	1				no_annot
8288	tttcacaaac	1				no_annot
8289	gctttgtttt	1	TC16471	23	3	
8290	gagatgcact	1				no_annot
8291	attgatatat	1	TC11740	89	0	weakly similar to GP|13661819|gb|AAK38111.1|AF360549_1|AF360549 BRCA1-binding helicase-like protein BACH1 {Homo sapiens}, partial (10%)
8292	tagctcaaaa	1				no_annot
8293	gaactctacc	1				no_annot
8294	tatagtgttg	1				no_annot
8295	tatagtgttc	1				no_annot
8296	ttactacaac	1	TC17015	40	5	
8297	tcttcattgg	1				no_annot
8298	agcttcgagg	1				no_annot
8299	taaattctaa	1				no_annot
8300	ttcaagatgt	1	TC17655	75	1	weakly similar to GP|7296187|gb|AAF51479.1||AE003589 CG2863-PA {Drosophila melanogaster}, partial (45%)
8301	ctctcgagaa	1				no_annot
8302	gtgtataagg	1	CD075448	87	0	weakly similar to GP|24348631|gb|AAN55622.1|AE015700_6|AE015700 dihydroorotate dehydrogenase {Shewanella oneidensis MR-1}, partial (12%)
8303	tctatagtgt	1				no_annot
8304	cagctattgt	1	C609621.1	94	0	
8305	gttagctgtt	1				no_annot
8306	acaccagttc	1	C308616.1	44	0	
8307	tgcttctggt	1	TC7427	91	0	

8307	tgcttctggt	1	TC7429	26	2	similar to GP|21928131|gb|AAM78093.1||AY125501 At2g29210/F16P2.41 {Arabidopsis thaliana}, partial (4%)
8307	tgcttctggt	1	TC14301	32	4	
8308	aaattgaatg	1				no_annot
8309	tacgtcatat	1				no_annot
8310	ttcaagatgc	1				no_annot
8311	aattggtcga	1				no_annot
8312	aataaaggtt	1				no_annot
8313	ctagtagata	1				no_annot
8314	gagcataaaa	1				no_annot
8315	tgttttaaat	1				no_annot
8316	gaaacggatt	1				no_annot
8317	catattttat	1				no_annot
8318	gctattttct	1	TC16891	35	3	weakly similar to GP|18033113|gb|AAL56988.1|AF328927_1|AF328927 serine dehydratase {Mus musculus}, partial (23%)
8319	tacaaattaa	1				no_annot
8320	gcgtgaaatg	1	TC13717	42	7	weakly similar to SP|Q9UK97|FBX9_HUMAN F-box only protein 9. [Human] {Homo sapiens}, partial (16%)
8321	taaaaatgat	1	TC14223	51	1	
8322	tgcataaatt	1				no_annot
8323	aggaaaatga	1				no_annot
8324	gtaagtaagt	1				no_annot
8325	taataaaata	1				no_annot
8326	gtattctagc	1	CD128094	62	3	
8327	ggagacagtg	1				no_annot
8328	cggcactgat	1				no_annot
8329	cttctataaa	1				no_annot
8330	tgcctcttgc	1				no_annot
8331	tacattgcca	1				no_annot
8332	aagaatggcc	1				no_annot

8333	tatggtcagg	1				no_annot
8334	ttaaaattgc	1				no_annot
8335	aatccaaacg	1				no_annot
8336	ttgttgtgtg	1				no_annot
8337	ctacaactga	1				no_annot
8338	caaaagcaaa	1	C209702.1	2	6	
8338	caaaagcaaa	1	C607103.1	1	10	
8339	aaaaacttag	1				no_annot
8340	tggcggtcga	1				no_annot
8341	ttccccgtac	1				no_annot
8342	cttcacaaac	1	TC9591	26	0	similar to SP|Q24368|ISWI_DROME Iswi protein (Imitation swi protein) (Nucleosome remodeling factor 140 kDa subunit) (NURF-140) (CHRAC 140 kDa subunit). [Fruit fly] {Drosophila melanogaster}, partial (11%)
8343	tatttgatcg	1				no_annot
8344	tttatatgtt	1				no_annot
8345	aaggatgatt	1	C313015.1	7	1	
8345	aaggatgatt	1	C703226.1	11	1	
8346	tatttgatca	1	TC8815	72	2	weakly similar to SP|Q05506|SYRC_YEAST Arginyl-tRNA synthetase, cytoplasmic(Arginine--tRNA ligase) (ArgRS). [Baker's yeast] {Saccharomyces cerevisiae}, partial (11%)
8346	tatttgatca	1	TC12266	22	3	
8347	catatgccaa	1				no_annot
8348	tggacagaaa	1				no_annot
8349	atgaaaaccc	1	CD165756	0	0	
8350	ttaaacaaat	1	TC18199	77	2	
8351	tgtaaagtta	1	TC9443	56	0	
8352	tgcagaccgc	1				no_annot
8353	gttgtcagcg	1	TC13827	61	1	similar to GP|4107017|dbj|BAA36294.1||AB001773 PEM-6 {Ciona savignyi}, partial (29%)

8353	gttgtcagcg	1	TC13828	4	2	homologue to GP|15529624|gb|AAL01375.1|AF406557_1|AF406557 PTPRE {Homo sapiens}, partial (8%)
8354	agcttgaaag	1				no_annot
8355	gtgtgcttga	1				no_annot
8356	ttatggtgcg	1	CD093237	45	1	
8357	tgtagtggaa	1				no_annot
8358	caatcggatt	1	TC17067	34	0	
8359	attgcataga	1				no_annot
8360	gtggatggag	1				no_annot
8361	taccgtgtgt	1	C609539.1	11	3	
8362	aagactaggt	1				no_annot
8363	gactaagaca	1	CD098192	57	0	weakly similar to GP|2804528|dbj|BAA24464.1||D85082 YfiX {Bacillus subtilis}, partial (11%)
8364	ctgaggagcg	1				no_annot
8365	catatacacc	1				no_annot
8366	ttaagagata	1				no_annot
8367	cagtagctgt	1				no_annot
8368	ttgttcaacg	1				no_annot
8369	ggcaaaaaaa	1				no_annot
8370	tttgattcaa	1	CD079956	67	0	similar to GP|26006387|gb|AAN77278.1|AF430841_1|AF430841 epsilon-tubulin {Xenopus laevis}, partial (25%)
8370	tttgattcaa	1	CD063645	91	0	
8371	atagtggtta	1				no_annot
8372	taggtttcaa	1				no_annot
8373	caactggctg	1				no_annot
8374	taggattgta	1	TC10530	74	0	similar to SP|O96860|DYL2_DROME Dynein light chain 2, cytoplasmic (8 kDa dynein light chain). [Fruit fly] {Drosophila melanogaster}, partial (74%)
8375	tacagcatca	1	TC10603	7	3	
8376	caaaatacaa	1				no_annot
8377	aggagcatta	1				no_annot

8378	atcttgctaa	1	TC11397	81	0	
8379	aagaattact	1	TC10674	33	8	
8379	aagaattact	1	TC10885	74	0	similar to SP|O00217|NUIM_HUMAN NADH-ubiquinone oxidoreductase 23 kDa subunit, mitochondrial precursor(Complex I-23KD) (CI-23KD) (TYKY subunit). [Human] {Homo sapiens}, partial (69%)
8380	gagatatgat	1				no_annot
8381	aagttcttat	1	CD149544	79	0	
8382	tcttgtgtta	1				no_annot
8383	tgccggattt	1				no_annot
8384	tgtatcatca	1	TC10025	97	0	
8385	ccaatgcatc	1				no_annot
8386	tctccgatcg	1				no_annot
8387	tacttctttg	1	TC10958	93	0	
8388	ctggggaata	1				no_annot
8389	atatctatcg	1				no_annot
8390	tgatgtactt	1	CD179964	19	0	
8391	aggggtaacg	1				no_annot
8392	tttcaattag	1	C612432.1	76	0	
8393	atatctatcc	1				no_annot
8394	tttcaattaa	1				no_annot
8395	gccaagagag	1	TC7417	33	4	weakly similar to GP|5114446|gb|AAD40318.1|AF158370_1|AF158370 DEAD-box RNA helicase {Gallus gallus}, partial (55%)
8396	gttgtacctc	1				no_annot
8397	tcagattcaa	1	TC12088	54	0	
8398	gcctgtaaac	1				no_annot
8399	gttactgaca	1				no_annot
8400	tcatcactca	1	C316001.1	21	2	
8401	gcttgccaaa	1				no_annot
8402	aagtagaaga	1	TC14048	43	1	

8402	aagtagaaga	1	CD202584	45	0	similar to GP|18249847|gb|AAK77986.1||AY040822 U4/U6 snRNP-associated 61 kDa protein {Homo sapiens}, partial (4%)
8403	cacccgtgac	1				no_annot
8404	tttaagcttt	1				no_annot
8405	gtgatcaaag	1				no_annot
8406	ttatgtaacc	1				no_annot
8407	ttatgtaaca	1				no_annot
8408	aacctttgta	1				no_annot
8409	cgggtggaag	1				no_annot
8410	gtgtataaat	1				no_annot
8411	tcttaactgt	1				no_annot
8412	ggctattgca	1	TC17338	48	2	
8413	agtagctcct	1				no_annot
8414	gactggggta	1				no_annot
8415	aaagtgaacg	1				no_annot
8416	tgtagtcgtt	1				no_annot
8417	tgtatcataa	1				no_annot
8418	tgtgatttac	1				no_annot
8419	gtagcaaaaa	1				no_annot
8420	tgtgatttaa	1				no_annot
8421	atttttaaaa	1				no_annot
8422	ctgctgatgg	1				no_annot
8423	tagtcaacat	1				no_annot
8424	tcatcccagt	1	C200657.1	53	1	
8424	tcatcccagt	1	C201869.1	40	1	
8424	tcatcccagt	1	C604428.1	53	1	
8425	tgatttcagt	1				no_annot
8426	cgcttctgct	1				no_annot
8427	ataggaagag	1	TC9513	82	0	
8428	cgtcaaagtg	1				no_annot

8429	gatggatgtg	1				no_annot
8430	gtaccagttt	1				no_annot
8431	ataggaagaa	1				no_annot
8432	ataatgttag	1				no_annot
8433	tgcaaaattc	1				no_annot
8434	tagcttggtc	1				no_annot
8435	actgtttgtt	1	TC14683	93	0	similar to GP|7300349|gb|AAF55508.1||AE003721 CG7187-PA {Drosophila melanogaster}, partial (22%)
8436	atacaaaaca	1				no_annot
8437	gtattctaag	1	C310937.1	27	1	
8438	ttgttgagct	1	TC15424	55	2	homologue to GP|7302308|gb|AAF57398.1||AE003790 CG3450-PA {Drosophila melanogaster}, partial (44%)
8439	cagcgtccat	1				no_annot
8440	gctcgcagaa	1				no_annot
8441	aattgatacc	1				no_annot
8442	cgacggagta	1				no_annot
8443	cagtagctca	1				no_annot
8444	gttggcaatg	1				no_annot
8445	cagatgcttg	1	TC7476	52	1	
8445	cagatgcttg	1	TC7477	57	2	
8445	cagatgcttg	1	CD188169	85	0	
8446	cgttgagtca	1				no_annot
8447	gccacagcct	1	C208026.1	6	1	
8448	tttcactact	1				no_annot
8449	aactctttct	1				no_annot
8450	tcagtgactg	1				no_annot
8451	ggttgacaaa	1				no_annot
8452	actaattcaa	1				no_annot
8453	gatgctttga	1				no_annot
8454	atcgtaccat	1				no_annot
8455	ctggaggcaa	1				no_annot

8456	agataattat	1				no_annot
8457	tttcgtatta	1				no_annot
8458	ggtcgaagca	1				no_annot
8459	tatttgttgt	1	TC17785	54	1	weakly similar to GP|927736|gb|AAB64908.1||U33050 Ydr492wp; CAI: 0.19 {Saccharomyces cerevisiae}, partial (15%)
8460	cattaggata	1				no_annot
8461	attgcataag	1				no_annot
8462	attgatttct	1				no_annot
8463	ggtttagtgg	1				no_annot
8464	tatttgttgg	1				no_annot
8465	atgatccaac	1				no_annot
8466	tcctgcatat	1	TC7118	93	1	weakly similar to GP|28274854|gb|AAO25692.1||AY195856 ankyrin repeat protein E4_8 {synthetic construct}, partial (28%)
8467	gcgctctaga	1	TC10819	22	0	
8468	gaaaaaggtt	1	C205224.1	87	3	
8468	gaaaaaggtt	1	C607765.1	76	5	
8469	ggtttagtga	1	C211125.1	16	4	
8470	cttgtgacta	1	TC13646	79	0	similar to GP|5733602|gb|AAD49698.1|AF163255_1|AF163255 adaptor protein DAPP1 {Mus musculus}, partial (5%)
8471	aatgacattt	1				no_annot
8472	taaacccaaa	1				no_annot
8473	cagtcaacaa	1	TC16839	55	0	
8474	tgtgtgattt	1				no_annot
8475	atcactcaga	1				no_annot
8476	ttccttacct	1				no_annot
8477	taataaggta	1				no_annot
8478	tataatgaag	1				no_annot
8479	aagactagag	1				no_annot
8480	ctgcgccaaa	1	L36833|Sma.1189	77	1	(clone C5-1B3) phosphoglycerate kinase (PGK) gene

8481	ggcaccacat	1	TC11462	47	2	similar to SP|O15491|AN1H_HUMAN WD-repeat protein An11 homolog. [Mouse] {Mus musculus}, partial (62%)
8482	ttcaatgtta	1				no_annot
8483	gtataagacg	1				no_annot
8484	ttacaccacc	1				no_annot
8485	cttacgatat	1				no_annot
8486	gtgcttgaaa	1				no_annot
8487	tcacactact	1				no_annot
8488	tcaccagcca	1				no_annot
8489	atttatatcg	1				no_annot
8490	attcaaagtc	1	TC8175	35	0	
8491	tgattggttg	1				no_annot
8492	taaaattcct	1				no_annot
8493	gtccgctgat	1				no_annot
8494	aagtttatca	1				no_annot
8495	gagagaaagg	1				no_annot
8496	ctgaggtggc	1	C610663.1	28	2	
8497	ttgttattac	1	C305312.1	2	1	
8498	tcgacactga	1				no_annot
8499	tcacactacc	1				no_annot
8500	gaaccaaaca	1				no_annot
8501	ttaagcatca	1				no_annot
8502	tgactaccgg	1				no_annot
8503	aaccagtcag	1				no_annot
8504	acagcataaa	1				no_annot
8505	aaatgacaac	1	TC7875	66	0	
8506	gcagtctggt	1				no_annot
8507	cacaataaga	1				no_annot
8508	cataaactac	1				no_annot
8509	cacaccagca	1				no_annot

8510	catacctaaa	1	CD136656	7	2	
8511	gtttccactg	1				no_annot
8512	ctgagttatg	1				no_annot
8513	ctctaccacc	1	C200935.1	90	0	
8513	ctctaccacc	1	C309102.1	28	2	
8513	ctctaccacc	1	C606965.1	85	0	
8513	ctctaccacc	1	C712718.1	26	2	
8514	aattattgga	1	C607487.1	82	1	
8515	taatgaatta	1	TC13866	73	3	
8515	taatgaatta	1	TC17341	40	6	
8515	taatgaatta	1	AW017061	75	0	
8515	taatgaatta	1	CD124443	73	0	
8516	aaaaggaacc	1	TC10387	70	1	
8516	aaaaggaacc	1	TC10387	70	0	
8516	aaaaggaacc	1	TC10405	19	3	
8517	ttacatcata	1	CD073704	24	1	
8518	tttaggactt	1				no_annot
8519	aaggagtaaa	1				no_annot
8520	gacgatttcc	1				no_annot
8521	ccttgccttg	1				no_annot
8522	aatggcccaa	1				no_annot
8523	tttaccgtat	1				no_annot
8524	gaggaaatga	1				no_annot
8525	atccagtata	1				no_annot
8526	aacattgtag	1				no_annot
8527	attatcataa	1				no_annot
8528	atcttttgta	1				no_annot
8529	ccaggttgta	1				no_annot
8530	gtgtattagc	1	Z32529|Sma.852	79	1	Cathepsin L
8531	cagcaagaga	1				no_annot

8532	aacattgtac	1	C314858.1	63	1	
8532	aacattgtac	1	C712246.1	63	1	
8533	attgatttaa	1				no_annot
8534	cacgccaatg	1				no_annot
8535	tttcataagt	1				no_annot
8536	tttcaccgtt	1	TC7708	91	0	similar to GP|28866891|dbj|BAC65166.1||AB092504 IP3R binding protein released with inositol 1,4,5-trisphosphate {Mus musculus}, partial (38%)
8537	ggtgagatac	1				no_annot
8538	ataggatggg	1	CD141285	60	1	
8539	gagcattaac	1				no_annot
8540	agatgcatca	1	C200663.1	11	3	
8541	ggatcgatga	1	CD201844	17	0	
8542	tgacagtcat	1				no_annot
8543	cataatgaac	1				no_annot
8544	tgttgacttg	1				no_annot
8545	cataatgaaa	1				no_annot
8546	tgccacgagc	1	M80214|Sma.820	12	2	Alpha-tubulin (sat-1)
8547	ctttcccacc	1				no_annot
8548	tgaatcctga	1	TC11625	9	5	
8548	tgaatcctga	1	TC11625	9	3	
8549	ccgttgcttg	1	TC7050	46	3	similar to GP|13171103|gb|AAK13589.1||AF139989 rRNA intron-encoded homing endonuclease {Oryza sativa}, partial (52%)
8550	aataaaaaaa	1				no_annot
8551	gaaccaaaac	1	CD191805	61	0	
8552	taacagaatg	1				no_annot
8553	ttgtttggtt	1				no_annot
8554	ctaaatggtg	1	AY948980|Sma.5319	43	0	Mitochondrial thioredoxin precursor, mRNA, complete cds; nuclear gene for mitochondrial product
8555	tagtctgtaa	1	C301232.1	82	0	
8555	tagtctgtaa	1	C611708.1	83	0	

8556	taacagaata	1	C210582.1	2	3	
8556	taacagaata	1	C709840.1	3	3	
8557	ctacgtgtgc	1				no_annot
8558	gatccaagtg	1				no_annot
8559	aaacttcaag	1	TC17102	89	0	similar to SP|Q9Y697|NFS1_HUMAN Cysteine desulfurase, mitochondrial precursor(HUSSY-08). [Human] {Homo sapiens}, partial (85%)
8560	tataaccaca	1				no_annot
8561	ccatattcga	1	C604294.1	17	3	
8562	gttaattcct	1				no_annot
8563	ataataacac	1				no_annot
8564	caagtctgcg	1				no_annot
8565	ataaaaagta	1	TC8201	13	0	
8566	gttggatttg	1				no_annot
8567	atttgagtac	1				no_annot
8568	gctgttgtga	1				no_annot
8569	ggatacagtg	1	C201766.1	89	0	
8570	gcagcggaag	1				no_annot
8571	cctatcgacg	1				no_annot
8572	ttcagggata	1				no_annot
8573	tgtccagtac	1	C611123.1	56	1	
8574	aaggtacaca	1	CD080042	65	0	similar to GP|2642187|gb|AAB86925.1||AF023657 endo-alpha-D-mannosidase {Rattus norvegicus}, partial (4%)
8575	tgttttcgtt	1				no_annot
8576	acggtgtatc	1				no_annot
8577	cggtccgtta	1				no_annot
8578	gttagctggt	1				no_annot
8579	ttgtggaata	1				no_annot
8580	tccctactgt	1				no_annot
8581	tagtgaaacg	1	TC18676	70	0	weakly similar to SP|P20908|CA15_HUMAN Collagen alpha 1(V) chain precursor. [Human] {Homo sapiens}, partial (4%)

8582	taagaatagc	1				no_annot
8583	acgaattttt	1	CD124031	67	0	
8583	acgaattttt	1	AW061369	65	0	
8584	aaattgaaga	1	L01634|Sma.848	82	4	Myosin heavy chain (MYH)
8585	atcccgagtt	1				no_annot
8586	gagaactgcg	1	TC10488	62	0	weakly similar to SP|P51989|RO21_XENLA Heterogeneous nuclear ribonucleoprotein A2 homolog 1 (hnRNP A2(A)). [African clawed frog] {Xenopus laevis}, partial (31%)
8587	gcgtactggg	1				no_annot
8588	gctactaaga	1				no_annot
8589	caagtttgtg	1				no_annot
8590	aaggaactgt	1				no_annot
8591	caatttagtt	1				no_annot
8592	tttggaccct	1				no_annot
8593	tacggtcggt	1				no_annot
8594	aattattgct	1				no_annot
8595	tttcgcttgc	1	C208486.1	70	0	
8596	aaaatttaat	1	CD120441	66	1	
8597	cggtgtctcc	1				no_annot
8598	cggtgtctca	1				no_annot
8599	gcaggattta	1				no_annot
8600	aaggaactga	1	C606634.1	19	6	
8601	tatttgttat	1	C714861.1	71	0	
8602	cattcgaatc	1				no_annot
8603	tatttaacga	1	TC13963	89	0	
8604	aaaacgtcac	1				no_annot
8605	ttttattcac	1				no_annot
8606	ttaaccagcc	1				no_annot
8607	cagtggacag	1				no_annot
8608	ggtttagtac	1				no_annot
8609	gagaattgtg	1	C306496.1	48	1	

8610	acgacgacca	1	C204144.1	50	0	
8610	acgacgacca	1	C605329.1	49	0	
8611	aactgctcaa	1				no_annot
8612	tcggtgctct	1	TC11268	19	2	weakly similar to GP|28628069|gb|AAO38689.1||AY167035 long-chain acyl-CoA synthetase {Mus musculus}, partial (19%)
8613	gttcagaatc	1	TC6991	82	1	weakly similar to GP|22164073|gb|AAM93547.1|AF412216_1|AF412216 polyprotein {Schistosoma japonicum}, partial (76%)
8613	gttcagaatc	1	TC15273	26	1	
8613	gttcagaatc	1	CD124735	6	0	
8614	aatgtaatca	1	gi|161041|gb|L07513.1|SCMMTEGANT	74	4	Schistosoma mansoni major tegumental antigen SM15 (GSM70) gene, complete cds
8615	taccgatcaa	1				no_annot
8616	aatatttgtc	1				no_annot
8617	aatcatttca	1	TC17785	95	0	weakly similar to GP|927736|gb|AAB64908.1||U33050 Ydr492wp; CAI: 0.19 {Saccharomyces cerevisiae}, partial (15%)
8618	taaatgactt	1				no_annot
8619	tacatacgat	1				no_annot
8620	ctggggtata	1				no_annot
8621	atgttaagta	1				no_annot
8622	tagctgtgaa	1				no_annot
8623	cgttgtgata	1				no_annot
8624	ctggcaaaca	1	TC12712	81	0	weakly similar to GP|13249171|gb|AAK16714.1||AF327458 caffeoyl-CoA 3-O-methyltransferase {Populus alba x Populus glandulosa}, partial (17%)
8625	ttcactattc	1				no_annot
8626	aatttcaatg	1	TC14666	82	0	weakly similar to GP|17946013|gb|AAL49050.1||AY071428 RE51791p {Drosophila melanogaster}, partial (60%)
8627	atatctttca	1	C315138.1	5	2	
8627	atatctttca	1	C607508.1	2	5	
8628	taagccacac	1				no_annot

8629	aggcaccagt	1	CD060192	3	1	homologue to GP|10176|emb|CAA29285.1||X05842 eggshell precursor protein {Schistosoma mansoni}, partial (34%)
8630	gacccgaagt	1				no_annot
8631	gtgatttatg	1				no_annot
8632	caactcatag	1				no_annot
8633	tcttgaacca	1	TC16499	63	0	
8634	tttacatctt	1	CD158137	69	0	
8634	tttacatctt	1	CD189574	45	1	
8635	attatggcca	1				no_annot
8636	caagttaggg	1				no_annot
8637	gtgccacatt	1	TC8579	81	0	similar to GP|5669107|gb|AAD46165.1|AF144732_1|AF144732 protein/lipid phosphatase Pten {Xenopus laevis}, partial (16%)
8638	aggtgtaaca	1	CD073947	77	0	
8639	attccagtca	1				no_annot
8640	gcgcaggcaa	1				no_annot
8641	taccatcagt	1				no_annot
8642	acattttacg	1				no_annot
8643	aacacgaaag	1	TC18054	18	3	
8643	aacacgaaag	1	CD085341	72	0	similar to PIR|G84130|G84130 ABC transporter (permease) BH3847 [imported] - Bacillus halodurans (strain C-125), partial (12%)
8644	ttgtgcgatt	1	TC9990	14	0	
8645	ctcaaatctt	1	TC11261	93	1	homologue to GP|28916470|gb|AAO59418.1||AY220751 calcineurin B {Schistosoma japonicum}, complete
8646	cgcataaaga	1				no_annot
8647	agacaccttc	1				no_annot
8648	aaacgtacgt	1				no_annot
8649	tatgtcctct	1				no_annot
8650	aattgtgcat	1				no_annot
8651	atcggattac	1				no_annot
8652	acggatcgcc	1				no_annot

8653	cagtccctca	1				no_annot
8654	attcgggtgt	1				no_annot
8655	ataccaagga	1				no_annot
8656	caggtaattc	1				no_annot
8657	tgctgattgc	1				no_annot
8658	agcagtgaaa	1				no_annot
8659	cagcattttc	1	C201326.1	12	5	
8659	cagcattttc	1	C611019.1	11	5	
8660	tctaatgtta	1				no_annot
8661	aaacgatggc	1				no_annot
8662	aaatatcgaa	1				no_annot
8663	gtttcaagtt	1	C208540.1	8	2	
8663	gtttcaagtt	1	C602466.1	8	2	
8664	ttgtaggcaa	1				no_annot
8665	tgtcctgatt	1				no_annot
8666	cttggtagaa	1				no_annot
8667	gggtgagcga	1				no_annot
8668	caaagttgtt	1				no_annot
8669	tgttgccata	1				no_annot
8670	tgcataaact	1				no_annot
8671	gttttgctat	1				no_annot
8672	cagcacttac	1				no_annot
8673	aggattgccg	1				no_annot
8674	gaatattggt	1				no_annot
8675	cacgcaatga	1				no_annot
8676	atttttcgtc	1				no_annot
8677	tgatatctaa	1				no_annot
8678	aatcaacacc	1	TC15193	97	0	
8679	gttgagtcat	1	TC14967	47	0	
8680	caatgcaata	1				no_annot

8681	gaatattgga	1	C205909.1	51	2	
8682	tcgaattcgg	1				no_annot
8683	tggttcacct	1				no_annot
8684	ctcaaaacga	1	TC6927	17	0	
8684	ctcaaaacga	1	TC10390	69	0	
8685	gtttgcaatg	1				no_annot
8686	cagtccctaa	1				no_annot
8687	acaaagggta	1				no_annot
8688	ttagagaagc	1				no_annot
8689	taatatgctt	1				no_annot
8690	cgagtggtaa	1				no_annot
8691	aaattgaaaa	1	CD116881	92	0	
8692	tattttgtgg	1				no_annot
8693	ccgttctgtt	1	TC18314	37	0	similar to GP|172215|gb|AAA34890.1||J03530 RNA polymerase A(I) large subunit {Saccharomyces cerevisiae}, partial (7%)
8693	ccgttctgtt	1	CD097516	11	3	
8694	tattttgtga	1				no_annot
8695	ttgtcaaagt	1				no_annot
8696	actaaaatta	1	CD076939	30	1	
8697	tattcgggtt	1				no_annot
8698	gagtgttgat	1				no_annot
8699	tcaccctgga	1	TC15427	91	0	
8700	tattcgggtg	1				no_annot
8701	ggtgtactgg	1				no_annot
8702	ggagacagaa	1				no_annot
8703	gcgatcagga	1	C204653.1	54	0	
8703	gcgatcagga	1	C601356.1	52	0	
8704	tgaaaaaggg	1	CD073067	77	0	
8705	tattcgggta	1				no_annot
8706	atcggctact	1				no_annot

8707	tatgacacag	1				no_annot
8708	aacttggtgt	1	TC9688	49	2	
8709	gaggacaact	1	TC12486	38	1	
8709	gaggacaact	1	CD191557	7	1	similar to GP|5597017|gb| reverse transcriptase {synthetic construct}, partial (2%)
8709	gaggacaact	1	CD073032	79	2	homologue to GP|1086564|gb|AAB68971.1||U39941 orfB; Method: conceptual translation supplied by author. {Agrobacterium tumefaciens}, partial (7%)
8710	cttgctcaac	1				no_annot
8711	tgcataaaaa	1				no_annot
8712	attaagtatc	1	C200945.1	12	5	
8712	attaagtatc	1	C607639.1	7	10	
8713	tgtacaatgt	1	TC18172	85	0	weakly similar to GP|25012420|gb|AAN71317.1||BT001562 RE14391p {Drosophila melanogaster}, partial (4%)
8714	tgggactgtt	1				no_annot
8715	acccaatttt	1				no_annot
8716	actagaccat	1				no_annot
8717	ataacagtag	1				no_annot
8718	gttggaatag	1				no_annot
8719	actgcttctt	1				no_annot
8720	aataaataca	1	C201481.1	0	8	
8720	aataaataca	1	C600633.1	0	3	
8721	aacacgtagt	1				no_annot
8722	tcaaacagct	1				no_annot
8723	tcctgtatgt	1				no_annot
8724	ttcaccttga	1	AW017462	91	0	
8724	ttcaccttga	1	CD167333	92	0	
8725	gctgcagtat	1				no_annot
8726	ctaacgctat	1				no_annot
8727	tgttaacaaa	1				no_annot
8728	aggaccagga	1	CD131622	6	0	

8729	ttccgaaacg	1				no_annot
8730	ttagtacata	1				no_annot
8731	cttatatgcg	1				no_annot
8732	atctgcaaca	1				no_annot
8733	gtggtgaagt	1	TC18025	57	0	
8734	tatgaaaggt	1	C605541.1	13	2	
8735	agtgtttcat	1				no_annot
8736	gtgaagccta	1	CD084327	51	0	
8736	gtgaagccta	1	CD129359	20	3	
8737	aatgtattgc	1				no_annot
8738	tgaagggtaa	1				no_annot
8739	cttgtgacgc	1				no_annot
8740	acgacaggga	1				no_annot
8741	gcgtcatcaa	1				no_annot
8742	gacaagtatc	1	TC7967	7	0	similar to GP|14335082|gb|AAK59820.1||AY037220 AT4g24060/T19F6_50 {Arabidopsis thaliana}, partial (7%)
8742	gacaagtatc	1	TC17657	75	0	weakly similar to GP|13625461|gb|AAK35065.1|AF353991_1|AF353991 BBP-like protein 1 {Homo sapiens}, partial (53%)
8743	atctgtaatt	1	TC10169	87	0	
8744	ttgcccgatc	1				no_annot
8745	tggcgtcctt	1				no_annot
8746	gttgaataag	1				no_annot
8747	cgaaaaaggt	1	TC7485	48	0	similar to GP|161038|gb|AAA29903.1||M15509 major egg antigen {Schistosoma mansoni}, partial (65%)
8747	cgaaaaaggt	1	TC7486	38	1	similar to GP|161038|gb|AAA29903.1||M15509 major egg antigen {Schistosoma mansoni}, partial (49%)
8748	atgcattgat	1				no_annot
8749	gcataaaaca	1	TC11514	58	0	similar to GP|17946706|gb|AAL49383.1||AY071761 RH66629p {Drosophila melanogaster}, partial (78%)
8750	gaggtataac	1	TC17529	83	1	weakly similar to GP|17862592|gb|AAL39773.1||AY069628 LD39967p {Drosophila melanogaster}, partial (17%)

8751	gttgaataaa	1				no_annot
8752	ccaaagctgc	1	TC10793	45	2	similar to GP|22832350|gb|AAF48587.2||AE003501 CG9915-PA {Drosophila melanogaster}, partial (8%)
8753	acaatttttc	1				no_annot
8754	agttttacag	1				no_annot
8755	cacaatggca	1	CD065016	93	0	
8756	acacacacac	1				no_annot
8757	ctggtttact	1				no_annot
8758	aagtttttaa	1				no_annot
8759	ctgtaagaaa	1				no_annot
8760	aataaataag	1				no_annot
8761	gtttatgcta	1				no_annot
8762	tattcagtgc	1				no_annot
8763	ggtactgcat	1	TC14165	14	0	weakly similar to GP|17944511|gb|AAL48144.1||AY070673 RH10688p {Drosophila melanogaster}, partial (12%)
8763	ggtactgcat	1	CD077977	85	0	
8763	ggtactgcat	1	CD078834	33	1	
8764	acatagccca	1				no_annot
8765	ctgctgttat	1	TC13812	20	1	similar to GP|4966317|gb|AAA88778.2||U46856 vitellogenin {Anolis pulchellus}, partial (3%)
8766	cagtgataga	1	TC13781	14	2	
8767	aataaataaa	1	AI067470	5	2	
8768	gctgacacca	1				no_annot
8769	agttacagat	1	CD179024	45	1	
8770	ttggtttaaa	1				no_annot
8771	gtatggaaat	1				no_annot
8772	gacgccccag	1				no_annot
8773	gatcctactg	1				no_annot
8774	tcgggatttc	1				no_annot
8775	caatgatttt	1				no_annot
8776	cagaaatatg	1	CD131829	46	1	

8777	attattatga	1	CD126588	13	1	weakly similar to GP|28057131|gb|AAO28996.1||AE012557 ankyrin-like protein {Xylella fastidiosa Temecula1}, partial (3%)
8778	tgaagcagta	1				no_annot
8779	aaaaaacaat	1	AW587534	24	0	
8779	aaaaaacaat	1	CD082397	73	2	
8780	atgatggttt	1				no_annot
8781	ataatatcag	1				no_annot
8782	tcaaacagag	1				no_annot
8783	cttaatagta	1				no_annot
8784	aaaaggtaaa	1	C601653.1	10	6	
8785	tgtaagaaaa	1				no_annot
8786	ctcatactag	1	C601258.1	61	0	
8787	ggatactgta	1	C202013.1	22	2	
8787	ggatactgta	1	C600572.1	42	0	
8788	atacgtaatc	1				no_annot
8789	atgatggtta	1				no_annot
8790	tgcaaaatca	1				no_annot
8791	tgaaaaagct	1				no_annot
8792	tatttcctgt	1				no_annot
8793	tcgttcagca	1	C200663.1	81	0	
8794	gcataaaaac	1				no_annot
8795	ataagagcta	1				no_annot
8796	gcgaagacga	1	TC10551	74	3	homologue to GP|1314718|gb|AAA99798.1||U54586 Pro-His-rich protein {Schistosoma mansoni}, complete
8797	cacaatggaa	1				no_annot
8798	atccttttga	1	AI976835	42	0	
8799	tgtggtcatc	1				no_annot
8800	tgtacctatt	1				no_annot
8801	tcggtttgtg	1				no_annot
8802	ctcctttcac	1				no_annot
8803	tctgatcaag	1				no_annot

8804	tagatgctaa	1				no_annot
8805	caacaagtga	1	CD139165	38	2	
8806	gtatgacttg	1				no_annot
8807	gattatctgt	1				no_annot
8808	gtgtggcccg	1	AF473536|Sma.90	27	1	Thioredoxin
8809	ttcatccact	1				no_annot
8810	ccgcgtggac	1	TC16725	43	2	similar to PIR|T13159|T13159 E1B-55kDa-associated protein - human, partial (18%)
8811	gtgtggcccc	1				no_annot
8812	acaacatttg	1				no_annot
8813	ttgcaggagg	1				no_annot
8814	atgtcgatga	1				no_annot
8815	caattttgta	1	TC10541	41	0	homologue to GP|21686538|gb|AAM74948.1|AF519808_1|AF519808 MA {Schistosoma japonicum}, partial (11%)
8816	gttgcaagag	1				no_annot
8817	cattttgtct	1				no_annot
8818	tactcattta	1				no_annot
8819	ggcagtggtt	1				no_annot
8820	agttggacgc	1				no_annot
8821	ctgtttaaaa	1				no_annot
8822	tgaaggcttg	1				no_annot
8823	tgagaaaata	1				no_annot
8824	cttactgtgc	1				no_annot
8825	gacaaagtca	1	C201041.1	12	3	
8825	gacaaagtca	1	C604199.1	11	3	
8826	attcactcgg	1				no_annot
8827	ttagttataa	1				no_annot
8828	aatcttcgga	1				no_annot
8829	tgttttcggt	1				no_annot
8830	ttggcataaa	1				no_annot
8831	ttcgtctctc	1				no_annot

8832	aagatgaact	1				no_annot
8833	gaacagccaa	1				no_annot
8834	gaaactactc	1				no_annot
8835	aagttacaag	1				no_annot
8836	atgactttag	1				no_annot
8837	cacgttttga	1				no_annot
8838	taataaggca	1				no_annot
8839	aatttaatca	1				no_annot
8840	tttaactggt	1				no_annot
8841	gaaggacgag	1				no_annot
8842	gacgtgtttt	1				no_annot
8843	tgacgtggtg	1				no_annot
8844	gtaaccaatt	1				no_annot
8845	aaaagactgg	1	CD077304	72	0	
8845	aaaagactgg	1	CD097384	15	0	
8846	gatacagtca	1				no_annot
8847	gtatgcgaac	1				no_annot
8848	tgcctacaca	1				no_annot
8849	taatgctatg	1				no_annot
8850	catttatcgc	1				no_annot
8851	ctgcaggagg	1				no_annot
8852	agacccttca	1	C206709.1	18	1	
8852	agacccttca	1	C608188.1	17	1	
8853	agcattgttt	1				no_annot
8854	caagcttcta	1				no_annot
8855	gtcgttctga	1				no_annot
8856	cactcattta	1				no_annot
8857	ttactcacta	1				no_annot
8858	caacttatgg	1				no_annot
8859	ccaggttgcg	1				no_annot

8860	gactaacaaa	1	TC7426	36	1	weakly similar to PIR|B47328|B47328 natural killer cell tumor-recognition protein - mouse, partial (5%)
8861	cacgaccatt	1				no_annot
8862	tgctgttatt	1				no_annot
8863	gcattctgag	1	C201383.1	40	2	
8863	gcattctgag	1	C610185.1	38	2	
8864	tgggtatgat	1				no_annot
8865	taaccaatgt	1				no_annot
8866	gagtaaatag	1				no_annot
8867	accgaatgag	1	C200247.1	16	2	
8867	accgaatgag	1	C603237.1	18	0	
8868	aaactggtaa	1				no_annot
8869	tagcatttga	1				no_annot
8870	ttcaatgtac	1	TC14158	97	0	weakly similar to GP|19343832|gb|AAH25482.1||BC025482 Trim24 protein {Mus musculus}, partial (5%)
8871	acttatgcgt	1				no_annot
8872	atcaaaatct	1	C711705.1	95	0	
8873	tccccacatc	1				no_annot
8874	aagcctgttg	1				no_annot
8875	tacagtttgc	1				no_annot
8876	taaatgacgc	1	C210739.1	7	4	
8876	taaatgacgc	1	C612550.1	7	4	
8877	aagctaagaa	1	TC6865	27	2	
8878	tttgtaaaac	1				no_annot
8879	gaacaacaag	1				no_annot
8880	attgggcgga	1				no_annot
8881	taaggattaa	1				no_annot
8882	ttattttttc	1	CD126541	13	0	
8882	ttattttttc	1	CD169547	34	1	
8883	ctaacttgta	1				no_annot

8884	aacaaatggt	1	TC11819	14	1	similar to SP|O87885|RL27_LAWIN 50S ribosomal protein L27. {Lawsonia intracellularis}, partial (20%)
8885	tcatctcaca	1				no_annot
8886	tgaacgtaaa	1				no_annot
8887	aggaataaca	1				no_annot
8888	taaatatatg	1	TC11450	94	0	weakly similar to SP|P30260|CC27_HUMAN Protein CDC27Hs (Cell division cycle protein 27 homolog) (H-NUC). [Human] {Homo sapiens}, partial (6%)
8889	tctgtggaga	1				no_annot
8890	tatttatcca	1	TC9266	87	0	weakly similar to SP|O00762|UBCC_HUMAN Ubiquitin-conjugating enzyme E2 C(Ubiquitin-protein ligase C) (Ubiquitin carrier protein C) (UbcH10). [Human] {Homo sapiens}, partial (74%)
8890	tatttatcca	1	TC9782	19	4	PIR|C53153|C53153 glucose transport protein SGTP4 - fluke (Schistosoma mansoni), partial (10%)
8891	cttcgtatac	1				no_annot
8892	taaatatatc	1				no_annot
8893	cacacacccc	1				no_annot
8894	gtttccacac	1				no_annot
8895	tgacgttatg	1				no_annot
8896	gcagcgcagc	1				no_annot
8897	tagagatttt	1				no_annot
8898	ttcgaagctg	1				no_annot
8899	tgttgactat	1				no_annot
8900	ttatgcgttt	1	CD083785	5	1	weakly similar to GP|1110512|gb|AAA83210.1||U40484 MAP kinase {Aplysia californica}, partial (28%)
8901	acgaaacagt	1	TC19441	28	2	
8902	ggcgttaacg	1				no_annot
8903	cttgaaatga	1				no_annot
8904	taataataat	1				no_annot
8905	aattaagatg	1				no_annot
8906	ttcagctcta	1				no_annot
8907	gccattccac	1				no_annot
8908	tccacatata	1				no_annot

8909	tatatcacta	1				no_annot
8910	agccttagta	1				no_annot
8911	tttagaaact	1				no_annot
8912	gggcccagca	1	TC14344	66	0	
8913	aatactacga	1				no_annot
8914	gacccgtaca	1				no_annot
8915	tggaggcaag	1				no_annot
8916	ggacatattt	1	CD077440	60	0	
8917	aatgggaatg	1				no_annot
8918	tgcaataatg	1				no_annot
8919	gtggaaactc	1				no_annot
8920	ggttaatctg	1				no_annot
8921	ctagcaataa	1				no_annot
8922	taacagaaac	1				no_annot
8923	gaattcggtt	1				no_annot
8924	acaaggtttc	1				no_annot
8925	acaacaatca	1				no_annot
8926	tttctgaatt	1				no_annot
8927	caaatatatc	1	CD116906	24	1	
8928	ttatcatttt	1	CD079010	42	0	
8929	catttatcca	1				no_annot
8930	tttcgtttgt	1	TC7628	27	5	similar to SP|P41091|IF2G_HUMAN Eukaryotic translation initiation factor 2 subunit 3 (Eukaryotic translation initiation factor 2 gamma subunit) (eIF-2-gamma). [Human] {Homo sapiens}, partial (93%)
8930	tttcgtttgt	1	CD066271	22	2	
8931	tacgtacacc	1				no_annot
8932	aagaacgtga	1				no_annot
8933	tggttttctc	1				no_annot
8934	tgtttgtttg	1				no_annot
8935	tccgcttgaa	1				no_annot
8936	gggatttctg	1				no_annot

8937	aagtaccaga	1				no_annot
8938	cctaatgtgt	1				no_annot
8939	tgtacattgc	1				no_annot
8940	cgaaactcag	1				no_annot
8941	ttttacaatt	1	TC14166	6	2	
8942	gctacccgat	1				no_annot
8943	aacattctca	1				no_annot
8944	ttatcattta	1				no_annot
8945	ttataagtcc	1				no_annot
8946	ataacgtaca	1	TC13077	50	1	
8947	ctcgaagctg	1				no_annot
8948	acaaatgatt	1				no_annot
8949	taatatgcgt	1				no_annot
8950	tttcggccgt	1				no_annot
8951	tacgcattat	1				no_annot
8952	tgccggttca	1				no_annot
8953	ccacaccgca	1				no_annot
8954	aatgtttatt	1				no_annot
8955	gccactagaa	1				no_annot
8956	tggtgaaatg	1				no_annot
8957	gtccgcattg	1				no_annot
8958	gcataatact	1				no_annot
8959	taacaggggt	1				no_annot
8960	tattcacttc	1	TC11383	22	0	similar to SP|P49964|SR19_ORYSA Signal recognition particle 19 kDa protein (SRP19). [Rice] {Oryza sativa}, partial (11%)
8961	acccacaact	1				no_annot
8962	ccaacatcga	1	TC9249	50	1	
8963	actaaaatga	1	CD089265	29	1	
8964	attaagtagt	1	TC8605	39	2	

8965	aagctatggt	1	TC14008	53	1	similar to GP|6288792|gb|AAF06740.1|AF169627_1|AF169627 Galpha16 {Oryctolagus cuniculus}, partial (19%)
8965	aagctatggt	1	N20761	56	0	
8966	atccaactgt	1				no_annot
8967	acaacaatat	1	CD083836	45	0	weakly similar to GP|28974767|gb|AAO53282.1||AY242996 NADH dehydrogenase subunit 4L {Antheraea pernyi}, partial (22%)
8968	aattttaatt	1				no_annot
8969	tccaagcctt	1				no_annot
8970	ttgtatttgt	1	TC13315	60	0	similar to GP|21645185|gb|AAF46686.2||AE003453 CG4266-PA {Drosophila melanogaster}, partial (6%)
8970	ttgtatttgt	1	TC15179	71	0	
8971	ccaatttcct	1				no_annot
8972	tattaaaggg	1				no_annot
8973	caacttatat	1				no_annot
8974	gccggcgtct	1				no_annot
8975	aatgcaggta	1				no_annot
8976	tgtttgatgt	1	TC13648	58	6	homologue to GP|832859|gb|AAA67384.1||L32580 presumptive protein 3 {Sceloporus grammicus}, partial (19%)
8977	attattttgt	1				no_annot
8978	atacgctaca	1	CD146027	17	0	similar to GP|21668043|gb|AAM74201.1|AF521017_1|AF521017 SOCIUS protein isoform 1 {Homo sapiens}, partial (5%)
8979	acaacaataa	1	TC11220	23	5	similar to GP|5533003|gb|AAD44976.1|AF157028_1|AF157028 protein phosphatase methylesterase-1 {Homo sapiens}, partial (19%)
8980	gatacggggc	1	TC11350	66	1	weakly similar to GP|21064815|gb|AAM29637.1||AY113632 RH72196p {Drosophila melanogaster}, partial (67%)
8981	tcaaattgta	1				no_annot
8982	atattggtaa	1	CD066721	86	0	
8983	ggctggaatc	1				no_annot

8984	cactaactta	1				no_annot
8985	gagttataaa	1				no_annot
8986	ctccgaggac	1				no_annot
8987	aaagttcatc	1	C301435.1	50	1	
8987	aaagttcatc	1	C604812.1	11	5	
8987	aaagttcatc	1	C611003.1	70	1	
8988	ttcaaatcaa	1	TC9145	1	1	weakly similar to GP|11991531|emb|CAC19674.1||AJ304845 RalB-binding protein {Xenopus laevis}, partial (6%)
8989	tgatagactg	1	CD066240	51	1	
8990	aatatttgaa	1	CD069377	63	1	
8991	gtattgattg	1	TC10665	34	4	similar to GP|11095909|gb|AAG30114.1|AF285758_1|AF285758 lysyl-tRNA synthetase {Homo sapiens}, partial (36%)
8992	cgcggccggg	1	C612227.1	91	0	
8993	ttgttttaaa	1				no_annot
8994	cttctgaata	1				no_annot
8995	agttgtgcta	1				no_annot
8996	gtaacaatga	1				no_annot
8997	acacatacgg	1				no_annot
8998	tttaggtcgt	1				no_annot
8999	cagtgtgccg	1				no_annot
9000	acagctccgt	1				no_annot
9001	tggcttaatg	1	TC7853	22	2	weakly similar to GP|6010208|emb|CAB57276.1||X77725 PIG-A {Homo sapiens}, partial (38%)
9002	ttgtcatact	1	C208818.1	28	3	
9003	gaaagtaatg	1				no_annot
9004	taccctcgcg	1				no_annot
9005	gcatttagat	1				no_annot
9006	atcaacaaca	1				no_annot
9007	gtgttagtca	1	C205353.1	98	0	
9008	acgtacgtgt	1				no_annot

9009	ttacccgttt	1				no_annot
9010	taatgcaaag	1	TC12267	7	5	weakly similar to GP|17946230|gb|AAL49155.1||AY071533 RE57556p {Drosophila melanogaster}, partial (12%)
9010	taatgcaaag	1	AW063002	19	1	
9011	gactcacggt	1				no_annot
9012	ttgtttcgta	1	TC10823	40	0	
9013	cggtgcgcta	1				no_annot
9014	aagtcgtgat	1				no_annot
9015	tcgttttgtt	1				no_annot
9016	cactcaatac	1				no_annot
9017	agacacctaa	1				no_annot
9018	gcaggtggtc	1	TC13521	88	1	similar to GP|7300349|gb|AAF55508.1||AE003721 CG7187-PA {Drosophila melanogaster}, partial (5%)
9019	cacgtacaag	1				no_annot
9020	cagtattgag	1				no_annot
9021	aagtgtgtgt	1				no_annot
9022	acttggagag	1				no_annot
9023	gttgggtggt	1				no_annot
9024	tcccgtactc	1				no_annot
9025	attgttgttg	1	AI395158	44	1	
9026	tttcgtttca	1				no_annot
9027	gtggtgggaa	1				no_annot
9028	gcgttcaatt	1				no_annot
9029	ttgctcctat	1				no_annot
9030	ttgttccgaa	1	TC15324	14	2	weakly similar to GP|18480026|gb|AAL61027.1||AY073364 olfactory receptor MOR23-1 {Mus musculus}, partial (7%)
9031	agtcgagtgt	1				no_annot
9032	cagcgccata	1				no_annot
9033	gcgttcaatg	1				no_annot
9034	gaagacatac	1				no_annot

9035	ctaatgaatt	1				no_annot
9036	tatgttctat	1				no_annot
9037	cagactactg	1	C205353.1	87	1	
9037	cagactactg	1	C208281.1	31	1	
9038	atgtgaaatt	1	CD082166	76	0	
9039	ccagaaatat	1				no_annot
9040	ttgtggtagc	1				no_annot
9041	tgatacgctt	1				no_annot
9042	tcctggccca	1	C602615.1	70	0	
9043	tagttatttg	1				no_annot
9044	agggacgacc	1				no_annot
9045	gtgacataac	1	C606075.1	24	0	
9046	aacgcaagtt	1				no_annot
9047	tttgtataca	1				no_annot
9048	aagaacgtaa	1	TC14321	15	3	
9049	actgcttcct	1				no_annot
9050	atgagtacac	1				no_annot
9051	atttcacgct	1				no_annot
9052	gacaagggat	1				no_annot
9053	aaatgcactg	1				no_annot
9054	aaaagtcatt	1				no_annot
9055	gggattaccg	1				no_annot
9056	tgtaaactgg	1				no_annot
9057	tgagaaaagt	1	TC17828	1	3	
9058	tagtacatta	1	TC18603	78	1	
9059	ggtatgatca	1				no_annot
9060	cgcggccgcg	1				no_annot
9061	cctactatgg	1				no_annot
9062	aagtctaaat	1				no_annot
9063	gacttgtttg	1	C302089.1	82	1	

9064	gtaacaatca	1	AY623659|Sma.1226	74	2	Sm50 protein
9065	acaacattga	1	C200370.1	86	1	
9065	acaacattga	1	C608406.1	85	1	
9066	gacaagtact	1				no_annot
9067	cactgaactt	1	TC15923	14	0	
9067	cactgaactt	1	CD061115	10	0	
9068	gcaatgacag	1				no_annot
9069	ctgatcttct	1	C611600.1	85	0	
9070	agggcaatat	1				no_annot
9071	cgagaatata	1				no_annot
9072	taacaccatc	1				no_annot
9073	tcataatttc	1				no_annot
9074	aaagatcttt	1	TC14175	51	0	similar to SP|Q92620|PR16_HUMAN Pre-mRNA splicing factor ATP-dependent RNA helicase PRP16 (ATP- dependent RNA helicase DDX38) (DEAH-box protein 38). [Human] {Homo sapiens}, partial (14%)
9075	ttttcggtgt	1	CD160234	87	0	similar to GP|5597017|gb|AAC24982.2||AF025672 reverse transcriptase {synthetic construct}, partial (4%)
9076	cagaaaggat	1				no_annot
9077	ttttaaatgt	1	BF936299	18	0	
9078	tggatcagtc	1				no_annot
9079	ccgctcttat	1	CD140090	3	0	
9080	tggcacatca	1				no_annot
9081	ggttaaacaa	1				no_annot
9082	tgaccttcat	1				no_annot
9083	aatcctccgg	1	C608846.1	24	6	
9084	ttttttgctt	1	C206571.1	62	0	
9085	cacacttgca	1	C203006.1	0	10	
9086	ttatatgata	1				no_annot
9087	ttatcaatat	1				no_annot
9088	tttctgggtt	1	TC10335	92	0	
9089	aaaatgatct	1	TC7493	56	1	

9089	aaaatgatct	1	TC14241	15	5	weakly similar to SP|Q92Q54|EFTS_RHIME Elongation factor Ts (EF-Ts). [Sinorhizobium meliloti] {Rhizobium meliloti}, partial (15%)
9089	aaaatgatct	1	TC18911	37	0	
9089	aaaatgatct	1	CD178860	53	0	
9090	aaaatcgtgg	1				no_annot
9091	atcgtcatca	1				no_annot
9092	cgaagagcat	1				no_annot
9093	gttattgctg	1	C602175.1	21	0	
9094	ctgtcacgtt	1				no_annot
9095	cgagaaaagt	1				no_annot
9096	ttgcctgata	1	TC14024	94	0	similar to GP|16226747|gb|AAL16250.1|AF428320_1|AF428320 AT5g05080/MUG13_6 {Arabidopsis thaliana}, partial (32%)
9097	tcatcggtgt	1				no_annot
9098	ataaagcaaa	1				no_annot
9099	gtttcctcac	1	TC10389	54	1	weakly similar to GP|16769662|gb|AAL29050.1||AY061502 LD46084p {Drosophila melanogaster}, partial (32%)
9100	tttggagata	1	TC10111	89	0	
9101	tatgcactaa	1				no_annot
9102	ctgattttta	1				no_annot
9103	aaccgttagt	1	C211377.1	66	0	
9103	aaccgttagt	1	C609185.1	69	0	
9104	tttaggtcat	1	CD083646	46	2	weakly similar to GP|20378335|gb|AAM20910.1|AF435959_1|AF435959 tetratricopeptide repeat-containing protein isoform B {Homo sapiens}, partial (25%)
9105	gaattcaacg	1				no_annot
9106	tatactcgga	1				no_annot
9107	acacatacac	1	AI067546	94	0	similar to PIR|H70112|H70112 periplasmic serine proteinase DO (htrA) homolog - Lyme disease spirochete, partial (4%)
9108	attacgagca	1				no_annot

9109	tagaaacgtt	1				no_annot
9110	caacagggat	1	TC16689	78	0	
9110	caacagggat	1	TC19691	44	0	
9110	caacagggat	1	AI395378	63	0	
9110	caacagggat	1	CD073199	30	1	
9110	caacagggat	1	CD156053	78	1	
9111	cactctaata	1	TC16959	74	0	weakly similar to GP|20977028|gb|AAM33247.1||AF419152 mitotic phosphoprotein 39 {Xenopus laevis}, partial (18%)
9112	atcgttatta	1	TC11060	70	3	similar to GP|2766687|gb|AAC69992.1||AF036955 tetraspanin TE736 {Schistosoma japonicum}, partial (8%)
9112	atcgttatta	1	TC18056	49	0	
9113	cccgagatgt	1				no_annot
9114	ctttaaatgt	1				no_annot
9115	cattcccata	1				no_annot
9116	tagaggtgta	1				no_annot
9117	tctgttacta	1				no_annot
9118	gcgcctgcct	1				no_annot
9119	aaataaacaa	1	TC10056	8	1	
9120	cattaaagag	1				no_annot
9121	catctatagc	1				no_annot
9122	ataaatacat	1				no_annot
9123	tactgtgttt	1				no_annot
9124	gcaataaaaa	1	TC13815	90	0	weakly similar to GP|25990819|gb|AAN76709.1|AF443827_1|AF443827 acheron {Manduca sexta}, partial (12%)
9124	gcaataaaaa	1	TC13816	37	2	similar to GP|25990819|gb|AAN76709.1|AF443827_1|AF443827 acheron {Manduca sexta}, partial (5%)
9125	cttgtataaa	1	TC9854	58	0	
9126	gaatttaatc	1				no_annot
9127	gttcagggac	1				no_annot
9128	agaaggaagt	1				no_annot

9129	tcgtaataca	1	TC7369	42	2	weakly similar to PIR|A36226|A36226 collagen alpha 1 chain - sea urchin (Paracentrotus lividus), partial (3%)
9130	gccctcaccc	1				no_annot
9131	tctactatag	1				no_annot
9132	tgacgttagt	1				no_annot
9133	atgatggtat	1				no_annot
9134	taatctacct	1				no_annot
9135	tggggagggc	1				no_annot
9136	ataaaatgac	1				no_annot
9137	tccgaaactt	1				no_annot
9138	ctatcaataa	1				no_annot
9139	aacactcata	1	TC8825	39	1	weakly similar to GP|7292570|gb|AAF47970.1||AE003484 CG1637-PA {Drosophila melanogaster}, partial (29%)
9140	gataagcaga	1				no_annot
9141	agaaggaaga	1				no_annot
9142	tggaagactt	1				no_annot
9143	tgtacttatt	1	TC10190	25	2	homologue to GP|14276277|gb|AAK58179.1|AF222566_1|AF222566 NADH dehydrogenase subunit 2 {Tragopan temminckii}, partial (6%)
9143	tgtacttatt	1	TC11448	7	0	
9143	tgtacttatt	1	CD093991	77	0	similar to SP|O97159|CHDM_DROME Chromodomain helicase-DNA-binding protein Mi-2 homolog (dMi-2). [Fruit fly] {Drosophila melanogaster}, partial (3%)
9144	ttcttgcata	1				no_annot
9145	cggtggtctc	1				no_annot
9146	ttaagacaca	1				no_annot
9147	tcattgtcgt	1				no_annot
9148	caaaactatg	1				no_annot
9149	tttgaattca	1				no_annot
9150	cggtgaggtc	1	C202903.1	61	0	
9150	cggtgaggtc	1	C602263.1	59	0	
9151	gttacggtct	1				no_annot

9152	attcgtagta	1	TC7060	15	7	weakly similar to SP|Q9UL36|Z236_HUMAN Zinc finger protein 236. [Human] {Homo sapiens}, partial (3%)
9152	attcgtagta	1	TC17157	2	0	weakly similar to GP|25004946|emb|CAB03018.2||Z81072 C. elegans STL-1 protein (corresponding sequence F30A10.5) {Caenorhabditis elegans}, partial (78%)
9153	cagacaagcg	1				no_annot
9154	gtgaaacaag	1				no_annot
9155	acacgcagca	1				no_annot
9156	taaccattca	1	C200940.1	33	2	
9156	taaccattca	1	C610121.1	20	3	
9157	caattttgat	1				no_annot
9158	atggattgtt	1				no_annot
9159	ctgggccaga	1				no_annot
9160	tgtgttgtgt	1				no_annot
9161	tgtgaggaga	1	TC8363	59	1	similar to EGAD|26444|27285 G/T mismatch binding protein {Homo sapiens}, partial (7%)
9162	tcgtaataac	1				no_annot
9163	gaatcaaatt	1				no_annot
9164	agaatttagg	1				no_annot
9165	acattgatca	1	TC9767	7	0	
9165	acattgatca	1	TC10696	56	0	
9165	acattgatca	1	TC15013	83	0	
9165	acattgatca	1	TC15901	41	2	
9165	acattgatca	1	CD093942	16	3	
9165	acattgatca	1	CD162395	80	1	
9165	acattgatca	1	CD200685	74	0	weakly similar to GP|19067879|gb|AAK14815.1||AY027869 polyprotein {Schistosoma japonicum}, partial (6%)
9166	ggcgttcgtg	1	AF254148|Sma.785	35	2	PUR-alpha-like protein (PUR-alpha)
9167	ttatcattgt	1				no_annot
9168	tcggcgcaac	1				no_annot
9169	gtaacattgt	1				no_annot

9170	ggatcatcac	1				no_annot
9171	cgagaaaaca	1				no_annot
9172	atacgtgggc	1				no_annot
9173	ttgggccact	1				no_annot
9174	gaattattct	1				no_annot
9175	ctgaacgagt	1				no_annot
9176	cagttaatct	1				no_annot
9177	tttaattgga	1	C201546.1	19	2	
9177	tttaattgga	1	C604464.1	17	2	
9177	tttaattgga	1	C610972.1	23	2	
9178	atcacagcca	1				no_annot
9179	ttcaatctga	1	C600221.1	63	0	
9180	gtaacattga	1				no_annot
9181	ggaaaaggtc	1	TC11822	42	1	
9182	taaatgtcaa	1				no_annot
9183	aagcctgtca	1				no_annot
9184	aatcctccaa	1				no_annot
9185	tggtgaaaga	1				no_annot
9186	cgtaaactat	1	CD076347	25	0	
9187	ttacttacta	1				no_annot
9188	tccaatttga	1				no_annot
9189	ctgattatct	1				no_annot
9190	ttgattatag	1	C313794.1	92	0	
9190	ttgattatag	1	C700663.1	85	0	
9191	ttgattataa	1	U83906|Sma.649	45	2	Putative cytosol aminopeptidase
9192	taaatataca	1	TC11370	86	1	weakly similar to GP|16768382|gb|AAL28410.1||AY060862 GM03430p {Drosophila melanogaster}, partial (56%)
9193	gtgtcctttc	1				no_annot
9194	tcagaattaa	1	TC8638	50	2	
9195	tccgcctttt	1				no_annot

9196	gaaatcattg	1				no_annot
9197	gctcagtgtg	1	C211455.1	0	0	
9198	gtttgcggaa	1	TC14513	45	0	
9199	aggtagaaat	1				no_annot
9200	gaaatcattc	1	TC17808	96	0	similar to GP|7292702|gb|AAF48099.1||AE003487 CG15741-PA {Drosophila melanogaster}, partial (15%)
9201	cttgcagcga	1				no_annot
9202	ttgttcgttc	1	CD154075	6	2	weakly similar to GP|25058380|gb|AAH40012.1||BC040012 myotubularin related protein 6 {Homo sapiens}, partial (17%)
9203	ttttccagta	1				no_annot
9204	agaacccctt	1				no_annot
9205	tcatacaagg	1				no_annot
9206	tcattgtcct	1				no_annot
9207	tgctttattt	1	TC10502	30	8	similar to GP|2661460|emb|CAA05921.1||AJ003165 ABI3 {Populus balsamifera subsp. trichocarpa}, partial (3%)
9207	tgctttattt	1	TC13944	58	2	similar to GP|1943772|gb|AAB52431.1||U97191 Rab family protein 2 {Caenorhabditis elegans}, partial (89%)
9208	gcaacggtag	1				no_annot
9209	agagaaactc	1	TC18249	77	0	similar to SP|Q9WUA2|SYFB_MOUSE Phenylalanyl-tRNA synthetase beta chain(Phenylalanine--tRNA ligase beta chain) (PheRS). [Mouse] {Mus musculus}, partial (26%)
9210	aaaatgttgg	1	TC11039	13	0	weakly similar to SP|Q06136|FVT1_HUMAN Follicular variant translocation protein 1 precursor (FVT-1). [Human] {Homo sapiens}, partial (12%)
9210	aaaatgttgg	1	TC15673	38	0	
9211	acgggaagta	1	C201166.1	14	2	
9212	ggatgatagt	1				no_annot
9213	gacagccttc	1	TC12896	21	1	weakly similar to GP|10121007|pdb|1EPU|A Chain A, X-Ray Crystal Structure Of Neuronal Sec1 From Squid, partial (4%)
9214	tatcgctttc	1				no_annot
9215	ctcaatctgc	1				no_annot
9216	tctttcacga	1	TC14538	69	4	

9217	tctgtaagcc	1	C314506.1	4	0	
9217	tctgtaagcc	1	C707202.1	3	0	
9218	tgtagaaccc	1				no_annot
9219	gtctcccagt	1				no_annot
9220	tgatttagtt	1	TC13678	79	0	similar to GP|1817731|gb|AAB42184.1||L36118 low-density lipoprotein receptor {Chiloscyllium plagiosum}, partial (3%)
9221	caaatgtcac	1				no_annot
9222	ttgagtcgaa	1				no_annot
9223	tggtcatcgc	1				no_annot
9224	atgtagctat	1				no_annot
9225	caaccattaa	1	M99494|Sma.23	77	4	200 kDa surface protein
9226	ttggtcgtgt	1	TC11505	16	7	
9227	tactgatcta	1				no_annot
9228	agtaaaaaaa	1				no_annot
9229	ggacggaaga	1				no_annot
9230	aatatagata	1	C300493.1	34	0	
9231	attacttatg	1				no_annot
9232	tcgtcctcct	1				no_annot
9233	taaaataatc	1				no_annot
9234	agctgctcct	1				no_annot
9235	tatatatgtt	1	C201636.1	15	2	
9235	tatatatgtt	1	C208491.1	92	0	
9235	tatatatgtt	1	C611787.1	14	2	
9236	gatattctat	1				no_annot
9237	tccgacgtat	1	TC13879	97	0	weakly similar to GP|7295350|gb|AAF50668.1||AE003563 CG10173-PA {Drosophila melanogaster}, partial (9%)
9238	actggttaat	1				no_annot
9239	tcccgtacgt	1				no_annot
9240	tgtcgttgtt	1				no_annot
9241	cagcgtgacg	1				no_annot

9242	gttgtctttc	1				no_annot
9243	gccatcgagt	1	TC17033	36	1	weakly similar to GP|5002178|gb|AAD37345.1|AF141864_1|AF141864 AtaAp {Emericella nidulans}, partial (23%)
9243	gccatcgagt	1	TC17034	71	0	weakly similar to SP|Q99943|PLCA_HUMAN 1-acyl-sn-glycerol-3-phosphate acyltransferase alpha(1-AGP acyltransferase 1) (1-AGPAT 1) (Lysophosphatidic acid acyltransferase-alpha) (LPAAT-alpha) (1-acylglycerol-3-phosphate O- acyltransferase 1) (G15 protein). [Human] {Homo sapiens}, partial (16%)
9244	gctgcccaca	1				no_annot
9245	taacgatctg	1				no_annot
9246	cgacgttacc	1				no_annot
9247	gaaaaacgct	1	TC8605	63	1	
9248	ctgttcgttc	1	TC14267	0	2	similar to GP|21668098|gb|AAM74228.1|AF520418_1|AF520418 lactation elevated 1 {Homo sapiens}, partial (20%)
9249	taaaacaaac	1				no_annot
9250	tttttataca	1				no_annot
9251	ccatcggtac	1				no_annot
9252	gcagtagata	1				no_annot
9253	tccgacgtac	1	TC10780	40	3	similar to GP|24415014|emb|CAD54736.1||AJ512486 core alpha-6-fucosyltransferase {Caenorhabditis elegans}, partial (13%)
9254	attaaactac	1	C206085.1	76	1	
9255	gatagcaggg	1				no_annot
9256	agtagatttg	1	AI740244	70	1	
9257	aaactgctca	1				no_annot
9258	aatatctttc	1	TC12896	12	2	weakly similar to GP|10121007|pdb|1EPU|A Chain A, X-Ray Crystal Structure Of Neuronal Sec1 From Squid, partial (4%)
9258	aatatctttc	1	BF440133	95	0	
9259	taatcttcgt	1	CD076682	36	1	similar to PIR|T44596|T44596 26S proteasome regulatory complex chain p50 [imported] - fruit fly (Drosophila melanogaster), partial (8%)
9260	gttaacaact	1				no_annot

9261	ttcaatctca	1	TC18164	61	1	similar to GP|21429070|gb|AAM50254.1||AY119600 LD21416p {Drosophila melanogaster}, partial (9%)
9262	cttcccatta	1				no_annot
9263	tataacagta	1				no_annot
9264	atagaaaaaa	1	C210382.1	82	0	
9265	ctcagctcca	1	C716699.1	42	1	
9266	ggaaaaaagt	1	TC15747	85	0	
9267	tattcactca	1				no_annot
9268	gagcaaacga	1				no_annot
9269	gtttgtaagt	1				no_annot
9270	gtggaaacaa	1				no_annot
9271	tggatctgta	1				no_annot
9272	gattaaaatg	1				no_annot
9273	tgcaataaac	1				no_annot
9274	atgctgtgga	1	TC18006	82	0	
9275	aatgtttaca	1				no_annot
9276	cgagtctgat	1	CD122474	12	0	
9277	agaagggggt	1				no_annot
9278	tacattgata	1				no_annot
9279	caaaataatc	1				no_annot
9280	caaatataac	1				no_annot
9281	aaatctatcc	1	TC12343	67	1	weakly similar to GP|21106778|gb|AAM35558.1||AE011695 tail-specific protease {Xanthomonas axonopodis pv. citri str. 306}, partial (3%)
9282	gacaaactac	1	TC10910	38	1	
9283	tagagcggcc	1				no_annot
9284	aaaatatcta	1	CD092963	64	1	
9285	catatatgtg	1				no_annot
9286	ttgcgagcgt	1				no_annot
9287	cggtggacaa	1				no_annot
9288	atgaaaaatg	1				no_annot

9289	catcaattca	1				no_annot
9290	catatatgta	1				no_annot
9291	taataacgag	1				no_annot
9292	tggctgtgac	1				no_annot
9293	cttgactacg	1				no_annot
9294	tactcccgtt	1				no_annot
9295	ttcctctatc	1	CD180963	50	1	weakly similar to GP|28972842|dbj|BAC65837.1||AK122555 mKIAA1781 protein {Mus musculus}, partial (5%)
9296	tggcacttat	1				no_annot
9297	gaagttgggg	1				no_annot
9298	ccacagtttt	1	TC11105	71	0	weakly similar to GP|7296288|gb|AAF51578.1||AE003591 CG5649-PA {Drosophila melanogaster}, partial (45%)
9299	ccgaaaatgt	1	C709325.1	93	0	
9300	taaaaattcg	1	TC17532	70	0	
9301	tacgctggct	1				no_annot
9302	agcatcctaa	1				no_annot
9303	aatgtccgcg	1				no_annot
9304	ccttcgtttg	1	C201078.1	43	1	
9304	ccttcgtttg	1	C606050.1	40	2	
9305	agcaaatgtt	1				no_annot
9306	caagaagttc	1				no_annot
9307	tgatagacat	1				no_annot
9308	cagttattga	1	TC12359	10	2	
9309	gtggtccacc	1	TC15772	14	0	
9310	tttctactta	1	C200554.1	62	0	
9310	tttctactta	1	C707059.1	54	0	
9311	caggcaggta	1				no_annot
9312	aatggtgatg	1	TC17390	37	2	
9313	gaattttatc	1	TC10873	90	0	similar to GP|2258465|gb|AAB94003.1||AF008588 succinyl-CoA synthetase alpha subunit {Sus scrofa}, partial (70%)

9314	agagacgtag	1				no_annot
9315	taaaagagat	1				no_annot
9316	tcatacaaaa	1	TC8889	9	1	homologue to GP|15042221|gb|AAK82001.1|AF303741_79|AF303741 079L {Chilo iridescent virus}, partial (18%)
9317	tcgcttttct	1				no_annot
9318	atacttattt	1				no_annot
9319	ataaattcaa	1				no_annot
9320	ctttacaaat	1				no_annot
9321	aagatccacc	1	AF303222|Sma.1081	10	11	SNaK1
9322	ctaggaacag	1				no_annot
9323	tgtgcagtaa	1				no_annot
9324	ttgttgtttt	1	C604525.1	66	0	
9325	gtagccgatg	1	TC17183	98	0	similar to SP|P51892|DNL1_XENLA DNA ligase I(Polydeoxyribonucleotide synthase [ATP]). [African clawed frog] {Xenopus laevis}, partial (39%)
9326	cgcatacgag	1				no_annot
9327	cggtgtgctc	1				no_annot
9328	attttgttcg	1				no_annot
9329	gtttggtgac	1	TC7391	40	3	
9330	gtaactggta	1				no_annot
9331	gtgttactgg	1				no_annot
9332	gagctactat	1	AF071011|Sma.779	19	4	Myosin light chain
9333	attcgttgtg	1	CD096435	79	1	
9334	gaaagtaaaa	1	CD186847	71	0	
9335	attcgttgtc	1				no_annot
9336	tattgtagta	1	TC11327	72	1	weakly similar to SP|Q9SMI3|RS12_CYAPA 40S ribosomal protein S12. {Cyanophora paradoxa}, partial (72%)
9337	cataagcttg	1				no_annot
9338	ttgtttcgag	1				no_annot

9339	gaatcgacgt	1	TC16682	35	4	similar to SP|P38646|GR75_HUMAN Stress-70 protein, mitochondrial precursor (75 kDa glucose regulated protein) (GRP 75) (Peptide-binding protein 74) (PBP74) (Mortalin) (MOT). [Human] {Homo sapiens}, partial (89%)
9340	ggctcaggat	1				no_annot
9341	aagagaagtc	1				no_annot
9342	cacaagatat	1				no_annot
9343	tcgttttgat	1	TC18326	61	0	
9344	attgaggaac	1				no_annot
9345	aatatcatct	1				no_annot
9346	ggctcgtcaa	1				no_annot
9347	caagttattt	1	C208754.1	4	3	
9348	acgatgatat	1				no_annot
9349	tgttgtcaaa	1	TC11143	10	2	
9350	atgcacttca	1	CD126143	39	0	
9351	attaaccaag	1				no_annot
9352	atcgtacatt	1	TC10593	47	1	weakly similar to SP|Q9JJT0|RCL1_MOUSE RNA 3'-terminal phosphate cyclase-like protein. [Mouse] {Mus musculus}, partial (45%)
9353	ttccaagatt	1	TC7370	13	1	weakly similar to GP|24659630|gb|AAH39185.1||BC039185 Tcerg1 protein {Mus musculus}, partial (10%)
9353	ttccaagatt	1	TC7376	82	0	weakly similar to GP|24659630|gb|AAH39185.1||BC039185 Tcerg1 protein {Mus musculus}, partial (22%)
9354	aatgcataaa	1	TC13159	86	0	
9355	agtgaagatc	1				no_annot
9356	aatgaacaga	1				no_annot
9357	tcatacggga	1				no_annot
9358	gacgtcctgt	1				no_annot
9359	tttaacattg	1	CD088545	38	0	
9360	gttgttattt	1	C304242.1	48	1	
9361	ttccaagatg	1				no_annot

9362	tatcattatt	1	CD180703	24	3	similar to GP|21645360|gb|AAM70960.1||AE003809 CG30087-PA {Drosophila melanogaster}, partial (8%)
9363	ttggtgttgc	1				no_annot
9364	gaagttggca	1	C608869.1	92	0	
9365	atgtattgta	1				no_annot
9366	aataagaatg	1	CD161578	38	0	
9367	taatcttcaa	1	C605600.1	53	0	
9368	tactatcata	1	TC7932	92	0	
9369	ctgacattgt	1	C207803.1	87	0	
9369	ctgacattgt	1	C604164.1	84	0	
9370	gttgttatta	1				no_annot
9371	tgataaaaaa	1	CD094031	69	2	similar to PIR|T39903|T39903 serine-rich protein - fission yeast (Schizosaccharomyces pombe), partial (5%)
9372	tcgaaaataa	1				no_annot
9373	cactgaacca	1				no_annot
9374	aggtccaaat	1				no_annot
9375	taacgtgtgg	1	CD074923	97	0	
9376	ggaaaaaaaa	1				no_annot
9377	ttaattgatt	1	CD080225	10	2	
9378	ggggacaact	1				no_annot
9379	aatattattc	1				no_annot
9380	ttttactaga	1				no_annot
9381	gtttcttccc	1				no_annot
9382	ggtcgaatgg	1				no_annot
9383	cagtacatct	1				no_annot
9384	gaactgttca	1	BG930302	53	0	
9385	ggcaccaatc	1				no_annot
9386	tttagcccaa	1				no_annot
9387	gcagttattt	1				no_annot
9388	ggggagaggt	1	C304527.1	23	2	
9388	ggggagaggt	1	C711945.1	23	2	

9389	tatataagaa	1				no_annot
9390	atagcaaggt	1				no_annot
9391	ggacttcgta	1	CD137247	16	0	
9392	taacaccaat	1				no_annot
9393	ggctaacagt	1				no_annot
9394	taaaactact	1				no_annot
9395	ccatacggga	1				no_annot
9396	tgcgcaatat	1				no_annot
9397	aaaattgtgt	1	CD194392	5	0	
9398	acttgtggaa	1				no_annot
9399	tgactcttct	1	C201116.1	48	0	
9399	tgactcttct	1	C608266.1	31	0	
9400	tcctcctttt	1	TC8094	93	0	similar to GP|4039014|gb|AAC97420.1||AF037338 cleft lip and palate transmembrane protein 1 {Homo sapiens}, partial (36%)
9401	ttatatgaat	1	TC12461	36	5	similar to GP|19715609|gb|AAL91627.1||AY075613 AT3g59630/T16L24_180 {Arabidopsis thaliana}, partial (5%)
9402	tgattctgca	1				no_annot
9403	caatgtgggg	1				no_annot
9404	tcatactaga	1				no_annot
9405	caaaatggtt	1				no_annot
9406	cactgaacaa	1				no_annot
9407	aattttggct	1	C702240.1	4	2	
9408	ccgatgtcct	1				no_annot
9409	agcaactcga	1				no_annot
9410	gaagggaaag	1	AF026805|Sma.730	69	1	Fructose bisphosphate aldolase
9411	cagttgagaa	1	C604864.1	7	3	
9412	caattagata	1				no_annot
9413	gtttcttcaa	1				no_annot
9414	cagctgatac	1				no_annot
9415	tgttcagtgc	1	CD162633	36	0	

9416	ctggcagtta	1				no_annot
9417	gttttagatg	1	TC16874	97	0	weakly similar to GP|4050087|gb|AAC97961.1||AF109907 S164 {Homo sapiens}, partial (26%)
9417	gttttagatg	1	CD082061	42	3	
9418	taagtggcca	1				no_annot
9419	aaaaaaagta	1				no_annot
9420	aaaataacaa	1	TC12316	11	2	
9420	aaaataacaa	1	CD155976	75	1	
9421	gggttcgcat	1				no_annot
9422	tctgacagca	1	TC14683	54	3	similar to GP|7300349|gb|AAF55508.1||AE003721 CG7187-PA {Drosophila melanogaster}, partial (22%)
9423	ggtacgtatg	1	TC12854	73	0	
9424	cagtacataa	1				no_annot
9425	ataataaatg	1	C211161.1	10	3	
9426	tgatgaatgg	1	TC19634	14	3	similar to GP|1736829|dbj|BAA15973.1||D90848 Histidine-rich glycoprotein precursor. {Escherichia coli}, partial (5%)
9427	atttgtcaat	1				no_annot
9428	actccttagg	1				no_annot
9429	tgacttttta	1	C200312.1	25	2	
9429	tgacttttta	1	C711705.1	22	2	
9430	tggtggtcaa	1				no_annot
9431	tggacaagtt	1				no_annot
9432	cgacggattc	1	TC11456	45	2	
9433	tactctgggg	1				no_annot
9434	gtaacgtgcg	1				no_annot
9435	taatatccca	1				no_annot
9436	ggtcccacaa	1				no_annot
9437	cgcttttttt	1	C606337.1	43	1	
9438	atttaggagg	1				no_annot
9439	ggttttcaca	1				no_annot

9440	attcgtagca	1				no_annot
9441	tagctcgtaa	1				no_annot
9442	ccttcgatac	1				no_annot
9443	ttgaaactga	1	TC11200	20	1	similar to GP|10798668|emb|CAC12827.1||AJ250180 beta thymosin {Oncorhynchus mykiss}, partial (51%)
9444	ccgccgagag	1	CD134023	34	0	weakly similar to GP|21465649|pdb|1IRU|H Chain H, Crystal Structure Of The Mammalian 20s Proteasome At 2.75 A Resolution, partial (59%)
9445	acacctcagc	1				no_annot
9446	gaagcaggaa	1				no_annot
9447	gatacgcgca	1				no_annot
9448	gggtgcattg	1				no_annot
9449	gtgtttgaat	1	TC9653	94	0	GP|23305766|gb|AAN17273.1||AF521088 DYRK2-like protein {Schistosoma mansoni}, complete
9450	tcactgggaa	1	CD059771	22	0	
9451	ccgtgttatg	1				no_annot
9452	tatgaaattt	1				no_annot
9453	tttatctatc	1				no_annot
9454	ttaacctttg	1				no_annot
9455	atataaaaaa	1				no_annot
9456	tcaggttttg	1				no_annot
9457	aacgatgcat	1				no_annot
9458	aaaacagtga	1				no_annot
9459	gcttctcatc	1				no_annot
9460	atttaaagtg	1				no_annot
9461	tgtttatcgc	1				no_annot
9462	catatgcagt	1				no_annot
9463	tccctcagta	1				no_annot
9464	tacgtcactc	1				no_annot
9465	cttgtacgat	1				no_annot
9466	ttctttaccg	1				no_annot
9467	caaacgctgt	1				no_annot

9468	tcttggactc	1	TC13987	67	0	
9469	ttgaatgaga	1				no_annot
9470	tatattacgc	1	TC18089	79	0	
9471	caggcaaaca	1				no_annot
9472	gttgtggcca	1				no_annot
9473	atcttcttct	1				no_annot
9474	ttttattata	1	TC18860	30	0	
9475	tacactatta	1				no_annot
9476	tataccottc	1	C202257.1	19	3	
9476	tataccottc	1	C601186.1	18	4	
9477	ctgacattat	1				no_annot
9478	cctgatagtt	1	TC10920	65	1	
9479	ggaaaaggct	1	TC18268	79	1	
9480	tccgaaacac	1				no_annot
9481	aaaggatgcc	1				no_annot
9482	gagatttata	1	TC11806	75	1	
9482	gagatttata	1	CD194008	21	2	
9482	gagatttata	1	AW063017	21	1	
9483	tacaccatat	1	CD093145	31	2	SP|P20287|G3P_SCHMA Glyceraldehyde 3-phosphate dehydrogenase(GAPDH) (Major larval surface antigen) (P-37). [Blood fluke] {Schistosoma mansoni}, partial (23%)
9484	ttatgtgtga	1				no_annot
9485	tatacatctt	1				no_annot
9486	tgaccatttt	1				no_annot
9487	taagccaatg	1				no_annot
9488	gtctgctgac	1				no_annot
9489	tgcttacata	1				no_annot
9490	gatgaatagt	1	AY354457|Sma.72	57	2	Putative neuropeptide receptor
9491	caaaactaaa	1				no_annot
9492	gaaaatccaa	1				no_annot
9493	tgttcagtct	1				no_annot

9494	atattctgct	1				no_annot
9495	gtgatgataa	1	TC14537	83	0	
9496	acggcacagt	1				no_annot
9497	aaccacgagt	1				no_annot
9498	acgcaaaagc	1				no_annot
9499	aagcattggt	1				no_annot
9500	tctgcaacat	1				no_annot
9501	cattattcat	1				no_annot
9502	tgaataggta	1				no_annot
9503	gagtattaat	1	TC11644	53	0	
9504	tgtgcccatt	1	TC13945	23	3	
9505	ccgaaattgc	1				no_annot
9506	aaaaaggaat	1				no_annot
9507	aacatatatt	1	C611930.1	73	2	
9508	aaatatctgt	1	TC16867	58	0	
9508	aaatatctgt	1	CD134416	63	3	homologue to GP|1103829|gb|AAC46967.1||U31768 elastase {Schistosoma mansoni}, partial (5%)
9509	atcaatggaa	1				no_annot
9510	cgtttatcgg	1				no_annot
9511	tctttttctc	1	C609439.1	91	0	
9512	tgctaagtaa	1				no_annot
9513	cgcatagttt	1				no_annot
9514	ggtcaataat	1				no_annot
9515	caataagttg	1				no_annot
9516	atcttcttat	1	BG930261	46	0	similar to GP|17946034|gb|AAL49060.1||AY071438 RE52392p {Drosophila melanogaster}, partial (26%)
9517	aagttggact	1				no_annot
9518	atttcagtga	1	CD133865	6	0	
9518	atttcagtga	1	AI395642	72	0	
9519	aactgaataa	1				no_annot
9520	tatgtgtctg	1				no_annot

9521	gaatcaaaac	1	TC7537	73	0	similar to GP|7300667|gb|AAF55815.1||AE003732 CG15697-PA {Drosophila melanogaster}, partial (44%)
9521	gaatcaaaac	1	TC9588	96	0	
9522	gaatcaaaaa	1				no_annot
9523	cgttttgtaa	1				no_annot
9524	aagagcaccc	1				no_annot
9525	gcaattcctt	1				no_annot
9526	ctatgtgtga	1	CD080974	97	0	
9527	ggggttgccg	1				no_annot
9528	gcatatccca	1				no_annot
9529	gcttgctgga	1				no_annot
9530	gcaaactaaa	1				no_annot
9531	atgaacgcgt	1	TC17349	44	0	similar to SP|P48444|COPD_HUMAN Coatomer delta subunit (Delta-coat protein) (Delta-COP) (Archain). [Human] {Homo sapiens}, partial (26%)
9532	ctggccgatt	1	C201100.1	86	0	
9533	ttgtttgttt	1				no_annot
9534	catcgggcac	1				no_annot
9535	ttactatgat	1	TC15743	72	0	
9536	atgtggtcag	1				no_annot
9537	attctaggtg	1	C205386.1	95	0	
9538	aaatgaagaa	1				no_annot
9539	taaaataact	1				no_annot
9540	gtgtttcatt	1				no_annot
9541	taattcatat	1	TC13409	73	0	
9542	tagacttcaa	1				no_annot
9543	caggtttagt	1	C205208.1	62	4	
9544	cctagtacca	1	C210348.1	10	3	
9544	cctagtacca	1	C608695.1	10	4	
9545	tttatgtgtt	1	TC7847	10	3	
9546	gtgctgctgc	1	TC17341	33	7	
9547	tcttttacgt	1	C210596.1	94	0	

9548	ggaagaatat	1				no_annot
9549	aactaataac	1				no_annot
9550	tcatataaga	1				no_annot
9551	caagaagtgg	1				no_annot
9552	tgttcgaaca	1				no_annot
9553	tcattcccaa	1				no_annot
9554	tgatgaatac	1				no_annot
9555	ttgacttata	1	TC14143	41	0	
9556	ataactcact	1				no_annot
9557	ttgttcgtag	1				no_annot
9558	agcaaatgga	1				no_annot
9559	ttacagctct	1				no_annot
9560	tgttttcttc	1				no_annot
9561	gaatatatat	1	AA999265	2	1	
9562	gactttgtct	1	CD072955	53	0	similar to GP|9944727|gb|AAG02910.1|AF250284_204|AF250284 AMV204 {Amsacta moorei entomopoxvirus}, partial (17%)
9563	acgataacgg	1				no_annot
9564	tatatatgca	1				no_annot
9565	aacatcgcgt	1				no_annot
9566	atttaggaag	1				no_annot
9567	caaaagtgat	1				no_annot
9568	tgagtttgat	1				no_annot
9569	ctgaggttcg	1				no_annot
9570	acaatggata	1	C210043.1	30	2	
9571	aatcaatgtt	1	CD090084	75	0	
9572	tacacagaga	1	C612432.1	72	1	
9573	aggcaagcga	1				no_annot
9574	ctgaaactcg	1	TC11441	59	0	similar to GP|22217757|emb|CAD23135.1||AJ430531 light regulation of gametogenesis6 protein {Chlamydomonas reinhardtii}, partial (4%)

9575	tttaacttta	1				no_annot
9576	ttgaaccaga	1	TC13773	32	3	
9577	aataaagtgc	1				no_annot
9578	tttatcaact	1	TC17234	93	1	weakly similar to GP|28569596|gb|AAO42744.1||AY198392 mitochondrial transcription factor b1 {Rattus norvegicus}, partial (23%)
9579	ttgttgttgt	1	TC12628	42	1	
9580	tttgatggaa	1	AI882670	68	0	
9581	acgcaaaacc	1				no_annot
9582	tcaaaagatg	1	C200155.1	3	2	
9582	tcaaaagatg	1	C200494.1	33	0	
9582	tcaaaagatg	1	C201807.1	40	3	
9582	tcaaaagatg	1	C202321.1	95	0	
9583	tagtattttt	1				no_annot
9584	aggctttctt	1				no_annot
9585	ggttgatggt	1				no_annot
9586	ttccttggtg	1				no_annot
9587	tttgattact	1	TC11965	28	0	
9588	atttttgtac	1				no_annot
9589	caatttatta	1				no_annot
9590	aatatttttt	1	C601744.1	67	1	
9591	gtgggaaggg	1				no_annot
9592	aattatttcc	1	TC9178	83	1	weakly similar to PIR|A46498|A46498 glucocorticoid-sensitive T cell-specific protein 30 - mouse, partial (5%)
9592	aattatttcc	1	TC15769	62	1	
9593	tcacagttcc	1				no_annot
9594	ttcaagactc	1	C611947.1	85	0	
9595	cttcaatctg	1	C611494.1	87	0	
9596	ccgcaccagc	1				no_annot
9597	cctcacttta	1				no_annot
9598	tgacttatat	1				no_annot

9599	taaaataaac	1	M27294|Sma.59	17	11	3-hydroxy-3-methylglutaryl coenzyme A reductase
9600	atcgaccata	1				no_annot
9601	agtctgtttc	1				no_annot
9602	gtgatgttga	1				no_annot
9603	gatggcaaga	1	TC18777	74	0	
9604	gatctggcca	1				no_annot
9605	taatcaatta	1				no_annot
9606	gttttcatat	1				no_annot
9607	caattcataa	1				no_annot
9608	tttattaatt	1				no_annot
9609	agtagattat	1				no_annot
9610	gaatggaaca	1				no_annot
9611	gaaacaatta	1	C305094.1	27	3	
9612	ggtcgtaata	1	AI975241	17	0	
9613	tggcatttcg	1				no_annot
9614	tgttacacaa	1	CD201453	92	0	
9615	tgattaccag	1				no_annot
9616	tacatccagc	1				no_annot
9617	aatatgccta	1				no_annot
9618	tggcatttca	1				no_annot
9619	gtcatttcgt	1				no_annot
9620	ttttcgctag	1				no_annot
9621	ggaataccag	1	TC10364	50	2	weakly similar to GP|1575609|gb|AAC50893.1||U69127 FUSE binding protein 3 {Homo sapiens}, partial (6%)
9622	aggtatgaat	1				no_annot
9623	ttcttttcgg	1				no_annot
9624	ctttcgctcg	1				no_annot
9625	cgattaccca	1				no_annot
9626	tatgtaaagt	1	CD080019	36	0	
9627	tggagaatga	1				no_annot
9628	taattagagt	1				no_annot

9629	caggttggac	1				no_annot
9630	aactgattga	1	CD117818	3	0	
9631	gcatttattg	1	TC10453	21	4	weakly similar to GP|2636680|gb|AAC06263.1||U66331 pol {Schistosoma mansoni}, partial (3%)
9631	gcatttattg	1	CD187895	42	0	
9631	gcatttattg	1	BF936070	34	3	
9632	tagggacttc	1				no_annot
9633	tgatatgcgc	1				no_annot
9634	tgttccgaca	1				no_annot
9635	acacacaatt	1	C210670.1	85	0	
9636	ctccttggtc	1				no_annot
9637	atatagtcaa	1				no_annot
9638	aaggtaagac	1	C202032.1	32	3	
9639	ctgttgttgg	1	TC10758	26	1	similar to GP|403456|gb|AAA35484.1||L02426 26S protease (S4) regulatory subunit {Homo sapiens}, partial (94%)
9640	cttcagttat	1				no_annot
9641	actattccgg	1				no_annot
9642	tcaattaatt	1				no_annot
9643	tgtctcgccc	1	TC7565	54	2	
9644	atgctttaat	1				no_annot
9645	cagtattttg	1				no_annot
9646	caaaataaag	1	TC13929	49	0	weakly similar to GP|6470251|gb|AAF13696.1|AF195141_1|AF195141 selenoprotein T {Homo sapiens}, partial (48%)
9647	tacacggcaa	1				no_annot
9648	taaaatgggc	1				no_annot
9649	tcaatattga	1				no_annot
9650	caccactgct	1				no_annot
9651	ggccactcag	1	TC7593	32	0	
9652	ctgcattcac	1	TC8988	47	3	weakly similar to GP|13195570|gb|AAK15762.1|AF329820_1|AF329820 cornichon {Boltenia villosa}, partial (93%)

9653	atgtgtgcga	1	TC16980	66	0	weakly similar to SP|O43791|SPOP_HUMAN Speckle-type POZ protein. [Human] {Homo sapiens}, partial (28%)
9654	atgaaaaaat	1	TC10845	10	5	
9654	atgaaaaaat	1	TC11891	13	1	weakly similar to GP|2231617|gb|AAB62067.1||U87243 Sm 20.8 {Schistosoma mansoni}, partial (29%)
9654	atgaaaaaat	1	T14415	13	1	
9655	cagtacttat	1				no_annot
9656	ttgagaacca	1	TC13479	81	2	homologue to PIR|B54525|B54525 major female-specific polypeptide (frame 2) - fluke (Schistosoma mansoni) (fragment), partial (95%)
9657	tctttgcaaa	1				no_annot
9658	gtggcacagg	1				no_annot
9659	taccgaaaac	1				no_annot
9660	gagagagtgt	1	TC7546	5	5	homologue to GP|16904828|gb|AAL30898.1||AF438343 phosphoglycerate mutase {Schistosoma japonicum}, complete
9661	ttggttgtct	1				no_annot
9662	tgatggaggc	1				no_annot
9663	actttcttcg	1				no_annot
9664	tgtcttgctg	1				no_annot
9665	agtagtggtt	1	C301200.1	69	0	
9666	aggcaagcca	1				no_annot
9667	cgcagacagc	1				no_annot
9668	tgatgattgc	1				no_annot
9669	gagacaaact	1				no_annot
9670	ttaaagttac	1	TC14489	82	0	
9671	aataaagtca	1				no_annot
9672	tggtgtgcac	1				no_annot
9673	tcagattaag	1				no_annot
9674	tgatgtaatg	1				no_annot
9675	tatacaacaa	1	TC8791	89	0	
9675	tatacaacaa	1	AI974215	58	0	

9676	gacttcctct	1				no_annot
9677	gtgtctatca	1				no_annot
9678	tgtgttctct	1	TC13937	95	1	weakly similar to GP|16197825|gb|AAL13552.1||AY058323 GH09295p {Drosophila melanogaster}, partial (21%)
9679	gagaggtata	1	CD155417	82	0	similar to PIR|S12791|SYHUTP CTP synthase- human, partial (13%)
9680	ctaatactga	1	CD192157	92	0	
9681	tgaataaact	1	TC15761	74	2	weakly similar to SP|P01122|RHO_APLCA RAS-like GTP-binding protein RHO. [California sea hare] {Aplysia californica}, partial (70%)
9682	ttgactaact	1				no_annot
9683	aaaagggtcg	1				no_annot
9684	gccacggcct	1				no_annot
9685	actgacgaag	1				no_annot
9686	ttttgttttc	1	TC17226	80	1	
9687	attgattctt	1				no_annot
9688	tgaagttgaa	1				no_annot
9689	gttagttttg	1				no_annot
9690	cagagtggca	1				no_annot
9691	ttgcgttgtc	1	TC16864	87	1	similar to GP|4886558|emb|CAB43352.1||AJ005937 lamin {Tealia sp.}, partial (7%)
9692	atctttatag	1				no_annot
9693	atccgtctca	1				no_annot
9694	gaatcgtcat	1				no_annot
9695	aatggtgaat	1				no_annot
9696	atataataat	1	TC8224	13	6	
9696	atataataat	1	AA999587	44	0	
9697	ggataggctt	1				no_annot
9698	agcaaatgac	1	C210299.1	28	3	
9699	attgcgtaaa	1				no_annot
9700	tatgtgacag	1				no_annot
9701	cagtttaaaa	1				no_annot

9702	tctcatattc	1	C207273.1	94	0	
9702	tctcatattc	1	C605099.1	94	0	
9703	ctcaaaaata	1				no_annot
9704	tactatcact	1	CD075674	49	0	
9705	catacaacat	1				no_annot
9706	acaaatcact	1				no_annot
9707	tgatatgcct	1				no_annot
9708	tacaatgtcg	1				no_annot
9709	acttgcgttt	1				no_annot
9710	ggctgtgacc	1				no_annot
9711	cttgatcgtg	1				no_annot
9712	caccctcttc	1				no_annot
9713	tatggttgac	1				no_annot
9714	tttggtttgc	1	TC13187	97	0	similar to GP|18652299|gb|AAL77055.1|AF467986_1|AF467986 ADP-ribosylation factor-like protein 3 {Xenopus laevis}, partial (82%)
9715	acaaatcacg	1				no_annot
9716	taaacttgtt	1				no_annot
9717	tgatatgccg	1				no_annot
9718	gggcttgata	1				no_annot
9719	gagactgctc	1				no_annot
9720	aagattcaat	1				no_annot
9721	acttttatgt	1				no_annot
9722	atgcatttcg	1	C200799.1	3	4	
9722	atgcatttcg	1	C300184.1	80	0	
9722	atgcatttcg	1	C704248.1	48	0	
9722	atgcatttcg	1	C701798.1	80	0	
9723	ggaggagtta	1				no_annot
9724	agtttattcg	1				no_annot
9725	acatatactt	1				no_annot
9726	gggatatttt	1				no_annot

9727	aaatgtacca	1	C210994.1	97	0	
9727	aaatgtacca	1	C608741.1	98	0	
9728	accttaaact	1				no_annot
9729	ccgaactaca	1				no_annot
9730	gagaatatcg	1				no_annot
9731	tttatattat	1				no_annot
9732	aaatgatgcc	1				no_annot
9733	tatcgtatag	1				no_annot
9734	tgaataaaaa	1	TC7989	40	3	weakly similar to GP|16768252|gb|AAL28345.1||AY060797 GH26345p {Drosophila melanogaster}, partial (23%)
9735	tatactgtcg	1				no_annot
9736	caagagaaaa	1	TC17593	15	5	
9737	tttaacatat	1	TC12270	75	0	
9738	tttccagaca	1				no_annot
9739	aacgaacgaa	1	TC11089	64	2	similar to SP|P79101|CPSC_BOVIN Cleavage and polyadenylation specificity factor, 73 kDa subunit (CPSF 73 kDa subunit). [Bovine] {Bos taurus}, partial (60%)
9740	aaattggtga	1	AY463158|Sma.1184	69	4	Actin-binding/filamin-like protein
9741	atgaaccctt	1				no_annot
9742	ttcttttcaa	1				no_annot
9743	gcattcttga	1				no_annot
9744	tacaatgtac	1				no_annot
9745	taagcaatat	1				no_annot
9746	agtttattat	1				no_annot
9747	aaccggatag	1	C302645.1	38	0	
9748	taggatttaa	1	TC8511	43	0	
9749	caagcaatct	1	TC12216	75	0	
9750	tcaatctagt	1				no_annot
9751	atcagtttat	1				no_annot
9752	tcgactagtt	1				no_annot
9753	ttgggggtgt	1				no_annot

9754	attcaaatga	1				no_annot
9755	cttcttgaag	1	TC12423	74	0	weakly similar to GP|23477189|emb|CAD22046.1||AJ428954 tissue factor pathway inhibitor-2 {Rattus norvegicus}, partial (11%)
9756	atgcatttag	1				no_annot
9757	gacaatcaaa	1				no_annot
9758	tatgtaaaaa	1	TC14371	41	5	weakly similar to GP|13872813|emb|CAC37630.1||AJ306906 fibulin-6 {Homo sapiens}, partial (3%)
9759	aacaactatc	1	TC11632	15	3	weakly similar to GP|3702848|gb|AAC62955.1||AF073333 reverse transcriptase SjR1 {Schistosoma japonicum}, partial (23%)
9760	gaaatagaag	1	TC13909	81	0	weakly similar to GP|29342364|gb|AAO80130.1||AE016947 cell division protein FtsH {Enterococcus faecalis V583}, partial (16%)
9761	atttccgaat	1				no_annot
9762	atagtcattt	1				no_annot
9763	ttgggggtga	1				no_annot
9764	attacgattt	1				no_annot
9765	ataccaatac	1				no_annot
9766	tgctccaaaa	1				no_annot
9767	ttacattgtc	1				no_annot
9768	tcaaacatag	1				no_annot
9769	ataaaatttc	1				no_annot
9770	ttcatagccg	1				no_annot
9771	taacatcaaa	1				no_annot
9772	agttgagttc	1				no_annot
9773	cgtataagta	1				no_annot
9774	gtgtgatgtg	1				no_annot
9775	gtttggattc	1				no_annot
9776	attggctgtg	1				no_annot
9777	agaatgatca	1				no_annot
9778	atattttggt	1				no_annot

9779	tgacccaaag	1	TC12244	5	1	
9780	ggtaactcct	1				no_annot
9781	catcttccca	1	CD073646	71	0	
9782	cttaacatac	1				no_annot
9783	acactaaaag	1				no_annot
9784	gtcttcgcgt	1				no_annot
9785	acttttatct	1	CD097335	56	0	
9786	gtaactcgta	1				no_annot
9787	tatagaaact	1				no_annot
9788	acgtgaagat	1				no_annot
9789	gcgtcaaaaa	1				no_annot
9790	taatagaaga	1	TC11245	71	2	similar to GP|7297705|gb|AAF52957.1||AE003628 CG5322-PA {Drosophila melanogaster}, partial (5%)
9791	aacaaaatac	1				no_annot
9792	agttttggtt	1				no_annot
9793	aataatgatt	1	TC18079	15	3	
9794	tttctccaat	1	TC7333	46	5	similar to GP|3309068|gb|AAC26004.1||AF072935 small GTP-binding protein rab5 {Rattus norvegicus}, partial (73%)
9795	aattcccccc	1				no_annot
9796	agcacccaca	1				no_annot
9797	aattgtgagc	1	CD064747	71	0	
9798	gttgcaatat	1				no_annot
9799	caaaatggag	1	CD189562	21	0	
9800	gtagaaagaa	1	CD135919	74	0	
9801	tgtatctcta	1	TC17403	94	0	
9802	tcgtttattg	1				no_annot
9803	cagccagtgc	1	TC11931	96	0	weakly similar to SP|Q9R0E1|PLO3_MOUSE Procollagen-lysine,2-oxoglutarate 5-dioxygenase 3 precursor(Lysyl hydroxylase 3) (LH3). [Mouse] {Mus musculus}, partial (12%)
9804	cgctggatac	1				no_annot

9805	ttggaatgtt	1	TC12681	22	2	similar to GP|26105953|gb|AAN78331.1||AC137988 TcC31.4 {Trypanosoma cruzi}, partial (8%)
9806	aaattagcta	1				no_annot
9807	atgaaaggat	1				no_annot
9808	aatcttctgt	1	TC17723	67	2	GP|22122171|dbj|BAC07174.1||AB084278 prepro-Neuropeptide W polypeptide {Rattus norvegicus}, partial (6%)
9809	tcgtttatta	1	TC8796	43	1	
9809	tcgtttatta	1	CD137125	3	5	homologue to GP|7290448|gb|A CG15239-PA {Drosophila melanogaster}, partial (2%)
9810	acaggctgtg	1	TC16172	20	0	
9811	ataattgctt	1				no_annot
9812	acagttctca	1	TC14470	45	1	
9813	attgaatgca	1				no_annot
9814	cttctccaca	1				no_annot
9815	tgacagtaaa	1	TC14530	59	0	
9815	tgacagtaaa	1	CD119208	38	1	
9816	gacgctgagg	1				no_annot
9817	tctaacgctt	1				no_annot
9818	cattctcaag	1				no_annot
9819	tatttatatg	1				no_annot
9820	ccctatgtct	1				no_annot
9821	gtgctttgtg	1				no_annot
9822	gggtgtattt	1	TC14523	22	0	similar to SP|P41891|GAR2_SCHPO Protein gar2. [Fission yeast] {Schizosaccharomyces pombe}, partial (6%)
9823	ggtcaacgga	1	TC10783	51	1	similar to GP|5833465|gb|AAD53521.1|AF155581_1|AF155581 proteasome subunit beta 7 {Danio rerio}, partial (75%)
9824	ataataaaaa	1				no_annot
9825	taaatagttt	1	TC17551	64	1	similar to SP|P13716|HEM2_HUMAN Delta-aminolevulinic acid dehydratase(Porphobilinogen synthase) (ALADH). [Human] {Homo sapiens}, partial (40%)
9826	ggaagtggtt	1				no_annot

9827	taaggaacta	1				no_annot
9828	tacactatca	1	DQ068274|Sma.1988	57	0	Protein arginine N-methyltransferase 1 (PRMT1)
9829	ttaacttttt	1	CD189291	47	0	
9830	aagaatctac	1				no_annot
9831	taaattccat	1	C202111.1	35	0	
9831	taaattccat	1	C605926.1	34	0	
9832	cagacaattc	1				no_annot
9833	tttgggccat	1				no_annot
9834	taaatagttg	1				no_annot
9835	tgaaaaataa	1	TC12181	41	2	weakly similar to GP|3581828|emb|CAA04390.1||AJ000917 O. volvulus cyclophilin 4 {Onchocerca volvulus}, partial (52%)
9835	tgaaaaataa	1	CD084621	49	0	
9836	tgggacatct	1	TC11657	88	0	similar to PIR|JC4365|JC4365 arginine-tRNA ligase-human, partial (8%)
9837	ggtctcattt	1				no_annot
9838	ttaacttttg	1				no_annot
9839	atacgcgtgc	1				no_annot
9840	tggacaagac	1				no_annot
9841	caatttatgt	1	TC14047	78	0	weakly similar to GP|17862566|gb|AAL39760.1||AY069615 LD37956p {Drosophila melanogaster}, partial (7%)
9842	gataacaata	1				no_annot
9843	cttcgaagta	1	C208329.1	77	0	
9843	cttcgaagta	1	C609380.1	77	0	
9844	tttaacttgc	1				no_annot
9845	tatagtgcta	1				no_annot
9846	tataactgaa	1	TC16697	35	1	
9846	tataactgaa	1	TC16719	71	4	GP|160960|gb|AAA29867.1||M86397 epidermal growth factor receptor {Schistosoma mansoni}, partial (5%)
9846	tataactgaa	1	CD132163	41	0	similar to GP|160960|gb|AAA29867.1||M86397 epidermal growth factor receptor {Schistosoma mansoni}, partial (5%)
9846	tataactgaa	1	CD146838	6	3	

9847	aactgttatt	1				no_annot
9848	ttattcactg	1				no_annot
9849	ccactgcggt	1				no_annot
9850	atcacttgat	1	CD202376	17	0	
9851	atttggcttg	1				no_annot
9852	ctcaaaggtt	1	CD145764	84	0	
9852	ctcaaaggtt	1	CD078202	60	0	
9853	attaccgtta	1				no_annot
9854	ttttttcccg	1	TC7881	64	1	
9855	ccttgcgcgt	1				no_annot
9856	aataagtaga	1				no_annot
9857	agtgggaagg	1				no_annot
9858	gtttgcgtta	1				no_annot
9859	aaccaactga	1				no_annot
9860	agccatccag	1				no_annot
9861	tacactatat	1				no_annot
9862	gatcagaaag	1				no_annot
9863	gcttctcaag	1	TC7719	78	3	weakly similar to GP|16769502|gb|AAL28970.1||AY061422 LD35289p {Drosophila melanogaster}, partial (18%)
9864	aacttcattt	1				no_annot
9865	tagtatttga	1				no_annot
9866	aaggaacctt	1				no_annot
9867	acttggtttc	1				no_annot
9868	aagctaatat	1				no_annot
9869	ggagaagacc	1	TC14194	84	1	
9870	gggtgggcgg	1				no_annot
9871	ggagaagaca	1				no_annot
9872	ttccggaagc	1				no_annot
9873	taatacgagc	1				no_annot
9874	taccgataag	1				no_annot

9875	aaaattctgt	1	gi|161049|gb|M74170.1|SCMP48EGG	36	11	Schistosoma mansoni p48 eggshell protein gene, complete cds
9876	ttcaaatatg	1	TC14064	19	7	similar to GP|25009985|gb|AAN71158.1||BT001403 GH07620p {Drosophila melanogaster}, partial (49%)
9877	atacctaata	1	TC8031	85	0	weakly similar to GP|18028086|gb|AAL55967.1|AF322903_1|AF322903 phospholipid hydroperoxide glutathione peroxidase {Raphanus sativus}, partial (49%)
9878	atacttttct	1	TC18243	33	1	weakly similar to SP|Q23237|PSB3_CAEEL Proteasome subunit beta type 3(Proteasome subunit beta 3). {Caenorhabditis elegans}, partial (19%)
9879	aggtctggca	1				no_annot
9880	catagaaaat	1	TC8872	33	1	
9880	catagaaaat	1	TC8872	33	0	
9881	ttaaatgtag	1	CD083929	82	0	
9881	ttaaatgtag	1	CD086658	80	0	
9882	gggataatat	1				no_annot
9883	cttatttatc	1	C610610.1	13	0	
9884	ggttgttcct	1				no_annot
9885	aacaacaaca	1	CD170809	1	0	homologue to GP|15553131|gb|AAL01649.1|AF247140_1|AF247140 voltage-dependent calcium channel alpha-2-delta-2 mutant subunit 1 {Mus musculus}, partial (8%)
9885	aacaacaaca	1	CD089947	43	0	homologue to SP|P33244|FTF1_DROME Nuclear hormone receptor FTZ-F1 (FTZ-F1 alpha). [Fruit fly] {Drosophila melanogaster}, partial (3%)
9885	aacaacaaca	1	CD151989	63	0	similar to GP|17016552|gb|AAL33158.1||AF332093 wsv154 {shrimp white spot syndrome virus} [Shrimp white spot syndrome virus], partial (10%)
9886	taaatgaatg	1				no_annot
9887	acagctgact	1	C211056.1	49	0	
9887	acagctgact	1	C603888.1	41	1	
9888	aacggttaga	1				no_annot
9889	tatacatcaa	1				no_annot
9890	tatgccagtc	1				no_annot
9891	agtagtaaaa	1				no_annot

9892	ctaagaagtt	1				no_annot
9893	tatgccagta	1				no_annot
9894	tgttcactgc	1				no_annot
9895	gtgaactctt	1				no_annot
9896	gagagtgata	1	TC7064	15	5	similar to SP|Q26540|1431_SCHMA 14-3-3 protein homolog 1. [Blood fluke] {Schistosoma mansoni}, partial (98%)
9897	acacaaatat	1				no_annot
9898	tctatctgta	1				no_annot
9899	tattgttgcg	1				no_annot
9900	gtcgataata	1	CD065628	49	1	
9901	cgcgatcatt	1	C208297.1	66	0	
9902	tgactacaag	1	TC11354	21	5	similar to GP|7293054|gb|AAF48440.1||AE003498 CG6146-PA {Drosophila melanogaster}, partial (3%)
9903	tatctcgata	1				no_annot
9904	tggacctcat	1				no_annot
9905	gcggtgatta	1				no_annot
9906	cacaaatcac	1				no_annot
9907	tttcatctta	1	TC16693	62	1	
9908	gtggtggtgg	1				no_annot
9909	aacaataata	1	CD194934	55	2	homologue to GP|7303490|gb|A CG13167-PA {Drosophila melanogaster}, partial (3%)
9910	ggcttgatac	1				no_annot
9911	aaaaatacgc	1				no_annot
9912	ttacggtccc	1				no_annot
9913	gctgcggtcc	1				no_annot
9914	acgatatcag	1				no_annot
9915	tgctctggtg	1	CD090183	25	0	
9916	gtcaatagac	1				no_annot
9917	ggcagccttt	1				no_annot
9918	ccgacctggt	1				no_annot
9919	agccgttaag	1				no_annot

9920	atacagtaag	1				no_annot
9921	attcagtgtt	1				no_annot
9922	atcccaactg	1				no_annot
9923	ggtgtagcac	1				no_annot
9924	agaggaagga	1	TC15936	57	3	
9924	agaggaagga	1	TC15936	57	2	
9925	gcttacaatg	1	CD085785	62	2	
9926	agtacttcat	1				no_annot
9927	ctatatcaat	1				no_annot
9928	catacatcat	1				no_annot
9929	tgtatcaccg	1	C315241.1	85	0	
9929	tgtatcaccg	1	C707231.1	77	0	
9930	gcaatagtta	1	CD157257	23	2	
9931	ataaaaatat	1				no_annot
9932	aagctgtgtc	1				no_annot
9933	gtgattgtat	1				no_annot
9934	gatcggatga	1				no_annot
9935	aagtgaaatg	1				no_annot
9936	cgacaactga	1				no_annot
9937	atcatagtca	1	C209628.1	29	2	
9937	atcatagtca	1	C610490.1	26	2	
9938	ttgtttgtaa	1	C205624.1	81	0	
9939	ctcctttagt	1				no_annot
9940	ataccgagca	1				no_annot
9941	cgactacaag	1				no_annot
9942	ctcatacta	1				no_annot
9943	tgaatataac	1				no_annot
9944	ggattcggct	1				no_annot
9945	gtagctgagt	1				no_annot
9946	tgaatataaa	1	C611772.1	73	2	

9947	tatgcgctta	1				no_annot
9948	tttatgtgaa	1	TC7762	20	2	
9948	tttatgtgaa	1	AW587551	5	2	
9949	tttaatttta	1	TC14248	76	2	weakly similar to GP|5670007|gb|AAD46560.1||AF156102 ELL complex EAP30 subunit {Homo sapiens}, partial (26%)
9950	cctcattttt	1				no_annot
9951	ataataggca	1	BF936772	54	1	
9952	ccttattgta	1				no_annot
9953	agttacttca	1	TC14217	88	1	
9953	agttacttca	1	TC15884	94	0	similar to GP|20521500|dbj|BAB92007.1||AP004641 B1147B04.21 {Oryza sativa (japonica cultivar-group)}, partial (3%)
9954	atttaatggt	1				no_annot
9955	aaaagctgtt	1	TC8108	30	3	
9955	aaaagctgtt	1	CD122393	17	6	
9956	ccttccctcc	1				no_annot
9957	attcacaaca	1				no_annot
9958	cctcattttg	1				no_annot
9959	ggacggcgaa	1				no_annot
9960	tggatttgca	1	CD083921	85	1	
9961	ggaaggtgca	1	TC17903	91	0	weakly similar to GP|22777405|dbj|BAC13678.1||AP004598 alcohol dehydrogenase {Oceanobacillus iheyensis}, partial (10%)
9962	tggagattac	1				no_annot
9963	tctcttaaaa	1				no_annot
9964	gaagtcgtgg	1	C208006.1	4	2	
9964	gaagtcgtgg	1	C606670.1	11	3	
9965	tattaaatga	1	TC12290	1	3	
9966	ggttcggatg	1				no_annot
9967	gaaaatgatc	1				no_annot
9968	atttgagcgc	1	C302165.1	12	3	
9968	atttgagcgc	1	C706694.1	12	3	

9969	tctaaaggta	1				no_annot
9970	aaacggtgag	1				no_annot
9971	gtgcaataac	1				no_annot
9972	tatgacaaag	1	TC13204	51	2	
9973	aggaaaacac	1				no_annot
9974	tcgattccga	1				no_annot
9975	ttagtatgta	1	TC14137	80	0	weakly similar to SP|P17152|PM1_HUMAN Protein PM1. [Human] {Homo sapiens}, partial (23%)
9976	gttgtgccac	1	C210727.1	47	0	
9977	cgaaacaaat	1	TC10360	27	9	weakly similar to GP|17158056|gb|AAA17976.2|AAA17976|U05040 FUSE binding protein {Homo sapiens}, partial (20%)
9977	cgaaacaaat	1	TC10404	28	5	weakly similar to GP|1575609|gb|AAC50893.1||U69127 FUSE binding protein 3 {Homo sapiens}, partial (18%)
9977	cgaaacaaat	1	TC10647	82	1	
9977	cgaaacaaat	1	CD123005	54	0	weakly similar to SP|Q8UVD9|ZBP2_CHICK Zipcode-binding protein 2. [Chicken] {Gallus gallus}, partial (9%)
9978	taatccaagc	1				no_annot
9979	agttacttac	1				no_annot
9980	aacgagaatg	1				no_annot
9981	gaaacaataa	1	C607899.1	19	6	
9982	atagtcttta	1	L31531|Sma.694	79	3	Phosphofructokinase (PFK)
9983	gtcgcagcgg	1	C207049.1	35	3	
9983	gtcgcagcgg	1	C605022.1	35	3	
9984	ggcttgttgt	1	TC17510	8	6	
9985	cgtatatgta	1				no_annot
9986	tgacaccagt	1				no_annot
9987	atcaagatgt	1				no_annot
9988	tagggactat	1				no_annot
9989	aatacattcc	1				no_annot
9990	catcggccac	1				no_annot
9991	ggagttgggg	1				no_annot

9992	ttgttatcca	1				no_annot
9993	tttgccattg	1				no_annot
9994	gtgattcttt	1				no_annot
9995	tttaaacatt	1				no_annot
9996	gtcctgaagc	1				no_annot
9997	gtcaattggc	1				no_annot
9998	attcgctttt	1				no_annot
9999	atctctggta	1				no_annot
10000	agacagacat	1	TC10674	38	5	
10001	ataaacaaca	1	TC10940	7	1	
10001	ataaacaaca	1	TC13419	34	2	similar to GP|19527767|gb|AAL89998.1||AY089260 AT04656p {Drosophila melanogaster}, partial (6%)
10002	ttcccgtcat	1				no_annot
10003	aatatacaca	1				no_annot
10004	aacaaattaa	1	TC14491	91	0	weakly similar to GP|5052630|gb|AAD38645.1|AF145670_1|AF145670 BcDNA.GH11690 {Drosophila melanogaster}, partial (4%)
10004	aacaaattaa	1	TC16958	9	9	
10004	aacaaattaa	1	CD080871	71	0	
10004	aacaaattaa	1	CD197571	36	1	
10005	ttttgtttca	1				no_annot
10006	aaataaaatg	1				no_annot
10007	ctaaacctac	1				no_annot
10008	aatgacactt	1				no_annot
10009	aatttcgtgt	1				no_annot
10010	taagctggtg	1				no_annot
10011	tctcctgctc	1				no_annot
10012	gatatgtcgg	1	TC7788	20	4	similar to GP|394780|emb|CAA50739.1||X71923 tenascin-X precursor {Homo sapiens}, partial (4%)
10013	gggtataaaa	1	TC17637	35	2	weakly similar to GP|3133134|dbj|BAA28173.1||AB001452 Sck {Rattus rattus}, partial (17%)

10013	gggtataaaa	1	CD160701	62	0	
10014	gtataaagag	1				no_annot
10015	ttgatcactc	1	C200260.1	30	4	
10015	ttgatcactc	1	C603239.1	14	7	
10015	ttgatcactc	1	C603681.1	30	4	
10016	gtttatgata	1	TC14525	87	0	
10016	gtttatgata	1	CD163691	12	0	
10017	gagtttgata	1				no_annot
10018	gtgtgatggt	1	M74233|Sma.65	46	6	Calcium-activated neutral proteinase (CANP)
10018	gtgtgatggt	1	gi|160936|gb|M67499.1|SCMCANPA	44	6	S.mansoni calcium-activated neutral proteinase (CANP) mRNA, complete cds
10019	ttggattctc	1				no_annot
10020	ttttcttgga	1	TC9129	78	1	
10021	gcagtacaca	1				no_annot
10022	aagccaaatg	1	C200154.1	28	0	
10022	aagccaaatg	1	C608925.1	27	0	
10023	aagatggtaa	1				no_annot
10024	gtgaaatgtt	1				no_annot
10025	aatttgatcc	1				no_annot
10026	tattcagcta	1				no_annot
10027	tttattgggc	1				no_annot
10028	tccagtcaaa	1	TC7178	83	1	
10029	cttattaaac	1	TC15917	16	2	
10030	tacactttcg	1				no_annot
10031	cttattaaaa	1	TC16894	68	1	similar to SP|P54727|R23B_HUMAN UV excision repair protein RAD23 homolog B (HHR23B) (XP-C repair complementing complex 58 kDa protein) (P58). [Human] {Homo sapiens}, partial (22%)
10032	aaagaaaagt	1	CD085226	24	2	
10033	aaccaccact	1				no_annot
10034	tgtacgatat	1				no_annot
10035	taaccgttta	1				no_annot

10036	taagcctact	1	TC7519	87	1	similar to SP|O08755|HNF6_MOUSE Hepatocyte nuclear factor 6 (HNF-6) (One cut domain family member 1). [Mouse] {Mus musculus}, partial (31%)
10037	gagatgattc	1				no_annot
10038	gtgaaatgta	1	TC9321	71	0	
10039	ataaacaaag	1	TC16925	39	3	weakly similar to GP|3005587|gb|AAC09321.1||AF048977 Ser/Arg-related nuclear matrix protein {Homo sapiens}, partial (20%)
10040	aacttagatt	1				no_annot
10041	gggtgtttta	1				no_annot
10042	gccattcagt	1	CD073369	93	0	
10043	ttttgtttag	1				no_annot
10044	tcgtagaagg	1				no_annot
10045	tggctgatct	1				no_annot
10046	gtatactccg	1				no_annot
10047	gaaagaactg	1	TC8330	8	7	weakly similar to GP|6594249|emb|CAB63561.1||AL031746 glutamic acid-rich protein (garp) {Plasmodium falciparum 3D7}, partial (6%)
10048	ttttgtttaa	1	CD085027	35	2	
10048	ttttgtttaa	1	CD085399	79	0	
10049	taatccaact	1	TC11820	49	4	
10049	taatccaact	1	TC16088	76	0	weakly similar to GP|21430852|gb|AAM51104.1||AY119244 SD20094p {Drosophila melanogaster}, partial (18%)
10050	cctcctgctt	1				no_annot
10051	gtttagtagt	1				no_annot
10052	ctgatcactt	1	TC10325	93	0	
10053	tatataattc	1	TC14362	18	3	weakly similar to GP|9963791|gb|AAG09695.1|AF183426_1|AF183426 HT004 protein {Homo sapiens}, partial (30%)
10054	ggattccgtt	1				no_annot
10055	tgcacctta	1				no_annot
10056	gcagtacaat	1				no_annot
10057	atctatgacg	1				no_annot

10058	atctggttat	1	TC19114	39	2	
10059	aataatgaga	1				no_annot
10060	tatttatagt	1	TC9682	4	4	weakly similar to PIR|I30010|I30010 NADH2 dehydrogenase (ubiquinone)chain 5 - Leishmania tarentolae mitochondrion, partial (4%)
10060	tatttatagt	1	AI395276	15	0	
10060	tatttatagt	1	CD116717	55	1	
10060	tatttatagt	1	CD095426	90	0	
10061	ttcaaagggg	1				no_annot
10062	ttatgtctaa	1	CD081283	79	1	
10063	gtccatcgat	1	C200386.1	46	1	
10063	gtccatcgat	1	C612175.1	62	1	
10064	tcttgaggat	1				no_annot
10065	tcgtaagtct	1	C601961.1	47	1	
10065	tcgtaagtct	1	C705477.1	28	0	
10066	ctcaaaaaaa	1				no_annot
10067	aaccatcatc	1				no_annot
10068	gaaacattgt	1				no_annot
10069	aggatccaac	1				no_annot
10070	gtttttttga	1				no_annot
10071	ccaagagtga	1				no_annot
10072	acaaacagtt	1	AA999630	74	0	
10073	ttcgtaatct	1	C606005.1	94	0	
10074	atatggtgat	1	TC15342	66	0	
10075	tttgtgaaag	1	TC12472	13	3	
10076	agacaaaaaa	1				no_annot
10077	ataggtggaa	1	TC17565	65	0	weakly similar to GP|28274786|gb|AAO34712.1||AY189675 dolichyl pyrophosphate phosphatase CWH8 {Homo sapiens}, partial (34%)
10077	ataggtggaa	1	TC17566	69	0	weakly similar to GP|28274786|gb|AAO34712.1||AY189675 dolichyl pyrophosphate phosphatase CWH8 {Homo sapiens}, partial (13%)

10078	gtcaattgcg	1				no_annot
10079	atttgagcat	1	AF030968|Sma.776	38	3	Clone 9 unknown
10080	cttcttcagt	1				no_annot
10081	aggactatgg	1				no_annot
10082	atcaagatcg	1				no_annot
10083	ggcgtcctgg	1				no_annot
10084	caaatgggtg	1				no_annot
10085	cagtggtaac	1				no_annot
10086	tgatcttcac	1				no_annot
10087	gaaacattga	1				no_annot
10088	atcaagatca	1	CD077308	63	0	similar to GP|14164385|dbj|BAB55793.1||AB061864 NCS homolog {Magnaporthe grisea}, partial (10%)
10089	ccaacaaaca	1				no_annot
10090	agtattattt	1	TC11875	20	4	
10090	agtattattt	1	TC15939	21	2	
10091	tgtgtaaata	1	CD085468	13	2	
10092	atggtctaac	1	TC7328	5	2	
10093	cgaaactagc	1				no_annot
10094	gaatttcgaa	1				no_annot
10095	atgatttgaa	1	TC18805	32	2	
10096	taccacattg	1	TC8714	51	2	
10096	taccacattg	1	TC17889	78	3	
10097	tgaaatggtg	1	AI976923	79	0	
10098	atacaccagt	1				no_annot
10099	tgaattgcag	1				no_annot
10100	gtttgaactt	1				no_annot
10101	cttcgaagga	1				no_annot
10102	aatagatctt	1				no_annot
10103	gcgagtcgga	1				no_annot
10104	taaaatcgga	1				no_annot

10105	gttatgggtc	1	TC19735	88	0	weakly similar to GP|25012781|gb|AAN71482.1||BT001727 RE69909p {Drosophila melanogaster}, partial (4%)
10106	taacacagtt	1				no_annot
10107	aataccggag	1				no_annot
10108	gtcatcaata	1				no_annot
10109	ccgtttatgg	1				no_annot
10110	acataatgac	1				no_annot
10111	ataaaattca	1	TC17752	84	0	
10111	ataaaattca	1	CD074585	45	3	
10112	ttaacgccga	1				no_annot
10113	ttaagaagga	1	C207957.1	94	0	
10113	ttaagaagga	1	C608223.1	91	0	
10114	gtgcacccgc	1				no_annot
10115	tttcctttcc	1				no_annot
10116	gggaacatac	1				no_annot
10117	tacacacaca	1				no_annot
10118	agtagtcgtt	1				no_annot
10119	cctcatataa	1	C610524.1	95	0	
10120	tatgatggtt	1	C605094.1	5	4	
10121	caaagacaac	1	TC18368	13	0	
10122	taatagtaat	1	C609091.1	96	0	
10123	cgatcttcat	1				no_annot
10124	atggaggcaa	1				no_annot
10125	attggctgcc	1	TC17186	20	3	SP|O01374|UCR6_SCHMA Ubiquinol-cytochrome C reductase complex 14 kDa protein. [Blood fluke] {Schistosoma mansoni}, complete
10126	cacttcacat	1				no_annot
10127	gtatatacga	1				no_annot
10128	ggattgtgat	1				no_annot
10129	agtccagtgt	1	TC8444	70	0	weakly similar to PIR|A57640|A57640 retinoblastoma binding protein RBQ-1 - human, partial (4%)

10130	ctcccataac	1				no_annot
10131	cttatttagt	1				no_annot
10132	tgcaagttga	1				no_annot
10133	cgattagatt	1	C201582.1	91	0	
10133	cgattagatt	1	C604741.1	78	1	
10134	tccggatgcc	1				no_annot
10135	aaagcggaca	1				no_annot
10136	gagttgggac	1	M86396|Sma.850	72	6	Epidermal growth factor receptor (SER, class 1 product)
10137	cactacagta	1				no_annot
10138	tgtaaagcgt	1				no_annot
10139	ttacattgat	1				no_annot
10140	caatggatat	1	TC7677	6	3	similar to GP|7677478|gb|AAF67180.1|AF241222_1|AF241222 mitochondrial import protein {Drosophila melanogaster}, partial (14%)
10141	ccagagatgt	1				no_annot
10142	tccaactctc	1				no_annot
10143	cttaatatat	1	C300274.1	88	1	
10144	taactaaggg	1				no_annot
10145	cgaattgcag	1				no_annot
10146	agcgtttggt	1				no_annot
10147	ttatcgtttt	1	TC18059	17	2	
10148	ttagctgttc	1				no_annot
10149	caatggatac	1				no_annot
10150	gtttttttct	1				no_annot
10151	gaacgagcag	1				no_annot
10152	tatttatact	1				no_annot
10153	cggctgataa	1				no_annot
10154	tttttgtatg	1				no_annot
10155	cagattataa	1				no_annot
10156	cacacacact	1				no_annot

10157	agaaaaagta	1	TC10471	34	12	similar to PIR|S05988|S05988 translation elongation factor eEF-2 - fruit fly (Drosophila melanogaster), partial (90%)
10158	ggtttgctga	1	TC12772	75	0	
10159	gaacgagcaa	1	TC11533	33	0	weakly similar to GP|17064956|gb|AAL32632.1||AY062554 nuclear receptor binding factor-like protein {Arabidopsis thaliana}, partial (11%)
10159	gaacgagcaa	1	TC17556	93	0	weakly similar to GP|3970880|dbj|BAA34804.1||AB015724 nuclear receptor binding factor-1 {Rattus norvegicus}, partial (19%)
10160	cgtactgttg	1	CD097959	56	0	
10161	gtagctcata	1				no_annot
10162	tgtatttctt	1				no_annot
10163	tttttgtata	1	CD152927	68	0	similar to GP|4958910|dbj| NADH dehydrogenase subunit 5 {Dictyostelium discoideum}, partial (2%)
10164	ttggagcaaa	1				no_annot
10165	ttccaacact	1	TC13817	29	2	similar to GP|25012819|gb|AAN71500.1||BT001745 RE74312p {Drosophila melanogaster}, partial (91%)
10166	tttacataga	1				no_annot
10167	catactctga	1				no_annot
10168	aatgttgtgt	1				no_annot
10169	tatggtatta	1				no_annot
10170	ccaacgtcgt	1				no_annot
10171	tttcctttaa	1				no_annot
10172	gttttttca	1				no_annot
10173	cacacacacc	1	C309708.1	74	1	
10174	aaagaaaaat	1	TC15732	8	1	
10174	aaagaaaaat	1	AI740394	80	0	
10175	agtacaatta	1				no_annot
10176	ataacctgtt	1				no_annot
10177	ccggctaatg	1				no_annot

10178	tttatttact	1	TC8898	93	0	similar to SP|Q09028|RBB4_HUMAN Chromatin assembly factor 1 subunit C (CAF-1 subunit C) (Chromatin assembly factor I p48 subunit) (CAF-I 48 kDa subunit) (CAF-Ip48) (Retinoblastoma binding protein p48) (Retinoblastoma-binding protein 4) (RBBP-4) (MSI1 protein homolog). [Human] {Homo sapiens}, partial (91%)
10178	tttatttact	1	CD127841	79	1	
10179	ttatctcgaa	1	CD152512	54	0	similar to GP|11602721|emb|CAC18547.1||AJ292373 spliceosome-associated-protein 114 {Echinococcus multilocularis}, partial (16%)
10180	tattcgcttg	1				no_annot
10181	ccctctttcc	1				no_annot
10182	gcgagtcgct	1				no_annot
10183	gggcttcatc	1				no_annot
10184	tgatgtggcg	1				no_annot
10185	gaaacattca	1	C302069.1	68	0	
10186	tattacaacg	1				no_annot
10187	ctgtgcagtt	1				no_annot
10188	ccgtcaatga	1				no_annot
10189	acaggttgtg	1				no_annot
10190	ggagtccgct	1	C604267.1	56	2	
10191	ggcactgggg	1				no_annot
10192	aactgtggag	1				no_annot
10193	aaataaggtt	1				no_annot
10194	aataatgaat	1	TC16112	9	0	
10194	aataatgaat	1	TC16248	90	0	
10195	ttcgtgtgtg	1				no_annot
10196	atctgaacat	1	C303857.1	87	0	
10196	atctgaacat	1	C601714.1	82	1	
10197	tttacaggca	1				no_annot
10198	aatgtaacac	1				no_annot
10199	ttattcaccg	1				no_annot
10200	tatgattatg	1				no_annot

10201	ttcaaaggat	1				no_annot
10202	gataagaggg	1	TC11140	55	2	homologue to GP|6692645|gb|AAF24788.1|U17009_20|U17009 NADH dehydrogenase subunit 3 {Phytophthora infestans}, partial (11%)
10203	aataatgaag	1				no_annot
10204	aaataaggta	1				no_annot
10205	tattataatt	1				no_annot
10206	aagtactggg	1				no_annot
10207	ttcgactttt	1				no_annot
10208	aaaggatttt	1				no_annot
10209	gataataatt	1	TC11892	65	2	similar to SP|Q9Y6D5|BIG2_HUMAN Brefeldin A-inhibited guanine nucleotide-exchange protein 2 (Brefeldin A-inhibited GEP 2). [Human] {Homo sapiens}, partial (3%)
10210	tatgcagcaa	1				no_annot
10211	tctaaaaaac	1				no_annot
10212	ggatttaaaa	1	TC11949	84	0	homologue to GP|6469624|gb|AAF13360.1||AF163839 N-Shc {Takifugu rubripes}, partial (5%)
10213	ctttcttgaa	1				no_annot
10214	tgttattcac	1				no_annot
10215	aaattgctgc	1	TC10588	77	1	
10216	tcaacgtcct	1	TC16558	6	2	weakly similar to PIR|T39903|T39903 serine-rich protein - fission yeast (Schizosaccharomyces pombe), partial (10%)
10216	tcaacgtcct	1	TC16559	36	3	similar to GP|4966317|gb|AAA88778.2||U46856 vitellogenin {Anolis pulchellus}, partial (7%)
10216	tcaacgtcct	1	CD094706	27	0	weakly similar to PIR|G71621|G71621 MAK16 homolog PFB0175c - malaria parasite (Plasmodium falciparum), partial (10%)
10217	ccgacaccat	1				no_annot
10218	caatcattct	1				no_annot
10219	ttggaatgaa	1				no_annot
10220	aattcagtta	1	C202256.1	53	1	
10220	aattcagtta	1	C716105.1	18	1	

10221	ttatcgatga	1				no_annot
10222	gtttttttaa	1				no_annot
10223	cacaggcagc	1				no_annot
10224	acttggttca	1				no_annot
10225	ccctatctgt	1	TC10798	84	0	similar to SP|Q9BZK7|TBLR_HUMAN Nuclear receptor co-repressor/HDAC3 complex subunit TBLR1 (TBL1-related protein 1). [Human] {Homo sapiens}, partial (68%)
10226	tgggcaaaaa	1				no_annot
10227	atgttattaa	1	CD163680	60	1	similar to GP|13661969|gb|AAK38131.1|AC058781_8|AC058781 L344.8 {Leishmania major}, partial (5%)
10228	cttcatctgg	1	C300153.1	64	1	
10228	cttcatctgg	1	C717574.1	62	1	
10229	taaggaacag	1				no_annot
10230	gtgttggtct	1				no_annot
10231	ttgtaaagag	1				no_annot
10232	tgtgaaatta	1				no_annot
10233	ggagttcgtg	1				no_annot
10234	atctaccaaa	1	TC8328	70	0	
10234	atctaccaaa	1	TC8329	25	0	weakly similar to GP|9652123|gb|AAF91409.1|AF271213_1|AF271213 disrupter of silencing SAS10 {Mus musculus}, partial (12%)
10235	tcgtcaatcc	1				no_annot
10236	gaattgatca	1				no_annot
10237	tcgtcctata	1	C602184.1	23	2	
10238	ttctacattg	1	CD202456	46	1	
10239	ttaaatctgt	1				no_annot
10240	ctaactttct	1				no_annot
10241	attactgtta	1				no_annot
10242	tattacaaaa	1	TC17970	25	0	similar to GP|17945032|gb|AAL48578.1||AY070956 RE05591p {Drosophila melanogaster}, partial (90%)

10242	tattacaaaa	1	AI447054	45	0	similar to GP|551606|gb|AAA67650.1||L34587 RNA polymerase II elongation factor SIII, p15 subunit {Homo sapiens}, partial (76%)
10243	cctcatttgg	1				no_annot
10244	atgtgagttg	1	TC13957	21	2	weakly similar to GP|19528133|gb|AAL90181.1||AY089443 AT26187p {Drosophila melanogaster}, partial (10%)
10245	tatttacgtt	1	TC9045	94	0	
10246	ccagttgatc	1	TC8948	10	5	similar to GP|12002020|gb|AAG43153.1|AF063594_1|AF063594 brain my036 protein {Homo sapiens}, partial (24%)
10247	gcgagtcgac	1				no_annot
10248	ccccaataca	1	TC17797	92	0	similar to SP|P53611|PGTB_HUMAN Geranylgeranyl transferase type II beta subunit(RAB geranylgeranyltransferase beta subunit) (RAB geranyl-geranyltransferase beta subunit) (RAB GG transferase beta) (RAB GGTase beta). [Human] {Homo sapiens}, partial (48%)
10249	atacaccaag	1				no_annot
10250	ttatacagta	1				no_annot
10251	tgaaccagat	1	C202324.1	5	3	
10251	tgaaccagat	1	C606510.1	5	3	
10252	gggggacact	1				no_annot
10253	atcctatgat	1				no_annot
10254	aactttattg	1	TC18303	77	0	weakly similar to GP|7303382|gb|AAF58440.1||AE003820 CG17019-PA {Drosophila melanogaster}, partial (7%)
10255	tttcgttcta	1				no_annot
10256	cattataatt	1				no_annot
10257	ttttatcgcg	1				no_annot
10258	tcgacttggt	1				no_annot
10259	tttagtgatg	1				no_annot
10260	gatgaagttt	1				no_annot
10261	gaacattcga	1				no_annot
10262	gaaaccggcg	1				no_annot

10263	tcttattgct	1	TC17717	11	0	weakly similar to GP|3985930|dbj|BAA34863.1||AB020623 DAM1 {Homo sapiens}, partial (77%)
10264	ttacacattc	1				no_annot
10265	gatagctttt	1				no_annot
10266	cgcgtcactt	1				no_annot
10267	agatatctgt	1				no_annot
10268	aaccctggcc	1				no_annot
10269	tttgtccatc	1				no_annot
10270	tattgcttta	1				no_annot
10271	gttaccatta	1				no_annot
10272	cgcaagttct	1				no_annot
10273	aagtctgtac	1				no_annot
10274	ataagcataa	1	TC8366	97	1	similar to GP|157310|gb|AAA28494.1||M37083 E74B protein {Drosophila melanogaster}, partial (4%)
10274	ataagcataa	1	CD075778	25	2	
10275	tcaagggggg	1				no_annot
10276	tgagtagaag	1				no_annot
10277	gatagcttta	1	CD196177	31	0	
10278	tcaatttaaa	1				no_annot
10279	ggatggaagg	1				no_annot
10280	gctgactaca	1				no_annot
10281	tagacgaggt	1				no_annot
10282	aatgttgtca	1				no_annot
10283	acgattctat	1	TC16846	90	0	GP|6980092|gb|AAF34722.1|AF232025_1|AF232025 TGF-beta signal transducer Smad2 {Schistosoma mansoni}, complete
10284	atggaacatt	1				no_annot
10285	tgtgtgtctg	1				no_annot
10286	aacgagaagc	1				no_annot
10287	ctgtaaagaa	1				no_annot
10288	tttgatgttc	1				no_annot

10289	ctccaacaaa	1	TC19074	77	1	
10290	ttactaatgt	1				no_annot
10291	tattacgggg	1				no_annot
10292	atggggtatg	1				no_annot
10293	gggaaggcaa	1	TC16835	91	0	weakly similar to SP|P09661|RU2A_HUMAN U2 small nuclear ribonucleoprotein A' (U2 snRNP-A'). [Human] {Homo sapiens}, partial (49%)
10294	gtacaattac	1	C611056.1	28	1	
10295	aagtggacaa	1	TC14057	47	0	weakly similar to GP|1399462|gb|AAB03268.1||U48736 serine/threonine-protein kinase PRP4h {Homo sapiens}, partial (29%)
10295	aagtggacaa	1	TC14058	90	0	weakly similar to GP|5052542|gb|AAD38601.1|AF145626_1|AF145626 BcDNA.GH04978 {Drosophila melanogaster}, partial (9%)
10295	aagtggacaa	1	TC14059	71	0	weakly similar to GP|1399462|gb|AAB03268.1||U48736 serine/threonine-protein kinase PRP4h {Homo sapiens}, partial (24%)
10296	attatgcagt	1	C603552.1	61	0	
10297	agatttcaag	1				no_annot
10298	caaatagtaa	1				no_annot
10299	atggaacatc	1				no_annot
10300	gtctacggca	1	TC7459	53	2	similar to GP|21068659|emb|CAD21558.1||AJ428513 HEL protein {Chironomus tentans}, partial (96%)
10300	gtctacggca	1	TC7461	68	0	similar to GP|2739119|gb|AAB94615.1||AF029061 BAT1 {Homo sapiens}, partial (69%)
10301	cttgacgtct	1				no_annot
10302	tatatgagtt	1	TC18042	6	1	
10303	agatcgccaa	1				no_annot
10304	cagaagggca	1				no_annot
10305	gttatagtag	1				no_annot
10306	caaatgaacc	1				no_annot
10307	tggtcatata	1	TC11826	14	0	similar to GP|17944341|gb|AAL48063.1||AY070592 RE69393p {Drosophila melanogaster}, partial (18%)
10308	tggacttcag	1				no_annot

10309	taacccgtat	1				no_annot
10310	tttaggtagg	1				no_annot
10311	aaagaataga	1	TC8419	70	2	
10312	tgttgttgtg	1				no_annot
10313	ttgggtagta	1				no_annot
10314	cattgctttt	1				no_annot
10315	gaatggcgac	1				no_annot
10316	aatgaggaag	1	TC17281	46	4	
10317	tgttgttgtc	1	CD201358	76	0	
10318	taacccgtag	1	C610184.1	59	1	
10319	aaacgtcgtc	1	TC7669	73	2	similar to PIR|T40486|T40486 phosphoinositide-dependent protein kinase 1SPBC4C3.11 [imported] - fission yeast (Schizosaccharomyces pombe), partial (5%)
10320	cgattcatcg	1				no_annot
10321	aacaataaaa	1	TC16671	89	0	
10322	atggttggga	1	CD195883	64	0	
10322	atggttggga	1	CD135048	52	0	similar to GP|17944580|gb|AAL48177.1||AY070706 RH56103p {Drosophila melanogaster}, partial (61%)
10323	tagaggatac	1				no_annot
10324	agtttccgaa	1	TC10068	44	0	similar to GP|22087553|gb|AAM90960.1|AF516365_1|AF516365 5-formyltetrahydrofolate cycloligase {Arabidopsis thaliana}, partial (9%)
10325	tagaggataa	1				no_annot
10326	gctgttcctt	1				no_annot
10327	tgtatgcaac	1	TC13635	79	0	weakly similar to GP|1749794|emb|CAA66229.1||X97630 serine/threonine protein kinase {Homo sapiens}, partial (5%)
10327	tgtatgcaac	1	TC13636	35	8	similar to GP|24496477|gb|AAN60072.1||AY151083 MAP/microtubule affinity-regulating kinase 4L {Mus musculus}, partial (28%)
10328	tccaaatgta	1				no_annot
10329	ggatatatat	1				no_annot
10330	tataacatta	1				no_annot

10331	cagaccaacc	1				no_annot
10332	taaatacttg	1				no_annot
10333	caaaagatga	1				no_annot
10334	ggggtgtatg	1				no_annot
10335	ttcgtattct	1	TC14367	80	2	weakly similar to GP|22946910|gb|AAN11076.1||AE003666 CG31683-PA {Drosophila melanogaster}, partial (13%)
10336	acaaactgtt	1	TC15342	64	1	
10337	gggtgtttgt	1				no_annot
10338	aaggtaattc	1				no_annot
10339	aagctgtgaa	1				no_annot
10340	aaggtaatta	1	AI977621	26	1	
10341	aattgggttt	1	TC8436	27	3	
10342	aacagagaaa	1				no_annot
10343	gcactaagcg	1				no_annot
10344	tccagagcta	1				no_annot
10345	ggtgaatacc	1	C606793.1	11	5	
10346	gattgtaaag	1				no_annot
10347	tgtgtatatg	1				no_annot
10348	acatcgcgca	1	TC16478	37	1	
10349	tgtgtatatc	1	TC11925	79	0	similar to GP|16266771|dbj|BAB69974.1||AB060291 kinase Akt/PKB {Asterina pectinifera}, partial (32%)
10350	agttatttca	1				no_annot
10351	gattgtaaaa	1				no_annot
10352	acggaaaagc	1				no_annot
10353	taatgaacga	1				no_annot
10354	cagcagcaaa	1				no_annot
10355	taaattgatg	1				no_annot
10356	agatcacaaa	1	CD196021	90	0	
10357	taaacggcta	1				no_annot
10358	ggagaacacc	1				no_annot
10359	gtaacgataa	1	C201508.1	34	2	

10359	gtaacgataa	1	C603862.1	33	2	
10360	ttccctcctc	1				no_annot
10361	tgatacgatg	1				no_annot
10362	gactattcac	1				no_annot
10363	tgaagagaat	1	CD121844	76	0	
10364	acaagcgcta	1				no_annot
10365	tccaaaaggg	1				no_annot
10366	taagcttagt	1				no_annot
10367	atattcatct	1	gi|161056|gb|M35499.1|SCMPMYA1	87	0	Schistosoma mansoni paramyosin mRNA, complete cds
10368	cgaagagact	1				no_annot
10369	cacggatatt	1	C605094.1	10	3	
10370	acaatatggg	1				no_annot
10371	tatagacggt	1				no_annot
10372	tagatgccac	1				no_annot
10373	aacgttatcg	1	TC13604	34	2	similar to SP|P00829|ATPB_BOVIN ATP synthase beta chain, mitochondrial precursor. [Bovine] {Bos taurus}, partial (91%)
10373	aacgttatcg	1	TC13605	66	0	similar to SP|Q25117|ATPB_HEMPU ATP synthase beta chain, mitochondrial precursor. [Sea urchin] {Hemicentrotus pulcherrimus}, partial (15%)
10374	agttatttat	1	TC10548	55	6	homologue to GP|2623606|gb|AAC48354.1||AF027877 mucin-like protein {Trypanosoma cruzi}, partial (10%)
10375	cactactgtt	1				no_annot
10376	ttcatttggt	1	TC15327	0	1	
10376	ttcatttggt	1	CD158137	42	1	
10377	caatatgact	1	TC18117	71	1	
10378	gaaaatgaat	1	CD129950	26	1	
10379	attcataaat	1	CD183662	35	0	
10379	attcataaat	1	CD141532	82	0	
10380	tagagcgtat	1				no_annot
10381	atagtagaaa	1				no_annot
10382	tttgacctta	1				no_annot

10383	agttatttaa	1				no_annot
10384	ctcagattgt	1	C207724.1	46	0	
10385	ctcttctatt	1				no_annot
10386	ttcatttggc	1				no_annot
10387	attacaacgt	1				no_annot
10388	tctttatttc	1				no_annot
10389	tgcattgaaa	1				no_annot
10390	aatgtatccc	1				no_annot
10391	acctataaag	1				no_annot
10392	gtgagctgta	1				no_annot
10393	gtagtaaata	1	TC7145	58	0	
10394	gtctttcctg	1	TC16874	71	1	weakly similar to GP|4050087|gb|AAC97961.1||AF109907 S164 {Homo sapiens}, partial (26%)
10395	catataatga	1	TC14645	64	0	homologue to GP|1916290|gb|AAC53117.1||U89876 ALY {Mus musculus}, partial (9%)
10396	gccgggacgc	1				no_annot
10397	tattcagcct	1				no_annot
10398	tgacatcagg	1				no_annot
10399	aatgttctta	1				no_annot
10400	ggtgaacgtg	1				no_annot
10401	ttgtagcaat	1	TC11028	9	1	weakly similar to GP|2224741|dbj|BAA57829.1||D50088 G-3-P dehydrogenase {Drosophila novamexicana}, partial (21%)
10401	ttgtagcaat	1	CD158150	63	1	
10401	ttgtagcaat	1	CD121738	7	0	weakly similar to GP|9758088|dbj|BAB08532.1||AB009052 dihydroxyacetone 3-phosphate reductase {Arabidopsis thaliana}, partial (12%)
10402	gggattaaaa	1				no_annot
10403	aagccaaacg	1				no_annot
10404	gtaataagag	1	TC14260	96	0	similar to GP|23180020|gb|AAF56673.2||AE003759 CG6051-PA {Drosophila melanogaster}, partial (3%)
10405	acctcctggg	1				no_annot

10406	aagccaaaca	1	CD063255	34	1	homologue to GP|5305329|gb|AAD41591.1||AF071011 myosin light chain {Schistosoma mansoni}, partial (21%)
10407	tattactact	1	TC12975	39	1	
10408	ttactcaagg	1	CD145418	3	1	
10409	tggtggtatg	1				no_annot
10410	tcaaaacacc	1	Z29959|Sma.25	62	0	Zinc finger protein (putative)
10411	cctaaaggat	1				no_annot
10412	tctgtattgc	1	TC11236	40	0	
10413	tttaggtaac	1				no_annot
10414	atctcttact	1	TC17953	29	3	
10415	gcaacggctt	1				no_annot
10416	aatacttagt	1				no_annot
10417	tggagttact	1				no_annot
10418	aattcgggtt	1				no_annot
10419	ttattctccg	1				no_annot
10420	tatatctatc	1				no_annot
10421	gtacataagg	1				no_annot
10422	atcctttcct	1				no_annot
10423	ttctaagatg	1	TC10824	73	0	
10424	acacgttttt	1				no_annot
10425	aaatacgcat	1				no_annot
10426	caggtatctg	1				no_annot
10427	taaccaacgt	1	C602308.1	80	0	
10428	gcttcctgtt	1				no_annot
10429	tggagttaca	1	C206711.1	61	1	
10430	ggggagttta	1				no_annot
10431	tatcagttgc	1	AI740380	68	1	similar to GP|20904371|gb| conserved protein {Methanosarcina mazei Goe1}, partial (1%)
10432	atgtattttg	1				no_annot
10433	tgtatttcgt	1	CD164082	79	0	
10434	ctcaatgcca	1				no_annot

10435	aagattagtt	1				no_annot
10436	gcacactgtc	1				no_annot
10437	ataaataaaa	1	TC8601	84	2	weakly similar to GP|27769360|gb|AAH42713.1||BC042713 DnaJ (Hsp40) homolog, subfamily B, member 9 {Mus musculus}, partial (32%)
10438	tatttcccca	1				no_annot
10439	cattgtattt	1				no_annot
10440	tgttgaccca	1	TC11208	22	1	
10441	cgcacagacg	1				no_annot
10442	cctattagca	1				no_annot
10443	tgttaaattt	1	TC13853	56	4	weakly similar to GP|27353219|dbj|BAC50219.1||AP005953 transcriptional regulatory protein {Bradyrhizobium japonicum USDA 110}, partial (6%)
10444	gataattata	1	C210589.1	18	2	
10444	gataattata	1	C607563.1	18	2	
10444	gataattata	1	C701404.1	68	0	
10445	ggtggtaatg	1	C202974.1	91	0	
10445	ggtggtaatg	1	C609224.1	89	0	
10446	tcagttgagt	1				no_annot
10447	gcaatcgaac	1				no_annot
10448	gctgataaat	1	CD135895	48	0	
10449	ggactcctca	1				no_annot
10450	ttcatttgct	1	TC15338	96	0	
10451	taatgaacat	1	TC11171	92	0	weakly similar to GP|7291278|gb|AAF46709.1||AE003453 CG9752-PA {Drosophila melanogaster}, partial (37%)
10452	aaaactcaga	1	M81085|Sma.1150	52	1	Stimulatory G protein alpha subunit
10453	agtaaagtaa	1				no_annot
10454	tctctcgttt	1				no_annot
10455	cattactact	1	TC19221	67	1	
10456	ggtaaaattt	1	C201718.1	13	5	
10456	ggtaaaattt	1	C603376.1	36	8	

10456	ggtaaaattt	1	C604621.1	12	5	
10457	tctacaagct	1				no_annot
10458	gttccatctg	1				no_annot
10459	atagtacatc	1				no_annot
10460	tgggatttca	1				no_annot
10461	cagttgatca	1	TC14134	18	1	
10462	taatatcatc	1				no_annot
10463	gctgataaac	1				no_annot
10464	gataactaag	1				no_annot
10465	gcactatggt	1				no_annot
10466	gataactaaa	1				no_annot
10467	caaacctttt	1	C204142.1	85	1	
10468	acagtatatg	1	C301031.1	28	0	
10469	aacttagaac	1				no_annot
10470	accaatatta	1				no_annot
10471	acaaatagtt	1				no_annot
10472	tgatccaatg	1	TC19546	35	0	
10473	tttgtactct	1				no_annot
10474	caaacctttc	1				no_annot
10475	aatcgaccaa	1	TC14859	73	1	similar to GP|4219081|gb|AAD12303.1||AF110638 cytochrome b {Himantura pacifica}, partial (6%)
10476	atcaatgtga	1				no_annot
10477	gaacgctgaa	1				no_annot
10478	cgattggcat	1	TC7118	86	2	weakly similar to GP|28274854|gb|AAO25692.1||AY195856 ankyrin repeat protein E4_8 {synthetic construct}, partial (28%)
10479	ctgatcacaa	1				no_annot
10480	atgggcgacc	1				no_annot
10481	caatgtgtgt	1				no_annot
10482	ttttacgtcg	1				no_annot
10483	cgtgaatttt	1	C300772.1	22	3	
10483	cgtgaatttt	1	C602264.1	10	6	

10484	tgcaaatcgg	1				no_annot
10485	tgcatccaaa	1	C201017.1	92	0	
10485	tgcatccaaa	1	C600144.1	86	0	
10486	aaaggattgg	1	C605201.1	12	4	
10487	gacattacac	1	TC15568	40	0	
10488	atcaaaacaa	1	TC12951	2	4	
10489	gcttccagga	1				no_annot
10490	gttgtggatg	1	TC13959	3	1	
10491	ggataacagt	1				no_annot
10492	gtttagcggt	1				no_annot
10493	cgggcataaa	1				no_annot
10494	ttactgaggt	1				no_annot
10495	tgtcattaat	1				no_annot
10496	aattttgtct	1	TC17284	31	0	similar to SP|Q24439|ATPO_DROME ATP synthase oligomycin sensitivity conferral protein, mitochondrial precursor(OSCP). [Fruit fly] {Drosophila melanogaster}, partial (27%)
10497	tttatcgtta	1	CD119350	37	0	
10498	agctgattaa	1	TC14115	64	1	similar to GP|18677192|gb|AAL78228.1|AF345800_1|AF345800 SCN esophageal secretory protein {Heterodera glycines}, partial (9%)
10499	ttttatgttt	1				no_annot
10500	tatcaatctt	1				no_annot
10501	tcgaagtttg	1				no_annot
10502	ataccttaaa	1	C207211.1	42	2	
10502	ataccttaaa	1	C608203.1	40	2	
10503	cagggggttg	1				no_annot
10504	gttcagctgt	1				no_annot
10505	caaaatgttc	1				no_annot
10506	tgtgtaaaaa	1				no_annot
10507	acctatggct	1				no_annot
10508	ataagtatgt	1				no_annot

10509	acaacaacca	1	TC19038	21	6	
10510	tgtcattaaa	1	CD095617	16	0	
10511	cgatggattt	1				no_annot
10512	gaactaaatc	1				no_annot
10513	ctgcaattaa	1				no_annot
10514	ttgtctgaaa	1				no_annot
10515	tctttctatc	1				no_annot
10516	agtgcacgac	1	C601125.1	49	0	
10517	cattttcctg	1				no_annot
10518	gtgctagatg	1	TC9245	23	0	similar to GP|17862956|gb|AAL39955.1||AY069810 SD05004p {Drosophila melanogaster}, partial (84%)
10519	tataaagatg	1				no_annot
10520	ggtatgtctg	1				no_annot
10521	tgattttttc	1	CD166967	35	0	
10522	ctcacgcact	1				no_annot
10523	gatgtcttct	1	TC14003	89	0	weakly similar to GP|9795244|dbj|BAB11685.1||D82983 prolidase {Mus musculus}, partial (67%)
10524	gataccagat	1	TC7989	47	1	weakly similar to GP|16768252|gb|AAL28345.1||AY060797 GH26345p {Drosophila melanogaster}, partial (23%)
10525	tatatgtgta	1	CD122239	50	1	similar to GP|15291169|gb|AAK92853.1||AY051429 GH10492p {Drosophila melanogaster}, partial (17%)
10526	ttagccctgt	1				no_annot
10527	ggtggattcg	1				no_annot
10528	tacagttcca	1				no_annot
10529	tgaaattacc	1	TC13998	57	2	
10530	tggtgagtag	1				no_annot
10531	tttgtactag	1				no_annot
10532	ttatcgttcc	1				no_annot
10533	ctttcctttc	1	TC13666	7	2	
10534	tcatatgttt	1				no_annot
10535	tgcacacata	1				no_annot

10536	ccgtcgagag	1				no_annot
10537	tggcttgtcc	1				no_annot
10538	gataccagaa	1				no_annot
10539	attagatata	1				no_annot
10540	agtacctatc	1				no_annot
10541	cactacagat	1				no_annot
10542	taagcccgaa	1				no_annot
10543	gacactgtgc	1				no_annot
10544	agtaacgaca	1	TC15043	52	0	
10544	agtaacgaca	1	TC18173	93	1	
10545	cactcaacct	1				no_annot
10546	ggcgcactgg	1				no_annot
10547	taaaccatct	1	AI976217	53	1	weakly similar to GP|20151533|gb|AAM11126.1||AY094773 GM14501p {Drosophila melanogaster}, partial (23%)
10548	ataacctgca	1				no_annot
10549	tcactggtaa	1				no_annot
10550	tcgtgccttc	1				no_annot
10551	attatttcgg	1				no_annot
10552	gtacataaag	1				no_annot
10553	ctacttaatt	1				no_annot
10554	tttttgtaat	1	CD156136	61	1	
10555	cggtatataa	1				no_annot
10556	gtgagcagaa	1				no_annot
10557	atacaggttg	1				no_annot
10558	acccctatgt	1				no_annot
10559	tgttgttggt	1				no_annot
10560	taaaccatcc	1				no_annot
10561	caaatgtaac	1	TC16995	40	1	
10562	atggatttcc	1				no_annot
10563	ttcactacac	1				no_annot

10564	aaaatgtctc	1	TC16788	34	4	similar to GP|6517204|dbj|BAA87880.1||AB035354 Drab11 {Drosophila melanogaster}, partial (77%)
10564	aaaatgtctc	1	TC16790	63	0	similar to GP|6517204|dbj|BAA87880.1||AB035354 Drab11 {Drosophila melanogaster}, partial (70%)
10565	tgtttatatt	1	C608903.1	28	0	
10566	atcttcacta	1				no_annot
10567	tatatgaggc	1	C210056.1	22	3	
10568	atatggattt	1	TC9227	67	1	
10568	atatggattt	1	TC15203	64	3	similar to GP|4038504|emb|CAA98273.1||Z73974 C. elegans GSP-1 protein (corresponding sequence F29F11.6) {Caenorhabditis elegans}, partial (83%)
10569	tattataacg	1	C606035.1	79	0	
10570	gtcaccgctt	1	TC18196	42	2	
10571	ccccgctaca	1				no_annot
10572	ggttgaatat	1				no_annot
10573	atgattattt	1	CD195171	21	1	
10574	atagacgacc	1				no_annot
10575	ttggtaaata	1	TC17862	8	2	weakly similar to GP|21627004|gb|AAF57625.2||AE003798 CG15100-PA {Drosophila melanogaster}, partial (5%)
10576	agaccgcaac	1				no_annot
10577	ttcagttatg	1				no_annot
10578	gttgctcgta	1				no_annot
10579	gtatgttgta	1				no_annot
10580	tgatcatttc	1				no_annot
10581	tgcactatcg	1				no_annot
10582	taacagctag	1				no_annot
10583	agcctacaca	1	TC18306	60	0	
10584	tgtatttcaa	1	C208297.1	21	6	
10585	gaatgtcaac	1				no_annot
10586	ttcgcaaagt	1				no_annot
10587	tacctctacg	1	TC8209	85	0	similar to SP|P50533|XCPE_XENLA Chromosome assembly protein XCAP-E. [African clawed frog] {Xenopus laevis}, partial (16%)

10588	atgattattc	1	TC9272	39	1	
10589	tgattacatt	1				no_annot
10590	ttaccaaata	1				no_annot
10591	gactgaccca	1				no_annot
10592	attactgtca	1				no_annot
10593	ttcgattttt	1	TC7997	75	0	similar to SP|O15269|LCB1_HUMAN Serine palmitoyltransferase 1(Long chain base biosynthesis protein 1) (LCB 1) (Serine-palmitoyl-CoA transferase 1) (SPT 1) (SPT1). [Human] {Homo sapiens}, partial (25%)
10594	cataacttta	1				no_annot
10595	tgaatagtta	1				no_annot
10596	acataaattg	1				no_annot
10597	ctcgcatata	1				no_annot
10598	ccactggtaa	1				no_annot
10599	cctacttctt	1				no_annot
10600	atatagtatt	1				no_annot
10601	tatatcaaac	1				no_annot
10602	attgaaatgc	1	CD075257	31	0	
10603	aagcatttga	1				no_annot
10604	gcgaatgact	1				no_annot
10605	aacactagaa	1				no_annot
10606	tgcgtatgtg	1				no_annot
10607	agggttacac	1				no_annot
10608	cggtcatagg	1				no_annot
10609	gtggcggaag	1	C200528.1	41	2	
10610	tgcgtatgtc	1	TC11378	78	0	
10611	gggaatttgt	1				no_annot
10612	gtaaacagca	1				no_annot
10613	cactgatatg	1	TC16313	49	1	
10614	tctaatgcaa	1	TC11214	13	1	similar to GP|19343976|gb|AAH25422.1||BC025422 cut-like 1, CCAAT displacement protein (Drosophila) {Homo sapiens}, partial (4%)

10615	gctatgtgtg	1				no_annot
10616	agccaactga	1	C611228.1	43	1	
10617	cagaatgaag	1				no_annot
10618	atagcaatag	1				no_annot
10619	gtaggttgga	1				no_annot
10620	gatgcatttg	1				no_annot
10621	atgtatatac	1	TC9854	38	1	
10622	caagtcactt	1				no_annot
10623	attttgtctc	1				no_annot
10624	tttgatgtct	1				no_annot
10625	tacaacgctt	1				no_annot
10626	catatattga	1	CD187234	7	1	homologue to GP|520556|gb|AAA20470.1||U12281 mariner transposase {Tetranychus urticae}, partial (8%)
10627	gaacaaattg	1	TC8198	21	10	similar to GP|26000552|gb|AAN75454.1||AY151155 Na-dependent Cl/HCO3 exchanger {Loligo pealei}, partial (24%)
10628	gtgttgctgt	1	TC10756	12	4	weakly similar to SP|Q14202|Z261_HUMAN Zinc finger protein 261 (DXS6673E protein). [Human] {Homo sapiens}, partial (4%)
10629	ataaattact	1				no_annot
10630	gagtatcttt	1				no_annot
10631	tccaagcacc	1				no_annot
10632	aattagcatc	1				no_annot
10633	aaaatatatg	1				no_annot
10634	aatttatcca	1	TC16167	48	0	
10635	aagcgtgatg	1				no_annot
10636	gattcaatta	1				no_annot
10637	ctaggaaact	1				no_annot
10638	ttactattat	1				no_annot
10639	ccttgacgaa	1	TC14137	38	2	weakly similar to SP|P17152|PM1_HUMAN Protein PM1. [Human] {Homo sapiens}, partial (23%)
10640	atgtttggca	1	TC11520	60	1	weakly similar to GP|13905162|gb|AAH06873.1||BC006873 LOC214597 protein {Mus musculus}, partial (26%)

10641	ggataacaaa	1	C308028.1	7	1	
10642	gttactattt	1	TC9229	11	1	
10643	cgtagtgctt	1	TC14986	55	0	
10644	tgaagtttgt	1	TC15553	26	2	weakly similar to GP|15559642|gb|AAH14178.1|AAH14178|BC014178 carbamoyl-phosphate synthetase 2, aspartate transcarbamylase, and dihydroorotase {Homo sapiens}, partial (17%)
10645	gggaggggga	1				no_annot
10646	ctcgattttt	1				no_annot
10647	tgatccggtt	1				no_annot
10648	tctagattaa	1				no_annot
10649	ttgcctagtg	1				no_annot
10650	tccgtgctgg	1				no_annot
10651	ggaaccggtt	1				no_annot
10652	gacttaggtt	1				no_annot
10653	acacaccgca	1				no_annot
10654	ctttccatct	1	C200492.1	6	2	
10654	ctttccatct	1	C603904.1	33	2	
10654	ctttccatct	1	C701577.1	30	1	
10655	gagaacacta	1				no_annot
10656	tttatgtttc	1	TC8330	18	6	weakly similar to GP|6594249|emb|CAB63561.1||AL031746 glutamic acid-rich protein (garp) {Plasmodium falciparum 3D7}, partial (6%)
10657	cgaaaccgat	1	CD091455	69	0	
10658	tagaatcata	1	TC11265	86	0	
10659	atgctcctgg	1				no_annot
10660	tcatctattt	1	TC14069	22	1	similar to GP|13365610|dbj|BAB39155.1||AB048713 SCARECROW {Pisum sativum}, partial (4%)
10661	gcgaatgaaa	1	TC11997	26	3	
10662	tcccggtgag	1				no_annot
10663	cattgcttcg	1				no_annot
10664	ggggatgttg	1				no_annot

10665	tacgaaccaa	1				no_annot
10666	tcaatgtttt	1	C201945.1	51	0	
10666	tcaatgtttt	1	C211350.1	14	2	
10666	tcaatgtttt	1	C211350.1	14	0	
10666	tcaatgtttt	1	C611870.1	30	2	
10666	tcaatgtttt	1	C611870.1	30	0	
10667	caaaagttag	1				no_annot
10668	aaggtaatct	1				no_annot
10669	tgcagcacca	1				no_annot
10670	aggttttctg	1				no_annot
10671	cctagattat	1	CD200832	3	2	
10672	taaagcactc	1				no_annot
10673	acaaggacaa	1				no_annot
10674	tcattactgc	1				no_annot
10675	atttgttgga	1				no_annot
10676	gggttagtag	1				no_annot
10677	tcgacagatg	1				no_annot
10678	cagcgttgtt	1				no_annot
10679	gagggaagaa	1				no_annot
10680	attatttcac	1	CD145408	1	1	similar to SP|Q91094|PRLR_ Prolactin receptor precursor (PRL-R) (TPRLR). [Common turkey] {Meleagris gallopavo}, partial (1%)
10681	attctgaata	1	TC9252	26	1	
10682	ttggcggtta	1				no_annot
10683	aaattaccaa	1				no_annot
10684	catacaaatg	1				no_annot
10685	catacaaata	1	TC16262	6	2	
10685	catacaaata	1	TC19347	91	0	
10686	tgatggtttt	1				no_annot
10687	tgttgttgag	1	TC16615	45	5	
10688	acaagaaaca	1				no_annot

10689	tatatgagaa	1				no_annot
10690	ttgatatgga	1	CD120497	45	1	
10691	taactttcca	1				no_annot
10692	gttcctgtcc	1	M86396|Sma.850	11	30	Epidermal growth factor receptor (SER, class 1 product)
10693	aaatatgcgc	1				no_annot
10694	ctacacataa	1				no_annot
10695	aactcaatat	1				no_annot
10696	aaaagttgcg	1	TC8399	95	0	
10697	gccaaaaatg	1	TC17364	67	2	similar to GP|17946096|gb|AAL49090.1||AY071468 RE54691p {Drosophila melanogaster}, partial (25%)
10698	aacctggaac	1	CD080937	78	0	weakly similar to GP|22945471|gb|AAN10459.1||AE003585 CG31938-PA {Drosophila melanogaster}, partial (15%)
10699	tattattagt	1	C201397.1	9	6	
10699	tattattagt	1	C603839.1	9	5	
10700	tacccaaagt	1				no_annot
10701	tccaatacaa	1				no_annot
10702	gctccagaga	1	TC9320	60	0	
10703	caggcgaaga	1				no_annot
10704	gagtgcacct	1	TC10827	2	6	similar to SP|P38062|AMP2_RAT Methionine aminopeptidase 2(MetAP 2) (Peptidase M 2) (Initiation factor 2 associated 67 kDa glycoprotein) (p67) (p67eIF2). [Rat] {Rattus norvegicus}, partial (76%)
10705	attgcggtat	1				no_annot
10706	ctagggtcgt	1				no_annot
10707	aggttatggt	1				no_annot
10708	tacttttcaa	1				no_annot
10709	gacaattgtg	1	TC19483	51	0	
10709	gacaattgtg	1	CD067669	58	2	
10710	tgcactttgt	1	TC17487	29	2	
10710	tgcactttgt	1	TC17488	87	0	similar to SP|P52485|UBC2_DROME Ubiquitin-conjugating enzyme E2-24 kDa(Ubiquitin-protein ligase) (Ubiquitin carrier protein). [Fruit fly] {Drosophila melanogaster}, partial (63%)

10711	ggaagtcgag	1				no_annot
10712	aagaaccctg	1	TC17938	21	0	similar to PIR|JC5970|JC5970 nuclear ubiquitin-conjugating enzyme - fruit fly (Drosophila melanogaster), partial (94%)
10712	aagaaccctg	1	TC19135	24	1	similar to GP|4732095|gb|AAD28601.1|AF128240_1|AF128240 ubiquitin-conjugating enzyme 9 {Danio rerio}, partial (95%)
10713	ttcctgttgg	1	CD121345	55	1	
10714	taaaatgtgt	1	TC16264	69	0	similar to GP|2114042|dbj|BAA20073.1||AB001742 Gi2 alpha subunit {Oryzias latipes}, partial (26%)
10715	ctgcagtgat	1				no_annot
10716	caaatactca	1	TC17170	42	3	similar to EGAD|66062|69017 ras-related protein RAL-A {Drosophila melanogaster}, partial (30%)
10717	gggttgaaat	1	C308572.1	26	1	
10718	taccattttc	1				no_annot
10719	ataacaccca	1				no_annot
10720	tgacgtctac	1				no_annot
10721	attttaaaga	1	TC13703	9	2	
10722	actatgcggg	1				no_annot
10723	agaactgaga	1				no_annot
10724	tggaagaagt	1				no_annot
10725	cagttgttac	1	TC10362	25	2	homologue to GP|2829289|gb|AAC00519.1||AF044412 HSP70 {Schistosoma japonicum}, complete
10725	cagttgttac	1	CD162166	49	0	homologue to GP|2829289|gb|AAC00519.1||AF044412 HSP70 {Schistosoma japonicum}, partial (21%)
10726	atcactatac	1				no_annot
10727	tataaggcgc	1				no_annot
10728	gcgttgttcc	1	TC13559	36	1	
10729	cagttgttaa	1				no_annot
10730	tgagaactgt	1	C210045.1	12	3	
10731	cataatattt	1				no_annot
10732	gggttgaaag	1				no_annot
10733	aaaatgacag	1				no_annot

10734	cattgtatga	1	C209235.1	30	0	
10735	gatgaacttt	1				no_annot
10736	tctactacaa	1				no_annot
10737	gatacttgtt	1				no_annot
10738	ggtaaaatgg	1				no_annot
10739	gttgcgattc	1				no_annot
10740	ataatgaatt	1				no_annot
10741	cttccggatg	1	TC7050	55	2	similar to GP|13171103|gb|AAK13589.1||AF139989 rRNA intron-encoded homing endonuclease {Oryza sativa}, partial (52%)
10742	atgtcatact	1				no_annot
10743	atagttttca	1				no_annot
10744	gttacagaga	1				no_annot
10745	ttactttatt	1				no_annot
10746	tctaaacgga	1	TC7669	22	6	similar to PIR|T40486|T40486 phosphoinositide-dependent protein kinase 1SPBC4C3.11 [imported] - fission yeast (Schizosaccharomyces pombe), partial (5%)
10747	ttcccagttc	1				no_annot
10748	taaacggcag	1	TC6865	57	1	
10749	gattccaatt	1				no_annot
10750	actattcata	1				no_annot
10751	agtattttac	1				no_annot
10752	actaccttac	1				no_annot
10753	gataatggcg	1				no_annot
10754	ataactagaa	1				no_annot
10755	ttccctccag	1				no_annot
10756	aaaagttgac	1	C207250.1	79	1	
10756	aaaagttgac	1	C612036.1	74	1	
10757	taatctcgtt	1				no_annot
10758	gacttaaagt	1				no_annot
10759	ttactttatg	1				no_annot

10760	tcgatgtaca	1	TC14100	93	0	similar to GP|2444183|gb|AAC01949.1||AF001536 Muscleblind isoform C {Drosophila melanogaster}, partial (23%)
10761	caaattgaca	1				no_annot
10762	attgccagtc	1				no_annot
10763	caaaccttgc	1				no_annot
10764	tgtgtacgtt	1				no_annot
10765	gctgtgaatt	1	TC7806	48	2	
10766	tgcctcaatg	1				no_annot
10767	taacactgag	1				no_annot
10768	agcgagaatg	1				no_annot
10769	atccattggg	1				no_annot
10770	atatccggta	1				no_annot
10771	aaaataaaca	1	C702811.1	43	2	
10772	cacctgtgat	1				no_annot
10773	tttataactt	1	TC8405	91	0	similar to GP|295139|gb|AAA71978.1||L13334 ORFB {Staphylococcus lugdunensis}, partial (7%)
10773	tttataactt	1	CD098003	40	2	
10774	aaatgcaaaa	1	TC8043	34	6	similar to GP|5305389|gb|AAD41627.1|AF072328_1|AF072328 dynein light chain 2 {Schistosoma japonicum}, complete
10775	attcgtttct	1				no_annot
10776	ttttccttga	1				no_annot
10777	tgtgtacgtg	1				no_annot
10778	taaccatcca	1				no_annot
10779	gatcatcaca	1	C610663.1	65	0	
10780	gaagcccatc	1				no_annot
10781	ttgaagctgc	1				no_annot
10782	acagtgtcca	1	TC10934	60	2	similar to GP|2970625|gb|AAC06013.1||AF051936 ryanodine receptor {Homarus americanus}, partial (7%)
10783	gatgatgatg	1	TC13054	93	0	weakly similar to SP|P87179|YB1E_SCHPO Protein C30B4.01c in chromosome II. [Fission yeast] {Schizosaccharomyces pombe}, partial (11%)
10784	ccgaatgttt	1				no_annot

10785	ggttgattca	1	TC7712	70	1	
10786	acattcgcga	1				no_annot
10787	tgaagtttaa	1				no_annot
10788	gatgatgata	1	TC16948	9	15	similar to PIR|PC4365|PC4365 calcium-binding protein - tapeworm (Echinococcus granulosus) (fragment), partial (43%)
10789	acaacatcac	1	H98376	24	0	
10790	gagattgtgt	1				no_annot
10791	ggtatagggt	1				no_annot
10792	tttgctatat	1				no_annot
10793	aaaaaaatga	1	C209684.1	71	1	
10794	ctcccagttc	1				no_annot
10795	catattggtt	1	C609315.1	96	0	
10796	gttaatgtgc	1				no_annot
10797	ctcttctaaa	1				no_annot
10798	caaattgaaa	1				no_annot
10799	gatcatcaat	1				no_annot
10800	gaatccgaga	1	TC14206	36	3	
10801	agaaaatgag	1				no_annot
10802	aaaattgctc	1				no_annot
10803	tagacttagg	1				no_annot
10804	caaccatcct	1				no_annot
10805	aatataagtt	1				no_annot
10806	aaatggagct	1				no_annot
10807	aaaataaaaa	1	TC7969	92	0	
10807	aaaataaaaa	1	TC11578	2	4	
10808	tattattacc	1				no_annot
10809	cttgctatcc	1				no_annot
10810	ttattttcct	1				no_annot
10811	ttcatagata	1				no_annot
10812	ctgtgttgtg	1				no_annot

10813	ctctcgacgt	1				no_annot
10814	aatcaaatca	1				no_annot
10815	aatcactata	1				no_annot
10816	ggaacgtgta	1				no_annot
10817	tttatgatat	1	TC7650	74	0	weakly similar to GP|19526271|gb|AAL89665.1|AF411956_6|AF411956 histone deacetylase {Takifugu rubripes}, partial (11%)
10817	tttatgatat	1	TC17163	35	2	homologue to GP|1736824|dbj|BAA15968.1||D90848 Ribokinase. {Escherichia coli}, partial (6%)
10818	attagataga	1				no_annot
10819	ttggacaatg	1				no_annot
10820	gggatgattg	1				no_annot
10821	aaatgattcg	1				no_annot
10822	tccagaccta	1				no_annot
10823	ggtcttttcc	1				no_annot
10824	ataagacagt	1				no_annot
10825	cattgtatcc	1				no_annot
10826	ggcgattcct	1				no_annot
10827	accactgcta	1				no_annot
10828	gaacgttgca	1				no_annot
10829	atatgtcgaa	1				no_annot
10830	aataccgttg	1				no_annot
10831	aagagcaagc	1				no_annot
10832	agtatacaaa	1	TC8294	71	1	weakly similar to GP|22758864|gb|AAN05591.1||AF526208 ribosomal protein L7 {Argopecten irradians}, partial (17%)
10833	tttcccactt	1				no_annot
10834	gcggcagtaa	1				no_annot
10835	atatatgatt	1				no_annot
10836	ctcccgaatt	1				no_annot
10837	tgcgtttctg	1				no_annot
10838	tgtataatta	1	TC19038	72	2	

10839	ttatactgcc	1	AI394983	52	0	
10840	gaataatgtt	1	CD073676	0	5	
10841	cctttctaga	1				no_annot
10842	agattagcga	1				no_annot
10843	gttggtctcc	1				no_annot
10844	atttttgctt	1	TC15709	66	3	similar to SP|P32391|ARP3_HUMAN Actin-like protein 3 (Actin-related protein 3) (Actin-2). [Bovine] {Bos taurus}, partial (36%)
10845	ctcagtgggg	1				no_annot
10846	cccctctttc	1	TC10746	92	0	similar to GP|13235592|emb|CAC33779.1||AJ301810 SclB protein {Streptococcus pyogenes}, partial (4%)
10847	aagaaataat	1	TC19627	50	0	
10847	aagaaataat	1	CD074158	7	0	
10848	agagcaagtg	1				no_annot
10849	tctgggtata	1				no_annot
10850	atcattcagg	1				no_annot
10851	ggagttgtta	1				no_annot
10852	atgaagaaga	1				no_annot
10853	gtagactatt	1	TC11198	5	3	
10854	gtgtatgcta	1				no_annot
10855	tttaggcgaa	1	TC10637	21	7	weakly similar to SP|O43315|AQP9_HUMAN Aquaporin 9 (Small solute channel 1). [Human] {Homo sapiens}, partial (21%)
10856	taaaaatcga	1				no_annot
10857	agcaacggaa	1				no_annot
10858	ggatgtgaag	1				no_annot
10859	aaatgattag	1				no_annot
10860	gcgagtgtaa	1				no_annot
10861	aaaacggtct	1				no_annot
10862	tagctaggtt	1				no_annot
10863	gttagggagg	1				no_annot
10864	tgaatagtgg	1				no_annot

10865	tagatggatg	1				no_annot
10866	tttctagcaa	1				no_annot
10867	tatcatcttt	1				no_annot
10868	tatacatatg	1				no_annot
10869	atgacatttg	1				no_annot
10870	cattttcccc	1				no_annot
10871	taacggtcct	1				no_annot
10872	cacaagacga	1				no_annot
10873	ggctatacaa	1				no_annot
10874	gctggtttat	1	CD183470	79	0	
10875	caaatccaat	1	R93106	19	1	
10876	cctgcaaact	1				no_annot
10877	atatttttgt	1				no_annot
10878	gggtacgtca	1				no_annot
10879	acagacaaac	1				no_annot
10880	ggaaccaaaa	1	TC14107	40	0	
10880	ggaaccaaaa	1	TC14108	37	1	
10881	actactatat	1	CD163045	35	0	
10882	gatgcattgg	1				no_annot
10883	cgcactttat	1				no_annot
10884	gaataaagga	1				no_annot
10885	ttagattgga	1				no_annot
10886	gcttgtatat	1				no_annot
10887	gtagacaagt	1				no_annot
10888	aaattttgac	1				no_annot
10889	ccgtgtgtac	1				no_annot
10890	gtaaccctat	1				no_annot
10891	gtattaaagc	1				no_annot
10892	gattcaatgc	1	TC16413	45	2	similar to GP|18181863|emb|CAC85211.2||AJ312106 cathepsin B endopeptidase {Schistosoma mansoni}, partial (20%)

10892	gattcaatgc	1	CD091443	1	2	
10893	tagtttgtat	1				no_annot
10894	gattaagtaa	1	gi|3599492|gb|AF085145.1|AF085145	50	2	Schistosoma mansoni NADH dehydrogenase subunit 5 (NU5M) mRNA, complete cds, mitochondrial gene encoding mitochondrial protein
10895	aatcagtgta	1	C317366.1	72	0	
10896	gcttgtataa	1				no_annot
10897	ttgcgtatca	1				no_annot
10898	acaagaggag	1				no_annot
10899	taagcgtttg	1	C200486.1	0	3	
10900	gaagcactgg	1				no_annot
10901	tgatttttat	1	TC13805	72	0	
10902	tgatagtaaa	1	C210119.1	34	0	
10903	aacgaccact	1				no_annot
10904	tagtacacgt	1	CD134302	67	0	
10905	tatacaaagt	1	TC18209	89	0	
10906	gtggcaagac	1	TC11187	88	1	similar to GP|12001996|gb|AAG43141.1|AF061730_1|AF061730 My027 protein {Homo sapiens}, partial (57%)
10907	ttacatatgc	1				no_annot
10908	aaaaaaatag	1	C313535.1	15	0	
10908	aaaaaaatag	1	C706506.1	15	0	
10909	ctatgaaaat	1	TC7649	90	0	similar to PIR|I38176|I38176 ras-related GTPase rag, splice form A [imported] - human, partial (70%)
10910	gtccgtactg	1				no_annot
10911	gatgggataa	1	DQ249305|Sma.5246	45	2	CD36-like class B scavenger receptor
10912	gttgtcacgc	1				no_annot
10913	gttaatgtag	1				no_annot
10914	ttgtgaccca	1				no_annot
10915	cattatcgtc	1				no_annot
10916	atgaagaact	1				no_annot
10917	gcaagaagtg	1				no_annot

10918	tataaagaaa	1	TC11447	44	1	
10918	tataaagaaa	1	TC13738	26	3	weakly similar to SP|P26268|ODPT_ASCSU Pyruvate dehydrogenase E1 component alpha subunit type II, mitochondrial precursor(PDHE1-A) (Fragment). [Pig roundworm, Ascaris lumbricoides] {Ascaris suum}, partial (74%)
10919	aagacaagaa	1				no_annot
10920	ttatgaggga	1	TC8643	13	6	
10921	tttcaatatg	1				no_annot
10922	aaaatgtccc	1				no_annot
10923	gtaacatcaa	1				no_annot
10924	gaagttctcg	1				no_annot
10925	aggtgatata	1	C608325.1	19	2	
10926	ctgcaccggt	1				no_annot
10927	ttgtaaatta	1	TC17489	2	13	weakly similar to PIR|B38128|B38128 epithelin/granulin precursor - rat, partial (13%)
10928	accattggaa	1				no_annot
10929	tgccgacgga	1				no_annot
10930	taatgtctgg	1				no_annot
10931	taacaaccaa	1				no_annot
10932	ttgcattacc	1	C611528.1	2	4	
10933	ttacaacatt	1				no_annot
10934	atcattcaca	1	TC8798	27	4	
10935	ccccgtggag	1				no_annot
10936	tgtgactacg	1				no_annot
10937	tcctttagtc	1				no_annot
10938	gtggtttggt	1				no_annot
10939	cgcacacaca	1	TC14031	76	1	
10939	cgcacacaca	1	CD166727	34	0	
10940	agtaaactac	1				no_annot
10941	ttcgcataat	1	C302165.1	36	2	
10941	ttcgcataat	1	C706694.1	36	2	

10942	ttctttgggg	1	CD086088	17	2	
10943	taaaatcttg	1				no_annot
10944	atgattatca	1	TC8103	33	0	
10944	atgattatca	1	TC8787	5	2	similar to GP|2194196|gb|AAB61133.1||U61158 GdmH {Staphylococcus gallinarum}, partial (5%)
10945	gcaactatga	1				no_annot
10946	gctgcttgca	1				no_annot
10947	tcgtcttact	1				no_annot
10948	tttcaaaagt	1	C202219.1	78	1	
10948	tttcaaaagt	1	C608531.1	70	0	
10949	gtgagaccag	1				no_annot
10950	ctttatgtaa	1				no_annot
10951	aacaatcgac	1				no_annot
10952	acaataattg	1				no_annot
10953	gtcacaaaag	1				no_annot
10954	catttgaata	1				no_annot
10955	tcaacagttt	1	TC18866	84	1	
10956	gtctgtttaa	1				no_annot
10957	ttcctaacac	1				no_annot
10958	gacataattc	1				no_annot
10959	ctgtaaattg	1	CD078997	28	1	similar to GP|17944791|gb|AAL48462.1||AY070840 GH21062p {Drosophila melanogaster}, partial (3%)
10960	atgattatat	1	TC17979	93	0	similar to GP|1125833|gb|AAC69020.1||U43283 Rab family protein 6.2 {Caenorhabditis elegans}, partial (82%)
10961	cgatagcgtg	1				no_annot
10962	agacatccac	1	C201239.1	25	1	
10962	agacatccac	1	C600580.1	24	1	
10963	taattaagtc	1				no_annot
10964	cataacttat	1				no_annot
10965	acagagagac	1	CD139422	51	0	similar to PIR|T42235|T42235 FKHR protein homolog - Caenorhabditis elegans, partial (9%)

10965	acagagagac	1	CD166495	76	1	
10966	taattaagta	1				no_annot
10967	ttcagttaac	1				no_annot
10968	tttggctcgt	1				no_annot
10969	cgatgtgtta	1				no_annot
10970	accagcgaca	1	C202257.1	47	1	
10971	caatgtctga	1				no_annot
10972	ttacctgcta	1				no_annot
10973	gccgctctat	1				no_annot
10974	catattaacg	1	TC10541	26	2	homologue to GP|21686538|gb|AAM74948.1|AF519808_1|AF519808 MA {Schistosoma japonicum}, partial (11%)
10975	gtattaaacc	1	TC18242	81	0	weakly similar to SP|P13051|UNG_HUMAN Uracil-DNA glycosylase, mitochondrial precursor(UDG). [Human] {Homo sapiens}, partial (58%)
10975	gtattaaacc	1	CD166976	95	0	similar to SP|P43731|UNG_HAEIN Uracil-DNA glycosylase(UDG). {Haemophilus influenzae}, partial (27%)
10976	gacctgaagg	1	TC9596	20	2	
10977	tgaatgaact	1				no_annot
10978	gttcaaccaa	1	U57003|Sma.847	15	2	Breast basic conserved protein (BBC1)
10979	ttgcttccat	1				no_annot
10980	gaatcccatt	1	TC11007	71	1	similar to GP|25989490|gb|AAM18788.1||AY092764 pontin {Danio rerio}, partial (92%)
10981	gagattcttt	1				no_annot
10982	tataaacatt	1				no_annot
10983	tacgtaatgc	1	TC16490	68	0	similar to GP|7298510|gb|AAF53729.1||AE003661 CG10639-PA {Drosophila melanogaster}, partial (17%)
10984	gttaatcttc	1	TC14511	14	1	
10985	agtgcagtgg	1	TC7029	94	0	
10985	agtgcagtgg	1	TC7082	21	14	
10985	agtgcagtgg	1	TC7082	21	0	
10985	agtgcagtgg	1	TC7123	78	0	
10985	agtgcagtgg	1	CD112937	58	0	

10985	agtgcagtgg	1	CD193168	69	0	
10985	agtgcagtgg	1	CD122521	56	0	
10985	agtgcagtgg	1	CD135768	83	0	
10985	agtgcagtgg	1	CD151846	63	0	
10986	gttaatctta	1				no_annot
10987	attttgtcat	1	CD142246	17	1	weakly similar to GP|4519169|dbj|BAA75507.1||AB009875 rhodopsin kinase {Octopus dofleini}, partial (14%)
10988	tctttgtgaa	1	C601632.1	82	0	
10989	gcacaaccat	1				no_annot
10990	agctgttaac	1				no_annot
10991	tagaatcacc	1				no_annot
10992	atctcaactc	1	TC11750	86	0	weakly similar to PIR|A41206|A33183 microtubule-associated protein 4 - human, partial (4%)
10992	atctcaactc	1	CD128123	76	0	
10992	atctcaactc	1	CD171437	20	1	
10993	gctcctatac	1	TC10683	96	1	similar to GP|21711773|gb|AAM75077.1||AY128484 RE61939p {Drosophila melanogaster}, partial (91%)
10994	cactgataaa	1				no_annot
10995	acgatgacaa	1				no_annot
10996	acgtgtaata	1				no_annot
10997	tacaaaaaac	1	TC14503	83	0	
10997	tacaaaaaac	1	AI975351	18	1	homologue to SP|P34096|RNS4_HUMAN Ribonuclease 4 precursor(RNase 4). [Human] {Homo sapiens}, partial (8%)
10998	tacaaaaaaa	1				no_annot
10999	cacaaaaacg	1				no_annot
11000	cttgctttct	1				no_annot
11001	atgtgtgaga	1				no_annot
11002	tttcgcaatc	1	TC8944	63	1	
11003	atcaggcagt	1				no_annot
11004	tgtggagagg	1				no_annot

11005	catattaaag	1	TC14250	76	0	similar to GP|12642042|gb|AAK00125.1|AF200412_1|AF200412 cytochrome oxidase subunit I (COI) {Wiebesia brusi}, partial (4%)
11006	aaaatataag	1				no_annot
11007	aactggttcc	1				no_annot
11008	tcaatatctg	1	C300278.1	36	0	
11008	tcaatatctg	1	C713979.1	36	0	
11009	ctcagttaac	1				no_annot
11010	ggattgataa	1	TC12497	66	0	
11011	gagtgatgga	1				no_annot
11012	ccattgcgca	1				no_annot
11013	taacgtgcga	1	CD156131	57	2	
11014	taattggacg	1	TC12417	58	0	similar to GP|17862908|gb|AAL39931.1||AY069786 SD02886p {Drosophila melanogaster}, partial (9%)
11015	tgcgagctcc	1				no_annot
11016	ccacaaaagt	1				no_annot
11017	tgttacggga	1				no_annot
11018	ttatgaggat	1				no_annot
11019	atatcgagca	1				no_annot
11020	catagagtga	1				no_annot
11021	ttgtgttgga	1				no_annot
11022	gttgtcacac	1				no_annot
11023	acattgtcat	1				no_annot
11024	atgcttctgc	1	C202208.1	23	3	
11024	atgcttctgc	1	C603441.1	22	4	
11025	gtgggctagc	1	TC9577	72	1	
11026	cagtttcttg	1				no_annot
11027	ggtaaattct	1				no_annot
11028	ctcgatttag	1				no_annot
11029	gtgatatatg	1				no_annot
11030	tatacaaaaa	1				no_annot

11031	ttcttccgga	1				no_annot
11032	gtagttgcgt	1				no_annot
11033	tttatgttag	1				no_annot
11034	tctggcgacc	1	TC7510	91	0	similar to GP|2245671|gb|AAB62569.1||AF006516 e3B1 {Homo sapiens}, partial (16%)
11035	gagaacacaa	1				no_annot
11036	ccttaagaaa	1				no_annot
11037	agagaataga	1				no_annot
11038	aagttgaggt	1	CD083463	74	0	
11039	ggcatctcca	1				no_annot
11040	cagaatcacc	1				no_annot
11041	acacagtttg	1				no_annot
11042	acgattgctt	1				no_annot
11043	ttttggcatt	1	TC8110	73	0	
11044	aactaactat	1				no_annot
11045	ctacgagtcc	1				no_annot
11046	gcaactatat	1				no_annot
11047	atctcgataa	1				no_annot
11048	aggtattgtt	1				no_annot
11049	attgattatc	1	C606035.1	51	1	
11050	aaccactgtt	1				no_annot
11051	ggaaggttca	1				no_annot
11052	aagttattgc	1	TC7454	64	5	similar to GP|21392234|gb|AAM48471.1||AY118442 SD06874p {Drosophila melanogaster}, partial (78%)
11053	aaaagcttta	1	C200627.1	1	5	
11053	aaaagcttta	1	C606438.1	11	5	
11054	acgataaaag	1				no_annot
11055	gaaaaggtgc	1	C609877.1	9	2	
11055	gaaaaggtgc	1	C610529.1	37	0	
11056	aaagatccac	1	TC17377	11	1	
11057	tgccaagatg	1				no_annot

11058	cttcagacag	1	CD133033	18	0	weakly similar to GP|14575528|emb|CAC42882.1||AJ312398 leishmanolysin-like peptidase, variant 2 {Homo sapiens}, partial (4%)
11059	gtcaggaaat	1				no_annot
11060	tacgtaatca	1				no_annot
11061	aattgagcag	1				no_annot
11062	tacatttgta	1				no_annot
11063	gacgagtggg	1				no_annot
11064	taaactttgg	1	TC15844	85	0	similar to GP|18447544|gb|AAL68333.1||AY075526 RE72863p {Drosophila melanogaster}, partial (28%)
11065	aaaggttacc	1				no_annot
11066	acataattgt	1				no_annot
11067	tgttaattac	1	TC16928	84	3	
11067	tgttaattac	1	TC16929	57	4	
11068	ataaatcgat	1				no_annot
11069	gagactgata	1				no_annot
11070	gttttggaca	1	TC11944	42	0	
11071	ctgaccctcg	1	CD118996	65	0	homologue to GP|21107906|gb|AAM36578.1||AE011803 transport protein {Xanthomonas axonopodis pv. citri str. 306}, partial (11%)
11072	tggcaatgaa	1	CD169489	82	0	
11073	tttcaaaaaa	1				no_annot
11074	tataatataa	1	TC14425	93	0	weakly similar to GP|19911056|dbj|BAB87844.1||AB060168 hArpNbeta-s {Homo sapiens}, partial (41%)
11074	tataatataa	1	BE473327	55	0	
11075	agttgggtta	1				no_annot
11076	tatcctttct	1				no_annot
11077	aattggctga	1				no_annot
11078	tcctaaaaag	1				no_annot
11079	aaggtccgtt	1	CD201401	84	0	similar to GP|27884034|gb|AAO23957.1||AY183476 HZGJ {Homo sapiens}, partial (16%)
11080	tatccacaga	1				no_annot

11081	tcctacgcat	1				no_annot
11082	tagacgattg	1				no_annot
11083	ttactttacc	1				no_annot
11084	taggctgagt	1				no_annot
11085	atatatgagg	1				no_annot
11086	ggaaggttaa	1				no_annot
11087	gctgtgaact	1				no_annot
11088	caggaatgga	1				no_annot
11089	ttttgatgtg	1				no_annot
11090	atttgctttt	1				no_annot
11091	tttgatctcc	1				no_annot
11092	gcagccgtgg	1	TC7453	73	0	similar to GP|20976876|gb|AAM27513.1||AY102684 LD24134p {Drosophila melanogaster}, partial (6%)
11093	acttttactc	1	CD079835	59	0	
11094	ttttgcacat	1	AA140569	32	0	
11095	agcttcttgt	1				no_annot
11096	atcggaggtt	1				no_annot
11097	ttaacctctt	1				no_annot
11098	aagaacccac	1				no_annot
11099	atcctaatat	1	TC10727	75	0	similar to GP|12964610|dbj|BAB32671.1||AB047391 pre-mRNA processing 8 protein {Mus musculus}, partial (13%)
11100	gctgtgaacc	1				no_annot
11101	gcccgcgaat	1				no_annot
11102	gctgtgaaca	1				no_annot
11103	tgcggaaagg	1				no_annot
11104	tcccggattt	1				no_annot
11105	cgaatcgacc	1				no_annot
11106	ccgccatcaa	1				no_annot
11107	tacgtcaaga	1				no_annot
11108	actcgcccca	1				no_annot
11109	gtcgacgtct	1	AY456264|Sma.1225	19	8	Ftz-F1 interacting protein

11110	acaagtgcaa	1				no_annot
11111	tatgttgatc	1	TC17212	75	1	similar to GP|7297912|gb|AAF53158.1||AE003634 CG14946-PA {Drosophila melanogaster}, partial (8%)
11112	ctagtgagta	1				no_annot
11113	gattctaatg	1	TC10274	5	0	
11114	gttactttgt	1				no_annot
11115	aagtcaaatg	1	L47086	54	0	
11115	aagtcaaatg	1	AI395291	60	0	weakly similar to SP|P58249|PHS_DROVI Pterin-4-alpha-carbinolamine dehydratase(PHS) (4-alpha-hydroxy-tetrahydropterin dehydratase) (Pterin carbinolamine dehydratase) (PCD). [Fruit fly] {Drosophila virilis}, partial (49%)
11116	ccaaccgatt	1				no_annot
11117	gtatcggtaa	1	TC18054	30	2	
11118	ataatgaaag	1				no_annot
11119	gaaaaagcta	1				no_annot
11120	ttgggggctt	1	M31106|Sma.60	33	1	Clone G2.[1,6] glutathione S-transferase
11121	gaatcactgc	1	TC10377	84	0	homologue to GP|160925|gb|AAA29853.1||M94346 A.1.12/9 antigen {Schistosoma mansoni}, partial (26%)
11122	gtcattcgtt	1	CD187122	91	0	
11123	tacaccacat	1	C300380.1	74	0	
11123	tacaccacat	1	C718551.1	74	0	
11124	atgtgtgaaa	1				no_annot
11125	ctatgataag	1				no_annot
11126	caaagaaggc	1	TC10582	69	0	similar to SP|Q13123|RED_HUMAN Red protein (RER protein) (IK factor) (Cytokine IK). [Human] {Homo sapiens}, partial (20%)
11127	ttccagcata	1				no_annot
11128	ttttttcagg	1	TC7486	76	0	similar to GP|161038|gb|AAA29903.1||M15509 major egg antigen {Schistosoma mansoni}, partial (49%)
11129	cattgacaat	1	TC9454	16	1	
11130	aacaacgtca	1	TC8767	70	0	weakly similar to GP|27805181|emb|CAD58849.2||AJ534334 SMC3 protein {Takifugu rubripes}, partial (7%)
11131	cggtggcggg	1				no_annot

11132	acccgtacat	1				no_annot
11133	gtgcacgaaa	1				no_annot
11134	ttaaccacgt	1				no_annot
11135	tagggcagga	1				no_annot
11136	actacacaca	1				no_annot
11137	attaacattt	1				no_annot
11138	gtggtcattc	1				no_annot
11139	tggacgagaa	1	C300413.1	64	0	
11139	tggacgagaa	1	C716245.1	64	0	
11140	ggtaccagga	1				no_annot
11141	atggtgttgt	1				no_annot
11142	tgatgaacct	1	CD179701	32	1	similar to GP|16215389|emb FGF receptor-like protein 1 {Mus musculus}, partial (2%)
11143	tttataacaa	1				no_annot
11144	ataatacttt	1				no_annot
11145	agaagaccga	1				no_annot
11146	catcgaagca	1				no_annot
11147	aaatatccgt	1				no_annot
11148	atgaccaaaa	1				no_annot
11149	agataaaaat	1	C308911.1	9	4	
11150	aatcattatt	1	C711801.1	38	0	
11151	gcctgtgcca	1	CD069063	40	2	
11152	atggtgttga	1				no_annot
11153	tgatgaacca	1				no_annot
11154	acttcaacta	1				no_annot
11155	gggtatgtgc	1				no_annot
11156	acactcgaat	1				no_annot
11157	gaggcggatg	1				no_annot
11158	agggattgct	1				no_annot
11159	tctagccgat	1				no_annot
11160	gaggaaaatg	1	TC8526	50	0	

11161	catcatctgt	1	TC17280	93	0	similar to PIR|A31589|A31589 carboxypeptidase Cprecursor - human, partial (26%)
11162	ttcaaagtgc	1	TC11120	58	1	weakly similar to SP|O15173|PGC2_HUMAN Membrane associated progesterone receptor component 2 (Progesterone membrane binding protein) (Steroid receptor protein DG6). [Human] {Homo sapiens}, partial (30%)
11163	agggattgcc	1				no_annot
11164	tacttattta	1	C304845.1	32	0	
11164	tacttattta	1	C700049.1	31	0	
11165	gttcattgag	1				no_annot
11166	gtcaggtaga	1				no_annot
11167	attccagatt	1				no_annot
11168	gtgcttttgg	1				no_annot
11169	gtttcgtttt	1	TC18376	92	1	weakly similar to GP|30016922|gb|AAP04002.1||AY253299 alpha-galactosidase {Carica papaya}, partial (9%)
11170	gtcaaacagc	1				no_annot
11171	acagagtgat	1				no_annot
11172	catattggac	1				no_annot
11173	ttgtaaatgc	1				no_annot
11174	aggcggaagc	1	C700843.1	58	0	
11175	taaactttat	1				no_annot
11176	atactgcagc	1	TC10973	16	4	
11177	tgttgggcga	1				no_annot
11178	gtgataaaat	1				no_annot
11179	gaaattacta	1				no_annot
11180	tgtctgtaaa	1				no_annot
11181	ctcaggattg	1				no_annot
11182	ataaaaacta	1				no_annot
11183	ggattgttca	1				no_annot
11184	atctcccttt	1				no_annot
11185	tacgtattgt	1				no_annot

11186	gctaggaaca	1				no_annot
11187	tatacaggaa	1	TC14841	6	3	
11188	tgaaatgtta	1	AF030969|Sma.246	91	1	Clone 10 unknown
11189	cctatggcgt	1	C200665.1	5	1	
11190	gtgataaaac	1				no_annot
11191	gggctcagga	1				no_annot
11192	aaaagtattg	1				no_annot
11193	cggaaactgc	1	C202525.1	70	0	
11193	cggaaactgc	1	C611070.1	60	0	
11194	aaaagtattc	1				no_annot
11195	cacgtgtgta	1				no_annot
11196	agcaatggat	1				no_annot
11197	catttgaagt	1	TC12843	45	1	
11198	taaggcgctg	1				no_annot
11199	agcaatggag	1				no_annot
11200	ttcagcgttt	1				no_annot
11201	tttggatgca	1				no_annot
11202	gtgcaccatt	1				no_annot
11203	gtaatcaatg	1				no_annot
11204	atggtccttg	1				no_annot
11205	ctgtaaatgt	1	C606783.1	5	2	
11206	gtacagatgt	1				no_annot
11207	acaataatga	1	TC12942	82	0	
11208	ttttttcacc	1				no_annot
11209	agacaagtgg	1				no_annot
11210	caattatgtt	1	CD069570	3	3	
11211	gaacaattat	1	TC15006	1	2	
11212	agacaagtgc	1				no_annot
11213	ttgttgtcgt	1	TC17234	3	4	weakly similar to GP|28569596|gb|AAO42744.1||AY198392 mitochondrial transcription factor b1 {Rattus norvegicus}, partial (23%)

11214	gaataatgaa	1	TC7050	63	1	similar to GP|13171103|gb|AAK13589.1||AF139989 rRNA intron-encoded homing endonuclease {Oryza sativa}, partial (52%)
11214	gaataatgaa	1	TC16957	64	0	GP|4097684|gb|AAD00154.1||U66493 ubiquitin conjugating enzyme {Metarhizium anisopliae}, partial (12%)
11214	gaataatgaa	1	TC18710	60	2	similar to SP|P30876|RPB2_HUMAN DNA-directed RNA polymerase II 140 kDa polypeptide(RNA polymerase II subunit 2) (RPB2). [Human] {Homo sapiens}, partial (19%)
11215	ctctatttca	1				no_annot
11216	ctaaaagggg	1				no_annot
11217	ttgcgacaag	1				no_annot
11218	tacaaataaa	1				no_annot
11219	tatgatgtta	1				no_annot
11220	gcattgcata	1				no_annot
11221	tacattagaa	1				no_annot
11222	ttcattatga	1				no_annot
11223	gttttatgtt	1				no_annot
11224	aatatgcagt	1				no_annot
11225	ttttctttat	1				no_annot
11226	gacgtacgcg	1				no_annot
11227	ccgtgtctga	1				no_annot
11228	tgcctattaa	1				no_annot
11229	aaatgtggct	1	TC11457	68	0	similar to GP|12007713|gb|AAF31361.2||AF187863 glycine amidinotransferase {Xenopus laevis}, partial (52%)
11230	tcttcaggca	1	TC7759	83	1	
11230	tcttcaggca	1	TC14141	14	0	
11231	tgctaacctg	1				no_annot
11232	gggggtactg	1				no_annot
11233	gaatcactat	1	C201145.1	39	3	
11233	gaatcactat	1	C605497.1	38	3	
11234	tgctgaggtc	1	TC19753	6	0	

11235	ttttctttag	1	TC16822	94	0	
11236	ttagtaataa	1	TC7689	87	0	weakly similar to EGAD|2204|2151 collagen, type IV, alpha 5 (GB:M58526) {Homo sapiens}, partial (20%)
11237	tgttgagaga	1	TC8263	8	3	weakly similar to PIR|S44279|S44279 CDM protein - human, partial (23%)
11238	tgtatgtgtt	1	TC10074	6	2	
11239	aacaccatct	1				no_annot
11240	gactatggtt	1				no_annot
11241	tgaatgtaaa	1	C305328.1	65	0	
11242	gactgcatct	1				no_annot
11243	tacacaagca	1				no_annot
11244	tatacataaa	1				no_annot
11245	cattccattg	1				no_annot
11246	caacgtcctt	1				no_annot
11247	gttattcaat	1				no_annot
11248	tgtatgtgtg	1	TC7925	73	1	
11248	tgtatgtgtg	1	TC14885	39	3	
11249	aacaccatcg	1				no_annot
11250	gcaagaagca	1	AI977706	35	1	
11251	actttaagat	1	C301643.1	33	0	
11252	cggggtcact	1	C208817.1	20	3	
11253	catcatctca	1				no_annot
11254	gttattcaac	1	CD083543	77	0	similar to GP|17068385|gb|AAH17570.1|AAH17570|BC017570 hepatocellular carcinoma-associated antigen 59 {Homo sapiens}, partial (20%)
11255	cctgtgattc	1				no_annot
11256	tggggtcaaa	1				no_annot
11257	aactgatcga	1	CD117066	75	1	
11258	taaacacaac	1				no_annot
11259	ctagaggctg	1				no_annot
11260	ggcactgtgg	1	CD093237	10	4	

11261	atggtaacga	1				no_annot
11262	tccatatatc	1	TC11900	91	0	
11263	caagcatctc	1				no_annot
11264	tccaacaatt	1	TC7565	78	1	
11264	tccaacaatt	1	AI068001	25	4	weakly similar to PIR|A56525|A56525 lysosomal integral membrane protein II - human, partial (8%)
11265	gtgcagtaat	1				no_annot
11266	ctatattgaa	1				no_annot
11267	aatcagtgaa	1	C308388.1	36	0	
11268	gtgttttgat	1				no_annot
11269	atgaatgaga	1	CD189666	21	2	
11270	ccttcaggca	1				no_annot
11271	attggtatgg	1				no_annot
11272	tgggaaagtt	1				no_annot
11273	tcgttctctt	1				no_annot
11274	gggatgttgg	1	TC15090	37	3	
11275	tgttataact	1				no_annot
11276	agttcagtct	1				no_annot
11277	ttgtacaagt	1	TC14739	60	1	
11278	ccatcgccca	1	C211196.1	55	1	
11279	gtttcaactg	1				no_annot
11280	tagcataatc	1	AI977551	29	1	
11281	catacataaa	1	TC19211	19	4	
11282	ctcggactac	1	C608401.1	98	0	
11283	gactagtggg	1				no_annot
11284	attatcaagt	1	TC17028	70	0	weakly similar to SP|Q9D8B3|CTH8_MOUSE Protein c20orf178 homolog. [Mouse] {Mus musculus}, partial (54%)
11285	gtttcaacta	1	TC14527	54	2	similar to GP|28564890|gb|AAO32529.1||AY144966 YPL105C {Saccharomyces castellii}, partial (4%)
11286	agggacatta	1				no_annot
11287	aatgctcaca	1				no_annot

11288	ttttgttctt	1				no_annot
11289	gaaatctcga	1				no_annot
11290	cagctatacc	1				no_annot
11291	atataccaat	1	CD183394	38	0	
11292	atcatcagtg	1	CD060071	36	0	similar to SP|P09792|GT28_SCHMA Glutathione S-transferase 28 kDa(GST 28) (SM28 antigen) (Protective 28 kDa antigen) (GST class-alpha). [Blood fluke] {Schistosoma mansoni}, partial (36%)
11293	tgtatattgc	1				no_annot
11294	taaaatctat	1				no_annot
11295	ttattatttc	1				no_annot
11296	taattaagcc	1				no_annot
11297	actttttctt	1				no_annot
11298	aatacgttcc	1	TC11443	2	4	weakly similar to GP|505150|dbj|BAA06693.1||D31895 xpacxe2 protein {Xenopus laevis}, partial (31%)
11298	aatacgttcc	1	CD119799	16	0	weakly similar to SP|O15116|LSM1_HUMAN U6 snRNA-associated Sm-like protein LSm1 (Small nuclear ribonuclear CaSm) (Cancer-associated Sm-like). [Human] {Homo sapiens}, partial (31%)
11299	ttatacattt	1				no_annot
11300	gggacagttc	1				no_annot
11301	caagaagcag	1	TC8265	35	0	weakly similar to SP|Q01518|CAP1_HUMAN Adenylyl cyclase-associated protein 1 (CAP 1). [Human] {Homo sapiens}, partial (24%)
11302	aactctggct	1				no_annot
11303	acgccttatt	1	TC9561	71	0	
11303	acgccttatt	1	TC14403	80	0	weakly similar to PIR|T41265|T41265 fumarate hydrataseprecursor - fission yeast (Schizosaccharomyces pombe), partial (15%)
11304	aaagagtatg	1				no_annot
11305	tgcctgtggt	1				no_annot
11306	ttacaacaaa	1				no_annot
11307	cagcacaaca	1				no_annot
11308	tccataaaga	1				no_annot
11309	actatggtgg	1				no_annot

11310	ttttcacaac	1	TC17338	96	0	
11311	aggacctctg	1				no_annot
11312	ggagatactt	1				no_annot
11313	ggcgtaaact	1				no_annot
11314	cacacaagat	1				no_annot
11315	gtaaacattc	1				no_annot
11316	tacgtctaga	1				no_annot
11317	cacagattgt	1	TC7065	22	6	
11318	agcgttttca	1				no_annot
11319	gcatcaacta	1				no_annot
11320	gcttatcgac	1				no_annot
11321	ccatttcaaa	1	AA525632	74	1	
11322	aaaaagtgag	1				no_annot
11323	taagttacaa	1				no_annot
11324	tgctactgga	1	TC7947	36	0	weakly similar to GP|12653397|gb|AAH00468.1|AAH00468|BC000468 ubiquitin-conjugating enzyme E2 variant 1 {Homo sapiens}, partial (80%)
11325	aaaaagtgaa	1				no_annot
11326	agaagttggt	1				no_annot
11327	gttgtgcaat	1				no_annot
11328	tatctggtgc	1	TC12360	23	1	
11329	ggataaagtc	1	TC6959	31	9	
11330	agaatatatt	1				no_annot
11331	aaattcatat	1				no_annot
11332	tggttcctta	1	CD165315	76	0	
11333	aagttgtgcc	1	TC7933	73	0	
11333	aagttgtgcc	1	TC7934	34	1	weakly similar to GP|4809026|gb|AAD30062.1||AF132856 suppressor of G2 allele of skp1 homolog {Homo sapiens}, partial (32%)
11334	ttttgatggt	1				no_annot
11335	gtgtggtgga	1				no_annot

11336	gaacactaag	1				no_annot
11337	agaagttgga	1				no_annot
11338	ttttgtacgg	1				no_annot
11339	ccccagaaac	1				no_annot
11340	attttacgga	1				no_annot
11341	atagctcgta	1				no_annot
11342	tggtcgaatg	1				no_annot
11343	gattaactaa	1	TC15538	82	0	weakly similar to SP|P57076|CU59_HUMAN Protein C21orf59. [Human] {Homo sapiens}, partial (82%)
11344	taactaatac	1				no_annot
11345	tggtcgaata	1	TC14736	52	1	
11346	tgttgttttt	1	TC17091	93	0	weakly similar to SP|Q9H3H5|GPT_HUMAN UDP-N-acetylglucosamine--dolichyl-phosphate N-acetylglucosaminephosphotransferase(GPT) (G1PT) (N-acetylglucosamine-1-phosphate transferase) (GlcNAc-1-P transferase). [Human] {Homo sapiens}, partial (12%)
11347	aggaccacgg	1				no_annot
11348	gatcgaaatg	1				no_annot
11349	gtgagggctt	1				no_annot
11350	tgtacaaatt	1	TC8065	91	1	weakly similar to GP|12666720|emb|CAC27996.1||AJ303077 mitochondrial RNA splicing protein 3/4 {Homo sapiens}, partial (50%)
11351	ttctgactga	1				no_annot
11352	cacagcaaaa	1				no_annot
11353	atgaaactat	1				no_annot
11354	ttaattagaa	1				no_annot
11355	tatcgatgag	1				no_annot
11356	tagcaattcg	1				no_annot
11357	gggggaagcg	1				no_annot
11358	aattgctgaa	1				no_annot
11359	atttcaccta	1				no_annot
11360	tgacctgtcg	1				no_annot
11361	atggaggaga	1				no_annot

11362	cgcggatagg	1				no_annot
11363	ttcacaatca	1				no_annot
11364	attaactttg	1	C200067.1	42	0	
11364	attaactttg	1	C602098.1	45	0	
11365	gtgagggctc	1				no_annot
11366	tgcgatagaa	1				no_annot
11367	tataaacaac	1				no_annot
11368	atgacctaac	1				no_annot
11369	ttgggcatat	1				no_annot
11370	gaatcccaaa	1	TC12023	89	0	similar to GP|24196227|gb|AAN49656.1|AE011413_10|AE011413 methionine aminopeptidase {Leptospira interrogans serovar lai str. 56601}, partial (14%)
11371	gaacataagg	1				no_annot
11372	agataataat	1				no_annot
11373	aataacagca	1	CD197411	40	0	homologue to PIR|T01246|T01246 30S ribosomal protein S31 [imported] - Arabidopsis thaliana, partial (14%)
11374	tggactaaca	1	TC7739	86	0	similar to GP|12654791|gb|AAH01238.1|AAH01238|BC001238 DEAD/H (Asp-Glu-Ala-Asp/His) box polypeptide 18 (Myc-regulated) {Homo sapiens}, partial (43%)
11375	ggaagatccg	1	TC14449	13	2	similar to GP|7299916|gb|AAF55090.1||AE003706 CG7552-PA {Drosophila melanogaster}, partial (16%)
11376	atagaggttg	1				no_annot
11377	gattatgaaa	1				no_annot
11378	acgtcttccg	1				no_annot
11379	ctgttgtcat	1				no_annot
11380	ttgtaattgt	1				no_annot
11381	attatcaaca	1	CD147007	95	0	similar to GP|1546894|emb|CAA69250.1||Y07952 ssRNA-binding protein {Dictyostelium discoideum}, partial (6%)
11382	aggaagtcga	1				no_annot
11383	ttgatcaatc	1				no_annot
11384	aaagtacatt	1				no_annot

11385	tttccctcca	1				no_annot
11386	tcttagccac	1	TC14981	88	0	similar to GP|10039641|gb|AAG12204.1|AF287482_5|AF287482 Orf122 {Chlorobium tepidum}, partial (69%)
11387	ctagtcaaat	1				no_annot
11388	aaaagcttcc	1				no_annot
11389	aataatagtg	1				no_annot
11390	gctgaccttt	1				no_annot
11391	tgtgtgaatg	1				no_annot
11392	aaatcaattc	1				no_annot
11393	ttgttataat	1	C302128.1	64	0	
11393	ttgttataat	1	C703382.1	64	0	
11394	caactaatac	1				no_annot
11395	taattttcta	1				no_annot
11396	gggatgttat	1				no_annot
11397	gggctctggt	1				no_annot
11398	acaaactttc	1				no_annot
11399	atagatcacc	1				no_annot
11400	agttgagctg	1	C603500.1	52	0	
11401	cttacgaaaa	1				no_annot
11402	ttgttataac	1				no_annot
11403	tggttgttaa	1				no_annot
11404	aaggatttgt	1	TC17985	49	0	
11405	tgttgtatga	1				no_annot
11406	cataggtaaa	1	AI974996	30	1	similar to GP|25012383|gb|AAN71300.1||BT001545 RE10625p {Drosophila melanogaster}, partial (5%)
11407	gaaagaaatt	1				no_annot
11408	tattcctttt	1	TC14412	93	0	
11409	aaagaagtat	1				no_annot
11410	cagaacagta	1				no_annot
11411	acacagttac	1				no_annot
11412	tgacactgct	1				no_annot

11413	aaagaagtag	1				no_annot
11414	cgatgtcttg	1	C716634.1	79	0	
11415	attatcaaaa	1				no_annot
11416	tatttccatt	1				no_annot
11417	gattcgtgat	1				no_annot
11418	atcaaaaatt	1	TC13764	98	0	
11419	tgcttttcag	1				no_annot
11420	tatgcagaga	1				no_annot
11421	tgcccaaaga	1				no_annot
11422	ccaaacacat	1	C203884.1	31	0	
11422	ccaaacacat	1	C600330.1	28	0	
11423	taattatgga	1	C608312.1	89	0	
11424	gaggcaagtg	1				no_annot
11425	gcgattactc	1				no_annot
11426	caattctcat	1				no_annot
11427	acctcagagc	1				no_annot
11428	tcaagggtat	1	C306926.1	91	0	
11429	taggtgactc	1				no_annot
11430	ctgttataaa	1				no_annot
11431	ttagatatta	1	CD076693	73	0	
11432	atcctctaaa	1				no_annot
11433	ggtgaacttt	1				no_annot
11434	atatctggca	1				no_annot
11435	tgagtaagta	1				no_annot
11436	tacaatcctg	1	C206674.1	72	0	
11436	tacaatcctg	1	C609157.1	24	5	
11437	ttgcaaactt	1				no_annot
11438	tatcgttcga	1				no_annot
11439	tagggttgtc	1				no_annot
11440	actatcagta	1				no_annot

11441	gctttgtagc	1				no_annot
11442	gtaataataa	1	TC15299	34	0	weakly similar to GP|6425002|gb|AAF08254.1|AF173552_1|AF173552 Cry1A toxin receptor A {Heliothis virescens}, partial (3%)
11442	gtaataataa	1	CD064106	70	1	
11443	attatgagcg	1				no_annot
11444	aagtccgccc	1				no_annot
11445	tgggttcttg	1				no_annot
11446	cacttattga	1				no_annot
11447	tttaactcgc	1				no_annot
11448	tgcaccactg	1	TC19432	60	0	
11449	gtgaagagca	1				no_annot
11450	cacatttgat	1				no_annot
11451	gctactgcat	1	TC11215	22	2	weakly similar to GP|1079683|gb|AAB68306.1||U39205 Lpe13p {Saccharomyces cerevisiae}, partial (41%)
11452	tcccctaatc	1				no_annot
11453	gtgaaattca	1				no_annot
11454	tgaacagacc	1				no_annot
11455	gaaatgcacc	1				no_annot
11456	aacatccaca	1				no_annot
11457	gccatttgcg	1				no_annot
11458	ttgaacattt	1				no_annot
11459	cgcggataaa	1				no_annot
11460	gatgatcagc	1				no_annot
11461	ctcattttga	1				no_annot
11462	tacactacat	1				no_annot
11463	gaggtctggt	1				no_annot
11464	ccaactgatc	1	TC7454	62	6	similar to GP|21392234|gb|AAM48471.1||AY118442 SD06874p {Drosophila melanogaster}, partial (78%)
11465	aacacttttg	1				no_annot
11466	gcaagtacat	1				no_annot

11467	aatatttcga	1				no_annot
11468	gtgttggctt	1				no_annot
11469	ggtttagaag	1				no_annot
11470	tacactacaa	1				no_annot
11471	cgacattgtc	1				no_annot
11472	gaagctcaac	1				no_annot
11473	tgtcaatcga	1				no_annot
11474	tgtgagatta	1				no_annot
11475	ggatcagtaa	1				no_annot
11476	ttcatacagt	1	CD169487	35	1	
11477	gtgacagcgg	1				no_annot
11478	tccctgcccc	1				no_annot
11479	tctccgtaca	1				no_annot
11480	ttccaatgcg	1	C202388.1	58	0	
11480	ttccaatgcg	1	C609211.1	43	0	
11481	ttccagcaaa	1				no_annot
11482	agtcactatt	1	TC17124	78	0	weakly similar to GP|5052652|gb|AAD38656.1|AF145681_1|AF145681 BcDNA.LD23181 {Drosophila melanogaster}, partial (9%)
11483	caatcgttca	1	TC10753	89	1	weakly similar to GP|161038|gb|AAA29903.1||M15509 major egg antigen {Schistosoma mansoni}, partial (24%)
11484	taattatgct	1	C208828.1	45	0	
11485	cacgaggcaa	1				no_annot
11486	tccttttgat	1	C610300.1	94	0	
11487	gaaaagctgc	1	TC7339	60	8	similar to GP|6942068|gb|AAF32311.1|AF217788_1|AF217788 O-glycosyltransferase {Drosophila melanogaster}, partial (81%)
11488	gactggccta	1				no_annot
11489	aaaggtcgaa	1	CD120128	13	3	
11490	gtgaagagaa	1				no_annot
11491	ttgtaattac	1				no_annot

11492	tcttgactgc	1				no_annot
11493	tgaacagaag	1				no_annot
11494	aaagatgatt	1	CD119262	51	0	
11495	tactacatca	1				no_annot
11496	ccccctaata	1				no_annot
11497	tgttgtatag	1				no_annot
11498	gtctaaacgg	1				no_annot
11499	ttgtttgcca	1				no_annot
11500	tacatcattg	1				no_annot
11501	gtatgcttga	1	TC11098	74	0	
11502	gcatcatctt	1				no_annot
11503	gttatggtca	1				no_annot
11504	tcccttttaa	1				no_annot
11505	gtaaactttg	1				no_annot
11506	tatcgttcca	1				no_annot
11507	agcaatcggt	1				no_annot
11508	tatagtgacg	1				no_annot
11509	ggttagaaga	1				no_annot
11510	cctccgtact	1	TC7455	80	1	weakly similar to SP|P13395|SPCA_DROME Spectrin alpha chain. [Fruit fly] {Drosophila melanogaster}, partial (21%)
11510	cctccgtact	1	BE505114	46	0	similar to GP|17862818|gb|AAL39886.1||AY069741 LP06350p {Drosophila melanogaster}, partial (4%)
11511	ttattcaacg	1				no_annot
11512	atcggataaa	1				no_annot
11513	gaggaaaaaa	1				no_annot
11514	tctgtgatct	1	TC7358	84	1	similar to SP|O00505|IMA3_HUMAN Importin alpha-3 subunit (Karyopherin alpha-3 subunit) (SRP1-gamma). [Human] {Homo sapiens}, partial (85%)
11515	gcgggttgtc	1				no_annot
11516	tacttattag	1				no_annot
11517	ggagagcagc	1				no_annot

11518	gtcccggatc	1				no_annot
11519	gtcgcacatt	1				no_annot
11520	taagtttcac	1				no_annot
11521	taagattgta	1				no_annot
11522	gaagtatata	1				no_annot
11523	cttgcggata	1	TC12131	52	1	similar to GP|7296948|gb|AAF52221.1||AE003608 CG8680-PA {Drosophila melanogaster}, partial (39%)
11524	gttgctgtaa	1	TC12175	35	0	weakly similar to GP|12003972|gb|AAG43826.1|AF212367_1|AF212367 spinster type II {Drosophila melanogaster}, partial (7%)
11525	tctaatgata	1				no_annot
11526	aattctcaat	1	BE505115	42	0	
11527	atttttccgg	1				no_annot
11528	taatatagta	1				no_annot
11529	ggagcgcgtt	1				no_annot
11530	gagggatttt	1				no_annot
11531	ttcaccgggt	1	C202265.1	54	3	
11531	ttcaccgggt	1	C611330.1	44	3	
11532	ttagtttata	1	TC7783	92	0	similar to SP|Q27954|COPA_BOVIN Coatomer alpha subunit (Alpha-coat protein) (Alpha-COP) (HEPCOP) (HEP-COP) [Contains: Xenin (Xenopsin-related peptide); Proxenin]. [Bovine] {Bos taurus}, partial (26%)
11533	caaatggtac	1				no_annot
11534	gactgacatt	1				no_annot
11535	aacgaagctt	1				no_annot
11536	ttcattttaa	1				no_annot
11537	gtatataaca	1	AF515706|Sma.79	93	0	Fes-like tyrosine kinase protein
11538	cactacatca	1				no_annot
11539	cacgtgtgat	1				no_annot
11540	gactattagg	1				no_annot
11541	ttgtatggta	1				no_annot
11542	tattctattg	1	TC14090	86	0	
11543	cttcatcctt	1				no_annot

11544	ccagaaaatt	1				no_annot
11545	ggtccgattg	1				no_annot
11546	cgtatttata	1				no_annot
11547	attcttcatt	1	TC9655	52	2	
11547	attcttcatt	1	TC10145	72	0	
11548	taacacatat	1				no_annot
11549	tgtacatatt	1	TC10743	89	0	GP|12239372|gb|AAG49449.1|AF158103_1|AF158103 FTZ-F1 {Schistosoma mansoni}, complete
11549	tgtacatatt	1	CD169276	60	0	weakly similar to SP|Q04656|AT7A_HUMAN Copper-transporting ATPase 1(Copper pump 1) (Menkes disease-associated protein). [Human] {Homo sapiens}, partial (3%)
11550	ccggaagcaa	1				no_annot
11551	atcaccacta	1	CD129935	45	4	
11552	cactatattt	1	AI395775	51	1	
11553	aagacaatta	1				no_annot
11554	ttccggctga	1				no_annot
11555	atgtaggagt	1				no_annot
11556	tgtggacaat	1	CD169397	94	0	
11557	tacaggtgcc	1				no_annot
11558	aatttgacgt	1				no_annot
11559	ggcagaggca	1				no_annot
11560	gccttattct	1				no_annot
11561	tcctggtgga	1				no_annot
11562	tgtacatatc	1				no_annot
11563	cctgtaactg	1				no_annot
11564	aatcaccgaa	1				no_annot
11565	aactcgattg	1	TC15536	57	1	
11566	atgtttgtgt	1				no_annot
11567	aataactgca	1				no_annot
11568	ttgtgtatta	1				no_annot
11569	gacggacagt	1				no_annot

11570	gtctccttgt	1	TC11182	27	0	weakly similar to SP|Q12522|IF6_YEAST Eukaryotic translation initiation factor 6 (eIF-6). [Baker's yeast] {Saccharomyces cerevisiae}, partial (88%)
11571	tatgctatgg	1				no_annot
11572	ttctgccaac	1				no_annot
11573	cgtgtgggtg	1				no_annot
11574	attatgtggt	1				no_annot
11575	tatgatgtaa	1				no_annot
11576	aatatttcat	1	TC11261	95	0	homologue to GP|28916470|gb|AAO59418.1||AY220751 calcineurin B {Schistosoma japonicum}, complete
11576	aatatttcat	1	CD110941	76	0	
11577	gtcgatcttg	1				no_annot
11578	acggtcttaa	1				no_annot
11579	ctagtttata	1				no_annot
11580	tttacgcgta	1				no_annot
11581	cattaatctc	1				no_annot
11582	ctgtttgcag	1	TC11061	22	0	
11582	ctgtttgcag	1	CD097985	51	0	
11583	tgccatataa	1				no_annot
11584	gaccccttat	1				no_annot
11585	ccacatcctc	1				no_annot
11586	gaaactgtgc	1				no_annot
11587	ataatccact	1				no_annot
11588	tgtcacctaa	1				no_annot
11589	tcgggaggtg	1				no_annot
11590	cagatttctt	1				no_annot
11591	tccaacaact	1	CD074846	40	2	
11592	tatatgatgt	1				no_annot
11593	aatctgaatc	1	TC18358	28	0	
11594	gccgtgtgag	1				no_annot
11595	atttattaca	1	TC12852	59	0	
11595	atttattaca	1	TC17580	88	0	

11595	atttattaca	1	BF936551	71	0	D. melanogaster CG6949 gene product
11596	tgctgaggaa	1	CD076203	16	1	
11597	ttgctgtaat	1				no_annot
11598	gaccacctgg	1	TC19085	3	0	weakly similar to PIR|S23809|S23809 collagen alpha 2(I) chain homolog - sea urchin (Strongylocentrotus purpuratus), partial (11%)
11598	gaccacctgg	1	AI067421	45	0	
11599	taacttggtg	1				no_annot
11600	ctattcaaat	1				no_annot
11601	ggtagtttgt	1				no_annot
11602	aatagaaaat	1	C210026.1	28	0	
11602	aatagaaaat	1	C600599.1	7	3	
11603	tgtatgtgat	1				no_annot
11604	taatgatatt	1	CD064680	65	1	
11605	aaaatagttc	1				no_annot
11606	acggttatgg	1				no_annot
11607	taaaagacta	1				no_annot
11608	taaagttcat	1				no_annot
11609	aaagaccatc	1				no_annot
11610	aaaaatcgtc	1	TC14427	88	0	
11611	gcagattgtc	1				no_annot
11612	acacaatcga	1				no_annot
11613	tccaataatt	1	C201074.1	60	2	
11613	tccaataatt	1	C610021.1	74	1	
11614	cttattctga	1				no_annot
11615	cttctcattt	1				no_annot
11616	ttattattcg	1	TC13976	56	0	
11617	acacctatga	1				no_annot
11618	aatgaaatat	1				no_annot
11619	tgaaatcttt	1				no_annot
11620	gtactactac	1				no_annot

11621	gtctttgaac	1	CD077969	35	0	
11622	ccacctacac	1				no_annot
11623	tactaggcct	1				no_annot
11624	gcttagttct	1				no_annot
11625	gcagactgag	1				no_annot
11626	ttgataataa	1				no_annot
11627	tctgttgttg	1	CD078781	46	0	
11628	cgccatataa	1				no_annot
11629	tacttttatg	1				no_annot
11630	tcaaaatggc	1				no_annot
11631	cattagttag	1	C610034.1	93	0	
11632	cattagttac	1				no_annot
11633	cgtgtgtatg	1				no_annot
11634	aagatggcat	1	CD077019	71	0	
11635	tctttgtttg	1				no_annot
11636	tcacgtaagc	1	TC17206	78	0	weakly similar to PIR|S45251|S45251 SNF2alpha protein - human, partial (7%)
11637	atgtcagtgg	1	TC11272	53	4	similar to PIR|JC2378|JC2378 acetyl-CoA C-acetyltransferase, cytosolic - human, partial (32%)
11637	atgtcagtgg	1	CD113954	53	0	similar to GP|7293068|gb|AAF48453.1||AE003499 CG11654-PA {Drosophila melanogaster}, partial (28%)
11638	aaccgataaa	1				no_annot
11639	agtcaattga	1	TC7317	47	1	
11639	agtcaattga	1	TC7323	8	1	
11639	agtcaattga	1	TC7326	46	0	
11639	agtcaattga	1	TC7327	66	1	
11639	agtcaattga	1	TC12840	25	3	weakly similar to PIR|T39903|T39903 serine-rich protein - fission yeast (Schizosaccharomyces pombe), partial (5%)
11639	agtcaattga	1	TC15792	85	1	
11639	agtcaattga	1	TC16151	13	4	
11639	agtcaattga	1	TC18037	79	0	

11639	agtcaattga	1	BE431234	72	1	
11639	agtcaattga	1	CD086206	30	0	
11639	agtcaattga	1	CD112184	41	0	
11639	agtcaattga	1	CD112865	57	0	
11639	agtcaattga	1	CD154428	60	1	
11639	agtcaattga	1	CD189458	74	1	
11639	agtcaattga	1	CD194579	27	0	
11639	agtcaattga	1	AI014233	63	1	
11639	agtcaattga	1	CD069252	93	1	
11639	agtcaattga	1	CD074715	48	2	
11639	agtcaattga	1	CD081776	3	0	
11639	agtcaattga	1	CD087645	94	0	
11639	agtcaattga	1	CD090074	73	1	
11639	agtcaattga	1	CD092870	55	2	
11639	agtcaattga	1	CD094438	84	0	
11639	agtcaattga	1	CD133280	71	0	
11639	agtcaattga	1	CD137193	42	1	
11639	agtcaattga	1	CD170844	57	0	
11639	agtcaattga	1	AI976121	50	1	repeat sequences
11639	agtcaattga	1	CD197227	12	0	homologue to GP|1669533|dbj|BAA09798.1||D63643 clathrin coat assembly protein-like {Homo sapiens}, partial (6%)
11639	agtcaattga	1	CD185272	71	1	
11640	tgagcgatga	1	TC10984	80	2	similar to GP|24286644|gb|AAN46874.1||AY160094 nucleotide exchange factor RasGEF E {Dictyostelium discoideum}, partial (3%)
11641	gacaaggcca	1				no_annot
11642	atccatatcg	1	AI976755	25	3	
11643	gattgcctaa	1				no_annot
11644	ttaatcatta	1	CD116819	39	0	
11645	tttattctcc	1				no_annot
11646	taacacttgt	1	TC16655	77	2	

11647	tgattaagtt	1				no_annot
11648	gaaagaaagg	1	CD113968	42	0	
11649	ttcagccttc	1				no_annot
11650	gtggccaatt	1				no_annot
11651	tatgatcttg	1				no_annot
11652	acactgcgca	1				no_annot
11653	tctgtgttgt	1	C612146.1	86	1	
11654	gagttgctag	1				no_annot
11655	ctcatacaaa	1				no_annot
11656	taaacggata	1				no_annot
11657	cctgaggata	1				no_annot
11658	taaaaaaata	1				no_annot
11659	tatatcgtgt	1				no_annot
11660	gcttagttat	1				no_annot
11661	gacattaagt	1				no_annot
11662	tccagctttc	1				no_annot
11663	agcgctaaaa	1				no_annot
11664	tcgctcaacg	1	C604219.1	40	3	
11665	tcatagactt	1				no_annot
11666	ttcacctact	1				no_annot
11667	tgttgtttct	1				no_annot
11668	aagaggatta	1	C206862.1	61	1	
11668	aagaggatta	1	C609700.1	61	1	
11669	ttgatgtgta	1				no_annot
11670	gtaaacatac	1				no_annot
11671	attaacttct	1				no_annot
11672	tctttgatgg	1				no_annot
11673	tagatgcaac	1				no_annot
11674	gaggaaggag	1				no_annot
11675	gtggaagtgg	1				no_annot

11676	tttaaatgca	1	TC13797	89	0	
11676	tttaaatgca	1	TC13800	88	0	
11676	tttaaatgca	1	TC17889	73	5	
11677	cagatgcacc	1	TC7037	2	8	similar to PIR|S20638|S20638 desmoyokin - mouse (fragment), partial (4%)
11677	cagatgcacc	1	TC7037	2	5	similar to PIR|S20638|S20638 desmoyokin - mouse (fragment), partial (4%)
11677	cagatgcacc	1	TC7074	2	1	
11677	cagatgcacc	1	TC7212	77	2	
11677	cagatgcacc	1	CD062077	12	1	
11677	cagatgcacc	1	CD094051	1	2	
11677	cagatgcacc	1	CD186116	39	2	similar to GP|6735500|emb|CAB69071.1||AJ271107 dsr S {Leuconostoc mesenteroides}, partial (23%)
11678	aaagttttaa	1	TC7831	34	4	weakly similar to GP|12060812|gb|AAG48248.1|AF282407_1|AF282407 p70 ribosomal protein S6 kinase {Artemia franciscana}, partial (57%)
11678	aaagttttaa	1	TC14671	90	0	weakly similar to GP|14278850|gb|AAK27730.1||AY028423 PRIP-interacting protein PIPMT {Homo sapiens}, partial (12%)
11679	gaggcaagga	1				no_annot
11680	cctacattga	1	TC8473	58	0	similar to GP|2895276|gb|AAC39596.1||AF026029 poly(A) binding protein II {Homo sapiens}, partial (7%)
11681	tgtaaacata	1	C610098.1	58	4	
11681	tgtaaacata	1	C612269.1	1	6	
11682	gtggagtatg	1				no_annot
11683	gaccatcttt	1	TC11402	91	0	similar to GP|23095606|gb|AAF50701.2||AE003564 CG13293-PA {Drosophila melanogaster}, partial (30%)
11684	acttgaaaaa	1				no_annot
11685	aagtcataag	1				no_annot
11686	gcgcataacc	1	U35432|Sma.1149	58	1	Rab-related GTP-binding protein
11687	aagaaagtgc	1				no_annot
11688	taatcatcga	1	TC10608	9	5	
11689	cttactgagg	1				no_annot

11690	cagttgactg	1	TC8281	19	0	similar to GP|16307142|gb|AAH09658.1||BC009658 serine/threonine kinase 38 {Mus musculus}, partial (21%)
11691	ggactataga	1				no_annot
11692	cccgaagttt	1				no_annot
11693	atgtttgtag	1				no_annot
11694	gttctgtgga	1	TC17557	53	2	
11695	caatgaaaga	1	C209722.1	55	2	
11695	caatgaaaga	1	C607638.1	54	2	
11696	tagtgtaggt	1				no_annot
11697	gtttgaaagt	1				no_annot
11698	cgtaatatga	1				no_annot
11699	gaggaataca	1				no_annot
11700	tacatagatg	1	C201157.1	19	1	
11700	tacatagatg	1	C602181.1	19	1	
11701	atttgtttgg	1				no_annot
11702	ggtacttggc	1				no_annot
11703	tgtgtagtag	1				no_annot
11704	gtatatggcg	1				no_annot
11705	gtctgaagtg	1				no_annot
11706	aagtatttca	1	TC13918	84	1	
11707	gtatcttgta	1				no_annot
11708	tggtatactg	1	TC7626	63	0	weakly similar to GP|10727262|gb|AAG22234.1||AE003477 CG11486-PA {Drosophila melanogaster}, partial (11%)
11709	caaaaaaata	1				no_annot
11710	tcccctaagt	1				no_annot
11711	ttatttggtg	1	TC8682	74	0	weakly similar to GP|15292429|gb|AAK93483.1||AY052059 LP08712p {Drosophila melanogaster}, partial (16%)
11711	ttatttggtg	1	CD187303	55	0	
11712	attaattttg	1				no_annot
11713	tgtcttcagt	1	TC9226	30	2	

11714	gttgaaccct	1				no_annot
11715	ttcaaactat	1	TC17261	92	0	weakly similar to SP|Q9U6P6|RNHL_CAEEL Ribonuclease HI large subunit(RNase HI large subunit) (RNase H(35)). {Caenorhabditis elegans}, partial (62%)
11716	cctttgatgt	1				no_annot
11717	acaaacttca	1				no_annot
11718	gtcatcgtaa	1	TC7649	67	1	similar to PIR|I38176|I38176 ras-related GTPase rag, splice form A [imported] - human, partial (70%)
11718	gtcatcgtaa	1	CD082047	48	0	similar to PIR|I38176|I38176 ras-related GTPase rag, splice form A [imported] - human, partial (6%)
11719	tggcaatttg	1				no_annot
11720	aacagcacaa	1				no_annot
11721	ttagcaaaca	1				no_annot
11722	gtatgtatct	1				no_annot
11723	cagatgcaac	1				no_annot
11724	cgcgtaatga	1				no_annot
11725	gttctctaaa	1				no_annot
11726	ttgacgactg	1				no_annot
11727	aatgaattga	1	AF009659|Sma.739	56	1	C-GMP dependent protein kinase (SMPK)
11728	tttcgtaaaa	1	TC7915	11	2	weakly similar to SP|O73747|FRG1_FUGRU FRG1 protein. [Japanese pufferfish, Takifugu rubripes] {Fugu rubripes}, partial (18%)
11728	tttcgtaaaa	1	CD073208	34	0	
11729	tgattctctc	1				no_annot
11730	ttgacgacta	1				no_annot
11731	acattgaatc	1	CD188188	44	1	
11732	aaaagtttcc	1	C201332.1	49	1	
11732	aaaagtttcc	1	C604239.1	43	1	
11733	tcttgccaag	1				no_annot
11734	ttcttgttgg	1				no_annot
11735	tacttgtgaa	1				no_annot
11736	gcgaagctgt	1				no_annot
11737	gaagcagctg	1				no_annot

11738	taattttcat	1	TC14730	96	0	similar to GP|1370092|emb|CAA65449.1||X96669 kinase {Gallus gallus}, partial (10%)
11739	tttgcggaga	1				no_annot
11740	gcaaaaaatg	1	TC16849	23	1	similar to PIR|S34109|S34109 ribosomal protein S13, cytosolic [validated] - human, complete
11741	aacagaaggg	1				no_annot
11742	aatggagatt	1				no_annot
11743	ttagctgctt	1				no_annot
11744	gtccttagtg	1				no_annot
11745	gagtaaaaaa	1				no_annot
11746	aaatcaatag	1	TC19654	9	4	weakly similar to GP|18044822|gb|AAH20002.1||BC020002 cDNA sequence BC020002 {Mus musculus}, partial (3%)
11747	ctgatcaaca	1				no_annot
11748	acgattccga	1				no_annot
11749	tgctattgca	1				no_annot
11750	ggatttctgt	1				no_annot
11751	tcgctgagct	1				no_annot
11752	actcaacata	1				no_annot
11753	ggacagtgtc	1				no_annot
11754	cctaagcgtg	1				no_annot
11755	gctatgatcc	1	TC7726	67	0	
11755	gctatgatcc	1	TC7955	66	1	weakly similar to GP|3326936|emb|CAA07014.1||AJ006408 alkaline phosphodiesterase I {Fowlpox virus}, partial (6%)
11756	agttgagcaa	1	C200662.1	53	0	
11757	cagagaggtt	1				no_annot
11758	taatctcttt	1				no_annot
11759	ttatacttga	1	CD069145	24	2	
11759	ttatacttga	1	CD076299	8	0	
11760	gataatgtca	1				no_annot
11761	tatattgttg	1				no_annot
11762	agtacaacta	1				no_annot

11763	aatggctttg	1				no_annot
11764	gtgccttacc	1	CD186610	95	0	
11765	attcgtacta	1				no_annot
11766	taacacttag	1				no_annot
11767	aattgcattt	1				no_annot
11768	gtcgtggttt	1				no_annot
11769	tttttctgtt	1	C700865.1	37	1	
11770	taacacttaa	1				no_annot
11771	acaaatatgg	1				no_annot
11772	gaattaaagt	1				no_annot
11773	ttgatcgggc	1	C205531.1	37	0	
11774	ttaagtaaaa	1	C211158.1	91	0	
11774	ttaagtaaaa	1	C611788.1	91	0	
11775	ttgctccagt	1	C202846.1	58	0	
11775	ttgctccagt	1	C606716.1	23	0	
11776	ttccgctgct	1				no_annot
11777	tgccatttat	1	AI976735	44	0	
11778	aaggtgtttt	1	L15635|Sma.31	76	0	Vif related mRNA sequence
11779	tatatcgtaa	1				no_annot
11780	gcgttaaatg	1				no_annot
11781	cactttaacc	1				no_annot
11782	ccagtacctg	1				no_annot
11783	gttccaaagt	1				no_annot
11784	gtggaagtag	1				no_annot
11785	tatctcagca	1				no_annot
11786	tgtgaaactt	1	TC19130	79	0	
11787	taacatatgc	1	TC15520	94	0	
11788	attattgggg	1				no_annot
11789	ctactgttga	1				no_annot
11790	caatgaaaat	1	TC12376	16	0	

11791	caacatttta	1				no_annot
11792	ccctcttcat	1				no_annot
11793	tagtaaattt	1				no_annot
11794	tggcgcattg	1				no_annot
11795	attaggaagt	1				no_annot
11796	tccagtatta	1	C611528.1	60	0	
11797	tagatttcgc	1				no_annot
11798	tgcaagtccg	1				no_annot
11799	taatgaggga	1				no_annot
11800	taacttaaaa	1	CD179193	3	1	
11801	atattggaca	1				no_annot
11802	atgcaatttg	1				no_annot
11803	ccctgtgggg	1				no_annot
11804	caacacttag	1	C307352.1	45	1	
11804	caacacttag	1	C604928.1	28	4	
11805	tacagttact	1				no_annot
11806	gccttgtgac	1				no_annot
11807	gtgttcttgg	1				no_annot
11808	gggtgatgag	1				no_annot
11809	gtacatcttt	1	CD199305	17	1	
11810	aaactgcatc	1				no_annot
11811	ttaggagtac	1				no_annot
11812	acaaccgcca	1				no_annot
11813	agcaccttta	1				no_annot
11814	gatcaaccct	1	CD072958	26	0	
11815	tgcaccacag	1				no_annot
11816	ttgccagact	1				no_annot
11817	gtgagtatga	1	TC14196	21	3	weakly similar to SP|O35587|TM21_MESAU Transmembrane protein Tmp21 precursor (21 kDa Transmembrane trafficking protein) (Integral membrane protein p23). [Golden hamster] {Mesocricetus auratus}, partial (39%)

11818	tggtaaagca	1				no_annot
11819	ggtctcacac	1	C316576.1	38	0	
11820	ataagtacta	1	C606449.1	20	2	
11821	gttatcagga	1	TC12394	23	3	
11822	accaaaagta	1				no_annot
11823	ctagtttaga	1				no_annot
11824	gagcgcgttt	1				no_annot
11825	gatacacata	1				no_annot
11826	tacgaacagc	1				no_annot
11827	cccagtattt	1				no_annot
11828	ggtgatgaaa	1				no_annot
11829	tactctgcta	1				no_annot
11830	actacaagtt	1	AI739702	36	0	
11831	gagtttagga	1				no_annot
11832	aagaagaaat	1	TC12148	4	1	
11832	aagaagaaat	1	TC17184	42	1	
11832	aagaagaaat	1	CD153468	7	1	
11832	aagaagaaat	1	AA952828	83	0	
11832	aagaagaaat	1	CD189144	37	1	
11833	cgctgtgcaa	1				no_annot
11834	ctgaacatcg	1	CD081067	73	2	
11835	aacttcacag	1				no_annot
11836	aacctattta	1	TC16317	73	0	
11837	tctacttatt	1	C207432.1	55	0	
11838	caatcatcaa	1	TC11931	16	3	weakly similar to SP|Q9R0E1|PLO3_MOUSE Procollagen-lysine,2-oxoglutarate 5-dioxygenase 3 precursor(Lysyl hydroxylase 3) (LH3). [Mouse] {Mus musculus}, partial (12%)
11839	gatgctcgat	1				no_annot
11840	aaaatgtatt	1	C313205.1	60	1	
11841	catcaatatg	1				no_annot
11842	gtaaaagaat	1				no_annot

11843	cgtattggat	1	TC8731	45	0	
11844	tacagttaat	1	TC7104	30	0	similar to GP|18873701|emb|CAD10446.1||AJ417719 cytochrome b {Necator americanus}, partial (5%)
11844	tacagttaat	1	TC12605	90	0	
11845	tgcttatttc	1	TC18210	44	0	
11846	gtttcatcag	1				no_annot
11847	gtattgaatg	1	TC17166	30	1	
11848	caggtaaacg	1				no_annot
11849	ttagtttact	1				no_annot
11850	tcgtagctac	1				no_annot
11851	gcagattgga	1				no_annot
11852	tacattattc	1				no_annot
11853	cattcacatt	1	CD068884	21	8	
11854	ctaacttcaa	1				no_annot
11855	tcaactcatt	1				no_annot
11856	cagagaaagg	1	TC8627	21	4	weakly similar to GP|15010482|gb|AAK77289.1||AY047557 GH07062p {Drosophila melanogaster}, partial (10%)
11857	taaccaaaaa	1				no_annot
11858	tgtgtgggcc	1				no_annot
11859	tgattatgta	1	TC14066	49	3	weakly similar to SP|Q95K73|ELO4_MACFA Elongation of very long chain fatty acids 4 protein. [Crab eating macaque, Cynomolgus monkey] {Macaca fascicularis}, partial (22%)
11860	tatgcgccct	1	AY277535|Sma.73	31	7	Isolate S47 high voltage-activated calcium channel beta subunit 1
11860	tatgcgccct	1	gi|33465408|gb|AY277536.1|	31	7	Schistosoma mansoni isolate S47 high voltage-activated calcium channel beta subunit 1 mRNA, complete cds
11860	tatgcgccct	1	gi|33465404|gb|AY277534.1|	31	7	Schistosoma mansoni strain Gene Pool high voltage-activated calcium channel beta subunit 1 mRNA, complete cds
11860	tatgcgccct	1	gi|33465402|gb|AY277533.1|	31	7	Schistosoma mansoni strain Puerto Rican high voltage-activated calcium channel beta subunit 1 mRNA, complete cds
11860	tatgcgccct	1	gi|15283996|gb|AY033598.1|	31	7	Schistosoma mansoni high voltage-activated calcium channel beta subunit CavB1 mRNA, complete cds

11861	acagtgaaac	1				no_annot
11862	tgtacgtcac	1				no_annot
11863	ccagtctggt	1				no_annot
11864	aacacacacc	1	TC13980	9	6	weakly similar to GP|16741131|gb|AAH16418.1||BC016418 expressed sequence AA960436 {Mus musculus}, partial (10%)
11864	aacacacacc	1	CD079010	20	2	
11865	tctaaccagc	1				no_annot
11866	tattctatcc	1				no_annot
11867	gggcctaccc	1				no_annot
11868	tatatgttct	1				no_annot
11869	tgacgcccca	1				no_annot
11870	tgactattta	1	TC17594	8	4	weakly similar to SP|P59517|SYN_BUCBP Asparaginyl-tRNA synthetase(Asparagine--tRNA ligase) (AsnRS). [subsp. Baizongia pistaciae] {Buchnera aphidicola}, partial (19%)
11871	tccgggattt	1	TC10884	15	1	weakly similar to SP|O95182|N4AM_HUMAN NADH-ubiquinone oxidoreductase subunit B14.5a(Complex I-B14.5a) (CI-B14.5a). [Human] {Homo sapiens}, partial (28%)
11872	cgcgtgtgtg	1				no_annot
11873	gagactcaaa	1				no_annot
11874	tatgttcaca	1				no_annot
11875	cacgatttcc	1				no_annot
11876	tgtcgttcta	1				no_annot
11877	atcgtaatac	1				no_annot
11878	cgcttatttt	1	TC10860	49	3	weakly similar to SP|Q9UGI0|TRAB_HUMAN TRABID protein. [Human] {Homo sapiens}, partial (17%)
11879	ggcgataacc	1	TC17088	63	1	weakly similar to GP|28853039|gb|AAO56108.1||AE016865 ABC transporter, ATP-binding/permease protein {Pseudomonas syringae pv. tomato str. DC3000}, partial (4%)
11880	ttttgaattc	1	TC8886	81	0	
11880	ttttgaattc	1	CD158303	3	1	
11881	aactcaacta	1				no_annot

11882	acaaatatac	1	C604280.1	95	0	
11883	attaggaaca	1				no_annot
11884	tctaatgaag	1				no_annot
11885	cactatatca	1				no_annot
11886	aagaagggga	1				no_annot
11887	gaagacaaat	1				no_annot
11888	catcacgcgt	1				no_annot
11889	tgagcccttg	1	CD118048	85	0	
11890	atagctgttg	1				no_annot
11891	gcaatagcaa	1				no_annot
11892	atacattgag	1				no_annot
11893	ctattgtgag	1				no_annot
11894	tgtagcgaag	1				no_annot
11895	atctctctag	1				no_annot
11896	cgtacgtcat	1				no_annot
11897	caacctgcta	1	TC13477	49	0	PIR|B54525|B54525 major female-specific polypeptide (frame 2) - fluke (Schistosoma mansoni) (fragment), partial (55%)
11898	catacgaatt	1	TC7891	79	0	
11898	catacgaatt	1	TC7892	86	0	similar to GP|15425738|dbj|BAB64346.1||AB069680 homeotic protein {Daphnia magna}, partial (4%)
11899	attttgtata	1	C604184.1	83	0	
11900	tgaacacaca	1				no_annot
11901	cgctgtcctt	1				no_annot
11902	tttgtttgtg	1				no_annot
11903	atcggacgag	1				no_annot
11904	ttggagattg	1				no_annot
11905	tagtgttggg	1				no_annot
11906	ttgatctaca	1	TC13697	38	2	weakly similar to GP|15418966|gb|AAK83461.1||AY039235 annexin 4 {Xenopus laevis}, partial (63%)
11907	gtcgatctca	1				no_annot

11908	ctactgttac	1				no_annot
11909	aacacacaaa	1				no_annot
11910	aatgactacg	1				no_annot
11911	gatgacagtc	1	TC10832	73	1	
11912	gtgaatggaa	1				no_annot
11913	ttgggtttat	1	CD076553	2	2	
11914	aacgcaacgg	1				no_annot
11915	tgtcgatggt	1				no_annot
11916	tgtttagtag	1				no_annot
11917	aaatcattca	1				no_annot
11918	gatcgataag	1				no_annot
11919	cataacactt	1				no_annot
11920	ttagcatacg	1				no_annot
11921	ggtaagttta	1	C201125.1	39	1	
11921	ggtaagttta	1	C608252.1	35	1	
11922	gttggtgccc	1				no_annot
11923	acagttgatg	1				no_annot
11924	tagatttcaa	1	C603853.1	72	1	
11925	actggcccac	1				no_annot
11926	gagaacaatt	1				no_annot
11927	ccctgtggag	1	C202220.1	19	1	
11927	ccctgtggag	1	C604744.1	44	1	
11928	tatgcacact	1				no_annot
11929	gcgaggacca	1				no_annot
11930	acgtagaaac	1				no_annot
11931	gtacgatgtg	1				no_annot
11932	caaatacctg	1				no_annot
11933	tttggcggtt	1				no_annot
11934	ttataacata	1				no_annot
11935	tccaataact	1				no_annot

11936	agaaataagt	1	TC16958	3	11	
11937	gtcggatgga	1				no_annot
11938	aagcatacga	1				no_annot
11939	attaatttgt	1				no_annot
11940	tgtgagttga	1				no_annot
11941	tacatagagc	1				no_annot
11942	caaaaaaagg	1				no_annot
11943	tacatagaga	1				no_annot
11944	cattaatcaa	1				no_annot
11945	ggtcccgtca	1				no_annot
11946	gtcctttgtg	1				no_annot
11947	agcaagttct	1	TC17087	87	0	similar to GP|15408877|dbj|BAB64268.1||AP004127 P0005H10.5 {Oryza sativa (japonica cultivar-group)}, partial (9%)
11948	tccttcatac	1	TC8580	2	6	similar to GP|13182761|gb|AAK14926.1|AF212241_1|AF212241 CDA02 {Homo sapiens}, partial (5%)
11949	aaaaagtttt	1	CD149513	88	0	
11949	aaaaagtttt	1	AI976406	59	0	
11950	tgtacacgtc	1				no_annot
11951	taagtatttt	1	TC18798	87	0	similar to GP|14335062|gb|AAK59795.1||AY037210 AT3g02110/F1C9_10 {Arabidopsis thaliana}, partial (4%)
11951	taagtatttt	1	CD118134	47	3	
11952	ctttttagtt	1	C201642.1	11	1	
11953	gacacaggct	1	C305104.1	93	0	
11954	taggaaatag	1				no_annot
11955	atatttactg	1				no_annot
11956	gccatcataa	1				no_annot
11957	ttagatttct	1	AF254148|Sma.785	96	0	PUR-alpha-like protein (PUR-alpha)
11958	ggtaagatgc	1				no_annot
11959	ctttaatata	1				no_annot
11960	ctatgagtgg	1				no_annot

11961	ctgatttatt	1				no_annot
11962	gtgaattaac	1				no_annot
11963	attcgttctt	1				no_annot
11964	atactgagta	1	TC11323	15	2	
11964	atactgagta	1	CD149278	75	0	
11965	tccttggcga	1				no_annot
11966	gaaagataat	1				no_annot
11967	ataactttac	1				no_annot
11968	ttatcaaaaa	1				no_annot
11969	taattaatta	1				no_annot
11970	aaaatgaaca	1	TC14773	0	8	weakly similar to PIR|C88197|C88197 protein ZK1127.10 [imported] - Caenorhabditis elegans, partial (62%)
11970	aaaatgaaca	1	CD190937	79	2	
11971	atattcacaa	1	TC17071	67	0	
11971	atattcacaa	1	TC17073	94	0	
11972	tctagaacaa	1				no_annot
11973	tgaagtacgg	1				no_annot
11974	ttgacatatt	1				no_annot
11975	tagacatcaa	1				no_annot
11976	ttttcaagta	1				no_annot
11977	tcgatctgtg	1				no_annot
11978	atgatggaca	1				no_annot
11979	tgtgtgcgta	1				no_annot
11980	atttccattg	1				no_annot
11981	cttggcggtc	1				no_annot
11982	tcagagtcct	1	TC13782	24	1	
11983	tctgacacgt	1				no_annot
11984	aaggttgttt	1				no_annot
11985	tatctctgct	1	AA233979	23	0	
11985	tatctctgct	1	CD066499	77	0	

11986	tactcgtcaa	1				no_annot
11987	tactcaggag	1				no_annot
11988	tattgcacaa	1				no_annot
11989	gacttagtct	1				no_annot
11990	caagtgagtg	1	CD126991	32	1	homologue to GP|12005330|gb|AAG44395.1|AF240460_1|AF240460 Nedd4-binding brain specific protein BEAN {Mus musculus}, partial (5%)
11991	tacccagtat	1				no_annot
11992	agtatgcaca	1	TC15015	59	0	similar to SP|P16436|HNT1_BOVIN Histidine triad nucleotide-binding protein 1 (Adenosine 5'-monophosphoramidase) (Protein kinase C inhibitor 1) (Protein kinase C- interacting protein 1) (PKCI-1) (17 kDa inhibitor of protein kinase C). [Rat] {Rattus norvegicus}, partial (37%)
11993	cacttttaca	1	TC13629	76	0	similar to GP|5578916|emb|CAB51359.1||AJ133756 jim zinc finger protein {Drosophila melanogaster}, partial (4%)
11993	cacttttaca	1	TC13630	96	0	similar to GP|27820070|gb|AAO25062.1||BT003302 GH11071p {Drosophila melanogaster}, partial (4%)
11993	cacttttaca	1	CD138948	15	1	similar to GP|7290925|gb|AAF46366.1||AE003443 CG10555-PA {Drosophila melanogaster}, partial (3%)
11993	cacttttaca	1	CD138948	15	0	similar to GP|7290925|gb|AAF46366.1||AE003443 CG10555-PA {Drosophila melanogaster}, partial (3%)
11994	tttgaaggat	1	U30266|Sma.16	12	1	ATPase inhibitor-like protein trans-spliced
11995	tactcaggaa	1				no_annot
11996	ggcaaggctg	1				no_annot
11997	aaggttgtta	1				no_annot
11998	catcaaaaaa	1				no_annot
11999	gaatacaagc	1	TC17383	76	0	weakly similar to GP|18135|emb|CAA35079.1||X17207 cgcr-1 product {Chlamydomonas reinhardtii}, partial (13%)
12000	actttcaatg	1	TC11094	94	0	
12001	tggtctgaga	1				no_annot
12002	tgcttaatac	1				no_annot
12003	ccattacctg	1				no_annot

12004	ttggtgtatg	1	TC16625	76	4	similar to GP|16182307|gb|AAL13472.1||AY058243 GH01077p {Drosophila melanogaster}, partial (62%)
12005	aagcatacca	1	TC17925	30	5	
12006	cgcagtaatc	1				no_annot
12007	tactttcgta	1				no_annot
12008	attaggtagg	1	TC6864	57	0	weakly similar to SP|P47179|DAN4_YEAST Cell wall protein DAN4 precursor. [Baker's yeast] {Saccharomyces cerevisiae}, partial (3%)
12008	attaggtagg	1	TC6880	15	6	similar to GP|17944974|gb|AAL48550.1||AY070928 RE03018p {Drosophila melanogaster}, partial (3%)
12008	attaggtagg	1	TC6897	52	0	
12008	attaggtagg	1	TC7009	37	1	
12008	attaggtagg	1	TC7036	87	0	similar to SP|Q12224|RLM1_YEAST Transcription factor RLM1. [Baker's yeast] {Saccharomyces cerevisiae}, partial (3%)
12008	attaggtagg	1	TC7084	64	0	similar to GP|25012496|gb|AAN71352.1||BT001597 RE29416p {Drosophila melanogaster}, partial (3%)
12008	attaggtagg	1	TC7127	85	1	homologue to GP|6478621|gb|AAF13926.1|AF195870_1|AF195870 brain tumor {Drosophila melanogaster}, partial (3%)
12008	attaggtagg	1	CD153977	29	1	
12009	tatcgaatgt	1				no_annot
12010	gtaaatatac	1				no_annot
12011	tgctgacgat	1				no_annot
12012	gagaaattta	1				no_annot
12013	acaagcgaaa	1				no_annot
12014	gtgtttatcc	1				no_annot
12015	cagctcgata	1				no_annot
12016	actaaccctc	1	C210784.1	79	1	
12017	aacctgtgtt	1				no_annot
12018	tacaaagtgt	1				no_annot
12019	tcagaatact	1	TC7153	26	0	
12020	gtagtggggg	1				no_annot
12021	aaggtgttga	1	TC14356	19	0	

12022	aaaatacttc	1	C207978.1	90	0	
12022	aaaatacttc	1	C607848.1	61	0	
12023	aaaatcgacc	1	C612541.1	38	0	
12024	tacccgaaat	1				no_annot
12025	aatgataact	1				no_annot
12026	atgcagaggt	1				no_annot
12027	caagtaatgc	1				no_annot
12028	acatatcgag	1	TC12629	34	1	weakly similar to GP|12597312|gb|AAG60033.1|AF302783_1|AF302783 tRNA-guanine transglycosylase {Homo sapiens}, partial (25%)
12029	agaagagaca	1	C201299.1	27	3	
12029	agaagagaca	1	C603032.1	24	3	
12030	tacatcttca	1	TC15337	81	0	
12031	tgtaaacaaa	1	C703879.1	23	2	
12032	ggctgagaga	1				no_annot
12033	tagtcggtgt	1				no_annot
12034	attttgaaat	1	TC9942	5	1	
12035	acaacataac	1				no_annot
12036	gcagacattg	1				no_annot
12037	ttggtgaagg	1				no_annot
12038	gagaaaatgt	1	TC16616	47	0	
12039	tcgtcatcaa	1				no_annot
12040	ttcaatcagt	1	TC9238	63	2	weakly similar to GP|17862672|gb|AAL39813.1||AY069668 LD44494p {Drosophila melanogaster}, partial (18%)
12040	ttcaatcagt	1	TC17449	37	2	homologue to GP|28395487|gb|AAO39081.1||AY191018 autophagy protein 4 {Dictyostelium discoideum}, partial (3%)
12041	cagttgacaa	1				no_annot
12042	aaactttctg	1	AI168870	86	0	
12043	cagtgttgat	1				no_annot
12044	taggataata	1				no_annot

12045	gacaatttcg	1				no_annot
12046	tttgaataaa	1	TC10657	98	0	similar to GP|9501238|emb|CAB99478.1||AJ271781 protein phosphatase 5 {Drosophila melanogaster}, partial (75%)
12047	agactggatc	1				no_annot
12048	ccaagttgtg	1				no_annot
12049	atcagattgc	1				no_annot
12050	cttggaatat	1	TC13469	7	4	
12051	gtaacatagg	1	TC18896	63	0	
12052	atcagattga	1	TC16842	92	0	similar to EGAD|126266|134665 chitinase {Emericella nidulans}, partial (4%)
12052	atcagattga	1	CD161952	50	0	
12053	caaaccacgt	1				no_annot
12054	ttttatgcta	1				no_annot
12055	cataaaagtg	1				no_annot
12056	tcaataaata	1				no_annot
12057	ttatggaaat	1	CD131678	12	2	similar to GP|10047307|dbj|BAB13470.1||AB036738 neural RNA-binding protein MSI-1 {Caenorhabditis elegans}, partial (17%)
12058	tgaatggtgc	1				no_annot
12059	ataagatggg	1				no_annot
12060	accactgatt	1	C610628.1	41	1	
12061	gttaaaaaaa	1				no_annot
12062	cttagtaggc	1				no_annot
12063	taaagaatgt	1	TC13264	47	5	weakly similar to PIR|JC5392|JC5392 zinc finger protein KF-1 precursor - human, partial (5%)
12063	taaagaatgt	1	BE505117	66	1	
12064	aagacattct	1				no_annot
12065	aatatcggtt	1	AI976911	30	2	
12066	taaatttgtc	1	AI977020	10	0	
12067	caaagatttt	1	TC10742	18	5	similar to SP|P53488|ARP2_CHICK Actin-like protein 2 (Actin-like protein ACTL). [Chicken] {Gallus gallus}, partial (92%)
12067	caaagatttt	1	CD080835	32	0	

12068	gaatacaacg	1				no_annot
12069	aatgataaat	1				no_annot
12070	tggtgagcag	1				no_annot
12071	gtaaggaaag	1				no_annot
12072	aacgctgtct	1				no_annot
12073	accactgatc	1				no_annot
12074	atggggctaa	1				no_annot
12075	ccataaaact	1	TC8322	84	0	similar to GP|20161512|dbj|BAB90435.1||AP003709 OSJNBb0006H05.12 {Oryza sativa (japonica cultivar-group)}, partial (17%)
12076	atcttcaagt	1				no_annot
12077	tttttctgct	1				no_annot
12078	tacaagaagc	1				no_annot
12079	tctttaacat	1				no_annot
12080	tacattatgt	1				no_annot
12081	tacaagaaga	1				no_annot
12082	ggggaaaaga	1				no_annot
12083	attaggtaca	1				no_annot
12084	gcaaatgctc	1				no_annot
12085	cagtaaatgc	1				no_annot
12086	tccaatggcc	1				no_annot
12087	agcaaaacac	1	CD156424	56	1	
12088	ggtatccatt	1				no_annot
12089	tctgacacag	1				no_annot
12090	aagttaactt	1				no_annot
12091	taagtcaagt	1				no_annot
12092	tgactgaggt	1				no_annot
12093	ctcctatatc	1				no_annot
12094	gctcgaatgg	1				no_annot
12095	tgtatcgttc	1				no_annot

12096	taatattgaa	1	TC17108	82	0	weakly similar to PIR|T50839|T50839 U4/U6 small nuclear ribonucleoprotein hPrp3 [imported] - human, partial (27%)
12097	gccaggatta	1				no_annot
12098	ttctgggtga	1				no_annot
12099	tttagtagca	1				no_annot
12100	attgagttcg	1				no_annot
12101	aatctgtact	1				no_annot
12102	caaccataaa	1	C301703.1	51	1	
12102	caaccataaa	1	C606787.1	70	1	
12103	atatctgttg	1	AI739723	86	0	
12104	gaaaattgaa	1				no_annot
12105	ataaggcaca	1				no_annot
12106	tctcaaatta	1	TC16973	90	0	similar to GP|18256123|gb|AAH21823.1||BC021823 WW domain binding protein 11 {Mus musculus}, partial (15%)
12107	accggttcat	1				no_annot
12108	ttgtggccca	1				no_annot
12109	atatggactt	1				no_annot
12110	ttttttgtt	1	CD084345	44	0	
12111	tgtcgttcgt	1				no_annot
12112	tatccaagga	1				no_annot
12113	gaatacaaac	1	CD197789	19	0	similar to GP|2150120|gb|AAB58746.1||AF001334 cardiac calsequestrin {Rattus norvegicus}, partial (7%)
12114	gttcatttct	1				no_annot
12115	ctatggaaac	1				no_annot
12116	tataacacgt	1				no_annot
12117	ttggatcgag	1	CD069377	71	0	
12118	gtaacaggag	1	C307050.1	67	0	
12119	gtctatctca	1	C300920.1	83	0	
12120	cataactctt	1				no_annot
12121	tattttcaac	1				no_annot

12122	atgatatgtc	1				no_annot
12123	cacaagaagc	1				no_annot
12124	tttggcaaat	1				no_annot
12125	gatacgccac	1				no_annot
12126	tttaaaattg	1				no_annot
12127	tattcacact	1				no_annot
12128	gtagtgggat	1				no_annot
12129	cagagatacg	1				no_annot
12130	aatcagttgg	1				no_annot
12131	aagcattcga	1				no_annot
12132	atcttgtgtg	1	C201302.1	75	1	
12132	atcttgtgtg	1	C603077.1	67	3	
12133	actaaataag	1				no_annot
12134	gcagtaatct	1				no_annot
12135	tgtgatgtgg	1				no_annot
12136	ataaggcaaa	1	TC16101	55	1	
12137	cattatctgt	1	C208923.1	21	3	
12137	cattatctgt	1	C603367.1	21	3	
12138	gcagtaatcg	1				no_annot
12139	tagaattggt	1				no_annot
12140	ttaaccaatg	1	AA566212	48	1	
12141	atacaagatt	1				no_annot
12142	tggtgcagaa	1				no_annot
12143	ccggtcaatg	1				no_annot
12144	aacggaaacg	1	C210829.1	49	1	
12144	aacggaaacg	1	C604281.1	38	2	
12145	acaatgattg	1	AF361883|Sma.92	56	11	High voltage-activated calcium channel Cav2A
12146	ttttccacca	1				no_annot
12147	tacacaatgc	1				no_annot
12148	gatgaagcca	1				no_annot

12149	cgcacgcaat	1				no_annot
12150	tacacaatga	1				no_annot
12151	gtcaaatgtc	1	C604307.1	57	2	
12152	cgcttcaaac	1				no_annot
12153	gcaagaatgg	1	TC18414	94	0	similar to GP|7292941|gb|AAF48332.1||AE003493 CG11071-PA {Drosophila melanogaster}, partial (3%)
12154	gtcaaatgta	1				no_annot
12155	ctggatcgag	1				no_annot
12156	acgttacact	1	TC16705	44	4	
12157	ataatggtga	1				no_annot
12158	atacaagatc	1				no_annot
12159	atcagattat	1	C206110.1	62	1	
12159	atcagattat	1	C608910.1	62	1	
12160	gttaaataga	1				no_annot
12161	cacatacatt	1				no_annot
12162	taatgcccct	1				no_annot
12163	ttactcctaa	1	CD130964	93	0	
12164	tacaagaaca	1				no_annot
12165	gatgtgcact	1	CD197375	46	0	
12165	gatgtgcact	1	CD161090	85	0	
12166	tcctgcgtta	1				no_annot
12167	aggattattt	1	TC16531	61	0	
12167	aggattattt	1	CD144615	44	0	
12168	tattcacaat	1	C201193.1	27	0	
12168	tattcacaat	1	C205607.1	48	0	
12168	tattcacaat	1	C603384.1	23	0	
12169	gttactactg	1				no_annot
12170	ggttgtgtct	1				no_annot
12171	aaaaacgtaa	1				no_annot
12172	aatgattaga	1	C314517.1	79	0	
12172	aatgattaga	1	C605909.1	79	0	

12173	cccttttta	1				no_annot
12174	gaaaacattt	1	CD064918	44	0	
12175	aaataccaaa	1				no_annot
12176	atcgcaggtt	1	TC10986	38	2	similar to SP|P08708|RS17_HUMAN 40S ribosomal protein S17. [Human] {Homo sapiens}, partial (78%)
12177	tatacagtaa	1				no_annot
12178	taacaacaaa	1				no_annot
12179	aatggacata	1				no_annot
12180	ttgaatataa	1				no_annot
12181	ttgctggtta	1	TC13864	61	2	similar to PIR|D88678|D88678 protein H06H21.3 [imported] - Caenorhabditis elegans, partial (59%)
12182	tattcacaaa	1	TC11772	47	0	
12183	atttacgtcg	1				no_annot
12184	tgcgattttt	1	TC18116	23	2	
12185	agcatccaaa	1				no_annot
12186	gcagtaatag	1				no_annot
12187	gatacacaaa	1				no_annot
12188	taagtcaaca	1	TC11140	2	6	homologue to GP|6692645|gb|AAF24788.1|U17009_20|U17009 NADH dehydrogenase subunit 3 {Phytophthora infestans}, partial (11%)
12189	tgcatcattg	1				no_annot
12190	gcggaattct	1	AF195529|Sma.951	52	1	14-3-3 epsilon
12191	tgactattcg	1				no_annot
12192	tatattcttt	1				no_annot
12193	ggcggtttgc	1				no_annot
12194	ccgaaaaagc	1				no_annot
12195	ttatcacgtg	1	TC11788	59	0	similar to GP|21647772|gb|AAM72982.1||AE012929 iron(III) ABC transporter, permease protein {Chlorobium tepidum TLS}, partial (6%)
12196	tgatacagca	1	TC7719	93	1	weakly similar to GP|16769502|gb|AAL28970.1||AY061422 LD35289p {Drosophila melanogaster}, partial (18%)
12197	ccttactctt	1				no_annot

12198	caagtgtgtg	1				no_annot
12199	agcaatgtac	1				no_annot
12200	gcaactactt	1				no_annot
12201	ggttctgggg	1	TC7048	3	9	weakly similar to GP|27497118|gb|AAO17319.1|AF465616_1|AF465616 death inducer with SAP domain DIS {Homo sapiens}, partial (6%)
12202	cttgatgcct	1				no_annot
12203	aactacagag	1				no_annot
12204	caattgagtt	1				no_annot
12205	caaagctatc	1	TC14202	55	1	
12206	tttactcaaa	1				no_annot
12207	aaaaagttgc	1				no_annot
12208	ctatcataat	1				no_annot
12209	taggatggtg	1	CD076993	76	0	
12210	tacaagaaac	1				no_annot
12211	ggggaaaaaa	1	TC12771	6	1	similar to GP|5869819|emb|CAB55576.1||AJ249395 NADH-ubiquinone oxidoreductase subunit 1 {Globodera pallida}, partial (9%)
12212	gagtaacggt	1				no_annot
12213	gaataattac	1				no_annot
12214	taggatggta	1				no_annot
12215	gcaactacta	1				no_annot
12216	actagattgc	1	CD075729	3	1	
12217	atgggttgtt	1	CD185103	27	3	similar to PIR|A35419|A35419 neutrophil protein - pig (fragment), partial (5%)
12218	tgtgtgcggt	1	TC16784	28	9	
12218	tgtgtgcggt	1	TC16784	28	5	
12219	tcaccgagta	1	TC13639	18	5	similar to GP|15487549|gb|AAB52440.3||U97193 Tropomodulin protein 1, isoform a {Caenorhabditis elegans}, partial (21%)
12220	gtaaatttat	1				no_annot
12221	ttatgccatt	1				no_annot
12222	aataaagatg	1	CD080187	96	0	

12223	aaacggtctt	1				no_annot
12224	tataatactt	1				no_annot
12225	tttgtttgct	1				no_annot
12226	tcaataggta	1				no_annot
12227	atcactacca	1				no_annot
12228	cattgttctc	1				no_annot
12229	tttatgacgg	1	C604564.1	76	0	
12230	taagtcaaag	1				no_annot
12231	gttacaagga	1				no_annot
12232	ctgaatataa	1				no_annot
12233	cttatgtctg	1				no_annot
12234	tttgtttgcg	1				no_annot
12235	ctggtacttt	1	TC14408	93	0	similar to SP|Q31125|KE4_MOUSE Histidine-rich membrane protein Ke4. [Mouse] {Mus musculus}, partial (32%)
12236	actgatattt	1				no_annot
12237	cttggattct	1				no_annot
12238	tgactattag	1				no_annot
12239	tattatctac	1				no_annot
12240	tacaaacttg	1				no_annot
12241	tttatatatt	1				no_annot
12242	catattcttt	1				no_annot
12243	cttactcaag	1	TC13827	66	0	similar to GP|4107017|dbj|BAA36294.1||AB001773 PEM-6 {Ciona savignyi}, partial (29%)
12243	cttactcaag	1	TC13828	16	1	homologue to GP|15529624|gb|AAL01375.1|AF406557_1|AF406557 PTPRE {Homo sapiens}, partial (8%)
12244	cttggattca	1	C203232.1	10	3	
12245	ctgcttcact	1	C301445.1	13	1	
12245	ctgcttcact	1	C714871.1	13	1	
12246	actatttggg	1				no_annot
12247	tggatgcaga	1				no_annot

12248	aaccttggtg	1				no_annot
12249	tccactagaa	1	CD189025	73	0	weakly similar to GP|3309254|gb|AAC26050.1||AF073921 regulator of G-protein signaling 6 variant form {Homo sapiens}, partial (16%)
12250	ttgatttaca	1	C610111.1	1	0	
12251	aagttgttgc	1	TC13700	21	3	similar to SP|O60832|DKC1_HUMAN Dyskerin (Nucleolar protein NAP57) (CBF5 homolog). [Human] {Homo sapiens}, partial (60%)
12251	aagttgttgc	1	TC13701	88	0	weakly similar to SP|O43100|CBF5_EMENI Centromere/microtubule binding protein CBF5 (Centromere-binding factor 5) (Nucleolar protein CBF5). [Aspergillus nidulans] {Emericella nidulans}, partial (13%)
12252	tacgtgatct	1				no_annot
12253	aacagttcat	1				no_annot
12254	acatacgtag	1				no_annot
12255	tttcccggtc	1				no_annot
12256	attgttgaaa	1				no_annot
12257	ggacattaga	1	C207919.1	2	2	
12257	ggacattaga	1	C610101.1	2	2	
12258	gcacaacaat	1				no_annot
12259	agctcgattc	1	TC8592	55	0	
12260	tcctccggtc	1				no_annot
12261	gatgacagat	1				no_annot
12262	ctaaagaagc	1	TC18494	67	0	weakly similar to PIR|JC5573|JC5573 copper-transporting ATPaseP-type - Caenorhabditis elegans, partial (10%)
12263	ggggtttgtg	1	C610724.1	59	1	
12264	gtgatgaagt	1	TC11041	19	2	
12265	ttgaatttgt	1				no_annot
12266	aagtcagttg	1				no_annot
12267	gcaaaataca	1				no_annot
12268	ctacctgatg	1				no_annot
12269	acatagttgt	1				no_annot

12270	gatggaggtg	1	C201491.1	17	3	
12270	gatggaggtg	1	C602991.1	16	3	
12271	gaatgtattt	1				no_annot
12272	tgacttggtt	1				no_annot
12273	atcctgatca	1				no_annot
12274	agaatagttt	1	C205759.1	54	0	
12274	agaatagttt	1	C603656.1	51	0	
12275	ttacactcgt	1				no_annot
12276	cacacaatcg	1				no_annot
12277	tgcttcggac	1				no_annot
12278	atcgcaaagt	1				no_annot
12279	tgtatgatgg	1				no_annot
12280	ttattgattg	1	CD195845	85	0	
12281	atattatgca	1	CD146090	15	3	
12282	tttcttatag	1				no_annot
12283	aatgattaat	1				no_annot
12284	atgataagac	1				no_annot
12285	gtgttgccac	1				no_annot
12286	cttatatata	1				no_annot
12287	cgacacatac	1	TC13907	88	1	
12288	tcgaggagta	1				no_annot
12289	cttaacaatt	1				no_annot
12290	tattggcaat	1				no_annot
12291	cacaaggggc	1				no_annot
12292	ggggaatagt	1				no_annot
12293	atcatcatta	1	CD113644	3	0	
12293	atcatcatta	1	CD153309	48	1	
12293	atcatcatta	1	CD145663	68	0	weakly similar to SP|Q9BQK8|LPN3_HUMAN Lipin 3 (Lipin 3-like) (Fragments). [Human] {Homo sapiens}, partial (4%)

12294	taggtcccat	1	CD124270	62	0	similar to GP|2564952|gb|AAB82009.1||AF030001 lysophatidic acid acyl transferase-alpha {Mus musculus}, partial (5%)
12295	cacaatgatt	1				no_annot
12296	atcgtgtgaa	1	CD080802	18	1	
12297	taagacatcc	1				no_annot
12298	ccaccaatgg	1				no_annot
12299	ccctttatca	1				no_annot
12300	aaaattgagt	1	TC11846	57	2	
12301	ggccctatgg	1	TC13521	59	4	similar to GP|7300349|gb|AAF55508.1||AE003721 CG7187-PA {Drosophila melanogaster}, partial (5%)
12302	atgctcccat	1	C303849.1	5	1	
12303	catacacttg	1	TC16261	58	2	
12304	ataatcagtg	1				no_annot
12305	tggaaagcca	1				no_annot
12306	agtgaaatat	1				no_annot
12307	tttcggtttc	1	CD116811	42	1	
12308	cacaatgata	1				no_annot
12309	aagttccttg	1				no_annot
12310	ctaatacata	1				no_annot
12311	aacgaaccct	1				no_annot
12312	taaagcaaat	1				no_annot
12313	tgattttcgt	1				no_annot
12314	gtggaccacg	1	U86674|Sma.752	29	8	Calponin homolog
12314	gtggaccacg	1	U86674|Sma.752	29	7	Calponin homolog
12315	gcaaaataac	1				no_annot
12316	agcttaagtc	1				no_annot
12317	atctacatat	1	TC11714	71	0	
12318	gattcgttga	1	TC10652	47	0	similar to GP|20336352|gb|AAM18191.1||AY092757 SNAP25 fusion protein {Loligo pealei}, partial (80%)
12319	atgcataaga	1				no_annot

12320	gtgaccattg	1	CD165257	61	2	homologue to GP|17224967|gb|AAL37189.1|AF321090_1|AF321090 cytochrome o ubiquinol oxidase B {Pseudomonas putida}, partial (9%)
12321	tgttgcactg	1	TC19660	16	2	
12322	tggcgagaca	1				no_annot
12323	gaatcagcag	1				no_annot
12324	gctcgattgg	1				no_annot
12325	tcacttatag	1				no_annot
12326	cgcaggtgat	1				no_annot
12327	agataagtac	1	TC12620	18	2	
12328	taaagcaaaa	1				no_annot
12329	tccgtacaca	1				no_annot
12330	tttgaacggt	1				no_annot
12331	ggctgtatac	1	TC19541	16	1	
12332	atctacataa	1	C603933.1	59	2	
12333	ggaatttcgc	1				no_annot
12334	ggcaaggcca	1				no_annot
12335	atattatgaa	1				no_annot
12336	actatttgca	1				no_annot
12337	atacatattg	1	TC9287	54	1	
12338	tcgagaatgt	1				no_annot
12339	tcggtctatc	1				no_annot
12340	tccatcgata	1				no_annot
12341	tcgcatttcc	1				no_annot
12342	ttttgcaaca	1				no_annot
12343	tattgtacat	1	TC14904	82	0	
12344	caaacaagac	1	TC7885	25	0	
12345	atactattac	1				no_annot
12346	aattgttttc	1				no_annot
12347	taattaataa	1	CD113467	52	0	
12348	tcgatctgat	1				no_annot

12349	agactaagta	1				no_annot
12350	taattcaagc	1				no_annot
12351	tagatgagtt	1	C200428.1	75	0	
12352	aatctccagt	1				no_annot
12353	aatacaaata	1	TC15476	55	2	
12354	gtgaagtttg	1				no_annot
12355	caagacatcc	1				no_annot
12356	attagtgaag	1				no_annot
12357	tttggttatc	1				no_annot
12358	ttttgtaatt	1				no_annot
12359	tattgtacaa	1				no_annot
12360	cttccaatct	1	C210656.1	18	4	
12360	cttccaatct	1	C600635.1	18	4	
12361	tttcggatgc	1				no_annot
12362	tgttccttga	1				no_annot
12363	tccaggcccc	1				no_annot
12364	aagaaccaat	1	TC18560	52	0	similar to GP|7293625|gb|AAF48997.1||AE003512 CG14223-PA {Drosophila melanogaster}, partial (3%)
12365	tttcacgaac	1				no_annot
12366	tgtataactg	1				no_annot
12367	tagctcgaaa	1				no_annot
12368	tttgtcattg	1				no_annot
12369	gtaatcgttt	1				no_annot
12370	atgagaaatg	1	TC16864	70	2	similar to GP|4886558|emb|CAB43352.1||AJ005937 lamin {Tealia sp.}, partial (7%)
12371	ctcagtctct	1	C206910.1	6	2	
12371	ctcagtctct	1	C604570.1	6	2	
12372	tacaccaacc	1	TC8643	89	1	
12373	ccctaacttg	1				no_annot
12374	tacttgtttt	1				no_annot
12375	ccctcaatca	1				no_annot

12376	aatcaaacat	1	C202144.1	53	2	
12376	aatcaaacat	1	C607915.1	53	2	
12377	aaatctggac	1	C202688.1	86	0	
12377	aaatctggac	1	C603140.1	90	0	
12378	tccagtttat	1	CD084139	78	1	
12379	tggatatgcc	1				no_annot
12380	acaggaagta	1				no_annot
12381	ttttgcaaaa	1				no_annot
12382	gaagatggca	1	C208072.1	45	1	
12382	gaagatggca	1	C607063.1	45	1	
12383	gtcaattctc	1	TC12773	3	2	weakly similar to GP|15010620|gb|AAK73969.1||AY045611 AT4g08960/T3H13_2 {Arabidopsis thaliana}, partial (15%)
12384	gtgaagatgt	1				no_annot
12385	aaacctgtac	1				no_annot
12386	cttgaacggc	1				no_annot
12387	caattaataa	1				no_annot
12388	ttgttgaaat	1	TC19304	89	0	
12389	gttgtctagc	1				no_annot
12390	ctggtgtacg	1				no_annot
12391	gagaagagaa	1	C603402.1	30	4	
12392	aagctaaatg	1	TC13836	80	1	
12393	cattgtacaa	1				no_annot
12394	gatccgactc	1	TC11413	74	1	similar to GP|28916483|gb|AAO59410.1||AY226980 nucleoside diphosphate kinase {Schistosoma japonicum}, partial (95%)
12395	gttacttctt	1				no_annot
12396	tgatctgtaa	1				no_annot
12397	gactaaacgt	1				no_annot
12398	ctgaaacagt	1				no_annot
12399	acgtgaacca	1	C210021.1	72	5	

12400	caacgtgact	1				no_annot
12401	gttttcaaga	1	TC13507	93	0	homologue to GP|3063620|gb|AAC14119.1||AF056330 AUT1 {Schistosoma mansoni}, partial (16%)
12401	gttttcaaga	1	CD077268	32	4	
12402	atacggaatg	1				no_annot
12403	gagattgccg	1				no_annot
12404	cttgtcattg	1				no_annot
12405	aatagagttg	1				no_annot
12406	gtgtgatcta	1	TC11920	87	0	similar to SP|P27815|CN4A_HUMAN cAMP-specific 3',5'-cyclic phosphodiesterase 4A(DPDE2) (PDE46). [Human] {Homo sapiens}, partial (31%)
12407	gaacccataa	1				no_annot
12408	cgagatagat	1				no_annot
12409	ttaaggatgc	1	C211342.1	4	5	
12410	aaaaatgtgt	1	TC15969	7	2	
12410	aaaaatgtgt	1	CD118325	57	0	
12411	cagagtcctt	1				no_annot
12412	tttgaacgcc	1				no_annot
12413	cacttgttta	1				no_annot
12414	gtgtcgtggt	1	BF936514	9	0	homologue to GP|5597017|gb|AAC24982.2||AF025672 reverse transcriptase {synthetic construct}, partial (8%)
12415	acgaatgttg	1				no_annot
12416	tgaatctgtg	1				no_annot
12417	tctgagcaac	1				no_annot
12418	agttaaatga	1				no_annot
12419	atgcgcttct	1				no_annot
12420	ttaaagtagt	1				no_annot
12421	cacattttct	1				no_annot
12422	tattgttcgt	1				no_annot
12423	tcacagggcg	1	CD073136	57	0	
12424	gctcggagag	1				no_annot
12425	agcagaaggg	1				no_annot

12426	tcctggatcg	1				no_annot
12427	gagagcatct	1				no_annot
12428	atacacttgt	1				no_annot
12429	aaaacggcaa	1				no_annot
12430	aaggagcata	1	C303509.1	34	1	
12431	tacattttaa	1	C606239.1	49	2	
12432	ttttatgcaa	1				no_annot
12433	aatgtcgatg	1				no_annot
12434	cctgttctcc	1				no_annot
12435	tcctcgaggt	1				no_annot
12436	ttaattttgt	1	C301158.1	39	0	
12436	ttaattttgt	1	C707215.1	39	0	
12437	aatgtcgatc	1				no_annot
12438	tatttggtaa	1				no_annot
12439	ttcaaagcta	1				no_annot
12440	tacagtgttt	1				no_annot
12441	taatttaata	1	CD086196	53	0	
12442	gagcatcatt	1				no_annot
12443	aatattggta	1				no_annot
12444	catcctaccg	1	TC17249	30	1	
12444	catcctaccg	1	TC17250	75	0	weakly similar to GP|17862114|gb|AAL39534.1||AY069389 LD09790p {Drosophila melanogaster}, partial (41%)
12445	gttgggaatc	1	CD125636	56	0	similar to SP|O00571|DDX3_HUMAN DEAD-box protein 3 (Helicase-like protein 2) (HLP2) (DEAD-box, X isoform). [Human] {Homo sapiens}, partial (7%)
12446	taattcaaat	1	C306518.1	18	1	
12447	tgagtaatta	1				no_annot
12448	attttactat	1	TC9151	13	4	
12449	tgacattttc	1				no_annot
12450	gtttactttt	1				no_annot
12451	tggactgtga	1				no_annot

12452	aaggtcctat	1	TC14479	39	0	weakly similar to OMNI|WD1008 aminopeptidase P {Wolbachia sp.}, partial (12%)
12453	ttctttctct	1	TC13637	75	1	weakly similar to GP|20151533|gb|AAM11126.1||AY094773 GM14501p {Drosophila melanogaster}, partial (73%)
12454	agtgttgcgt	1				no_annot
12455	caagaagagg	1	C314037.1	68	0	
12455	caagaagagg	1	C609916.1	75	1	
12456	gtgattgata	1				no_annot
12457	tttggtaact	1				no_annot
12458	aatccgcctc	1				no_annot
12459	ctaacaatac	1				no_annot
12460	ttctgctgtt	1				no_annot
12461	aatatcggag	1				no_annot
12462	aaccatatgc	1	CD159400	75	1	
12463	tacacattca	1				no_annot
12464	tgctatatgt	1				no_annot
12465	aaccatatga	1				no_annot
12466	ggaccaccac	1	C201297.1	22	2	
12466	ggaccaccac	1	C317901.1	96	0	
12466	ggaccaccac	1	C608663.1	18	3	
12467	tttctacagc	1				no_annot
12468	ccggtgtgtc	1				no_annot
12469	cactgccact	1				no_annot
12470	gtgaagatcc	1				no_annot
12471	acttatattg	1				no_annot
12472	atctcatatt	1	CD082461	65	0	
12473	tcaagcattt	1				no_annot
12474	caagaggcct	1				no_annot
12475	tagatctatt	1				no_annot
12476	tgttccttac	1				no_annot
12477	caggataaac	1				no_annot

12478	ttgatgattc	1	C201392.1	16	2	
12479	atcacttcat	1				no_annot
12480	aaacatagga	1				no_annot
12481	cgaatctgta	1				no_annot
12482	aggatacatc	1				no_annot
12483	atttttgaat	1	BG932497	18	2	
12484	tcaccgagga	1				no_annot
12485	ttacagcacc	1	CD113114	79	0	weakly similar to GP|16769552|gb|AAL28995.1||AY061447 LD38375p {Drosophila melanogaster}, partial (5%)
12486	gatgtttcaa	1	C209823.1	25	3	
12487	acataaacca	1				no_annot
12488	atcacttcag	1				no_annot
12489	tttaaaatca	1	CD080118	23	3	weakly similar to SP|O94111|DYL1_EMENI Dynein light chain, cytoplasmic (8 kDa cytoplasmic dynein light chain). [Aspergillus nidulans] {Emericella nidulans}, partial (56%)
12490	aaaccatcat	1	CD180843	0	1	
12491	cttcaccata	1				no_annot
12492	gcacgtgaag	1				no_annot
12493	ggagaaatag	1	TC13988	51	1	weakly similar to GP|17945259|gb|AAL48687.1||AY071065 RE14259p {Drosophila melanogaster}, partial (66%)
12494	gtagtccaca	1				no_annot
12495	gctaaaagaa	1				no_annot
12496	tgcgttcgtt	1				no_annot
12497	atttttgaaa	1				no_annot
12498	gttgtttata	1	TC19615	11	2	
12499	gtcaaatgct	1				no_annot
12500	ttctagcaag	1				no_annot
12501	gatggtgcta	1				no_annot
12502	aaaacagaac	1				no_annot
12503	ttaaaccata	1	C607631.1	39	1	

12504	agtctgtata	1				no_annot
12505	tggtaaattt	1				no_annot
12506	tacaaactga	1	M99494|Sma.23	59	10	200 kDa surface protein
12507	atctacttca	1				no_annot
12508	tgtcaatatg	1				no_annot
12509	tagagacgcc	1				no_annot
12510	cgtattgttg	1				no_annot
12511	ttcaacagtt	1				no_annot
12512	taaactacaa	1				no_annot
12513	taactgatct	1	TC8630	95	0	weakly similar to GP|6980000|gb|AAF34689.1|AF222864_1|AF222864 Dspt5 {Drosophila melanogaster}, partial (4%)
12514	tacatacaag	1				no_annot
12515	gcattattgt	1	TC15532	49	4	similar to SP|O00743|PPP6_HUMAN Serine/threonine protein phosphatase 6(PP6). [Human] {Homo sapiens}, partial (84%)
12515	gcattattgt	1	CD080096	49	2	similar to GP|5759301|gb|AAD51079.1|AF176121_1|AF176121 protein phosphatase 6 catalytic subunit {Dictyostelium discoideum}, partial (51%)
12516	ccaagcattt	1				no_annot
12517	agtagataag	1				no_annot
12518	agactgcata	1				no_annot
12519	acatttgaaa	1	CD144928	24	1	
12520	gactttgaag	1				no_annot
12521	ttttatcctc	1				no_annot
12522	aaaagaagta	1	AI111123	78	0	
12523	ccttcgggac	1				no_annot
12524	ctccgcttta	1	CD083525	72	0	weakly similar to GP|18447513|gb|AAL68318.1||AY075510 RE62710p {Drosophila melanogaster}, partial (24%)
12525	aaggctgatt	1				no_annot
12526	aacaaatatt	1				no_annot
12527	tggagaaagc	1				no_annot

12528	ccattttgct	1				no_annot
12529	aactgataga	1				no_annot
12530	tttctgccac	1				no_annot
12531	cctccggtac	1				no_annot
12532	cgtgctttga	1	TC15714	14	1	
12532	cgtgctttga	1	CD123546	29	0	
12533	cctagtctca	1				no_annot
12534	gttgcgacga	1				no_annot
12535	aaaatccaaa	1	C209980.1	89	0	
12536	atggtaaaga	1	TC12007	60	1	similar to PIR|D71616|D71616 ribosome releasing factor (OO, TP) PFB0390w [imported] - malaria parasite (Plasmodium falciparum), partial (4%)
12536	atggtaaaga	1	CD144530	59	0	
12537	gaacaaacat	1				no_annot
12538	tatattctct	1	TC17551	83	0	similar to SP|P13716|HEM2_HUMAN Delta-aminolevulinic acid dehydratase(Porphobilinogen synthase) (ALADH). [Human] {Homo sapiens}, partial (40%)
12539	atatgcgcca	1				no_annot
12540	acgccaacaa	1				no_annot
12541	agtgttgcct	1				no_annot
12542	tgctatatct	1	C200996.1	18	1	
12542	tgctatatct	1	C608279.1	18	1	
12543	agatcattga	1				no_annot
12544	gatcttggtt	1	TC15073	90	0	weakly similar to EGAD|87676|95456 cut9 protein {Schizosaccharomyces pombe}, partial (4%)
12545	tgctcaaaaa	1				no_annot
12546	gcgtcagccc	1				no_annot
12547	aaaactatgc	1	TC17124	66	1	weakly similar to GP|5052652|gb|AAD38656.1|AF145681_1|AF145681 BcDNA.LD23181 {Drosophila melanogaster}, partial (9%)
12548	taagttaact	1	AI976911	85	0	
12549	gaacaaacaa	1				no_annot

12550	taattattat	1				no_annot
12551	atacccgcgt	1				no_annot
12552	ttaatgccca	1				no_annot
12553	aaatatcaaa	1	Y10166|Sma.734	33	2	Cercarial antigen, partial
12554	ggtgcgttta	1				no_annot
12555	agattacagg	1				no_annot
12556	tgaatcagca	1				no_annot
12557	tatgtgctgg	1	TC16843	81	1	similar to GP|25989211|gb|AAL31707.1||AY061936 prophenoloxidase activating factor 3 {Bombyx mori}, partial (10%)
12557	tatgtgctgg	1	CD180703	68	1	similar to GP|21645360|gb|AAM70960.1||AE003809 CG30087-PA {Drosophila melanogaster}, partial (8%)
12558	atgactgtga	1				no_annot
12559	gcaagtggtc	1				no_annot
12560	tgcgcacgtg	1				no_annot
12561	agatttttcg	1	C209730.1	36	2	
12561	agatttttcg	1	C607916.1	39	2	
12562	gttgtgtgca	1				no_annot
12563	ccgaaacgtg	1	TC15587	47	2	
12564	taagacttac	1				no_annot
12565	ccgctcgtta	1	CD179333	81	0	
12566	gagagagaga	1	TC7805	9	2	similar to GP|458203|gb|AAA17551.1||U03627 beta-arrestin 2 {Rattus norvegicus}, partial (13%)
12567	attattgttg	1	C314675.1	16	1	
12567	attattgttg	1	C715995.1	16	1	
12568	tgtatccttc	1				no_annot
12569	tcactacact	1				no_annot
12570	gtgaatgtta	1				no_annot
12571	ttatgccacc	1				no_annot
12572	gtcaggctgc	1	C208885.1	80	0	
12572	gtcaggctgc	1	C603365.1	79	0	
12573	gccaggttgt	1				no_annot

12574	cctcagtgtt	1				no_annot
12575	cgttgctctt	1				no_annot
12576	atagattgga	1				no_annot
12577	gtgctgcaaa	1				no_annot
12578	aggtgactga	1	TC16559	20	4	similar to GP|4966317|gb|AAA88778.2||U46856 vitellogenin {Anolis pulchellus}, partial (7%)
12579	accggattta	1				no_annot
12580	tttgtctttg	1	C602652.1	4	0	
12581	gaggaagtgg	1				no_annot
12582	aaccatatac	1	CD088376	8	1	
12583	gaaagagtta	1				no_annot
12584	ctaaccaaac	1				no_annot
12585	tgtaacactt	1	C208488.1	75	0	
12585	tgtaacactt	1	C608618.1	75	0	
12586	ctttacccaa	1				no_annot
12587	tgtgttcaca	1				no_annot
12588	tagtgcttga	1				no_annot
12589	actatggctt	1				no_annot
12590	gagagcttgg	1	TC7552	27	2	weakly similar to GP|6759903|gb|AAF28099.1||AF137273 alpha 1 (V) collagen {Gallus gallus}, partial (20%)
12591	tagcccatag	1				no_annot
12592	acttacttgc	1				no_annot
12593	gtttacatca	1	CD063855	84	0	
12594	aagtttgtat	1	TC7551	26	4	
12595	aagtcgaaga	1	C206346.1	13	1	
12595	aagtcgaaga	1	C607401.1	13	1	
12596	cgctcaaaaa	1				no_annot
12597	agtttgtcat	1				no_annot
12598	gttgaagaag	1	C200742.1	83	4	
12599	ggtttggagc	1				no_annot
12600	cgctatatcg	1	TC10013	39	3	

12601	aaggactcga	1				no_annot
12602	ttcggttgtt	1				no_annot
12603	aagtttgtag	1	CD195720	42	0	
12604	gtgaagttgt	1				no_annot
12605	atttatgtaa	1				no_annot
12606	gacattgtta	1	TC9513	39	1	
12607	tatgaatatc	1	TC11975	94	0	
12608	tatgaatata	1	TC14343	0	2	
12609	agaatgaaga	1				no_annot
12610	gtaagccaat	1				no_annot
12611	tagatgaggt	1				no_annot
12612	actacttccc	1				no_annot
12613	gattctgtct	1				no_annot
12614	taaacttcgg	1				no_annot
12615	tatgttagtc	1				no_annot
12616	ggagacaaag	1				no_annot
12617	ctctggctgg	1	CD063808	31	2	homologue to GP|3063620|gb|AAC14119.1||AF056330 AUT1 {Schistosoma mansoni}, partial (23%)
12617	ctctggctgg	1	CD063808	31	0	homologue to GP|3063620|gb|AAC14119.1||AF056330 AUT1 {Schistosoma mansoni}, partial (23%)
12618	tatacactcc	1				no_annot
12619	ctgttgtact	1	CD093269	45	0	weakly similar to GP|5305395|gb|AAD41630.1|AF072331_1|AF072331 dynein light chain 5 {Schistosoma japonicum}, partial (62%)
12620	gttttgagaa	1				no_annot
12621	tattaaaatg	1				no_annot
12622	atgtaaattg	1	TC8293	80	0	
12623	gggagttggt	1	TC7875	2	1	
12624	attcaatata	1				no_annot
12625	ggtactatat	1	TC17081	10	0	weakly similar to SP|O17528|SEL9_CAEEL Suppressor/enhancer of lin-12 protein 9 precursor. {Caenorhabditis elegans}, partial (74%)

12626	ataatagcac	1				no_annot
12627	tgcgaacacc	1				no_annot
12628	tagatattgg	1	TC9829	47	0	
12629	ggtgacagtc	1				no_annot
12630	aaagtcaccg	1	CD073199	52	0	
12631	aaacgatact	1	CD163199	66	1	
12632	tttggtggct	1				no_annot
12633	ttccgtattg	1				no_annot
12634	ggcgttgatt	1				no_annot
12635	tacaaactaa	1				no_annot
12636	tgatggtcaa	1				no_annot
12637	cttttcattt	1	TC11464	2	1	homologue to PIR|T51146|T51146 ring-box protein 1 [imported] - human, partial (83%)
12637	cttttcattt	1	TC11464	2	0	homologue to PIR|T51146|T51146 ring-box protein 1 [imported] - human, partial (83%)
12637	cttttcattt	1	CD201242	31	3	
12637	cttttcattt	1	CD131284	74	0	homologue to PIR|T51146|T51146 ring-box protein 1 [imported] - human, partial (78%)
12638	tcgaggagct	1				no_annot
12639	ctaccccagt	1				no_annot
12640	tggctgaata	1				no_annot
12641	gtattactag	1	TC19654	70	1	weakly similar to GP|18044822|gb|AAH20002.1||BC020002 cDNA sequence BC020002 {Mus musculus}, partial (3%)
12642	aaacgataca	1				no_annot
12643	tttatataaa	1	TC12266	64	2	
12643	tttatataaa	1	TC13662	80	0	weakly similar to GP|2828147|gb|AAC00005.1||AF042384 BC-2 protein {Homo sapiens}, partial (60%)
12644	aaattggagg	1				no_annot
12645	aagactcgta	1	TC8492	79	1	weakly similar to GP|4567179|gb|AAD23607.1|AC007228_2|AC007228 BC37295_1 {Homo sapiens}, partial (18%)
12646	aaagaggtac	1	C201118.1	52	1	

12646	aaagaggtac	1	C604209.1	50	1	
12647	aaagcgatgc	1	TC12438	8	0	
12648	attttgcgcc	1	TC15742	69	1	
12649	taaagtaact	1				no_annot
12650	atcatcatcc	1				no_annot
12651	gaagagattt	1	CD170552	75	1	
12652	tacttgttga	1	TC13749	7	4	homologue to SP|Q95WA2|TCTP_SCHMA Translationally controlled tumor protein homolog (TCTP) (Histamine- releasing factor) (Fragment). [Blood fluke] {Schistosoma mansoni}, partial (84%)
12653	acgttggata	1				no_annot
12654	atcatcatca	1	TC15029	52	4	
12654	atcatcatca	1	TC16843	1	11	similar to GP|25989211|gb|AAL31707.1||AY061936 prophenoloxidase activating factor 3 {Bombyx mori}, partial (10%)
12654	atcatcatca	1	TC16843	1	10	similar to GP|25989211|gb|AAL31707.1||AY061936 prophenoloxidase activating factor 3 {Bombyx mori}, partial (10%)
12654	atcatcatca	1	CD060781	57	0	
12654	atcatcatca	1	CD116866	71	0	
12654	atcatcatca	1	CD197265	43	1	weakly similar to GP|20905799|gb|AAM31026.1||AE013365 Chemotaxis protein CheW {Methanosarcina mazei Goe1}, partial (15%)
12655	cccgaccaac	1				no_annot
12656	tttcggttct	1				no_annot
12657	gaggttatgc	1				no_annot
12658	gtatttgaat	1				no_annot
12659	gatcttaagg	1				no_annot
12660	tacaatgaaa	1	TC15817	78	0	
12661	taactgttgt	1	C201489.1	47	2	
12662	atacttggga	1				no_annot
12663	agatcattat	1	C201145.1	75	2	
12663	agatcattat	1	C605497.1	74	2	

12664	gttgcgacag	1				no_annot
12665	caacttgttt	1	TC14849	8	3	
12666	tggactcttg	1				no_annot
12667	tgaagcaatt	1				no_annot
12668	tgcgaacaag	1				no_annot
12669	tcccccgtac	1				no_annot
12670	cagatgaggg	1				no_annot
12671	gtatttgaaa	1				no_annot
12672	cctacgtttc	1				no_annot
12673	gacacagttg	1				no_annot
12674	gaaatttatt	1				no_annot
12675	gcgcagttca	1				no_annot
12676	taactgttgc	1	CD186719	31	4	similar to SP|Q02218|ODO1_HUMAN 2-oxoglutarate dehydrogenase E1 component, mitochondrial precursor(Alpha-ketoglutarate dehydrogenase). [Human] {Homo sapiens}, partial (6%)
12677	cacaggttaa	1				no_annot
12678	tagccagaga	1	CD080835	8	1	
12679	atcagttaaa	1	TC8366	22	8	similar to GP|157310|gb|AAA28494.1||M37083 E74B protein {Drosophila melanogaster}, partial (4%)
12680	ctcatctcga	1	TC12859	65	0	
12681	tagtcctggt	1	C207915.1	87	0	
12681	tagtcctggt	1	C610459.1	78	0	
12682	aaaactataa	1				no_annot
12683	cgatggtcaa	1				no_annot
12684	tttagctttg	1				no_annot
12685	ataaaatatc	1	TC10872	54	0	weakly similar to GP|16768340|gb|AAL28389.1||AY060841 GM02110p {Drosophila melanogaster}, partial (27%)
12686	gtgtgatcga	1	C201510.1	34	2	
12686	gtgtgatcga	1	C607958.1	34	2	
12687	ataaaatata	1				no_annot
12688	tgtcaaaaaa	1				no_annot

12689	ttaactggtt	1				no_annot
12690	tatgtgctag	1				no_annot
12691	cagtcgctac	1	TC13662	63	1	weakly similar to GP|2828147|gb|AAC00005.1||AF042384 BC-2 protein {Homo sapiens}, partial (60%)
12692	agtgaccgtt	1	CD166959	87	0	
12693	actgttggag	1				no_annot
12694	tcttgagcca	1				no_annot
12695	ttgcagacta	1				no_annot
12696	atcatcataa	1	CD197516	69	2	
12697	gtaactccgc	1				no_annot
12698	tctaacagta	1	C318335.1	67	0	
12699	tccccgttga	1	TC6882	64	0	weakly similar to GP|23477189|emb|CAD22046.1||AJ428954 tissue factor pathway inhibitor-2 {Rattus norvegicus}, partial (23%)
12700	tacgcagtgg	1	TC7460	6	6	
12701	atagattgat	1				no_annot
12702	gttttctact	1				no_annot
12703	tcgttattgt	1	TC17755	69	0	weakly similar to GP|13435996|gb|AAH04829.1||BC004829 synaptogyrin 2 {Mus musculus}, partial (26%)
12703	tcgttattgt	1	CD064099	5	4	weakly similar to SP|P51815|ZN75_HUMAN Zinc finger protein 75. [Human] {Homo sapiens}, partial (19%)
12704	attatgttca	1				no_annot
12705	tgtgatctca	1				no_annot
12706	ctaatacaaa	1	CD060073	61	1	similar to SP|P25319|FRH1_SCHMA Ferritin-1 heavy chain. [Blood fluke] {Schistosoma mansoni}, partial (19%)
12707	atgagaaact	1				no_annot
12708	cagtgcttct	1				no_annot
12709	atatatagat	1	CD111037	11	1	weakly similar to GP|19310393|gb|AAL84936.1||AY078930 At2g43990/F6E13.12 {Arabidopsis thaliana}, partial (3%)
12710	acttacttat	1				no_annot

12711	gtatccttcg	1				no_annot
12712	tccaaaactg	1	CD184218	65	0	
12713	caattttatt	1				no_annot
12714	tcgttattgg	1				no_annot
12715	cagctactcc	1				no_annot
12716	acaggaagct	1				no_annot
12717	ttcacgttgt	1	TC13629	34	5	similar to GP|5578916|emb|CAB51359.1||AJ133756 jim zinc finger protein {Drosophila melanogaster}, partial (4%)
12718	attcgcaata	1				no_annot
12719	gattattgtg	1	C609176.1	55	1	
12720	cactctccac	1				no_annot
12721	taggtgaatt	1	C607315.1	35	1	
12722	gatggataat	1				no_annot
12723	aggacgaggt	1				no_annot
12724	tgaggcccag	1				no_annot
12725	tacacgtgac	1				no_annot
12726	aatacaaaat	1				no_annot
12727	taatcatatt	1	TC17543	35	1	similar to GP|20988956|gb|AAH30330.1||BC030330 Unc84a protein {Mus musculus}, partial (10%)
12727	taatcatatt	1	CD155913	3	0	similar to GP|23326189|gb|AAN24767.1|AE014718_4|AE014718 permease protein of ABC transporter system {Bifidobacterium longum NCC2705}, partial (7%)
12728	cagcttgact	1				no_annot
12729	aagcgttgat	1				no_annot
12730	atatgaggat	1	AF074400|Sma.114	97	1	Calcium ATPase 2 (SMA2)
12731	tgtattgtgt	1	TC17473	21	2	
12732	tgctttcgtt	1				no_annot
12733	ggtgtctcga	1				no_annot
12734	gatggataaa	1	CD080120	69	0	
12735	tcataacgaa	1				no_annot
12736	gaaacatatt	1				no_annot

12737	ttcatttctg	1				no_annot
12738	tggccttgga	1				no_annot
12739	ggtactttga	1				no_annot
12740	gtaggtactg	1				no_annot
12741	ttgtagttac	1				no_annot
12742	acagctttat	1	TC18028	38	0	weakly similar to GP|17946436|gb|AAL49251.1||AY071629 RE67594p {Drosophila melanogaster}, partial (22%)
12743	taagtccggt	1	AI394925	27	1	
12744	tgtgatctag	1				no_annot
12745	gattgaaaaa	1				no_annot
12746	attatgttac	1	TC19439	49	2	weakly similar to GP|15808059|gb|AAC50484.2||U40271 transmembrane receptor precursor {Homo sapiens}, partial (10%)
12747	tgtaaaagtt	1				no_annot
12748	tgtcgaatgt	1	C207569.1	5	2	
12749	taattgtgca	1	C611785.1	42	0	
12750	cggagagggc	1				no_annot
12751	ggccgctcac	1				no_annot
12752	aaaagtactc	1				no_annot
12753	cagatgagcg	1				no_annot
12754	ttcatctcag	1				no_annot
12755	tagaatattc	1				no_annot
12756	ctttgtaacc	1				no_annot
12757	gcagcaaaac	1				no_annot
12758	gtcctcatcg	1				no_annot
12759	ccggcgtctc	1				no_annot
12760	ccaagctttc	1				no_annot
12761	ataatgctaa	1	CD093991	66	1	similar to SP|O97159|CHDM_DROME Chromodomain helicase-DNA-binding protein Mi-2 homolog (dMi-2). [Fruit fly] {Drosophila melanogaster}, partial (3%)
12762	gtcaattcat	1	C603505.1	22	3	
12763	cggcgtttcc	1				no_annot

12764	tccccatctc	1				no_annot
12765	aatgtgaaac	1				no_annot
12766	tcttctgaac	1				no_annot
12767	ttctattcac	1	CD184888	37	1	
12768	atggtatagc	1	CD081882	16	2	
12769	gtcgtctgat	1				no_annot
12770	cggcggccct	1				no_annot
12771	tcagttattc	1				no_annot
12772	catagcagaa	1				no_annot
12773	ttccagtgag	1	TC14962	52	0	
12773	ttccagtgag	1	CD132842	42	1	weakly similar to SP|O00233|PSD9_HUMAN 26S proteasome non-ATPase regulatory subunit 9 (26S proteasome regulatory subunit p27). [Human] {Homo sapiens}, partial (20%)
12774	ctaaaactcg	1				no_annot
12775	tgcaaagtgg	1				no_annot
12776	tttgtggcga	1				no_annot
12777	gcaccagttg	1				no_annot
12778	aacatttgta	1	TC18556	59	0	
12779	atgttaaagg	1				no_annot
12780	tgctaacagg	1				no_annot
12781	caaaatccgt	1				no_annot
12782	atgaataggg	1				no_annot
12783	atagtgagta	1				no_annot
12784	cggcggccca	1				no_annot
12785	gatcttaaag	1	C208297.1	25	5	
12786	cttgttattg	1				no_annot
12787	ggttaagctg	1				no_annot
12788	gaatgtttga	1				no_annot
12789	aaacattgcg	1				no_annot
12790	agaggtgtaa	1	C201896.1	74	1	
12790	agaggtgtaa	1	C606600.1	74	1	

12791	ttttatccgt	1				no_annot
12792	gaaacaaaga	1	TC15915	90	0	
12792	gaaacaaaga	1	AI977472	72	0	
12793	ccacaccagc	1	TC17449	48	1	homologue to GP|28395487|gb|AAO39081.1||AY191018 autophagy protein 4 {Dictyostelium discoideum}, partial (3%)
12794	ttagtaacag	1	TC13875	3	1	homologue to GP|1432175|gb|AAB04027.1||U63062 YELA {Dictyostelium discoideum}, partial (3%)
12794	ttagtaacag	1	TC13876	60	2	similar to SP|P54753|EPB3_HUMAN Ephrin type-B receptor 3 precursor(Tyrosine-protein kinase receptor HEK-2). [Human] {Homo sapiens}, partial (3%)
12795	gttacttcat	1				no_annot
12796	attggttctg	1				no_annot
12797	tatcaagttt	1	TC7753	90	0	weakly similar to GP|19570497|dbj|BAB86334.1||AB057596 VANGL1 {Homo sapiens}, partial (9%)
12798	tgaccataat	1				no_annot
12799	gttttgtgct	1				no_annot
12800	agtataatgg	1	TC17424	88	0	weakly similar to SP|Q99447|PCY2_HUMAN Ethanolamine-phosphate cytidylyltransferase(Phosphorylethanolamine transferase) (CTP:phosphoethanolamine cytidylyltransferase). [Human] {Homo sapiens}, partial (33%)
12801	cgaaaaaaaa	1				no_annot
12802	ggttcgtttc	1				no_annot
12803	taatacttat	1				no_annot
12804	ctgatgatga	1				no_annot
12805	tctatctcga	1	TC19673	15	0	
12806	caccgctgag	1				no_annot
12807	gaaaagcctt	1				no_annot
12808	tataattcct	1	TC14282	30	1	
12809	gaggaggggt	1				no_annot
12810	caacacactg	1				no_annot
12811	gttctgttct	1				no_annot
12812	tggttaaaaa	1	AI740245	4	2	

12813	gagtggattg	1	C205322.1	58	1	
12813	gagtggattg	1	C608143.1	40	0	
12814	tagggtttct	1				no_annot
12815	actatcatct	1	C209290.1	5	4	
12815	actatcatct	1	C606296.1	5	4	
12816	accactcagt	1				no_annot
12817	tcgtacatcg	1				no_annot
12818	aggacattct	1				no_annot
12819	gtgattgacc	1				no_annot
12820	ttctgctgct	1				no_annot
12821	acgaaatcga	1				no_annot
12822	aggacattca	1	AY168137|Sma.687	65	3	Leucine-rich protein (SMV0119)
12823	caaatcttta	1	CD180726	17	0	
12824	acttcaaaaa	1	CD202718	1	0	
12825	atgcgtttca	1				no_annot
12826	gtggtggaga	1	TC10511	75	2	weakly similar to GP|28564801|dbj|BAC57731.1||AP004401 P0534A03.23 {Oryza sativa (japonica cultivar-group)}, partial (9%)
12826	gtggtggaga	1	TC10515	61	2	
12827	gaaaatatgt	1				no_annot
12828	agccttaatg	1				no_annot
12829	gtgatactag	1				no_annot
12830	aatgaaacta	1				no_annot
12831	atgttgacat	1				no_annot
12832	ccgttattct	1				no_annot
12833	atcccgaact	1				no_annot
12834	ttccataaac	1				no_annot
12835	ggatataatg	1				no_annot
12836	atttttcagt	1	CD156248	64	0	
12837	aatttgtata	1	TC17503	98	0	weakly similar to GP|7303535|gb|AAF58590.1||AE003823 CG8344-PA {Drosophila melanogaster}, partial (31%)

12838	gatccaaaat	1				no_annot
12839	gaaaatatga	1				no_annot
12840	tctccagcaa	1				no_annot
12841	caagtttaca	1				no_annot
12842	tatacgcgca	1				no_annot
12843	ttcacgttat	1				no_annot
12844	ccaggtcgtg	1				no_annot
12845	tattaatatg	1				no_annot
12846	ggcgaccttt	1				no_annot
12847	atccacacct	1				no_annot
12848	gggtggagat	1				no_annot
12849	ttctgttgtg	1				no_annot
12850	gtaacgaatt	1				no_annot
12851	aaatctcgaa	1				no_annot
12852	gtttacttat	1				no_annot
12853	gtcagttgtg	1				no_annot
12854	tcaaccagga	1	TC10856	69	3	similar to GP|21745342|gb|AAM77350.1|AF520987_1|AF520987 LIMS2 {Homo sapiens}, partial (65%)
12855	tgcggccagt	1				no_annot
12856	accctttttt	1	TC13856	15	0	
12856	accctttttt	1	CD073316	54	1	
12857	ttaagtgtgt	1				no_annot
12858	aagcaaatcc	1				no_annot
12859	gtaacgaata	1				no_annot
12860	gatcccgcaa	1				no_annot
12861	tatcgttaaa	1				no_annot
12862	tcctctaaaa	1				no_annot
12863	ccggattttc	1				no_annot
12864	ttctgctgaa	1				no_annot
12865	gcgaaggcgg	1				no_annot

12866	ggctttgatt	1	C602410.1	52	3	
12867	gtgcgaaaac	1				no_annot
12868	tatttcagaa	1				no_annot
12869	aataatttgt	1	TC10641	96	0	homologue to GP|2687973|gb|AAC66478.1||AE001121 B. burgdorferi predicted coding region BB0079 {Borrelia burgdorferi B31}, partial (5%)
12869	aataatttgt	1	CD095759	6	1	
12869	aataatttgt	1	CD139859	14	0	
12870	ttaaatcatc	1				no_annot
12871	ccgttattac	1				no_annot
12872	tagagcgatt	1				no_annot
12873	acaataacac	1	TC14216	97	0	
12874	tgcacaatgt	1				no_annot
12875	agctttacca	1				no_annot
12876	attttggttt	1	TC13596	88	0	weakly similar to GP|20336352|gb|AAM18191.1||AY092757 SNAP25 fusion protein {Loligo pealei}, partial (37%)
12877	taatggaagt	1	C606835.1	54	2	
12878	aaatttactc	1				no_annot
12879	gctcgtggac	1	C302550.1	34	0	
12879	gctcgtggac	1	C715687.1	34	0	
12880	gaaattggga	1	C201637.1	27	2	
12880	gaaattggga	1	C611832.1	20	5	
12881	tttaaccgtt	1				no_annot
12882	ggtgcatctg	1	C300380.1	25	2	
12882	ggtgcatctg	1	C718551.1	25	2	
12883	caatgagtgc	1				no_annot
12884	ccaagtattt	1				no_annot
12885	aaattcacat	1	TC16224	41	0	
12886	cgcacatttg	1				no_annot
12887	agcctattcc	1				no_annot
12888	gttgttcgtt	1				no_annot

12889	atggacaaaa	1	C605619.1	41	1	
12890	tgattatttt	1				no_annot
12891	ttattgttag	1				no_annot
12892	agtatcaagt	1				no_annot
12893	agttactaaa	1	CD067673	36	1	
12894	ggcgcggcgg	1				no_annot
12895	taaattattt	1	TC10747	98	0	similar to GP|19386782|dbj|BAB86162.1||AP003537 B1140D12.10 {Oryza sativa (japonica cultivar-group)}, partial (17%)
12896	acgagcacga	1				no_annot
12897	cagacaggtc	1				no_annot
12898	tgttgttctt	1				no_annot
12899	gtacaaaacg	1				no_annot
12900	tgaaaaggaa	1	AF006678|Sma.1002	79	1	Lysophospholipase homolog (SmLPLH)
12901	cgcatacata	1				no_annot
12902	ctgctcagga	1				no_annot
12903	ggttcgatca	1	C201500.1	37	3	
12903	ggttcgatca	1	C609931.1	39	3	
12904	gtgatgcggg	1				no_annot
12905	atattcaata	1				no_annot
12906	tatagttgtt	1				no_annot
12907	aagcccagtg	1	C611742.1	31	2	
12908	caccgacccg	1	AW400406	58	0	
12909	atttttcacg	1	TC13507	56	2	homologue to GP|3063620|gb|AAC14119.1||AF056330 AUT1 {Schistosoma mansoni}, partial (16%)
12910	gttcgtcaat	1				no_annot
12911	tgttcacaag	1				no_annot
12912	atgagaggaa	1				no_annot
12913	gtacacgccg	1				no_annot
12914	atcaagaagc	1	N21919	18	0	
12915	cgcgtgtttc	1				no_annot
12916	tcataagttt	1				no_annot

12917	gctcagaatg	1	CD183396	11	0	
12917	gctcagaatg	1	CD168127	70	0	similar to GP|5597017|gb|AAC24982.2||AF025672 reverse transcriptase {synthetic construct}, partial (3%)
12918	cccccaaccg	1	TC10604	84	1	weakly similar to SP|Q99733|NPL4_HUMAN Nucleosome assembly protein 1-like 4 (Nucleosome assembly protein 2) (NAP2). [Human] {Homo sapiens}, partial (31%)
12918	cccccaaccg	1	TC10605	10	4	similar to PIR|D82572|D82572 TonB protein XF2327 [imported] - Xylella fastidiosa (strain 9a5c), partial (7%)
12918	cccccaaccg	1	TC18821	84	1	similar to GP|4679150|gb|AAD27038.1|AF095316_1|AF095316 cytochrome b {Heterotis niloticus}, partial (10%)
12919	cctatttctt	1				no_annot
12920	caatctgctt	1				no_annot
12921	gtctaaaaaa	1				no_annot
12922	ttttgttaca	1				no_annot
12923	gtaaccgata	1	TC17766	57	0	similar to GP|17861816|gb|AAL39385.1||AY069240 GM01519p {Drosophila melanogaster}, partial (13%)
12924	aatgatgttc	1	C210449.1	58	0	
12924	aatgatgttc	1	C610553.1	56	0	
12925	gctcagaatc	1	TC6948	5	2	similar to GP|19067879|gb|AAK14815.1||AY027869 polyprotein {Schistosoma japonicum}, partial (3%)
12925	gctcagaatc	1	TC6952	2	3	similar to GP|2130630|gb|AAC23724.1||AF000430 dynamin-like protein {Homo sapiens}, partial (14%)
12925	gctcagaatc	1	TC6954	2	1	homologue to GP|22164073|gb|AAM93547.1|AF412216_1|AF412216 polyprotein {Schistosoma japonicum}, partial (32%)
12925	gctcagaatc	1	TC7120	41	2	similar to GP|19067879|gb|AAK14815.1||AY027869 polyprotein {Schistosoma japonicum}, partial (11%)
12925	gctcagaatc	1	TC7168	67	1	similar to GP|19067879|gb|AAK14815.1||AY027869 polyprotein {Schistosoma japonicum}, partial (10%)
12925	gctcagaatc	1	TC7229	48	2	similar to GP|19067879|gb|AAK14815.1||AY027869 polyprotein {Schistosoma japonicum}, partial (12%)
12925	gctcagaatc	1	TC7252	58	0	similar to GP|19067879|gb|AAK14815.1||AY027869 polyprotein {Schistosoma japonicum}, partial (10%)

12925	gctcagaatc	1	TC13480	32	1	homologue to GP|160966|gb|AAA29870.1||J03982 eggshell protein precursor {Schistosoma mansoni}, partial (36%)
12925	gctcagaatc	1	TC15836	51	0	similar to GP|19067879|gb|AAK14815.1||AY027869 polyprotein {Schistosoma japonicum}, partial (11%)
12925	gctcagaatc	1	TC19233	47	0	weakly similar to GP|19067879|gb|AAK14815.1||AY027869 polyprotein {Schistosoma japonicum}, partial (9%)
12925	gctcagaatc	1	CD145176	84	0	similar to GP|19067879|gb|AAK14815.1||AY027869 polyprotein {Schistosoma japonicum}, partial (6%)
12925	gctcagaatc	1	CD190874	11	2	
12925	gctcagaatc	1	CD081664	73	0	similar to GP|22164073|gb|AAM93547.1|AF412216_1|AF412216 polyprotein {Schistosoma japonicum}, partial (47%)
12925	gctcagaatc	1	CD088345	63	0	similar to GP|19067879|gb|AAK14815.1||AY027869 polyprotein {Schistosoma japonicum}, partial (5%)
12925	gctcagaatc	1	CD091629	33	1	similar to GP|19067879|gb|AAK14815.1||AY027869 polyprotein {Schistosoma japonicum}, partial (8%)
12925	gctcagaatc	1	CD115955	75	0	similar to GP|19067879|gb|AAK14815.1||AY027869 polyprotein {Schistosoma japonicum}, partial (9%)
12925	gctcagaatc	1	CD132814	58	0	similar to GP|22164073|gb|AAM93547.1|AF412216_1|AF412216 polyprotein {Schistosoma japonicum}, partial (87%)
12926	gctaattcaa	1				no_annot
12927	gtcgcaagct	1	TC10806	3	3	weakly similar to GP|28194088|gb|AAO33395.1|AF468651_1|AF468651 karyopherin-beta 3 variant {Xenopus laevis}, partial (26%)
12928	acgttaagtc	1				no_annot
12929	ctcatatgat	1	C202242.1	52	0	
12929	ctcatatgat	1	C610958.1	49	0	
12930	ctattgttat	1	TC11929	95	0	
12931	atttctttat	1	CD177785	86	0	
12932	tctggtagag	1	TC14578	18	2	PIR|D40335|HSHU4 histone H4 [validated] - human, complete
12933	tgtattcttg	1				no_annot

12934	aagaaggtga	1	TC7894	75	2	weakly similar to GP|1196428|gb|AAA88032.1||M14123 neutral protease large subunit {Homo sapiens}, partial (27%)
12934	aagaaggtga	1	TC17548	64	0	similar to GP|28971805|dbj|BAC65455.1||AB087802 uridine diphosphate glucose pyrophosphatase {Homo sapiens}, partial (14%)
12935	gaattgggtg	1				no_annot
12936	aaactaatgc	1				no_annot
12937	tattagacag	1				no_annot
12938	ttgtatgtat	1				no_annot
12939	gttgcataaa	1				no_annot
12940	ttttagaatt	1	TC9151	94	0	
12941	tttgtttttt	1	TC8244	79	0	GP|15277863|gb|AAK94014.1|AF398551_1|AF398551 mucin-like protein MUC-loc2 {Trypanosoma cruzi}, partial (5%)
12941	tttgtttttt	1	TC9663	11	4	
12941	tttgtttttt	1	CD089288	26	0	weakly similar to GP|3243131|gb|AAC23966.1||AF045777 titin {Drosophila melanogaster}, partial (3%)
12941	tttgtttttt	1	BF936895	72	0	
12942	gatgtattta	1	AJ276884|Sma.89	51	1	Calcineurin A
12943	gtacaaaaag	1				no_annot
12944	caaaagaatc	1				no_annot
12945	gttcaaagtt	1				no_annot
12946	caaattattt	1				no_annot
12947	atccccgaac	1				no_annot
12948	ttgtatgtag	1	AI740263	5	1	
12949	atttttcaat	1	TC12302	68	2	
12950	ctgcagtctc	1				no_annot
12951	gtacaaaaaa	1	C609539.1	41	0	
12952	tctacatctg	1				no_annot
12953	aataaacagg	1	TC7046	81	2	weakly similar to SP|O96614|SER1_GALME Sericin-1 (Silk gum protein 1) (Fragment). [Wax moth] {Galleria mellonella}, partial (27%)

12954	ttgtatgtac	1				no_annot
12955	tagtgtttgg	1	C201152.1	10	2	
12955	tagtgtttgg	1	C208420.1	16	2	
12955	tagtgtttgg	1	C601013.1	12	2	
12955	tagtgtttgg	1	C602947.1	9	3	
12956	ttgtatgtaa	1				no_annot
12957	cgcgcacgca	1				no_annot
12958	tgcccgtaca	1				no_annot
12959	tcaaccagat	1	AI977416	96	0	
12959	tcaaccagat	1	CD062774	74	0	
12960	cgcattatca	1	C209407.1	85	1	
12961	taacaggaca	1	C203278.1	90	0	
12962	tatttctgga	1	TC11071	73	0	weakly similar to GP|19528553|gb|AAL90391.1||AY089653 RH12051p {Drosophila melanogaster}, partial (12%)
12963	cagacatata	1				no_annot
12964	tgaaaataac	1	CD128993	36	0	
12965	actttttaat	1				no_annot
12966	tgcacctatt	1				no_annot
12967	cagaaagacc	1				no_annot
12968	tattaaaaca	1	TC14553	84	0	similar to GP|17978533|gb|AAL17747.1||AY055726 synovial sarcoma associated SS18-beta {Mus musculus}, partial (8%)
12969	atcccgtagt	1	TC16370	10	3	
12969	atcccgtagt	1	AI395491	24	3	
12970	taaacaattt	1	TC9305	79	0	
12971	ctgctgctga	1				no_annot
12972	aaagaagcat	1				no_annot
12973	tatataaatg	1	CD086428	3	0	
12974	aattagatgt	1				no_annot
12975	agatgtgtca	1				no_annot
12976	gacggtacat	1				no_annot

12977	atgagataag	1				no_annot
12978	tagaacttat	1	CD162114	53	1	
12979	atgagataac	1				no_annot
12980	taaaacgatg	1	TC17865	73	0	
12981	tggcatcgaa	1				no_annot
12982	taatggaaca	1				no_annot
12983	aaagagctgt	1	AF030965|Sma.713	46	1	Clone A26 unknown
12984	tacttatcgc	1				no_annot
12985	tttaataaca	1	C609097.1	87	0	
12986	acaggatgag	1				no_annot
12987	ttgctacctt	1				no_annot
12988	ttgttgcgct	1				no_annot
12989	atcacaatgt	1	TC15076	74	0	
12990	agagaggttg	1				no_annot
12991	tcagtttttc	1	TC11846	24	3	
12992	tcatttattt	1				no_annot
12993	gtttttttagg	1				no_annot
12994	agcaagacta	1	C608226.1	70	0	
12995	atcaaagtac	1	TC8739	83	0	similar to GP|2104785|gb|AAB57835.1||AF000168 9ORF binding protein 1 {Mus musculus}, partial (7%)
12996	ttctcggttg	1				no_annot
12997	cttgtttttc	1				no_annot
12998	cagttgaatc	1				no_annot
12999	gtactttgtt	1				no_annot
13000	tcattcatat	1				no_annot
13001	gggaataatt	1				no_annot
13002	ttccattaca	1				no_annot
13003	atattatttg	1				no_annot
13004	gtggtgcatt	1				no_annot
13005	aatagccata	1				no_annot
13006	atacaacaat	1				no_annot

13007	tttcgcctat	1				no_annot
13008	tgccttctga	1	CD122502	71	0	
13009	cgattcggtt	1				no_annot
13010	taacttgtaa	1				no_annot
13011	gtactttgta	1				no_annot
13012	tgcaacgaag	1				no_annot
13013	agagtcactc	1				no_annot
13014	tgaaggaggt	1	CD196723	39	1	
13015	tggaattgag	1				no_annot
13016	accataaagg	1	TC16698	48	4	GP|160960|gb|AAA29867.1||M86397 epidermal growth factor receptor {Schistosoma mansoni}, complete
13016	accataaagg	1	CD146838	50	2	
13017	ggagatgggt	1				no_annot
13018	gctgagtcac	1				no_annot
13019	cactttgtat	1				no_annot
13020	tacgcgggat	1	TC18116	70	0	
13021	tatactcagc	1				no_annot
13022	aataaacact	1				no_annot
13023	ccaaagtgat	1				no_annot
13024	cctgtgtctc	1				no_annot
13025	gctagctgaa	1	TC12256	76	0	
13026	tagttcgata	1				no_annot
13027	gtgtgtctga	1				no_annot
13028	tgcaagtagt	1	C610872.1	7	1	
13029	gggtggtgca	1				no_annot
13030	gttacaatta	1				no_annot
13031	caatttggat	1				no_annot
13032	ataagaataa	1				no_annot
13033	tgctgactct	1	TC17332	78	0	
13034	atgcatcgca	1				no_annot
13035	tcgtattttt	1	C316227.1	31	0	

13036	agctactggc	1				no_annot
13037	aaagttacaa	1				no_annot
13038	gagctggaat	1	CD080664	72	0	
13039	cattaaaaaa	1				no_annot
13040	tactccgagc	1				no_annot
13041	gaagcagata	1				no_annot
13042	gatccataat	1				no_annot
13043	aatgctatta	1	TC12827	50	0	
13044	agggttgtct	1				no_annot
13045	ttacgaccaa	1				no_annot
13046	cggtataata	1				no_annot
13047	ctttgccgaa	1				no_annot
13048	gggagctttg	1				no_annot
13049	acaccaagca	1				no_annot
13050	aagacgatgc	1	TC13961	61	3	homologue to SP|P36417|GBF_DICDI G-box binding factor (GBF). [Slime mold] {Dictyostelium discoideum}, partial (4%)
13051	ttatcgtatt	1				no_annot
13052	ttgcagacat	1	CD153442	50	0	
13053	gatatttgtt	1	CD137890	40	1	
13054	atttatctat	1	TC10442	56	0	
13055	ggtttttctc	1	TC11516	95	0	weakly similar to SP|O43309|Z305_HUMAN Zinc finger protein 305. [Human] {Homo sapiens}, partial (9%)
13056	aattagatcc	1				no_annot
13057	caattttaca	1	C606835.1	11	3	
13058	aaatggactg	1				no_annot
13059	agggttgtcc	1				no_annot
13060	agggttgtca	1				no_annot
13061	gtaacagtgg	1				no_annot
13062	gatcaacaga	1				no_annot
13063	ctgttgcgat	1	TC12661	72	0	

13064	gtaacagtga	1	TC9682	42	2	weakly similar to PIR|I30010|I30010 NADH2 dehydrogenase (ubiquinone)chain 5 - Leishmania tarentolae mitochondrion, partial (4%)
13065	cccttggatg	1				no_annot
13066	taaaagggtc	1				no_annot
13067	atatgacggt	1	CD161030	59	0	
13068	tgtggttcaa	1				no_annot
13069	ctctgaccag	1				no_annot
13070	agctcatcgt	1				no_annot
13071	ttaggggtaa	1				no_annot
13072	tgattaataa	1				no_annot
13073	tacgattctt	1	C201382.1	85	0	
13074	tgttgtacag	1				no_annot
13075	ttaactggac	1				no_annot
13076	ctcgtaaaat	1				no_annot
13077	gtaggatgcg	1				no_annot
13078	tttttggccc	1	TC13580	16	18	weakly similar to SP|Q00963|SPCB_DROME Spectrin beta chain. [Fruit fly] {Drosophila melanogaster}, partial (15%)
13078	tttttggccc	1	CD082065	23	0	
13079	tgttgatgag	1				no_annot
13080	tagacaaaaa	1				no_annot
13081	tacaatcaca	1				no_annot
13082	gttatggccg	1				no_annot
13083	gaaacataca	1				no_annot
13084	gggaagagca	1				no_annot
13085	ggatataagt	1	TC13591	32	8	similar to GP|3153910|gb|AAC17451.1||AF066859 muscle glycogen phosphorylase {Homo sapiens}, partial (91%)
13086	gggcaactaa	1	TC11156	58	0	weakly similar to GP|16445289|gb|AAL23507.1||AE006471 conjugative transfer: DNA transport {Salmonella typhimurium LT2}, partial (3%)
13087	gattattgat	1				no_annot

13088	tacgttctgt	1				no_annot
13089	caatatttga	1				no_annot
13090	gttgctgcat	1				no_annot
13091	taacacaccg	1				no_annot
13092	ttaagatcag	1				no_annot
13093	tgcaaccatc	1				no_annot
13094	ggagatggct	1				no_annot
13095	aattagatag	1				no_annot
13096	atcaatgata	1	TC15482	28	0	weakly similar to SP|O95218|Z265_HUMAN Zinc finger protein 265 (Zinc finger, splicing). [Human] {Homo sapiens}, partial (8%)
13096	atcaatgata	1	CD077304	6	1	
13097	agatctggag	1	TC17815	42	0	
13098	atgtaattga	1				no_annot
13099	gaaatccgaa	1				no_annot
13100	gacaaatgta	1	CD082166	43	2	
13101	gcgattaaaa	1				no_annot
13102	catttttgtg	1				no_annot
13103	tggaacatta	1				no_annot
13104	gaatagttta	1				no_annot
13105	tttgttatat	1	TC11513	63	0	
13106	acgatggtta	1				no_annot
13107	tttgagaaaa	1				no_annot
13108	taggtgaaaa	1				no_annot
13109	catttttgta	1				no_annot
13110	tacaatcaat	1				no_annot
13111	aacgtgtggt	1				no_annot
13112	agtcaaacag	1				no_annot
13113	ttatttgtat	1				no_annot
13114	agaacagaga	1				no_annot
13115	agcggataca	1				no_annot

13116	ggcagtgaaa	1				no_annot
13117	tttttggcag	1				no_annot
13118	attgtgcttt	1				no_annot
13119	gtatttcaat	1				no_annot
13120	acaacacttt	1				no_annot
13121	ggtgctgtca	1				no_annot
13122	gaaacataaa	1				no_annot
13123	taaaagtatc	1	CD155067	89	0	
13124	tctccctgag	1	TC13163	97	0	
13125	tgattgtgta	1				no_annot
13126	tgtctttgaa	1				no_annot
13127	ttgcaaaaaa	1				no_annot
13128	acaacactta	1	TC14457	70	0	
13129	aagaccgtga	1				no_annot
13130	atcgtgatat	1	TC10604	37	3	weakly similar to SP|Q99733|NPL4_HUMAN Nucleosome assembly protein 1-like 4 (Nucleosome assembly protein 2) (NAP2). [Human] {Homo sapiens}, partial (31%)
13130	atcgtgatat	1	TC10606	95	0	similar to GP|2605800|gb|AAC14590.1||AF027736 minor ampullate silk protein {Nephila clavipes}, partial (6%)
13130	atcgtgatat	1	TC18578	57	0	similar to GP|7291002|gb|AAF46440.1||AE003446 CG32708-PA {Drosophila melanogaster}, partial (10%)
13131	aagtcactaa	1	CD150552	16	0	
13131	aagtcactaa	1	CD091629	17	3	similar to GP|19067879|gb|AAK14815.1||AY027869 polyprotein {Schistosoma japonicum}, partial (8%)
13132	aaaagatgaa	1				no_annot
13133	cccaaaacaa	1				no_annot
13134	ggagatggat	1				no_annot
13135	ctcagaaacc	1				no_annot
13136	tattccacaa	1				no_annot
13137	aaattatggt	1				no_annot
13138	tgcttgatgt	1	C200676.1	1	2	
13138	tgcttgatgt	1	C611665.1	1	2	

13139	atgtgctttt	1				no_annot
13140	tgtcgtggta	1				no_annot
13141	tagcttcact	1	TC10727	55	2	similar to GP|12964610|dbj|BAB32671.1||AB047391 pre-mRNA processing 8 protein {Mus musculus}, partial (13%)
13142	gtccagaaag	1				no_annot
13143	atagatattt	1	CD073369	46	1	
13144	acgtttactc	1	TC13982	81	0	weakly similar to SP|P45700|M1A1_MOUSE Mannosyl-oligosaccharide 1,2-alpha-mannosidase IA(Processing alpha-1,2-mannosidase IA) (Alpha-1,2-mannosidase IA) (Mannosidase alpha class 1A member 1) (Man(9)-alpha-mannosidase). [Mouse] {Mus musculus}, partial (43%)
13145	acaccttctc	1				no_annot
13146	caaaaagtgt	1	C208653.1	85	0	
13146	caaaaagtgt	1	C602473.1	86	0	
13147	aaagactggg	1				no_annot
13148	tgtaactcct	1				no_annot
13149	gctctagaaa	1				no_annot
13150	ttaactcgtg	1	C711228.1	42	1	
13151	caacaatgtt	1				no_annot
13152	gatagaatca	1				no_annot
13153	gtttcataat	1	TC14137	72	1	weakly similar to SP|P17152|PM1_HUMAN Protein PM1. [Human] {Homo sapiens}, partial (23%)
13154	tgcattttat	1				no_annot
13155	gtttaacagt	1				no_annot
13156	tactgggtag	1	TC18500	54	0	weakly similar to GP|7547035|gb|AAF63767.1|AF251041_1|AF251041 SGC32445 protein {Homo sapiens}, partial (38%)
13157	tttgatgata	1				no_annot
13158	tactccgaag	1				no_annot
13159	tttgggatgt	1	CD118864	35	0	similar to PIR|S66072|S66072 yabG protein - Bacillus subtilis, partial (35%)
13160	gttatcttca	1	TC19087	65	0	
13161	cagacaggga	1				no_annot

13162	tcgtatatca	1				no_annot
13163	tacgttctct	1				no_annot
13164	gcaatagacc	1				no_annot
13165	tagattggat	1				no_annot
13166	agcgattggc	1	TC7437	34	2	weakly similar to GP|6899846|dbj|BAA90542.1||AB034205 cisplatin resistance-associated overexpressed protein {Homo sapiens}, partial (20%)
13166	agcgattggc	1	TC7438	7	2	weakly similar to GP|21711793|gb|AAM75087.1||AY128494 RH42690p {Drosophila melanogaster}, partial (10%)
13167	cgcattttcg	1				no_annot
13168	agaaaaacaa	1				no_annot
13169	gatgactttg	1	C200793.1	91	0	
13169	gatgactttg	1	C205187.1	55	1	
13169	gatgactttg	1	C608850.1	89	0	
13170	taaggtgact	1				no_annot
13171	atagacatac	1				no_annot
13172	ttcacatcac	1				no_annot
13173	catccatttg	1	TC13024	18	0	
13174	gggaatggtg	1				no_annot
13175	aatgtactac	1				no_annot
13176	aatattcggg	1				no_annot
13177	tgttgttcgc	1				no_annot
13178	ttcgccagtc	1				no_annot
13179	catcaagtcc	1				no_annot
13180	ttatcagtac	1				no_annot
13181	gcccaatttt	1				no_annot
13182	accgggtgaa	1				no_annot
13183	gatcaacaac	1	C600579.1	22	2	
13184	gtatgttctc	1				no_annot
13185	taatggtact	1				no_annot

13186	cttgaggggt	1				no_annot
13187	gatcaacaaa	1				no_annot
13188	ctcgacaagt	1				no_annot
13189	taaacgagta	1				no_annot
13190	tatatatata	1	TC13621	44	0	
13190	tatatatata	1	CD076968	94	0	
13190	tatatatata	1	CD083544	81	0	
13190	tatatatata	1	CD134607	20	0	
13190	tatatatata	1	AI014218	64	1	
13190	tatatatata	1	AW087120	71	1	
13191	ctcgaaatag	1				no_annot
13192	ccttcggttt	1				no_annot
13193	caaatcttac	1				no_annot
13194	gccaatcaat	1	TC15937	12	2	
13195	tctcattaat	1	C710631.1	4	1	
13196	cgtaactcct	1				no_annot
13197	tcagggatgt	1				no_annot
13198	agttgtatca	1	TC14006	20	5	
13199	cattgctata	1				no_annot
13200	ctagggctta	1				no_annot
13201	ttgaaaagta	1				no_annot
13202	tagacgtccc	1				no_annot
13203	ggttacagca	1	TC6875	18	0	
13204	cagaggaacc	1				no_annot
13205	gatgtattga	1	C314308.1	57	0	
13206	tgcatacaca	1	TC14393	4	4	
13207	ataagattcc	1				no_annot
13208	cttgacgaat	1				no_annot
13209	caacctgatg	1				no_annot
13210	gtatgatggt	1				no_annot

13211	atcaagtaca	1				no_annot
13212	ctaaaggttt	1				no_annot
13213	actggtcatc	1				no_annot
13214	tcccaacccg	1				no_annot
13215	gtttggggat	1				no_annot
13216	aaaggaaact	1				no_annot
13217	tcccaacccc	1				no_annot
13218	tagaatttgc	1	TC10812	34	2	
13219	aaaccaaata	1	CD113976	41	0	
13220	ggttacagat	1				no_annot
13221	cttttttttc	1				no_annot
13222	ttgttggttt	1				no_annot
13223	aacgtgtgat	1				no_annot
13224	tacataagta	1				no_annot
13225	ggctatgtgt	1				no_annot
13226	ggaacaaatt	1	CD091621	14	2	
13227	ccttgaccaa	1	TC18975	71	0	
13228	ttacagagtc	1				no_annot
13229	ttgacagtgg	1	CD179052	87	0	
13230	actagtcgaa	1	CD184798	60	0	
13231	ccgtatatat	1				no_annot
13232	tagcggcagt	1	TC10687	26	0	similar to SP|P24928|RPB1_HUMAN DNA-directed RNA polymerase II largest subunit(RPB1). [Human] {Homo sapiens}, partial (10%)
13233	tttttacatt	1	TC17238	93	0	
13234	actaatgatt	1				no_annot
13235	aacctgatat	1				no_annot
13236	ttgaaggacg	1				no_annot
13237	agtatctaac	1				no_annot
13238	tttggcgtga	1				no_annot
13239	tgactgatcc	1				no_annot

13240	tactcgggag	1				no_annot
13241	actaatgatg	1				no_annot
13242	aggcctcaca	1				no_annot
13243	ctaaatcaca	1				no_annot
13244	gtcaaaatgg	1				no_annot
13245	cctactgtag	1				no_annot
13246	catataaagt	1				no_annot
13247	tatagttgca	1				no_annot
13248	gacaaaagaa	1	CD194466	92	0	
13249	gtcaaaatga	1	TC7365	50	16	PIR|I51382|I51382 achaete-scute homolog - chicken, partial (7%)
13249	gtcaaaatga	1	CD140628	75	0	weakly similar to GP|25012791|gb|AAN71487.1||BT001732 RE70963p {Drosophila melanogaster}, partial (25%)
13250	aggtcgtatt	1				no_annot
13251	aattaatctc	1				no_annot
13252	tgagcaaagc	1				no_annot
13253	aaagactgag	1				no_annot
13254	atagacttgg	1				no_annot
13255	gtgattcacc	1	TC8134	76	0	weakly similar to GP|15291159|gb|AAK92848.1||AY051424 GH10029p {Drosophila melanogaster}, partial (40%)
13256	tgagcaaaga	1				no_annot
13257	tgagaagaaa	1	TC16512	64	0	
13258	cattttagaa	1	CD153124	49	1	similar to GP|2145046|gb|AAB58406.1||AF000409 merozoite surface protein-1 {Plasmodium berghei}, partial (13%)
13259	gtcattgctg	1				no_annot
13260	tcatcatttg	1				no_annot
13261	atccgagcag	1				no_annot
13262	taaaagaaat	1	TC14162	59	1	weakly similar to PIR|S10501|LUCH2 annexin II - chicken, partial (9%)
13262	taaaagaaat	1	AA269094	31	3	

13263	aaaggaaaaa	1	TC7418	2	1	GP|2246652|gb|AAC62254.1||AF006678 lysophospholipase homolog {Schistosoma mansoni}, partial (15%)
13264	tgatatattt	1	TC16980	62	2	weakly similar to SP|O43791|SPOP_HUMAN Speckle-type POZ protein. [Human] {Homo sapiens}, partial (28%)
13265	attcaacggt	1				no_annot
13266	gttggtgaac	1				no_annot
13267	gatagcaaca	1	TC14591	79	2	
13268	gagtcattac	1	TC9283	49	0	similar to SP|Q10588|BST1_HUMAN ADP-ribosyl cyclase 2 precursor(Cyclic ADP-ribose hydrolase 2) (cADPr hydrolase 2) (Bone marrow stromal antigen 1) (BST- 1) (CD157 antigen). [Human] {Homo sapiens}, partial (5%)
13269	ctgttggttg	1	CD152825	48	0	
13270	tagtagttta	1	TC14349	74	0	similar to GP|17945837|gb|AAL48965.1||AY071343 RE37932p {Drosophila melanogaster}, partial (56%)
13270	tagtagttta	1	CD067209	56	1	
13271	tggtagtgct	1				no_annot
13272	taaattatat	1	TC7212	16	9	
13273	tttgttttct	1				no_annot
13274	tattcaagaa	1				no_annot
13275	cagagtgatt	1				no_annot
13276	cattggtgta	1				no_annot
13277	caacaaagca	1				no_annot
13278	ttgattgtat	1				no_annot
13279	aattccatac	1				no_annot
13280	gtctatgcat	1				no_annot
13281	ggaatctagg	1				no_annot
13282	aactttggtt	1	CD074496	23	1	
13283	attattctgg	1				no_annot
13284	gagtatgagt	1	TC18656	22	3	
13285	tgttgttcaa	1				no_annot
13286	tgctggcgaa	1				no_annot

13287	ataagtggtg	1	C603853.1	85	0	
13288	gtgaatctga	1				no_annot
13289	aattaaacgt	1				no_annot
13290	aagagaaata	1	C201882.1	80	0	
13290	aagagaaata	1	C600242.1	92	0	
13291	cttattccac	1				no_annot
13292	aagccgctcg	1				no_annot
13293	ctgtttcaca	1				no_annot
13294	ccaaattaaa	1				no_annot
13295	agcaaaaaga	1				no_annot
13296	gttattatca	1	TC8160	44	0	
13296	gttattatca	1	TC17793	53	1	weakly similar to GP|5649170|gb|AAD43131.2|AF159092_1|AF159092 syld709613 protein {Homo sapiens}, partial (8%)
13297	gcataacaat	1				no_annot
13298	ctcttaaaag	1	CD123557	71	0	
13299	tactattcta	1				no_annot
13300	tcagtacagg	1				no_annot
13301	aacaggtgat	1				no_annot
13302	gatacctccc	1				no_annot
13303	agtgcttgaa	1				no_annot
13304	tttaggccgc	1				no_annot
13305	atcttaacta	1	CD060526	66	2	
13305	atcttaacta	1	CD090450	1	3	
13306	tatttttggg	1	TC12917	26	1	
13307	gttcggcttc	1	W06714	48	1	similar to GP|12958632|gb|AAK09382.1|AF321922_1|AF321922 purine-nucleoside phosphorylase {Schistosoma mansoni}, partial (28%)
13308	caacaaagag	1				no_annot
13309	aaatagctat	1	C211019.1	9	2	
13309	aaatagctat	1	C605167.1	9	2	

13310	gaattgtact	1	AA528883	63	1	
13311	gatttcaagt	1	CD089812	21	2	
13312	actaagggca	1				no_annot
13313	atagcatata	1	TC17142	55	0	similar to GP|18558797|gb|AAL47945.1||AY065540 vomeronasal receptor V1RH14 {Mus musculus}, partial (9%)
13314	tcgccgaact	1				no_annot
13315	attaaatgga	1	C209273.1	38	0	
13316	aggaatcagt	1				no_annot
13317	agtttgaatc	1				no_annot
13318	ttacgcattc	1				no_annot
13319	gtcaactatt	1				no_annot
13320	ttgcaataat	1				no_annot
13321	cttgttttcg	1	TC10927	93	0	
13322	agtcctatac	1				no_annot
13323	tctgccagtc	1				no_annot
13324	gaattgtaca	1				no_annot
13325	tatattgctt	1	C203208.1	82	0	
13325	tatattgctt	1	C611942.1	79	0	
13326	tgtaatacac	1				no_annot
13327	tactaatggt	1	AI977382	57	0	
13328	tctcgacagc	1	CD077517	82	0	
13329	tggattaatt	1	CD159498	47	2	PIR|JQ1320|JQ1320 high mobility group protein Pf16 - malaria parasite (Plasmodium falciparum), partial (8%)
13330	tcgttccgat	1				no_annot
13331	agcagcaatt	1				no_annot
13332	ttggtggtca	1				no_annot
13333	taaacaatac	1	CD165978	21	0	
13334	agtaacttac	1	TC10554	80	3	similar to GP|8099346|gb|AAF72103.1|AF154845_1|AF154845 MARK {Homo sapiens}, partial (13%)
13335	taaaccaaga	1				no_annot

13336	gcctcgtaca	1				no_annot
13337	cgtattctag	1				no_annot
13338	gatgttggtt	1				no_annot
13339	actttgtttg	1				no_annot
13340	gcttggaggg	1				no_annot
13341	gaatagttga	1				no_annot
13342	ttgcggtgtc	1	C603542.1	18	3	
13343	tgaagtaact	1				no_annot
13344	gggtgtcgta	1	TC14287	30	2	weakly similar to SP|Q9UIJ5|ZDH2_HUMAN Zinc finger DHHC domain containing protein 2 (Zinc finger protein 372) (Reduced expression associated with metastasis protein) (Ream) (Reduced expression in cancer protein) (Rec). [Human] {Homo sapiens}, partial (24%)
13345	ggggttatgg	1				no_annot
13346	tctgtatact	1				no_annot
13347	ttgaagcata	1	TC8093	57	0	weakly similar to GP|21391928|gb|AAM48318.1||AY118289 AT18469p {Drosophila melanogaster}, partial (15%)
13348	ataagctaaa	1	C203647.1	62	0	
13349	tttagtttct	1				no_annot
13350	tccttctcta	1				no_annot
13351	atgagacgaa	1	TC13034	6	4	
13352	agtaatatgg	1				no_annot
13353	aacagtaaaa	1				no_annot
13354	actaccaaat	1	TC8610	68	1	
13355	atatatattc	1				no_annot
13356	tacaaagcta	1	TC8731	17	3	
13357	ttactccctg	1				no_annot
13358	tcgtatttca	1	CD183764	60	2	
13359	acaacactgc	1				no_annot
13360	cacgattcga	1	CD076753	4	1	
13361	atagacttag	1				no_annot
13362	actaccaaag	1				no_annot

13363	aattccttga	1				no_annot
13364	tcgatttttg	1				no_annot
13365	accagctttg	1	C209036.1	2	0	
13365	accagctttg	1	C609765.1	2	0	
13366	ctgcttagaa	1	C201164.1	67	0	
13366	ctgcttagaa	1	C608646.1	63	0	
13367	atttgttcac	1	C606169.1	95	0	
13368	ccaacaccaa	1				no_annot
13369	tttgcattaa	1				no_annot
13370	ttttacgaat	1				no_annot
13371	ataagtaaga	1				no_annot
13372	gcaacaatca	1				no_annot
13373	caaaacgaaa	1				no_annot
13374	gaattgcgtc	1	TC11770	75	2	weakly similar to GP|27497737|gb|AAO13168.1||AY171233 protein tyrosine phosphatase {Homo sapiens}, partial (6%)
13375	tagatgattt	1	TC9659	93	0	
13375	tagatgattt	1	TC11312	69	1	
13376	ctgtggcagg	1				no_annot
13377	gtacatcctg	1				no_annot
13378	ggggtacatc	1				no_annot
13379	gaaagtgact	1				no_annot
13380	tgaacttcct	1	C301574.1	59	0	
13381	ttatcgtaca	1				no_annot
13382	gcagtaactt	1				no_annot
13383	acgctattgt	1	TC14265	86	3	weakly similar to SP|Q92544|T9S4_HUMAN Transmembrane 9 superfamily protein member 4. [Human] {Homo sapiens}, partial (53%)
13384	ctgcggtgtc	1				no_annot
13385	tgtttctgtt	1				no_annot
13386	aagatatgca	1	X98619|Sma.12	12	2	Sm10 protein
13387	tacgattcct	1				no_annot

13388	ctcaattggg	1				no_annot
13389	gtgaatctag	1				no_annot
13390	tggtgttttc	1	CD089475	54	0	
13391	gagtatgaat	1				no_annot
13392	cacaaagctt	1				no_annot
13393	atagatatga	1				no_annot
13394	gtgaatctaa	1	TC18313	78	0	
13395	actcaatgaa	1				no_annot
13396	actaagtaac	1	TC10554	64	6	similar to GP|8099346|gb|AAF72103.1|AF154845_1|AF154845 MARK {Homo sapiens}, partial (13%)
13396	actaagtaac	1	TC13446	23	1	
13397	ctttatgatt	1				no_annot
13398	gactgttcag	1				no_annot
13399	attgttagtt	1	TC17224	98	0	similar to SP|Q16831|UDP_HUMAN Uridine phosphorylase(UDRPase). [Human] {Homo sapiens}, partial (8%)
13400	ggatattaag	1				no_annot
13401	ttgaacacaa	1				no_annot
13402	aaatctgtag	1	C200780.1	37	2	
13402	aaatctgtag	1	C612571.1	37	2	
13403	tcaacttgga	1				no_annot
13404	agcaccacgt	1	TC15570	64	1	
13405	gggttagagc	1				no_annot
13406	taacctgagc	1				no_annot
13407	gttcaatggg	1				no_annot
13408	tgcaaccaca	1				no_annot
13409	cactagcacg	1				no_annot
13410	gttaagtagc	1				no_annot
13411	tgcacgtgag	1				no_annot
13412	taaacattgt	1	CD135269	7	2	

13413	ctaggagcac	1	TC10427	20	2	similar to GP|22080666|emb|CAC82104.1||AJ278736 muscle protein {Homo sapiens}, partial (7%)
13414	aggctgacac	1				no_annot
13415	tgggtgaatg	1				no_annot
13416	tttgctggtc	1	TC16822	48	4	
13417	aagaatagtc	1				no_annot
13418	aactacttca	1	TC14106	28	4	
13418	aactacttca	1	CD127875	67	3	
13419	tgcagtgtgc	1				no_annot
13420	gaagacctgg	1	CD086280	54	0	similar to GP|15292063|gb| LD37002p {Drosophila melanogaster}, partial (3%)
13421	atacttgtta	1				no_annot
13422	atgtgggcac	1				no_annot
13423	ttcaattgcc	1	TC19117	23	0	
13424	tgccgttgtc	1				no_annot
13425	ttactgtcaa	1	TC7741	58	0	similar to GP|5679146|gb|AAD46879.1|AF160939_1|AF160939 BcDNA.LD18931 {Drosophila melanogaster}, partial (62%)
13426	tgtgaaaaca	1				no_annot
13427	gagacagatc	1	TC13898	24	10	similar to SP|Q9Y297|FW1A_HUMAN F-box/WD-repeat protein 1A (F-box and WD-repeats protein beta-TrCP) (E3RSIkappaB) (pIkappaBalpha-E3 receptor subunit). [Human] {Homo sapiens}, partial (50%)
13428	ggttgaaaaa	1	TC15381	74	1	
13428	ggttgaaaaa	1	CD094692	35	3	
13429	tataaaatac	1	C610174.1	97	0	
13430	tattctacca	1	U30261|Sma.677	2	4	G protein beta subunit-like protein trans-spliced
13431	gactaaaatt	1				no_annot
13432	ccatcaatat	1				no_annot
13433	ttccagatgt	1				no_annot
13434	cttaggccat	1				no_annot
13435	caacgctgtg	1				no_annot

13436	caagaggata	1	C609913.1	8	5	
13437	ctttagtagc	1	Z29947|Sma.26	56	1	ATPase (putative)
13438	atataagaga	1				no_annot
13439	tgtcatcccc	1				no_annot
13440	aatatcgtca	1				no_annot
13441	gttgttgttt	1				no_annot
13442	taggcagaga	1				no_annot
13443	aaacaagaac	1				no_annot
13444	tagatcgtta	1				no_annot
13445	acagaaaagt	1	TC6882	24	1	weakly similar to GP|23477189|emb|CAD22046.1||AJ428954 tissue factor pathway inhibitor-2 {Rattus norvegicus}, partial (23%)
13446	caaacattgg	1				no_annot
13447	catatataga	1	C315099.1	12	2	
13447	catatataga	1	C315099.1	12	1	
13447	catatataga	1	C315099.1	12	0	
13447	catatataga	1	C602509.1	22	2	
13447	catatataga	1	C602509.1	22	1	
13447	catatataga	1	C602509.1	22	0	
13448	tcgacaatgc	1				no_annot
13449	agaactttga	1				no_annot
13450	cgtttagcct	1				no_annot
13451	ttgttggtgc	1				no_annot
13452	acctcttcag	1				no_annot
13453	tatccattat	1				no_annot
13454	tgttaaggtg	1				no_annot
13455	ggaacaaaga	1	C606401.1	66	0	
13456	aaagattggt	1				no_annot
13457	tattgttatt	1				no_annot
13458	ttttcattga	1				no_annot
13459	ctcatcaatt	1				no_annot

13460	gatttgagca	1				no_annot
13461	ttacataata	1				no_annot
13462	caaatacagc	1	TC7698	51	1	
13463	taaattttag	1				no_annot
13464	gattccgctg	1				no_annot
13465	tcggcacgcc	1				no_annot
13466	agttacgtta	1				no_annot
13467	gatctgatcg	1				no_annot
13468	agcggacgca	1				no_annot
13469	catccattca	1	TC17711	73	0	
13469	catccattca	1	AW087116	5	1	
13470	tttgaactaa	1				no_annot
13471	agtccgccct	1				no_annot
13472	tttatgtatg	1	M21308|Sma.702	18	6	Asparaginyl endopeptidase (c197 Sm32 gene)
13473	tatttcattt	1	TC17146	82	0	similar to GP|16226045|gb|AAL16062.1|AF420278_1|AF420278 carbonyl reductase {Anguilla japonica}, partial (16%)
13474	taaacattct	1				no_annot
13475	gtatgttcca	1				no_annot
13476	ttgtgatgaa	1	TC7731	86	2	
13477	ttcggagagt	1				no_annot
13478	agttgacaaa	1	TC8635	26	1	weakly similar to GP|15450581|gb|AAK96562.1||AY052658 AT4g39960/T5J17_130 {Arabidopsis thaliana}, partial (14%)
13479	ccggtgtttc	1				no_annot
13480	taccgcatct	1				no_annot
13481	cataccagta	1	TC8467	61	0	similar to GP|7290773|gb|AAF46218.1||AE003439 CG14432-PA {Drosophila melanogaster}, partial (14%)
13481	cataccagta	1	CD093260	0	0	
13482	agtccgcccc	1				no_annot
13483	ctcaattgag	1				no_annot
13484	gcatttcgat	1				no_annot

13485	attgagacaa	1	CD156754	29	1	
13486	tcttcggtag	1				no_annot
13487	tgaggacttg	1				no_annot
13488	cttctcaccc	1				no_annot
13489	tggaggaata	1				no_annot
13490	aagtcggttt	1				no_annot
13491	tcttaaatac	1				no_annot
13492	cgatcaaagc	1				no_annot
13493	ttaatgcata	1				no_annot
13494	ctttcgagga	1				no_annot
13495	tgtgtttgtt	1				no_annot
13496	gatttctagt	1				no_annot
13497	tcgcaaagac	1				no_annot
13498	gcaactaatc	1	TC13882	64	3	similar to GP|29825445|gb|AAO92283.1||AY241962 26S proteasome regulatory subunit {Dermacentor variabilis}, partial (94%)
13499	tcgcaaagaa	1				no_annot
13500	caaaagtaaa	1				no_annot
13501	gttttgatga	1				no_annot
13502	gcaaccaaat	1				no_annot
13503	gtagtgctgg	1	CD165761	15	4	similar to GP|7299276|gb|AAF54471.1||AE003685 CG5358-PA {Drosophila melanogaster}, partial (17%)
13504	cagctggtaa	1				no_annot
13505	tcaatgtata	1	CD077072	14	1	
13506	taattcgtgt	1				no_annot
13507	aatacagttt	1				no_annot
13508	tattgtaagc	1				no_annot
13509	gcgtcctttg	1				no_annot
13510	tgttaatatg	1	TC9678	58	1	
13511	actaccggaa	1				no_annot
13512	tcgccgtaca	1				no_annot
13513	gcgtccttta	1				no_annot

13514	tgcttaacca	1				no_annot
13515	gatagtaaga	1	CD066613	62	0	
13516	gcgtacgtca	1				no_annot
13517	gattacatag	1				no_annot
13518	catttcattt	1	AY227022|Sma.285	62	4	SINA (SINA)
13519	agaatggttc	1				no_annot
13520	tttttttaa	1	C202140.1	95	0	
13521	accaattatg	1	CD079350	26	0	
13522	ttcatattta	1	C607586.1	9	3	
13523	agtcttcaac	1				no_annot
13524	cccaatctgt	1				no_annot
13525	aggcaaatcc	1				no_annot
13526	caaatgccaa	1				no_annot
13527	taaacattac	1				no_annot
13528	gtaaaatgcg	1				no_annot
13529	tgctttgtgt	1				no_annot
13530	aacatcatcc	1				no_annot
13531	tggatttatc	1				no_annot
13532	ttttgtgttg	1				no_annot
13533	gtgctcaaag	1				no_annot
13534	tattggtgcg	1	C609026.1	34	1	
13535	gtgatggtcg	1				no_annot
13536	tcgagtcgaa	1				no_annot
13537	ggagagtttt	1	TC8488	91	0	
13538	acttttgtta	1				no_annot
13539	tgtgaccctc	1	TC7744	42	1	similar to GP|11559534|gb|AAG37999.1|AF249296_1|AF249296 dihydropyrimidinase {Mus musculus}, partial (11%)
13540	gtcgttatta	1				no_annot
13541	aaaatcagca	1				no_annot
13542	gtcaaattat	1				no_annot

13543	ttaatcacaa	1				no_annot
13544	cctactctgt	1	TC17880	1	3	similar to SP|Q04446|GLGB_HUMAN 1,4-alpha-glucan branching enzyme(Glycogen branching enzyme) (Brancher enzyme). [Human] {Homo sapiens}, partial (35%)
13545	ccaatgtatc	1	TC14826	33	2	
13545	ccaatgtatc	1	CD060904	38	1	
13546	taacattcga	1				no_annot
13547	cggatctacc	1				no_annot
13548	cttttttat	1				no_annot
13549	tgaagttaca	1				no_annot
13550	gaataaacaa	1				no_annot
13551	tgccaaattt	1	CD186532	67	0	
13552	cctttaggca	1				no_annot
13553	taaaatgaga	1				no_annot
13554	agtaatttga	1				no_annot
13555	gggtttatga	1				no_annot
13556	tttaccattg	1				no_annot
13557	gatgggtatg	1				no_annot
13558	aaatatagca	1				no_annot
13559	gccgtgactt	1	C600899.1	78	0	
13560	cactgacctt	1	TC9038	8	0	
13561	gttgtatcta	1				no_annot
13562	tgcccggtta	1				no_annot
13563	cataataata	1				no_annot
13564	tgtgaaggcg	1				no_annot
13565	ttttaactgt	1	TC10988	83	0	weakly similar to GP|22833076|gb|AAF46565.3||AE003449 CG2967-PA {Drosophila melanogaster}, partial (4%)
13566	aattctatca	1				no_annot
13567	gtaaaatgaa	1	TC17771	50	1	similar to GP|1749794|emb|CAA66229.1||X97630 serine/threonine protein kinase {Homo sapiens}, partial (30%)

13567	gtaaaatgaa	1	TC18515	86	0	weakly similar to SP|Q9BXI6|EP64_HUMAN EBP50-PDZ interactor of 64 kDa (EPI64 protein). [Human] {Homo sapiens}, partial (27%)
13568	agcagaatac	1				no_annot
13569	aaatggtccg	1	TC11158	44	2	weakly similar to GP|12805489|gb|AAH02220.1||BC002220 Clk4 protein {Mus musculus}, partial (8%)
13570	agtacaggaa	1				no_annot
13571	cgcgatactt	1	CD141973	56	0	
13572	atatacttac	1	TC13895	43	0	similar to GP|12247854|gb|AAG50053.1|AF332983_1|AF332983 eIF4E-binding protein 3 {Danio rerio}, partial (43%)
13573	gagactattg	1				no_annot
13574	tagagtgaat	1				no_annot
13575	tattggtgaa	1	M83294|Sma.264	37	2	Triose phosphate isomerase
13576	ctttgtgtta	1				no_annot
13577	cataacaaaa	1	TC10015	0	2	weakly similar to SP|O13547|CW14_YEAST Covalently-linked cell wall protein 14 precursor (Inner cell wall protein). [Baker's yeast] {Saccharomyces cerevisiae}, partial (15%)
13577	cataacaaaa	1	TC10015	0	0	weakly similar to SP|O13547|CW14_YEAST Covalently-linked cell wall protein 14 precursor (Inner cell wall protein). [Baker's yeast] {Saccharomyces cerevisiae}, partial (15%)
13578	gaacccactg	1				no_annot
13579	tttaagtttc	1	TC17436	80	0	weakly similar to GP|11321422|gb|AAG34161.1|AF312926_1|AF312926 focal contact protein paxillin {Drosophila melanogaster}, partial (24%)
13580	ttctttgcat	1				no_annot
13581	tggggacaag	1				no_annot
13582	tggaaagaca	1				no_annot
13583	gagctttcgt	1				no_annot
13584	ttgttcagtg	1				no_annot
13585	tcgaagtagc	1	TC11423	47	0	
13586	gtaacactac	1				no_annot

13587	acgccgtcta	1				no_annot
13588	ttacacggcc	1				no_annot
13589	ttctgcatcc	1	TC7499	46	6	
13590	caccttcctt	1				no_annot
13591	catattgcgc	1				no_annot
13592	ctcataatgc	1				no_annot
13593	ccacccgtga	1	C301024.1	81	0	
13593	ccacccgtga	1	C602736.1	55	1	
13594	ttgctcattc	1				no_annot
13595	aatatgttat	1	TC13769	74	0	weakly similar to GP|5668703|dbj|BAA82642.1||AB030502 XDRP1 {Xenopus laevis}, partial (30%)
13596	agtttagtag	1				no_annot
13597	tctagtgcat	1				no_annot
13598	aacacaattc	1				no_annot
13599	gattcgtcta	1	TC14859	95	0	similar to GP|4219081|gb|AAD12303.1||AF110638 cytochrome b {Himantura pacifica}, partial (6%)
13600	actacgtgca	1	CD081531	55	1	
13600	actacgtgca	1	CD081532	55	1	
13601	ttatctgaac	1				no_annot
13602	atgatttatt	1	AI067495	93	0	homologue to GP|20515976|gb| predicted membrane-associated HD superfamily hydrolase {Thermoanaerobacter tengcongensis}, partial (1%)
13603	gtgggtgctg	1	TC17143	27	3	weakly similar to GP|10726648|gb|AAG22161.1||AE003730 CG5085-PA {Drosophila melanogaster}, partial (50%)
13604	gtgctctagt	1				no_annot
13605	cattgtaact	1				no_annot
13606	tgttcggact	1				no_annot
13607	taaagaacgt	1	C302165.1	94	0	
13607	taaagaacgt	1	C706694.1	94	0	
13608	ctgacgtaaa	1				no_annot
13609	gaaattgtta	1				no_annot

13610	ttctgtattc	1				no_annot
13611	gtgggaaaaa	1				no_annot
13612	cagagtgaac	1				no_annot
13613	gagcctgtcg	1				no_annot
13614	accaagtgaa	1				no_annot
13615	ttttgcctta	1				no_annot
13616	agaacacaat	1				no_annot
13617	catcaactac	1				no_annot
13618	tggacaaaga	1				no_annot
13619	tgttcggacc	1	TC8371	85	0	weakly similar to SP|O95922|TTLL_HUMAN Tubulin tyrosine ligase-like protein 1. [Human] {Homo sapiens}, partial (32%)
13620	tggatgtgaa	1				no_annot
13621	tggaattttc	1	TC18876	97	0	
13621	tggaattttc	1	CD111760	84	0	similar to GP|148017|gb|AAA24682.1||M37393 transposition protein D {Escherichia coli}, partial (54%)
13622	tttatgaaaa	1	AF074400|Sma.114	34	12	Calcium ATPase 2 (SMA2)
13623	tggacgacct	1				no_annot
13624	tacattacgt	1				no_annot
13625	gccactgttc	1				no_annot
13626	gactttagtg	1				no_annot
13627	aggcacaaca	1				no_annot
13628	acttcgaata	1	CD118820	45	1	
13629	tgaacaatta	1				no_annot
13630	gtgtagctca	1				no_annot
13631	tgaaacgatc	1	TC14918	89	0	
13632	ccatcattaa	1				no_annot
13633	taattgttgt	1				no_annot
13634	attgagtcct	1				no_annot
13635	cggaacttcg	1				no_annot
13636	gtccctaaaa	1	AY308018|Sma.749	85	0	LTR-retrotransposon Boudicca nonfunctional RNaseH and reverse transcriptase mRNA, partial sequence

13637	catttggctg	1				no_annot
13638	gattcatatt	1	TC18239	68	0	
13639	tacaacaggt	1	C210626.1	79	2	
13639	tacaacaggt	1	C612207.1	72	2	
13640	taattgttga	1				no_annot
13641	gaaatgttgg	1				no_annot
13642	tatttagatt	1				no_annot
13643	gaacttagtc	1				no_annot
13644	gaaacagttt	1	TC7546	58	1	homologue to GP|16904828|gb|AAL30898.1||AF438343 phosphoglycerate mutase {Schistosoma japonicum}, complete
13645	atatatatcc	1				no_annot
13646	cttggtgtct	1				no_annot
13647	agaaatctat	1				no_annot
13648	aaaaaaaacc	1				no_annot
13649	ggtggacggg	1				no_annot
13650	caaaaccagt	1				no_annot
13651	accgcattta	1				no_annot
13652	gaattgcgct	1				no_annot
13653	ctaatctcgc	1				no_annot
13654	attttgctaa	1				no_annot
13655	ttgggaattt	1	TC13818	98	0	similar to GP|4099082|gb|AAD00539.1||U82808 muscle-specific serine kinase 1 {Homo sapiens}, partial (32%)
13655	ttgggaattt	1	CD097449	69	0	
13656	gttaggttat	1	AF031196|Sma.99	90	0	Histamine-responsive G-protein coupled receptor (GPCR)
13657	atttgcaatt	1	AI977072	78	0	synaptotagmin
13658	acgcaagaag	1				no_annot
13659	ttaaggactt	1				no_annot
13660	tttatcgaat	1				no_annot
13661	aaagtaatgg	1	TC14344	38	2	
13661	aaagtaatgg	1	CD200554	65	1	

13662	tagttactaa	1	TC10003	35	3	weakly similar to SP|Q01658|TBAP_HUMAN TATA-binding protein-associated phosphoprotein (Down-regulator of transcription 1) (DR1 protein). [Human] {Homo sapiens}, partial (20%)
13663	tcttaattat	1				no_annot
13664	tagttccaga	1				no_annot
13665	tgcgaaagac	1	C201151.1	38	0	
13665	tgcgaaagac	1	C607893.1	34	0	
13666	aaacatttga	1	CD114108	22	0	
13667	caaaatgaat	1	CD154006	12	0	
13667	caaaatgaat	1	CD185103	56	0	similar to PIR|A35419|A35419 neutrophil protein - pig (fragment), partial (5%)
13668	atatatatat	1	BG930331	86	0	
13668	atatatatat	1	AI976735	18	1	
13668	atatatatat	1	AI559075	7	0	
13668	atatatatat	1	CD081916	10	2	
13669	ttggtcagcg	1				no_annot
13670	gggagacagc	1				no_annot
13671	caattgttga	1				no_annot
13672	aatattgtgt	1	TC16818	92	0	
13672	aatattgtgt	1	CD167967	94	0	
13673	agagatagta	1				no_annot
13674	cgaccgaagg	1	TC8029	19	1	
13675	ttggagacga	1				no_annot
13676	gtagaaaaac	1				no_annot
13677	ctacattatt	1				no_annot
13678	aaaaaaaaat	1	CD137712	45	0	
13679	gtagaaaaaa	1				no_annot
13680	atgaacttca	1	C607693.1	52	0	
13681	cgacttgtat	1				no_annot
13682	caaaatgaac	1				no_annot

13683	gccagaaatc	1	CD121713	39	0	weakly similar to GP|17944346|gb|AAL48065.1||AY070594 RE69515p {Drosophila melanogaster}, complete
13684	ggtaagtcga	1				no_annot
13685	ttacataagc	1	TC15107	6	1	similar to SP|Q37374|COX3_ACACA Cytochrome c oxidase polypeptide III. [Amoeba] {Acanthamoeba castellanii}, partial (5%)
13686	aaaaaaaaag	1	TC14552	17	1	weakly similar to SP|O04486|RB1C_ARATH Ras-related protein Rab11C. [Mouse-ear cress] {Arabidopsis thaliana}, partial (36%)
13686	aaaaaaaaag	1	CD187413	91	0	weakly similar to GP|2895557|gb|AAC40065.1||AF037071 carboxyl-terminal PDZ ligand of neuronal nitric oxide synthase {Rattus norvegicus}, partial (12%)
13687	aataaaagtt	1				no_annot
13688	caatcagttg	1	TC14580	63	2	
13688	caatcagttg	1	CD077564	60	0	
13689	tccagatgaa	1				no_annot
13690	atgatgtgat	1				no_annot
13691	catattgcac	1				no_annot
13692	gtattagcaa	1	CD165956	27	0	
13693	caatcagtta	1				no_annot
13694	tttgctggct	1	AI975486	15	0	
13695	ctaccagcaa	1				no_annot
13696	accgagaacg	1				no_annot
13697	gtgaaaaatt	1				no_annot
13698	gtcgtcttcg	1	CD080934	53	0	
13699	acatagacat	1				no_annot
13700	ttgaaatgat	1	CD189729	81	0	homologue to GP|3930133|gb|AAD10112.1||U82793 melanoma receptor tyrosine kinase {Xiphophorus helleri} [Xiphophorus hellerii], partial (43%)
13701	tttcacaggt	1				no_annot
13702	attggaataa	1	TC7934	59	0	weakly similar to GP|4809026|gb|AAD30062.1||AF132856 suppressor of G2 allele of skp1 homolog {Homo sapiens}, partial (32%)

13703	aaggacaaca	1				no_annot
13704	aagaatagcc	1				no_annot
13705	tcgaagcgtg	1				no_annot
13706	gtgaaaaatg	1				no_annot
13707	ctaataagaa	1				no_annot
13708	ttgaatacac	1				no_annot
13709	tgcatcactt	1				no_annot
13710	tataactaat	1				no_annot
13711	ctttcctaaa	1				no_annot
13712	gtgaaaaata	1				no_annot
13713	tcctaagcct	1				no_annot
13714	ctgaataccc	1				no_annot
13715	tccctattga	1				no_annot
13716	tcatatgaag	1				no_annot
13717	tataactaag	1				no_annot
13718	tgactatcca	1				no_annot
13719	agcttgtgtg	1				no_annot
13720	attatcgcta	1	M85304|Sma.283	32	7	Homeodomain protein (smox-5)
13721	cctcagatac	1				no_annot
13722	caattccttg	1				no_annot
13723	cgctcagtag	1	TC18126	83	0	
13724	cagtaaacat	1	C211376.1	69	0	
13724	cagtaaacat	1	C609094.1	69	0	
13725	cacacaacga	1				no_annot
13726	cacaaagcaa	1	C605927.1	74	1	
13727	aatttggttt	1	TC12414	83	0	
13728	gaataatcag	1	TC16942	75	0	similar to SP|P51610|HFC1_HUMAN Host cell factor C1 (HCF) (VP16 accessory protein) (HFC1) (VCAF) (CFF). [Human] {Homo sapiens}, partial (18%)
13729	ttaccaccta	1				no_annot
13730	cagtaaacag	1	C204876.1	9	0	

13731	atgaatatgt	1	TC18823	20	2	
13732	ggagatgtta	1	C609888.1	16	11	
13733	tagttccact	1				no_annot
13734	acaataaatg	1	TC8088	92	0	weakly similar to GP|23171634|gb|AAF55546.2||AE003721 CG7675-PB {Drosophila melanogaster}, partial (8%)
13735	cagtaaacaa	1				no_annot
13736	tgtactcacg	1				no_annot
13737	gacctcttgc	1				no_annot
13738	tcaaagatac	1				no_annot
13739	caattgttct	1				no_annot
13740	aggcagagaa	1	TC11175	40	1	homologue to GP|9246916|gb|AAF86198.1|AF238885_1|AF238885 leucyl-tRNA-synthetase {Bacillus anthracis}, partial (11%)
13741	agcagctagt	1				no_annot
13742	tgcaatcaat	1				no_annot
13743	gaaggaagaa	1				no_annot
13744	tgttagtcct	1	TC15133	3	1	
13745	ggattcgagc	1				no_annot
13746	atttggagtt	1				no_annot
13747	ctaatgcagt	1				no_annot
13748	ggaacataag	1				no_annot
13749	gaacaaaaca	1				no_annot
13750	tacaacagac	1				no_annot
13751	ttttctaact	1	C300037.1	88	0	
13751	ttttctaact	1	C606676.1	74	0	
13752	tacaacagaa	1	TC8564	34	3	
13753	ctaatttcta	1	TC7744	88	0	similar to GP|11559534|gb|AAG37999.1|AF249296_1|AF249296 dihydropyrimidinase {Mus musculus}, partial (11%)
13754	ttttatcatt	1	TC11766	46	3	weakly similar to GP|11182364|dbj|BAB17922.1||AB033759 NCBE {Mus musculus}, partial (18%)

13754	ttttatcatt	1	TC16283	67	1	similar to GP|6630459|gb|AAF19547.1|AC007190_15|AC007190 F23N19.12 {Arabidopsis thaliana}, partial (6%)
13754	ttttatcatt	1	AI975845	3	3	
13755	ccttatggtt	1				no_annot
13756	gtgtggacac	1	TC7364	11	4	similar to GP|3892185|gb|AAC78302.1||AF064591 protein disulfide isomerase {Schistosoma japonicum}, complete
13757	tgacaatgta	1				no_annot
13758	ggaaaacaga	1				no_annot
13759	atcacccgat	1				no_annot
13760	gtcttggcgg	1				no_annot
13761	aactatttac	1				no_annot
13762	gtctgccgat	1				no_annot
13763	ttttatcatc	1				no_annot
13764	gattttgggt	1	C209267.1	12	0	
13764	gattttgggt	1	C612336.1	63	0	
13765	tgtatgtcta	1				no_annot
13766	cgtctcttac	1	TC16071	88	0	
13767	tcatcgtgat	1	TC9535	12	1	weakly similar to GP|25988816|gb|AAN76273.1|AF453745_1|AF453745 proton/amino acid transporter 3 {Mus musculus}, partial (7%)
13768	ccctcgttca	1				no_annot
13769	atcaaggttt	1				no_annot
13770	cacttaaata	1				no_annot
13771	aaaaaatagg	1				no_annot
13772	aacgttcgct	1				no_annot
13773	taggctttgg	1				no_annot
13774	aaagtcaagg	1				no_annot
13775	tatgagtatg	1				no_annot
13776	cataataaga	1				no_annot

13777	gtccgcctac	1	TC6921	37	3	similar to GP|13508744|emb|CAC35115.1||AJ299689 NADH dehydrogenase subunit 6 {Ficedula parva}, partial (10%)
13778	cgcgcgcgtg	1				no_annot
13779	gttgttgtag	1				no_annot
13780	cttgtggatc	1				no_annot
13781	ttatgctgct	1				no_annot
13782	ctttgtgtgt	1				no_annot
13783	aaagtcaaga	1				no_annot
13784	gatatcatcc	1				no_annot
13785	acgcatatcc	1				no_annot
13786	cactgaccgg	1	C200395.1	7	2	
13787	aaatgtgcag	1	TC8356	24	1	similar to GP|17861970|gb|AAL39462.1||AY069317 LD03179p {Drosophila melanogaster}, partial (18%)
13788	tatgtagaaa	1				no_annot
13789	ccttgtattc	1	TC10712	95	0	weakly similar to GP|1399838|gb|AAB09671.1||U59212 transmembrane protein {Caenorhabditis elegans}, partial (6%)
13789	ccttgtattc	1	TC10713	28	0	similar to SP|Q9VAS7|INX3_DROME Innexin Inx3 (Innexin-3). [Fruit fly] {Drosophila melanogaster}, partial (10%)
13790	gattcaaaac	1	C310195.1	64	0	
13791	tttgcttaac	1				no_annot
13792	cgttctactg	1				no_annot
13793	tttgcttaaa	1				no_annot
13794	aagctgtatc	1				no_annot
13795	cacgtaaagt	1	C201118.1	12	2	
13795	cacgtaaagt	1	C604209.1	11	2	
13796	cgctgggtca	1				no_annot
13797	cttcagctgc	1				no_annot
13798	ccagtggatt	1	TC10938	45	0	weakly similar to GP|21553659|gb|AAM62752.1||AY085528 UDP-galactose 4-epimerase-like protein {Arabidopsis thaliana}, partial (84%)

13799	aacatacata	1	CD169722	19	4	
13800	tacaagctac	1				no_annot
13801	agtgtactac	1				no_annot
13802	ataccgaacg	1	C202106.1	18	5	
13803	tataataact	1				no_annot
13804	ttcacaaata	1	CD128799	71	0	
13805	ccaacctttc	1				no_annot
13806	atcgattctg	1				no_annot
13807	gtgtctcttt	1				no_annot
13808	actgctgatg	1	TC17028	38	2	weakly similar to SP|Q9D8B3|CTH8_MOUSE Protein c20orf178 homolog. [Mouse] {Mus musculus}, partial (54%)
13809	tttctatgac	1				no_annot
13810	attggcaaac	1				no_annot
13811	ataaccaatt	1				no_annot
13812	atacttcttg	1				no_annot
13813	atgaatatca	1	CD201436	38	0	
13814	tacgtgacag	1				no_annot
13815	tgttgaatac	1				no_annot
13816	gcttgtggat	1				no_annot
13817	gacttttgtc	1				no_annot
13818	aaagaagatc	1				no_annot
13819	tatggcgttc	1	AY551181|Sma.1208	74	0	Putative divalent metal transporter DMT1B
13820	ccaatgggag	1				no_annot
13821	tgaggactaa	1				no_annot
13822	aactaacaag	1	TC11647	38	0	
13823	cgcgagcact	1				no_annot
13824	ttcggtttgg	1				no_annot
13825	aagtacaagt	1				no_annot
13826	cttgcttaac	1	TC10255	86	0	
13826	cttgcttaac	1	CD128828	26	0	

13827	tactcggttg	1				no_annot
13828	gatggccata	1	TC13500	24	4	GP|3892187|gb|AAC78303.1||AF064592 RNA-binding protein {Schistosoma japonicum}, complete
13828	gatggccata	1	TC13509	21	4	GP|3892187|gb|AAC78303.1||AF064592 RNA-binding protein {Schistosoma japonicum}, complete
13829	ataatcttcg	1	TC8035	27	3	
13829	ataatcttcg	1	TC10799	67	1	weakly similar to GP|4138422|emb|CAA04022.1||AJ000347 3'(2'),5'-bisphosphate nucleotidase {Rattus norvegicus}, partial (38%)
13830	ggataaactt	1	CD114601	40	0	weakly similar to GP|2217968|emb|CAA73006.1||Y12337 myotonic dystrophy protein kinase like protein {Homo sapiens}, partial (13%)
13831	tttatgcgtc	1	TC8999	14	1	
13831	tttatgcgtc	1	CD081502	17	1	
13832	ctattgactt	1				no_annot
13833	gaactttgtt	1	TC11678	78	0	
13834	gatagccgat	1				no_annot
13835	ttacgcttac	1				no_annot
13836	gaaattgtga	1				no_annot
13837	atatcataaa	1				no_annot
13838	cttatgtaat	1				no_annot
13839	tttaaccttt	1	TC13555	61	5	
13840	cgtgggagag	1	U39883|Sma.210	33	1	Y-box binding protein
13841	gaaaaaagtt	1				no_annot
13842	aattcacaat	1				no_annot
13843	taataaagtg	1				no_annot
13844	ggattcgaat	1	TC14985	56	1	similar to GP|6694371|gb|AAF25206.1||AF200187 EBNA2-like protein {cercopithicine herpesvirus 15} [Cercopithicine herpesvirus 15], partial (3%)
13845	atactatcaa	1				no_annot
13846	ataatttttt	1	TC7786	56	2	
13847	gagtcgatta	1	TC8620	69	3	similar to GP|20152061|gb|AAM11390.1||AY095062 RE01809p {Drosophila melanogaster}, partial (4%)

13848	acagtttggc	1				no_annot
13849	tgaacaatgt	1	TC11166	11	5	weakly similar to GP|16945233|emb|CAD11441.1||AJ418713 limpet {Drosophila melanogaster}, partial (47%)
13850	ttcaatatta	1	AY172995|Sma.1012	91	0	Peptidylglycine alpha hydroxylating mono-oxygenase
13851	aatacgaact	1				no_annot
13852	agactcggga	1				no_annot
13853	tataataaaa	1				no_annot
13854	aaatacattg	1				no_annot
13855	tcaatgcgtt	1				no_annot
13856	cttgctcgtg	1				no_annot
13857	ttcatattac	1	TC8086	84	2	similar to GP|16611713|gb|AAL27306.1|AF376481_1|AF376481 BgaC {Carnobacterium piscicola}, partial (8%)
13858	taggctttcg	1				no_annot
13859	gtcgagaaat	1	C316386.1	45	0	
13859	gtcgagaaat	1	C607552.1	11	3	
13860	tagacgaatt	1				no_annot
13861	cttgctcgtc	1				no_annot
13862	tacactgtca	1				no_annot
13863	aatccaagcg	1				no_annot
13864	tcatctgggt	1				no_annot
13865	tgtttaccat	1				no_annot
13866	aataagcatc	1				no_annot
13867	tcccttggtt	1				no_annot
13868	tgtgacccat	1				no_annot
13869	atccaaatgt	1				no_annot
13870	gagagcacgt	1				no_annot
13871	gcaagcctgt	1				no_annot
13872	tgttggtgtc	1				no_annot
13873	cccctattaa	1				no_annot
13874	cattgtcgtg	1				no_annot

13875	ttggtctgct	1				no_annot
13876	tatatacgat	1				no_annot
13877	tttactattg	1				no_annot
13878	cactcggttg	1				no_annot
13879	cctcaatctg	1				no_annot
13880	tacgtaaaaa	1	CD094098	77	2	
13880	tacgtaaaaa	1	CD117660	44	0	
13880	tacgtaaaaa	1	BF936772	32	2	
13881	gttgatactt	1				no_annot
13882	caataaagtt	1				no_annot
13883	ttaaaacctg	1				no_annot
13884	agagattgta	1	C202198.1	63	0	
13884	agagattgta	1	C602649.1	35	1	
13885	tcaccaacat	1				no_annot
13886	tgcagtctct	1				no_annot
13887	tgggatgttt	1	TC8117	81	1	similar to GP|19909841|dbj|BAB87810.1||AB025426 HOYS7 {Homo sapiens}, partial (59%)
13888	tatgcaatgg	1				no_annot
13889	gtctggattt	1				no_annot
13890	ttttcttaga	1	C202175.1	34	2	
13890	ttttcttaga	1	C603834.1	34	2	
13891	ctcccagacg	1				no_annot
13892	gaacaaggct	1				no_annot
13893	cataataaaa	1				no_annot
13894	aactgactga	1				no_annot
13895	attcagaact	1				no_annot
13896	aagctgaact	1				no_annot
13897	tgatctgcaa	1				no_annot
13898	caacaaatta	1				no_annot
13899	aataaatgtc	1				no_annot
13900	atgattggag	1				no_annot

13901	ggtacaagat	1	TC16139	21	0	weakly similar to GP|22832170|gb|AAN09317.1||AE003490 CG4407-PB {Drosophila melanogaster}, partial (18%)
13902	taatactctt	1	TC8450	78	0	
13903	gtgtgataga	1	TC13880	8	7	weakly similar to SP|Q13614|MTR2_HUMAN Myotubularin-related protein 2. [Human] {Homo sapiens}, partial (38%)
13904	attgcgcact	1				no_annot
13905	tcaatactgg	1				no_annot
13906	ttctcctgaa	1	T14479	42	0	
13907	tctatcaaag	1				no_annot
13908	acctttatct	1				no_annot
13909	gatatcttgg	1				no_annot
13910	acctagaaca	1	C202173.1	60	1	
13910	acctagaaca	1	C316174.1	83	1	
13910	acctagaaca	1	C611269.1	39	3	
13910	acctagaaca	1	C706608.1	83	1	
13911	tgtgtcattt	1				no_annot
13912	tctatcaaaa	1				no_annot
13913	cgctcccata	1				no_annot
13914	gtatgatttg	1				no_annot
13915	aaaacaagca	1	C201431.1	3	4	
13915	aaaacaagca	1	C709290.1	19	3	
13916	tgtccaataa	1				no_annot
13917	gggtatgagt	1				no_annot
13918	taaacttaca	1				no_annot
13919	tacttgacgc	1	TC13866	64	4	
13920	agacgtccaa	1				no_annot
13921	atgttgaagt	1				no_annot
13922	atggtgtagc	1				no_annot

13923	gatttttaat	1	CD179803	41	0	weakly similar to GP|11182094|emb|CAC16125.1||AJ251006 orotidine-phosphate decarboxylase {Saccharomyces pastorianus}, partial (32%)
13924	aaacccccct	1				no_annot
13925	catttagaga	1				no_annot
13926	aagtaattga	1	TC7940	52	1	similar to GP|3641346|gb|AAC36355.1||AF091341 apoferritin-2 {Schistosoma japonicum}, partial (98%)
13927	acataataca	1	TC8419	75	1	
13928	ttgttaccta	1				no_annot
13929	gtcgccaagc	1				no_annot
13930	aacaccaagt	1				no_annot
13931	caatcagtga	1				no_annot
13932	gtttcagtgt	1				no_annot
13933	gagtgtctca	1				no_annot
13934	atttaataac	1	TC7404	35	0	similar to SP|P58374|RL30_BRABE 60S ribosomal protein L30. [Amphoxius] {Branchiostoma belcheri}, partial (86%)
13935	aacaccaagg	1				no_annot
13936	tcaaagttac	1				no_annot
13937	gccgcggtgg	1				no_annot
13938	tacaattgtc	1				no_annot
13939	atttaataaa	1	TC8617	22	3	
13940	catttcttgt	1				no_annot
13941	aacaccaagc	1				no_annot
13942	tatgttatag	1	TC15591	75	0	
13943	aacaccaaga	1	CD067092	30	2	
13944	caactgtctg	1				no_annot
13945	tatacctgcc	1				no_annot
13946	aagaattgaa	1				no_annot
13947	gtgatgctca	1				no_annot
13948	tcgcgttgtt	1				no_annot
13949	ttcacaggtt	1				no_annot

13950	aatgtgcttg	1				no_annot
13951	tcttgttttc	1				no_annot
13952	aaaagattgg	1	TC17701	18	1	
13953	ataacaatga	1	C210389.1	82	0	
13953	ataacaatga	1	C610249.1	73	1	
13954	gacaacaata	1				no_annot
13955	tgaaatgatg	1	TC17481	89	0	
13956	ggtttcaaga	1	CD122577	0	1	
13957	tttttggata	1	CD164206	78	0	
13958	gattttcgtg	1				no_annot
13959	taactatata	1	T14642	4	1	
13960	gatggactga	1				no_annot
13961	gatcagcata	1				no_annot
13962	tatgaacttg	1				no_annot
13963	gctagcttat	1	TC17709	53	1	
13964	aacaagtagg	1				no_annot
13965	caaggcagtg	1				no_annot
13966	atggccttta	1	TC13952	8	1	weakly similar to SP|P36543|VATE_HUMAN Vacuolar ATP synthase subunit E(V-ATPase E subunit) (Vacuolar proton pump E subunit) (V-ATPase 31 kDa subunit) (P31). [Human] {Homo sapiens}, partial (75%)
13967	tacactcttg	1				no_annot
13968	aacattttgt	1	TC10753	67	4	weakly similar to GP|161038|gb|AAA29903.1||M15509 major egg antigen {Schistosoma mansoni}, partial (24%)
13968	aacattttgt	1	TC17884	1	5	similar to PIR|S21173|S21173 ATP citrate (pro-S)-lyase - human, partial (9%)
13969	tgtattgctc	1				no_annot
13970	taatcagtca	1				no_annot
13971	acaataaagt	1				no_annot
13972	gctgaagtaa	1				no_annot
13973	acttatactg	1				no_annot
13974	acacagaaac	1				no_annot

13975	ttttgatcaa	1	CD131566	55	1	similar to PIR|H84288|H84288 3-oxoacyl-[acyl-carrier-protein] reductase [imported] - Halobacterium sp. NRC-1, partial (9%)
13975	ttttgatcaa	1	CD196472	55	0	similar to GP|27526325|emb protoscolex specific coiled-coil protein {Echinococcus multilocularis}, partial (3%)
13976	gtgtgataca	1				no_annot
13977	acacttatag	1				no_annot
13978	tgtcggatct	1				no_annot
13979	cggaagccca	1				no_annot
13980	cgtcttttgt	1				no_annot
13981	attgaacggt	1				no_annot
13982	tatgatgatg	1				no_annot
13983	attgctacaa	1				no_annot
13984	catagacata	1				no_annot
13985	gtttttgtat	1	TC7341	4	4	SP|P72983|Y20L_SYNY3 Ycf20-like protein. [strain PCC 6803] {Synechocystis sp.}, partial (10%)
13986	tatttagaat	1				no_annot
13987	acatcaagca	1				no_annot
13988	agcttctaac	1	BE431259	4	2	
13989	cctacaaatg	1				no_annot
13990	atgatccgaa	1	TC10552	10	3	
13991	aaggtacgga	1	AF316827|Sma.610	35	1	Zinc finger protein SmZF1
13992	gacatcgcga	1				no_annot
13993	acgttcaatg	1				no_annot
13994	atgtagtttc	1	TC8365	56	1	
13995	attatgacat	1				no_annot
13996	acaatgacca	1				no_annot
13997	atggtgtacg	1	TC7712	95	0	
13998	cacagtgcta	1				no_annot
13999	aactgccagt	1				no_annot
14000	gtttttgtac	1				no_annot

14001	aggtgttgtt	1				no_annot
14002	gatatttttt	1	TC13844	3	5	
14003	taagcgtaca	1				no_annot
14004	cgaacaatat	1	C604710.1	78	0	
14005	aaccctgagt	1				no_annot
14006	cataatggcg	1				no_annot
14007	catacttgtc	1				no_annot
14008	tagattgtga	1				no_annot
14009	tgcactcgat	1				no_annot
14010	caatcagtca	1	TC7322	81	0	
14011	tactgttgca	1	TC10759	98	0	weakly similar to GP|1549241|gb|AAC50694.1||U66616 SWI/SNF complex 170 KDa subunit {Homo sapiens}, partial (7%)
14012	tccccaaagt	1				no_annot
14013	gacgggtggt	1				no_annot
14014	gatattttta	1	TC11076	57	0	
14015	aatccgcact	1				no_annot
14016	tatcatcaaa	1	TC15357	56	1	
14017	ctgatgacta	1	TC11285	38	1	weakly similar to GP|7299193|gb|AAF54390.1||AE003683 CG9378-PA {Drosophila melanogaster}, partial (49%)
14018	aacaccaacc	1	TC7379	48	3	
14019	acagggtggc	1				no_annot
14020	tgttggagac	1				no_annot
14021	tttcgaaaat	1				no_annot
14022	gtctaagtat	1	TC10508	49	1	
14023	tgttgattag	1				no_annot
14024	taatttcttc	1				no_annot
14025	tcgggggtgt	1				no_annot
14026	gagcctctct	1				no_annot
14027	aggtgctgag	1				no_annot
14028	cgacatacgt	1	C200665.1	80	0	

14029	cagtgcacaa	1				no_annot
14030	taaaatcact	1				no_annot
14031	gctttggcaa	1				no_annot
14032	acaggttatg	1				no_annot
14033	tttcctcttt	1	TC11733	78	0	
14034	aacacattgg	1				no_annot
14035	gatcccgaca	1				no_annot
14036	gattatttat	1				no_annot
14037	gtcgctaata	1				no_annot
14038	gaaaacaccc	1	CD083251	27	1	
14039	tgtcccaaaa	1	C205737.1	86	1	
14040	tgtaactatg	1				no_annot
14041	ttcaacttca	1	TC10556	99	0	RTNL [Schistosoma mansoni]
14042	atgaagccca	1	C604761.1	23	1	
14043	cattagtatg	1	AA528900	89	0	
14043	cattagtatg	1	CD131978	71	0	
14043	cattagtatg	1	BF936473	50	0	NADH-ubiquinone oxidoreductase
14044	ctttatcagt	1	TC11137	69	0	
14044	ctttatcagt	1	TC11138	24	0	
14044	ctttatcagt	1	TC11139	66	0	
14044	ctttatcagt	1	CD080036	76	0	
14045	cttgtaagtt	1				no_annot
14046	gccacatcca	1				no_annot
14047	tctattggtt	1				no_annot
14048	gcacacaatt	1				no_annot
14049	cgggaatcta	1	C605481.1	6	2	
14050	tcaattgaac	1				no_annot
14051	gttgaaagaa	1	TC10507	67	3	GP|29894281|gb|AAP07572.1||AE016999 Internalin protein {Bacillus cereus ATCC 14579}, partial (3%)
14052	gaaatcctac	1				no_annot
14053	tcggcgaaca	1				no_annot

14054	gtgcatttga	1				no_annot
14055	ttggaataag	1				no_annot
14056	tataattaat	1				no_annot
14057	tgcattactt	1	AI975604	74	1	
14058	catcatcaat	1	TC14264	43	3	
14059	tcaacagact	1	TC7595	26	6	similar to GP|2052191|emb|CAB06295.1||Z83869 serine/threonine kinase {Rattus norvegicus}, partial (41%)
14060	gatacgagtt	1	TC18892	4	1	
14061	taactgacag	1				no_annot
14062	tccaccactg	1				no_annot
14063	taagcgcgtt	1				no_annot
14064	ttaaattgtt	1				no_annot
14065	gccttaaaga	1				no_annot
14066	gaagtcagta	1				no_annot
14067	tgtgcttcca	1				no_annot
14068	attataaaac	1				no_annot
14069	attataaaaa	1				no_annot
14070	aggagaggtt	1				no_annot
14071	gtcgttttat	1	CD090700	7	1	
14072	tgttgtggtg	1				no_annot
14073	tgacttctat	1	TC8227	48	0	
14073	tgacttctat	1	CD110896	52	1	
14074	tgacagcaaa	1				no_annot
14075	gttcgttgct	1				no_annot
14076	ataccgcgtc	1				no_annot
14077	tccaaaggtt	1				no_annot
14078	gaaacacttt	1	TC12684	65	0	
14078	gaaacacttt	1	TC16959	13	3	weakly similar to GP|20977028|gb|AAM33247.1||AF419152 mitotic phosphoprotein 39 {Xenopus laevis}, partial (18%)

14079	gtttttcttc	1	TC10353	53	1	GP|160925|gb|AAA29853.1||M94346 A.1.12/9 antigen {Schistosoma mansoni}, partial (86%)
14080	acgaaaggat	1				no_annot
14081	gtgaacgcaa	1	TC16127	2	3	
14082	ctctgtttgt	1				no_annot
14083	tgtcgtgttg	1	C206603.1	46	2	
14083	tgtcgtgttg	1	C600102.1	46	2	
14084	tttacttttc	1				no_annot
14085	gtctttatat	1	TC17348	83	0	similar to SP|P27924|UBC1_HUMAN Ubiquitin-conjugating enzyme E2-25 kDa(Ubiquitin-protein ligase) (Ubiquitin carrier protein) (Huntingtin interacting protein 2) (HIP-2). [Bovine] {Bos taurus}, partial (52%)
14086	tgtatgtccg	1				no_annot
14087	tttactttta	1				no_annot
14088	aagagacgga	1				no_annot
14089	acctgcgtgg	1	CD067038	70	0	
14090	gttgattctt	1	C606483.1	60	1	
14091	tacaatagat	1				no_annot
14092	ttgtgattta	1				no_annot
14093	gtataataat	1				no_annot
14094	aaattattga	1	TC14821	73	0	
14094	aaattattga	1	CD088910	20	2	similar to GP|5597017|gb| reverse transcriptase {synthetic construct}, partial (1%)
14095	tcagtggaca	1				no_annot
14096	agaggataat	1				no_annot
14097	attgggtgat	1				no_annot
14098	caacaatttt	1	TC12476	10	0	homologue to GP|2842525|emb|CAA16838.1||AL021746 serine rich protein {Schizosaccharomyces pombe}, partial (5%)
14098	caacaatttt	1	TC15594	88	0	
14099	tttaccttac	1				no_annot

14100	attccgaggg	1	TC17134	61	0	weakly similar to GP|4091774|gb|AAC99294.1||AF051321 Sam68-like phosphotyrosine protein alpha {Homo sapiens}, partial (24%)
14101	ttcattaaat	1	TC7178	92	0	
14102	caattatcaa	1				no_annot
14103	tacctgggta	1				no_annot
14104	ttaaataggt	1				no_annot
14105	cccaccactc	1				no_annot
14106	attagcattg	1				no_annot
14107	caacagagta	1				no_annot
14108	caacgttgat	1				no_annot
14109	tagtatctgt	1	TC10556	70	3	RTNL [Schistosoma mansoni]
14110	ttgagtagca	1	C201152.1	83	0	
14110	ttgagtagca	1	C602947.1	78	1	
14111	taactaaaat	1				no_annot
14112	tacgtacgtg	1				no_annot
14113	tctacgtctc	1				no_annot
14114	tgttaacggt	1	C206674.1	29	1	
14114	tgttaacggt	1	C609157.1	12	6	
14115	gatttgattc	1	TC17942	17	0	
14115	gatttgattc	1	TC18041	89	1	
14116	ttctgacagt	1				no_annot
14117	aagtacggac	1				no_annot
14118	agtaaggata	1				no_annot
14119	gccccttggg	1				no_annot
14120	caatgagctg	1	TC10570	45	3	
14121	ggaatgatga	1				no_annot
14122	tacggctttt	1				no_annot
14123	gtgatttgtt	1				no_annot
14124	gaaggttcca	1				no_annot
14125	tcaagaagtt	1				no_annot

14126	cttcaaccga	1	TC10213	43	1	
14127	agatcatcca	1				no_annot
14128	aaaggcgatg	1				no_annot
14129	gaatgatgct	1				no_annot
14130	taaacgattt	1				no_annot
14131	ctgtgatttt	1				no_annot
14132	gagattcaat	1				no_annot
14133	acacaccata	1				no_annot
14134	tacagatact	1				no_annot
14135	ttgttttgta	1				no_annot
14136	tgtaagtgtg	1	C714921.1	61	1	
14137	tatgccaaaa	1				no_annot
14138	tgatggcggt	1				no_annot
14139	gggaacgtat	1				no_annot
14140	tcaattcata	1				no_annot
14141	aacgtgttct	1	C202138.1	0	4	
14142	atgaaacaac	1				no_annot
14143	atctatacct	1				no_annot
14144	aaagcatatt	1	C201297.1	12	3	
14144	aaagcatatt	1	C608663.1	10	4	
14145	ctgagtagct	1				no_annot
14146	ggtatcattt	1				no_annot
14147	gccgacaacc	1				no_annot
14148	aataaatggt	1	CD153947	78	1	
14149	aacgtgttca	1	TC12519	21	0	similar to GP|29824887|gb|AAO92025.1||AY253340 UDP-Gal:beta-GlcNAc beta-1,3-galactosyltransferase 3; B3GALT3 {Sus scrofa}, partial (5%)
14150	ctcaatatga	1				no_annot
14151	gtatgcaact	1				no_annot
14152	ttgtaccctt	1				no_annot
14153	atacttctca	1				no_annot

14154	ccacaaccgc	1				no_annot
14155	tgatgtcatc	1	TC7332	37	2	weakly similar to SP|P05388|RLA0_HUMAN 60S acidic ribosomal protein P0 (L10E). [Human] {Homo sapiens}, partial (65%)
14155	tgatgtcatc	1	CD095991	57	0	similar to GP|3876334|emb|CAB02098.1||Z79754 C. elegans RPA-0 protein (corresponding sequence F25H2.10) {Caenorhabditis elegans}, partial (38%)
14155	tgatgtcatc	1	CD096451	57	0	weakly similar to SP|P47826|RLA0_CHICK 60S acidic ribosomal protein P0 (L10E). [Chicken] {Gallus gallus}, partial (29%)
14156	gaggaagcct	1				no_annot
14157	tatggcgtcg	1	C200935.1	54	1	
14157	tatggcgtcg	1	C606965.1	45	1	
14157	tatggcgtcg	1	C712206.1	54	1	
14158	gcttaagtcc	1				no_annot
14159	cacttcgcaa	1				no_annot
14160	ttgtccacca	1				no_annot
14161	aagctgtaac	1	C201283.1	16	1	
14161	aagctgtaac	1	C605522.1	15	2	
14162	tttacacata	1				no_annot
14163	tagcaataat	1				no_annot
14164	gtgattaggt	1	TC18923	81	0	
14165	gaggaagccc	1				no_annot
14166	ttgtaatagc	1	C201199.1	38	7	
14166	ttgtaatagc	1	C605948.1	1	8	
14166	ttgtaatagc	1	C610885.1	61	1	
14167	aatagagcgt	1				no_annot
14168	tggttgtaat	1	C605029.1	33	4	
14169	ttcacaaaat	1	C205036.1	49	2	
14169	ttcacaaaat	1	C210186.1	91	1	
14169	ttcacaaaat	1	C606030.1	50	3	
14170	attgcttccg	1				no_annot
14171	aaactgatct	1	C202245.1	74	0	

14171	aaactgatct	1	C716667.1	58	0	
14172	gacaactatg	1				no_annot
14173	gtacccaagt	1				no_annot
14174	cactgcattt	1				no_annot
14175	tggttgtaaa	1				no_annot
14176	gtaaaatttt	1				no_annot
14177	caaacgattg	1				no_annot
14178	gtacggatga	1				no_annot
14179	tgtggtggct	1				no_annot
14180	gcctttgctc	1				no_annot
14181	tgtaataatg	1	TC18384	45	0	similar to PIR|PC6507|JC6573 outer-arm dynein light chain 3 - sea urchin (Anthocidaris crassispina), complete
14182	tatgcattcc	1				no_annot
14183	ttattaaact	1				no_annot
14184	aaggattagg	1	C611123.1	67	0	
14185	cgggatgtgt	1				no_annot
14186	ccaatccaag	1				no_annot
14187	attatgtcca	1				no_annot
14188	aaagcaaagg	1				no_annot
14189	aaagaagaag	1				no_annot
14190	ccatatcaat	1	C610587.1	9	2	
14191	catacaccaa	1				no_annot
14192	tttcggtcaa	1				no_annot
14193	tatggcgtaa	1				no_annot
14194	aatagctgtt	1				no_annot
14195	gaaccgcaca	1				no_annot
14196	aaaatcattc	1				no_annot
14197	gttggctgta	1				no_annot
14198	atagtgttgc	1				no_annot
14199	cactaaatct	1	TC17323	32	2	
14200	tttaaatcca	1				no_annot

14201	tcagcgtttc	1				no_annot
14202	tatagacagc	1				no_annot
14203	tgcaccgtca	1				no_annot
14204	ccgctgtctc	1				no_annot
14205	caagataccg	1				no_annot
14206	tttactatca	1				no_annot
14207	gggaccatct	1	TC17015	46	3	
14208	ttataaatgt	1				no_annot
14209	agtatccttt	1				no_annot
14210	atacatacag	1	TC15374	44	1	
14210	atacatacag	1	CD089280	0	0	weakly similar to GP|7302046|gb|AAF57148.1||AE003778 CG1607-PA {Drosophila melanogaster}, partial (19%)
14211	tagaacacca	1				no_annot
14212	tcaaacctcc	1	TC14382	21	0	weakly similar to SP|P78381|UGAT_HUMAN UDP-galactose translocator (UDP-galactose transporter) (UGT) (UDP-Gal- Tr). [Human] {Homo sapiens}, partial (26%)
14213	atgtatgttg	1	CD068517	9	3	
14214	tggtccatca	1				no_annot
14215	tttgtatgtt	1				no_annot
14216	tacttaggcc	1				no_annot
14217	gtaacggtgg	1				no_annot
14218	gagagtacgt	1	CD076377	7	2	
14219	tatgcattac	1				no_annot
14220	atttgttatt	1				no_annot
14221	ggctccactg	1	TC10364	81	0	weakly similar to GP|1575609|gb|AAC50893.1||U69127 FUSE binding protein 3 {Homo sapiens}, partial (6%)
14222	gaaaaaagac	1	TC14424	70	0	unknown
14223	agtgctttca	1				no_annot
14224	gtcgcctacg	1	TC18452	7	1	
14225	gatacaatat	1				no_annot

14226	tgccttcctt	1	TC14291	67	0	weakly similar to SP|Q92503|S141_HUMAN SEC14-like protein 1. [Human] {Homo sapiens}, partial (3%)
14226	tgccttcctt	1	BG931048	52	0	similar to GP|21391472|gb vitellogenin 1 {Danio rerio}, partial (1%)
14227	tgggtgcggg	1				no_annot
14228	agaaaatata	1	TC11833	90	0	weakly similar to GP|2462488|emb|CAA63694.1||X93207 NRD2 convertase {Homo sapiens}, partial (3%)
14229	taagcccagt	1				no_annot
14230	gtgcctgcct	1				no_annot
14231	aagatgtgta	1				no_annot
14232	agttctatcg	1				no_annot
14233	ttgggattat	1	AI976217	39	2	weakly similar to GP|20151533|gb|AAM11126.1||AY094773 GM14501p {Drosophila melanogaster}, partial (23%)
14234	caacaaatcc	1				no_annot
14235	agtacgaata	1				no_annot
14236	cacttatagc	1				no_annot
14237	ataacattct	1				no_annot
14238	tgttggtgaa	1				no_annot
14239	agtggaagca	1	C200386.1	88	0	
14239	agtggaagca	1	C307653.1	57	1	
14239	agtggaagca	1	C612175.1	89	0	
14240	ttgggattac	1				no_annot
14241	gtgcaacaac	1	TC17440	41	5	
14242	gcacgcattc	1				no_annot
14243	tttactatac	1				no_annot
14244	tgaaccggat	1				no_annot
14245	gatcatacgt	1	TC13327	6	1	weakly similar to GP|16197849|gb|AAL13568.1||AY058339 GH11341p {Drosophila melanogaster}, partial (7%)
14246	ttaccttgca	1				no_annot
14247	gtctcgagga	1				no_annot
14248	ctaaaaccct	1				no_annot

14249	agaatacctg	1				no_annot
14250	gaggatagtt	1				no_annot
14251	accaatcgag	1	TC18061	72	0	
14252	tcgtcagtat	1				no_annot
14253	tattttttgt	1	C205786.1	24	0	
14253	tattttttgt	1	C603902.1	38	0	
14254	gatcaatggg	1				no_annot
14255	cttttggaga	1	C209706.1	58	1	
14255	cttttggaga	1	C603073.1	65	2	
14256	attaacaaca	1				no_annot
14257	gtggtgagga	1	C608226.1	7	1	
14258	taagctgaac	1				no_annot
14259	acgaatgccg	1				no_annot
14260	tgcatttctt	1				no_annot
14261	atctgtagtg	1				no_annot
14262	tctttcgatt	1				no_annot
14263	cgggtgcgga	1				no_annot
14264	aagggtatac	1				no_annot
14265	atcccagcca	1				no_annot
14266	tcgaaggtta	1				no_annot
14267	ttcatttact	1				no_annot
14268	ttactgtatt	1				no_annot
14269	ggaggtactc	1				no_annot
14270	gacatataca	1				no_annot
14271	ctgatatatt	1	TC8866	93	0	
14272	tttgcaactc	1				no_annot
14273	tacatcaatt	1	TC10199	88	0	weakly similar to GP|3043760|dbj|BAA25544.1||AB012759 rPOP {Rattus norvegicus}, partial (15%)
14274	ccagttactt	1				no_annot

14275	gtggtcaaac	1	TC17059	33	2	weakly similar to GP|14198122|gb|AAH08121.1||BC008121 AI504353 protein {Mus musculus}, partial (10%)
14276	aacttgttgg	1	TC18694	12	4	
14277	ggcttcagcc	1	TC14006	86	0	
14278	tgattcgtat	1	C612391.1	22	4	
14279	aatagagcaa	1				no_annot
14280	aacaatgaat	1	CD158574	76	0	
14281	gacaacaaca	1				no_annot
14282	gtgaggtgtt	1				no_annot
14283	ctgtgaataa	1				no_annot
14284	gcggtggtag	1				no_annot
14285	ggattccaat	1	CD094906	45	0	weakly similar to SP|P23232|GBB_LOLFO Guanine nucleotide-binding protein beta subunit. [Northern European squid] {Loligo forbesi}, partial (23%)
14286	tgatctccct	1				no_annot
14287	caagctgaag	1	TC11845	93	0	
14288	aaggattaac	1	C604350.1	38	0	
14289	gtaaactata	1				no_annot
14290	gctgcattaa	1				no_annot
14291	acgactacga	1				no_annot
14292	gatgttgttt	1				no_annot
14293	aactcagtta	1				no_annot
14294	tgaacctaaa	1				no_annot
14295	tatagacaat	1				no_annot
14296	gacaataatc	1	TC12840	77	0	weakly similar to PIR|T39903|T39903 serine-rich protein - fission yeast (Schizosaccharomyces pombe), partial (5%)
14296	gacaataatc	1	TC16509	84	0	
14297	gtattttgat	1	TC18289	75	0	
14298	tgattgttgt	1				no_annot
14299	ttagaattat	1	CD131636	68	0	weakly similar to SP|Q9Z2W0|DNPE_MOUSE Aspartyl aminopeptidase. [Mouse] {Mus musculus}, partial (11%)

14300	ctaccttgat	1				no_annot
14301	cacgactacc	1				no_annot
14302	actattacta	1				no_annot
14303	gatacattgt	1	TC10754	97	0	similar to GP|20177013|gb|AAM12268.1||AY095175 GH12731p {Drosophila melanogaster}, partial (83%)
14304	ctttctcggt	1	TC8489	73	1	weakly similar to GP|23574715|dbj|BAC20586.1||AB083307 mitochondrial carnitine/acylcarnitine carrier protein {Macaca fascicularis}, partial (52%)
14305	atcactgttc	1	C203140.1	74	3	
14305	atcactgttc	1	C611085.1	74	3	
14306	taacaattgc	1				no_annot
14307	atgagccagc	1				no_annot
14308	attagcttta	1				no_annot
14309	gattttcgat	1				no_annot
14310	gatcaatgct	1				no_annot
14311	cgtaagagag	1				no_annot
14312	tcttgtttaa	1				no_annot
14313	taagctcatt	1	TC17922	27	0	weakly similar to GP|6492276|gb|AAF14249.1|AF110233_1|AF110233 clathrin-associated adaptor complex AP-3 medium chain {Drosophila melanogaster}, partial (37%)
14313	taagctcatt	1	CD129879	58	0	homologue to SP|Q15631|TSN_HUMAN Translin. [Human] {Homo sapiens}, partial (25%)
14314	tagtgtacat	1	TC11827	15	3	weakly similar to PIR|T31666|T31666 natriuretic peptide receptor homolog COS1.2a - sea squirt (Ciona intestinalis), partial (12%)
14315	tatctatgtt	1				no_annot
14316	gacagagata	1				no_annot
14317	tattttttct	1	TC14690	77	1	
14318	tgcacgttct	1				no_annot
14319	gtttttctgg	1				no_annot
14320	atgagtgaat	1				no_annot
14321	gtaacggtag	1	TC9343	26	1	homologue to GP|6114870|dbj|BAA85700.1||AB026381 ORF1 {TT virus}, partial (5%)

14322	ttcacatact	1				no_annot
14323	taaacgtttt	1				no_annot
14324	cacactctct	1				no_annot
14325	cttttctctt	1	TC10799	83	0	weakly similar to GP|4138422|emb|CAA04022.1||AJ000347 3'(2'),5'-bisphosphate nucleotidase {Rattus norvegicus}, partial (38%)
14326	ttatacaaga	1				no_annot
14327	tgatcagttg	1				no_annot
14328	tgtccgtgaa	1				no_annot
14329	ttcattcgtt	1				no_annot
14330	caatatacgt	1				no_annot
14331	atccataagg	1				no_annot
14332	gatatacaga	1				no_annot
14333	cccaaaaaaa	1				no_annot
14334	tactgctttg	1				no_annot
14335	cactataata	1	TC17170	61	2	similar to EGAD|66062|69017 ras-related protein RAL-A {Drosophila melanogaster}, partial (30%)
14336	gtgttaatcc	1				no_annot
14337	aagtgcttac	1				no_annot
14338	tatctcacac	1	CD079994	11	3	
14339	tttattcaga	1	CD078809	53	0	
14340	gcttcgtgtg	1				no_annot
14341	tcaactttgt	1				no_annot
14342	ttgtcctcct	1				no_annot
14343	atggatgtag	1	TC7110	30	4	similar to GP|6715146|gb|AAF26302.1|AF184617_1|AF184617 proprotein convertase aPC6C isoform {Branchiostoma californiense}, partial (3%)
14343	atggatgtag	1	CD155921	0	6	
14343	atggatgtag	1	CD179891	63	0	similar to PIR|T02995|T02995 unspecific monooxygenase- common tobacco, partial (4%)
14343	atggatgtag	1	CD201546	86	0	similar to PIR|T02995|T02995 unspecific monooxygenase- common tobacco, partial (20%)

14344	tccgtctatc	1				no_annot
14345	gtaaccagtg	1				no_annot
14346	cccagatttt	1	TC17117	41	0	similar to GP|32709|emb|CAA44450.1||X62570 IFP53 {Homo sapiens}, partial (73%)
14347	cggttactca	1				no_annot
14348	tgattccttt	1				no_annot
14349	aacaatcatt	1	CD150233	69	2	
14350	ccttgtttac	1				no_annot
14351	tgtcaccaac	1	CD075782	51	1	homologue to GP|6503027|gb|A F-box protein 26 {Xenopus laevis}, partial (1%)
14352	tgtagcatat	1				no_annot
14353	tgcacaacgg	1	TC10324	22	1	
14353	tgcacaacgg	1	CD179666	33	1	
14354	agtctttgtt	1				no_annot
14355	taggaaaatg	1				no_annot
14356	gcgtgtacct	1				no_annot
14357	attaaattat	1				no_annot
14358	tatctaaggg	1	AI067495	39	1	homologue to GP|20515976|gb| predicted membrane-associated HD superfamily hydrolase {Thermoanaerobacter tengcongensis}, partial (1%)
14359	gtcatagtaa	1				no_annot
14360	gttcataatg	1	TC9987	68	0	
14361	agtctttgtg	1	TC16985	33	0	similar to GP|10581292|gb|AAG20052.1||AE005085 Vng1846c {Halobacterium sp. NRC-1}, partial (6%)
14362	attgtcatat	1	TC14339	80	1	
14363	gttcataatc	1				no_annot
14364	catctatgtc	1				no_annot
14365	ttcacataaa	1	C200444.1	10	4	
14365	ttcacataaa	1	C607208.1	12	4	
14366	gtgttaatag	1	C202255.1	13	1	
14366	gtgttaatag	1	C606840.1	16	1	
14367	aagaatatta	1				no_annot

14368	tatttgccat	1	C202173.1	27	2	
14368	tatttgccat	1	C611269.1	14	5	
14369	tatgcgtgca	1				no_annot
14370	ttcaaacaga	1				no_annot
14371	gttacgtttt	1	TC8179	24	1	
14372	ttagacggaa	1				no_annot
14373	aattccactg	1				no_annot
14374	taataatgca	1	TC7813	72	0	weakly similar to GP|22859164|emb|CAD30680.1||AJ459409 RNA-binding protein S1 {Drosophila melanogaster}, partial (7%)
14375	aaacgtcaga	1				no_annot
14376	ttacagtttc	1	TC13667	5	7	weakly similar to SP|Q92900|RNT1_HUMAN Regulator of nonsense transcripts 1 (Nonsense mRNA reducing factor 1) (NORF1) (Up-frameshift suppressor 1 homolog). [Human] {Homo sapiens}, partial (3%)
14376	ttacagtttc	1	AW053297	51	0	similar to GP|24636502|dbj|BAC22917.1||AB084520 azetidine-2-carboxylic acid acetyltransferase {Saccharomyces paradoxus}, partial (6%)
14377	tcactcaata	1				no_annot
14378	gaaaagcaaa	1				no_annot
14379	ttgaaaatga	1	TC19461	30	1	
14380	attattgccg	1				no_annot
14381	taaatatgtc	1	TC10618	1	8	weakly similar to SP|O14638|NPP3_HUMAN Ectonucleotide pyrophosphatase/phosphodiesterase 3 (E-NPP 3) (Phosphodiesterase I/nucleotide pyrophosphatase 3) (Phosphodiesterase I beta) (PD-Ibeta) (CD203c antigen) [Includes: Alkaline phosphodiesterase I; Nucleotide pyrophosphatase(NPPase)]. [Human] {Homo sapiens}, partial (6%)
14382	gttatgcatt	1				no_annot
14383	aagcccttct	1				no_annot
14384	gtattcattg	1				no_annot
14385	ttttctcgaa	1				no_annot
14386	ccgcaaatgc	1	TC17551	43	2	similar to SP|P13716|HEM2_HUMAN Delta-aminolevulinic acid dehydratase(Porphobilinogen synthase) (ALADH). [Human] {Homo sapiens}, partial (40%)

14387	gctccgatgg	1				no_annot
14388	atattgttgt	1				no_annot
14389	ctgcagtagt	1				no_annot
14390	cacgtacgga	1				no_annot
14391	ctgttttggt	1	CD075658	92	0	homologue to PIR|H72173|H72173 D5L protein - variola minor virus (strain Garcia-1966), partial (12%)
14392	tttgtctccg	1				no_annot
14393	gctgaactat	1				no_annot
14394	agcaaagtga	1				no_annot
14395	attcgaaccc	1				no_annot
14396	tataccattt	1				no_annot
14397	cgtaagtggt	1				no_annot
14398	caggcggatg	1				no_annot
14399	gaataaaatt	1	AY227022|Sma.285	9	13	SINA (SINA)
14400	gcgtgatgat	1	TC17216	7	2	weakly similar to GP|2804786|gb|AAC39576.1||AF040991 roundabout 2 {Homo sapiens}, partial (8%)
14400	gcgtgatgat	1	CD081014	51	2	
14401	tacataatgt	1				no_annot
14402	gcacacaaat	1	TC9558	22	3	
14403	tctattggat	1				no_annot
14404	caccagatcc	1				no_annot
14405	catatgaagg	1				no_annot
14406	gtaaaattgt	1				no_annot
14407	acagtggcgt	1				no_annot
14408	cttcattgag	1				no_annot
14409	ataactaagg	1				no_annot
14410	cccaaccctt	1	CD155798	96	0	
14410	cccaaccctt	1	CD073947	57	1	
14411	atgagccaat	1	TC7069	24	1	

14412	agccgagctg	1	TC7048	66	3	weakly similar to GP|27497118|gb|AAO17319.1|AF465616_1|AF465616 death inducer with SAP domain DIS {Homo sapiens}, partial (6%)
14413	tatggtgtga	1	C206072.1	80	0	
14413	tatggtgtga	1	C602736.1	4	2	
14414	atgggggaac	1				no_annot
14415	acctaccacc	1				no_annot
14416	ttttcgatgc	1	TC12139	67	0	weakly similar to SP|P49588|SYA_HUMAN Alanyl-tRNA synthetase(Alanine--tRNA ligase) (AlaRS). [Human] {Homo sapiens}, partial (19%)
14417	cccaccaccc	1				no_annot
14418	gtgactacga	1				no_annot
14419	gttacgatgg	1				no_annot
14420	ttattataag	1				no_annot
14421	tgttatccat	1				no_annot
14422	cattcgagca	1				no_annot
14423	tattcattac	1				no_annot
14424	attagcatca	1	TC9306	39	1	
14424	attagcatca	1	CD088511	44	0	
14425	atgataactt	1				no_annot
14426	gttggctggg	1				no_annot
14427	catcagcttg	1				no_annot
14428	taatcactat	1	C202194.1	54	1	
14428	taatcactat	1	C600259.1	61	1	
14429	cctaggaaca	1				no_annot
14430	acgatcattg	1	AW018281	34	0	
14431	aattgaattc	1				no_annot
14432	tttctatttt	1	TC12092	93	0	weakly similar to GP|5281316|gb|AAD41476.1|AF133124_1|AF133124 transcription factor IIIC63 {Homo sapiens}, partial (8%)
14432	tttctatttt	1	TC14140	62	1	
14433	attcatcatc	1				no_annot

14434	tgcaaacctg	1	TC10820	0	0	weakly similar to GP|16323051|gb|AAL15260.1||AY057629 At2g22720/T9I22.16 {Arabidopsis thaliana}, partial (8%)
14435	agaagaatga	1	AY838777|Sma.5389	36	1	Clone F04 Perere 3 retrotransposon mRNA, partial sequence
14435	agaagaatga	1	AY838778|Sma.1285	36	2	Clone 124B5 unknown mRNA
14435	agaagaatga	1	AY838776|Sma.5388	26	3	Clone C09 Perere 3 retrotransposon mRNA, partial sequence
14436	cccaagtcct	1	C210345.1	38	0	
14437	ggttaacata	1	TC7763	78	0	
14438	tgttgttact	1	C201331.1	71	0	
14439	aagaggaatc	1				no_annot
14440	tagcgttatg	1				no_annot
14441	tgcagaggta	1				no_annot
14442	cgaagaacac	1				no_annot
14443	atgttactaa	1	TC16350	52	0	similar to GP|24061781|gb|AAN39843.1||AY147933 UDP-glucuronic acid decarboxylase {Caenorhabditis elegans}, partial (31%)
14444	ctagagtgga	1				no_annot
14445	aatttcagca	1				no_annot
14446	agctatttaa	1				no_annot
14447	tataatcgaa	1				no_annot
14448	gaaaggctga	1				no_annot
14449	tggcgaattt	1				no_annot
14450	agctgccata	1				no_annot
14451	ctagcagcat	1				no_annot
14452	cgcattacat	1				no_annot
14453	acacgtgtgc	1	C602269.1	71	0	
14454	aatcagaatt	1				no_annot
14455	ccccggtaca	1				no_annot
14456	tcaactttat	1				no_annot
14457	tttgtatggg	1	C608072.1	26	1	
14458	gaaccttcag	1				no_annot

14459	ctttaggtaa	1				no_annot
14460	acaaacggag	1				no_annot
14461	tgtgtttttt	1				no_annot
14462	gtggtttata	1				no_annot
14463	tattgccttt	1				no_annot
14464	gtttaatgaa	1	TC14659	11	0	similar to GP|10436078|gb|AAG16781.1|AF231511_1|AF231511 RNA binding motif protein 8A {Homo sapiens}, partial (73%)
14464	gtttaatgaa	1	TC14660	16	0	weakly similar to GP|7303943|gb|AAF58987.1||AE003834 CG8781-PA {Drosophila melanogaster}, partial (86%)
14465	atctgctgat	1				no_annot
14466	gtatggtgat	1				no_annot
14467	tactactact	1				no_annot
14468	gacaacggaa	1				no_annot
14469	tgagagcatt	1	CD065018	72	0	
14469	tgagagcatt	1	CD093869	0	2	
14469	tgagagcatt	1	AI014201	63	1	
14470	ggttggaaga	1				no_annot
14471	aatcttattg	1				no_annot
14472	cataatcgat	1				no_annot
14473	cgcaacagac	1				no_annot
14474	cttgactgtg	1				no_annot
14475	gcttgcgtag	1				no_annot
14476	tttgaagcca	1				no_annot
14477	gtcatcgaaa	1				no_annot
14478	caaacaacta	1				no_annot
14479	catttacaaa	1				no_annot
14480	tgttttattg	1				no_annot
14481	cttactttaa	1				no_annot
14482	ttgaaaatat	1	TC11578	67	1	

14483	cctccacacc	1				no_annot
14484	agcatacata	1				no_annot
14485	atgatgataa	1	TC13919	8	1	
14485	atgatgataa	1	TC13961	54	4	homologue to SP|P36417|GBF_DICDI G-box binding factor (GBF). [Slime mold] {Dictyostelium discoideum}, partial (4%)
14486	tacatctatg	1	TC15089	5	2	
14487	tcgcccagtg	1				no_annot
14488	atagaggaat	1	TC17031	41	0	weakly similar to GP|15559219|gb|AAK83217.2|AF335277_1|AF335277 centrosomal protein centrin 3 {Rattus norvegicus}, partial (92%)
14489	tacataatca	1				no_annot
14490	tctgtccata	1	AA140578	20	0	
14491	aattaaaagt	1				no_annot
14492	tattaactct	1	TC13903	35	2	weakly similar to SP|Q9JID6|LCFA_CAVPO Long-chain-fatty-acid--CoA ligase 1(Long-chain acyl-CoA synthetase 1) (LACS 1) (Palmitoyl-CoA ligase). [Guinea pig] {Cavia porcellus}, partial (12%)
14493	aaaagttacc	1				no_annot
14494	ggtgatttat	1				no_annot
14495	taccagttga	1	TC18893	35	3	similar to GP|10187175|emb|CAC09061.1||AX025502 cDNA~Mango esterase {Mangifera indica}, partial (23%)
14496	gacaattatg	1	C311758.1	71	0	
14496	gacaattatg	1	C609710.1	42	0	
14496	gacaattatg	1	C611003.1	28	5	
14497	ggcctttttt	1				no_annot
14498	gttggctgcg	1				no_annot
14499	cattctggtt	1				no_annot
14500	tacctgtaag	1				no_annot
14501	aagagcgatc	1	TC10418	57	0	
14501	aagagcgatc	1	TC10429	87	0	
14501	aagagcgatc	1	CD187418	81	0	
14501	aagagcgatc	1	CD136152	79	0	

14502	tgttgtcgta	1				no_annot
14503	atgatcgtcg	1				no_annot
14504	cgtaagtgat	1	TC11997	35	2	
14505	tcagcgttcc	1				no_annot
14506	ggcctttttc	1	CD169381	0	0	
14507	attaataaga	1				no_annot
14508	ctatacgggg	1				no_annot
14509	gaagaaaaca	1	TC7371	23	1	
14510	tgccattacg	1				no_annot
14511	agtcaataaa	1	TC10431	67	0	GP|2226370|gb|AAC50020.1||AF005359 RNA-binding protein {Nicotiana glutinosa}, partial (10%)
14512	ttgcgcgtaa	1				no_annot
14513	gcctatggtt	1				no_annot
14514	cgttttattg	1				no_annot
14515	tgtatacgcc	1				no_annot
14516	acagtggcat	1				no_annot
14517	catatgaaaa	1	L40328|Sma.796	42	5	Ca2+ transport ATPase (SMA1)
14518	cacatctatg	1	TC7756	84	1	similar to SP|O14807|RASM_HUMAN Ras-related protein M-Ras (Ras-related protein R-Ras3). [Human] {Homo sapiens}, partial (75%)
14519	tcgctatacg	1				no_annot
14520	tcaacacaat	1				no_annot
14521	ataactaaaa	1				no_annot
14522	taaactgttt	1	CD152930	62	0	
14523	ctttcaacta	1	AI740356	39	1	
14523	ctttcaacta	1	BG931072	53	0	
14523	ctttcaacta	1	CD201235	33	1	
14523	ctttcaacta	1	CD076310	88	0	
14523	ctttcaacta	1	CD079263	53	1	
14524	agtacagtag	1	AI394831	10	2	
14525	ggaaaaagtg	1				no_annot
14526	ttacagataa	1				no_annot

14527	ggcactcact	1	TC10485	67	2	SP|P53442|ALF_SCHMA Fructose-bisphosphate aldolase. [Blood fluke] {Schistosoma mansoni}, complete
14528	tggatcctta	1				no_annot
14529	aatagttgtg	1				no_annot
14530	tgtagcttcc	1				no_annot
14531	taaactgtta	1				no_annot
14532	tataacgtgc	1				no_annot
14533	agccggcttc	1	CD171182	23	0	
14534	tgttcaattc	1	TC11113	46	0	weakly similar to SP|P82650|RT22_HUMAN Mitochondrial 28S ribosomal protein S22 (S22mt) (MRP-S22) (GK002). [Human] {Homo sapiens}, partial (11%)
14535	tttgacagct	1	TC13280	61	2	similar to GP|21627500|gb|AAF58723.2||AE003827 CG17326-PA {Drosophila melanogaster}, partial (5%)
14536	tgttcaatta	1	TC7691	78	0	weakly similar to GP|14280321|gb|AAK57547.1||AY033553 F-box protein FBW7 {Homo sapiens}, partial (23%)
14537	acaaataaga	1				no_annot
14538	atccgacatc	1				no_annot
14539	tgtagagaat	1				no_annot
14540	aaaatcatag	1	C600599.1	27	2	
14541	tgtgtacatc	1	TC9070	83	0	
14542	tgtgtacata	1	AF510339|Sma.805	90	1	Elastase 2a (CE-2a)
14542	tgtgtacata	1	gi|1103828|gb|U31768.1|SMU31768	92	1	Schistosoma mansoni elastase gene, 3045 bp clone, complete cds
14542	tgtgtacata	1	gi|160947|gb|J03946.1|SCMCPROT	96	0	S.mansoni cercarial protease mRNA, complete cds
14543	tccccccccc	1				no_annot
14544	tcttagatgg	1				no_annot
14545	gataaactac	1	C304969.1	0	1	
14546	gttggctgaa	1	C200405.1	59	0	
14546	gttggctgaa	1	C611449.1	25	2	
14547	aaatatatat	1	TC7863	8	2	

14548	gggttggagt	1	TC13970	61	0	weakly similar to GP|20905799|gb|AAM31026.1||AE013365 Chemotaxis protein CheW {Methanosarcina mazei Goe1}, partial (13%)
14549	cccacaagct	1				no_annot
14550	gaagaaaaac	1				no_annot
14551	gcaggttgtg	1				no_annot
14552	gagggatagt	1				no_annot
14553	tttctaatcg	1	TC13919	0	3	
14553	tttctaatcg	1	CD073930	22	1	
14554	ctagagtgaa	1				no_annot
14555	aacagcctag	1				no_annot
14556	ggtctcctgc	1				no_annot
14557	aaatatatag	1				no_annot
14558	ccatcaacta	1				no_annot
14559	tctaatttat	1				no_annot
14560	ttgacgcttt	1	C301392.1	94	0	
14560	ttgacgcttt	1	C610205.1	94	0	
14561	gataatgaat	1	TC7428	48	0	weakly similar to SP|Q27450|CYP1_BRUMA Peptidylprolyl isomerase CYP-1(Peptidylprolyl cis-trans isomerase) (Cyclophilin) (PPIase). {Brugia malayi}, partial (19%)
14561	gataatgaat	1	TC8430	29	0	similar to GP|16769316|gb|AAL28877.1||AY061329 LD25651p {Drosophila melanogaster}, partial (4%)
14562	gtggcaaagg	1	TC16902	68	3	similar to GP|22946759|gb|AAF53680.2||AE003659 CG10333-PA {Drosophila melanogaster}, partial (59%)
14563	ctaagtcctg	1				no_annot
14564	aaagggggag	1				no_annot
14565	gaaacccaac	1				no_annot
14566	cggcaaaagc	1				no_annot
14567	gataatgaag	1				no_annot
14568	catattgatt	1				no_annot
14569	gtgtatattt	1	TC13603	19	0	
14570	aagaattttt	1	TC14206	85	0	

14571	aagaaagaag	1	TC10362	51	1	homologue to GP|2829289|gb|AAC00519.1||AF044412 HSP70 {Schistosoma japonicum}, complete
14572	gtatcttcaa	1	AF030968|Sma.776	21	4	Clone 9 unknown
14573	tatcttacga	1				no_annot
14574	atgatgttgg	1	TC19422	81	0	
14575	aatggaatac	1				no_annot
14576	tgaactggat	1	AI977486	5	1	weakly similar to SP|Q13616|CUL1_HUMAN Cullin homolog 1 (CUL-1). [Human] {Homo sapiens}, partial (9%)
14577	gaaggatttg	1				no_annot
14578	atgcttagtg	1				no_annot
14579	aatataaatg	1	C210026.1	18	1	
14579	aatataaatg	1	C600599.1	5	4	
14580	ctctacgtca	1				no_annot
14581	aatataaatc	1				no_annot
14582	gtaagcatca	1	TC14045	67	0	weakly similar to SP|P08621|RU17_HUMAN U1 small nuclear ribonucleoprotein 70 kDa (U1 snRNP 70 kDa) (snRNP70) (U1-70K). [Human] {Homo sapiens}, partial (17%)
14583	acatagaatt	1				no_annot
14584	aaaacaatta	1				no_annot
14585	aattacgccc	1				no_annot
14586	agaacaagct	1				no_annot
14587	tgaagcctag	1				no_annot
14588	gttcactaac	1				no_annot
14589	catcgaaata	1				no_annot
14590	taaatgcaga	1	TC14314	1	2	
14591	ttcgttgaat	1	TC11130	2	9	
14592	tcatccgtca	1				no_annot
14593	tggatgttaa	1				no_annot
14594	ttaggcttat	1				no_annot
14595	gctggatcgg	1				no_annot
14596	tggccggaca	1				no_annot

14597	taacccagac	1				no_annot
14598	ggttatttgt	1	TC8898	81	1	similar to SP|Q09028|RBB4_HUMAN Chromatin assembly factor 1 subunit C (CAF-1 subunit C) (Chromatin assembly factor I p48 subunit) (CAF-I 48 kDa subunit) (CAF-Ip48) (Retinoblastoma binding protein p48) (Retinoblastoma-binding protein 4) (RBBP-4) (MSI1 protein homolog). [Human] {Homo sapiens}, partial (91%)
14599	gggtccgtta	1				no_annot
14600	ttatgtattc	1				no_annot
14601	ctaccctttc	1				no_annot
14602	cactgagaga	1				no_annot
14603	cgacgaccag	1				no_annot
14604	taaatctcac	1				no_annot
14605	cactctagta	1				no_annot
14606	tgaagatcac	1				no_annot
14607	tcgcttgcga	1	TC17775	1	0	
14608	ttcgaaacaa	1	TC8169	48	1	homologue to SP|P52798|EFA4_HUMAN Ephrin-A4 precursor (EPH-related receptor tyrosine kinase ligand 4) (LERK-4). [Human] {Homo sapiens}, partial (6%)
14608	ttcgaaacaa	1	CD076480	87	0	
14609	tacatattgt	1				no_annot
14610	tcgacgatgt	1				no_annot
14611	tatgtggatt	1				no_annot
14612	tcaggaggtt	1	TC6942	7	0	
14612	tcaggaggtt	1	TC7259	36	1	
14612	tcaggaggtt	1	TC7266	47	0	
14612	tcaggaggtt	1	TC18745	39	1	similar to GP|28202419|gb|AAO34865.1||AE015936 conserved protein {Clostridium tetani E88}, partial (9%)
14612	tcaggaggtt	1	CD127443	34	1	
14613	gaagtttgtc	1				no_annot
14614	tcttttgata	1				no_annot
14615	aattaaaaaa	1	AF183577|Sma.103	15	6	Alpha 1-3 fucosyltransferase (FucTA)
14616	tgcaattgtt	1				no_annot

14617	cgatcagtgc	1	TC9399	22	5	similar to PIR|H86803|H86803 prophage pi3 protein 59 [imported] - Lactococcus lactis subsp. lactis (strain IL1403), partial (5%)
14618	taagaaattt	1				no_annot
14619	ttaatctata	1				no_annot
14620	ttttcgttgt	1	TC11337	65	1	weakly similar to GP|4105772|gb|AAD02543.1||AF049917 PGPS/D1 {Petunia x hybrida}, partial (24%)
14621	tactgggcaa	1				no_annot
14622	cctttacttt	1				no_annot
14623	gtaaagtgga	1	gi|161025|gb|L02415.1|SCMHSP70X	16	4	Schistosoma mansoni heat shock protein 70 (HSP70) gene, complete cds
14624	tttagatgcg	1				no_annot
14625	atattaacag	1				no_annot
14626	tcaggaggta	1				no_annot
14627	tgtgttatag	1				no_annot
14628	tacattaata	1	TC8149	61	1	
14629	gcgattctat	1				no_annot
14630	aatgaacgtc	1				no_annot
14631	tatcagctgt	1				no_annot
14632	gagccatatg	1				no_annot
14633	atacaaattg	1	TC13661	74	1	similar to GP|13436359|gb|AAH04964.1|AAH04964|BC004964 oxoglutarate dehydrogenase (lipoamide) {Homo sapiens}, partial (70%)
14634	gttatttctt	1	CD154456	17	0	
14635	tacatcaaat	1	TC18369	28	0	
14635	tacatcaaat	1	BG930960	36	1	
14636	taaaacaggt	1	CD192249	59	0	
14637	taaacatctt	1				no_annot
14638	tgggcacaat	1	CD077157	53	0	
14639	tataatgttg	1				no_annot
14640	cttctaataa	1				no_annot

14641	aagttcgata	1	TC13821	87	0	weakly similar to GP|13561418|gb|AAK30301.1|AF289866_1|AF289866 haploid germ cell-specific nuclear protein kinase {Mus musculus}, partial (10%)
14642	ggatcgccaa	1				no_annot
14643	cggctctgga	1				no_annot
14644	tacatcaaac	1	TC8461	1	2	similar to PIR|T40901|T40901 ranbpm homolog - fission yeast (Schizosaccharomyces pombe), partial (4%)
14645	tattgaacag	1	TC19409	40	3	
14646	tgcactcttt	1				no_annot
14647	ctttccgtac	1	C610193.1	46	0	
14648	gcttctaaga	1	C717040.1	35	1	
14649	ttcagtgacg	1	TC7049	19	7	
14650	acagcgagta	1				no_annot
14651	gtggttgggt	1				no_annot
14652	catctgcacc	1				no_annot
14653	ctaataatcg	1	TC9510	51	1	
14654	ttctaccttc	1				no_annot
14655	tactataaaa	1				no_annot
14656	caatattcag	1				no_annot
14657	cacatattgg	1				no_annot
14658	ttcgtccaca	1				no_annot
14659	gagttttcat	1	TC18741	88	1	
14660	tgtcatcacc	1				no_annot
14661	tccaggagat	1				no_annot
14662	tttcacttta	1				no_annot
14663	actttcgctt	1	TC18825	64	1	similar to GP|7292634|gb|AAF48033.1||AE003485 CG18361-PA {Drosophila melanogaster}, partial (8%)
14663	actttcgctt	1	CD080027	93	0	
14664	tggataaccc	1				no_annot
14665	ggtggtgagg	1				no_annot

14666	atggctttag	1	TC8400	0	4	weakly similar to PIR|S65953|S65953 [phosphorylase] phosphatase65K regulatory chain isotype alpha - African clawed frog, partial (25%)
14667	acgtcgacca	1				no_annot
14668	aattgaatgt	1	CD117603	29	0	homologue to PIR|T50773|T507 translation initiation factor homolog p105 [imported] - Arabidopsis thaliana, partial (1%)
14669	aaatcaggaa	1				no_annot
14670	ttattcccaa	1				no_annot
14671	atcactgtaa	1				no_annot
14672	caaatatggg	1				no_annot
14673	ggttaacagt	1				no_annot
14674	atgcagacag	1				no_annot
14675	gggttggaaa	1				no_annot
14676	tttggaaagt	1	TC8795	2	1	
14676	tttggaaagt	1	TC14238	85	1	
14677	atgggaagat	1				no_annot
14678	tgtagtatca	1				no_annot
14679	ctatcagcaa	1				no_annot
14680	tataagttgg	1				no_annot
14681	aatctttttg	1				no_annot
14682	aattcgcatt	1				no_annot
14683	ctgagcttta	1	C606688.1	24	2	
14684	attgttatcg	1				no_annot
14685	gcacgcttgg	1				no_annot
14686	ttcttactgc	1				no_annot
14687	cacatcaaaa	1				no_annot
14688	taggtctgga	1				no_annot
14689	aacaatcacc	1	TC15033	33	3	
14690	tatcttacaa	1				no_annot
14691	ctatactaaa	1	C315977.1	23	1	
14692	ttgttggctg	1				no_annot

14693	ttacaatctg	1				no_annot
14694	gtagccaatg	1				no_annot
14695	tcgtcactaa	1				no_annot
14696	atgatgttaa	1	TC10935	33	5	
14697	tgactgacct	1				no_annot
14698	cgtggtcgag	1				no_annot
14699	tttgctgtca	1	TC14155	33	4	weakly similar to GP|19344076|gb|AAH25628.1||BC025628 expressed sequence AA420417 {Mus musculus}, partial (23%)
14700	aactaatgtt	1	TC11060	80	2	similar to GP|2766687|gb|AAC69992.1||AF036955 tetraspanin TE736 {Schistosoma japonicum}, partial (8%)
14701	aacgttctgt	1				no_annot
14702	ttaaaagatc	1				no_annot
14703	tgtttcttat	1	U07345|Sma.756	73	0	Puerto rican preprocathepsin L (SmCL1)
14704	aaacacaccg	1	C306072.1	54	1	
14704	aaacacaccg	1	C602686.1	58	1	
14705	attcctcgtg	1				no_annot
14706	tgtcatcaac	1				no_annot
14707	ttgtgtaaaa	1				no_annot
14708	aactatactc	1				no_annot
14709	gacaatggga	1				no_annot
14710	tcaggaaagt	1				no_annot
14711	tagacgcgga	1	TC11258	31	1	similar to GP|3646128|emb|CAA09375.1||AJ010841 thioredoxin-like protein {Homo sapiens}, partial (27%)
14712	taaatgcaac	1				no_annot
14713	gtagaagtgg	1	TC10621	15	3	
14714	cacgttccag	1				no_annot
14715	acaagtatgt	1				no_annot
14716	aaggtgaaaa	1	CD061733	68	0	
14716	aaggtgaaaa	1	CD082012	45	4	weakly similar to GP|27355728|dbj|BAC52710.1||AP005962 blr7445 {Bradyrhizobium japonicum USDA 110}, partial (14%)

14717	agctgtgaac	1				no_annot
14718	aagtgacacc	1	CD177807	33	1	similar to GP|23094278|gb|AAF51781.2||AE003595 CG7152-PB {Drosophila melanogaster}, partial (5%)
14719	aaaatcttca	1	TC19747	43	0	
14720	agaagattca	1	BG930359	56	1	similar to GP|6649234|gb|AAF21436.1|AF195529_1|AF195529 14-3-3 epsilon {Schistosoma mansoni}, partial (50%)
14721	gctaatgttc	1				no_annot
14722	tgaatcacta	1	CD096809	17	1	
14723	gaattctggt	1				no_annot
14724	tgatatactt	1	TC16608	76	1	
14725	taggaaaaaa	1				no_annot
14726	caaaaagcag	1				no_annot
14727	tgagattcta	1				no_annot
14728	caggaaaaca	1	AI975497	88	0	
14729	ttacttgagc	1				no_annot
14730	tgttttatgc	1				no_annot
14731	ataacttaag	1				no_annot
14732	tacatattac	1				no_annot
14733	tgatcgaaaa	1				no_annot
14734	ataacttaaa	1				no_annot
14735	atcaactgaa	1				no_annot
14736	gtttgttgtt	1				no_annot
14737	cactatgggc	1				no_annot
14738	tgataatgtc	1				no_annot
14739	gagaaaggtc	1				no_annot
14740	gtagtgcctg	1				no_annot
14741	tttatggtat	1	TC9449	28	1	
14742	ctgattgcct	1				no_annot
14743	ttaattaatt	1	CD146047	45	1	
14744	cggataacac	1				no_annot
14745	aaatcacgtc	1				no_annot

14746	ttggacgatg	1				no_annot
14747	taaatatgac	1	CD093237	28	3	
14747	taaatatgac	1	CD178476	14	2	
14748	tgttcatttg	1	TC7863	51	1	
14749	caaattacca	1	TC17522	13	7	weakly similar to SP|Q9I047|TAL_PSEAE Transaldolase. {Pseudomonas aeruginosa}, partial (73%)
14750	tataaatcta	1				no_annot
14751	tttatggtag	1				no_annot
14752	tatatactgt	1				no_annot
14753	gagccaaaca	1				no_annot
14754	gtggggaaca	1				no_annot
14755	cctttgggag	1				no_annot
14756	aagtgacaac	1				no_annot
14757	tgatgagcta	1				no_annot
14758	gttattaccg	1				no_annot
14759	gtagagaagg	1				no_annot
14760	gcagctcaat	1				no_annot
14761	actgtaaact	1				no_annot
14762	cttttgcacc	1				no_annot
14763	taggagtcgc	1				no_annot
14764	tgtatagtta	1				no_annot
14765	caaaacagcc	1	TC11065	16	3	similar to GP|3885972|gb|AAC78143.1||AF102552 270 kDa ankyrin G isoform {Rattus norvegicus}, partial (3%)
14766	attgatgatg	1	C208883.1	94	0	
14767	ttgagtattt	1				no_annot
14768	ccgggttgtg	1				no_annot
14769	aattaaggag	1				no_annot
14770	ttgttctttg	1				no_annot
14771	tggtgaagcg	1				no_annot
14772	tccaaacgga	1				no_annot
14773	ctatagtgac	1				no_annot

14774	gtacgtcttc	1	C611742.1	59	0	
14775	tactttccgc	1	C609108.1	83	0	
14776	acaacttgta	1				no_annot
14777	ttagattaac	1				no_annot
14778	gtgttgtgat	1	TC8784	68	1	
14779	attgggatgg	1	C300809.1	38	0	
14779	attgggatgg	1	C704279.1	38	0	
14780	tgcttaaaaa	1				no_annot
14781	gtgtattttt	1				no_annot
14782	aagttgtatc	1	TC16873	28	2	
14783	taaactgtgt	1				no_annot
14784	ttctccttgt	1				no_annot
14785	caaatatgat	1				no_annot
14786	ccccgtcatc	1				no_annot
14787	cgactgacag	1				no_annot
14788	ttttatagtt	1	TC11180	96	1	
14788	ttttatagtt	1	TC14039	27	0	weakly similar to GP|21483368|gb|AAM52659.1||AY122147 LD02379p {Drosophila melanogaster}, partial (10%)
14789	gaatggagtt	1	TC11329	76	0	similar to GP|18460924|gb|AAK00530.1||AY024338 sialoglycoprotease GCP1 {Arabidopsis thaliana}, partial (8%)
14790	caaaatagtc	1	TC17440	4	8	
14791	gtaaataaac	1				no_annot
14792	aaacgggtag	1				no_annot
14793	gaatgatttt	1				no_annot
14794	aactgcagtc	1				no_annot
14795	ggagtgcttg	1				no_annot
14796	gtcatccagt	1	TC11537	62	1	
14797	catcagctac	1				no_annot
14798	gtatacgatg	1				no_annot
14799	cagaattcca	1	C307246.1	68	0	

14800	atgataacag	1				no_annot
14801	gttcgtatgc	1				no_annot
14802	tttttatgaa	1	TC17220	94	0	weakly similar to SP|O15488|GYG2_HUMAN Glycogenin-2(GN-2) (GN2). [Human] {Homo sapiens}, partial (41%)
14803	accaatgtac	1				no_annot
14804	atgataacaa	1	TC6994	71	0	similar to SP|P51136|KG3H_DICDI Glycogen synthase kinase-3 homolog(GSK-3). [Slime mold] {Dictyostelium discoideum}, partial (4%)
14804	atgataacaa	1	CD138333	30	1	
14805	ctgacagcct	1	C201168.1	26	1	
14805	ctgacagcct	1	C606297.1	26	1	
14806	aagaggaaaa	1				no_annot
14807	cagtgaattc	1	TC13211	66	1	similar to GP|18000909|gb|AAL55210.1||AF280305 cytochrome b {Mabuya fogoensis nicolauensis}, partial (6%)
14808	acgccagtgt	1				no_annot
14809	aataacaatc	1				no_annot
14810	acagggatgc	1				no_annot
14811	aaggagatat	1	TC8723	25	0	
14812	attgttttgt	1				no_annot
14813	taggaatagt	1				no_annot
14814	ttaacaaatg	1	TC12951	40	1	
14815	cggtcgcccg	1				no_annot
14816	gcgacccaac	1				no_annot
14817	ataaatcacg	1				no_annot
14818	ataatcactg	1				no_annot
14819	cacggacagt	1				no_annot
14820	gggagaagaa	1				no_annot
14821	gcgtaaggtc	1				no_annot
14822	tgcgacgggg	1	CD098357	51	1	
14823	gcgtaaggta	1				no_annot

14824	aggtattatt	1	TC16969	95	0	weakly similar to SP|Q12849|GRF1_HUMAN G-rich sequence factor-1 (GRSF-1). [Human] {Homo sapiens}, partial (15%)
14825	tcttaatctg	1				no_annot
14826	taccctatga	1				no_annot
14827	aatgctacct	1				no_annot
14828	gtagaagtag	1				no_annot
14829	ccctacatcg	1				no_annot
14830	cttgtttcac	1	TC10803	81	1	weakly similar to GP|7769869|gb|AAF69547.1|AC008007_22|AC008007 F12M16.18 {Arabidopsis thaliana}, partial (9%)
14831	atactgacat	1				no_annot
14832	cggacgaagt	1				no_annot
14833	gtgtatatga	1				no_annot
14834	tagtaaaaga	1	TC14476	44	0	
14835	tcaggaaaaa	1	C200047.1	34	0	
14835	tcaggaaaaa	1	C604243.1	33	0	
14836	gattcgtatg	1				no_annot
14837	tgtggcgtcc	1				no_annot
14838	cagtttagtc	1				no_annot
14839	tggtgctccg	1				no_annot
14840	acagttttgt	1				no_annot
14841	gacgagtagg	1	TC10736	20	1	similar to GP|22758850|gb|AAN05585.1||AF526199 ribosomal protein L22 {Argopecten irradians}, partial (96%)
14842	atcaatagcc	1				no_annot
14843	cccagctaac	1				no_annot
14844	aggctgtaaa	1				no_annot
14845	aaaacaatga	1				no_annot
14846	ttaaacatca	1				no_annot
14847	cggaaggaaa	1				no_annot
14848	aagatttaaa	1	CD201921	46	0	
14849	tacacaggtc	1				no_annot

14850	gaatgaatga	1				no_annot
14851	atcgaattgt	1				no_annot
14852	tacatctaat	1				no_annot
14853	gctatactgc	1				no_annot
14854	cgtgttttca	1				no_annot
14855	atttcatttg	1	M64539|Sma.624	87	0	Ferritin-2
14856	tatacagaga	1				no_annot
14857	taataaattc	1	TC9925	88	0	
14858	gattttgtca	1				no_annot
14859	gactagaaga	1	C606432.1	39	0	
14860	aacgaatgac	1				no_annot
14861	caggaatagt	1				no_annot
14862	ggatgatgaa	1	TC14445	68	1	similar to GP|27820011|gb|AAO25038.1||BT003281 LD01705p {Drosophila melanogaster}, partial (45%)
14863	cgttttatcc	1	C207992.1	70	0	
14863	cgttttatcc	1	C603334.1	74	0	
14864	tggagggtgt	1				no_annot
14865	cggcaataca	1				no_annot
14866	cagttcagaa	1				no_annot
14867	gcccctttttt	1	C210428.1	41	1	
14867	gcccctttttt	1	C606747.1	38	2	
14868	ttagcaccac	1	TC18533	60	1	
14869	ttagcaccaa	1				no_annot
14870	aaggtgggat	1				no_annot
14871	ctactactag	1				no_annot
14872	tgaataagtg	1				no_annot
14873	agaatcattt	1				no_annot
14874	tgttggtttc	1				no_annot
14875	tgacactata	1				no_annot
14876	cgcctagtgg	1				no_annot
14877	acgttttact	1				no_annot

14878	cacatttatt	1	CD117958	85	1	
14879	actttatatg	1	gi|4138864|gb|AF101196.1|AF101196	66	3	Schistosoma mansoni cytochrome c oxidase subunit 1 (cox1) mRNA, mitochondrial gene encoding mitochondrial protein, complete cds
14880	ttctcagaat	1				no_annot
14881	gaacagaaga	1				no_annot
14882	tacaaggcag	1				no_annot
14883	accttggagt	1	C314917.1	6	1	
14884	gactgtcggt	1				no_annot
14885	tgtagtttct	1				no_annot
14886	gacatagttt	1	TC8007	28	6	homologue to GP|9828635|gb|AAG00258.1|AC002130_23|AC002130 F1N21.1 {Arabidopsis thaliana}, partial (9%)
14887	gttgaatttt	1	C208915.1	38	3	
14888	tagtgcaata	1				no_annot
14889	acgccagtct	1	TC9255	61	0	
14890	tgaaccgtta	1				no_annot
14891	agactgatat	1				no_annot
14892	gcaacaacaa	1				no_annot
14893	tccaaacgac	1				no_annot
14894	agatatatta	1	C203140.1	1	8	
14894	agatatatta	1	C611085.1	1	8	
14895	taccatcttt	1				no_annot
14896	tacacatatg	1				no_annot
14897	ttactccact	1				no_annot
14898	ttatcttgtt	1	TC17082	35	3	similar to GP|1943772|gb|AAB52431.1||U97191 Rab family protein 2 {Caenorhabditis elegans}, partial (37%)
14899	tgataaagag	1				no_annot
14900	atgattgtat	1	TC16927	2	1	
14901	tatcattgtg	1	CD201806	86	0	
14902	aaagcacgaa	1				no_annot
14903	gaataaggca	1				no_annot

14904	accaacctaa	1				no_annot
14905	atttttatct	1	TC7414	48	2	PIR|S38867|S38867 eggshell protein - fluke (Schistosoma mansoni), complete
14906	gtcattcatt	1				no_annot
14907	cgcagataaa	1				no_annot
14908	attgggataa	1				no_annot
14909	gtgtcaaaaa	1	TC8134	3	7	weakly similar to GP|15291159|gb|AAK92848.1||AY051424 GH10029p {Drosophila melanogaster}, partial (40%)
14910	gaaccatttg	1	TC11368	72	3	
14911	tgttacgaat	1				no_annot
14912	ttcattgttc	1				no_annot
14913	tacggacaaa	1	C211164.1	95	0	
14913	tacggacaaa	1	C608732.1	92	0	
14914	caatctcact	1				no_annot
14915	cagccaattg	1				no_annot
14916	tatcactgat	1				no_annot
14917	atttttatcg	1	TC11379	98	0	similar to GP|20379845|gb|AAH27762.1||BC027762 mitochondrial ribosomal protein L2 {Mus musculus}, partial (17%)
14918	tgcctagtcc	1				no_annot
14919	acatcaatgt	1				no_annot
14920	attctaagta	1				no_annot
14921	aaaaaacttt	1	TC14752	77	0	
14922	agatgagttc	1				no_annot
14923	gtagaggggt	1	C304719.1	66	0	
14923	gtagaggggt	1	C702369.1	66	0	
14924	atttttatca	1				no_annot
14925	gttaactgga	1	TC18716	57	0	similar to GP|5668705|dbj|BAA82655.1||AB030506 B9 {Homo sapiens}, partial (21%)
14926	cgaataagta	1				no_annot
14927	tccctgttga	1	C602691.1	87	0	
14928	atagttctcc	1				no_annot

14929	atagttctca	1				no_annot
14930	aactttctgt	1				no_annot
14931	aaccagtgtg	1				no_annot
14932	catatccagg	1	AI977610	84	0	
14933	gccacgtcta	1	TC10504	39	3	
14934	cacattaaga	1				no_annot
14935	ttgagggcag	1				no_annot
14936	gcgtacgcgg	1				no_annot
14937	acgtcaggaa	1	CD150046	90	1	
14938	ccatcaacaa	1				no_annot
14939	gctgcgattc	1				no_annot
14940	acaatgaatt	1				no_annot
14941	attgttttat	1	TC13804	75	0	weakly similar to GP|2313045|dbj|BAA21711.1||D84347 rab8 {Drosophila melanogaster}, partial (87%)
14941	attgttttat	1	CD117678	56	0	
14942	cacgacgttg	1				no_annot
14943	acgcgggtgg	1	TC16803	7	2	weakly similar to GP|15293875|gb|AAK95130.1||AF401558 ribosomal protein L6 {Ictalurus punctatus}, partial (50%)
14944	tccttctaag	1				no_annot
14945	gttgtaaaat	1				no_annot
14946	gatttcctct	1	TC17552	26	0	weakly similar to GP|13785146|emb|CAA72626.2||Y11881 rab4A-like protein {Trichinella pseudospiralis}, partial (63%)
14947	tccttctaaa	1				no_annot
14948	ctgtagaatt	1				no_annot
14949	aagttgaaag	1				no_annot
14950	acgtcatacg	1				no_annot
14951	tcaaaaaatt	1	TC18203	18	0	
14951	tcaaaaaatt	1	CD183662	19	1	
14952	gacactagaa	1				no_annot
14953	caacaccgca	1				no_annot

14954	gcgcacgatt	1				no_annot
14955	agcaatgacg	1				no_annot
14956	catcattgtg	1				no_annot
14957	caaactgtat	1				no_annot
14958	tgatcgtaat	1	TC9083	50	1	weakly similar to GP|22946236|gb|AAF53076.2||AE003631 CG6258-PA {Drosophila melanogaster}, partial (35%)
14958	tgatcgtaat	1	CD080941	53	0	similar to GP|22946236|gb|AAF53076.2||AE003631 CG6258-PA {Drosophila melanogaster}, partial (10%)
14959	atggtggtct	1				no_annot
14960	aaatttcgga	1				no_annot
14961	gttgtaaaaa	1				no_annot
14962	taagcagctc	1				no_annot
14963	ttgagagaca	1	TC17727	22	0	similar to GP|2921270|gb|AAC24196.1||AF030130 corneodesmosin {Homo sapiens}, partial (4%)
14964	gaagtttgca	1				no_annot
14965	ctagtcgatt	1	TC16979	83	3	weakly similar to SP|Q04451|SYG_BOMMO Glycyl-tRNA synthetase(Glycine--tRNA ligase) (GlyRS). [Silk moth] {Bombyx mori}, partial (76%)
14966	ttttctgtat	1	TC13907	42	2	
14967	taactgaatt	1				no_annot
14968	agacttgttg	1	TC14801	84	0	
14969	gaaatatatt	1				no_annot
14970	aggtataacc	1	C605209.1	34	3	
14971	cgtgtacaag	1				no_annot
14972	gagaaaaaaa	1				no_annot
14973	cctacacgag	1	C606337.1	93	0	
14974	cttacggatg	1				no_annot
14975	actccttggt	1				no_annot
14976	aaaaacgaaa	1	AI977471	40	0	
14977	atgacagtag	1				no_annot
14978	atgagggtgc	1	TC7424	86	1	similar to GP|7294506|gb|AAF49848.1||AE003539 CG11274-PA {Drosophila melanogaster}, partial (6%)

14979	tgtttgccga	1				no_annot
14980	ttgctggatg	1	C200397.1	6	15	
14980	ttgctggatg	1	C200841.1	14	4	
14980	ttgctggatg	1	C201517.1	11	10	
14980	ttgctggatg	1	C600216.1	16	11	
14980	ttgctggatg	1	C610370.1	5	13	
14980	ttgctggatg	1	C611803.1	50	2	
14980	ttgctggatg	1	C700782.1	5	2	
14981	taaccaccat	1				no_annot
14982	ctatgtatct	1				no_annot
14983	gtaaattaca	1				no_annot
14984	acatagaaaa	1				no_annot
14985	cagagaccgt	1				no_annot
14986	tatacttttg	1				no_annot
14987	tcgaacagct	1				no_annot
14988	agaaaacgac	1				no_annot
14989	tttttttcca	1	M27294|Sma.59	98	0	3-hydroxy-3-methylglutaryl coenzyme A reductase
14990	cacacaaagt	1				no_annot
14991	gtcgaagctg	1				no_annot
14992	agattgtggt	1				no_annot
14993	atgacgaaca	1	TC10785	43	7	similar to SP|Q90YP3|RS28_ICTPU 40S ribosomal protein S28. [Channel catfish] {Ictalurus punctatus}, partial (87%)
14994	tgggtgctta	1				no_annot
14995	taaacatcca	1				no_annot
14996	tgatatacgt	1	TC10437	46	4	PIR|S34275|S34275 protein disulfide-isomerase homolog precursor - fluke (Schistosoma mansoni), complete
14997	tgttaccatt	1	TC14184	86	0	similar to GP|14646761|dbj|BAB61954.1||AB060654 23-kDa proteolipid {Mus musculus}, partial (33%)
14997	tgttaccatt	1	TC14185	56	3	similar to GP|14646761|dbj|BAB61954.1||AB060654 23-kDa proteolipid {Mus musculus}, partial (63%)
14998	aaagtgatag	1				no_annot

14999	gttggcattc	1				no_annot
15000	tatattcatc	1	AI976755	87	0	
15000	tatattcatc	1	CD083291	92	0	
15001	tgataatggt	1				no_annot
15002	tctgctataa	1				no_annot
15003	gaagtttgaa	1				no_annot
15004	ttccaaaaga	1				no_annot
15005	gctattgaac	1				no_annot
15006	aatagcattg	1	CD076770	30	0	
15007	atgtgtacgt	1				no_annot
15008	aaccacaaac	1	TC7108	61	8	
15008	aaccacaaac	1	TC7108	61	4	
15008	aaccacaaac	1	CD144918	54	0	homologue to GP|160925|gb|AAA29853.1||M94346 A.1.12/9 antigen {Schistosoma mansoni}, partial (6%)
15008	aaccacaaac	1	CD153646	89	0	
15009	atacaaatat	1				no_annot
15010	agattttatg	1				no_annot
15011	tgcaattgac	1				no_annot
15012	ctgcgcctat	1				no_annot
15013	gatttatcaa	1	TC10132	57	1	
15014	aattgattat	1				no_annot
15015	caaatttcac	1				no_annot
15016	gttaattgtt	1				no_annot
15017	gtggtgattg	1				no_annot
15018	ctgtcaattt	1	TC7554	65	2	similar to GP|21901963|dbj|BAC05520.1||AB078412 casein kinase I {Ciona savignyi}, partial (78%)
15019	ggtttgtttg	1				no_annot
15020	ggcaacaaag	1				no_annot
15021	cttggaggac	1				no_annot
15022	ctaaacttgt	1	TC17839	28	5	
15023	catttcactt	1	TC9938	62	0	

15024	tgtgcctaat	1				no_annot
15025	gatctaaact	1	AY308023|Sma.1181	55	0	LTR-retrotransposon Boudicca nonfunctional integrase mRNA, partial sequence
15026	ttcacgaatc	1				no_annot
15027	ggttgtgaaa	1				no_annot
15028	atgtatctag	1				no_annot
15029	aggtgagaaa	1				no_annot
15030	ttatctagct	1				no_annot
15031	aagaggtaag	1				no_annot
15032	tgtatgtatt	1				no_annot
15033	ttcgttcacc	1	C204694.1	2	1	
15034	agcttccttt	1				no_annot
15035	tttcattttg	1				no_annot
15036	aagaggtaaa	1	TC10640	39	7	
15037	tacacaaaca	1	C211301.1	31	3	
15038	aatcccaaaa	1				no_annot
15039	caaccaccaa	1				no_annot
15040	tgctgctttt	1	CD081047	47	0	weakly similar to GP|21430834|gb|AAM51095.1||AY119235 SD18780p {Drosophila melanogaster}, partial (12%)
15041	atgagagctt	1				no_annot
15042	aataactatc	1				no_annot
15043	gaccaggtgc	1	TC16470	53	0	
15044	ttttctcttt	1	TC11215	54	1	weakly similar to GP|1079683|gb|AAB68306.1||U39205 Lpe13p {Saccharomyces cerevisiae}, partial (41%)
15045	ggtcttctac	1				no_annot
15046	tgacacaaaa	1				no_annot
15047	ggaacactga	1				no_annot
15048	ttttgaaaaa	1	TC11108	20	2	similar to GP|7299327|gb|AAF54521.1||AE003686 CG12818-PA {Drosophila melanogaster}, partial (16%)
15048	ttttgaaaaa	1	TC12673	17	3	
15048	ttttgaaaaa	1	CD191597	20	1	similar to GP|7299327|gb|AAF54521.1||AE003686 CG12818-PA {Drosophila melanogaster}, partial (9%)

15048	ttttgaaaaa	1	CD191597	20	0	similar to GP|7299327|gb|AAF54521.1||AE003686 CG12818-PA {Drosophila melanogaster}, partial (9%)
15049	tttatagcct	1				no_annot
15050	ggtaggatac	1				no_annot
15051	acatcaatag	1	TC11300	53	1	weakly similar to SP|Q94758|DYL1_SCHMA Dynein light chain. [Blood fluke] {Schistosoma mansoni}, complete
15052	aggaacatct	1				no_annot
15053	tcactgtgat	1				no_annot
15054	ctaagcgatt	1				no_annot
15055	ggacaacgca	1				no_annot
15056	aaccaattct	1				no_annot
15057	taggtttggg	1	TC12274	58	0	
15058	caagacaagg	1				no_annot
15059	tctttgcggt	1	TC18357	21	0	
15059	tctttgcggt	1	CD080979	58	0	
15060	tcttatgtct	1				no_annot
15061	ctcgctttcg	1				no_annot
15062	acaaagattg	1				no_annot
15063	gtgtatttgg	1				no_annot
15064	aaagttgtta	1	CD093734	77	0	
15065	gccattaaca	1				no_annot
15066	acctctcgca	1	TC7928	66	1	
15067	acactcacga	1				no_annot
15068	tacgtgaaca	1				no_annot
15069	cactgtttct	1	TC8477	80	1	similar to GP|9294023|dbj|BAB01926.1||AP001307 multispanning membrane protein-like {Arabidopsis thaliana}, partial (3%)
15070	ttacacctag	1				no_annot
15071	tatatgcgcc	1				no_annot
15072	aataaaatat	1	U30663|Sma.20	87	0	Sm24
15073	atatttctgt	1	TC19377	28	2	

15074	ttggagaaac	1				no_annot
15075	ctagacgttt	1	TC8273	80	0	
15076	agtgtctgga	1				no_annot
15077	ctaattaaga	1				no_annot
15078	tgggaaaatg	1	TC8217	7	1	
15078	tgggaaaatg	1	TC12382	12	0	
15079	cacacaaacg	1	C200575.1	18	0	
15079	cacacaaacg	1	C603654.1	8	3	
15080	gaaaagagtg	1				no_annot
15081	gaaatgacac	1	TC7783	71	3	similar to SP|Q27954|COPA_BOVIN Coatomer alpha subunit (Alpha-coat protein) (Alpha-COP) (HEPCOP) (HEP-COP) [Contains: Xenin (Xenopsin-related peptide); Proxenin]. [Bovine] {Bos taurus}, partial (26%)
15082	cgaacgtttt	1				no_annot
15083	agaacttcaa	1				no_annot
15084	gcaagaaaac	1				no_annot
15085	aataaaatac	1	TC13165	50	0	
15085	aataaaatac	1	CD135121	28	0	similar to GP|20073103|gb|AAH26747.1||BC026747 U6 snRNA-associated SM-like protein 4 {Mus musculus}, partial (20%)
15086	gttacatcat	1				no_annot
15087	aataaaataa	1				no_annot
15088	gtagttttca	1				no_annot
15089	atttgaactt	1				no_annot
15090	aagtcctttt	1				no_annot
15091	gtaactagac	1				no_annot
15092	aaaatacaga	1	TC7969	13	4	
15093	gttttgaccc	1				no_annot
15094	catagggact	1	CD135581	22	1	similar to SP|P33552|CKS2_HUMAN Cyclin-dependent kinases regulatory subunit 2 (CKS-2). [Human] {Homo sapiens}, partial (70%)
15095	gtaatgaata	1				no_annot
15096	ggaaagcaag	1				no_annot

15097	caactcgata	1				no_annot
15098	acatttttga	1				no_annot
15099	aataccagtt	1				no_annot
15100	tttaaaggtg	1				no_annot
15101	agttccacta	1				no_annot
15102	ccatattgta	1	CD166810	8	3	
15103	aaagtgttgc	1				no_annot
15104	ccaccaaagc	1	TC7478	16	1	similar to GP|24266991|gb|AAN52391.1||AF548336 ribosomal protein S25 {Branchiostoma belcheri}, partial (72%)
15105	tgaatatgta	1				no_annot
15106	atgtggcaaa	1	C610908.1	11	2	
15107	ggacgtaaca	1				no_annot
15108	cgacgctttt	1				no_annot
15109	tcacttaaat	1				no_annot
15110	ctatcaccca	1	TC11044	15	3	weakly similar to GP|22268103|gb|AAH27427.1||BC027427 STIP1 homology and U-Box containing protein 1 {Mus musculus}, partial (69%)
15111	actctgtatg	1				no_annot
15112	tgtccgatca	1	TC15653	43	2	
15113	acttaaatct	1				no_annot
15114	tccaaagttc	1				no_annot
15115	tgaaacagtc	1				no_annot
15116	ggtgcgaatt	1				no_annot
15117	tgaaacagta	1				no_annot
15118	cgctagagca	1	TC10924	61	1	
15119	tatttggagc	1				no_annot
15120	tatgttactt	1				no_annot
15121	gtatagaaag	1	C202110.1	20	1	
15121	gtatagaaag	1	C604221.1	19	1	
15121	gtatagaaag	1	C608594.1	38	0	

15122	atacaattgg	1				no_annot
15123	gagagaatca	1	C709781.1	21	0	
15124	ttcagtcaca	1				no_annot
15125	cgctaattca	1				no_annot
15126	tcacggtatt	1				no_annot
15127	ttccaaaaaa	1				no_annot
15128	tacctggtta	1				no_annot
15129	tatgttactg	1				no_annot
15130	cattcgattg	1				no_annot
15131	cgcatatttg	1	TC6907	81	1	weakly similar to GP|28852477|gb|AAO55550.1||AE016863 glutathione S-transferase family protein {Pseudomonas syringae pv. tomato str. DC3000}, partial (20%)
15132	gcctggccca	1				no_annot
15133	atacacaaat	1	TC18244	75	0	
15134	attttacaga	1				no_annot
15135	gtgaagtatg	1	TC12250	63	0	similar to GP|23093178|gb|AAF49211.2||AE003519 CG6841-PA {Drosophila melanogaster}, partial (15%)
15136	gaaataaaaa	1	CD077122	9	1	
15137	tccatcatct	1	C204411.1	95	0	
15137	tccatcatct	1	C601339.1	93	0	
15138	tttaaatatt	1				no_annot
15139	catactatct	1				no_annot
15140	tgctcctgtc	1				no_annot
15141	tacatccggt	1	TC14126	96	0	weakly similar to GP|18700307|dbj|BAB85122.1||AB066095 tyrosine-rich heat shock protein {Mus musculus}, partial (32%)
15142	cctctcagcc	1				no_annot
15143	tgcgataaat	1				no_annot
15144	ccctcaaact	1	CD158218	83	0	similar to SP|Q9BXB4|ORPB_HUMAN Oxysterol binding protein-related protein 11 (OSBP-related protein 11) (ORP-11). [Human] {Homo sapiens}, partial (7%)
15145	gttgacaacg	1	M80214|Sma.820	42	1	Alpha-tubulin (sat-1)
15146	tatactatac	1	CD093234	43	0	

15146	tatactatac	1	CD125243	90	0	
15147	atgaaaagcg	1				no_annot
15148	gtgattttta	1				no_annot
15149	ccttatgtaa	1				no_annot
15150	cttcaacata	1	AI395559	72	0	
15151	tgcatcggtc	1				no_annot
15152	tttgctctct	1				no_annot
15153	gcggatgccg	1				no_annot
15154	tttattagtc	1				no_annot
15155	accacgccat	1	CD120500	85	0	similar to SP|P49366|DHYS_HUMAN Deoxyhypusine synthase(DHS). [Human] {Homo sapiens}, partial (11%)
15156	cgtaccattt	1				no_annot
15157	tgaacaacta	1				no_annot
15158	ccggtaaact	1				no_annot
15159	cgtttacaca	1				no_annot
15160	tacgaaacta	1	TC15697	57	2	
15161	acacttactg	1				no_annot
15162	gcgcttatga	1				no_annot
15163	tggacgcgtt	1				no_annot
15164	ttcattgtgt	1				no_annot
15165	aaatgccaac	1				no_annot
15166	agttaatatt	1	C208300.1	44	1	
15167	agcgacacct	1				no_annot
15168	atacttgcga	1				no_annot
15169	tgctgcatca	1				no_annot
15170	ggattagcga	1	C706801.1	7	2	
15171	gggtatagtt	1				no_annot
15172	tacacataga	1				no_annot
15173	tgcggagaag	1				no_annot
15174	tgcgtacgat	1	C201718.1	4	6	
15174	tgcgtacgat	1	C604621.1	4	6	

15175	ttggttttgg	1				no_annot
15176	atgctttgat	1				no_annot
15177	tttatacctc	1				no_annot
15178	cacagcgagt	1				no_annot
15179	tatcgacgta	1				no_annot
15180	tctggctcgg	1				no_annot
15181	cagagttggt	1				no_annot
15182	gcgactcaat	1				no_annot
15183	aataataatg	1	CD073195	27	3	
15184	tagtaataaa	1				no_annot
15185	tatgcaactg	1				no_annot
15186	gagaaataag	1				no_annot
15187	ataacgatgc	1				no_annot
15188	agcagtgtaa	1	CD069962	89	0	weakly similar to PIR|T39903|T39903 serine-rich protein - fission yeast (Schizosaccharomyces pombe), partial (7%)
15189	tcacatatca	1				no_annot
15190	aactattcgg	1				no_annot
15191	tgctactact	1				no_annot
15192	gaacagtaca	1	C200438.1	40	1	
15192	gaacagtaca	1	C601986.1	40	1	
15193	cgtttacaat	1				no_annot
15194	aactattcgc	1				no_annot
15195	ctagccattt	1				no_annot
15196	ttcaaaagta	1				no_annot
15197	tttgctctat	1				no_annot
15198	tcagattgat	1	TC10600	90	2	
15198	tcagattgat	1	TC12650	23	0	similar to EGAD|3557|3526 transforming growth factor beta {Homo sapiens}, partial (5%)
15198	tcagattgat	1	CD195418	86	0	homologue to GP|2959981|emb|CAA10901.1||AJ222641 GSK3 beta {Paracentrotus lividus}, partial (22%)

15199	aattggtgca	1	CD197583	14	0	weakly similar to GP|7290625|gb|AAF46074.1||AE003435 CG4078-PA {Drosophila melanogaster}, partial (5%)
15200	cgtttacaaa	1	C606449.1	64	0	
15201	acgagtcgaa	1				no_annot
15202	gaaaaaatca	1				no_annot
15203	tgacccaggc	1				no_annot
15204	agatttgggt	1				no_annot
15205	ttaacaaaac	1	TC7761	80	3	
15205	ttaacaaaac	1	CD147775	62	2	
15206	acttagtgtc	1				no_annot
15207	ctatgtttcc	1				no_annot
15208	ttatggccat	1				no_annot
15209	tatttctcta	1				no_annot
15210	taaagataaa	1	C202687.1	94	0	
15211	aatgaagttt	1				no_annot
15212	cacacatagg	1				no_annot
15213	cacttgtata	1				no_annot
15214	ggcaagtgga	1				no_annot
15215	gctgtatgta	1	TC10094	15	4	similar to GP|20151661|gb|AAM11190.1||AY094837 LD45081p {Drosophila melanogaster}, partial (4%)
15216	agttcggagg	1				no_annot
15217	ggccctaaag	1	AF310263|Sma.695	5	6	Heat shock protein HSP60
15218	tacatttaat	1	TC14713	57	1	weakly similar to GP|12958095|gb|AAK07485.1||AY013563 gag-pol polyprotein {Clonorchis sinensis}, partial (8%)
15219	ccaccaaaaa	1	BG930414	30	0	
15220	tcgcctagat	1				no_annot
15221	caccttcaat	1	TC17151	43	1	similar to GP|927736|gb|AAB64908.1||U33050 Ydr492wp; CAI: 0.19 {Saccharomyces cerevisiae}, partial (9%)
15222	gaatacccca	1				no_annot
15223	tacagacttc	1	TC8319	71	1	

15224	tgttcctacc	1				no_annot
15225	aacactgttg	1				no_annot
15226	ggtaggttcc	1				no_annot
15227	gttagataca	1				no_annot
15228	aaattgataa	1	L01634|Sma.848	66	9	Myosin heavy chain (MYH)
15229	aagggaagtt	1				no_annot
15230	attccgatat	1				no_annot
15231	acgaacgaac	1				no_annot
15232	ctagatcgaa	1				no_annot
15233	attgatcatc	1				no_annot
15234	tatgtctcga	1				no_annot
15235	caatcggtcc	1				no_annot
15236	acaatagtcc	1	TC7791	79	1	weakly similar to GP|5360166|gb|AAD42887.1|AF158186_1|AF158186 pEachy {Rattus norvegicus}, partial (83%)
15237	aaaactggta	1				no_annot
15238	agttcaagtc	1	AI014229	47	0	GP|29340501|gb penicillin-binding protein D-alanyl-D-alanine carboxypeptidase, partial (2%)
15239	tttaagacat	1				no_annot
15240	gtaagacaca	1				no_annot
15241	gacatagtcc	1				no_annot
15242	cgtatgaaaa	1				no_annot
15243	tagtgtaatt	1	CD084799	73	0	
15244	ttcggatgcg	1				no_annot
15245	tatattcagt	1	TC11345	53	0	
15246	gtgaaagtac	1				no_annot
15247	aatcaactac	1				no_annot
15248	tgttaactat	1				no_annot
15249	tccccgaagc	1				no_annot
15250	cgttgatcta	1	TC7411	38	1	weakly similar to SP|Q9DA39|ZPRO_MOUSE Z-protein (0610007H07Rik). [Mouse] {Mus musculus}, partial (46%)

15251	attatcgaga	1	TC9592	56	2	
15252	tacgtccata	1	CD145686	67	2	weakly similar to SP|O02414|DYL1_ANTCR Dynein light chain LC6, flagellar outer arm. [Sea urchin] {Anthocidaris crassispina}, partial (60%)
15253	ggttctccct	1				no_annot
15254	aaaaccggaa	1				no_annot
15255	ccaatgctcc	1				no_annot
15256	cttatgctat	1	TC17044	15	0	similar to GP|15010510|gb|AAK77303.1||AY047571 GH08349p {Drosophila melanogaster}, partial (13%)
15257	ttgatgaatg	1				no_annot
15258	taatccagtc	1				no_annot
15259	ataacgatcc	1				no_annot
15260	gggctctact	1				no_annot
15261	gaagtagatt	1	C207558.1	26	0	
15261	gaagtagatt	1	C612189.1	26	0	
15262	agttgcaatc	1				no_annot
15263	ttacttcact	1	C302118.1	53	0	
15263	ttacttcact	1	C709194.1	48	0	
15264	ctgctggagt	1				no_annot
15265	cctgttcaaa	1				no_annot
15266	catagcacac	1				no_annot
15267	agaagtcgtg	1				no_annot
15268	gcactgtata	1				no_annot
15269	cgtatcgacc	1				no_annot
15270	tccgccccga	1				no_annot
15271	tacttcgagc	1				no_annot
15272	aatggttaaa	1				no_annot
15273	gaaaagtgtt	1				no_annot
15274	cgttgaacgt	1				no_annot
15275	aataaattat	1				no_annot
15276	ttagttgatc	1				no_annot
15277	cagaagagtg	1				no_annot

15278	ctaccacagt	1				no_annot
15279	aataaattaa	1				no_annot
15280	gaaataggac	1				no_annot
15281	gattcactgg	1				no_annot
15282	ttaattggat	1	TC11158	22	3	weakly similar to GP|12805489|gb|AAH02220.1||BC002220 Clk4 protein {Mus musculus}, partial (8%)
15283	catattcagc	1				no_annot
15284	tagaaaatga	1	TC7950	5	6	weakly similar to GP|28272155|emb|CAD65059.1||AL935260 extracellular protein {Lactobacillus plantarum WCFS1}, partial (7%)
15284	tagaaaatga	1	TC8570	73	0	weakly similar to SP|Q96A29|FCT1_HUMAN GDP-fucose transporter 1. [Human] {Homo sapiens}, partial (57%)
15285	ctctttcaca	1				no_annot
15286	actctggggt	1				no_annot
15287	ccaccccctt	1				no_annot
15288	agatgagtaa	1	C208428.1	47	1	
15289	tggtaaaata	1				no_annot
15290	tgtcaagcct	1	TC7092	72	0	
15290	tgtcaagcct	1	TC7109	77	1	weakly similar to PIR|T13154|T13154 polycomb protein enhancer - fruit fly (Drosophila melanogaster), partial (4%)
15291	cattctcgct	1				no_annot
15292	cgtaaaacta	1	TC7212	68	4	
15293	aacgaaattt	1	C203189.1	42	0	
15294	ggaggattta	1				no_annot
15295	aatattgcca	1	CD148998	18	0	weakly similar to SP|Q8WYQ5|DGR8_HUMAN DGCR8 protein (DiGeorge syndrome critical region 8). [Human] {Homo sapiens}, partial (3%)
15296	aggaagcctt	1				no_annot
15297	ttgtttataa	1				no_annot
15298	tctataaccc	1				no_annot
15299	atctattatt	1				no_annot

15300	tgtaagatac	1				no_annot
15301	ccacttggat	1				no_annot
15302	ttgtggtttt	1				no_annot
15303	tttaaaaaaa	1	TC11232	6	4	
15303	tttaaaaaaa	1	CD193909	20	0	
15304	ttcacagtca	1				no_annot
15305	ctcatcctga	1	C201342.1	15	4	
15305	ctcatcctga	1	C604293.1	28	4	
15306	tagtgattct	1	TC13425	31	2	similar to GP|8050807|gb|AAF71747.1||AF257741 E4tf1-60 transcription factor {Danio rerio}, partial (9%)
15307	cgtgcttaga	1	TC7143	64	8	similar to GP|16930529|gb|AAL31950.1||AF421549 CDH1-D {Gallus gallus}, partial (19%)
15307	cgtgcttaga	1	BG932091	87	0	homologue to EGAD|131264|140075 hemolysin {Acanthamoeba polyphaga}, partial (11%)
15308	attggcctgg	1				no_annot
15309	ttaatttacc	1	C300304.1	59	0	
15309	ttaatttacc	1	C709602.1	59	0	
15310	cctcaaaaaa	1				no_annot
15311	cttcatttgt	1				no_annot
15312	ttaacgtcca	1	CD166380	56	1	weakly similar to GP|28302258|gb|AAH46594.1||BC046594 ORM1-like 3 (S. cerevisiae) {Mus musculus}, partial (60%)
15313	tatttcaccg	1				no_annot
15314	ccggtgtcca	1				no_annot
15315	ggtgcagtgg	1				no_annot
15316	atcaaggctg	1				no_annot
15317	tcgtgttgat	1	TC17699	96	1	
15318	tgaagcgctt	1				no_annot
15319	cacaaacagc	1				no_annot
15320	gtctcaacta	1				no_annot
15321	ctacctttat	1				no_annot
15322	gaacctggtg	1				no_annot

15323	atacattata	1				no_annot
15324	gactaactct	1				no_annot
15325	tgttaccacc	1				no_annot
15326	aaatcagtta	1				no_annot
15327	attattgatt	1	TC15363	74	0	weakly similar to SP|Q9V6K3|ROR2_DROME Tyrosine-protein kinase transmembrane receptor Ror2 precursor(Neurospecific receptor tyrosine kinase). [Fruit fly] {Drosophila melanogaster}, partial (7%)
15328	accttttctt	1	C210668.1	19	4	
15328	accttttctt	1	C604172.1	54	4	
15329	gatacgatgc	1	CD152387	85	0	
15330	ggtacgtgat	1				no_annot
15331	tctaaaagta	1	CD137890	94	0	
15332	aacctacgct	1				no_annot
15333	tggcatccgt	1	TC7636	57	1	weakly similar to GP|21064457|gb|AAM29458.1||AY113453 RE35686p {Drosophila melanogaster}, partial (32%)
15334	tagataggga	1				no_annot
15335	gtatcattct	1				no_annot
15336	actctgtagt	1	CD200803	37	1	
15337	tgttctaact	1	C209342.1	6	4	
15338	ccatattgga	1				no_annot
15339	tgaaccctta	1				no_annot
15340	gggtggacaa	1	C202110.1	8	5	
15340	gggtggacaa	1	C604221.1	8	5	
15341	catacacaga	1	TC13493	96	0	homologue to PIR|B54525|B54525 major female-specific polypeptide (frame 2) - fluke (Schistosoma mansoni) (fragment), partial (63%)
15342	cggccttccc	1				no_annot
15343	atgaataccg	1				no_annot
15344	ggtttgttca	1	CD133809	7	2	
15345	caatacaacc	1				no_annot
15346	cagtggagcg	1				no_annot

15347	catcctctaa	1				no_annot
15348	gaaaacaaaa	1	TC14143	25	1	
15348	gaaaacaaaa	1	CD088384	52	0	weakly similar to GP|28272281|emb|CAD65194.1||AL935260 extracellular protein {Lactobacillus plantarum WCFS1}, partial (15%)
15349	tgttatcatc	1				no_annot
15350	cgccagttat	1				no_annot
15351	acgttgatgc	1				no_annot
15352	aggcgatgta	1	TC10997	15	2	
15353	gcattgtcag	1				no_annot
15354	tgttgaacaa	1				no_annot
15355	gatgattcag	1				no_annot
15356	ggactcataa	1				no_annot
15357	gaacaactcg	1				no_annot
15358	tatcacattg	1				no_annot
15359	aaacgctgct	1				no_annot
15360	agattttaat	1				no_annot
15361	ccgtaatttc	1				no_annot
15362	tactaaactg	1				no_annot
15363	ccaaaaaaaa	1				no_annot
15364	tccatttttc	1				no_annot
15365	agtttttgtt	1	TC10955	25	3	similar to GP|23273556|gb|AAH35974.1||BC035974 DEAD/H (Asp-Glu-Ala-Asp/His) box polypeptide 15 {Homo sapiens}, partial (34%)
15366	atgtaaaatt	1				no_annot
15367	tctacgtatt	1				no_annot
15368	ctgtatgatg	1				no_annot
15369	gattaatgaa	1	TC9510	13	4	
15369	gattaatgaa	1	CD124060	46	1	
15370	tagttttgat	1				no_annot
15371	agaatggcat	1				no_annot

15372	tccatcttat	1	TC13682	75	1	similar to GP|3851612|gb|AAC72372.1||AF095937 succinate dehydrogenase Ip subunit {Gallus gallus}, partial (84%)
15372	tccatcttat	1	TC13683	49	1	similar to GP|3851612|gb|AAC72372.1||AF095937 succinate dehydrogenase Ip subunit {Gallus gallus}, partial (64%)
15373	aactgctgat	1	TC14203	75	0	similar to GP|3283072|gb|AAC25114.1||AF056022 p60 katanin {Homo sapiens}, partial (36%)
15374	ttagtgggag	1				no_annot
15375	ccaaggagtt	1				no_annot
15376	ttcaaatgtt	1	TC10858	92	0	similar to SP|P23232|GBB_LOLFO Guanine nucleotide-binding protein beta subunit. [Northern European squid] {Loligo forbesi}, complete
15376	ttcaaatgtt	1	TC18038	32	1	weakly similar to GP|6822210|emb|CAB70916.1||AL939109 adenine phosphoribosiltransferase {Streptomyces coelicolor A3(2)}, partial (40%)
15377	cgttcgtgaa	1				no_annot
15378	catttatgtg	1				no_annot
15379	atgtaaaatg	1				no_annot
15380	tatgacaggc	1				no_annot
15381	aacatatgcg	1				no_annot
15382	tattcaactt	1	TC13012	6	4	
15383	cagacagctt	1				no_annot
15384	attggcctcg	1				no_annot
15385	atctcaccta	1	TC16556	40	2	weakly similar to PIR|T39903|T39903 serine-rich protein - fission yeast (Schizosaccharomyces pombe), partial (10%)
15386	gaacaactat	1				no_annot
15387	ttcaaatgta	1				no_annot
15388	tttgtaatag	1				no_annot
15389	taaatgagtt	1				no_annot
15390	gaataacgaa	1				no_annot
15391	tagaaaatat	1				no_annot
15392	atttcgtgcc	1				no_annot

15393	cagacagctc	1				no_annot
15394	ttagtgtaca	1	TC10718	49	0	weakly similar to GP|160687|gb|AAA29765.1||J03983 serine rich protein (SERP I) {Plasmodium falciparum}, partial (3%)
15394	ttagtgtaca	1	CD078537	60	2	
15395	cttatttgtg	1	AY842253|Sma.1228	91	1	Putative fructose-1,6-bisphosphatase
15396	tattcaacta	1				no_annot
15397	agatttcgtg	1				no_annot
15398	gagttcaaag	1	TC16639	50	0	
15399	cagacagcta	1	C310317.1	89	0	
15400	attcaagcca	1				no_annot
15401	aacgtatatt	1	CD069125	17	0	
15402	atgcgtaaac	1				no_annot
15403	cttatttgta	1	CD077157	38	1	
15404	tctaactctc	1				no_annot
15405	ccggataatg	1				no_annot
15406	cttttcaatg	1				no_annot
15407	tattcgatca	1	C609621.1	72	2	
15408	gcttaggtag	1	CD074757	27	2	
15409	tttactactg	1				no_annot
15410	aaagaggaaa	1				no_annot
15411	tgcgctagat	1				no_annot
15412	tatgtgcaca	1				no_annot
15413	atatattcat	1	CD080278	7	1	similar to GP|1517816|gb|AAB06962.1||U33833 helicase {Homo sapiens}, partial (3%)
15414	ggcgttatct	1	TC7336	65	2	homologue to SP|P05217|TBB2_HUMAN Tubulin beta-2 chain. [Mouse] {Mus musculus}, complete
15415	ttgaacgttt	1				no_annot
15416	atcattaatt	1	CD136598	68	1	
15417	gtcaggcacc	1				no_annot

15418	gttctcgaca	1	TC11346	58	0	weakly similar to GP|27527126|emb|CAC84717.1||AJ311981 invasion-associated protein {Listeria monocytogenes}, partial (21%)
15419	gcatacaaag	1				no_annot
15420	aaacgtaggt	1				no_annot
15421	aaattgttag	1				no_annot
15422	tcaattagga	1				no_annot
15423	cacttcgaac	1				no_annot
15424	gcatacaaac	1	C201394.1	31	2	
15424	gcatacaaac	1	C610914.1	29	4	
15425	ccaatttgta	1	AI977563	32	0	
15426	gaggcatatg	1				no_annot
15427	gaatgtaaat	1				no_annot
15428	ttcaaaagga	1				no_annot
15429	ctgtcgtgtt	1				no_annot
15430	tttgtgtgtt	1				no_annot
15431	tagtgctacc	1				no_annot
15432	caacaaactg	1				no_annot
15433	ttattgaaat	1				no_annot
15434	tgggatgcta	1				no_annot
15435	gaaaactagt	1				no_annot
15436	cttgtaataa	1				no_annot
15437	ttgcaggtat	1	TC7952	30	1	
15438	gaaaagagaa	1				no_annot
15439	taacagatag	1				no_annot
15440	ctgtcgtgtc	1				no_annot
15441	tcttcgccaa	1				no_annot
15442	aaccgacatt	1				no_annot
15443	tgatgtaggg	1	C610532.1	62	0	
15444	gtgactaatt	1				no_annot
15445	gagctgatct	1				no_annot

15446	attaatgtta	1	TC14287	79	1	weakly similar to SP|Q9UIJ5|ZDH2_HUMAN Zinc finger DHHC domain containing protein 2 (Zinc finger protein 372) (Reduced expression associated with metastasis protein) (Ream) (Reduced expression in cancer protein) (Rec). [Human] {Homo sapiens}, partial (24%)
15447	cggttcagga	1				no_annot
15448	gatacgatac	1	TC17965	44	1	similar to SP|O75027|ABC7_HUMAN ATP-binding cassette, sub-family B, member 7, mitochondrial precursor (ATP-binding cassette transporter 7) (ABC transporter 7 protein). [Human] {Homo sapiens}, partial (27%)
15449	gggcgagtgg	1				no_annot
15450	gtcagtacct	1				no_annot
15451	ccaaaccctt	1	CD069584	38	0	
15452	gattgtagta	1	CD186168	22	0	
15453	aaactcgctg	1				no_annot
15454	ctgtgcgtgt	1				no_annot
15455	catacacaat	1				no_annot
15456	ataaaagccg	1	C203435.1	4	2	
15457	atttaccaat	1				no_annot
15458	cctatcctgg	1	TC17160	12	4	
15459	tttttatttc	1	C604690.1	24	5	
15460	atgttctgtg	1				no_annot
15461	atcctactcg	1	TC16966	75	1	weakly similar to GP|2104697|gb|AAB58269.1||U93847 elongation factor-2 kinase EFK-1B isoform {Caenorhabditis elegans}, partial (27%)
15462	aggattggca	1				no_annot
15463	tacttgggat	1	TC8888	71	0	
15463	tacttgggat	1	TC16415	75	0	
15464	gaggaagaat	1	CD080191	22	0	
15465	ttgctatgta	1				no_annot
15466	ctatagatgt	1				no_annot
15467	gtctcgttga	1				no_annot
15468	gccttcgaaa	1				no_annot

15469	aaatgtcacc	1				no_annot
15470	gaatcttcat	1				no_annot
15471	tttagcaagt	1	TC15287	33	1	
15472	ttattacttg	1	TC17096	99	0	similar to GP|10443347|emb|CAC10445.1||AJ297711 CDC2L5 protein kinase {Sphaerechinus granularis}, partial (27%)
15473	taaccctttc	1				no_annot
15474	gaccagctgg	1				no_annot
15475	cttgtgtgtt	1				no_annot
15476	caactgtaga	1	TC17113	85	1	
15477	gaccttacct	1	C204903.1	70	1	
15477	gaccttacct	1	C604065.1	69	1	
15478	tcattatttt	1	TC17546	95	1	
15479	gtgttgatgt	1	CD171019	1	1	
15480	acatatcatc	1				no_annot
15481	taaggcttcg	1				no_annot
15482	cgggaaaaac	1	TC10827	62	3	similar to SP|P38062|AMP2_RAT Methionine aminopeptidase 2(MetAP 2) (Peptidase M 2) (Initiation factor 2 associated 67 kDa glycoprotein) (p67) (p67eIF2). [Rat] {Rattus norvegicus}, partial (76%)
15483	gtggtgttgc	1				no_annot
15484	gccgcggcgg	1				no_annot
15485	gactatcata	1	TC14688	20	0	
15486	cttgtgtgtg	1	CD097890	90	0	
15487	tgctcttgtc	1	AF217404|Sma.771	74	2	Endoplasmin
15488	gagttctagt	1	CD186769	61	0	
15489	aagatgttgc	1				no_annot
15490	gtactttctt	1				no_annot
15491	catttctcgg	1				no_annot
15492	taatgcatcc	1				no_annot
15493	ataacaacgg	1	TC18685	35	1	
15494	taatacggat	1	TC15904	55	2	

15495	tgtattgagg	1				no_annot
15496	tggtttagtt	1	TC18686	26	1	
15497	aagtcacatt	1	AI394915	36	2	
15498	ttatttgatc	1	TC11331	3	2	
15499	ttataaactg	1				no_annot
15500	gctacacaaa	1	TC7108	74	5	
15500	gctacacaaa	1	TC7108	74	1	
15500	gctacacaaa	1	CD153646	76	1	
15501	aatttcatag	1	CD133873	33	2	
15502	ataaagctgc	1				no_annot
15503	ttgatgaagc	1				no_annot
15504	aagacagttc	1				no_annot
15505	atcaacatct	1	C200208.1	83	0	
15505	atcaacatct	1	C300222.1	77	1	
15505	atcaacatct	1	C603438.1	43	1	
15505	atcaacatct	1	C716472.1	77	1	
15506	cgtactattg	1	CD178518	9	0	
15507	aagacagtta	1	TC11328	30	0	
15507	aagacagtta	1	CD163209	3	0	
15508	taatactact	1				no_annot
15509	gtgattttac	1				no_annot
15510	tttaaataaa	1	TC8871	75	0	
15510	tttaaataaa	1	TC9435	90	0	similar to GP|13702464|dbj|BAB43605.1||AP003137 SA2302 {Staphylococcus aureus subsp. aureus N315}, partial (6%)
15510	tttaaataaa	1	TC14720	96	0	
15510	tttaaataaa	1	TC16843	24	8	similar to GP|25989211|gb|AAL31707.1||AY061936 prophenoloxidase activating factor 3 {Bombyx mori}, partial (10%)
15510	tttaaataaa	1	CD076190	2	3	weakly similar to GP|6007612|gb|AAF00976.1|AF116341_1|AF116341 poly(A)-binding protein II {Drosophila melanogaster}, partial (39%)

15511	acgacgtggt	1				no_annot
15512	tttattagac	1	C300018.1	44	0	
15513	ataattacca	1	CD074565	78	0	
15513	ataattacca	1	CD156768	41	0	
15514	gtttcctgtg	1				no_annot
15515	caatatggtt	1				no_annot
15516	ttcgaaaatt	1	TC19590	38	0	
15516	ttcgaaaatt	1	AW186591	5	0	
15517	aattgccgat	1				no_annot
15518	ccgtcggtac	1				no_annot
15519	gcgaagtgtt	1	C300036.1	41	2	
15520	aaagcgaaaa	1	CD181067	44	3	
15521	tcaaaataat	1	CD195876	87	0	
15522	gagaaacgcc	1				no_annot
15523	ggcaatggaa	1				no_annot
15524	gtatatgaag	1	C316485.1	44	0	
15524	gtatatgaag	1	C703978.1	44	0	
15525	accgatagga	1	TC6901	21	2	similar to SP|P52742|Z135_HUMAN Zinc finger protein 135. [Human] {Homo sapiens}, partial (5%)
15525	accgatagga	1	TC6901	21	1	similar to SP|P52742|Z135_HUMAN Zinc finger protein 135. [Human] {Homo sapiens}, partial (5%)
15525	accgatagga	1	TC7023	29	2	similar to SP|P52742|Z135_HUMAN Zinc finger protein 135. [Human] {Homo sapiens}, partial (5%)
15525	accgatagga	1	TC7023	29	1	similar to SP|P52742|Z135_HUMAN Zinc finger protein 135. [Human] {Homo sapiens}, partial (5%)
15525	accgatagga	1	TC7023	29	0	similar to SP|P52742|Z135_HUMAN Zinc finger protein 135. [Human] {Homo sapiens}, partial (5%)
15525	accgatagga	1	TC7035	21	4	
15525	accgatagga	1	TC7035	21	3	
15525	accgatagga	1	TC7035	21	2	
15525	accgatagga	1	TC7035	21	1	
15525	accgatagga	1	TC7035	21	0	

15525	accgatagga	1	TC7241	1	4	
15525	accgatagga	1	TC7241	1	3	
15525	accgatagga	1	TC7241	1	2	
15525	accgatagga	1	TC7241	1	0	
15525	accgatagga	1	CD134801	10	1	
15525	accgatagga	1	CD142962	70	0	
15526	taatattatt	1				no_annot
15527	gatatctccc	1				no_annot
15528	ttcaggcttc	1				no_annot
15529	tggttgctcg	1	TC8323	47	0	similar to GP|414797|gb|AAA30697.1||L18966 pyruvate dehydrogenase phosphatase {Bos taurus}, partial (3%)
15530	tttataccac	1				no_annot
15531	acctctgtgt	1				no_annot
15532	cctatcctcc	1				no_annot
15533	agagtgtttc	1				no_annot
15534	aatgcattta	1				no_annot
15535	aatgaagtcc	1				no_annot
15536	atcaatatta	1	CD158745	34	0	
15537	aatgaagtca	1				no_annot
15538	cagaaattga	1	TC11929	7	3	
15539	aatagcttca	1				no_annot
15540	aatcgatcat	1				no_annot
15541	aaaccggtta	1	C201170.1	40	2	
15541	aaaccggtta	1	C602095.1	39	2	
15542	tccaaacttg	1	C301218.1	77	0	
15542	tccaaacttg	1	C703307.1	77	0	
15543	tatttttctg	1				no_annot
15544	ctatacgtga	1				no_annot
15545	taactgtaaa	1				no_annot
15546	tccacaatca	1				no_annot
15547	aattcaatta	1				no_annot

15548	tgttctggaa	1	CD094730	60	0	
15549	gttggagaaa	1				no_annot
15550	catctcaaga	1	TC15538	53	1	weakly similar to SP|P57076|CU59_HUMAN Protein C21orf59. [Human] {Homo sapiens}, partial (82%)
15551	cgttctggca	1	AY826981|Sma.4207	50	2	SARC
15552	atcagtccat	1				no_annot
15553	tgatcggttc	1				no_annot
15554	tttccccccc	1				no_annot
15555	atgtgaattt	1				no_annot
15556	tgatgtagac	1				no_annot
15557	cagaacaaac	1	gi|161041|gb|L07513.1|SCMMTEGANT	50	17	Schistosoma mansoni major tegumental antigen SM15 (GSM70) gene, complete cds
15557	cagaacaaac	1	gi|161041|gb|L07513.1|SCMMTEGANT	50	13	Schistosoma mansoni major tegumental antigen SM15 (GSM70) gene, complete cds
15557	cagaacaaac	1	gi|161041|gb|L07513.1|SCMMTEGANT	50	9	Schistosoma mansoni major tegumental antigen SM15 (GSM70) gene, complete cds
15558	cagaacaaaa	1	gi|161041|gb|L07513.1|SCMMTEGANT	66	5	Schistosoma mansoni major tegumental antigen SM15 (GSM70) gene, complete cds
15559	gttcacgtac	1				no_annot
15560	ttggaccaat	1	C202838.1	35	0	
15561	gctgttacaa	1	TC10536	21	1	similar to PIR|JC4277|JC4277 ribosomal protein L4, cytosolic [validated] - rat, partial (51%)
15562	aatgaagtat	1	C206250.1	84	3	
15562	aatgaagtat	1	C310035.1	93	0	
15562	aatgaagtat	1	C600919.1	84	3	
15562	aatgaagtat	1	C703133.1	93	0	
15563	gttatctatt	1	TC10613	51	6	weakly similar to GP|12006785|gb|AAG44917.1|AF290330_1|AF290330 phosphoglucomutase {Drosophila melanogaster}, partial (52%)
15564	aatagcttat	1				no_annot
15565	cccccgtaca	1				no_annot
15566	gccgtccagg	1				no_annot
15567	tttttctatg	1				no_annot

15568	tagcaactaa	1				no_annot
15569	ggatttaggt	1				no_annot
15570	tgtattgaca	1				no_annot
15571	cagcaactcc	1				no_annot
15572	acagtcactt	1				no_annot
15573	gatgcggttt	1				no_annot
15574	ggcaattaag	1				no_annot
15575	aatgaagtaa	1				no_annot
15576	acaccaacaa	1				no_annot
15577	catttttctt	1				no_annot
15578	gtaacctttg	1				no_annot
15579	taattcccaa	1				no_annot
15580	aacactgtaa	1	TC9972	54	0	
15581	actgttccct	1				no_annot
15582	gactagcgac	1				no_annot
15583	agatctgcag	1				no_annot
15584	tgagaaatga	1				no_annot
15585	cctatatcaa	1	BG931878	62	0	
15586	acaactattg	1				no_annot
15587	cgcatttatc	1				no_annot
15588	ttttaggctt	1				no_annot
15589	tgccaatcct	1	L36833|Sma.1189	21	4	(clone C5-1B3) phosphoglycerate kinase (PGK) gene
15590	tcattaatct	1				no_annot
15591	aacaggacaa	1				no_annot
15592	tttgtctagt	1	CD146894	93	0	
15593	gtacaagcaa	1	TC7494	63	0	
15593	gtacaagcaa	1	TC7496	58	0	
15594	aaaagcctac	1	TC11194	50	1	similar to GP|16769690|gb|AAL29064.1||AY061516 LD46870p {Drosophila melanogaster}, partial (46%)
15595	aaaatggata	1	TC14651	44	2	
15596	gcgaacaaag	1				no_annot

15597	caccgttccc	1				no_annot
15598	ggcatagttc	1				no_annot
15599	gggttaattt	1	TC9345	58	0	
15600	ttcattctgt	1				no_annot
15601	aatttcttga	1	TC10916	69	1	similar to GP|25990444|gb|AAN76523.1|AF383945_1|AF383945 MAGO protein {Gallus gallus}, complete
15601	aatttcttga	1	CD135958	61	0	homologue to GP|12802873|gb|AAK08102.1|AF338343_1|AF338343 mago nashi-like protein {Oryza sativa} [Oryza sativa (japonica cultivar-group)], partial (32%)
15602	gatgctcact	1				no_annot
15603	ttcttcagtt	1				no_annot
15604	tccgtgatat	1				no_annot
15605	ggcgaccatc	1				no_annot
15606	tttgtattaa	1				no_annot
15607	tggttctggt	1				no_annot
15608	gagagtaaat	1				no_annot
15609	catttctcac	1				no_annot
15610	gaaactagta	1				no_annot
15611	ttgatgaaat	1	TC13931	53	0	similar to SP|Q15647|CSN2_HUMAN COP9 signalosome complex subunit 2 (Signalosome subunit 2) (SGN2) (Thyroid receptor interacting protein 15) (TRIP15). [Mouse] {Mus musculus}, partial (89%)
15612	atgcttatta	1				no_annot
15613	aaaagatcac	1				no_annot
15614	tggttctggg	1	C201387.1	34	1	
15614	tggttctggg	1	C609801.1	34	1	
15615	catagatgag	1				no_annot
15616	acttataaaa	1				no_annot
15617	gagacttcca	1	C206454.1	85	0	
15618	cttttctata	1	CD118386	36	1	similar to SP|P51610|HFC1_HUMAN Host cell factor C1 (HCF) (VP16 accessory protein) (HFC1) (VCAF) (CFF). [Human] {Homo sapiens}, partial (7%)

15619	tgcaacttct	1	CD133415	83	1	
15619	tgcaacttct	1	CD153066	46	1	
15620	tactttgagc	1				no_annot
15621	tggtcctctt	1				no_annot
15622	aagagggtta	1				no_annot
15623	taatgcttgg	1				no_annot
15624	atgttgctct	1	CD145148	34	0	
15625	ggtcacaata	1				no_annot
15626	cactaatctg	1				no_annot
15627	acggttgtct	1	CD140563	60	1	
15628	tcagtattat	1	TC15978	83	0	weakly similar to GP|1354207|gb|AAB82061.1||U49453 rof1 {Arabidopsis thaliana}, partial (4%)
15629	aaatcgaagg	1	TC7492	66	0	
15630	tatatggtca	1				no_annot
15631	gaaacaccta	1				no_annot
15632	aacctgaatg	1	TC7048	72	1	weakly similar to GP|27497118|gb|AAO17319.1|AF465616_1|AF465616 death inducer with SAP domain DIS {Homo sapiens}, partial (6%)
15633	gtcctgagat	1				no_annot
15634	aacttatata	1				no_annot
15635	acgtcgaata	1				no_annot
15636	taagctaggc	1				no_annot
15637	tgctgtttgt	1	TC14209	17	0	
15638	tgcgtgtata	1	AI977019	74	0	
15639	ttagttgaat	1				no_annot
15640	caagatggcc	1	TC10626	16	2	weakly similar to GP|3142634|gb|AAC78612.1||AF063665 small nuclear ribonucleoprotein N {Mus musculus}, partial (36%)
15641	agctttctgc	1				no_annot
15642	gactctggcc	1				no_annot
15643	agttatgcct	1				no_annot
15644	ctggctaaac	1				no_annot

15645	aaaaatcagt	1				no_annot
15646	ccaacaagac	1				no_annot
15647	taacaaacga	1	CD115547	8	1	
15648	tcattcaaga	1	CD132010	51	1	similar to GP|19067879|gb|AAK14815.1||AY027869 polyprotein {Schistosoma japonicum}, partial (7%)
15649	aatgaacttt	1				no_annot
15650	caaatgtgtt	1				no_annot
15651	aacgtatagt	1	TC7599	68	2	PIR|A53153|A53153 glucose transport protein SGTP1 - fluke (Schistosoma mansoni), complete
15652	gttgatggca	1				no_annot
15653	agaaatgatc	1	TC15707	63	0	
15654	aggggggatc	1				no_annot
15655	gcatataagt	1	C607452.1	94	0	
