# Supplementary material for: A quantitative view of the transcriptome of Schistosoma mansoni adult-worms using SAGE
Source: BMC Genomics. 2007 Jun 21;8:186. doi: 10.1186/1471-2164-8-186 (PMC1914358; doi:10.1186/1471-2164-8-186)
Supplement: Additional file 2 — Protein and gene rank. Comparison of the top 10 proteins ranked by proteome analysis with the expression rank obtained by SAGE. [file 1471-2164-8-186-S2.pdf]

Table 2 (supplementary information) – Comparison of the top 10 proteins ranked by proteome analysis with the expression rank obtained by SAGE.

Protein/Gene	Protein rank	SAGE tag	Tags sequenced	Expression rank
14-3-3 homolog 1	1	TCATACAAGA	588	4
Cyclophilin	2	TTGTTTTCGG	100	59
GST28	3	TGACTGATCT	254	19
TPI	4	TCAGTTACTT	64	122
Fabp	5	TATCGTTCTA	302	15
Actin	6	ACATCAACAA	225	21
Ubiquitin	7	TGAATAAGTA	87	78
Fructose 1,6 bisphosphate aldolase	8	CGGCTCAGGA	398	9
GST26	9	GACTCACAGT	35	253
GAPDH	10	CATAATGAAG	438	8

Protein rank – stands for the list of most abundant identifiable proteins in the soluble transcriptome of S. mansoni adult worms, as identified by Curwen et al. (2004). Expression rank – stands for the expression level based on SAGE tags generated here.
